# Moulting and development in a freshwater prawn from the Cretaceous of Morocco

**DOI:** 10.7717/peerj.20463

**Published:** 2026-04-09

**Authors:** Sinéad Lynch, Pierre Gueriau, Harriet B. Drage, Didier B. Dutheil, Sylvain Charbonnier, Nora Corthésy, Javier Luque, Cristina Martin Olmos, Allison C. Daley

**Affiliations:** 1 Institute of Earth Sciences, Université de Lausanne, Lausanne, Switzerland; 2 IPANEMA, CNRS, UVSQ, MC, MNHN, Université Paris Saclay, Paris, France; 3 Sorbonne Université-MNHN-CNRS, Muséum National d’Histoire Naturelle, Centre de Recherche en Paléontologie—Paris (CR2P, UMR 7207), Paris, France; 4 Department of Zoology, Museum of Zoology, University of Cambridge, Cambridge, United Kingdom; 5 Center for Advanced Surface Analysis, Institute of Earth Sciences, Université de Lausanne, Lausanne, Switzerland; 6 School of Architecture Civil and Environmental Engineering, École Polytechnique Federale de Lausanne, Lausanne, Switzerland

**Keywords:** *Cretapenaeus berberus*, Cuticle microstructure, Moulting, Freshwater, Dendrobranchiata, Penaeidae

## Abstract

The lowermost Upper Cretaceous (Cenomanian) Kem Kem Group of Morocco is renowned for its rich fossil record, particularly that of macrofossils from freshwater facies, which includes insects, fish, and the penaeid prawn *Cretapenaeus berberus* Garassino, Pasini & Dutheil, 2006 (Dendrobranchiata: Penaeidae). Here, we provide information on growth in this extinct species, based on a sample of 65 specimens. These provide valuable insight into its moulting behaviour and development, while their exceptional preservation enables detailed examination of cuticle microstructure. Moults were identified based on a repeated pattern of preservation characterised by the absence of the carapace, as a result of exuviation, and/or the absence of muscles, which are exceptionally well-preserved in carcasses. Among the 65 specimens of *C. berberus*, only eight moults were identified. These moults reveal a similar moulting mechanism to extant penaeids. Scanning electron microscopy revealed a thicker inner endocuticle than outer exocuticle, with one specimen showing what is likely the membranous layer. Lamination is present in the exo- and endocuticle, with fewer lamination present in the exocuticle, and the endocuticle generally shows laminae that are more widely spaced in the outer layers. No difference in cuticle lamination, luminescence or reflection were observed between carcasses and moults. Carapace lengths range from ∼2 to 20 mm, and a detailed morphological analysis of the smallest specimens revealed that early larval stages (nauplius, protozoeae) were absent, with the smallest individual being either a late postlarva or a juvenile. Morphological changes likely occurred during development from juvenile to adult, with adults having a proportionally longer and more spinous rostrum and proportionally longer antennae and elongated fourth and fifth pereiopods. The adults also exhibit pleopods with more equally sized rami that are more curled, annulated, thinner distally than in juveniles. *C. berberus*, a rare representative of freshwater-dwellers among penaeids and dendrobranchiates, is assessed for its potential development strategies in freshwater environments, in comparison with other freshwater decapod taxa in Pleocyemata.

## Introduction

### Moulting in living and fossil decapod crustaceans

With over 15,000 extant species, decapod crustaceans, *i.e*, shrimps, prawns, lobsters, crabs, and relatives, are one of the most speciose, diverse, and ecologically important groups of aquatic arthropods (De Grave et al., 2009). The moult cycle is the succession of stages (intermoult, premoult, exuviation, postmoult) that describe a series of physiological, morphological, and behavioural transformations tied to the periodic replacement of the old exoskeleton (Drach, 1939; Passano, 1960; Skinner, 1962; Drach & Tchernigovtzeff, 1967). The composition and structure of the cuticle change throughout this moult cycle (Roer & Dillaman, 1984; Roer & Dillaman, 1993). During the intermoult stage, the crustacean cuticle is composed of four layers, which, from internal to external, consist of the membranous layer, the endocuticle, the exocuticle, and the epicuticle. The following premoult stage is characterised by the separation of the cuticle from the underlying hypodermis (apolysis) and the dissolution of the membranous layer. Minerals and organics are partially resorbed from the old cuticle, and the new epi- and exocuticle are formed. Only then does the physical act of exiting the old exoskeleton occur, called exuviation. Then, during the postmoult stage, the epi- and exocuticle are calcified, and the new endocuticle and membranous layer are deposited (Roer & Dillaman, 1984; Roer & Dillaman, 1993). The thickness and relative proportions of the exo- and endo- cuticle layers can vary throughout the moult cycle, as shown in the penaeid *Penaeus monodon* (Fabricius, 1798; Promwikorn, Boonyoung & Kirirat, 2005). In extant decapods, morphological changes through the moult cycle can be recorded to infer moult cycle stage, typically changes to “setae” of the uropod (Mills & Lake, 1975; Burton & Mitchell, 1987; Dall et al., 1990a; Gorissen & Sandeman, 2022; Kim et al., 2024). Occasionally, setal development is associated with other morphological changes throughout the moult cycle for moult staging, such as changes in cuticle texture (*e.g.,* Dall et al., 1990a: table 6.1). Before the new exoskeleton of arthropods fully hardens, it can be soft and wrinkled (Drach, 1939; Daley & Drage, 2016).

Although, to our knowledge, decapods have not yet been found fossilised in the act of moulting, rare examples of stem euarthropods were fossilised during exuviation, as they were exiting their old exoskeleton (García-Bellido & Collins, 2004; Yang et al., 2019). Nevertheless, in some cases, decapods fossils from Solnhofen can preserve exuviae together with traces that are directly associated with moulting itself (Schweigert, 2007). More often, empty, discarded exuviae have been identified based on repeated disarticulation patterns, because when moulting occurs the old exoskeleton separates along specific lines of weakness that leave behind characteristic assemblages of exoskeleton sclerites (Daley & Drage, 2016). Fossils of carcasses preserved immediately after moulting can also be identified based on characteristic features. For example, postmoult trilobites were recognised by their wrinkled, flattened, and thinner exoskeletons compared with intermoult carcasses and exuviae (Drage et al., 2019).

In decapods, fossil moults can be identified because of their characteristic disarticulation patterns (Schweigert, 2011; Charbonnier, Pérès & Letenneur, 2012; Charbonnier & Garassino, 2012; Charbonnier, Audo & Garassino, 2017; Chény, Charbonnier & Audo, 2023), which are similar to those of extant decapods. Moulting typically results in the carapace being displaced at an angle relative to the sternum and the pleon (=abdomen), a posture preserved in some decapod fossil moults and referred to as Salter’s position (Glaessner, 1929; Glaessner, 1969; Schäfer, 1951; Bishop, 1986; Daley & Drage, 2016). However, this posture is not always indicative of a moult, as decomposition can cause the planes of weakness to open on carcasses (Bishop, 1986; Feldmann & Tshudy, 1987) and physical disturbances, such as the action of waves, currents, or scavengers can displace the carapace or other exoskeleton elements. For this reason, the identification of quiet depositional settings is crucial for identifying fossil moults (Daley & Drage, 2016). Examples of decapod fossils preserved in Salter’s position include brachyuran crabs and lobsters (Bishop, 1986). Similarly, mudlobster fossils may also display a dorsal midline plate of the carapace at an angle to the carapace (Murray & Hanley, 1986). In decapod fossils, the cuticle structure can also be used to differentiate moults from carcasses. Moults of Cretaceous nephropid lobsters could be differentiated from carcasses because they had lost the lamination of the inner endocuticle (Feldmann & Tshudy, 1987). Conversely, carcasses can also be recognised, for example numerous glypheoids from the Cretaceous of Mexico were interpreted as being carcasses due to their complete cuticle, the good preservation of the carapace, and its articulation with appendages (González-León et al., 2020).

### Development and mode of life in prawns (Dendrobranchiata)

Decapods are generally divided into two main monophyletic sister groups: the Dendrobranchiata Bate, 1888, and the Pleocyemata Burkenroad, 1963. Members of Dendrobranchiata do not brood their eggs but release them directly into the water, which subsequently hatch as a nauplius larva (except for luciferids, which carry their eggs for a short time) (Tavares & Martin, 2010; Martin, Criales & Santos, 2014; Olesen, 2018). Typically, morphological changes between successive larval stages are considered to be more gradual in Dendrobranchiata than in Pleocyemata (Anger, 2001; Tavares & Martin, 2010; Martin, Criales & Santos, 2014; Olesen, 2018).

Within the suborder Dendrobranchiata, *Cretapenaeus berberus* Garassino, Pasini & Dutheil, 2006 belongs to the family Penaeidae Rafinesque, 1815, commonly known as prawns (Garassino, Pasini & Dutheil, 2006). The larval development of Dendrobranchiata comprises the following main stages: nauplius, zoea (protozoea and mysis) and postlarva (or decapodid) (Tavares & Martin, 2010; Martin, Criales & Santos, 2014; Olesen, 2018). Although the number of substages within each main stage can vary greatly (Dall et al., 1990b: table 3.2), penaeids generally have five or six nauplii, six zoeae (with three protozoeae and three mysis), and three to six postlarval stages. In penaeids, the nauplius, protozoea, and mysis are pelagic, the postlarval stage spans both late pelagic and early benthic phases, and juveniles and adults are benthic (Dall et al., 1990c; Dall et al., 1990b).

Many morphological characteristics of penaeid developmental stages were documented by Dall et al. (1990d: see also their summary in table 2.2), some of which are summarised here. The nauplius (Dall et al., 1990d: fig. 2.34) lacks thoracic somites, swims using cephalic appendages, has a single naupliar eye and does not feed. The protozoea (Dall et al., 1990d: fig. 2.35) has thoracic somites and its carapace partly covers the thorax. During this stage, the thoracopods become prominent and biramous, the rostrum and supra-orbital spine appear, the pleon shows visible segmentation, biramous uropods appear and the telson becomes separated from the pleon. In the mysis stage (Dall et al., 1990d: fig. 2.36A), functional pereiopods with large exopods develop, the carapace covers most thoracic somites, rostral spines appear, and rudimentary chelae appear on the first three pereiopods. The mysis substages are divided based on changes in pleopod morphology. In the first mysis, pleopod buds are small or absent, and in the second mysis, the pleopod buds are prominent but unsegmented. In the third mysis, the pleopods are small and divided into two segments. Several morphological features define the postlarval stage (Dall et al., 1990d: fig. 2.36B), including the presence of large, setose uniramous pleopods, the absence of supra-orbital spines, segmentation of the distal rami of the first antennae, reduced size and setosity of the exopods of the pereiopods, and a squarish telson (Dall et al., 1990d). The transition from the postlarval to juvenile stage is marked by a reduction in telson spines, a shift in telson shape from squarish to V-shape, an increase in chromatophores, a “bulkier” body appearance, and the development of adult rostral and branchial features (Dall et al., 1990b, p. 114). After their larval development penaeids moult frequently, every few days or weeks (Dall et al., 1990a; Corteel et al., 2012).

Extant dendrobranchiates are mostly marine, except for the sergestid *Acetes paraguayensis* (Hansen, 1919), known from the Guyana Shield (*e.g.,* Brazil, Colombia, Venezuela, Suriname, Peru, Argentina) (D’Incao & Martins, 2000; Magalhães & Pereira, 2007; Tavares & Martin, 2010; Pileggi et al., 2013; Vereshchaka, Lunina & Olesen, 2016), and a few other species that have juvenile stages occupying estuarine and brackish water, although none are truly freshwater (Bauer, 2023). Among the freshwater decapod fossils listed by Garassino, Pasini & Dutheil (2006), all belong to Pleocyemata, except for *Paleomattea deliciosa* (Maisey & Carvalho, 1995), which was subsequently reassigned to the Sergestidae (Dana, 1852a) in Dendrobranchiata (Schweitzer et al., 2010). Subsequently, a mid-Cretaceous freshwater prawn, *Xiaopenaeus electrinus* (Xing et al., 2021), was described from Burmese amber deposits associated with numerous freshwater penny beetle larvae (family Psephenidae) (Xing et al., 2021). *C. berberus* is, therefore, one of only three documented fossil taxa of freshwater Dendrobranchiata and one of two known extinct representatives of freshwater Penaeidae, with no known extant representatives of the family being freshwater-dwellers (Garassino, Pasini & Dutheil, 2006).

In extant Dendrobranchiata, the developmental pattern of the freshwater sergestid *Acetes paraguayensis* (D’Incao & Martins, 2000; Magalhães & Pereira, 2007; Tavares & Martin, 2010; Vereshchaka, Lunina & Olesen, 2016) remains undescribed. Primary freshwater dwellers are more common in the suborder Pleocyemata, the sister group to Dendrobranchiata, in which members of four major groups, *i.e*., Caridea (shrimps) (Dana, 1852b), Astacidea (Latreille, 1802) (crayfishes), Anomura (MacLeay, 1838) (‘false’ crabs) and Brachyura (Latreille, 1802) (true crabs), have colonised freshwater multiple times (Bond-Buckup et al., 2008; Crandall & Buhay, 2008; De Grave, Cai & Anker, 2008; Yeo et al., 2008; Vogt, 2013; Yeo, Cumberlidge & Klaus, 2014; Robin et al., 2019; Luque et al., 2021). Due to the differences between freshwater and marine environments, such as physico-chemical conditions and plankton availability, freshwater pleocyematans typically display two distinct developmental patterns after hatching (Jalihal, Sankolli & Shenoy, 1993; Bauer, 2011a; Bauer, 2011b; Bauer, 2013; Anger, 2016). In the first pattern, the planktonic larval stage takes place in salt or brackish waters, and then migration to and from freshwater takes place at different life stages (referred to as diadromous, as in Anger, 2016; Bauer, 2013, or amphidromous, for example, in Bauer, 2011a; Bauer, 2011b). In the second pattern, they undergo a shorter larval development, ranging from abbreviated (with fewer larval stages) to fully direct development (hatching directly as juveniles) (Jalihal, Sankolli & Shenoy, 1993; Anger, 2016). More rarely, some decapods undergo extended larval development in freshwater environments, under certain conditions such as the presence of sufficient plankton, and early larval stages that are less or not dependent on food and possess the osmoregulatory abilities needed for life in freshwater (Anger, 2016).

### The Cretaceous freshwater prawn *Cretapenaeus berberus*

**Figure 1:**
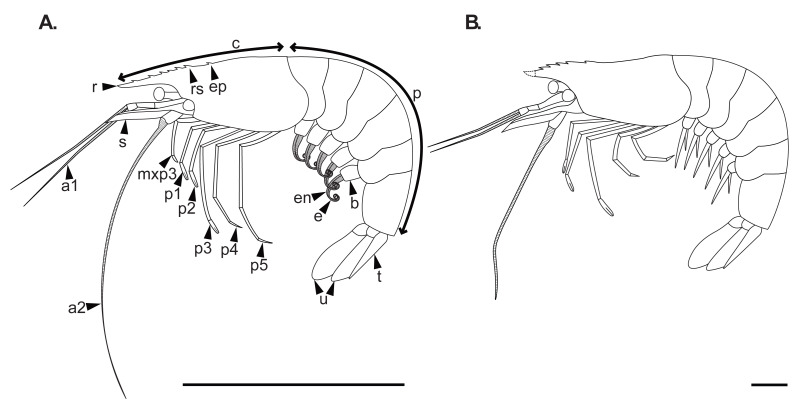
Reconstructions of *Cretapenaeus berberus*, highlighting key morphological differences between adults (A) and juveniles (B). The juvenile reconstruction is primarily based on specimen MHNM-KK-OT 79, with additional features drawn from other juvenile specimens (mainly MHNM-KK-OT 92 and 93). The very tip of the rostrum is not visible in any of the specimens and is therefore represented by a dotted line. The adult reconstruction is mainly derived from MHNM-KK-OT 75, supplemented by the additional new specimens presented in this study and the descriptions and reconstructions by Garassino, Pasini & Dutheil (2006). The third maxilliped was observed only in adult specimens. Some proximal pereiopods segments are obscured by the carapace, which likely accounts for certain proportional length differences compared to Garassino, Pasini & Dutheil (2006: fig.7). Scale bars equal five mm. a1, antennule; a2, antenna; b, pleopod basis; c, cephalothorax; e, exopod (pleopod ramus); en, endopod (pleopod ramus); ep, epigastric tooth; mxp3, third maxilliped; p, pleon; p1–5, pereiopod 1–5; r, rostrum; rs, rostral spine; s, scaphocerite; t, telson; u, uropod. Line drawings: S. Lynch.

The penaeid *C. berberus* (Fig. 1) was described by Garassino, Pasini & Dutheil (2006) from the freshwater Jbel Oum Tkout (OT1) locality in Morocco. Specimens of *C. berberus* are exceptionally well-preserved with phosphatised cuticle and soft tissues, including delicate structures such as the muscular fibres (Garassino, Pasini & Dutheil, 2006: fig. 5). The preserved cuticle and soft tissues are composed of nanometric crystallites of francolite (<30 nm) (Gueriau et al., 2020) that incorporated various trace elements during diagenesis, including arsenic, rare earth elements, strontium and thorium (Gueriau et al., 2014; Gueriau et al., 2020). Dark red regions of the fossils have been identified as iron hydroxides that contain neodymium and lead (Gueriau et al., 2014; Celeux et al., 2022). These areas have been interpreted as microbial mats, which were present both as extensive coverings of sedimentary surfaces throughout the outcrop, and as biofilms around and within the phosphatised soft tissues of the fossils (Gueriau et al., 2020). The preservation model of this locality proposes that iron hydroxides precipitated at the same time as phosphatisation of soft tissues, and that both processes occurred in slightly oxic conditions, which supported living microbial mats that covered the recently deceased carcasses and mediated fossil preservation (Gueriau et al., 2020).

*Cretapenaeus berberus* is one of the few freshwater penaeid and dendrobranchiates known, extinct or extant, offering a rare opportunity to investigate developmental strategies in freshwater environments in deep time. This study expands on Garassino, Pasini & Dutheil (2006) by examining previously undescribed smaller specimens and newly discovered smaller and larger specimens of *C. berberus* that offer insights into the morphological development and moulting of this species, and their exceptional preservation allows a detailed examination of cuticular microstructures. With a dataset of 65 specimens of *C. berberus* from the lowermost Upper Cretaceous (Cenomanian) Kem Kem Group of Morocco and careful consideration of the taphonomic context, we distinguish empty moults from carcasses to infer moulting behaviour, and investigate differences in the cuticle composition and microstructure between moults and carcasses. We characterise the developmental stages of *C. berberus*, describe morphological changes throughout its development, and draw comparisons with the larval development of extant freshwater decapods.

## Materials & Methods

The dataset analysed consists of 65 specimens of *C. berberus* from the Jbel Oum Tkout (OT1) locality in Morocco (Garassino, Pasini & Dutheil, 2006: figs. 1–3). 31 specimens were collected between 1999 and 2002, of which 17 are housed at the Muséum national d’Histoire naturelle (MNHN, Paris, France) including the holotype and paratypes, 3 paratypes at the Museo Civico di Storia Naturale (MSNM, Milan, Italy), and 11 at the Museum of Natural History of Marrakesh (MHNM-KK-OT, Marrakesh, Morocco). Details of the fieldwork and collection campaigns, for which all necessary permits were obtained, can be found in Ibrahim et al. (2020), which in particular highlights the long-standing collaborations between several Moroccan universities, the Museo Civico di Storia Naturale, Milan, Italy, and the Muséum national d’Histoire naturelle, Paris, France. The remaining 34 specimens were collected in 2012 and are also accessioned at the Museum of Natural History of Marrakesh, Morocco. For the material collected in 2012, all required permits were obtained, ensuring full adherence with applicable regulations, by the Moroccan Ministère de l’Énergie, des Mines, de l’Eau et de l’Environnement. The collection is temporarily housed in the Muséum national d’Histoire naturelle for analysis under an agreement between the MNHN and the MHNM, and specimens will be returned to the MHNM following completion of study. A thorough effort was made to collect all visible specimens in the field, including those of very small size. Accession number, measurements, moult/carcass assignment, moult evidence type, and developmental stage assignment are available for each specimen in Supplemental Information 1. All supplementary materials are accessible on the Open Science Foundation (https://osf.io/zdhuv/). Only specimens with sufficient morphological information to be confidently assigned to *C. berberus* were included in this study. We provide a detailed assessment of the morphological characters supporting the original assignment to Penaeidae by Garassino, Pasini & Dutheil (2006), based on the key of the Penaeoidea of Farfante & Kensley (1997), in Supplemental Information 2.

### Geological setting

The specimens were collected from clay beds of the Jbel Oum Tkout (OT1) locality, located in the Errachidia Province of southeastern Morocco, approximately 10 km south of Tafraoute Sidi Ali. Fossils derive from within the Douira Formation of the Kem Kem Group, and are dated to a Late Cretaceous (Cenomanian) age (Sereno et al., 1996; Dutheil, 1999; Ibrahim et al., 2020). The presence of insect larvae and unionid bivalves that are restricted to freshwater, the absence of marine organisms, and the composition of the fish and flora, all indicate a freshwater habitat for the OT1 locality (Garassino, Pasini & Dutheil, 2006). Palaeontological and sedimentological data suggest that the depositional environment was a low-energy seasonally dried freshwater setting, such as an oxbow lake or other small pool or pond (Brito et al., 2024). During the Cenomanian, the basin where the Kem Kem Group was deposited, was situated near the northwestern margin of the African continent, forming the headland of a vast river system that flowed northwards for ∼250 km towards the Tethys Ocean (Ibrahim et al., 2020: fig. 33).

**Figure 2:**
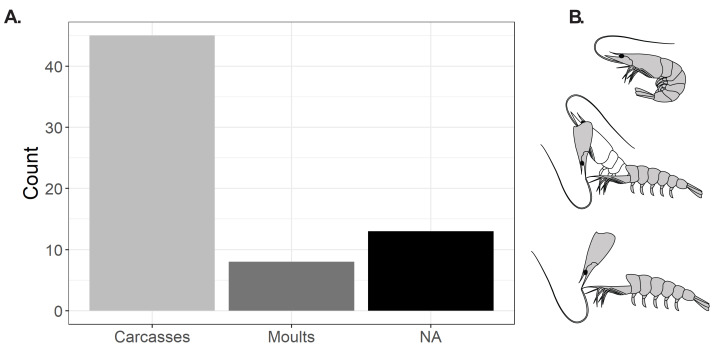
*Cretapenaeus berberus* from the Late Cretaceous of Jbel Oum Tkout, Morocco. (A) Proportion of carcasses and moults. (B) Schematic drawing of moulting, showing three sequential stages from top to bottom: before moulting, in the act of moulting (exuviation), and the empty moult left behind after moulting. Line drawings: S. Lynch.

**Figure 3:**
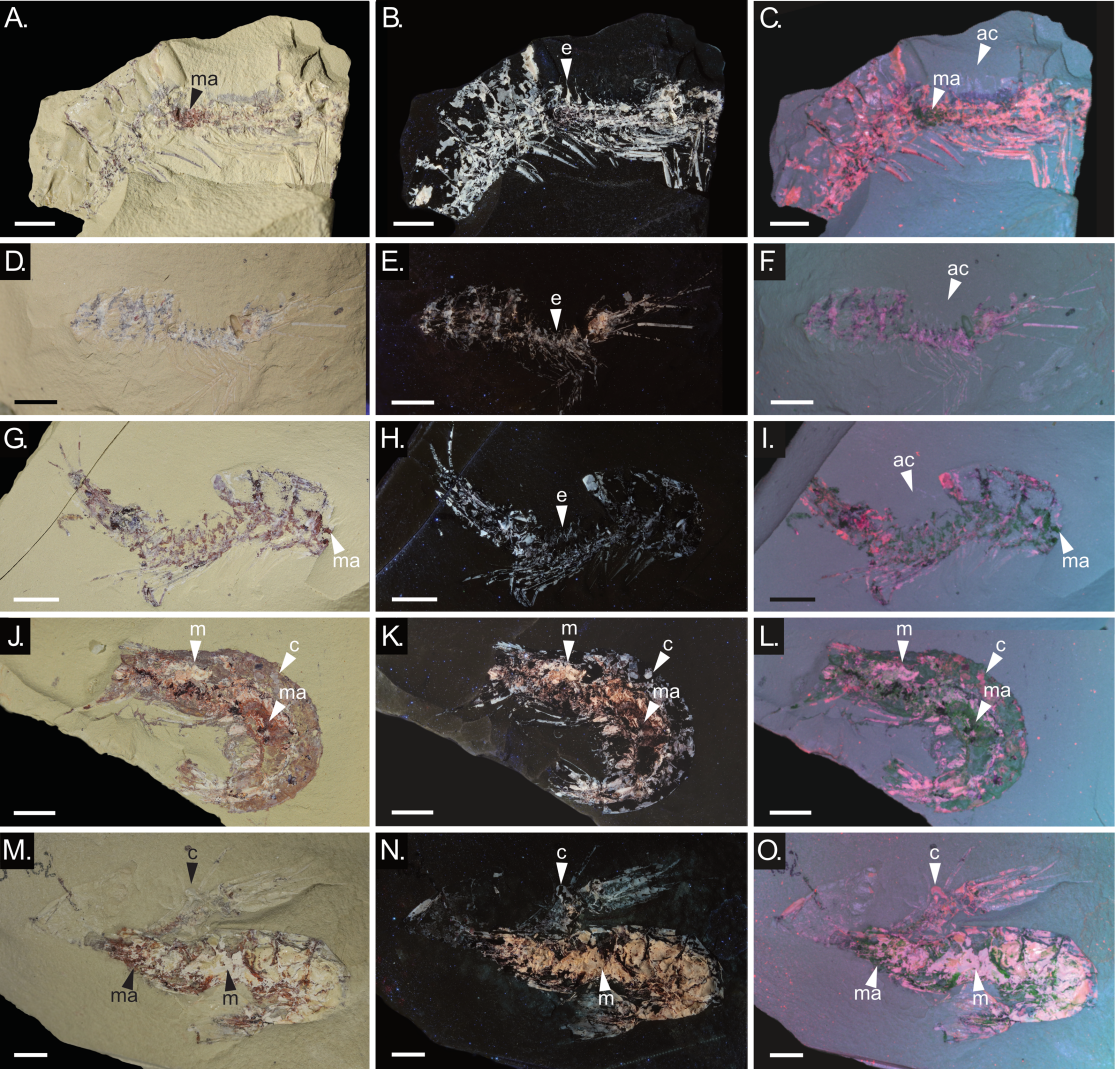
Moults and carcasses of *Cretapenaeus berberus* exhibiting different preservation, with the moults preserving only cuticle and the carcasses both cuticle and muscles. Photographs taken under visible light illumination (left column), UV illumination (middle column), and false-colour images generated from multispectral imaging (right column). (A–C) Moult MNHN.F.A88748. (D–F) Moult MHNM-KK-OT 64b. (G–I) Moult MHNM-KK-OT 82b. (J–L) Carcass MHNM-KK-OT 78a. (M–O) Specimen consisting of a moult (MHNM-KK-OT 87-2), located above the carcass of another individual (MHNM-KK-OT 87-1). Scale bars equal five mm. c, cuticle; ac, absent carapace; e, endophragmal element; m, muscles; ma, microbial mat. Photographs: P. Gueriau; S. Lynch.

### Moult/carcass assessment

We distinguished moults from carcasses of *C. berberus* by analysis of the following two morphological characteristics, recording uncertain cases as NA:

1. Moults may have an absent carapace, as a result of its displacement during exuviation (see Figs. 2B, 3A–3I).
2. Carcasses show the presence of well-preserved muscles (see muscular fibres in Garassino, Pasini & Dutheil, 2006: fig. 5). Moults lack muscles and only preserve the cuticle (see Figs. 3A–3I, 3M–3O).

Based on these identifications, we further investigated differences between moults and carcasses using multispectral imaging and spectroscopy, as well as scanning electron microscopy, described in detail below.

### Developmental stage assessment

Early larval stages (nauplius, zoeae) are absent from the sample. Specimens were classified as postlarvae, juveniles, intermediates and adults. This classification was based on detailed morphological analysis, comparing features such as the pereiopods, pleopods, and rostrum with documented developmental sequences of extant penaeids (Dobkin, 1961; Rao, 1973; Motoh & Buri, 1980a; Motoh & Buri, 1980b; Hassan, 1982; Paulinose, 1986; Jackson et al., 1989; Dall et al., 1990d; Dall et al., 1990b; Ronquillo & Saisho, 1997; Choi & Hong, 2001; Türkmen, 2005; Ronquillo, Saisho & McKinley, 2006).

### Carapace measurements and analyses

Measurements of the carapace length (CL) and maximum height (CMH) were taken for 26 specimens, with both measurements possible on 25 specimens. CL is the linear distance between the dorsal projection of the posterior rim of the orbit to the dorsal distal extremity of the carapace. Measurements were made on scaled digital images of specimens using Adobe Photoshop. A simple linear regression model for CL and CMH was fitted using R (version 4.2.1), and the graphics were also produced in R, using the ggplot2 package (version 3.4.4) (Wickham, 2016; R Core Team, 2022). The R script is available in the Supplemental Information 3.

### Photography and figures

Specimens were photographed under visible and UV light using a Canon camera (EOS-800D) equipped with a macro lens (EFS 60 mm 1:2.8) and the EOS Utility photo software. UV light was applied with a U1c 6W 365 nm UV LED flashlight (JAXMAN). Digital microscopy images were taken with a Keyence microscope (VHX-7000) fitted with a dual objective zoom lens (VH-ZST), using the ZS-20 macro lens (20-200x). Photo stacking and editing of images (such as removing background and adjusting brightness and contrast) were accomplished using Adobe Photoshop and Affinity Photo. Figures were created in Adobe Illustrator and Affinity Designer, with additional luminosity and contrast adjustments applied to selected images.

Specimen MSNM i26605 from the Museo Civico di Storia Naturale, Milano (Italy) in Supplemental Information 2 was photographed with a Canon PowerShot S50. Specimens MNHN.F.A24633b (holotype) and MNHN.F.A24651 from the Muséum national d’Histoire naturelle, Paris (France) Supplemental Information 2 were photographed by Lilian Cazes using a Nikon D800. Specimen MNHN.F.A24651 in Supplemental Information 2 were photographed by Peter Massicard (RECOLNAT, ANR-11-INBS-0004) with a Canon EOS 60D.

### Multispectral imaging and spectroscopy

Compositional differences between specimens designated as moults and carcasses (see the Moult/carcass assessment section) were assessed using multispectral imaging and spectroscopy. Multispectral imaging is based on the premise that variations in compositions of different parts of fossils can result in distinct reflections or luminescence (Brayard et al., 2019; Robin et al., 2021). It employs a broad spectrum of light wavelengths from UV-A to the near-infrared (NIR) to enhance contrast within the fossil remains. Each selected carcass (MHNM-KK-OT 75a, 60a, 78a, MHNM-KK-OT 87-1) and moult (MNHN.F.A88748, MHNM-KK-OT 82b, 64b, MHNM-KK-OT 87-2, MHNM-KK-OT 88-2) was imaged under the same conditions (*i.e*, same distance from the camera, same LED intensity, same exposure time for three illumination-emission wavelength combinations: 385 nm 472 ± 15 nm (luminescence), 660 nm/719 ± 30 nm (reflectance + luminescence) and 460 nm/472 ± 15 nm (reflectance), which each produce a grayscale image). For each specimen, the three resulting greyscale images were combined into false-colour RGB overlays (red: illumination 385 nm/detection 472 ± 15 nm, green: 660 nm/719 ± 30 nm, and blue: 460 nm/472 ± 15 nm) using ImageJ software (W. S. Rasband, US National Institutes of Health, Bethesda, MD, http://imagej.nih.gov/ij, 1997–2014), using the same processing for each specimen (*i.e*, same false-colour overlay, same minimum and maximum intensity values). The luminosity was further adjusted in Fig. 3 using Affinity, but the raw images of these stacks and a tiff file containing the raw grayscale images are available in Supplemental Information 4.

Following the method described in Mayo de la Iglesia et al. (2025), UV–vis–NIR emission spectra were obtained from the cuticle of moults as well as the cuticle and muscle tissue of carcasses with a spectroradiometer Specbos 1211UV (JETI). UV light was emitted with UV LED flashlight (U1c, JAXMAN, 6W, 365 nm). To filter any diffuse reflection caused by excitation of the sample, a long-pass optical filter with a wavelength cutoff of 410 nm was positioned in front of the detector. Spectral data, up to a maximum of 1,000 nm, were obtained from an area of ∼1 mm^2^, precisely targeted with the spectroradiometer’s built-in spot laser. Spectra were normalised to their maximum intensity for comparison and plotted using R and ggplot 2 (Wickham, 2016; R Core Team, 2022). The raw spectral data and the R script are available respectively in Supplemental Informations 4 and 5.

### Scanning electron microscopy

Images of the cuticle macrostructure were captured using a Zeiss Gemini 500 scanning electron microscope (SEM) in variable pressure (environmental) mode on uncoated specimens. The SEM was operated with the backscattered electron detector (BSD), at an electron high tension voltage (EHT) set to 15 kV, an aperture of 60 µm and the chamber pressure maintained at 40 Pa during imaging. Six specimens were scanned for cuticle microstructure (moults: MHNM-KK-OT 87-2, MHNM-KK-OT 82a, 56a; carcasses: MHNM-KK-OT 87-1, MHNM-KK-OT 86b, 83b). Cuticle lamination was examined across the body wherever it was visibly present on randomly distributed rock breakage surfaces. Two smaller specimens (either late postlarvae or juveniles) were scanned for morphological details of this stage (MHNM-KK-OT 80ab, MHNM-KK-OT 70ab-1). The BSE-EDS calcium map of one specimen (MHNM-KK-OT 61b) was generated using the same settings as described above, with the Oxford AZtec Microanalysis System (Oxford X-max 150 detector, software version 4.2 SP1; Oxford Instruments, High Wycombe, UK).

## Results

### Moulting

Carcasses are more numerous (45) than moults (8) (with 12 specimens not assignable) (Fig. 2A). Carapace measurements were not available for moults, as the carapace is either not exposed, broken or absent. Only moults from larger specimens were found, with the smallest one being an incomplete moult (MHNM-KK-OT 90-2) measuring ∼20 mm anteroposteriorly based on the preserved portion, and the largest specimen exposing only a partial pleon and telson of ∼45 mm anteroposteriorly (MHNM-KK-OT 88-2). Five moults (MHNM-KK-OT 56, 84, 87-2, 90-2, 88-2) were identified based only on the absence of muscles, and three moults (MHNM-KK-OT 64, 82, MNHN.F.A88748) were identified because of a missing carapace and absent muscles. Laterally preserved moults with a visibly absent carapace (MNHN.F.A88748, MHNM-KK-OT 82, 64) have visible endophragmal elements (Figs. 3B, 3E, 3H). Two moults of *C. berberus* with an absent carapace (MHNM-KK-OT 82, 64) exhibit only the sternum (ventral part of cephalothorax with pereiopods) and the cephalic appendages (Figs. 3D–3I). The carapace of moult MNHN.F.A88748 is also disarticulated, but due to the specimen’s anterior breakage it is impossible to determine how much of the carapace, if any, remained attached to the sternum (Figs. 3A–3C). Two other moults (MHNM-KK-OT 87-2, 90-2) also retain at least part of the cephalothorax. However, their orientation in a ventral-up position makes it difficult to determine whether the carapace is present or not (Figs. 3M–3O).

Under visible light, the muscles of *C. berberus* appear typically pink, and the microbial mats dark red. The cuticle is generally transparent, but can be slightly pink in thicker areas (Figs. 3A, 3D, 3G, 3J, 3M). Both the cuticle and muscles luminesce under UV light (Figs. 3B, 3E, 3H, 3K, 3N), notably enhancing the visibility of the cuticle, and thereby making moults more visible. In the false-colour RGB overlays generated from multispectral imaging, muscles and cuticle appear pink to purple, while microbial mats appear green (Figs. 3C, 3F, 3I, 3L, 3O). Even when using alternative illumination–emission wavelength combinations, multispectral imaging does not reveal differences between moults and carcasses. Microbial mats are present both on moults and carcasses. Some carcasses have only patchy microbial mat remains (*e.g.,* MHNM-KK-OT 87-1, Figs. 3M–3O), while in others they are more extensive and form an outline of the fossil where muscles and cuticle are either not preserved or detached (*e.g.,* MHNM-KK-OT 78, Figs. 3J–3L). Moult MHNM-KK-OT 82 shows the largest microbial mat coverage (Figs. 3G–3I), although it is not as extensive as seen in some carcasses (*e.g.,* MHNM-KK-OT 78, Figs. 3J–3L). Generally, only patches of microbial mats are visible on moults (Figs. 3A–3C).

**Figure 4:**
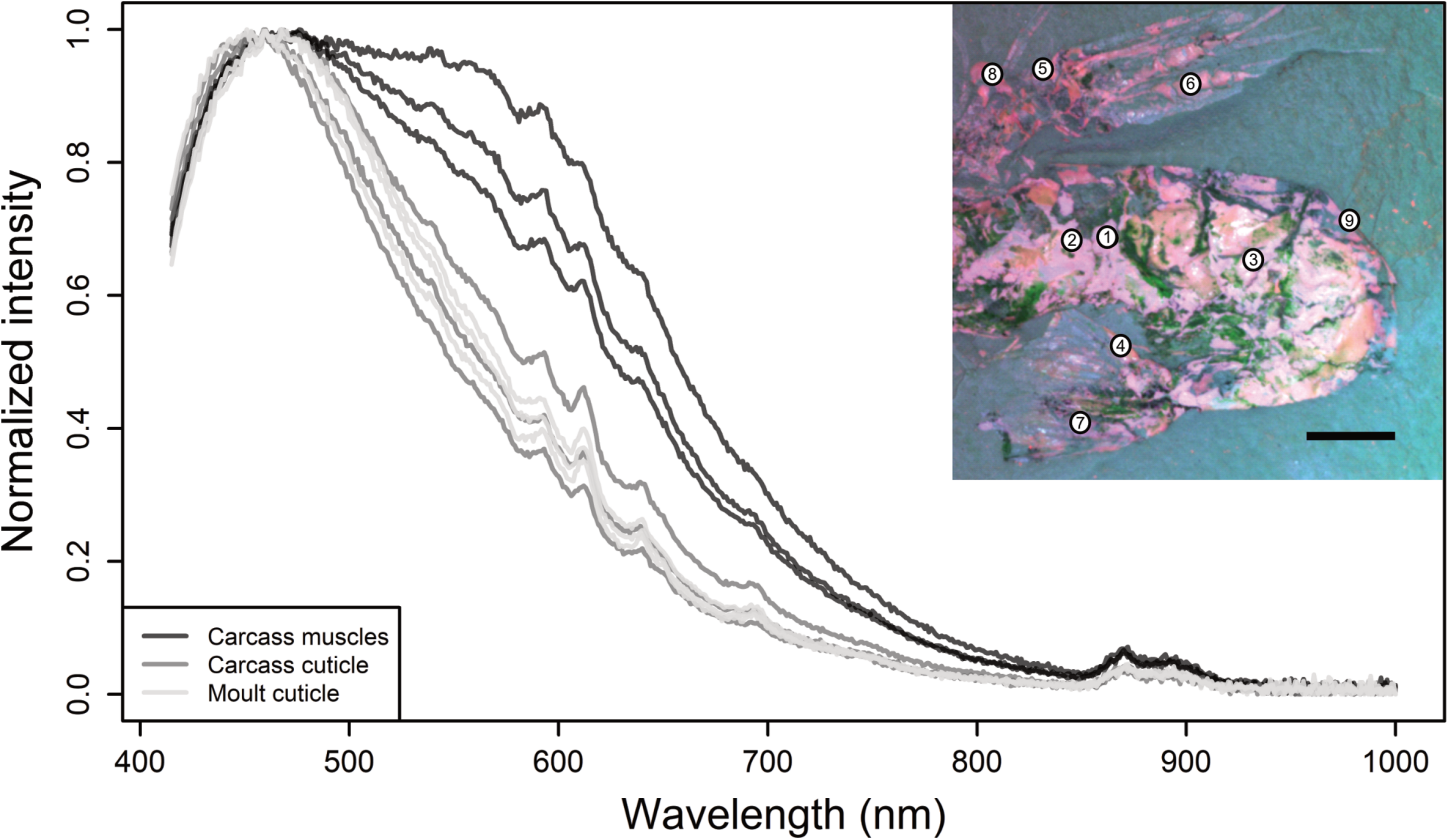
UV–vis–NIR emission spectra of the cuticle of a moult (MHNM-KK-OT 87-2) and cuticle and muscles of a carcass (MHNM-KK- OT 87-1) of *Cretapenaeus berberus*. Spectra (from top to bottom) were collected from the numbered analysis areas 1–9 marked on the inset false-colour image generated from multispectral imaging (Scale bar equals five mm).

As with multispectral imaging, no difference in luminescence could be detected between moult and carcass cuticle using UV−vis−NIR emission spectroscopy (Fig. 4; Supplemental Informations 5–6). Muscles, however, exhibit a distinct luminescence spectrum from that of cuticle, in the form of an additional broad emission band between 500 and 700 nm. This difference in luminescence between cuticle and muscles, combined with the fact that spectra obtained from all areas of the moults are identical to those of the cuticle in carcasses, confirms the absence of muscles in specimens interpreted as moults.

**Figure 5:**
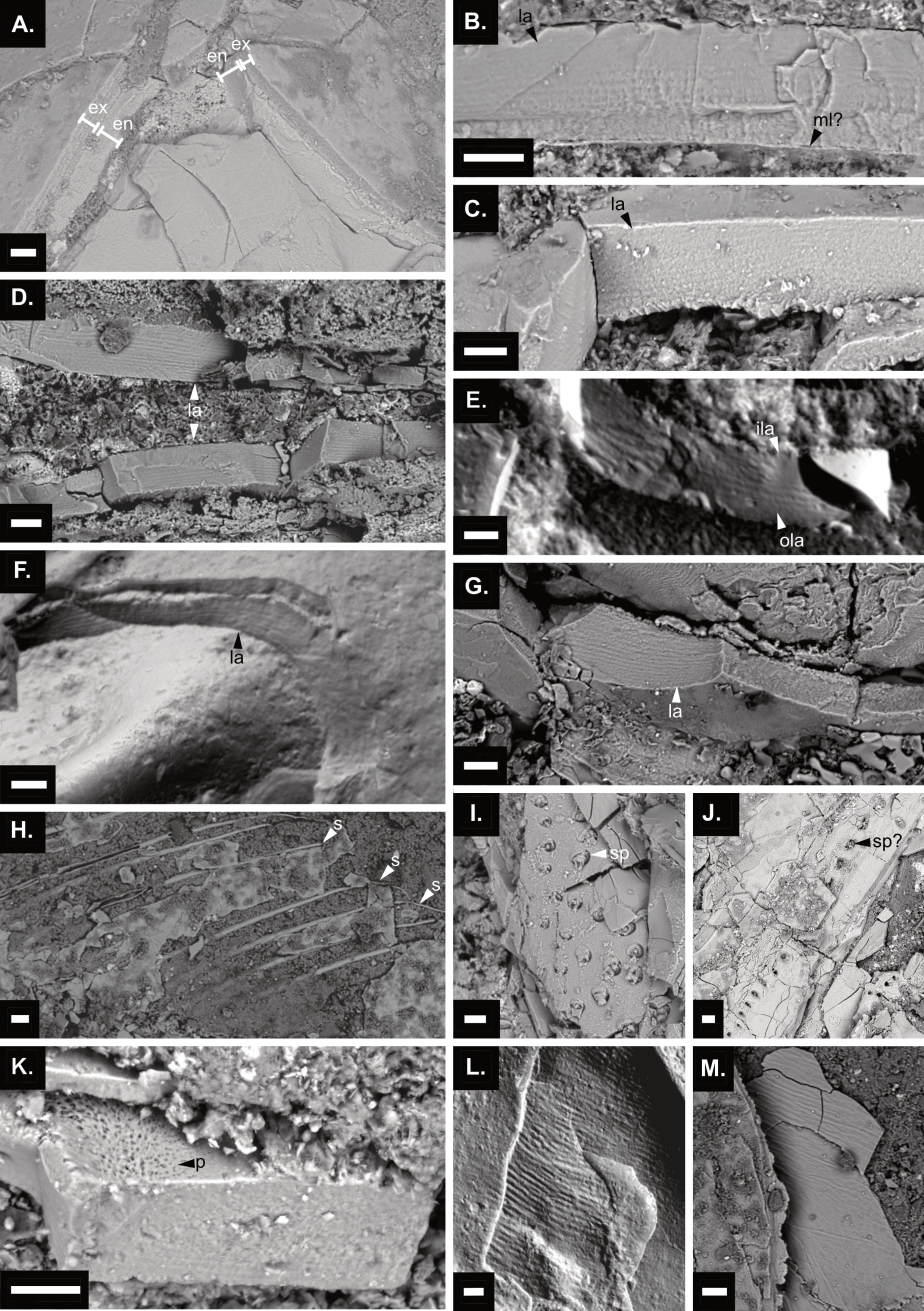
Backscattered electron micrographs of the cuticle microstructure of *Cretapenaeus berberus*. (A) Cuticle structure showing both the endocuticle and exocuticle layers on the region between pleon and telson (MHNM-KK-OT 83b). (B–D) Endocuticle lamination in carcasses: (B) Region between pleon and telson (MHNM-KK-OT 83b). (C) Pleon (MHNM-KK-OT 86b). (D) Cephalothorax (MHNM-KK-OT 87-1). (E–G) Endocuticle lamination in moults: (E) Pleon (MHNM-KK-OT 82 a). (F) Unidentified appendage (MHNM-KK-OT 56a). (G) Cephalothorax (MHNM-KK-OT 87-2). (H) Uropod setae (MHNM-KK-OT 87-1). (I) Setal pits on telson (MHNM-KK-OT 83b). (J) Probable setal pits on uropod or telson (MHNM-KK-OT 87-1). (K) Unidentified porous structures on pleon (MHNM-KK-OT 86b). (L–M) Wrinkled cuticle surfaces in moults. (L) Region between cephalothorax and pleon (MHNM-KK-OT 82 a). (M) Cephalothorax (MHNM-KK-OT 87-2). Scale bars equal 20 µm. en, endocuticle; ex, exocuticle; la, lamination; ila, inner lamination; ola, outer lamination; ml?, possible membranous layer; p, porous structure; s, seta; sp, setal pit; sp?, possible setal pit. Images: S. Lynch; C. Martin Olmos.

### Cuticle structure

A thin cuticle layer, likely the membranous layer, is visible on MHNM-KK-OT 83. However, determining with certainty the inner and outer side of the cuticle was challenging at that location, therefore this could alternatively be the epicuticle layer (Fig. 5B). In multiple specimens, both the endo- and exocuticle layers are visible, and are physically separated layers (Fig. 5A), which together with their fragmentary nature, makes their joint preservation uncommon. The endocuticle is thicker than the exocuticle; for example, on MHNM-KK-OT 83 the endocuticle is ∼30 µm and the exocuticle ∼20 µm (Fig. 5A). However, these measurements remain approximate due to the fragmentary nature of the cuticle and because the cuticle is not flat on these images. Additionally, cuticle thickness varies considerably depending on the body region, even in close proximity.

Lamination is present in both the endo- and exocuticle, with fewer lamination present in the exocuticle (Fig. 5A). The endocuticle laminae are spaced further apart in the outer layers than in the inner layers (Fig. 5E). However, this pattern is not always evident, with some specimens showing closely spaced laminae on both sides (Figs. 5D, 5G). Endocuticle lamination was observed in the three moults and three carcasses of adult *C. berberus* analysed (Fig. 5). Lamination was not observed in the two late postlarvae or juveniles (MHNM-KK-OT 80, 70-1), likely due to preservational or instrumental resolution.

Other structures were observed on the cuticle such as wrinkled surfaces on moults (Figs. 5L, 5M) and complete setae (Fig. 5H). Additionally, clusters of setal pits containing truncated setae are exposed from an inner cuticle layer (Fig. 5I), while possible setal pits appear aligned on the outermost cuticle surface (Fig. 5J). An unidentified porous structure was also observed, likely on the endocuticle surface (Fig. 5K).

### Development and developmental stage identification

Of the 65 specimens, two were identified as either late postlarvae or juveniles, six as definite juveniles, six as intermediates and 51 as adults (see dataset in Supplemental Information 1). Key specimens are described below, and the characteristics that allow attribution of specimens to these developmental stages are detailed in the discussion.

**Figure 6:**
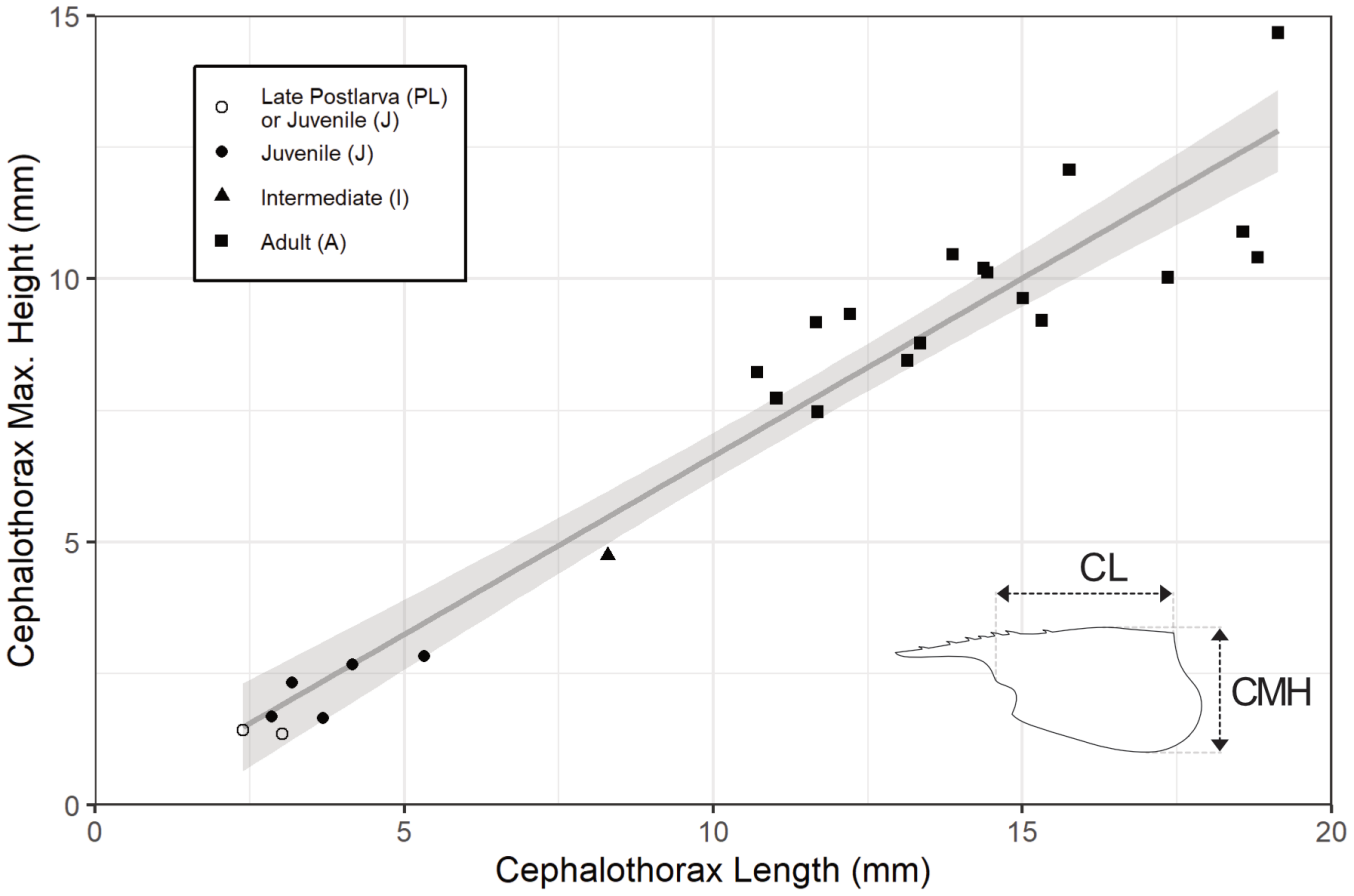
Carapace length (CL) and maximum height (CMH) of 25 *Cretapenaeus berberus* specimens. See upper left box for developmental stage identification. Linear regression line with 95% confidence interval (Adjusted *R*^2^: 0.93; Regression coefficient: 0.68; *p* < 0.001).

**Figure 7:**
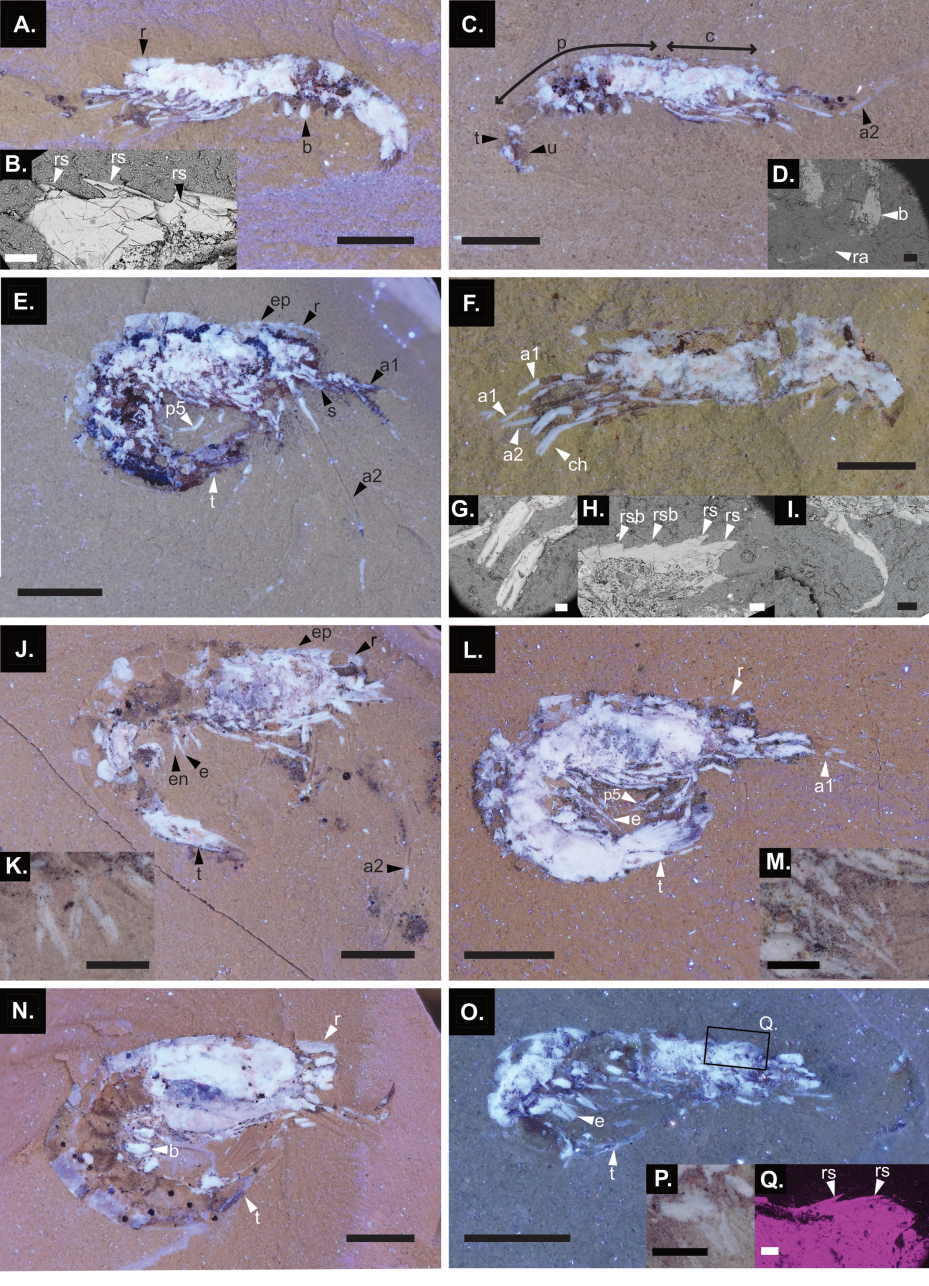
Juveniles (including two possible late postlarvae: MHNM-KK-OT 70-1, 80) of *Cretapenaeus berberus*. (A, C, E, F, J, L, N, O). UV photographs. (B, D, G, H, I). Backscattered electron micrographs. Q. BSE-EDS mapping of calcium. (K, M, P) Digital microscopy images. (A) MHNM-KK-OT 80b, with zoom on rostrum (B). (C) MHNM-KK-OT 80a, with zoom on pleopod (D). (E) MHNM-KK-OT 79. (F) MHNM-KK-OT 70a-1, with zoom on the chelae (G). (H–I) MHNM-KK-OT 70b-1, zoom on the pleopod ramus (H) and rostrum (I). (J) MHNM-KK -OT 92 , with zoom on pleopods (K). (L) MHNM-KK -OT 93 , with zoom on pleopods (M). (N) MHNM-KK -OT 91. (O) MHNM-KK-OT 61b, with zoom on pleopods (P), and black square indicating the zoomed-in area of the rostrum (Q). Scale bars in (A, C, E, F, J, L, N, O) equal two mm, 100 μ m in (B, D, G, H, I, Q), and 0.5 mm in (K, M, P). a1, antennule; a2, antenna; b, pleopod basis; c, cephalothorax; ch, pereiopod with chela; e, exopod; en, endopod; ep, epigastric tooth; r, rostrum; ra, pleopod ramus; rs, rostral spine; rsb, rostral spine base; p, pleon; p5, pereiopod 5; s, scaphocerite; t, telson; u, uropod. Photographs: S. Lynch; C. Martin Olmos.

**Figure 8:**
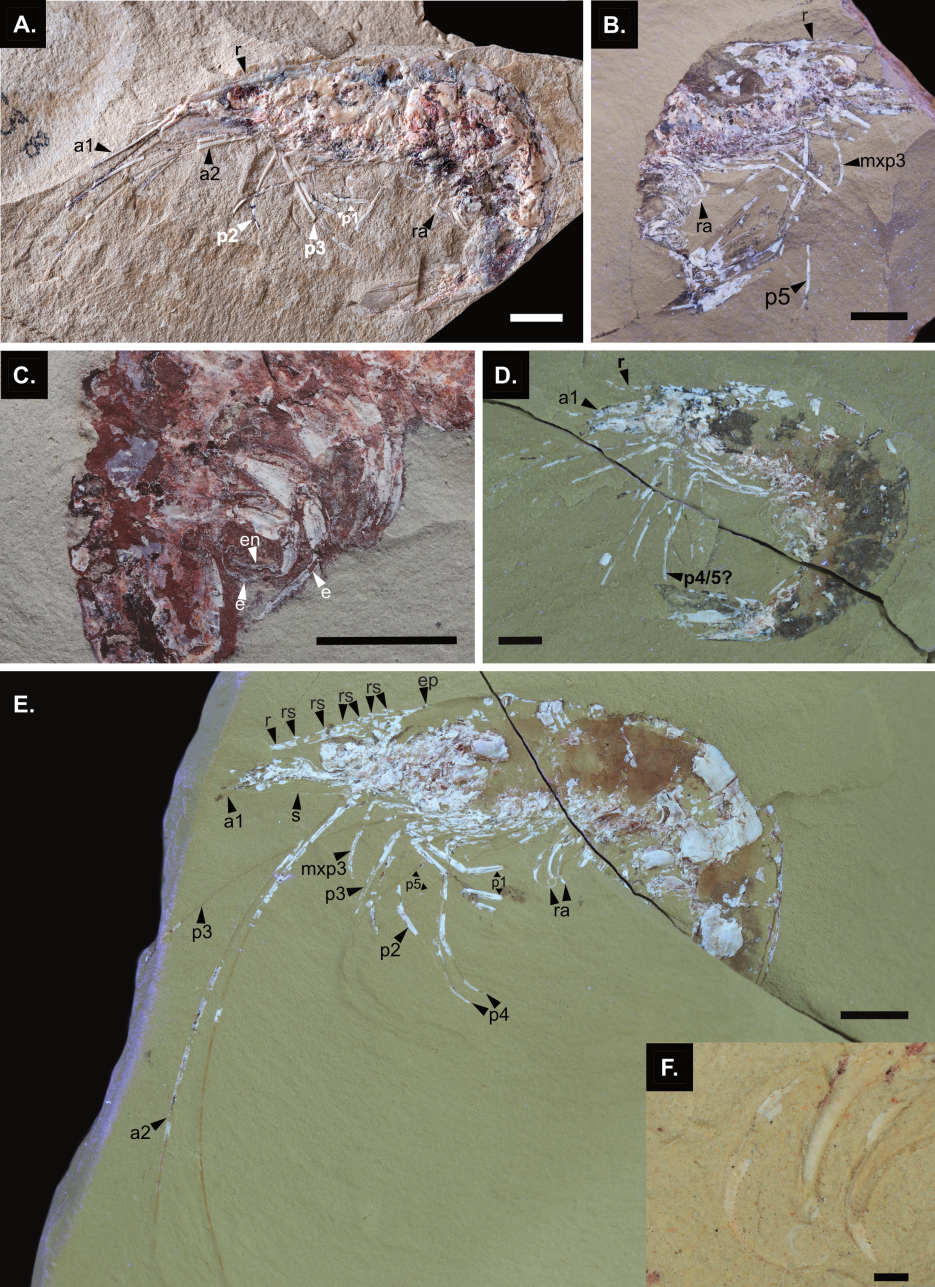
Adults of *Cretapenaeus berberus*. (A) Visible light photography of holotype MNHN.F. A24633b. (B–E) UV photography. (B) Paratype MNHN.F. A24634a. (C) Pleopods of MNHN.F. A88745b. (D) MHNM-KK-OT 7a. (E) MHNM-KK-OT 75a, with digital microscopy images of pleopods (F). Scale bar equal five mm in (A, B, C, D, E), and 0.5 mm in F. a1, antennule; a2, antenna; e, exopod; ep, epigastric tooth; mxp3, third maxilliped; p1–5, pereiopod 1–5; p4/5?, pereiopod 4 or 5; r, rostrum; ra, pleopod ramus (endopod or exopod); rs, rostral spine; s, scaphocerite. Photographs: S. Lynch, L. Cazes for holotype MNHN.F.A 24633b in A.

Modeling the maximum height of the carapace (CMH) as a function of its length (CL) with a regression model showed a significant linear relationship (Fig. 6; Adjusted R^2^: 0.93; unstandardised regression coefficient: 0.68; *p* = 1.07 × 10^−14^). Two size groups are apparent, with smaller specimens (7 specimens with measurable CL and CMH, with ≤5.32 mm CL) being less abundant than their larger counterparts (18 specimens with ≥10.71 mm CL, of which 17 have measurable CL and CMH, and one with only CL, thus not plotted on Fig. 6), and with only one specimen having a CL between these size groups (Fig. 6).

MHNM-KK-OT 80 and 70-1 were identified as either late postlarvae or juveniles. Of these, the smallest specimen based on carapace length (CL) (MHNM-KK-OT 80, 2.39 mm CL) already exhibits a cephalothorax, a pleon, a telson, uropods, pereiopods and pleopods (Figs. 7A–7D). Several pleopod bases are visible, with only one weakly preserved natatory ramus visible (Fig. 7D). While the pereiopods of MHNM-KK-OT 80 are present, their poor preservation prevents determination of whether they bear chelae. However, they are proportionally elongated and lack enlarged exopods. MHNM-KK-OT 80 has three spines on the rostrum, though the tip is concealed (Fig. 7B). The epigastric tooth is not visible, but may be broken. An elongated antenna is visible, along with another cephalic appendage that is probably a poorly preserved antennule (Fig. 7C). The uropods and telson are partly visible, though the tips are not exposed. The second possible late postlarva or juvenile specimen, MHNM-KK-OT 70-1 (Figs. 7F–7I), has a slightly longer CL (3.03 mm CL) than one specimen identified as a definitive juvenile (MHNM-KK-OT 79, 2.85 mm CL) but has a slender body similar to MHNM-KK-OT 80. Like MHNM-KK-OT 80, MHNM-KK-OT 70-1 retains the pleopod basis with one well-preserved ramus (Fig. 7I) and it has proportionally elongated pereiopods, lacking enlarged exopods. MHNM-KK-OT 70-1 also has an incomplete rostrum, though it displays two complete rostral spines, and the base of two additional rostral spines (Fig. 7H). The epigastric tooth is not visible. MHNM-KK-OT 70-1 also exhibits new characteristics compared to MHNM-KK-OT 80, specifically chelae on the pereiopods (Fig. 7G), and the antennules (Fig. 7F).

The sample includes six definite juveniles (MHNM-KK-OT 61, 79, 81, 91, 92, 93), five of which are figured (Figs. 7E–7J–7Q). Part of the rostrum is visible in some juveniles (MHNM-KK-OT 61, 79, 91, 92, 93). At least two rostral spines are visible on MHNM-KK-OT 61 (Fig. 7Q). Three rostral spines are visible on specimens MHNM-KK-OT 91, 92, and 93, while MHNM-KK-OT 79 displays four. The epigastric tooth is visible on specimens MHNM-KK-OT 79 and 92. However, none of these specimens clearly reveal the very tip of the rostrum, though its outline may be faintly perceptible in MHNM-KK-OT 93, and is almost complete on MHNM-KK-OT 92. An elongated antenna is visible on juveniles MHNM-KK-OT 79 and 92. The antennules are visible on juveniles MHNM-KK-OT 79 and MHNM-KK-OT 93. In MHNM-KK-OT 92, both the endopod and exopod are present on the pleopod ramus (Fig. 7J), with the endopod appearing smaller. In MHNM-KK-OT 61 and 93, the morphology of the pleopod ramus is clearly defined: it is long, leaf-like, tapered distally, and lacks strong annulation (Figs. 7M–7P), similar to the well-preserved ramus of the late postlarva or juvenile MHNM-KK-OT 70-1 (Fig. 7I). The pereiopods are well-preserved on juvenile specimens MHNM-KK-OT 79 and 93.

Key differences between adults and juveniles include the following characters (Fig. 1). Although no complete juvenile rostrum has been observed, some appear to be missing only the very tip, indicating that the adult rostrum is proportionally longer. Additionally, the adult rostrum likely bears more spines than the juveniles, with six dorsal spines and an epigastric tooth observed in adults (Fig. 8E), compared to a maximum of four rostral spines and an epigastric tooth in juvenile rostrums, though none were complete in the juveniles. The antenna, and the fourth and fifth pereiopods are also proportionally longer in adults than in juveniles (Figs. 8A, 8B, 8E). The adults have pleopods with two equally sized rami (Fig. 8C), whereas in juveniles the endopod appears smaller. The rami of the adult pleopods (Fig. 8F) are strongly annulated, thinner, and can exhibit strong curling compared to juveniles. While the adult carpus of the third pereiopod (based on the holotype MNHN.F.A24633, Fig. 8A) is only slightly longer than that of juveniles, the incomplete carpus on other specimens, such as MHNM-KK-OT 75 (Fig. 8E), suggests it may have reached even larger sizes. The third maxilliped, previously undescribed in Garassino, Pasini & Dutheil (2006) is observable in the new adult specimens of *C. berberus* examined here. It is pediform, as it is morphologically similar to the pereiopods; however, its segmentation could not be observed.

## Discussion

### Moulting

Moults are rare (8) compared to carcasses (45). Collection bias favouring carcasses is unlikely as an explanation for this, as careful attention was made on-site to collect all specimens observed. However, this cannot be fully excluded as moults are harder to detect than carcasses because they lack the more visible muscles shown by carcasses. Another possible explanation for the scarcity of moults is their greater fragility, which may have led to poorer preservation than for carcasses. Alternatively, *C. berberus* may have consumed its own moults or parts of them. Such behaviour has been documented in extant penaeids like *Penaeus monodon* and *Penaeus* (*Litopenaeus*) *vannamei* (Boone, 1931), which consume the softer parts of their moults (pers. obs. by H. Truong, CSIRO and H. Duong, Viet-Uc reported in Truong et al. (2022). Another study also reported *Penaeus vannamei* consuming its own moults (Chan, Rankin & Keeley, 1988). Similarly, *Farfantepenaeus duorarum* (Burkenroad, 1939) has been reported to eat its own moults, excluding the harder carapace and non-moulting individuals may also eat the moults of other individuals (Bursey & Lane, 1971). In contrast, *Penaeus* (*Fenneropenaeus*) *merguiensis* De Man, 1888 was reported not to consume its moults (Dall et al., 1990a). Our small sample of *C. berberus* moults does not allow us to determine whether specific parts are systematically missing, which could indicate their partial consumption.

Extant penaeids have been reported to exuviate through an initial opening between the carapace and the pleon (Bursey & Lane, 1971; Dall et al., 1990a). In *Farfantepenaeus duorarum*, the exoskeleton was observed shed in a single piece, without detachment of the carapace from the rest of the cephalothorax (Bursey & Lane, 1971). Two probable penaeid fossil moults from the Late Jurassic of Solnhofen (Germany) also show a displaced but unfragmented carapace attached to the sternum (Charbonnier & Garassino, 2012). *C. berberus* appears to have moulted through an opening in the same general area as extant and extinct penaeids (Fig. 2B), but moulting in *C. berberus* may have caused the full detachment of the carapace, or they may have ingested part of it (MHNM-KK-OT 82, 64).

The absence of the carapace in *C. berberus* moults (MHNM-KK-OT 82, 64) is unlikely to be due to taphonomic processes, as decay experiments on modern shrimps indicate that the early stage of decomposition results in a slight separation of the carapace followed by the complete separation of the cephalothorax, including the sternum, from the pleon (Plotnick, 1986; Klompmaker, Portell & Frick, 2017; Corthésy et al., 2025), which is different from the disarticulation pattern observed in *C. berberus* fossils. The detachment of the carapace from the cephalothorax was observed in shrimps decaying on the surface of sediments in the absence of burial and without a thick microbial mat on the substrate (Corthésy et al., 2025). The *C. berberus* specimens studied here were not rapidly buried by sediment immediately after death, but were likely covered rapidly by the microbial mat that covered the sedimentary surfaces, which increased their preservation potential and enabled their soft tissues to be preserved (Iniesto et al., 2013; Iniesto et al., 2015; Iniesto et al., 2016; Gueriau et al., 2020). The absence of visible exoskeleton disarticulation in the moult specimens (Figs. 3A–3I, 3M–3O), other than the characteristic and repeated loss of part of the carapace (Figs. 3A–3I), indicates that the specimens were not displaced or transported. This in turn, suggests that the moults were not exposed for a long period of time to the water-sediment interface (Allison, 1986), and that entombment by microbial mat occurred relatively quickly. The loss of the carapace therefore most likely results from the moulting process (Fig. 2B). The moults studied cannot be said to be decomposed carcasses, as the detachment of carapace does not correspond to a decay pattern observed in extant shrimps in taphonomic experiments (Plotnick, 1986; Klompmaker, Portell & Frick, 2017; Corthésy et al., 2025).

The presence of visible endophragmal elements does not conclusively identify specimens as moults (Feldmann & Tshudy, 1987). However, moults in Salter’s position, with a displaced carapace (Feldmann & Tshudy, 1987), are likely to expose these elements (Jones et al., 2016). Endophragmal elements were observed in all *C. berberus* moults with a laterally preserved cephalothorax (Figs. 3B, 3E, 3H). Additionally, moults contain no soft tissues, which can obscure the visibility of these structures in carcasses.

Carcasses are generally more colourful than moults because they contain opaque pink muscles in addition to the almost transparent cuticle, whereas moults are primarily composed of almost transparent cuticle in visible light (Figs. 3A, 3D, 3G, 3M). Additionally, the carcasses selected for multispectral imaging have more surface area coverage of microbial mats than the moults, as highlighted on the false-colour RGB multispectral images where microbial mats appear green (Figs. 3I, 3L). It is possible that carcasses generally have more coverage of microbial mat owing to the presence of soft tissues, and therefore, more decaying organic matter that facilitated the growth of microbial mat. Although in this case multispectral imaging and spectroscopy highlight contrasts in the fossil compositions (specifically distinguishing between muscle and cuticle), neither of these approaches provide herein a new tool for discriminating carcasses from moults based on cuticle alone.

### Cuticle microstructure

The exocuticle and endocuticle of *C. berberus* form distinct layers and tend to physically separate, a trait also reported in the extant penaeid *Metapenaeus* sp. (Dall, 1965a). This detachment, combined with the fragmentary nature of the cuticle, means that the exocuticle and endocuticle are rarely preserved intact and in direct contact within *C. berberus* specimens. This makes distinguishing between these layers and identifying outer surfaces from inner ones challenging. Lamination occurs in both the endo- and exocuticle of *C. berberus* and extant penaeids (Dall, 1965a; Dall et al., 1990d; Promwikorn, Boonyoung & Kirirat, 2005). In both, lamination is finer in the endocuticle compared to the exocuticle (Dall, 1965a; Corteel & Nauwynck, 2010). In *C. berberus*, generally the endocuticle laminae are spaced further apart in the outer layers compared to the inner layers (Fig. 5E). However, this pattern is not always consistent (*e.g.,* Figs. 5D, 5G), possibly due to the uneven nature of the broken surfaces. A similar pattern, with more widely spaced laminae in the outer endocuticle, also occurs in extant *Penaeus monodon* (Promwikorn, Boonyoung & Kirirat, 2005), and a histological figure illustrates the same condition in *P. duorarum* (Schafer, 1967: fig. 5).

Based on the observation in recent lobsters that exocuticle resorption occurs only along specific breakage lines, the loss of exocuticle lamination in Cretaceous nephropid lobsters was attributed to taphonomic processes (Feldmann & Tshudy, 1987). In contrast, the loss of endocuticle lamination was attributed to resorption prior to moulting (Feldmann & Tshudy, 1987). Although evidence remains limited, with only 10 specimens in Feldmann & Tshudy (1987) study showing cuticle lamination, the lack of lamination in the inner endocuticle or exclusive exocuticle lamination was observed only in disarticulated Cretaceous nephropid lobsters or those in Salter’s position. In contrast, no differences in endocuticle lamination were found between moults and carcasses of *C. berberus* (Figs. 5A–5G). Additionally, in the fossil nephropid lobsters, endocuticle lamination was more often seen in the pereiopods, whereas *C. berberus* showed no clear pattern of localised lamination. The higher prevalence of lamination in the nephropid pereiopods was attributed to greater calcification and limited resorption during moulting (Feldmann & Tshudy, 1987).

The extent of cuticular resorption prior to moulting varies among decapod groups and even within a species (Passano, 1960). If mineral and organic material resorption is confirmed to cause reduced lamination, as Feldmann & Tshudy (1987) suggested, differences in lamination patterns between moults and carcasses of *C. berberus* and the Cretaceous nephropid lobsters may reflect varying resorption levels. These nephropid lobsters had a thicker, likely more calcified cuticle than *C. berberus,* and extant penaeids are believed to have lower calcium demands than decapods with more robust exoskeletons (Dall et al., 1990a). However, unlike extant penaeids, which likely obtain sufficient calcium from their marine environment (Dall, 1965b; Dall & Smith, 1981; Dall et al., 1990a), *C. berberus* lived in a freshwater environment where calcium may have been scarcer. If so, then *C. berberus* may have required increased calcium resorption and storage before moulting compared to its marine counterparts, as the freshwater crayfish (Dall et al., 1990a; Luquet & Marin, 2004). While they focused on mineral resorption, Feldmann & Tshudy (1987) also associated organic material resorption with the corroded appearance of the endocuticle in the nephropid lobsters. This could be particularly relevant in penaeids, as a study showed that in modern *Metapenaeus* sp. negligible amounts of inorganic material were resorbed before moulting, with chitin and protein content decreasing by 39%, making moulted carapaces only 77% of the weight of intermoult carapaces (Dall, 1965a). However, that study did not address lamination differences between moults and carcasses. Considering these aspects, further investigation into the impact of resorption on cuticle lamination, particularly in extant penaeids, is essential before drawing conclusions about *C. berberus.*

A thinner cuticle and an abraded inner cuticle surface have also been proposed as indicators of resorption in decapod moults (Feldmann & Tshudy, 1987). However, these features were inconclusive in distinguishing moults from carcasses in fossil nephropid lobsters (Feldmann & Tshudy, 1987) and were not tested in *C. berberus.* Identifying cuticle surfaces in *C. berberus* is challenging due to its fragmentary nature, especially the inner surface, which is often in contact with other tissues or sediment. Confirming thickness differences would require destructive cross-sections to be made to ensure the cuticle is entirely preserved and to obtain flat surfaces suitable for accurate measurement. However, considering that meaningful thickness comparisons require a large sample of specimens with comparable dimensions, sectioned at the same body location—as decapod cuticle thickness can vary depending on specimen size (Feldmann & Tshudy, 1987), body region (Dall, 1965a), and moult cycle stage (Feldmann & Tshudy, 1987; Promwikorn, Boonyoung & Kirirat, 2005)—and that the limited *C. berberus* material does not allow for such an approach, this method could not be applied in this study.

Dall et al. (1990d) reported that in penaeids the endocuticle and exocuticle are of comparable thickness, though the endocuticle can be slightly thicker depending on the part of the body. In the intermoult stage of *F. duorarum*, the endocuticle was reported to constitute 80% of the total thickness of the integument, though the accompanying images show a thicker exocuticle (Schafer, 1967). In *Metapenaeus* sp., however, the exocuticle generally makes up 52–55% of total cuticle thickness, with the intermoult stage clearly showing a thicker exocuticle than endocuticle (Dall, 1965a: fig. 1). In *C. berberus*, the endocuticle and exocuticle were rarely preserved intact and in association, but carcass MHNM-KK-OT 83 exhibits a thicker endocuticle than the exocuticle (Fig. 5A), resembling the intermoult stage of *Penaeus monodon*, as illustrated by Promwikorn, Boonyoung & Kirirat (2005: fig. 1C) (though in their fig. 1 in general, depending on staining, the membranous layer can appear almost as thick as the endocuticle). A specimen of *Penaeus* sp. was found to have a cuticle with a thickness of ∼126 µm in the branchial region, with the endocuticle accounting for ∼56 µm (Amato et al., 2008), although the moult stage of the examined specimens was not specified. By contrast, in *C. berberus* specimen MHNM-KK-OT 83, the cuticle measures ∼50 µm in total, comprising an endocuticle of ∼30 µm and an exocuticle of ∼20 µm at the region between the pleon and telson (Fig. 5A). In this case, the endocuticle represents ∼60% of the cuticle in *Cretapenaeus*, compared to ∼44% in *Penaeus* sp. However, these measurements come from a different region of the body. Additionally, although we could not directly measure the cuticle thickness in MHNM-KK-OT 83, the *Penaeus* sp. individual had a cephalothorax length of 29.8 mm, which is longer than the largest *C. berberus* specimen measured here (∼20 mm CL). A wrinkled surface was observed on the cuticle of some *C. berberus* moults (Figs. 5L, 5M). Wrinkles also occur on the postmoult cuticle of extant decapods (O’Halloran & O’Dor, 1988; Hunter & Uglow, 1998; Amer et al., 2016), before water is reabsorbed and the cuticle expands (O’Halloran & O’Dor, 1988). Since the wrinkled surface of *C. berberus* appeared within inner cuticle layers rather than on the outermost surface, it likely resulted from taphonomic processes. While observed only in moults, cuticle wrinkles may be less visible in carcasses due to the additional tissues concealing it. The possible aligned setal pits on the outermost cuticle surface (Fig. 5J) resemble those of *Penaeus* (*Eopenaeus*) *semisulcatus* De Haan, 1844 (Rashwan et al., 2024: fig. 8F).

### Developmental stage identification

Several morphological characteristics indicate that the smallest specimen in the sample (MHNM-KK-OT 80) has progressed beyond the mysis stage (Figs. 7A–7D). Firstly, although MHNM-KK-OT 80 may initially appear to have large pleopod buds, characteristic of the second mysis stage (Dall et al., 1990d), these structures represent the enlarged proximal pleopod segment (basis) of a later developmental stage with the fragile distal ramus only preserved on one pleopod (Fig. 7D). Additionally, the pereiopods of MHNM-KK-OT 80 are longer than usually figured for the third mysis stage (*e.g.,* Choi & Hong, 2001: fig. 6F; Dobkin, 1961: fig. 17A; Jackson et al., 1989: fig. 6; Ronquillo, Saisho & McKinley, 2006: fig. 10) and appear to be at least as long as the ones of postlarvae (*e.g.,* Choi & Hong, 2001: fig. 6H; Dobkin, 1961: fig. 18A; Jackson et al., 1989: fig. 7; Ronquillo, Saisho & McKinley, 2006: fig. 11). However, their exact length cannot be established due to their fragmentary condition. Exopods are present in both the mysis and postlarval stages of extant penaeids, but they are proportionally larger in the mysis than in the postlarval stage (Dall et al., 1990d). Although the presence of smaller exopods cannot be excluded, enlarged exopods are definitively absent from the pereiopods MHNM-KK-OT 80 (Figs. 7A, 7C). Finally, MHNM-KK-OT 80 displays part of a developed rostrum with three visible spines (Fig. 7B), although the tip remains concealed by sediment. Dall et al. (1990b), Dall et al. (1990d) noted that rostral spines first emerge during the mysis stage, and that adult rostral features begin to develop in juveniles. However, Motoh & Buri (1980b) showed that, even among *Penaeus* (Fabricius, 1798) species, rostral spines can appear at different developmental stages. For example, they found that in *Penaeus* (*Marsupenaeus*) *japonicus* (Bate, 1888), rostral spines appear as early as the first mysis, whereas they are still absent in the first postlarva of *Penaeus (F.) merguiensis.* Although typically it seems that three rostral spines are not found before the third mysis (Ronquillo & Saisho, 1997), or the first postlarva (Motoh & Buri, 1980b; Paulinose, 1986).

The distinction between the postlarval and juvenile stage is more challenging to assess, as the morphologies of earlier larval stages in penaeids are better documented. Motoh & Buri (1980a) provided further insights into the morphological development of the postlarval stages and juveniles of the penaeid *P. monodon*, referring to the entire sequence as “postmysis” and describing 12 postmysis stages. Dall et al. (1990b: table 3.2) instead divided this postmysis sequence into six postlarval and four juvenile stages that were considered to be an incomplete sequence. Following Dall et al. (1990b), the first six postmysis stages of Motoh & Buri (1980a) are termed postlarvae in this study. Since Dall et al. (1990b) did not justify selecting only four juvenile stages from Motoh & Buri (1980a), the last six postmysis stages are considered as a continuous juvenile sequence herein.

According to Dall et al. (1990b), the transition from the postlarval to the juvenile stage is marked by several changes. Those observable in fossils include a bulkier body appearance, the emergence of adult rostral and branchial characteristics, and a pointed telson. Motoh & Buri (1980a) examined some of these traits in more detail in *P. monodon*. They noted, for example, that the telson becomes more slender from the third postlarva (third postmysis) and sharply pointed by the third juvenile (ninth postmysis), a more gradual change than described by Dall et al. (1990b). Additionally, the number of telson spines begins decreasing at the second juvenile stage (eight postmysis). They also found that the rostral spine formula is completed by the first or second juvenile stage (seventh or eighth postmysis), providing a detailed example of juvenile rostral development mentioned by Dall et al. (1990b). However, the rostral spine formula is highly variable between taxa (Motoh & Buri, 1980b) and is therefore unsuitable for directly comparing *C. berberus* with *P. monodon*. Additionally, the telson tip cannot be observed and assessed in MHNM-KK-OT 80.

Motoh & Buri (1980a) documented additional morphological changes throughout the developmental sequence of *P. monodon*. Of these, the antenna (endopod of the second antenna in Motoh & Buri, 1980a) of MHNM-KK-OT 80 (Fig. 7C) is clearly at least at the sixth postlarval or first juvenile stage (sixth or seventh postmysis) of Motoh & Buri (1980a), where the antenna becomes longer than the scaphocerite (exopod of the second antenna in Motoh & Buri, 1980a). Motoh (1985) also introduced the shortening of the sixth pleonal segment relative to the carapace length in *P. monodon* as marker of the “juvenile” stage. Although the juvenile onset was set at the fourth “postmysis” in Motoh (1985) (fourth postlarva herein), rather than following the sixth postlarval stage as in Dall et al. (1990b), it still represents a key developmental change (Motoh, 1985: fig. 6; Motoh & Buri, 1980a: fig. 14). In MHNM-KK-OT 80, these proportions more closely resemble illustrated juveniles of *P. monodon* than postlarval ones, though the sixth postlarva is not depicted in Motoh (1985: fig. 6) and Motoh & Buri (1980a: fig. 14). In conclusion, certain morphological features of MHNM-KK-OT 80, such as the proportionally elongated antennae and proportionally reduced sixth pleonal segment, suggest that it is at least a late postlarva. However, its slender appearance compared to juveniles does not support a juvenile classification. Therefore, MHNM-KK-OT 80 is conservatively interpreted as corresponding to a late postlarval to juvenile stage.

Although the carapace length (CL) of MHNM-KK-OT 70-1 (Figs. 7F–7I) is slightly longer than in the juvenile MHNM-KK-OT 79, its slender body more closely resembles MHNM-KK-OT 80. Similar conditions of the pleopods (Fig. 7I), pereiopods and rostrum (Fig. 7H) to those of MHNM-KK-OT 80 support that MHNM-KK-OT 70-1 has passed the mysis stage. Specimen MHNM-KK-OT 70-1 also exhibits new characteristics compared to MHNM-KK-OT 80. Firstly, its enlarged chelae (Fig. 7G) also support an identification as a later developmental stage than mysis, as they align with Dall et al. (1990d)’s description of postlarval chelae, which become functional only after the mysis stage when they are rudimentary. Additionally, MHNM-KK-OT 70-1 displays proportionally an elongated flagellum on the antennule (second antenna of Motoh & Buri, 1980a), resembling the sixth juveniles (twelfth postmysis) illustrated by Motoh & Buri, 1980a: fig. 3), although the fifth juvenile (eleventh postmysis) is not illustrated. Given this characteristic, but considering the less bulky body appearance than juvenile MHNM-KK-OT 79 and overall resemblance to MHNM-KK-OT 80, MHNM-KK-OT 70-1 is also conservatively interpreted as corresponding to a late postlarval to juvenile stage.

The proportionally elongated flagella on the antennules of MHNM-KK-OT 79 (Fig. 7E) resemble that of the sixth juvenile (twelfth postmysis), and the elongated antennae are similar to those of the second and sixth juvenile stages (eight and twelfth postmysis) (Motoh & Buri, 1980a: figs. 3 and 14). However, since these features are not illustrated across all postlarval and juvenile stages, a more precise assignment is not possible. Based on its bulkier body than MHNM-KK-OT 80 and MHNM-KK-OT 70-1, and its pointed telson, MHNM-KK-OT 79 is classified as a definite juvenile. MHNM-KK-OT 79 displays the first definitive epigastric tooth along with four rostral spines, though the rostrum tip is absent. However, the epigastric tooth typically appears in earlier developmental stages in extant penaeids, for example at the second mysis (Ronquillo & Saisho, 1997), the third mysis (Rao, 1973; Hassan, 1982; Türkmen, 2005), or the first postlarval stage (Choi & Hong, 2001). Other specimens (see full list in Supplemental Information 1) were identified as juveniles (Figs. 7J–7Q), based on their resemblance to MHNM-KK-OT 79 and their similar or larger sizes.

Although Dall et al. (1990d: table 2.2) noted that postlarvae have uniramous pleopods, Motoh & Buri (1980a) observed that the endopods of the pleopods appear as small structures at the second juvenile stage (eighth postmysis) and gradually elongate subsequently. Biramous pleopods were observed in only one juvenile *C. berberus* (MHNM-KK-OT 92, Figs. 7J, 7K). As described by Garassino, Pasini & Dutheil (2006), the pleopods of *C. berberus* adults are biramous with two equal-sized rami, similar to extant penaeids which have both rami but with a larger exopod (Dall et al., 1990d) (Fig. 8C).

In this study, juveniles are not distinguished from the adolescent or subadult stages described by Motoh (1985), as the criteria are too challenging to apply to fossils. Since the morphological transition from juveniles to adults is not yet well-defined and likely gradual in extant penaeids, some specimens were classified as intermediates based on their sizes between those of juveniles and adults, though some may exhibit adult traits, such as strongly annulated and curled pleopods. *C. berberus* adults are distinguished from juveniles by their larger sizes, and distinctive morphological features (complete rostrum with a full formula, proportionally elongated antennae, proportionally elongated fourth and fifth pereiopods, and pleopods that are strongly annulated, tapered, and curled). While the morphology of juveniles and adults is very similar, morphological changes continued throughout this transition (Fig. 1). Currently, there is little information about this transition in extant penaeids, although Motoh (1985) and Motoh & Buri (1980a) illustrated two juvenile stages in *P. monodon* (eighth and twelfth postmysis in Motoh, 1985: fig. 6, and Motoh & Buri, 1980a: fig. 14), which exhibit a proportionally shorter antenna than the adults (Motoh, 1985: fig. 1).

The adult morphology of *C. berberus* was first described and reconstructed by Garassino, Pasini & Dutheil (2006). The new material presented herein (Fig. 1) provides only minor updates to the description of the adult morphology compared to Garassino, Pasini & Dutheil (2006). In our reconstruction (Fig. 1), the pereiopods are proportionally shorter than described in Garassino, Pasini & Dutheil (2006: fig. 7). This is partly due to some proximal pereiopod segments (as the ischium and part of the merus) being hidden by the carapace herein, whereas some of these segments were exposed in Garassino, Pasini & Dutheil (2006)’s reconstruction. Excluding the ischium, the fourth pereiopod is proportionally comparable in length to that in Garassino, Pasini & Dutheil (2006). There is a smaller length difference between the fourth and fifth pereiopods herein, although the fifth remains longer than the fourth. The carpus is notably proportionally longer in both the fourth and fifth pereiopods compared to Garassino, Pasini & Dutheil (2006). As a result, the carpus of the fifth pereiopod is proportionally longer than the propodus, whereas the reverse is true in Garassino, Pasini & Dutheil (2006). Although segmentation is not visible, the previously undescribed third maxilliped is figured herein, as it was preserved on some new adult specimens. However, it remains undescribed for earlier developmental stages. The adult antenna is also proportionally longer herein. Although some specimens were also observed with only curved pleopods, as figured in Garassino, Pasini & Dutheil (2006), many have strongly curled pleopods as depicted here.

### Developmental pattern

The lower abundance of smaller specimens of *C. berberus* (<10 mm CL, Fig. 6) is partly due to some small specimens lacking definitive morphological characteristics necessary for reliable species identification. The absence of distinct size-grouping among larger specimens (>10 mm CL, Fig. 6) may indicate frequent moulting, a behaviour known in extant penaeids (Dall et al., 1990a; Corteel et al., 2012).

Nauplius and protozoea were not observed for this species or among the other shrimp-like decapods from the Jbel Oum Tkout locality that could not be confidently assigned to *C. berberus*. Of the specimens confidently identified as *C. berberus* and therefore included in this study, there are no nauplius, protozoea, or mysis stages. The nauplius and protozoea are pelagic stages in extant penaeids (Dall et al., 1990c), with a transition from pelagic to benthic habitats during postlarval stages (as defined by Dall et al., 1990b, p. 114). The sample of *C. berberus* consists mainly of juveniles and adults, which are benthic in extant penaeids (Dall et al., 1990c). The smallest specimens may be late postlarvae, but these may also be benthic as the transition occurs in this stage (Dall et al., 1990b). The absence of early larval stages, which were likely pelagic (Dall et al., 1990c; Dall et al., 1990b), may be due to taphonomic and/or collection bias. The pelagic early larval stages might have lived in a different environment tothat preserved at the Jbel Oum Tkout locality, as described below, and/or the preservational processes at this site may have favoured the preservation of benthic over pelagic organisms, as mode of life is known to influence preservation potential. For example, in the Cambrian Walcott quarry in Canada, endobenthic animals are preferentially preserved over nektobenthic/epibenthic and nektonic/planktonic animals (Saleh et al., 2021). Another possibility is that their small size made them challenging to detect and collect during excavation, particularly since the highly fossiliferous OT1 mudstone preferentially splits along the largest remains.

The absence of early larvae could also reflect developmental processes, as in freshwater-dwelling pleocyematans the planktonic larval stages generally do not inhabit freshwater. Typically, their development is either abbreviated to direct, or they migrate between marine and freshwater settings at different life stages (Jalihal, Sankolli & Shenoy, 1993; Bauer, 2011a; Bauer, 2011b, Bauer, 2013; Anger, 2016). Alternatively, a few extant species of Pleocyemata have the necessary osmoregulatory abilities to inhabit a freshwater setting during their early larval stages (Anger, 2016), in which case the absence of early larvae could be due to taphonomic or collection bias as described above. It is not possible to determine which of these scenarios is more likely, given the available evidence. An argument against migration between marine and freshwater settings is that *C. berberus* inhabited the headland of a large river system, ∼250 km from the Tethys Ocean (Ibrahim et al., 2020: fig. 33), although some decapods, such as some species of the caridean *Macrobrachium*, are capable of migrating for hundreds of kilometres or more as part of their life cycle (Bauer, 2011a; Bauer, 2013; Anger, 2016). Conversely, an argument against abbreviated to direct development arises from the species’ classification within Dendrobranchiata, a group known for hatching at an early developmental stage (Anger, 2001; Tavares & Martin, 2010; Martin, Criales & Santos, 2014; Olesen, 2018).

## Conclusions

This study provides a detailed view of the growth of one of a rare freshwater Penaeidae *C. berberus*. Key contributions include:

• Insight into moulting patterns and cuticle microstructure compared to extant penaeids.
• Morphological changes during growth, particularly in the appendages.
• Evaluation of developmental strategies and possible freshwater life histories.
• Interesting aspects of the ontogeny and ecology of fossil freshwater decapods, rare among Dendrobranchiata.

The exceptional preservation of *C. berberus* specimens allowed for the identification of fossil moults and carcasses, and for an investigation of cuticle microstructure in both. Only eight moults were present among the 65 studied specimens of *C. berberus*, and their disarticulation pattern revealed they moulted similarly to extant penaeids. The cuticle microstructure of *C. berberus* is similar to extant penaeids, with lamination present both in the exo- and the thicker endocuticle. No differences were observed between carcasses and moults in either cuticle lamination or luminescence.

Specimens of *C. berberus* ranging from ∼2 to 20 mm in carapace length provided insight into its development. The smaller specimens preserved with high morphological details enabled a precise comparison with the adults, revealing continued morphological changes throughout their development. Specimens ranged from possible late postlarvae through juvenile stages and into adulthood. Early larval stages are absent from the collection. Compared to juveniles, some morphological structures, such as the antennae and some pereiopods, became proportionally longer in the adults, while the pleopods changed more drastically during development, becoming more curled and annulated and with equally sized rami in adults. The various possible developmental scenarios of *C. berberus* were evaluated in comparison with other freshwater decapod taxa in Pleocyemata. These include migration between freshwater and marine water, abbreviated to direct development, or larvae with the necessary osmoregulatory abilities for living in freshwater.

### Abbreviations

MSNM = Museo Civico di Storia Naturale, Milano (Italy).

MNHN.F. = Muséum national d’Histoire naturelle, Paris (France), palaeontological collections.

MHNM-KK-OT = Musée d’Histoire naturelle de Marrakech (Museum of Natural History of Marrakesh) (Morocco).

If these collection numbers are followed by a hyphen and a numeral (*e.g.,* MHNM-KK-OT 70ab-1), this indicates that multiple specimens are present on a single slab.

## Supplemental Information

10.7717/peerj.20463/supp-1 Supplemental Information 1 Export attestion UC specimens

10.7717/peerj.20463/supp-2 Supplemental Information 2 Kemkem fieldwork history

## Additional Information and Declarations

### Field Study Permissions

The following information was supplied relating to field study approvals (i.e., approving body and any reference numbers): For the newly collected 2012 material, all required permits were obtained, ensuring full adherance with applicable regulations, by the Moroccan Ministère de l’Énergie, des Mines, de l’Eau et de l’Environnement.

### Data Availability

The following information was supplied regarding data availability: The data is available at OSF: Lynch, Sinéad, Harriet B. Drage, Allison C. Daley, Pierre Gueriau, and Nora Corthésy. 2025. “Cretapenaeus Berberus.” OSF. November 7. osf.io/zdhuv.
