# Supplementary material for: Moulting and development in a freshwater prawn from the Cretaceous of Morocco
Source: PeerJ. 2026 Apr 9;14:e20463. doi: 10.7717/peerj.20463 (PMC13070322; doi:10.7717/peerj.20463)
Supplement: Supplemental Information 1 [file peerj-14-20463-s001.pdf]

ROYAUME DU MAROC

MINISTERE DE L'ENERGIE ET DES MINES

DIRECTION DE LA GEOLOGIE

DIVISION DE LA GEOLOGIE GENERALE

N° 327 ; [illegible] /DG/1

ATTESTATION

Je soussigné Monsieur Mohammed DAHMANI, Chef de la Division de la Géologie Générale, certifie que les échantillons fossilifères qui consistent en des restes et des ossements de:

Poissons (Coelacantes, Elanobranches, Teleosteens);
Lepidosauriens (serpents, lézards, varans);
Tortues (plusieurs espèces);
Dinosaures (Théropodes, Sauropodes)

sont emballés comme suit:

01	Boite de carton	15 kg
42	Boites en plastique "Curver"	1400 kg
05	Plâtres	175 kg
03	Boites en plastique	10 kg

dont le poids est d'environ 1600 kg (mille six cents kilogrammes), représentés par Monsieur le Professeur Paul SERENO de nationalité américaine sont prélevés au Maroc, en accord avec la Direction de la Géologie.

Ces échantillons sont destinés à des études scientifiques et n'ont aucune valeur commerciale.

Fait à Rabat, le 04 Juillet 1995

[illegible] Service de [illegible] Fondamentales

[illegible] Moh[illegible]
