# Supplementary material for: Moulting and development in a freshwater prawn from the Cretaceous of Morocco
Source: PeerJ. 2026 Apr 9;14:e20463. doi: 10.7717/peerj.20463 (PMC13070322; doi:10.7717/peerj.20463)
Supplement: Supplemental Information 2 [file peerj-14-20463-s002.pdf]

ZooKeys 928: 1–216 (2020)
doi: 10.3897/zookeys.928.47517
http://zookeys.pensoft.net

MONOGRAPH

A peer-reviewed open-access journal
ZooKeys
Launched to accelerate biodiversity research

Geology and paleontology of the Upper Cretaceous Kem Kem Group of eastern Morocco

Nizar Ibrahim[1], Paul C. Sereno[2], David J. Varricchio[3], David M. Martill[4], Didier B. Dutheil[5], David M. Unwin[6], Lahssen Baidder[7], Hans C.E. Larsson[8], Samir Zouhri[9], Abdelhadi Kaoukaya[7]

1 *Department of Biology, University of Detroit Mercy, Detroit, Michigan 48221, USA* **2** *Department of Organismal Biology and Anatomy and Committee on Evolutionary Biology, University of Chicago, Chicago, Illinois 60637, USA* **3** *Department of Earth Sciences, Montana State University, Bozeman, Montana 59717, USA* **4** *School of the Environment, Geography and Geological Sciences, University of Portsmouth, Portsmouth PO1 3QL, UK* **5** *Centre de Recherche sur la Paléobiodiversité et les Paléoenvironnements, UMR7207 (CNRS-MNHN-UPMC), Muséum national d'Histoire naturelle, 75005 Paris, France* **6** *School of Museum Studies, University of Leicester, Leicester LE1 7RF, UK* **7** *Laboratoire Géosciences, Département de Géologie, Faculté des Sciences Aïn Chock, Université Hassan II, Casablanca, Morocco* **8** *Redpath Museum, McGill University, Montreal, Quebec H3A 0C4, Canada* **9** *Laboratoire de Biodiversité et Santé, Faculté des Sciences Aïn Chock, Université Hassan II, Casablanca, Morocco*

Corresponding author: Nizar Ibrahim (ibrahini@udmercy.edu), Paul C. Sereno (dinosaur@uchicago.edu)

Academic editor: Massimo Delfino | Received 23 October 2019 | Accepted 2 March 2020 | Published 21 April 2020

http://zoobank.org/6C47C920-CBBC-4A7C-A05F-1E12D13076F1

Citation: Ibrahim N, Sereno PC, Varricchio DJ, Martill DM, Dutheil DB, Unwin DM, Baidder L, Larsson HCE, Zouhri S, Kaoukaya A (2020) Geology and paleontology of the Upper Cretaceous Kem Kem Group of eastern Morocco. ZooKeys 928: 1–216. https://doi.org/10.3897/zookeys.928.47517

Abstract

The geological and paleoenvironmental setting and the vertebrate taxonomy of the fossiliferous, Cenomanian-age deltaic sediments in eastern Morocco, generally referred to as the "Kem Kem beds", are reviewed. These strata are recognized here as the Kem Kem Group, which is composed of the lower Gara Sbaa and upper Douira formations. Both formations have yielded a similar fossil vertebrate assemblage of predominantly isolated elements pertaining to cartilaginous and bony fishes, turtles, crocodyliforms, pterosaurs, and dinosaurs, as well as invertebrate, plant, and trace fossils. These fossils, now in collections around the world, are reviewed and tabulated. The Kem Kem vertebrate fauna is biased toward large-bodied carnivores including at least four large-bodied non-avian theropods (an abelisaurid, *Spinosaurus*, *Carcharodontosaurus,* and *Deltadromeus*), several large-bodied pterosaurs, and several large crocodyliforms.

Copyright *Nizar Ibrahim et al.* This is an open access article distributed under the terms of the Creative Commons Attribution License (CC BY 4.0), which permits unrestricted use, distribution, and reproduction in any medium, provided the original author and source are credited.

No comparable modern terrestrial ecosystem exists with similar bias toward large-bodied carnivores. The Kem Kem vertebrate assemblage, currently the best documented association just prior to the onset of the Cenomanian-Turonian marine transgression, captures the taxonomic diversity of a widespread northern African fauna better than any other contemporary assemblage from elsewhere in Africa.

Keywords
Africa, Cretaceous, dinosaur, Gara Sbaa Formation, Douira Formation, paleoenvironment, vertebrate

Table of contents

Introduction ... 3
  Geological status and presumed age ... 3
  Paleoenvironmental and paleoecological interpretations ... 7
  Fossil discoveries ... 8
  Vertebrate fauna ... 8
Institutional and collections abbreviations ... 9
Materials and methods ... 10
  Fieldwork ... 10
  Collections research ... 13
  Geographic feature names ... 14
Geology ... 15
  Geological context ... 15
  Hamadian Supergroup ... 25
  Kem Kem Group ... 27
  Gara Sbaa Formation ... 27
  Douira Formation ... 35
  Paleogeography and paleoenvironments ... 45
  Age ... 51
Taphonomy ... 53
Systematics ... 64
  Plant and nonvertebrate fossils ... 64
  Elasmobranchii Bonaparte, 1838 ... 64
  Actinopterygii Klein, 1885 ... 73
  Sarcopterygii Romer, 1955 ... 76
  Amphibia Gray, 1825 ... 82
  Testudines Batsch, 1788 ... 83
  Squamata, Oppel, 1811 ... 91
  Crocodyliformes Hay, 1930 ... 92
  Pterosauria Kaup, 1834 ... 112
  Dinosauria Owen, 1842 ... 125
Discussion ... 172
  Stratigraphic resolution ... 172

Biostratigraphic resolution 173
Taxonomic resolution........ 174
Kem Kem assemblage........ 176
Comparable African assemblages........ 182
Conclusions 185
Kem Kem paleoenvironments 185
Kem Kem paleoecosystem........ 187
Acknowledgements........ 189
References 190

Introduction

Richly fossiliferous strata, commonly referred to as the "Kem Kem beds" (Lavocat 1949, Sereno et al. 1996), are exposed on the face of a long, winding escarpment near the Moroccan-Algerian border on the northwestern edge of the Sahara Desert (Figs 1–3). Secondary outcrops of similar rocks extend westward toward the Atlas Mountains from this escarpment at Erfoud to Jorf and eventually to Goulmima and Asfla. At distant locales in northern Africa, early geological and paleontological surveys identified comparable fossiliferous rocks in the Western Desert of Egypt (Stromer 1915, 1934, Nothdurft et al. 2002) and in north and central regions of the Sahara (Haug 1904, Lapparent 1951, 1960, Depéret and Savornin 1927).

The Kem Kem beds, nevertheless, are more fossiliferous, better exposed and often more accessible than comparable strata in most other northern African locations. These strata have been studied by several teams and are accessible to locals in some areas; fossils have been collected by researchers affiliated with institutional collections as well as local private collectors that often utilize commercial intermediaries. Our aim in this report is to review both the geological and paleontological aspects of the Kem Kem beds, to describe and name strata as needed, to summarize the taxonomic status of the fauna based on all major collections of Kem Kem fossils, and to evaluate paleoenvironments and the paleoecological significance of the Kem Kem assemblage.

Geological status and presumed age

Lavocat (1954a, 1954b) referred to the strata in the Kem Kem area of Morocco as a component of the "Continental intercalaire", a term used for broadly comparable rocks in many other locales in northern Africa that are capped by a distinctive hard limestone platform of Cenomanian-Turonian age (Figs 3, 4). Lavocat (1948, 1954a) and Choubert (1952) also referred to this continental-marine package of rock as the "trilogie mésocrétacée", comprising two successive continental units underlying the marine Cenomanian-Turonian limestone. The continental beds were described by Joly (1962) as the "unité inférieure", or "grès rouges infracénomaniens", and the "unité supérieure",

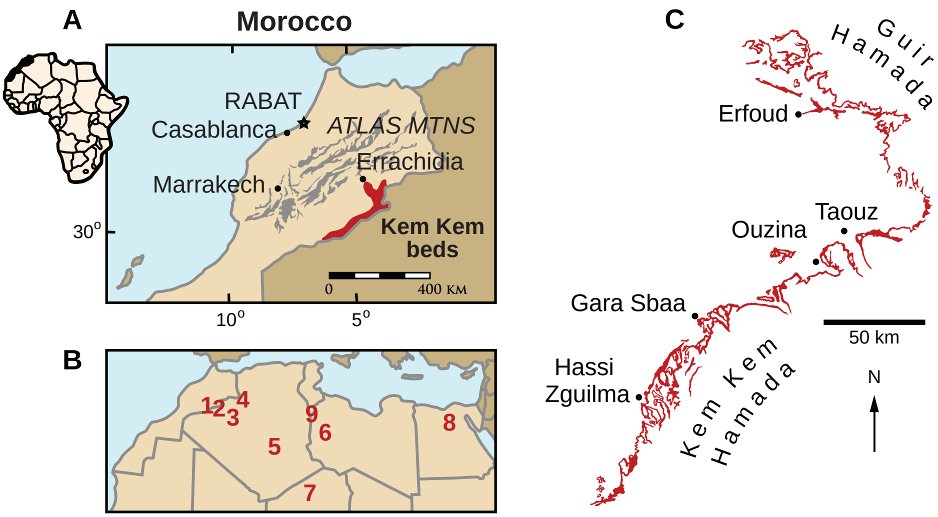

Figure 1. Geographical setting of the Kem Kem region and outcrops. **A** View of the position of Morocco in Africa and location of the Kem Kem beds (shown in red). **B** Map showing the geographical location of the Kem Kem in North Africa relative to roughly coeval sites in northern Africa. **C** Cretaceous outcrops along the Kem Kem and Guir Hamadas (modified from Sereno et al. 1996). Numbers: **1** Kem Kem, Morocco. **2** Gara Samani, Algeria. **3** Timimoun, Algeria. **4** Monts des Ksours, Algeria. **5** Djoua Valley, Algeria. **6** Al Hamra Hamada, Libya. **7** In Abangharit, Niger. **8** Bahariya, Egypt, **9** Tataouine, Tunisia.

or "marnes versicolores à gypse." This two-part division of the continental facies beneath the Cenomanian-Turonian limestone along the escarpment in the Kem Kem region of Morocco has thus been recognized for nearly 70 years, although no formal geological nomenclature has been proposed for these fossiliferous continental facies.

Sereno et al. (1996) introduced the informal term Kem Kem beds for the two lower units of Choubert's (1952) "trilogie mésocretacée", which underlie the Cenomanian-Turonian limestone complex (Fig. 3). These lower beds, composed of sandstone and mudstone, reach a maximum thickness of approximately 200 m. More recently, Ettachfini and Andreu (2004) and Cavin et al. (2010) used formational names originally proposed in notes to a geological map of the High Atlas to the northwest of the Kem Kem by Dubar (1949; sometimes mis-cited as 1948). Dubar's formational names (Ifezouane and Aoufous) are based on his observations of strata on the eastern flank of the High Atlas Mountains (Tinghir, west of Goulmima). As we discuss below, these rocks are generally un-fossiliferous, have a greater presence of evaporite facies, and lack other features of both terrestrial units of the Kem Kem beds (Sereno et al. 1996). Thus, although we do correlate the two units of the Kem Kem beds with the Ifezouane and Aoufous formations on a continuum of relatively small interconnected basins, we show that the Kem Kem units are mappable, distinctive and deserving of formal recognition, and we designate effective type sections for each. We summarize the latest geological and paleontological evidence that suggests that they accumulated on a continental ramp in the Kem Kem region through to ocean margins to the north and west (Fig. 2).

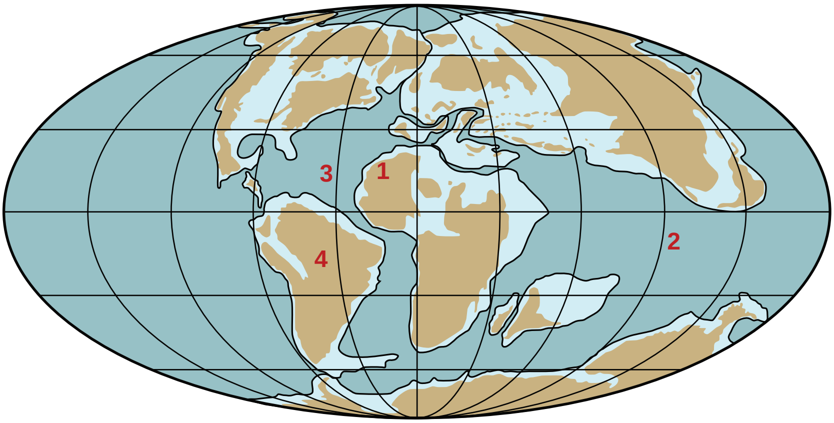

Figure 2. Geographical setting of the Kem Kem region. Cenomanian (~94 Mya) paleogeographic world map showing key localities (map after Scotese 2002, da Silva and Gallo 2007). Abbreviations: **1** Kem Kem region in Africa **2** Tethys Ocean **3** opening Atlantic Ocean **4** South America.

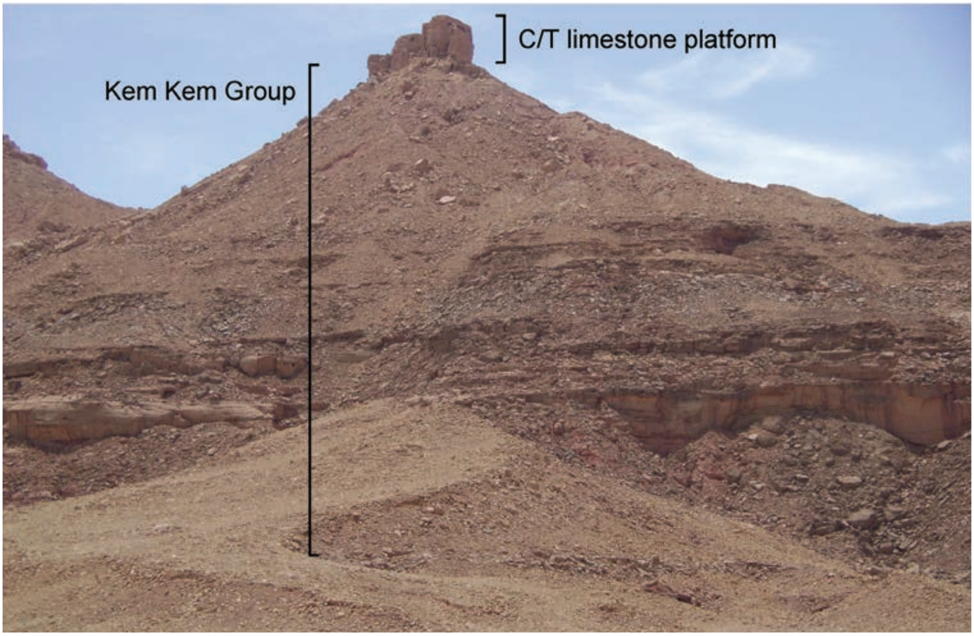

Figure 3. Outcrops of the Kem Kem sequence near Gara Sbaa. The red beds are overlain by a Cenomanian-Turonian limestone platform.

The age of the "Kem Kem beds" has been regarded variously as mid or early Late Cretaceous (Albian-Cenomanian). Lavocat (1948) described the most common faunal elements and estimated the age as Albian or Cenomanian. Choubert (1952) and Lavocat (1954b) noted similarities with the Bahariya Formation in Egypt (Stromer

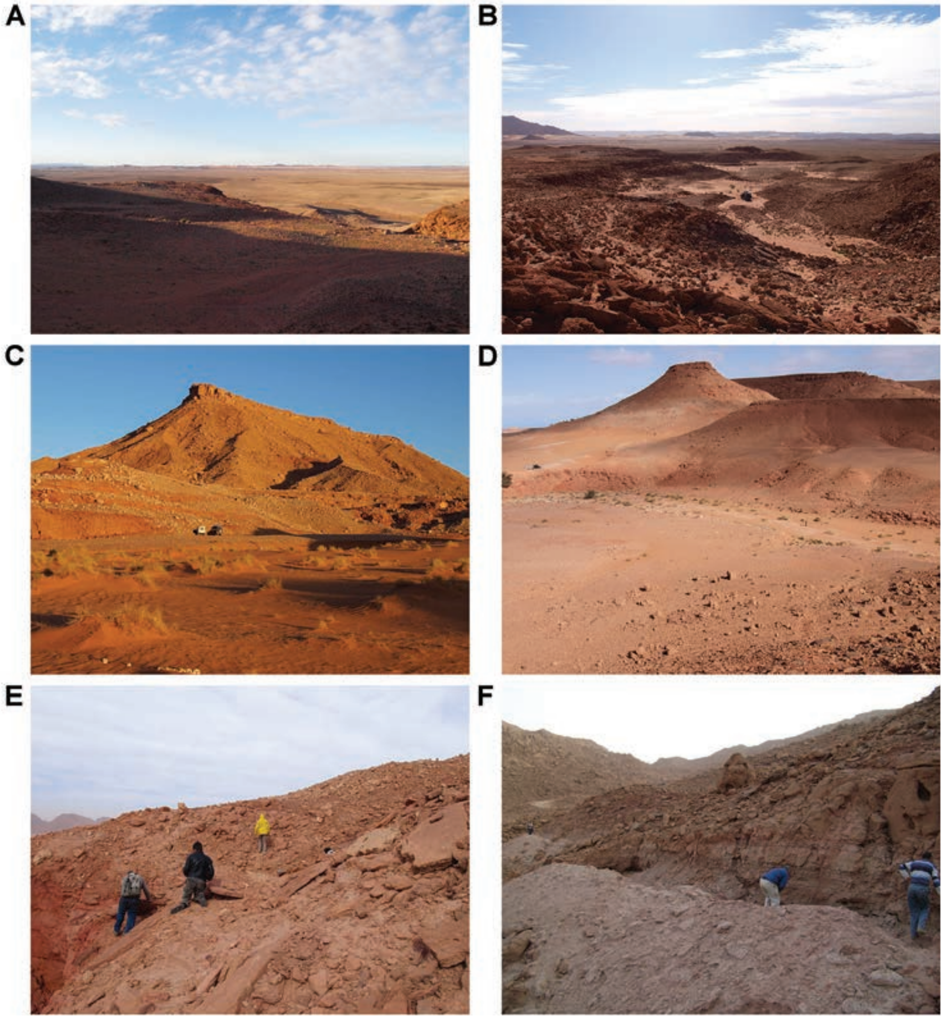

Figure 4. Example Kem Kem localities. **A** Basal outcrop at Aferdou N'Chaft **B** Iferda N'Ahouar **C** Gara Sbaa **D** Other outcrops at Gara Sbaa **E** Outcrops south of Jbel Zireg **F** Outcrop at Moher (south of Tafraoute) near the Morocco-Algeria border.

1936, Dominik 1985, Soliman and Khalifa 1993), which was regarded as Cenomanian in age. Choubert (1952) and Lavocat (1954a), however, assigned the Kem Kem beds to the "infracénomanien," suggesting a probable Albian age. Although ammonites and other nonvertebrate fossils have established the Late Cenomanian-Turonian age of the overlying limestone complex (Ferrandini et al. 1985; Ettachfini and Andreu 2004; Ettachfini et al. 2005; Essafraoui et al. 2015), no fossils were known at that time from the Kem Kem beds that could be reasonably associated with stage-level temporal resolution.

Seven elasmobranchs and several dinosaur genera (*Spinosaurus*, *Carcharodontosaurus,* and *Deltadromeus*) reported from the Kem Kem beds are shared with the Bahariya Formation in Egypt (Sereno et al. 1996). One of these elasmobranch species (*Haimirichia amonensis*; Cappetta and Case 1975), in addition, has a broad circum-Mediterranean distribution seemingly restricted to Cenomanian strata. As the fauna from both lower and upper units appears similar, Sereno et al. (1996) inferred a Cenomanian age for these sediments, which has generally been accepted (Wellnhofer and Buffetaut 1999, Cavin et al. 2001, Dal Sasso et al. 2005, Cavin et al. 2010). Martin and de Lapparent de Broin (2016) recently reviewed the geology and age of the Kem Kem beds and proposed a late Albian-early Cenomanian age for the locality that yielded *Lavocatchampsa*.

Paleoenvironmental and paleoecological interpretations

Paleoenvironmental interpretations of the Kem Kem beds all agree on their continental status, but differ in regarding either fluvial and floodplain deposits (Russell 1996), or deltaic facies with rare lacustrine environments, as predominant (Sereno et al. 1996). Cavin et al. (2010) described the Kem Kem beds as mostly terrestrial with local brackish and freshwater deposits.

Paleoecological interpretation has centered around the taxonomic and numerical dominance of predators and, more specifically, piscivorous terrestrial and aquatic vertebrates. Based on a collection of commercially acquired fossils, Russell (1996) suggested that the abundance of fish served as the primary resource for a diversity of piscivorous crocodyliforms and theropods, and later authors have added supporting evidence (Sereno et al. 1996, Russell and Paesler 2003, Läng et al. 2013, Ibrahim et al. 2014a). Others have regarded this as an oversimplification of a more complex food web (McGowan and Dyke 2009, Cavin et al. 2010).

A notable feature of the Kem Kem assemblage is the taxonomic, numerical and ichnological dominance of theropods among dinosaurs. Some authors regard this as an accurate reflection of the dominance of theropods in the fauna during the Cenomanian (Russell 1996, Mahler 2005, Läng et al. 2013, Ibrahim et al. 2014a, Ibrahim et al. 2014b, Ibrahim et al. 2016). Others have suggested that the perceived diversity (Dyke 2010) and abundance (McGowan and Dyke 2009, Cavin et al. 2010, Dyke 2010) of theropods is a result of geological or collecting biases. As many of the fossils are based on isolated remains, there are ongoing questions over the taxonomic diversity of some subgroups including theropods (e.g., Evers et al. 2015).

With a similar approach, it has been suggested that Kem Kem fossils as a whole represent a "compound assemblage" derived from two formations (Cavin et al. 2010) or from disparate paleoenvironments (Dyke 2010). The geological and paleontological evidence reviewed in this report bears directly on these controversies.

Fossil discoveries

In the late 1940s, Choubert (1948) described bony fish from the Kem Kem beds exposed on the escarpment of the Guir Hamada along the Morocco-Algeria border. From 1948 to 1951, Lavocat brought to light a range of vertebrate fossils, prospecting and collecting on camelback and vehicle along the escarpment formed by the Guir and Kem Kem Hamadas (Lavocat 1949, 1954a, 1954b, Lapparent 1958). His best-known fossil discovery is the partial skeleton of the diplodocoid sauropod, *Rebbachisaurus garasbae* (Lavocat 1954b, Wilson and Allain 2015) from the locality Gara Sbaa (Fig. 4C).

During the following 45 years from 1950–1995, only sporadic, small-scale fieldwork was undertaken. In the 1970s a small team led by German scientist Helmut Alberti collected fossil vertebrates near Taouz at the northeastern end of the escarpment of the Kem Kem Hamada (pers. com. M. Reich to NI, 2007). The fossils, housed at the University of Göttingen, include isolated remains of cartilaginous and bony fish, crocodyliforms and non-avian dinosaurs. The coelacanth remains were described by Wenz (1981), and some jaw remains were referred to *Spinosaurus* (Buffetaut 1989, 1992).

Russell (1996) described a collection of commercially acquired fossils, probably collected from the Kem Kem beds, assigning specimens to *Carcharodontosaurus saharicus*, a new species, *Spinosaurus maroccanus*, a new genus and species *Sigilmassasaurus brevicollis*, and an unnamed abelisaurid. Sauropod bones were referred to *Rebbachisaurus garasbae* and a family of basal titanosauriforms.

In the last 25 years, paleontologists brought to light a diverse array of new vertebrate fossils (e.g., Sereno et al. 1996, Ibrahim et al. 2010, Cavin et al. 2010, Ibrahim et al. 2014a). Commercial fossil collecting in the Kem Kem beds has also accelerated during this time. Many of the fossils described in recent years were acquired from commercial sources of uncertain geographic origin (e.g., Wellnhofer and Buffetaut 1999, Gaffney et al. 2002, Milner 2003, Evers et al. 2015, Hendrickx et al. 2016). These specimens, with very rare exception, are isolated bones recovered from channel deposits with no locality information or taxonomic association.

Vertebrate fauna

The Kem Kem beds have revealed an important and remarkably diverse vertebrate assemblage including elasmobranchs, osteichthyes, and basal sarcopterygians (Choubert 1939, Tabaste 1963, Wenz 1981, Forey 1997, Forey and Grande 1998, Taverne and Maisey 1999, Cavin and Dutheil 1999, Dutheil 1999a, 1999b, Cavin and Brito 2001, Taverne 2000, 2004, Cavin and Forey 2001a, 2001b, Filleul and Dutheil 2001, 2004, Cavin and Forey 2004, Forey and Cavin 2007, Cavin et al. 2010, Martill and Ibrahim 2012), amphibians (Rage and Dutheil 2008), lepidosauromorphs (Rage and Dutheil 2008, Apesteguía et al. 2016a, Klein et al. 2017), turtles (Gmira 1995, Tong and Buffetaut 1996, Gaffney et al. 2002, 2006), crocodyliforms (Buffetaut 1976, 1994, Larsson and Sidor 1999, Larsson and Sues 2007, Sereno and Larsson 2009, Martin and de

Lapparent de Broin 2016, Young et al. 2017), pterosaurs (Wellnhofer and Buffetaut 1999, Mader and Kellner 1999, Ibrahim et al. 2010, Rodrigues et al. 2011, Martill and Ibrahim 2015, Martill et al. 2018, 2020, Jacobs et al. 2019, 2020, McPhee et al. 2020), non-avian dinosaurs (Lavocat 1948, 1951, 1952, 1954b, Buffetaut 1989, 1992, Sereno et al. 1996, Russell 1996, Milner 2003, Amiot et al. 2004, Novas et al. 2005a, Mahler 2005, Dal Sasso et al. 2005, Ibrahim at al. 2014a, Ibrahim et al. 2014b, Evers et al. 2015, Ibrahim et al. 2016, Chiarenza and Cau 2016, Ibrahim et al. 2017), and a possible avian (Cavin et al. 2010).

Kem Kem vertebrates are typically preserved in two general taphonomic situations, most commonly in clastic fluvial or deltaic facies or, rarely, within a lake, or pond, facies at the locality Oum Tkout. In the predominant fluvial facies, isolated and transported fossils are the norm. Only three associated partial dinosaur skeletons have been recovered, the diplodocoid sauropod *Rebbachisaurus garasbae* (Lavocat 1954b, Wilson and Allain 2015) and the theropods *Deltadromeus agilis* (Sereno et al. 1996) and *Spinosaurus aegyptiacus* (Ibrahim et al. 2014b). In addition, one associated dinosaur skull pertaining to the large theropod *Carcharodontosaurus saharicus* has been recovered (Sereno et al. 1996). Because of the prevalence of isolated remains, taxonomic identification of some Kem Kem vertebrates remains controversial, with some authors splitting and others lumping recorded taxonomic diversity (Russell 1996, Sereno et al. 1996, Mahler 2005, Rauhut and López-Arbarello 2006, Averianov et al. 2008, Cavin et al. 2010, Ibrahim et al. 2014b, Evers et al. 2015, Ibrahim et al. 2017).

The lentic waters and fine bottom mud at Oum Tkout preserve leaves, crustaceans (prawn, macruran decapod), and the intact skeletons and scales of bony fish (Dutheil 1999b, Filleul and Dutheil 2001, 2004, Garassino et al. 2006). The specimens are acquired by quarrying and splitting blocks followed by fine preparation. Multiple specimens of a given taxon are often preserved.

Institutional and collections abbreviations

**BSPG**	Bayerische Staatssammlung für Paläontologie und Geologie, Munich, Germany (formerly BSP)
**CMN**	Canadian Museum of Nature, Ottawa, Canada (formerly NMC)
**FMNH**	Field Museum of Natural History, Chicago, USA
**FSAC**	Faculté des Sciences Aïn Chock, Casablanca, Morocco
**IMGP**	Institut und Museum für Geologie und Paläontologie, University of Göttingen, Göttingen, Germany
**LINHM**	Long Island Natural History Museum, Long Island, USA
**MN**	Museu Nacional/Universidade Federal do Rio de Janeiro, Rio de Janeiro, Brazil
**MFN**	Museum für Naturkunde, Berlin, Germany
**MNHN**	Muséum national d'Histoire naturelle, Paris, France
**MNBH**	Musée national Boubou Hama, Niamey, Niger

**MPDM**	Musée Parc des Dinosaures, Mèze, France
**MSNM**	Museo Civico di Storia Naturale, Milan, Italy
**NHMUK**	Natural History Museum, London, United Kingdom
**ROM**	Royal Ontario Museum, Toronto, Canada
**SGM**	Service Géologique du Maroc, Rabat, Morocco
**UCRC**	University of Chicago Research Collection, Chicago, USA

Materials and methods

Fieldwork

In 1995 a joint expedition from the University of Chicago and the Service Géologique du Maroc explored southern outcrops of the Kem Kem beds beween Erfoud and Hassi Zguilma (Fig. 1C). Geological sections were logged at intervals, which comprise the majority of the stratigraphic sections presented in this report (Sereno et al. 1996). Notable paleontological finds included the discovery of a partial postcranial skeleton of the theropod *Deltadromeus agilis* in the upper part of the lower unit. Near the base of the upper unit, a pond locality named Oum Tkout was discovered with complete remains of decapods and articulated bony fish (Dutheil, 1999b). The upper unit also yielded a partial skull of the theropod dinosaur *Carcharodontosaurus saharicus* and footprint horizons including the only record to date of an ornithischian dinosaur in the Kem Kem assemblage (Sereno et al. 1996). Dozens of macro- and micro-vertebrate fossil localities were mapped and hundreds of isolated fossils and footprints were collected. These specimens currently reside in the University of Chicago Research Collection.

In 2007 and 2008, joint expeditions from University College Dublin, the University of Portsmouth and the Faculté des Sciences Aïn Chock (Casablanca) collected fossils and recorded ichnological, taphonomic, and sedimentological data, focusing on seven sites between Erfoud and Hassi Zguilma (Tables 1–3, Figs 4, 5; Ibrahim et al. 2014a, 2014b, 2016). Vertebrate fossils include bony fishes, turtles, crocodyliforms, the large azhdarchid *Alanqa saharica* (Ibrahim et al. 2010, Martill and Ibrahim 2015), and dinosaurs, including a partial humerus of a large titanosaurian sauropod (Ibrahim et al. 2016). These specimens reside in the collections of the Faculté des Sciences Aïn Chock in Casablanca.

In 1999, 2000, 2002, and 2011, field work was undertaken at the pond locality Oum Tkout by a joint team from the Muséum national d'Histoire naturelle, the Service géologique du Maroc, and the University Cadi Ayyad. Quarrying operations resulted in the discovery of numerous nonvertebrate fossils (plants, insects, ostracods, decapods, etc.) as well as articulated elasmobranchs and actinopterygians. Much of this material remains to be thoroughly prepared and studied and likely represents several new taxa.

In 2013, field work was undertaken by a joint team from the University of Chicago, the Museo Civico di Storia Naturale (Milan), and the Faculté des Sciences Aïn

Table 1. Presence in three research collections of specimens from 15 localities in the Kem Kem Group. Abbreviations: FSAC Faculté des Sciences Aïn Chock, Casablanca, Morocco MNHN Muséum national d'Histoire naturelle, Paris, France UCRC University of Chicago Research Collection, Chicago, USA.

Number	Locality	FSAC	MNHN	UCRC
1	Aferdou N'Chaft/Ouzina	✓	✓	✓
2	Boumerade/Gara Acacia	✓	✓	
3	Dar el Karib			✓
4	Douira	✓		✓
5	Gara Sbaa	✓	✓	✓
6	Gara Tabroumit		✓	
7	Iferda N'Ahouar	✓		✓
8	Kouah Trick		✓	
9	Moher	✓		
10	Oum Tkout			✓
11	Talidat			✓
12	Taouz	✓		✓
13	Valley near Boumerade	✓		
14	Zguilma	✓	✓	✓
15	Zrigat	✓		

Table 2. Six fossil vertebrate localities with geographic coordinates prospected during field work in 2008. Coordinates for other localities may be obtained with permission from the authors.

Number	Locality	Geographic coordinates	
		North	East/West
1	Aferdou N'Chaft	30°53'51.23"N	3°52'13.42"E
2	Boumerade	30°32'49.00"N	4°42'55.45"E
3	Douira	31°38'16.93"N	4°20'20.23"E
4	Gara Sbaa	30°30'40.64"N	4°50'42.87"E
5	Iferda N'Ahouar	30°47'54.33"N	4°22'43.74"W
6	Zguilma	30°12'07.61"N	5°7'11.48"E

Table 3. Taphonomic stages for bone abrasion from transport (following Anderson et al. 2007).

Stage	Identification	Description
1	Very angular	Bone and teeth fresh and unabraded
2	Subangular	Bone edges slightly abraded and polished
3	Subrounded	Bone edges moderately rounded
4	Rounded	Bone edges and processes broken and rounded
5	Extremely rounded	Marked abrasion of all external surfaces

Chock (Casablanca) to explore a locality (Zrigat) approximately 20 km north of Erfoud, where a partial skeleton of *Spinosaurus aegyptiacus* was discovered by a local collector (Ibrahim et al. 2014b: fig. 1A). Additional fragmentary pieces of this specimen were recovered at the site, and the specimen is catalogued in the collections of the Faculté des Sciences Aïn Chock. A geological section was logged across both units of the Kem Kem beds at the nearby Al Gualb Mesa, positioning this locality at the base of

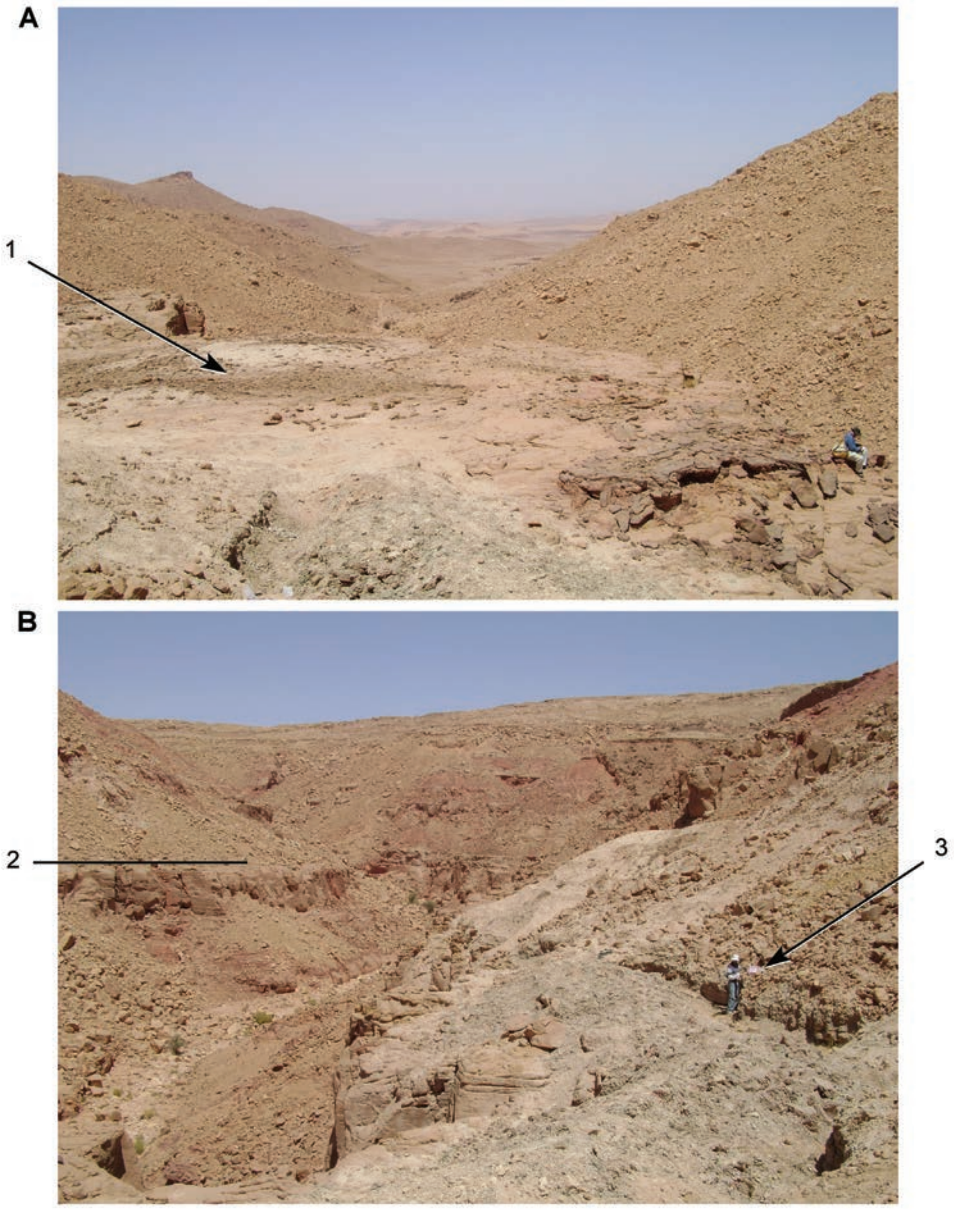

Figure 5. Outcrop near Boumerade, identified in 2008. **A** Flat erosional surface yielding fossils (turtle and pterosaur) **B** Sloped erosional surfaces yielding fossils (turtle). Abbreviations: **1** Collecting surface **2** Boundary between upper and lower members **3** Locality of a partial turtle carapace.

the upper unit (Ibrahim et al. 2014b: fig. 1B). Additional fieldwork in the Kem Kem was performed in 2015 on an expedition led by one of us (NI).

In 2018 and 2019, a multidisciplinary team of scientists from the University of Detroit Mercy, the Faculté des Sciences Aïn Chock, the Museo Civico di Storia Natu-

rale (Milan), and the University of Portsmouth, led by NI, explored a number of sites along the Kem Kem escarpment, including several new localities. Finally, regular field-work by University of Portsmouth researchers and students has continued to grow the Casablanca collection (Faculté des Sciences Aïn Chock).

Collections research

Besides major collections in Casablanca (Faculté des Sciences Aïn Chock), Paris (Muséum national d'Histoire naturelle), and Chicago (University of Chicago), additional collections were assembled over the last 50 years from privately acquired specimens collected by locals in villages near the Kem Kem escarpment, although without specific locality data. During a general survey of the Kem Kem beds in 1995, excavation pits in channel sandstones made by local collectors were observed along the entire length of the outcrop. Commercial collecting was therefore already well established by the mid-1990s along most of the available outcrop of the Kem Kem beds with activity concentrated in the northern one-half between Erfoud and Taouz.

The most important collections of commercially collected fossils are in Canada (CMN, Ottawa; ROM, Toronto) and Europe (MNHM, Paris; MPDM, Mèze; BSPG, Munich; MSNM, Milan; NHMUK, London). Several authors (PCS, DMM, DBD, HCEL) have visited some of these collections; one author (NI) has visited all the major collections in the course of doctoral research at University College Dublin, collecting quantitative data on thousands of specimens (Fig. 6, Table 3).

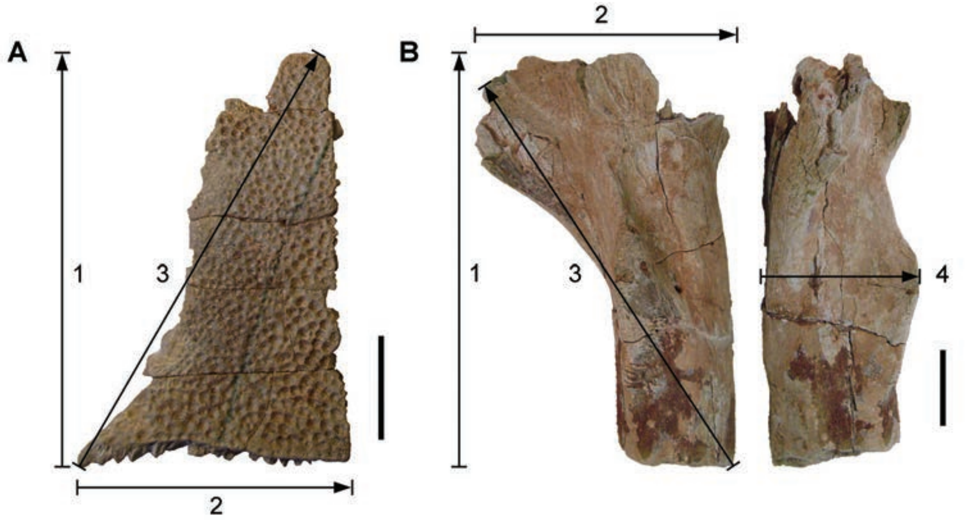

Figure 6. Examples of specimen measurements. **A** Turtle carapace fragment (MRS 172, MNHN specimen) **B** Femur (MPDM 270) in anterior and lateral view. Scale bars equal 2 cm in **A** and 10 cm in **B**. Abbreviations: **1** Length of specimen **2** Width measurement **3** Maximum length measurement **4** Depth measurement.

Geographic feature names

Geographic feature names are often cited or adopted for geographic locations, fossil localities and geological terms. Whereas the Geological Survey maintains an authoritative federal database for geographic feature names in the United States (Geographic Names Information System), no such resource is currently available for Morocco. In the Sahara, geographic feature names often originate in Arabic or Amazigh (Berber) languages and exhibit considerable spelling variation in scholarly papers in German, French or English. Sometimes the meaning of feature names is lost, as may be the case with the most salient feature name in this study, Kem Kem. If a specific meaning to feature names persists, nevertheless, that meaning is often unknown to western scholars. To remedy that situation, we have compiled a list of important fossil localities in the Kem Kem region along with their meanings and spelling variants (Table 4).

Variation in usage can run counter to the original meaning of the name or the feature to which it was originally applied. The term "Tafilalt", for example, is an Amazigh word referring to a jar made of clay for water and was used as a feature name for the valley of oases south of Errachidia in eastern Morocco. It is used to refer to pre-Mesozoic (mostly Paleozoic) outcrops in the scientific literature (e.g., Wendt et al. 1984, Baidder et al. 2016). It should not be used to describe the Kem Kem escarpment or the location of the Kem Kem vertebrate fauna (Russell 1996), which derives from a larger area extending much farther to the south.

Other variation in geographic names is the result of official name-changing, with older names on geological maps and reports supplanted by newer names. "Ksar es-Souk", for example, is Arabic meaning "fortified village of the market" and was the longstanding name of a pivotal city in east-central Morocco. In the 1970s it was renamed "Errachidia". Both names have several spelling variants. Neither should be used with "Province" as the location for the entirety of the Kem Kem outcrop (e.g., Kellner and Mader 1997, Weishampel et al. 2004). The relatively large administrative area of Errachidia Province does not include the southern one-half of the exposures of the Kem Kem beds.

Some geographic names are simple errors that gain traction in secondary citations. In a prominent compilation of dinosaur localities, for example, the term "Tegana Formation" was cited for the "Kem Kem beds" (Weishampel et al. 1990). This may have arisen as a misspelling of the "Tegama Group", a name for Cretaceous age beds in Niger. Although the error was noted (Sereno et al. 1996), it has reappeared in subsequent publications (e.g., Bailey 1997, Kellner and Mader 1997, Taverne and Masey 1999, Weishampel et al. 2004).

In this report, we formally name several geological units, abiding by the guidelines of the North American Stratigraphic Code (NACSN 2005). Sereno et al. (1996) previously introduced the informal geological term "Kem Kem beds" for the fossiliferous outcrop of the Kem Kem region. Some authors have mistakenly referred to the "Kem Kem Formation" (e.g., McGowan and Dyke 2009, Holliday and Gardner 2012), when no formal geological unit by that name was ever proposed.

Table 4. Nomenclature of Kem Kem localities and geographic terms and their meanings in Arabic or Amazigh (Berber language).

Locality/Term	Meaning	Synonym/Variant
Aferdou N'Chaft	(aferdou) mortar, (n'chaft) "of the pass" (Amazigh)	"El Begâa" (Cavin et al. 2010) refers to the closest village near the locality here identified as Aferdou N'Chaft
Boumerade	acacia (Amazigh)	"Gara Acacia" by Lavocat on field records and museum labels for the locality here identified as Boumerade
Douira	small house (Arabic)	"Jorf" refers to the closest village to the outcrop here identified as Douira
Gara Sbaa	lion hill or mound (Arabic)	"Gara es Sbaa" (Sereno et al. 1996), "Gara Sbâa" (Cavin et al. 2010)
Hamada	plateau or platform (Arabic)	–
Iferda N'Ahouar	(iferda) mortars, (n'ahouar) large plate (Amazigh)	"Er Remlia" (Sereno et al. 1996) refers to the closest village to the outcrop here identified as Iferda N'Ahouar
Oum Tkout	particular *Tamarix* tree (Amazigh)	"Oum Tkiout" (Martill and Ibrahim 2015)
Talidat	little finger (Amazigh)	–
Tamenkhirt	common songbird (wheatear) (Amazigh)	–

Geology

Geological context

"Continental intercalaire". The Kem Kem beds correlate to the top of a package of continental sediments in basins across northern Africa referred to by Kilian (1931) as the "Continental intercalaire", or the "intercalated continental (deposits)". In many regions, the beds assigned to the "Continental intercalaire" discordantly overlie Paleozoic marine sediments that Kilian termed the "Continental de base". They are overlain, in turn, by a prominent limestone plateau and younger sediments he termed the "Continental terminal". Additional informal subdivisions (between the three outlined by Kilian; Table 5) have been inserted by geologists and paleontologists from time to time, prominent among them Lapparent and Lelubre (1948). Despite its widespread use, "Continental intercalaire" remains an evolving, poorly defined stratigraphic term. Below we question its utility.

The stratotype for the "Continental intercalaire" is located in the Djoua Valley in Algeria. Rocks of comparable age are located in continental basins to the east in Libya and Egypt, to the north and west in Tunisia, Morocco and Mauritania, and to regions south of the Hoggar Mountains in Niger (Lavocat 1954a, Bellion et al. 1990, Lefranc and Guiraud 1990, Sereno et al. 1996, Benton et al. 2000, O'Leary et al. 2004). Lapparent and Lelubre (1948) and Lefranc (1958) described and divided strata of the "Continental intercalaire" across northern Africa, delineating a number of "series" (Table 6).

The lower boundary is problematic. It does not correspond to any geological event across northern Africa. Kilian (1931) suggested the lower boundary should include all marine sediments of Carboniferous age (thus including the Tiguentourine series; Table 6). Later papers inferred a Triassic age for the earliest beds (Kilian 1937, Lefranc 1958, 1963, Busson 1970), whereas Lapparent (1949) included only the Cretaceous

Table 5. Divisions of northern African sediments. Kilian's (1931) three terms (in italics), defined in the Djoua Valley near the Algerian-Libyan border, were supplemented by Lapparent and Lelubre (1948) (modified from Lefranc and Guiraud 1990). Abbreviation: ICS International Commission on Stratigraphy.

ICS timescale	Division
Danian-Pleistocene	*Continental terminal*
Late Cenomanian-Danian	Continental hamadien
Namurian-Late Cenomanian	*Continental intercalaire*
Frasnian-Namurian	Continental post-tassilien
Ordovician-Frasnian	Continental tassilien
Cambrian	*Continental de base*

Table 6. Subdivisions of the "Continental intercalaire" introduced by Lapparent and Lelubre (1948) and Lefranc (1958) (modified from Lefranc and Guiraud 1990). Abbreviation: ICS International Commission on Stratigraphy.

Division	Sediment series	ICS timescale
Hamadian series	limestone platform	Cenomanian-Turonian
Continental intercalaire	Djoua series	Early Cretaceous
	Taouratine series	Jurassic
	Zarzaitine series	Triassic
	Tiguentourine series	Late Carboniferous (Pennsylvanian)-Permian
Post-tassilian series	marine limestone	Early Carboniferous (Mississippian)

outcrops of the Djoua series (Table 5) in the "Continental intercalaire". In their review, Lefranc and Guiraud (1990) identified a lower boundary that includes Late Paleozoic strata "younger than Namurian", although many of these largely un-fossiliferous beds are difficult to correlate. Some authors exclude contemporaneous continental rocks in coastal basins from the "Continental intercalaire". Cavin et al. (2010: 393), for example, exclude the fossiliferous Upper Cretaceous strata in marginal basins along Morocco's Atlantic coast.

The upper boundary of the "Continental intercalaire", in contrast, is bounded by a well-dated, fossiliferous Late Cenomanian-Turonian limestone platform (Fig. 7), which may be the reason the term has persisted. The limestone platform records a sudden global transgression (Gale 2000) that is well exposed in Morocco (Kilian 1931, Lefranc and Guiraud 1990, Choubert 1948, Lavocat 1954a, Ferrandini et al. 1985, Ettachfini and Andreu 2004). The nearshore and terrestrial strata underlying the platform in Morocco correspond with the Djoua series in Algeria, although their precise correlation is poorly constrained. These underlying strata include the Kem Kem beds, which rank amongst the most fossiliferous continental beds of the "Continental intercalaire".

In sum, there is no consensus regarding the lower boundary of the "Continental intercalaire", which has never been tied to any regional geological event or episode. Some coeval continental rocks of Cretaceous age, in addition, are excluded because of their location in coastal basins. For these reasons, we question the continued use of "Continental intercalaire" as a heuristic term in discussions of northern African continental strata.

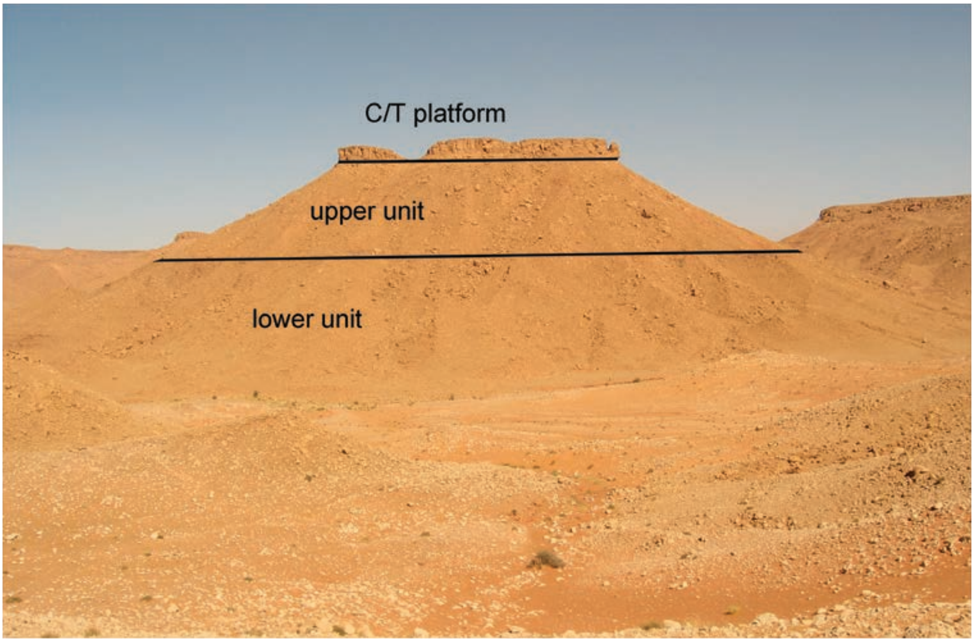

Figure 7. Outcrops of the "Continental intercalaire infracenomanien" in the Kem Kem (near Gara Sbaa). Lines separate the Cenomanian-Turonian platform that caps the "Continental intercalaire" from the upper and lower units.

Late Cenomanian-Turonian carbonate platform. Choubert (1948) recognized the Late Cenomanian-Turonian platform ("Calcaires cénomano-turoniens") as the uppermost unit of his "trilogie mésocrétacée". The carbonate platform was first described in detail by Ferrandini et al. (1985: 561, fig. 2) on the basis of a stratotype section at Akrabou (cited as "Akerboûss") approximately 45 km north of Erfoud. Ferrandini et al. (1985) and Ferrandini (1988) divided the platform into four successive subunits, the lower three within the Late Cenomanian and the last in the Turonian, with ages based on considerable biostratigraphic evidence. The subunits, which represent successive transgressive facies (coastal, reef, interior platform, and benthic platform), were not formally designated as members.

Additional sections were logged across the Akrabou Formation (at Ziz near Akrabou and at Tadighoust 50 km to the west) by Ettachfini and Andreu (2004), who also identified four subunits. Only the lower two were placed within the Late Cenomanian; the upper two units were regarded as Turonian in age. A little farther west in the High Atlas Mountains, Ettachfini et al. (2005) divide the Cenomanian-Turonian platform into five subunits, describing it as a new formation (Ben Cherrou Formation). Only the lowest subunit is regarded as Late Cenomanian in age, whereas the upper four are assigned to the Turonian. To the east across the Algerian border in the Béchar region, Benyoucef et al. (2012: fig. 2) divide the platform into four subunits dated by ammonites and other nonvertebrate fossils, the lower three of which were regarded as Late Cenomanian in age and only the uppermost Early Turonian.

Correlation between sections of the Cenomanian-Turonian platform in central Morocco is challenging, because of lateral variation in the character and age of platform subunits. Transgressive conditions, nonetheless, characterize all of these sections, which grade upward from shallow-water coastal facies to a more massive, deeper-water (subtidal) carbonate platform (Ferrandini et al. 1985). The platform complex also markedly thickens to the north of what Essafraoui et al. (2015) identify as the "Kem Kem embayment", on which the fluvio-deltaic Kem Kem beds were deposited. The platform is typically ~ 30 m thick over the Kem Kem beds. It rapidly thickens north of Aoufous to 90 m at Tazouguerte, which is located only 50 km northeast of Aoufous. Far to the north in the Anoual Syncline of the High Atlas, the Cenomanian-Turonian platform is often thinner, measuring ~ 20 m in thickness.

Kem Kem beds and embayment. The Kem Kem beds are exposed along the generally western-facing escarpment of the Guir and Kem Kem Hamadas. Capped by the Late Cenomanian-Turonian platform, the escarpment extends north-south approximately 200 km near the Morocco-Algeria border (Figs 1A, C, 8–11). The northern boundary of the outcrop is located approximately 30 km south of Errachidia (near Zrigat). It extends southeasterly toward Taouz, following the western edge of the Guir Hamada. From there it extends southwest along the western edge of the Kem Kem Hamada, diving under the low outcrop of the platform ca. 30 km north of Mhamid.

The Kem Kem beds rest unconformably on fossiliferous marine Paleozoic rocks of Silurian, Devonian, and Cambrian age, a contact exposed only in few areas (Figs 12, 13A). The escarpment, generated by Cenozoic erosion (Schmidt 1992), varies in topographic height from ca. 50 to 200 m (Figs 10–12). As much as 90% of the face of the escarpment is covered by limestone talus from the capping Cenomanian-Turonian platform. Available Kem Kem outcrop, therefore, is considerably less than the relatively continuous band shown on regional geological maps. Exposures most often are isolated patches or on the flanks of ravines. Broader areas of outcrop of the Kem Kem beds occur only in regions where the escarpment is prominent, such as near Zrigat, Gara Sbaa and Iferda N'Ahouar.

The Kem Kem beds comprise two units representing predominantly deltaic deposition on a northeast-southwest trending ramp recently termed the "Kem Kem embayment" (Essafraoui et al. 2015: 140, fig. 2). The Kem Kem embayment is bounded to the west by the Anti-Atlas and to the east by the Bechar promontory (Essafraoui et al. 2015). The "Taouz Basin" (Cavin et al. 2010) is another name applied to the structural depression that received sediments in the Kem Kem region. Deposition within this depression constitutes reactivation of the Paleozoic "Tafilalt Basin" in the same area (Wendt 1985, Bockwinkel et al. 2013).

Essafraoui et al. (2015: 162) used published sections (Cavin et al. 2010) and satellite images to conclude that the Kem Kem beds "thin to the south" and were laid down on a "South to North dipping (Tethyan-oriented), low-gradient ramp". The stratigraphic sections and paleocurrent data we logged during fieldwork in 1995, 2008, and 2013 confirm the main thrust of this interpretation. The exact thickness of the lower unit of the Kem Kem beds often cannot be established, because the contact with the Paleozoic is usually covered. The upper unit and the overlying limestone, nevertheless,

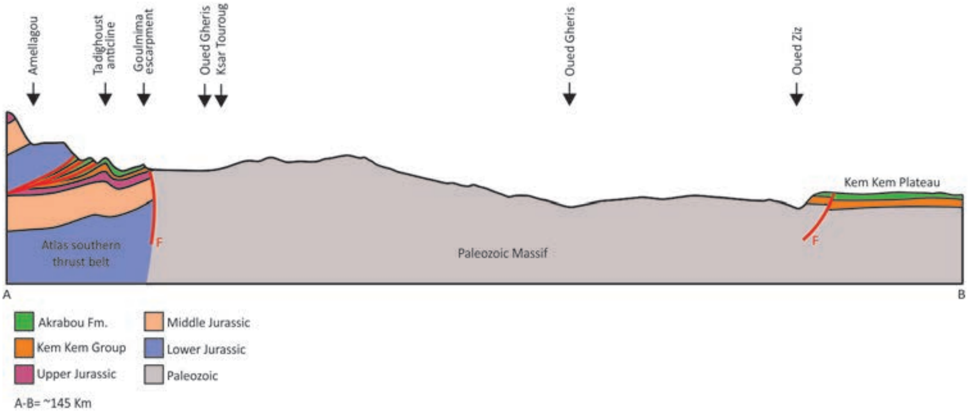

Figure 8. Schematic and simplified geological cross section from the southern Atlas mountains at Amellagou to the northern margin of the Hamada du Kem Kem at Ouzina spanning the Tafilalt Platform. Notice that the Kem Kem Group strata overlie a thick Mesozoic sequence in the north within the southern Atlas Mountain thrust belt but rest unconformably on the Paleozoic basement in the south. Likely the Tafialt Platform was an 'island' within the gigantic Kem Kem river system. The line of sections extends from **A** at Amellagou to **B** at Ouzina.

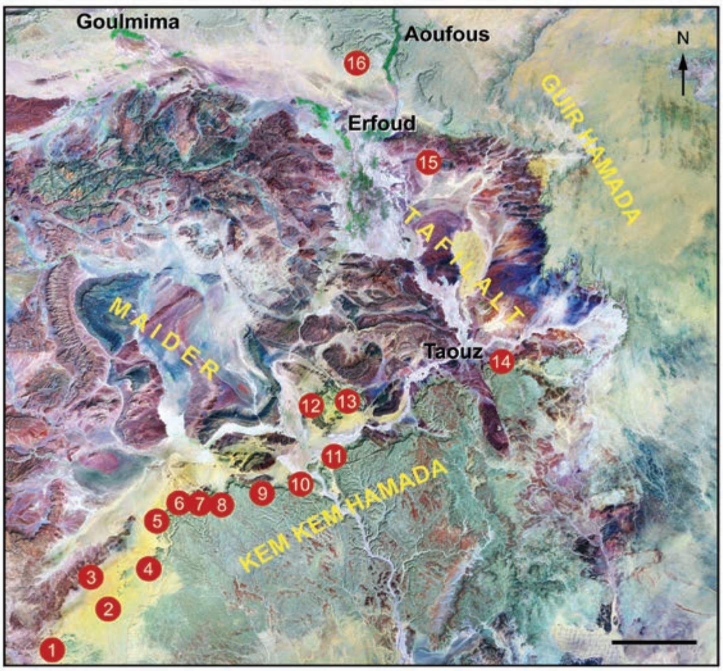

Figure 9. Landsat image of southeastern Morocco showing key localities, villages and cities, modified from Ibrahim et al. 2010. Scale bar equals 25 km. Abbreviations: **1** Tiknioune Bou Tazoult (just east of Zguilma), **2** Talidat, **3** Jbel Sdila, **4** Iferda Timenkhirt, **5** Gara Sbaa, **6** Oum Tkout, **7** Boumerade, **8** Moher, **9** locality just south of Zireg, **10** Daoura, **11** locality of the neotype of *Carcharodontosaurus saharicus* (Sereno et al. 1996), **12** Iferda N'Ahouar west, **13** Iferda N'Ahouar east, **14** Aferdou N'Chaft, **15** Hmar Lakhdad, **16** Douira Tagemout.

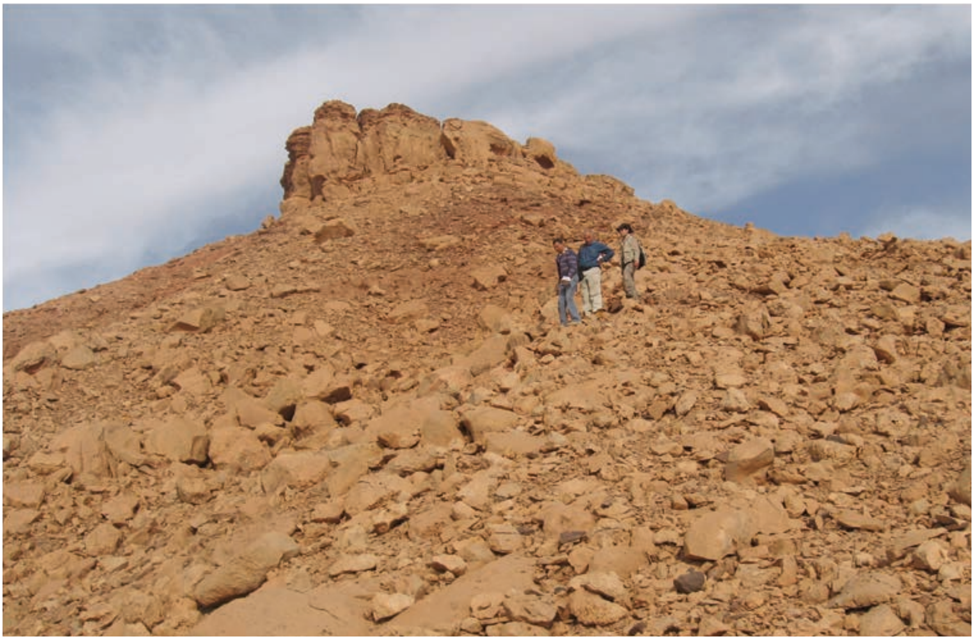

Figure 10. Jagged limestone talus from the overlying Cenomanian-Turonian platform at Gara Sbaa obscures the majority of the outcrop of the Kem Kem Group.

thin to the south toward Hassi Zguilma and to the east toward Taouz. The Kem Kem beds, thus, do appear to have been deposited on a low-gradient ramp opening to the north, with sediments derived from the Anti Atlas Massif to the west and lowland regions to the south and east in southern Algeria (Busson 1970).

Northeastern Kem Kem equivalents. Continental beds underlying the Cenomanian-Turonian platform are also exposed in the Béchar area of western Algeria northeast of the Kem Kem embayment. Lapparent (1960) reported on isolated bones and teeth of fossil vertebrates from these beds that are similar to those of the Kem Kem fauna. More recently, Benyoucef et al. (2012, 2014, 2015) documented the presence under the carbonate platform of coastal sabkha deposits in the Guir Basin of western Algeria. These deposits of Cenomanian age, although much thinner (~ 10 m) than the Kem Kem beds, have yielded a fauna broadly comparable to that reported here from the Kem Kem beds (Benyoucef et al. 2015). These data led Essafraoui et al. (2015) to conclude that a north-dipping, low-gradient ramp also existed during the Cenomanian on the Algerian side of the western Saharan craton.

Northern Kem Kem equivalents. The so called "Sillon Préafrican", or Pre-African Trough, trends southeast-northwest between the Anti-Atlas Massif to the south and the Central and Eastern High Atlas to the north. The trough follows a major rift system (South Atlas Fault or Front) that separates the northern Atlas and Rif regions from the African craton proper to the south (Fig. 13A). Possible equivalents of the Kem Kem beds are preserved on the shoulders, or margins, of the trough and taper in outcrop

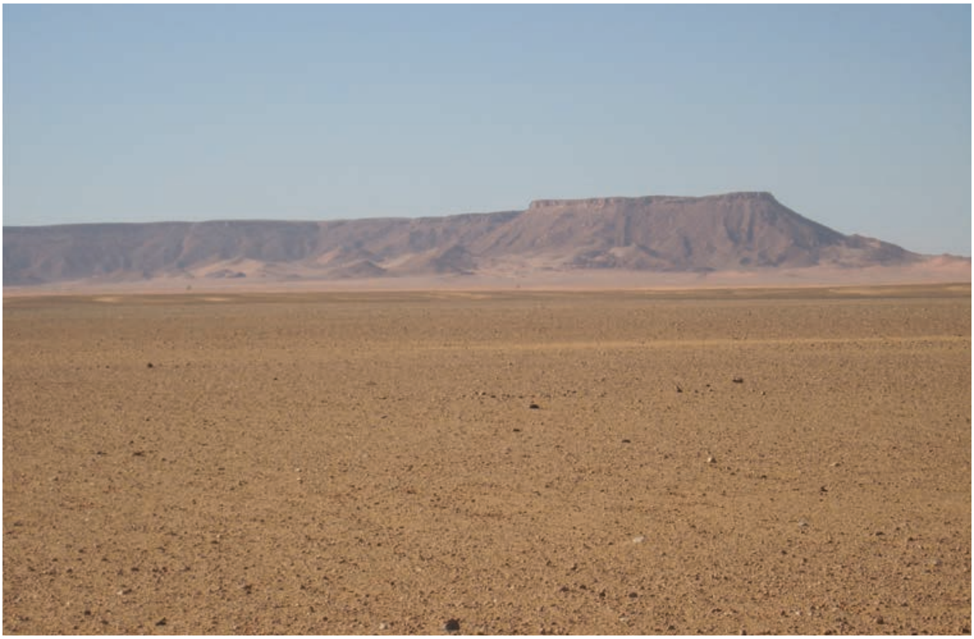

Figure 11. The escarpment at Gara Sbaa serves as a geographic landmark in the Kem Kem region.

width from Meski and Aoufous in the west to Goulmima and Tinghir in the east, where they pinch out.

Just a few kilometers north of Zrigat near Aoufous, there are two units of red beds underlying the limestone platform along the Meski-Aoufous Hamada. These beds lack the well-developed cross-bedding common in the lower unit of the Kem Kem beds and are considerably thinner, markedly gypsiferous, and barren of footprints or fossils (Fig. 14). Less than 10 km to the southeast near Zrigat, by contrast, both units of the Kem Kem beds are thick and well developed, as fully exposed and measured on the side of Al Gualb Mesa (Ibrahim et al. 2014b: supplementary Information). Not far from that mesa, a typical Kem Kem fauna was recovered, including a partial skeleton of the dinosaur *Spinosaurus aegyptiacus* (Ibrahim et al. 2014b).

Farther east near Goulmima, a generalized geological section shows the carbonate platform and underlying marl sediments that could correlate with the upper unit of the Kem Kem beds (Cavin et al. 2010: fig. 3). The published section, however, did not include significant sandy deposits down-section that might correspond with the lower unit of the Kem Kem beds. Confidence in correlation with units of the Kem Kem beds was further weakened by the absence of fossils.

One of us (DMM), however, discovered a better outcrop approximately 10 km to the north of Goulmima at a locality near Tadighoust known as Asfla. Both units comparable to those of the Kem Kem beds are exposed, although the facies differ from those typical of the Kem Kem beds. The lower unit has mudstones that pass upward into more typical channel sandstones, although commonly these are more cemented

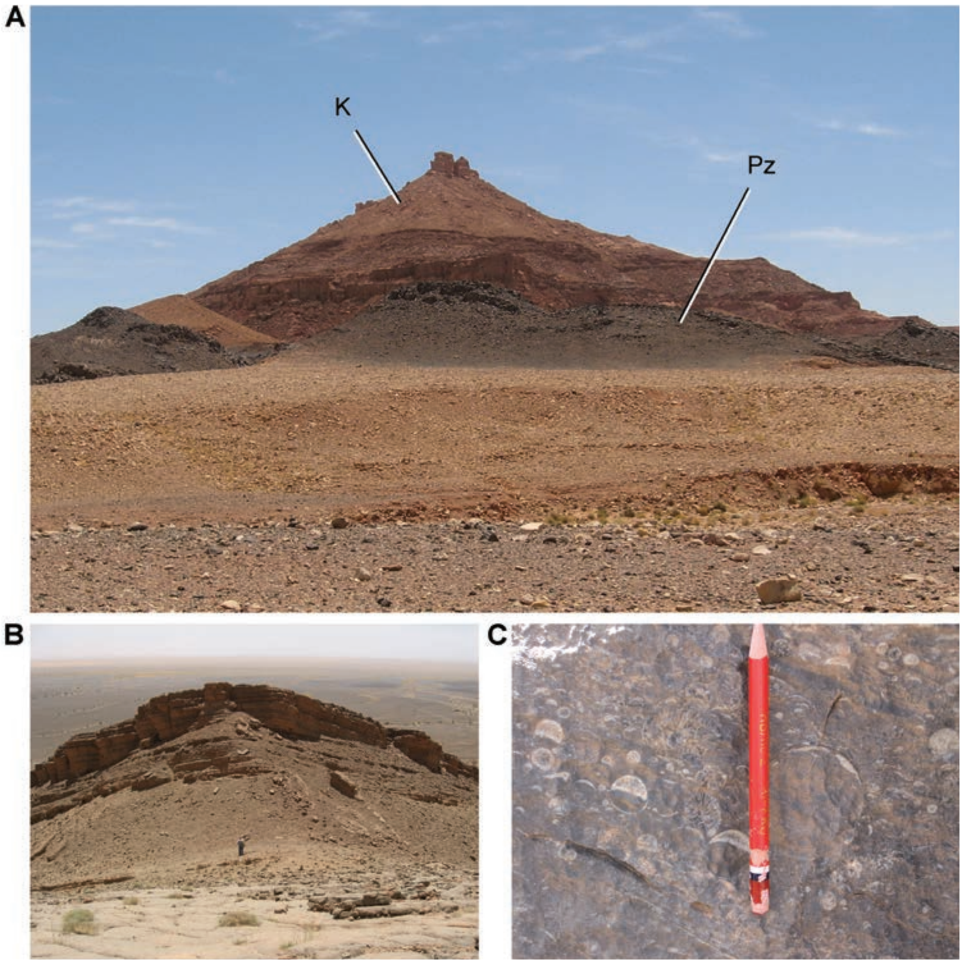

Figure 12. Paleozoic strata in the Kem Kem and Tafilalt regions. **A** Unconformity at Iferda N'Ahouar **B** Devonian outcrop at Hmar Lakhdad (Fig. 9: locality 15) **C** Geopetal infill structures in Devonian strata near Hmar Lakhdad. Abbreviations: **K** Cretaceous **Pz** Paleozoic.

than in the Kem Kem region. The upper unit is finer-grained, as in the Kem Kem beds, but significant gypsiferous evaporates are more common. Fossils appear to be absent from the upper unit but are present, if rare, in the cemented sandstones of the lower unit. Vertebrate teeth recovered pertain to the sawfish *Onchopristis*, the theropod cf. *Carcharodontosaurus*, and a large, subconical tooth pertaining to either a crocodylomorph or spinosaurid.

Farther to the west on the flanks of the High Atlas near Tinghir, a similarly more complete section is present and was visited by one of us (DBD). Two units are present under the carbonate platform that correspond well with the two units of the Kem Kem beds (Gauthier 1960, Essafraoui et al. 2015). The finer-grained upper unit, nevertheless, has more marine marls, marine sandstones, a beach sandstone, and gypsiferous

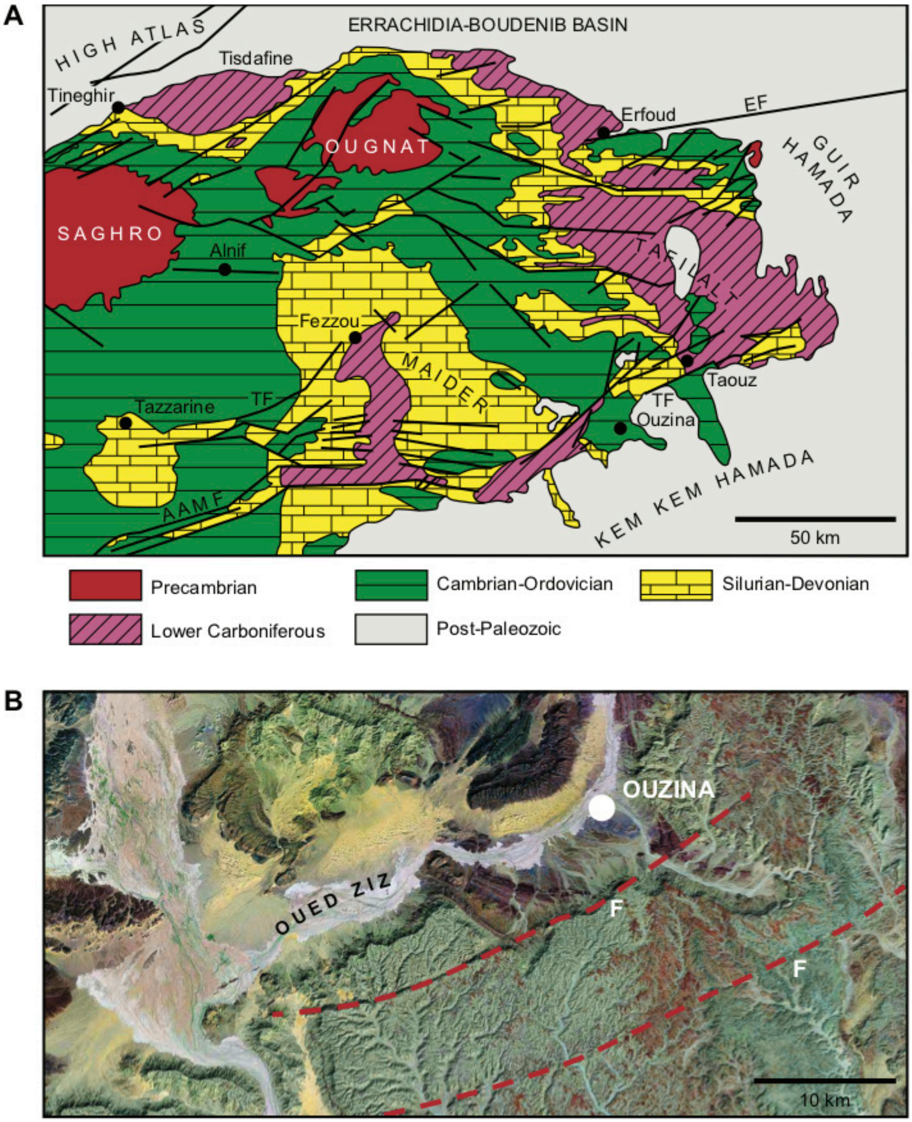

Figure 13. Tectonic setting of the Kem Kem region. **A** Major tectonic complexes in southeastern Morocco (modified from Baidder 2007) **B** Kem Kem Hamada fault lines (top unnamed, bottom Omjrane-Taouz fault, Google Imagerie 2010 Digital Globe). Abbreviations: **AAMF** Anti Atlas Major Fault **EF** Erfoud Fault **F** fault line **TF** Taouz Fault.

evaporate beds than typical in the corresponding Kem Kem unit (Essafraoui et al. 2015: fig. 16). The lower sandy unit shows ripple marks and occasional bioturbation as in the Kem Kem beds, but lacks the tiered cross-bedding common in the Kem Kem beds. Unlike the Kem Kem beds, vertebrate fossils are very rare and none have been figured (Gauthier 1960).

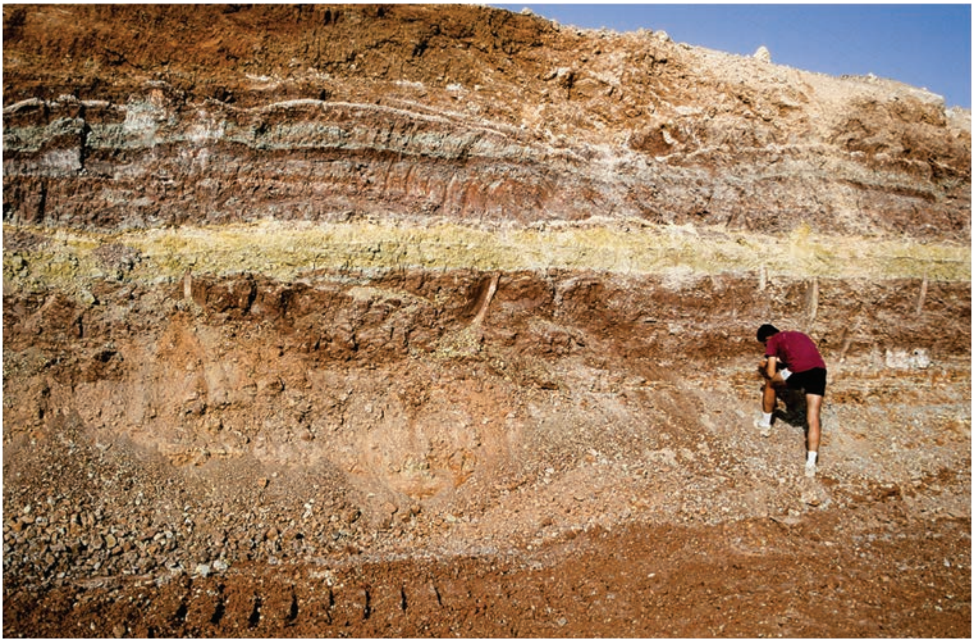

Figure 14. Continental red beds at Aoufous.

Choubert (1948), who described the Kem Kem beds, was aware of these infra-platform continental deposits but thought they were laid down in an Atlasic basin separate from the Kem Kem embayment. Using satellite images, Essafraoui et al. (2015) described Tinghir-like infra-platform deposits around the margins of the Anti Atlas Massif, which they regarded as the likely source of terrigenous input for these beds during the Cenomanian. Perhaps that accounts, in part, for the difference in lithology from comparable units in the Kem Kem embayment, which also appear to have had terrigenous input from the south. No matter their sediment source, we agree with the overall Cenomanian correlation outlined by Essafraoui et al. (2015), linking more coastal (Tinghir) and inland (Kem Kem) regions regarding the carbonate platform and two underlying units. Marked lithologic, ichnological and paleontological differences, however, suggest differing depositional conditions and favor the distinction of these units as lateral equivalents.

The two units in the Tinghir region have come to be called, in succession, the Ifezouane and Aoufous formations. Ettachfini and Andreu (2004: 278) and Cavin et al. (2010: 393) attributed authorship of the Ifezouane, Aoufous, and Akrabou formations to Dubar (1949; cited as "Dubar 1948"). Although it may have been Dubar's intention to replace Choubert's (1948) "trilogie mésocrétacée" with formations, no formation names, stratotypes or type localities are mentioned in this publication, which comprises notes accompanying a geological map of the High Atlas in the region of Midelt. These formation names appear to have been adopted in 1997 in notes accompanying a more recent geological map of the region around Tinejdad to the south of Goulmima (Ettachfini and Adreu 2004: 279). The next citation of these formation names appears to have been in a doctoral dissertation (Rahlmi 2000).

Given that notes to maps and dissertations are often difficult to obtain and of limited distribution, neither are considered "adequate publication" by the North American Stratigraphic Code (NACSN 2005). Here we suggest that stratotypes be recognized for the Ifezouane, Aoufous, and Akrabou formations based on the most detailed published sections, which are located at Tinghir (Essafraoui et al. 2015: fig. 16), Goulmima (Ettachfini and Andreu 2004: fig. 12), and Akrabou (Ferrandini et al. 1985: fig. 2), respectively.

Far north Kem Kem equivalents. Red bed units that may also correspond to the Kem Kem beds are situated some 50 km north in the Anoual Syncline in the eastern High Atlas, a basin known for yielding a diverse Lower Cretaceous (Berriasian) vertebrate assemblage including mammals and dinosaurs (Hahn and Hahn 2003, Knoll and Ruiz-Omeñaca 2009, Lasseron et al. 2019). The section extends upwards from these fossiliferous horizons to units immediately below a capping Cenomanian-Turonian platform. In 1995 fossils were collected from localities in the Lower Cretaceous beds and a section was logged across well-exposed Cretaceous outcrop immediately north of the well at Aïn Mellouk. No fossils were found in the two units immediately below the Cenomanian-Turonian platform, which in this region has a depth of ~ 20 m.

More recent geological and paleontological work in the Anoual region has resulted in the naming of several formations and the establishment of a better temporal framework based on recovered fossils (Haddoumi et al. 1998). The coarser-grained Dekkar 2 and finer-grained Dekkar 3 formations were named for the beds underlying the Cenomano-Turonian platform (Haddoumi et al. 2016: fig. 2). These formations bear a strong general resemblance to the lower and upper units of the Kem Kem beds, located some 500 km to the south. The Dekkar 2 Formation, for example, is composed of fine-grained marls interleaved with sand- and silt-stones, calcarenitic lenses, and gypsum evaporates. The overlying Dekkar 3 Formation, in contrast, is finer-grained and composed principally of marls interleaved with thin-bedded carbonate and gypsum evaporites (Haddoumi et al. 2016).

Haddoumi et al. (2016) tentatively regarded the Dekkar 2 Formation as Aptian in age, although the only fossils on which the date could have been established are charophytes, ostracods and bivalves near the base of the unit. The Dekkar 3 Formation was regarded as Cenomanian in age; it must predate the Late Cenomanian age of the base of the overlying limestone platform. These ages, therefore, broadly correspond with the age of the Kem Kem beds.

Dekkar formations 2 and 3 are preceded by Dekkar Formation 1, a coarse-grained unit lacking vertebrate remains and tentatively regarded as Barremian to Aptian in age. These Dekkar formations compose the Dekkar Group, bounded below by an unconformity and above by the Cenomanian-Turonian platform.

Hamadian Supergroup

Here we recognize the Hamadian Supergroup for a package of continental rocks across north Africa (Table 7), exemplified in central and eastern Morocco where they were

Table 7. Designation and correlation of Hamadian Supergroup and Kem Kem Group strata in central and eastern Morocco as proposed in this study. Hamadian Supergroup strata have been historically referred to as the "trilogie mésocrétacée" (Choubert 1948). The three strata composing the trilogy in the Kem Kem region include the Gara Sbaa, Douira and Akrabou formations. Stage and substage calibrations are based on ammonite zonation in western Europe (Voigt 2000).

Stage/Age	Substage	Lithostratigraphic units		Central and Eastern Morocco		
				South, (Kem Kem)	Central, (Tinghir)	North, (Anoual)
Turonian, 93.5–89.0 Ma	Upper<br>Middle<br>Lower	Hamadian Supergroup			Akrabou Formation	
Cenomanian, 99.0–93.5 Ma	Upper					
	Middle		Kem Kem Group	Douira Formation	Aoufous Formation	Deckar 3 Formation
	Lower			Gara Sbaa Formation	Ifezouane Formation	Deckar 2 Formation

first identified as the "trilogie mésocrétacée" (Choubert 1948). The name derives from the Arabic word "hamada", or rocky plateau, which refers to the resistant Upper Cenomanian-Turonian carbonate platform that forms barren plateaus across broad areas of northern Africa. Softer underlying sediments of sandstone, mudstone and marl are exposed on the edges of these resistant plateaus. The carbonate platform has yielded vertebrate fossils as well as a rich nonvertebrate fauna of Late Cenomanian and Early Turonian age. The softer underlying deltaic and marginal marine sediments are the source of most of what is known about terrestrial vertebrate life on Africa during the Early and Middle Cenomanian.

The Hamadian Supergroup includes strata laid down during a sustained, stepwise transgressive trend during the Cenomanian and Early Turonian. The rocks record a global eustatic second-order stratigraphic cycle that generated a tripartite rock record in continental areas, with two rock units underlying a carbonate platform. The underlying formations are deltaic or near shore, the first coarser-grained than the second, the base of which can often be recognized by sustained and significant mudstone deposition. Several pairs of formations of this general description are recognized below as regional manifestations of the Kem Kem sequence.

The "Hamadian series" or "Continental hammadien" (Lefranc and Guiraud 1990) are little used terms coined by Kilian (1931, 1937) for marine strata across northern Africa situated between the "Continental intercalaire" and "Continental terminal" (Tables 5, 6). They included the Late Cenomanian-Turonian carbonate platform and various younger strata of the early Paleogene (Danian). Unfortunately, the upper boundary of this package of units is ill-defined, much like the lower boundary of the "Continental intercalaire". The problematic upper boundary is limited only by the overlying, poorly dated, terrestrial rocks of the "Continental terminal".

We recommend, by contrast, the aptly named Hamadian Supergroup as a heuristic term that includes rocks related to a sustained transgressive stratigraphic cycle that are well exposed across northern Africa. The age of initial deposition is best estimated as Early Cenomanian in the Kem Kem region (see below), and the upper boundary of

deposition of the carbonate platform is well established as the end of the Early Turonian (Ferrandini et al. 1985, Ettachfini and Andreu 2004, Ettachfini et al. 2005). In this report, we do not extend correlations of the Hamadian Supergroup to northern African formations farther afield (i.e., outside Morocco). We anticipate future studies will extend correlations across northern Africa, anchored by the well-exposed Late Cenomanian-Turonian cliff-forming carbonate platform.

Kem Kem Group

Here we recognize the Kem Kem Group for rocks in central and eastern Morocco that comprise the first two non-marine units of the Hamadian Supergroup (Choubert's "trilogie mésocrétacée", Table 7). As discussed below, we cannot differentiate the Kem Kem Group strata in the Kem Kem region on the basis of their rich vertebrate remains. For this reason alone, it is heuristic to have an inclusive term for the pair of infra-platform non-marine units under the Late Cenomanian-Turonian platform. The Kem Kem Group appears to extend across much of central and eastern Morocco north and south of the Pre-African Trough. In this report, we do not extend Kem Kem Group correlations to strata west of Morocco, although we anticipate future studies making correlations to the thinner terrestrial beds in western Algeria (Benyoucef et al. 2015) and beds farther east across northern Africa.

Gara Sbaa Formation

The Gara Sbaa Formation, with stratotype at the locality Gara Sbaa (Fig. 15, Table 7), comprises the lower unit of Kem Kem Group rocks in the Kem Kem region of eastern Morocco. Northernmost exposures are located approximately 30 km south of Errachidia (near Zrigat). The formation is exposed as a relatively narrow band that extends southeasterly toward Taouz, following the western edge of the Guir Hamada, and then southwest along the northwestern edge of the Kem Kem Hamada, pinching out ca. 30 km north of Mhamid (Figs 15–17). Much of the outcrop is exposed in isolated patches or on the flanks of ravines, the remainder covered by limestone talus from the resistant Late Cenomanian-Turonian platform that holds the edge of the hamada. Broader areas of outcrop occur where the escarpment is prominent near Zrigat, Gara Sbaa, and Iferda N'Ahouar.

The Gara Sbaa Formation rests unconformably on fossiliferous marine Paleozoic rocks of Silurian, Devonian and Cambrian age (Figs 18, 19), a contact of some relief that is exposed only in few areas (Figs 12A, 13A, 19). The contact is exposed in sections at Taouz and Ouzina (Fig. 16), where the formation thickens from approximately 60 m to 100 m, respectively. It is thicker and well exposed at Gara Sbaa, where a ~ 135 m section is exposed (Fig. 17). Projecting from Paleozoic outcrops nearby, the basalmost 10–20 m of the formation is covered at Gara Sbaa. Maximum thickness of the forma-

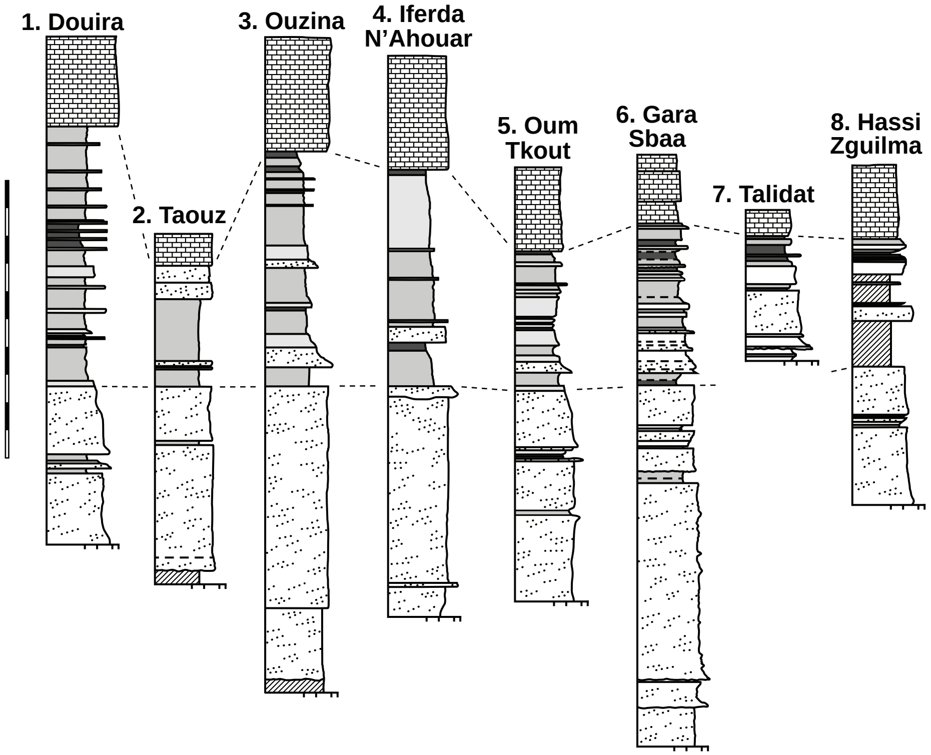

Figure 15. Generalized lithostratigraphic sections of the Kem Kem Group from Douira in the north to Hassi Zguilma in the south. Vertical scale bar equals 100 m.

tion, thus, is ~ 150 m in the region of the stratotyope Gara Sbaa, thinning to ~ 60 m to the northeast at Taouz and ~ 50 m to the south at Hassi Zguilma (Figs 15–17). The basal contact with bluish Ordovician siltstones is clearly exposed at Ouzina, which is here designated as the boundary stratotype for the base of the formation. The formation, often terminating in a cemented sandstone, is conformably overlain by a substantial red mudstone of the Douira Formation (Figs 20, 21). This contact is well exposed at Gara Sbaa and in other sections.

Lithology. The Gara Sbaa Formation is composed predominantly of red-colored, fine- and medium-grained sandstone beds. The basal one-quarter of the formation is characterized by poorly cemented coarse sandstones with minor conglomerate beds. The basal bed at Tikniouine Bou Tazoult consists of a relatively thin (< 1 m thick) conglomerate with sub-rounded pebbles and cobbles derived from the underlying Paleozoic clastics and, rarely, Paleozoic volcanics. A red-colored medium sandstone forms the basal bed in some areas (e.g., Aferdou N'Chaft). The remainder of the bottom one-quarter of the formation is predominantly medium-to-fine, poorly sorted sandstone with rare siltstone and mudstone beds. The sandstone has interstitial mud and some mafic and feldspar clasts. The poorly sorted sandstone is reddish on both weathered and freshly broken surfaces. Sandstone beds are generally thickly bedded (30–100 cm)

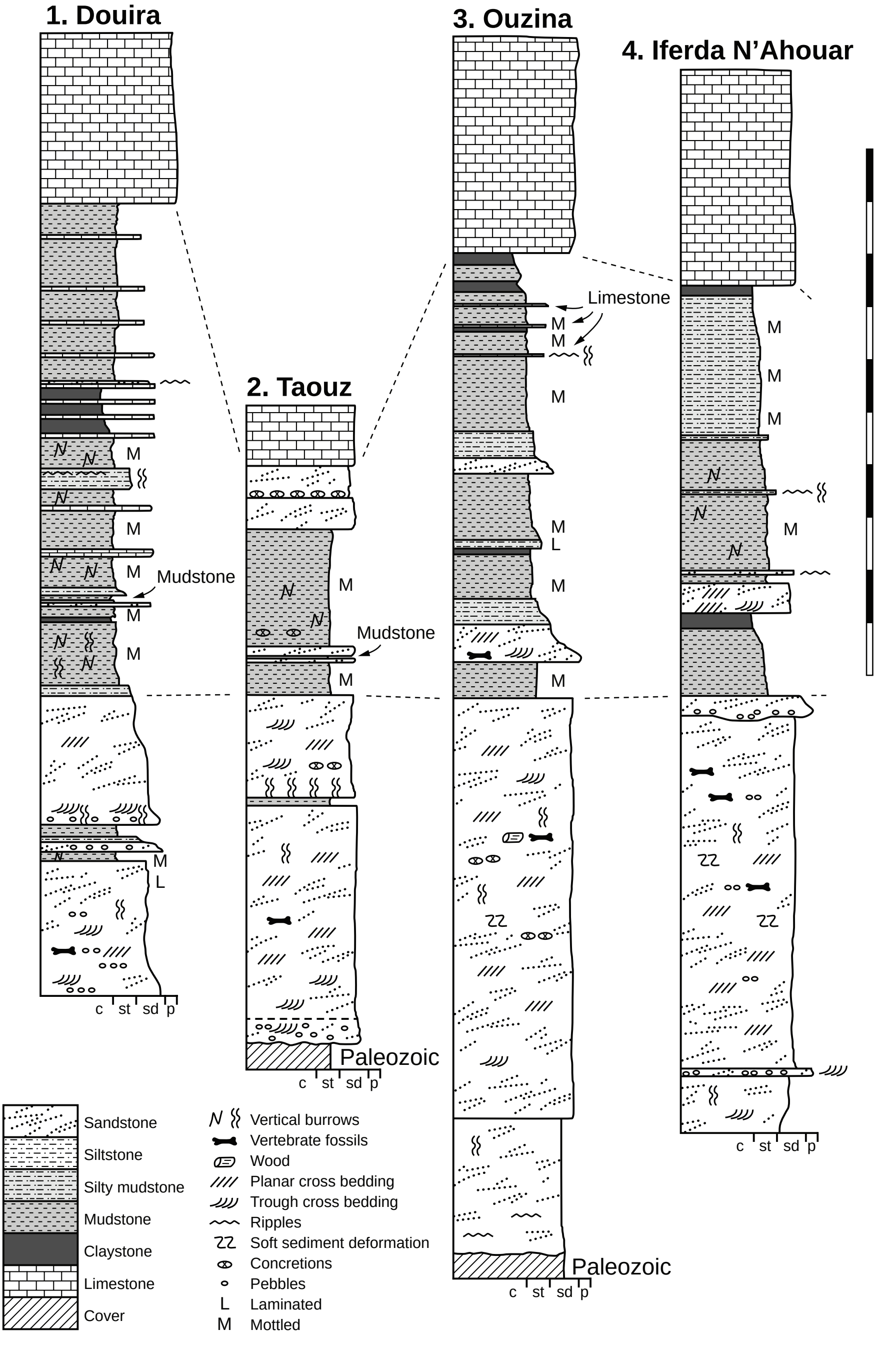

Figure 16. Northern lithostratigraphic sections of the Kem Kem Group. Vertical scale bar equals 100 m. Abbreviations: **c** clay **p** pebble **sd** sand **st** silt.

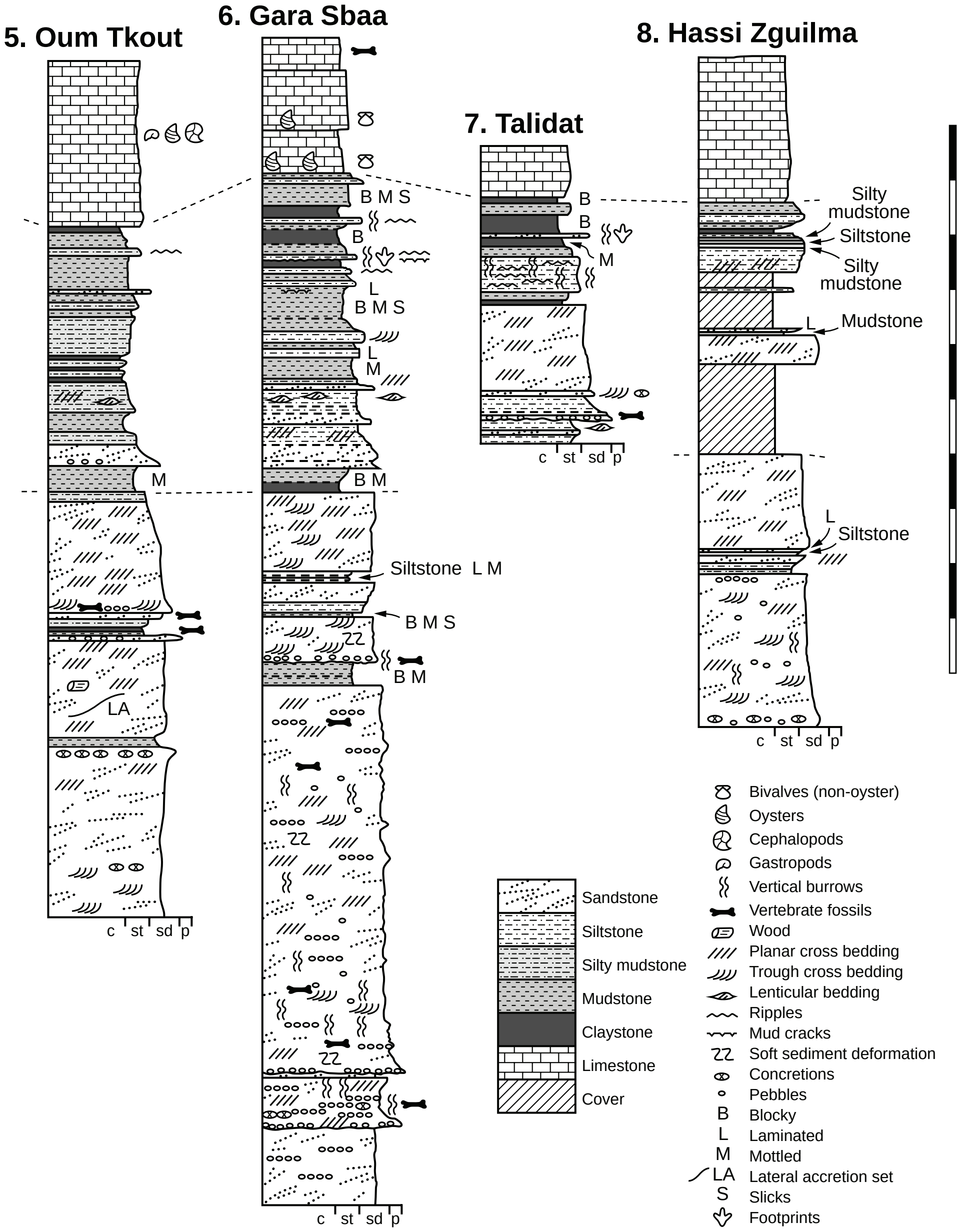

Figure 17. Southern lithostratigraphic sections of the Kem Kem Group. Vertical scale bar equals 100 m. Abbreviations: **c** clay **p** pebble **sd** sand **st** silt.

with occasional erosional channel scours up to 1 m deep. Limited in exposure, the lower one-quarter of the formation did not yield any body or trace fossils.

The remainder of the formation is composed of coarse-to-fine sandstone beds interspersed with pebble lags. Thin fine-grained beds and paleosols occur rarely. The

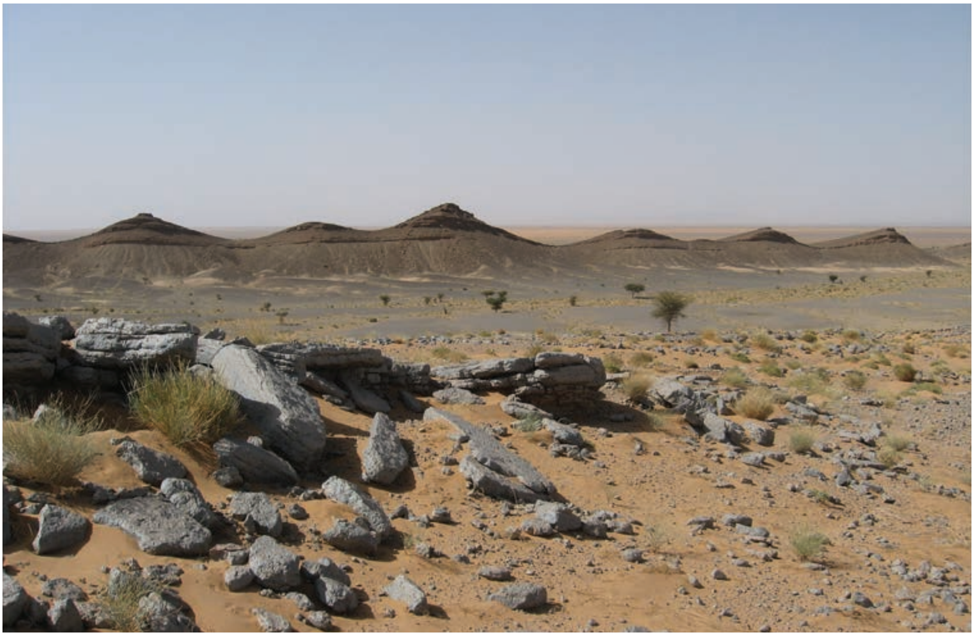

Figure 18. Outcrop of Paleozoic rocks underlying the Kem Kem Group near Hmar Lakhdad.

sandstones show greater maturity than in the basal beds and are composed almost exclusively of well-sorted, well-rounded quartz grains. The sandstone weathers buff-to orange-pink-red and is typically lighter in color, tan-to-yellow, on freshly broken surfaces.

Bed thickness ranges from thinner beds 0.3 m to 1.5 m thick to major units 2 to 80 m in thickness. Thicker beds, which occur toward the top of the formation, occasionally preserve lateral accretion surfaces with paleo-relief up to 8 m. Sandstone bodies typically accrete as tabular beds with a horizontal bottom. Tabular and trough cross-stratification is common with foresets dipping at 20–25° (Figs 22–25). Cross-beds frequently exhibit sorting, the coarser beds including coarse to very coarse sand, granules, and small pebbles. Desiccation cracks and ripple marks indicate intervals of very shallow water conditions (Fig. 25D, E).

The beds in the upper three-quarters of the formation often show cyclic deposition. A basal sandstone bed exhibits trough cross-stratification and sometimes thin conglomerates. This is followed by tabular cross-stratified beds that indicate channel infill. These beds are followed by sandstones rich in yellowish clay and bone fragments that may indicate incipient soil development. These cycles comprise the infilling of channels.

Unusual sedimentary structures include disrupted bedding, spherical concretions, and slab-forming carbonate and iron cemented beds. Disrupted bedding (escape structures, sand volcanoes) and overturned cross-bedding (Fig. 25A-C) in some places suggests thixotropy (liquefaction). Dewatering and slumping on gentle slopes may have been triggered by earthquakes. Pinkish spherical concretions, with diameters of 35–150 mm, are common and sometimes are present in cemented aggregations on

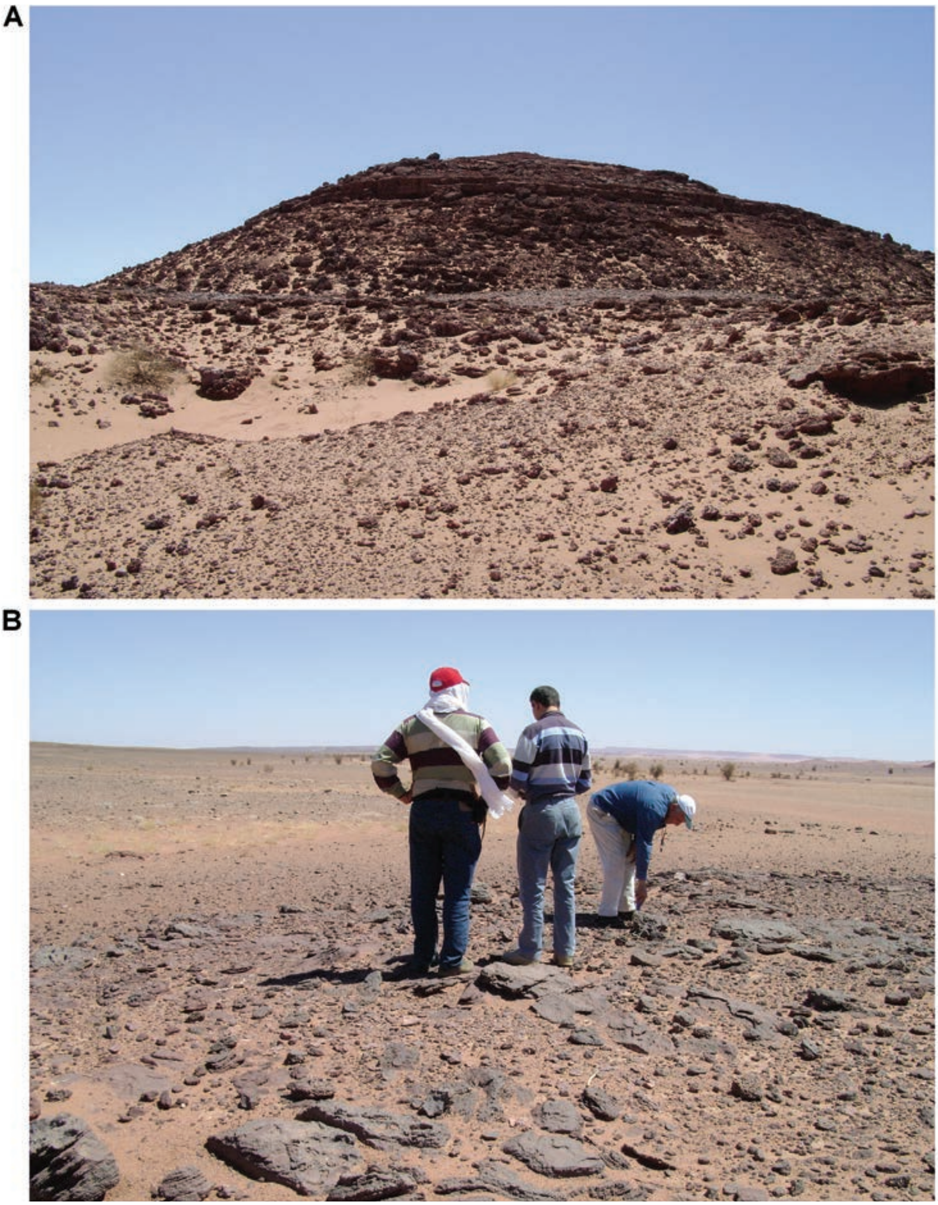

Figure 19. Paleozoic rocks underlying the Kem Kem Group. **A** Jbel Sdila locality. **B** Close-up of Paleozoic basement.

bedding surfaces (Figs 26, 27). These iron-rich concretions, sometimes called 'kerkoubs' (Cailleux and Soleilhavoup 1976), often, but not always, follow bedding planes. Carbonate- and iron-cemented beds that are dark brown or even black in color occur in places at the top of the formation as a resistant ledge.

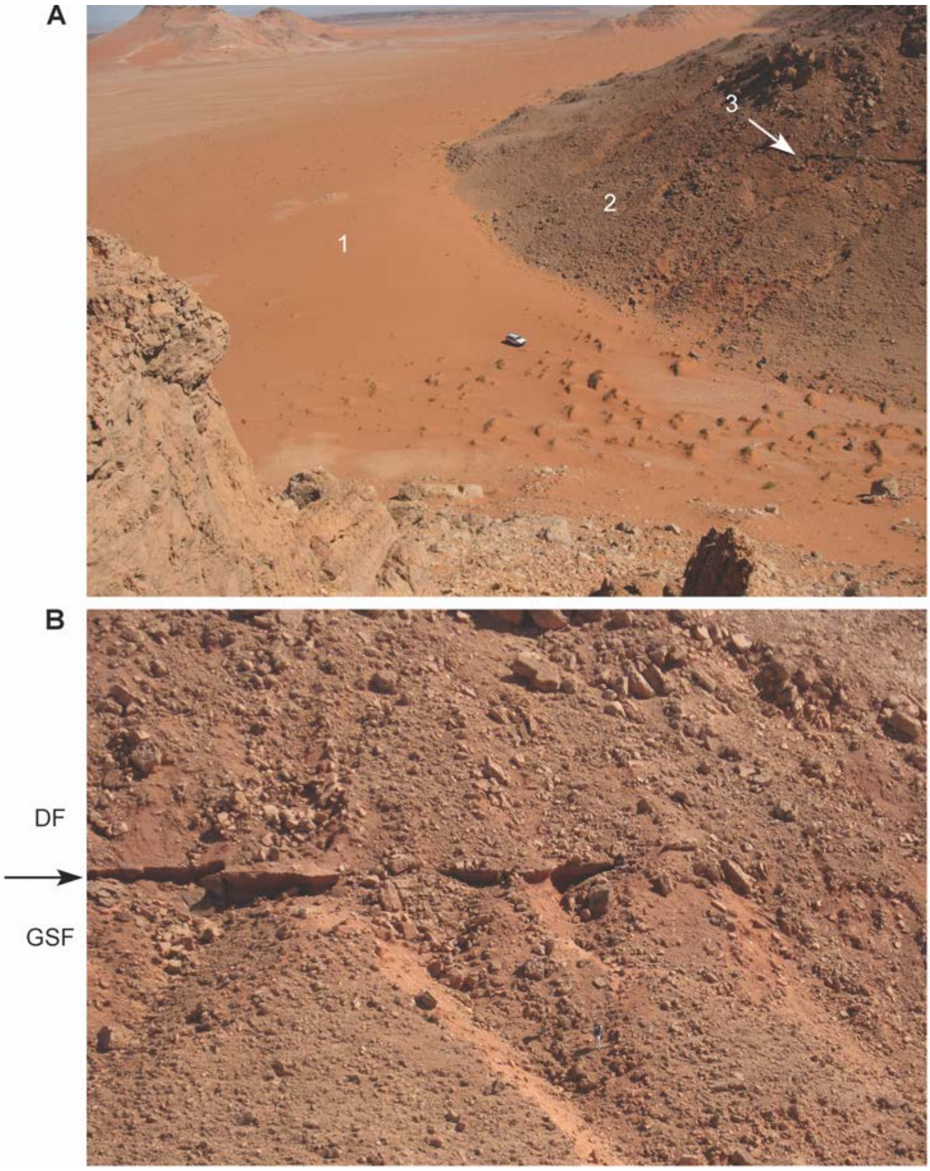

Figure 20. The Kem Kem Group at Iferda Timenkherin. **A** Overview **B** Close-up view of the boundary between members at Gara Sbaa: **1** Quaternary aeolian sand **2** Lower member **3** Boundary between the upper and lower members. Abbreviations: **DF** Douira Formation **GSF** Gara Sbaa Formation.

The sedimentary structures described above and the lack of substantial conglomerates suggest that the Gara Sbaa Formation records large-scale transport of detrital material typical of a deltaic river system. Cross-bed orientation in the lower quarter of the formation strongly favors a northward flow direction parallel to the axis of the

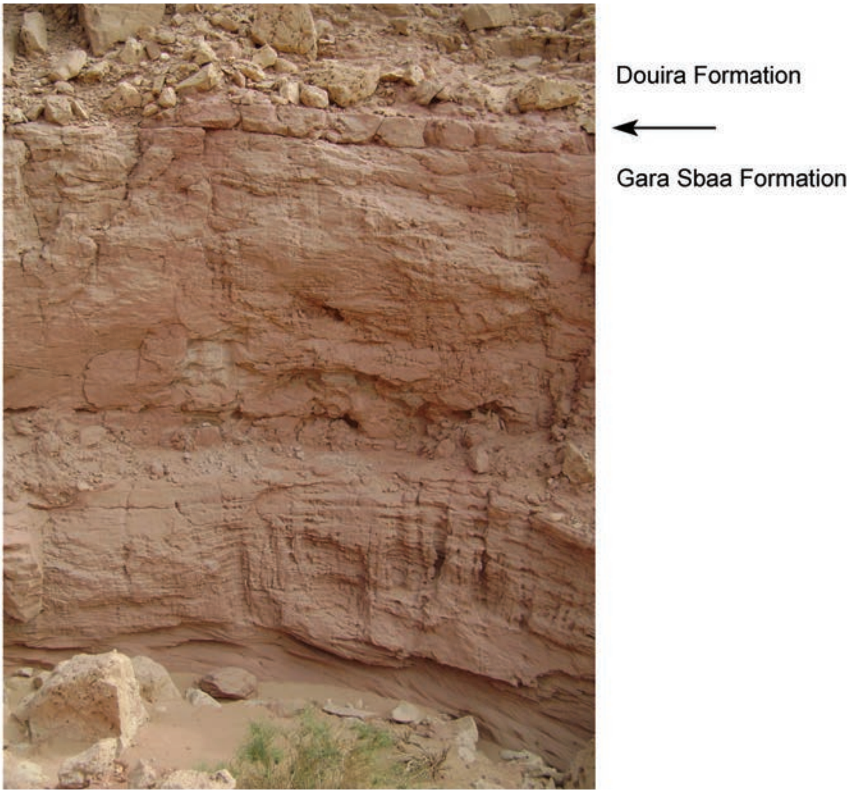

Figure 21. Limit (arrow) between the Gara Sbaa and Douira Formations.

Kem Kem embayment. The change in sedimentary style in the upper three-quarters of the formation suggest the infilling of an expansive alluvial basin centered on the low-gradient Kem Kem ramp.

Paleontology. Vertebrate body fossils most frequently occur as isolated elements (especially bone fragments, teeth, scales) in conglomeratic deposits characterized by rip-up clasts and pebbles. Rostral teeth of a large sawfish, *Onchopristis numidus*, are often the most common taxon among recovered teeth. Other common fossils include a wide range of aquatic and terrestrial species including polypterids, lepisosteids, amiids, bony fishes, dipnoans, turtles, crocodyliforms, and isolated teeth of the theropods *Spinosaurus* and *Carcharodontosaurus*. The teeth of terrestrial herbivores are conspicuously rare and only include sauropods. Articulated skeletons are extremely rare and only include the holotypic partial skeletons of *Rebbachisaurus garasbae* and *Deltadromeus agilis* (Sereno et al. 1996, Wilson and Allain 2015). Nonvertebrate remains are rare and include unionoid bivalves recovered at Aferdou N'Chaft and Zrigat. Trace fossils include root traces within rare mudstones, straight, vertical burrows averaging 2 cm in diameter and tens of centimeters long within cross-bedded sandstones, and borings in dinosaur bone fragments (Ibrahim et al. 2014a). The borings are curved, unbranched, tubular traces situated most commonly in the outer portion of weathered bone. These may represent the insect ichnotaxon *Cubiculum ornatus* or another arthropod feeding

A

B

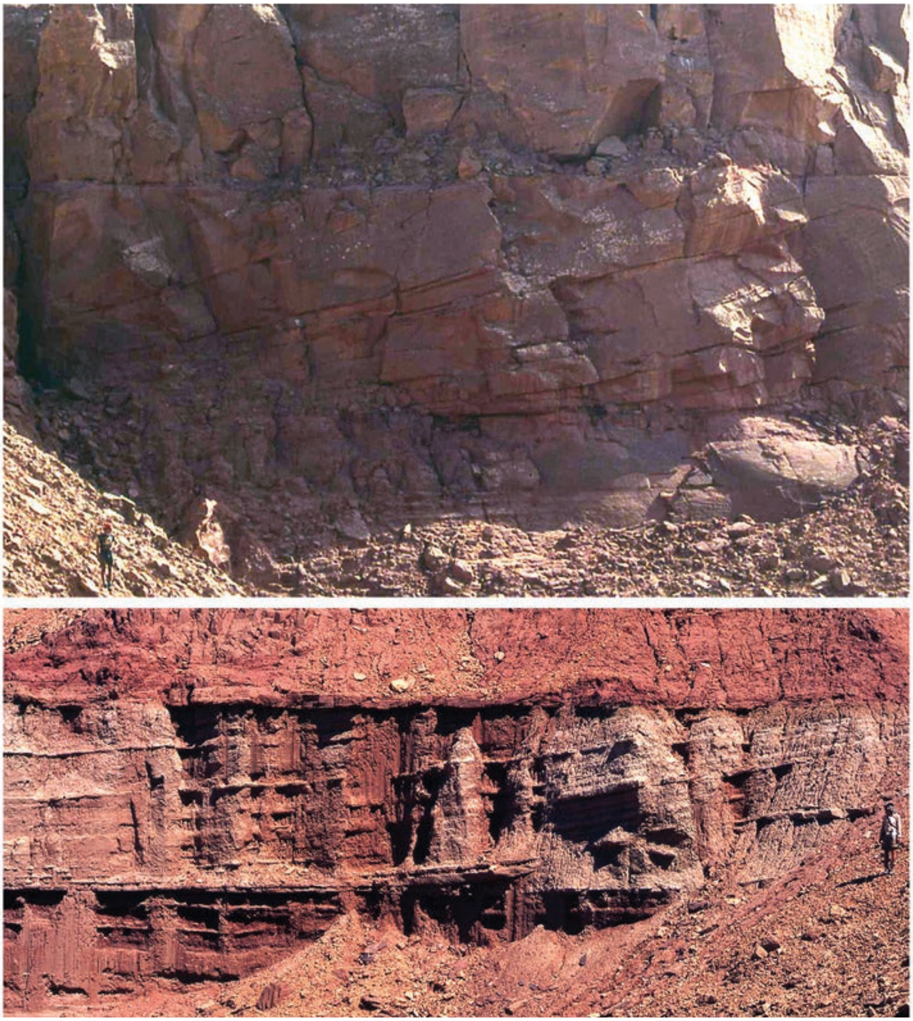

Figure 22. Large-scale primary sedimentary structures. Large sedimentary structures occur occasionally in the upper parts of the Gara Sbaa Formation and the lower parts of the Douira Formation. In contrast to the primarily sandstone unit in (**A**), the lithology of (**B**) consists of inter-bedded sandstone and finer-grained beds. Human for scale in each equals 185 cm. The large-scale cross-bedding with good bottomsets and relatively low angle foresets is consistent with small Gilbert-type delta fronts. Alternatively, they could represent large point bar deposition with the differing lithologies of **B** as inclined heterolithic stratification.

on subaerially exposed bone (Ibrahim et al. 2014a). *Thalassinoides*-like burrows occur in a thin limestone in the Gara Sbaa Formation at Zrigat.

Douira Formation

The Douira Formation, with stratotype at the locality Douira (Fig. 16, Table 2), comprises the upper unit of Kem Kem Group rocks in the Kem Kem region of eastern Mo-

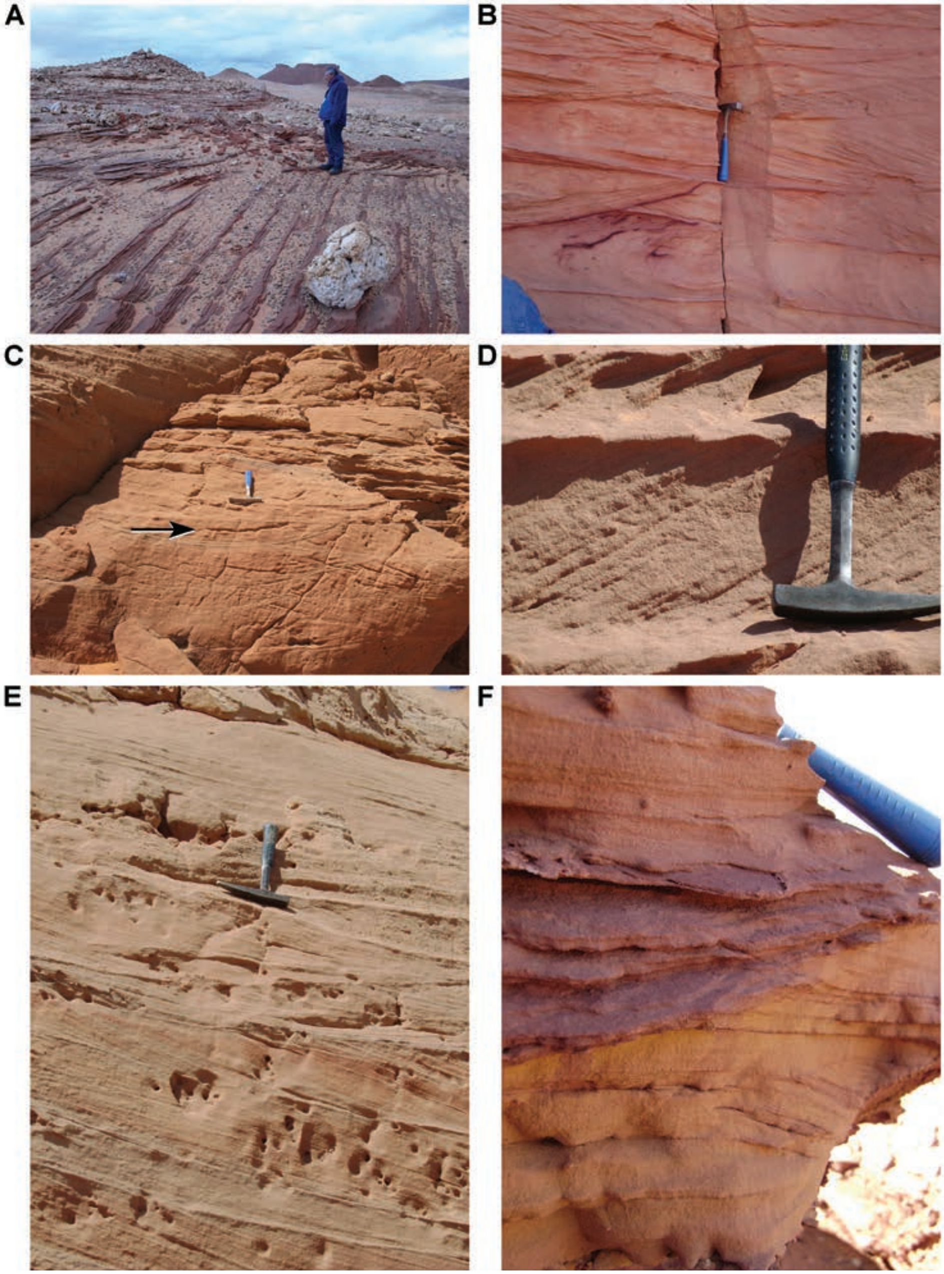

Figure 23. Primary sedimentary structures in the Gara Sbaa Formation. **A** Linear cross-bedding at Gara Sbaa **B** Trough cross-bedding at Aferdou N'Chaft **C** Shallow channel cross-bedding (arrow) at Gara Sbaa **D** Tabular cross-bedding from unidirectional flow at Gara Sbaa **E** Large-scale cross-bedding at Gara Sbaa **F** Trough cross-bedding at Aferdou N'Chaft.

A

B

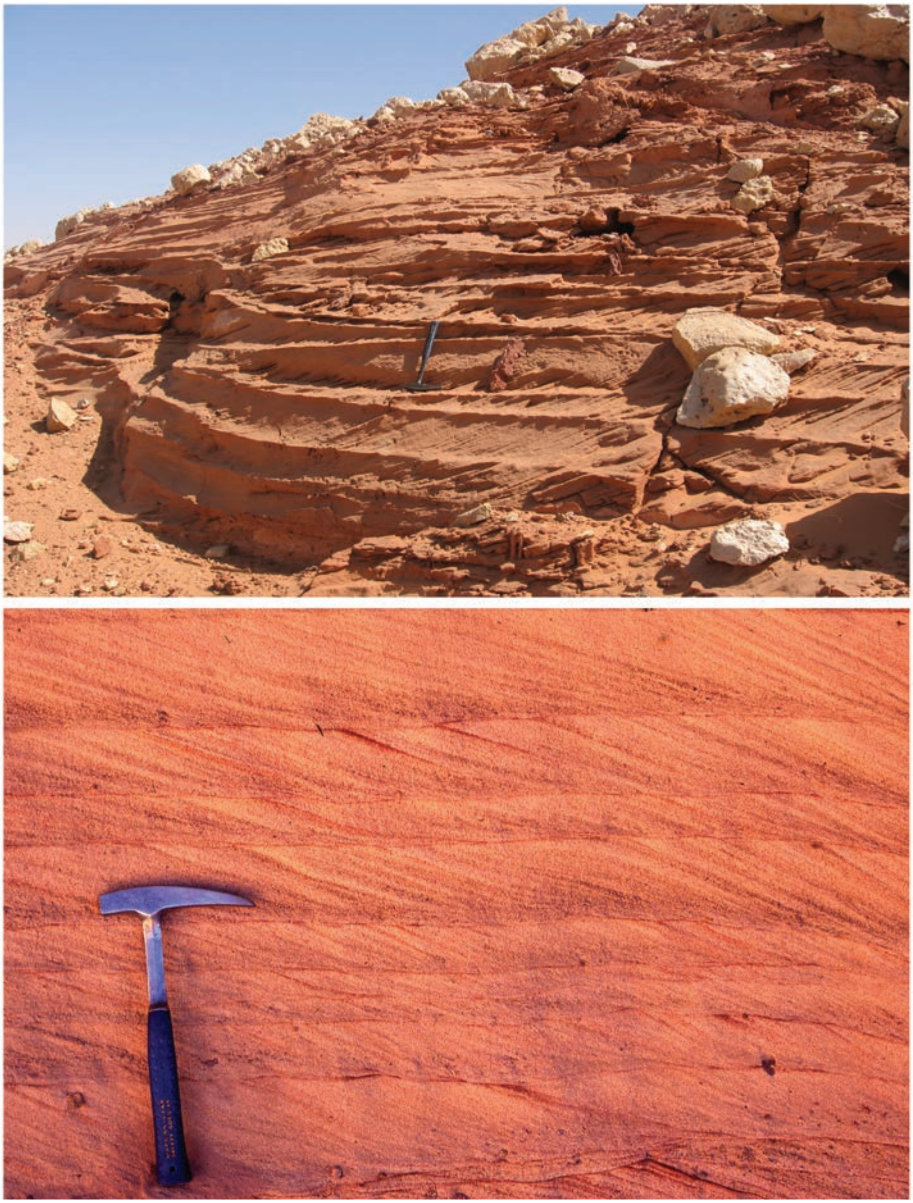

Figure 24. Tabular cross-bedding in the Gara Sbaa Formation. **A** Weathering of common tabular cross-beds **B** Close-up view of tabular cross-beds.

rocco. As with the underlying Gara Sbaa Formation, the northernmost outcrop is located approximately 30 km south of Errachidia (near Zrigat). The formation is exposed as a relatively narrow outcrop that extends southeasterly toward Taouz, following the western edge of the Guir Hamada, and then southwest along the western edge of the Kem Kem Hamada, pinching out ca. 30 km north of Mhamid. Much of the outcrop

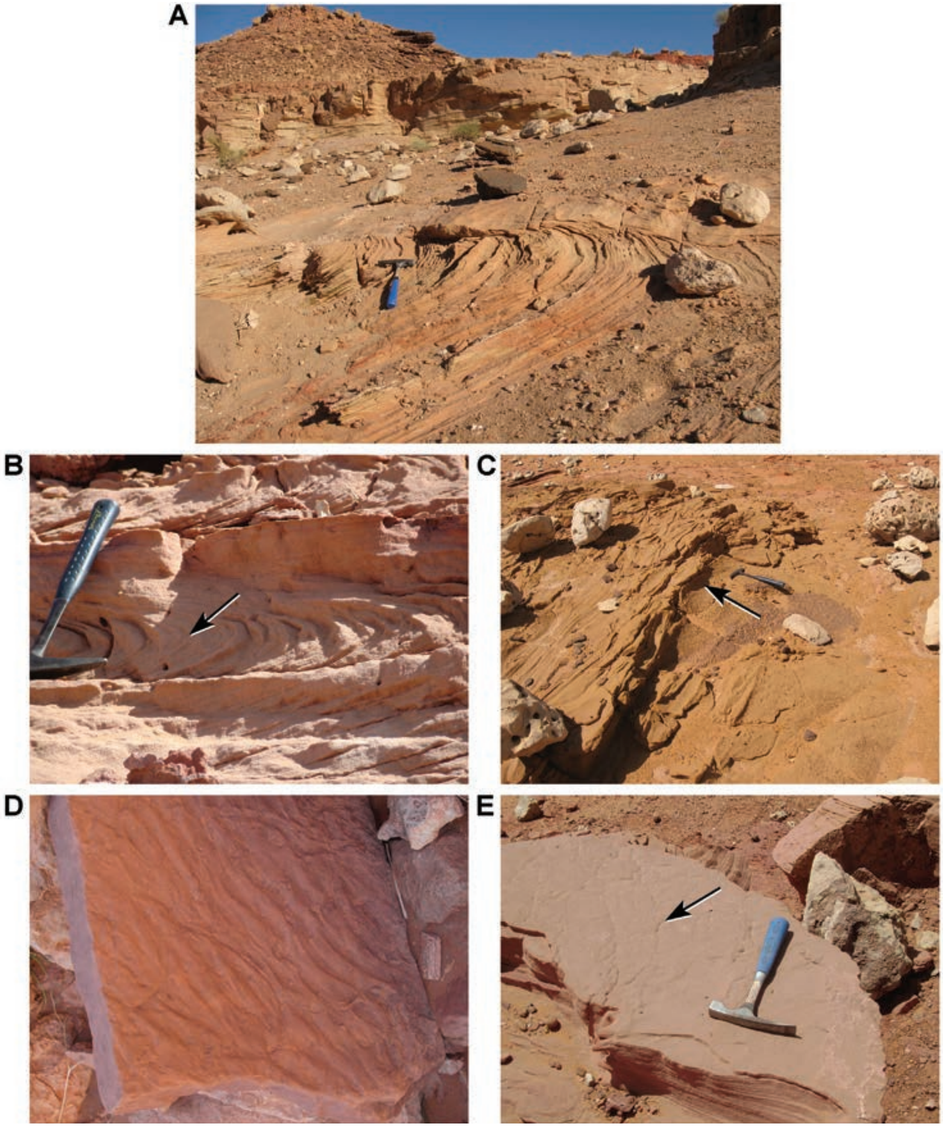

Figure 25. Sediment deformation and surface structures in the Gara Sbaa Formation at Gara Sbaa. **A** Large-scale overturned cross-bedding **B** Cross-section of overturned cross-bedding. **C** Surface view of overturned cross-bedding **D** Ripple marks **E** Desiccation cracks.

is exposed in isolated patches or on the flanks of ravines, the remainder covered by limestone talus from the overlying Late Cenomanian-Turonian platform that holds the edge of the Hamada (Figs 28, 29). Areas of outcrop are restricted and sometimes vertical, given the softness of the formation underlying the resistant carbonate escarpment. The Douira locality is well exposed and particularly rich in micro-vertebrate remains. Work in this section resulted in the discovery of a number of the shark teeth that established a Cenomanian age for Kem Kem Group strata (Sereno et al. 1996).

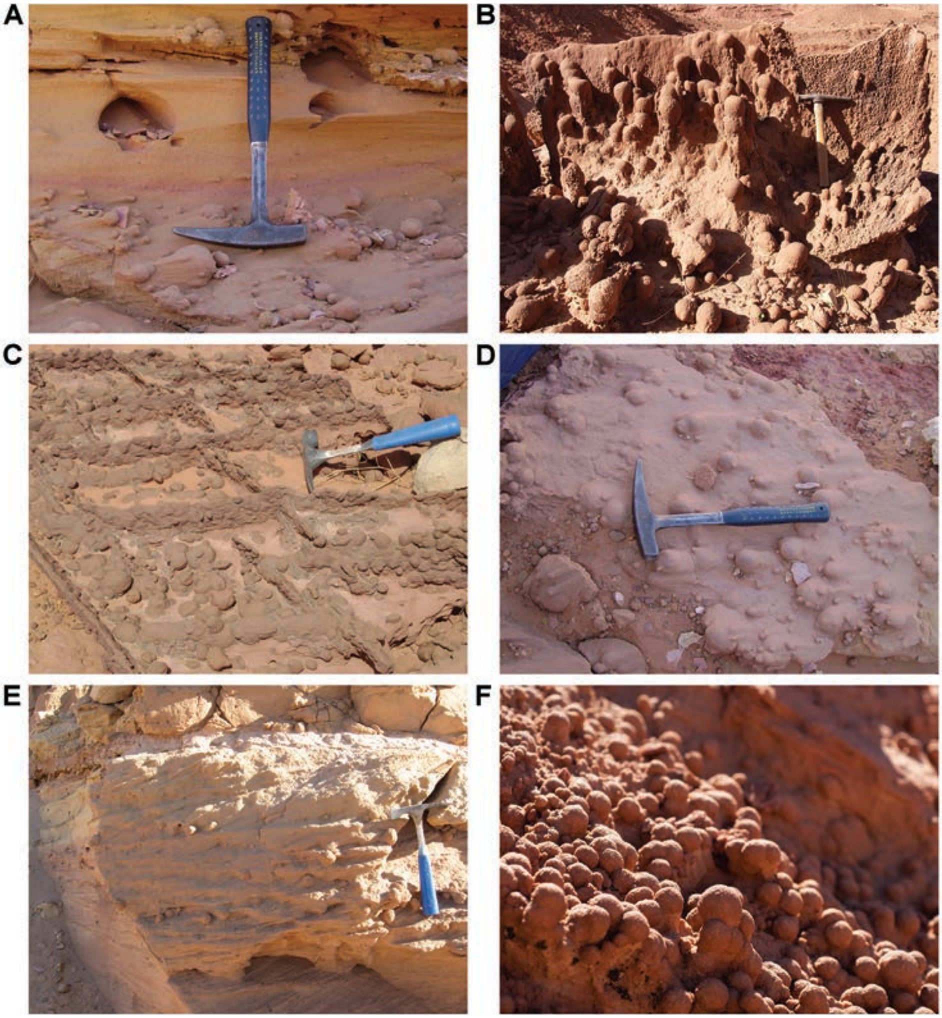

Figure 26. Enigmatic concretions ('kerkoubs') in the Gara Sbaa Formation. **A** In cross-stratified sandstones at Gara Sbaa **B** In flow direction near Boumerade **C** With linear sedimentary structures at Gara Sbaa **D** With great size variation at Gara Sbaa **E** In tabular cross-bedding at Gara Sbaa **F** In high density.

The formation is thickest at Douira, where a 124-m section is exposed. Maximum thickness of this formation is located more distal (north) than the thickest part of the underlying, progradational Gara Sbaa Formation. Like the Gara Sbaa Formation, it thins to the east to 52 m at Taouz and to the south to approximately 100 m at Oum Tkout and Gara Sbaa and to 46 m farther south at Hassi Zguilma (Figs 16, 17). The Douira Formation rests conformably on the Gara Sbaa Formation, a contact that shows very little relief. The basal bed of the Douira Formation is an easily recognizable reddish mudstone averaging ~ 1 m in thickness. This bed is always the first substantial mudstone in Kem Kem Group strata (Fig. 21). The uniform presence of this fine-

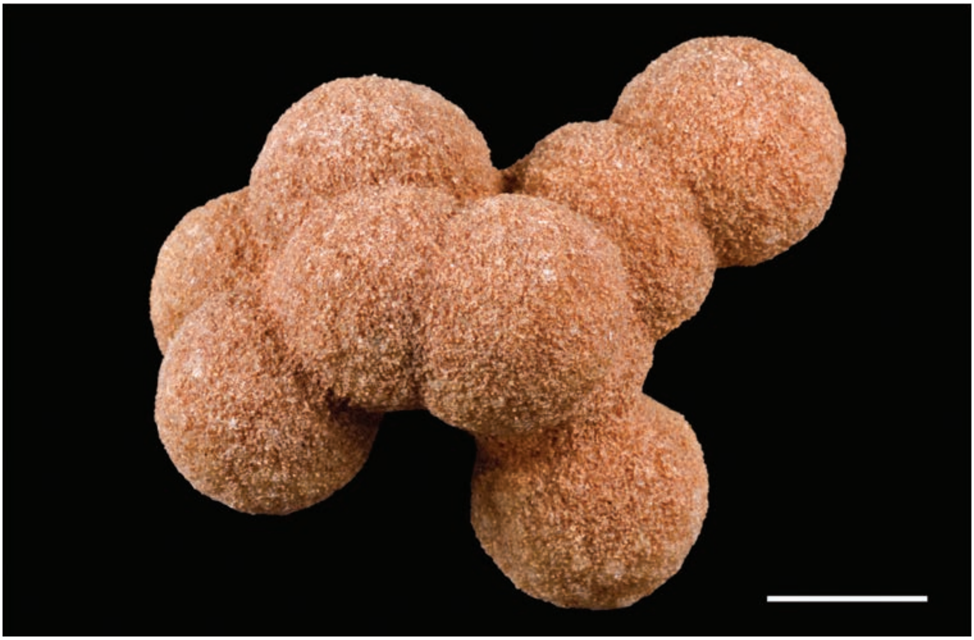

Figure 27. Close-up of 'kerkoubs' showing granular surface texture. Scale bar equals 1 cm.

grained bed over a broad geographic region suggests there was a transgressive pulse of some sort that altered the sedimentary regime for the region. The upper boundary of the Douira Formation, like the lower boundary, shows only minor relief, and is marked by a massive fossiliferous limestone, the basal unit of the overlying Akrabou Formation. The last sediments of the Douira Formation are typically green or gray claystone or mudstone. In northern sections of the Douira Formation, a few thin-bedded marl or limestone units occur some meters below the massive limestone of the Akrabou Formation. These are recorded in the section at Ouzina and become more substantial and spaced in the more distal (north) section at Douira, the first located some 75 m beneath the boundary carbonate (Fig. 16).

Lithology. The Douira Formation is finer-grained and has a greater variety of rock types than the Gara Sbaa Formation. It consists of fining-upwards, coarse-to-fine grained sandstones intercalated with siltstones, variegated mudstones, and occasional thin gypsiferous evaporites (Fig. 17). Like the Gara Sbaa Formation, the Douira Formation fines upward, and red-hued rocks predominate. Sandstones dominate the lower portion, whereas mudstone, claystone and ledge-forming siltstones and fine sandstones dominate the upper portion. Non-sandstone lithologies, which are rare in the Gara Sbaa Formation, comprise approximately two-thirds of the Douira Formation. Rock types include mudstones (64%), lesser amounts of sandstone (25%) and siltstone (10%), and other lithologies (1%) such as rare thin-bedded gastropod-rich limestones. In measured sections, the volume of silt and finer lithologies ranges from 50% at Gara Sbaa to 84% farther distally (north) on the delta at Iferda N'Ahouar.

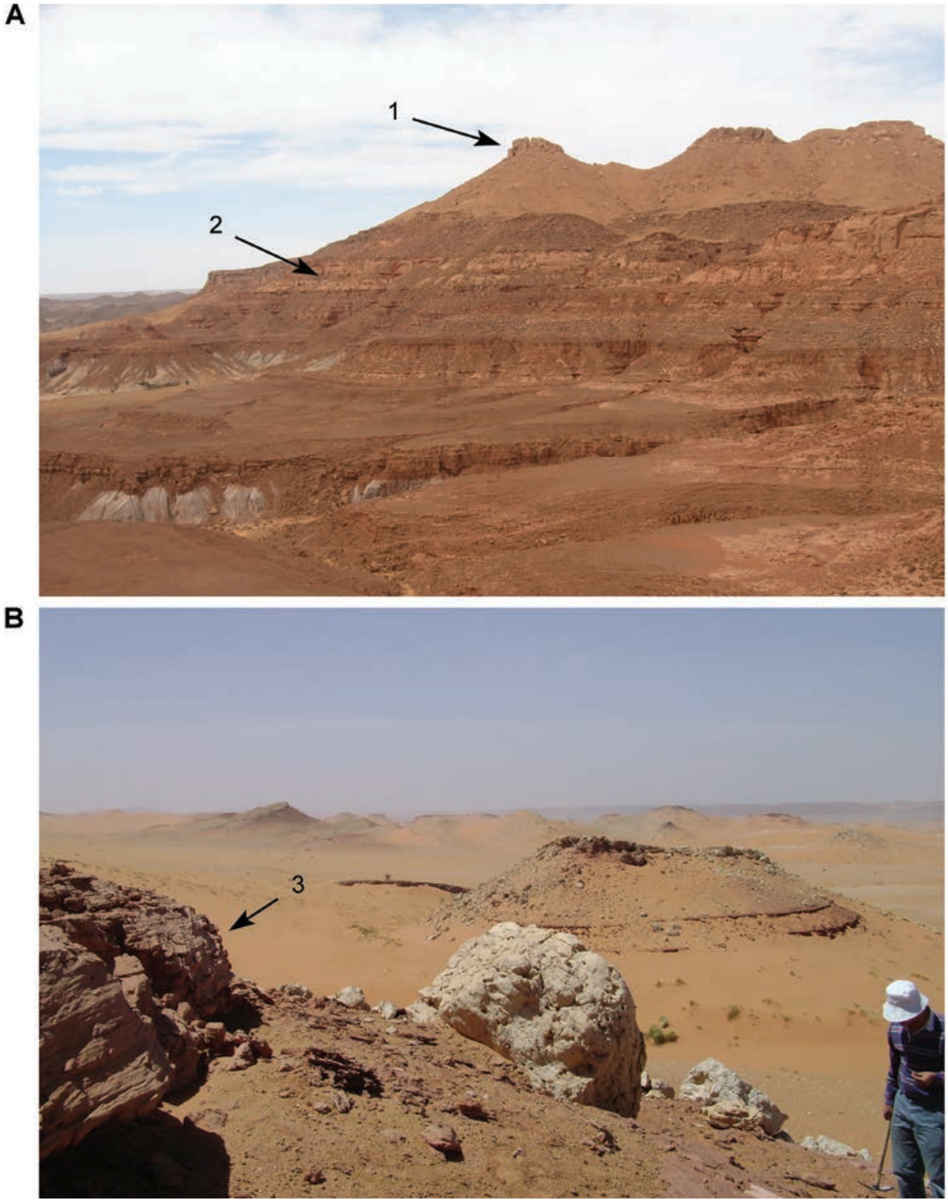

Figure 28. Exposures of the Douira Formation and underlying sandstones. **A** Outcrop at Iferda N'Ahouar **B** Outcrop at Tikniouine Bou Tazoult. Abbreviations: **1** Limestone platform **2** Douira Formation **3** Sandstone of the Gara Sbaa Formation.

Five sandstone facies occur within the Douira Formation. Some 60% of the sandstones are fining-up beds that begin with a coarse-grained sandstone that is poorly sorted with pebble-sized lithics. This basal bed contains cobble-to-pebble-sized clay balls, mud rip-up clasts, bone fragments and isolated teeth. The rest of the facies is

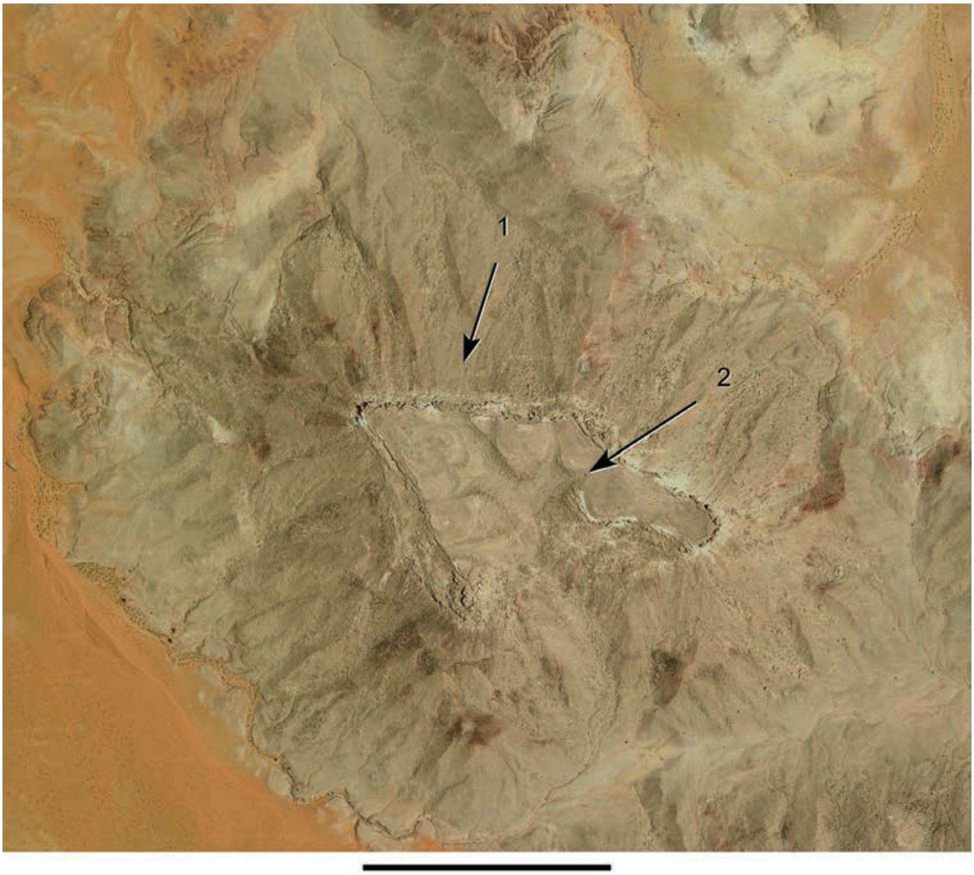

Figure 29. Satellite image at Gara Sbaa (Photo credit: Google Imagerie, 2010 Digital Globe). Scale bar equals 500 m. Abbreviations: **1** Kem Kem Group **2** Overlying limestone platform of the Akerbous Formation (Cenomanian-Turonian).

composed of fine sandstone or siltstone often characterized by trough and planar large-scale cross-bedding. These beds, which range from 5 to 50 cm in thickness, are also characterized by mud drapes, flaser bedding, vertical burrows and soft-sediment deformation. Rock colors include yellow, orange, red, and tan, with yellow beds sometimes weathering red.

A second sandstone facies comprising ca. 23% of sandstones consists of stacks of cross-bedded, fine- to medium-grained beds with sharp lower and upper contacts. Cross-bedding includes both trough and planar varieties commonly 10–20 cm deep, with a maximum depth of 40 cm. This facies is well-sorted, fine-grained, and buff, red, and occasionally white in color.

A third sandstone facies comprising ca. 16% of sandstones are red to orange, fine-grained beds interbedded with siltstones and mudstones. These sandstones, commonly 5–50 cm thick, often have ripple-scale cross-bedding, laminations, and mud drapes and preserve dinosaur footprints and burrows (Belvedere et al. 2013, Ibrahim et al.

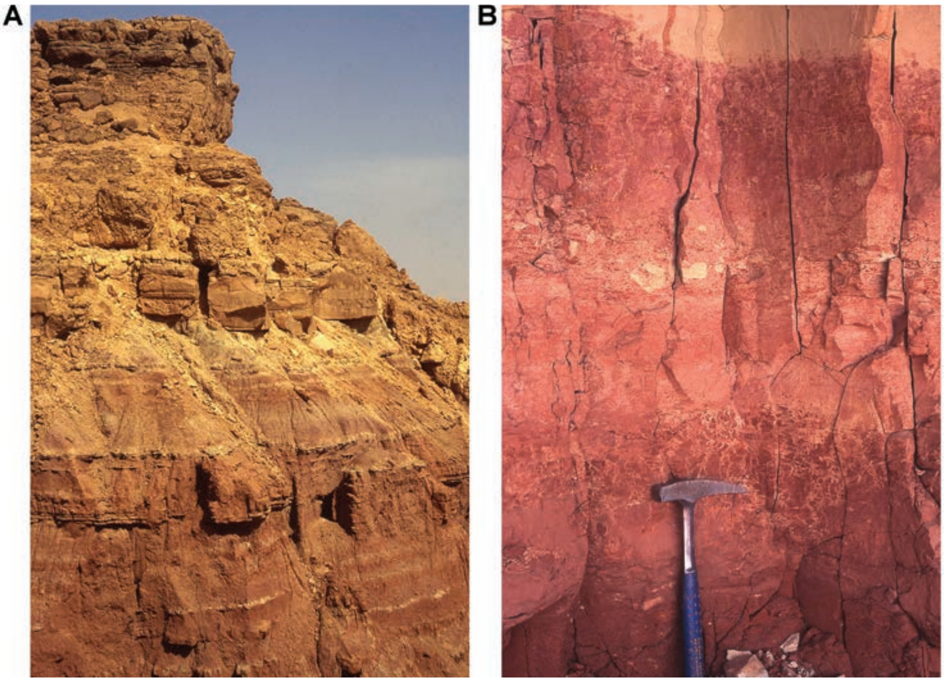

Figure 30. Primary sedimentary structures in the Douira Formation. **A** Fine-grained interbedding of the upper portion of the Douira Formation underlying the Cenomanian-Turonian limestone **B** Fine-grained mudrock with vertical paleosol zonation and root traces.

2014a). Typically this facies occurs with thinner overall sequences, usually less than 50 cm in thickness. However, some meters-thick inclined heterolithic stratifications were observed outside of the measured sections. Rarer sandstone facies include massive, medium-grained, orange to red-orange sandstones that are 2 m in thickness or less and yellow units. Finally, there are coarsening-up sequences, from one to a few meters thick.

Siltstones and very fine sandstones are persistent but uncommon in the Douira Formation, representing only ca. 10% of the entire stratigraphic thickness. Often these lithologies occur as units less than 50 cm thick, with bed thicknesses more commonly between 5 and 20 cm. Color may vary from bluish white to red-orange. Small-scale cross-bedding, laminae, and nonvertebrate traces are abundant. Occasionally these units also preserve mud-cracks, dinosaur footprints, mud drapes, and flaser bedding. Soft sediment deformation is rare.

Mudstones and claystones dominate the Douira Formation. In the complete and well-exposed sections, these fine-grained units represent an average of 64% of the stratigraphic thickness. Mudstones occur in four facies: reddish-brown massively bedded units (38%), interbedded mudstones (40%), minor laminated beds, and green claystone. Red-brown to red mudstones, which are common in the lower portion of the formation, are characterized by mottling, slickensides, and blocky to crumbly textures. Calcitic nodules, gypsum crystals, root traces, and burrows are rare.

Interbedded mudstone with claystone is the dominant facies of the upper half of the Douira Formation (Fig. 30). This facies typically shows a mix of diffuse bedding and gradational contacts together with sharper boundaries. Color of these mudstones includes red-brown, purple, red, green, and tan. Mottling and slickensides occur commonly. These lithologies exhibit occasional root traces, and rare four-sided mudcracks and are variably calcareous, with massive blocky and crumbly textures.

Laminated mudstones, best developed at Oum Tkout (Figs 17, 31) and composed mainly of illite, are a minor component of the lower portion of the formation. They are variable in color (red-brown, tan, gray, white). At Oum Tkout, this facies represents a pond setting that preserved an array of plants, insects, decapods, elasmobranchs, and actinopterygians (e.g., Dutheil 1999a, 1999b). The last minor mudstone facies is green claystone, which occurs just below the massive limestone of the Cenomanian-Turonian transgression.

Limestones and marls, which comprise a very small fraction (< 1%) of the Douira Formation consist of blueish gray-to-white, ledge-forming beds less than 50 cm thick with substantial clay and silt. This facies occurs in the upper 20 m of the Ouzina section. At Douira calcareous beds are more common and comprise 7% of the section. Beds 20–30 cm in thickness occur inter-bedded with mudstones and claystones. One very thin (1 cm) gypsum horizon, in addition, occurs just below a calcareous mudstone. This facies and more significant evaporite deposits become much more common in beds of the Kem Kem Group outside the Kem Kem Hamada (Table 7).

Paleontology. Fossils are less abundant than in the Gara Sbaa Formation, although the same range of vertebrate taxa are recorded. Associated dinosaur remains from the Douira Formation include the cranium of *Carcharodontosaurus* and partial skeleton of *Spinosaurus* (Sereno et al. 1996, Ibrahim et al. 2014b).

Trace fossils are common in many horizons. The finer-grained deposits and soil development facilitated footprint and other trace fossil preservation. Horizons with vertebrate tracks occur throughout the formation from within a few meters of its base to within 6 or 7 m of the overlying limestone platform (Ibrahim et al. 2014a). They are most common in a "footprint zone" from 30 to 10 m below the top of the Douira Formation (Sereno et al. 1996: fig. 1C). Tracks pertain to medium- and large-sized theropods and, much less frequently, large-sized ornithopods and sauropods (Sereno et al. 1996, Belvedere et al. 2013, Ibrahim et al. 2014a). Tracks occur as impressions and natural casts, the latter the more common and usually occurring in the rare siltstone and sandstone beds. Some are particularly deep and show striations from the motion of the toes through the soft sediment. Nonvertebrate traces include *Conichnus*, a possible resting trace of a sea-anemone, *Scolicia*, a gastropod trail, worm-like *Beaconites* horizontal meniscate burrows, and short, sub-vertical burrows that appear to be dwelling traces of a crustacean (Ibrahim et al. 2014a). These last two trace types occur together in abundance and may represent the activity of detritivores on a tidal flat.

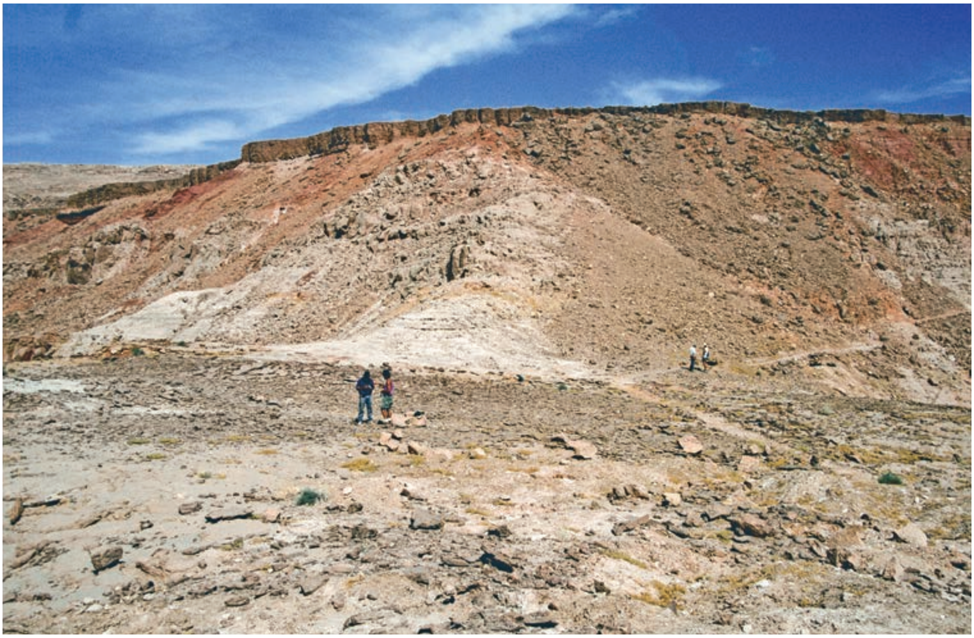

Figure 31. Oum Tkout locality where quarrying has recovered an abundance of exceptionally preserved aquatic vertebrates and invertebrates in a still-water setting.

Paleogeography and paleoenvironments

Cenomanian paleogeography. Currently, there are no major river drainages from northern Africa into the Mediterranean west of the Nile River. In Morocco uplift during the Cenozoic created the Alpine Belt to the north of the Preafrican Trough, cutting off drainage to the Mediterranean. In Cretaceous time, however, a major drainage existed between eastern Morocco and western Algeria that flowed northward into the Tethys Ocean (Delfaud and Zellouf 1995). Our paleocurrent measurements and those of Cavin et al. (2010) strongly support this drainage direction (contra Russell 1996). The Morocco-Algeria border region, and in particular the Kem Kem embayment, would have provided a ramp for northward drainage from the western Sahara (Fig. 32). Interconnected basins along that trough may also have allowed secondary drainage from the Kem Kem embayment to the west into the central Atlantic Ocean (Delfaud and Zellouf 1995, Essafraoui et al. 2015). The Kem Kem embayment and Kem Kem Group formations, thus, may be envisioned as the headland of a vast river system feeding north to a prograding delta (Fig. 33).

An evolving delta. The Gara Sbaa and Douira formations of the Kem Kem Group in Morocco have long been envisioned as deltaic in general character (Sereno et al. 1996). Sedimentary structures of the Gara Sbaa Formation were deposited by an anastomosing fluvial system, starting with conglomeratic beds over eroded Paleozoic strata (Fig. 32, stages 1, 2). Fining-upward sediments and sediment cycles characterize the

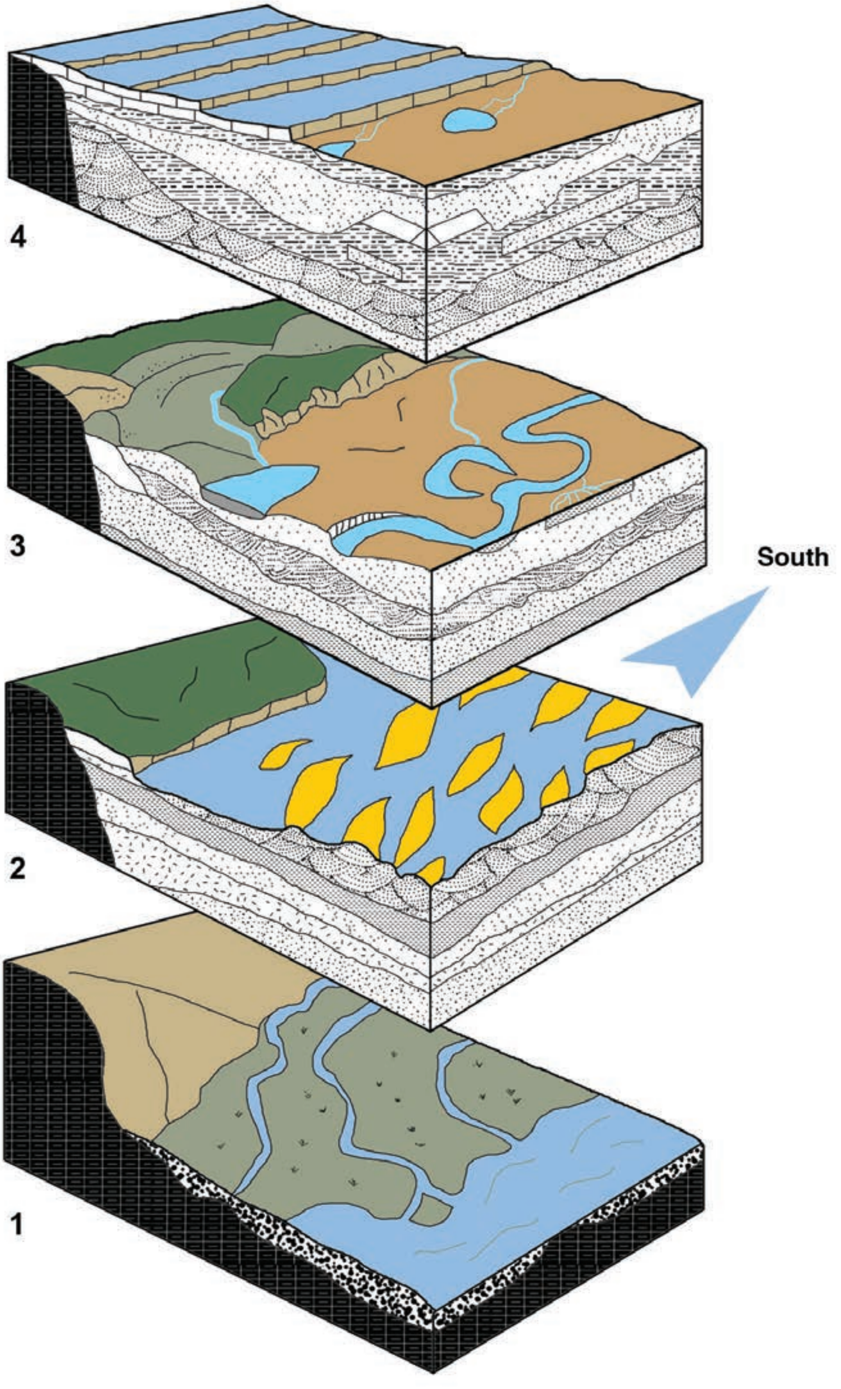

Figure 32. Schematic paleoenvironmental stages depicting the Kem Kem region during the Cretaceous. Stages: **1** wide rivers **2** large river systems with substantial sandbanks **3** deltaic conditions **4** rise of the limestone platform.

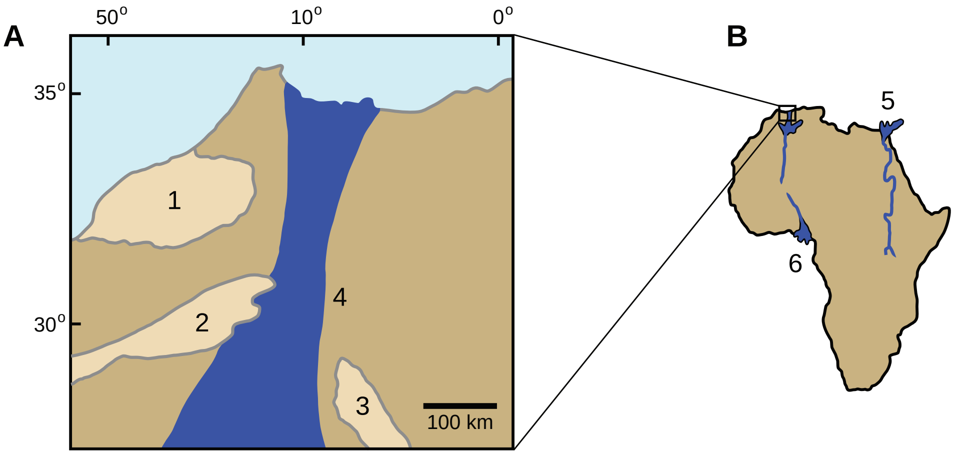

Figure 33. Schematic geography of the major rivers systems in northern and western Africa during the Late Cretaceous (modified from Zellouf and Delfaud 1986, Delfaud 1986, Dutheil 2000). **A** Kem Kem river system with flow to the north **B** Kem Kem, paleo-Nile and paleo-Niger river systems. Abbreviations: **1** Meseta **2** Anti-Atlas **3** Ougarta **4** Kem Kem river and delta **5** Paleo-Nile river and delta **6** Paleo-Niger river and delta.

Gara Sbaa and Douira formations. Prograding delta sediments of the upper Gara Sbaa and lower Douira Formation give way and coastal deposits and sabkas in the upper part of the Douira Formation, prior to inundation by a marine transgression (Guiraud et al. 2005, Figs 34, 35). The Gara Sbaa and Douira formations, thus, capture the transition, likely in the Early and Middle Cenomanian, from fluvial to deltaic to lower-energy coastal, pond and sabkha paleoenvironments. Coastal mangrove deposits, recorded in the likely coeval Bahariya Formation in Egypt (Smith et al. 2001), are not present in the sediments of the Kem Kem embayment.

During deposition of the Gara Sbaa and Douira formations as well as other Kem Kem Group formations recognized to date (Table 7), marine influence increases, sedimentary gradient decreases, and lower energy paleoenvironments predominate. The vertebrate fauna, as best as can be determined from transported fossils, does not change appreciably during this interval. Modern analogs for Kem Kem Group beds in Morocco must include a large-scale river system coursing through arid habitats to a prograding delta within reach of a sea or ocean. On Africa the best present-day analog is the Niger delta (Reijers et al. 1997).

Gara Sbaa sediments and paleoenvironments. The conglomeratic components, locally derived clasts, and mix of smaller sandstones indicates within-basin deposition via small-scale fluvial systems at the base of the Gara Sbaa Formation (Fig. 32, stage 1). The increase in bed thickness and larger-scale cross-bedding of overlying sandstones indicate broader and deeper fluvial channels (Fig. 32, stage 2). The rarity of mudstones, the increased lateral extent of sandstone bodies, and their more mature composition indicate reduced accommodation space and lateral redeposition by channels of earlier

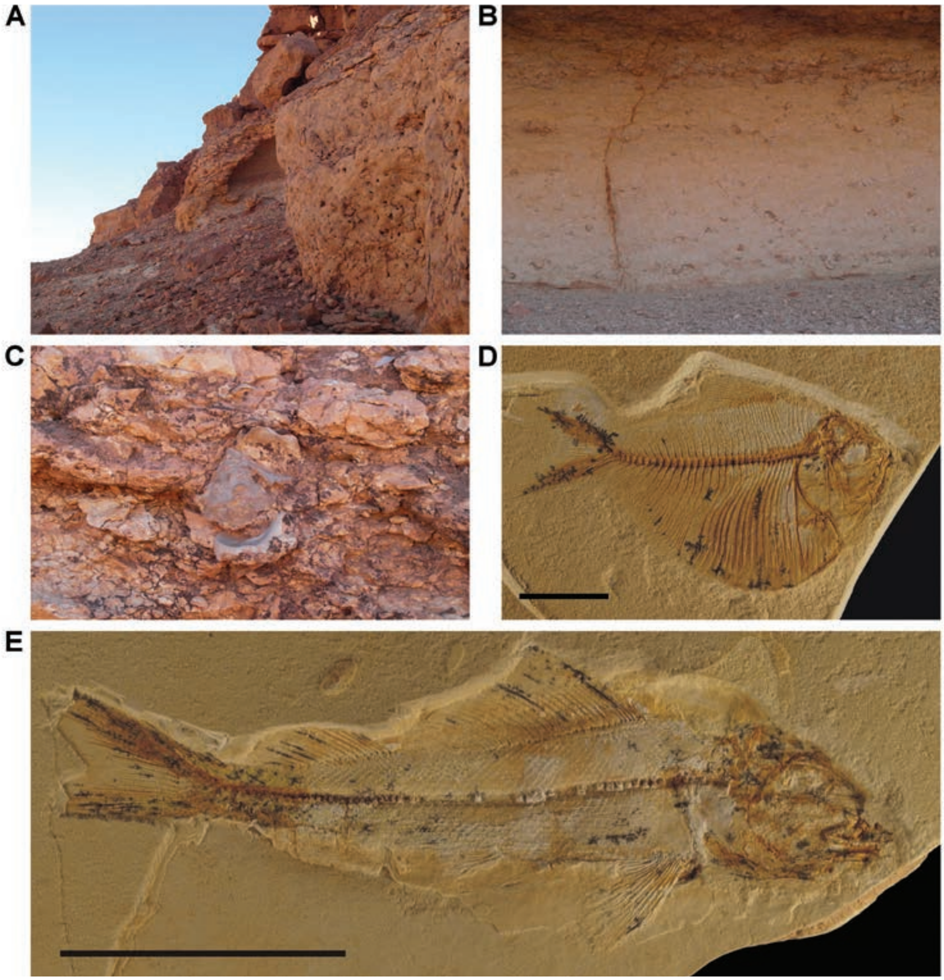

Figure 34. Geology and paleontology at the boundary between the Douira Formation and the Gara Sbaa Member of the Cenomanian-Turonian limestone at Gara Sbaa. **A** Marly limestone separating the Kem Kem Group from the Cenomanian-Turonian limestone **B** Close-up of a basal limestone unit in the Gara Sbaa Member **C** Oyster fossil in situ in the basal limestone **D** Deep-bodied teleost (*Diplomystus* sp.) from the Gara Sbaa Member of the Cenomanian-Turonian limestone **E** Long-bodied teleost (*Agoultichthys chattertoni*) showing preservation of soft fin structures from the Gara Sbaa Member (Murray and Wilson 2009) **D** and **E** from Martill et al. (2011). Scale bars equal 1 cm in **D** and 2 cm in **E**.

channel and floodplain deposits. Reworking may have generated the conglomeratic beds with rip-up clasts and pebbles that contain vertebrate teeth and bone (Fig. 36; Rogers 1993).

Several possible indicators of tidal influence occur within the upper portion of the Gara Sbaa Formation through the Douira Formation. These include mud drapes, flaser and lenticular bedding and inclined heterolithic strata. The maturity of the sandstones within this stratigraphic interval may also reflect tidal influence (Tucker 2011). The

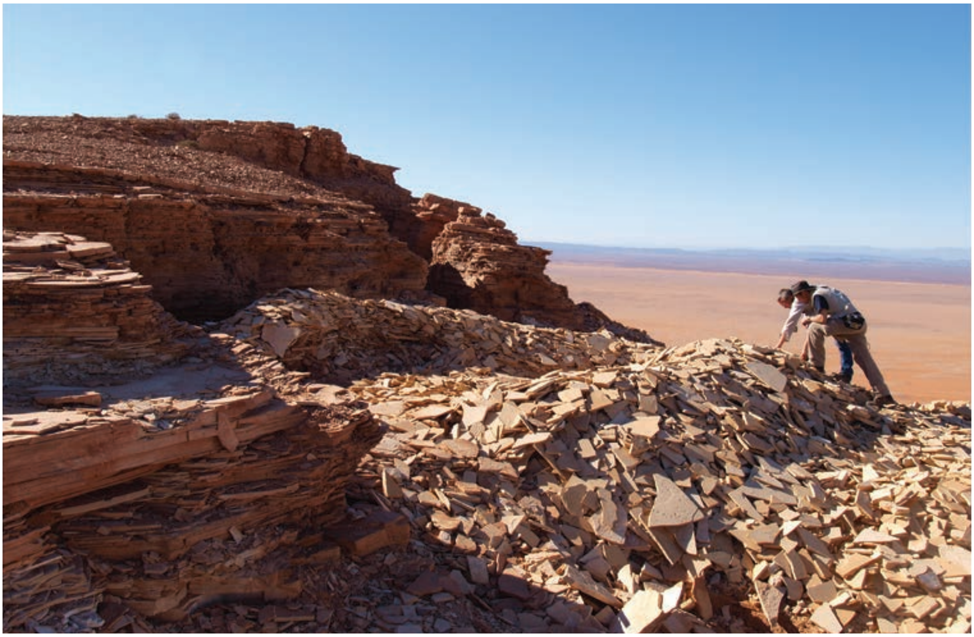

Figure 35. Fissile weathering of the Cenomanian-Turonian limestone platform.

larger, possibly tidally influenced, channels of the upper portion of the Gara Sbaa Formation indicate deltaic environments. Large-scale cross-bedding with depths exceeding 6 m may indicate progradation of the delta front (Fig. 24).

Douira sediments and paleoenvironments. Evidence favoring deltaic progradation is limited to the lowermost portion of the Douira Formation. As the transgression continued, the entire Kem Kem fluvial system appears to have stalled. Grain size and channel forms diminish up-section. In the deeper northern region of the ramp, evaporites and limestones become more common as clastic input waned. The lowered gradient of the Douira Formation largely consists of a variety of low-energy depositional environments under tidal, and later more fully marine, influence.

These depositional settings involve smaller fluvial channels, floodplains with some incipient paleosol development with root traces, crevasse splay deposits important for the preservation of dinosaur tracks, and a freshwater pond deposit at Oum Tkout with decapods and small-bodied bony fishes (Fig. 32, stage 3; Dutheil 1999a). Marginal marine environments also occur within the uppermost Douira Formation prior to the deposition of the limestone platform of the Akrabou Formation (Fig. 32, stage 4). These environments consist of shallow water tidal flats represented by mudstones with abundant simple tubular dwelling traces or *Conichnus*, possible sea-anemone resting traces (Ibrahim et al. 2014a). In some sections, green claystones and marls occur at the top of the formation transitional to the overlying limestone. Arid to semi-arid conditions were common in low-latitude northern Africa during the Cenomanian (Scotese 2002), generating intervals of sabkha-like conditions.

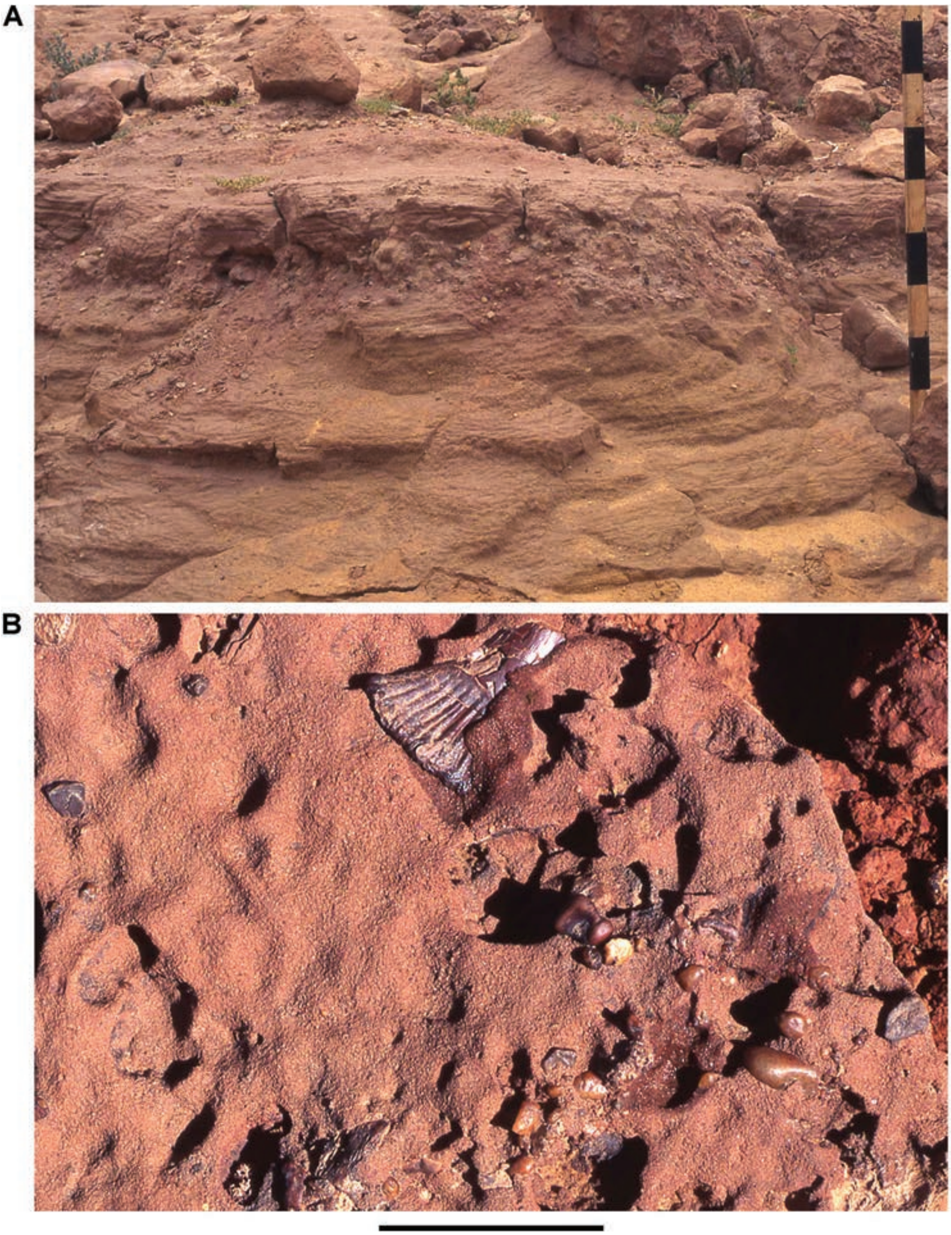

Figure 36. Pebble and fossil lag deposits in the Kem Kem Group. **A** Common pebble lag deposit composed of two beds **B** Abraded vertebrate remains including a partial sawfish rostral tooth (*Onchopristus numidus*). Scale bars equal 60 cm (10 cm intervals) in **A** and 5 cm in **B**.

Brackish water. The Kem Kem delta was dominated by rapidly moving freshwater/brackish (lotic) paleoenvironments with water flowing toward the open ocean, as opposed to the much rarer still water (lentic) paleoenvironments represented by

ponds. The Oum Tkout locality, interpreted here as a pond paleoenvironment, preserves small-bodied (< 10 cm) osteichthyans, such as polypterids and osteoglossiforms. Extant representatives live either in exclusively or predominantly freshwater habitats.

Within the much more common lotic paleoenvironments, the question arises as to whether these were predominantly freshwater, brackish or fully marine. Cartilaginous and bony fish can provide evidence regarding the nature of the water systems. Available evidence points to both freshwater and brackish conditions. Dipnoan tooth plates are common and support freshwater conditions, as all extant dipnoans occupy freshwater habitats.

A diverse assemblage of lamnifom shark teeth, however, suggests that brackish conditions were common. Although the batoid *Onchopristis dunklei* (McNulty and Slaughter 1962) is a coastal marine species, it is plausible that *O. numidus* was adapted to freshwater, as the taxon also occurs in Cenomanian deposits in Niger (Lapparent 1953, DBD pers. obs.) at a considerable distance from any maritime coast. Some extant batoids, such as the South American stingray *Potamotrygon* reside exclusively in freshwater habitats (Stauch and Blanc 1962) but can adjust if subjected to saline conditions (Thorson 1970). The same argument applies to the large Kem Kem coelacanth *Axelrodichthys lavocati* (previously referred to *Mawsonia*, Carnier Fragoso et al. 2019), which has also been found in freshwater deposits in Niger and Brazil.

In summary, the Kem Kem fluvial system shows evidence of both freshwater, and brackish conditions. Up section, in the upper portion of the Gara Sbaa Formation and the Douira Formation, tidal indicators suggest brackish conditions may have become stronger.

Hothouse climate. Hothouse conditions likely prevailed during deposition of Kem Kem Group rocks in much of the area now occupied by the Sahara, with harsh seasonality, arid conditions and strong convective storms predominating (Russell and Paesler 2003, Holz 2015). The Earth's climate is now widely understood as oscillating on a scale of millions of years between icehouse, greenhouse and hothouse states (Fischer 1982, Kidder and Worsley 2010, 2012, Wendler and Wendler 2016, Hu et al. 2017). During the Cenomanian, sea levels reached their maximum during the Cretaceous (Holz 2015), sea surface temperatures were very elevated (Boucot and Gray 2001), and temperate forests grew within the polar circles (e.g., Herman and Spicer 1996, Falcon-Lang et al. 2001).

Age

The ages of the Gara Sbaa and Douira formations are based on relative dating of a suite of nine elasmobranch genera collected from both the Gara Sbaa and Douira formations (Sereno et al. 1996). The overlying Akrabou Formation offers additional relative dates based on marine nonvertebrates, providing a young age limit for the underlying Kem Kem Group sediments (Ferrandini et al. 1985, Garassino et al. 2008, Martill et al. 2011a).

Nine elasmobranchs were collected in the Gara Sbaa and Douira formations (Sereno et al. 1996). Seven of these (and three theropod genera, *Carcharodontosaurus*, *Spinosaurus*, *Deltadromeus*) are shared with the Bahariya Formation in Egypt (Fig. 1),

also regarded as Cenomanian in age. One of these elasmobranch species (*Haimrichia amonensis* Cappetta & Case, 1975) has a broad circum-Mediterranean distribution and has been found elsewhere in Africa and Asia in Cenomanian-age strata (Vullo et al. 2016). No elasmobranch genera have been recovered with an age range restricted to the Albian or earlier. The evidence from elasmobranchs, thus, suggests a Cenomanian age for both the Gara Sbaa and Douira formations.

The Gara Sbaa, Douira, and Akrabou formations comprise a single, stepped transgressive sequence recording a succession of fluvial, deltaic, low-energy coastal environments, to finally an offshore carbonate platform. The formations do not show any major erosional or hiatal surfaces or incised channels that would argue for a contained regressive phase. Instead, marine influence increases steadily up-section to a comformable and gradational contact with the overlying Akrabou Formation. The contact between the Douira and Akrabou formations is conformable and shows almost no topography, as observed at many places along the Guir and Kem Kem Hamadas. A thin laminated clay layer a few centimeters thick is often present in well exposed sections at the top of the Douira Formation immediately below the first carbonate layers of the platform. This suggests that inundation and development of an initial coastal platform occurred swiftly without a significant temporal hiatus sometime in the Late Cenomanian.

This transgression corresponds in general to rising eustatic sea levels during the Cenomanian, although rising sea levels began during the Albian (Holz 2015). The depositional history of the Kem Kem Group appears to represent a single transgressive sequence leading up to this sea-level maximum. Global sea-level curves would thus suggest that the Kem Kem Formation is Cenomanian in age with a maximum age of approximately 98 or 97 Ma (Haq et al. 1987, Ogg et al. 2016) and a total duration of 3.5 to 4.5 Ma. The uniformity of the Kem Kem vertebrate fauna from the two formations is consistent with this relatively short temporal span.

The age of the boundary between the Gara Sbaa and Douira formations is an open question. That boundary is easily distinguished along the length of the Kem Kem region. It also appears to register as a regional event that occurs in comparable strata to the north and east (Table 7). This boundary, which separates a sandy deltaic unit from a predominantly gypsiferous mud, is linked to an abrupt rise of eustatic sea level. Along the Atlantic coast, there are many observable sea level cycles in sections across the Cenomanian, but these cannot be correlated westward by continuous outcrop to Kem Kem Group strata in central and eastern Morocco (Essafraoui et al. 2015). A marked sea level rise during the Cenomanian, nevertheless, is recognized above all others globally and in the well-studied Tarfaya Basin on the Atlantic coast of Morocco. Called the "Mid Cenomanian Event", it has been dated to approximately 96.0 Ma (Kuhnt et al. 2009, Holz 2015). Here we suggest that this global rise in sea level may be linked to the distinctive shift to finer-grained sedimentation observed in Kem Kem Group rocks.

Regarding the age of the Akrabou Formation, several studies of the carbonate platform in central Morocco have described the range of ammonites and many other nonvertebrates that correspond with two major transgressive events. The first transgression, located at the base of the Akrabou Formation, has yielded a diverse flora and

nonvertebrate (limulid, crustacean, insect), elasmobranch and actinopterygian fauna from localities atop buttes near Gara Sbaa with an estimated age near the end of the Cenomanian (Garassino et al. 2008, Martill et al. 2011a, Vullo et al. 2016, Lamsdell et al. 2020). At approximately 94.5 Ma, eustatic sea level swiftly rose to inundate near-shore environments across northern Africa and Europe (Voigt et al. 2006, Kuhnt et al. 2009, Essafraoui et al. 2015). The second transgression occurs within the platform sequence, when rising sea levels generated benthic conditions across much of northern Africa at the Cenomanian-Turonian boundary at approximately 93.6 Ma (Ferrandini et al. 1985, Parente et al. 2008).

The dates discussed above can be assembled into a chronology for Hamadian Supergroup rocks in central and eastern Morocco (Table 7). Deposition may have commenced on a prograding delta in the latest Albian or earliest Cenomanian approximately 100.0 to 99.0 Ma. Sedimentation shifted to finer-grained coastal sedimentation tied to the Mid Cenomanian Event around 96.0 Ma and later underwent rapid inundation and development of a carbonate platform at approximately 94.5 Ma. The platform waters deepened near the Cenomanian-Turonian boundary at approximately 93.6 Ma, with marine conditions persisting until the Middle Turonian regression around 92.0 Ma. The chronology sketched above suggests that the fauna we review in the pages that follow probably comes from a temporal interval within the Cenomanian of less than 5 Ma from approximately 100 to 95 Ma.

Taphonomy

Preservation. Five modes of preservation are possible to distinguish when fossils are found *in situ* in the Gara Sbaa and Douira formations: (mode 1) *channel lags* of concentrated resistant material (teeth, vertebrae, etc.); (mode 2) *microsite lags* of concentrated small material (especially teeth); (mode 3) *isolated elements* (bone fragments or teeth); (mode 4) *associated remains* (partial vertebrate skulls or skeletons); and (mode 5) a *pond deposit* (plants, small-bodied teleost fish and decapods).

Most body fossils in the Kem Kem region were probably preserved in modes 1–3 and are discovered as isolated, fragmentary pieces weathered out from sandstones in both formations. Associated or articulated vertebrate specimens (mode 4) are very rare (Fig. 37A). Fossils tend to collect in erosional lag horizons with other hard rock debris (Figs 36B, 38, 39), and so their precise mode of preservation is difficult to ascertain. Robust fossils, such as teeth or vertebral processes, predominate (Fig. 38C), and these are frequently broken and abraded to some degree from transport (Fig. 38B, D). Some small specimens show no discernable indications of transport (Fig. 38E, F), although their size may have reduced the evidence of abrasion.

The best preserved large-bodied vertebrates are isolated specimens of four dinosaurs from different locations, two in the Gara Sbaa Formation and two in the Douira Formation. The partial skeleton of *Rebbachisaurus garasbae* appears to be the only associated large vertebrate specimen recovered in the Gara Sbaa Formation. Its stratigraphic

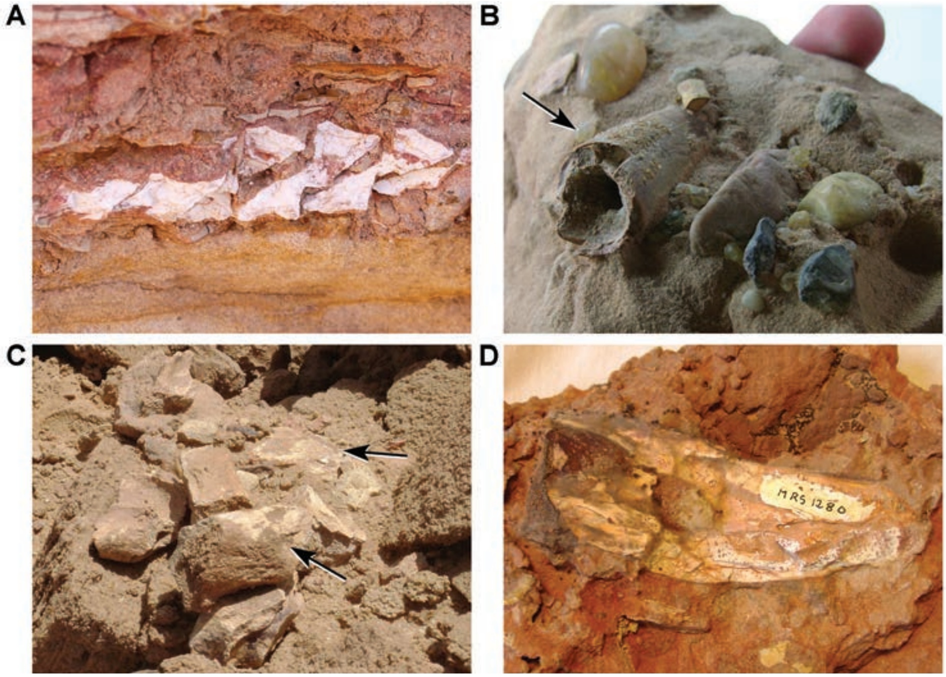

Figure 37. Taphonomy of Kem Kem vertebrate bone and teeth. **A** Articulated fish vertebrae in cross-section at Boumerade. **B** Cf. *Spinosaurus* tooth (arrow; FSAC-KK 201) adjacent to rounded pebbles **C** Concentration of broken bone fragments (<10 cm) **D** Partial archosaur tooth (MNHN-MRS 1280).

position and locality are based on the historical records of R. Lavocat (Wilson and Allain 2015: fig. 2). Also in the Gara Sbaa Formation at the locality Aferdou N'Chaft, a partial skeleton of *Deltadromeus agilis* was discovered weathering from a coarse-grained sandstone. The skeleton was preserved largely intact, as shown by articulated right and left shoulder girdles and forelimbs, right and left pedes and a section of caudal vertebrae and chevrons. It may have been buried suddenly in a channel deposit, judging from the coarse-grained matrix. In the Douira Formation, a partially articulated cranium of *Carcharodontosaurus saharicus* was discovered weathering from a fine-grained sandstone bluff several km distant attributed to the same locality. The braincase and several complete cranial bones were preserved including a pair of nasals close to one another and one maxilla with teeth preserved in most of the alveoli. Finally, a partial skeleton of *Spinosaurus aegyptiacus* was found near Zrigat in the northern exposures of the Kem Kem region. From the preserved position of some adjacent bones, it also appears to have been at least partially articulated. Inspection of the site of collection by several of us confirmed its origin in the Douira Formation and that it was preserved in isolation. Given their partial articulation, these four dinosaur specimens could not have been transported significantly before final burial.

The most complete fossils are from a singular pond deposit, Oum Tkout (mode 5), discovered in 1995 and revisited several times in the ensuing years (Dutheil

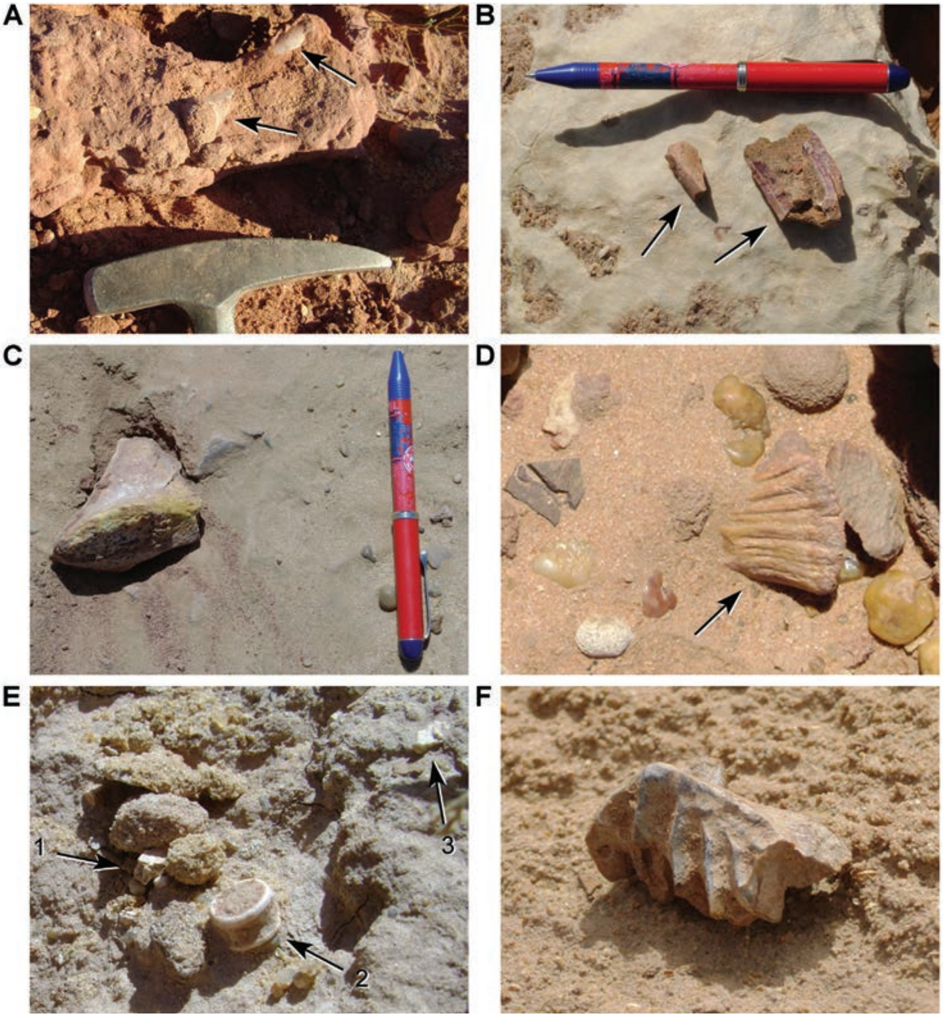

Figure 38. Taphonomy of Kem Kem vertebrate bone and teeth. **A** Isolated theropod quadrate ('valley near Boumerade' locality) **B** Fragmentary archosaur teeth (Gara Sbaa) **C** Cf. *Spinosaurus* vertebral zygapophysis (Gara Sbaa) **D** Abraded sawfish (*Onchopristis numidus*) rostral tooth **E** Isolated mixed sample of small (<1 cm) fossil vertebrates (Boumerade) **F** Lungfish toothplate (Boumerade). Abbreviations: **1** Turtle carapace fragment, **2** Fish vertebra, **3** Calcified rostral cartilage of *Onchopristis numidus*.

1999b). Fossils are recovered by quarrying and splitting small blocks of the fine-grained pond deposit. The locality contains small-bodied teleost fishes, prawns, macruran decapods and other soft-bodied nonvertebrates (Fig. 40). The fossils are not concentrated in a single layer or oriented in any particular direction. The rarity of broken specimens suggests that bottom feeding on the remains of decapods and actinopterygians may not have been common. Plant debris is common. The light color of the illite clays that form the majority of the pond mud suggests that anoxia was not a prevalent condition.

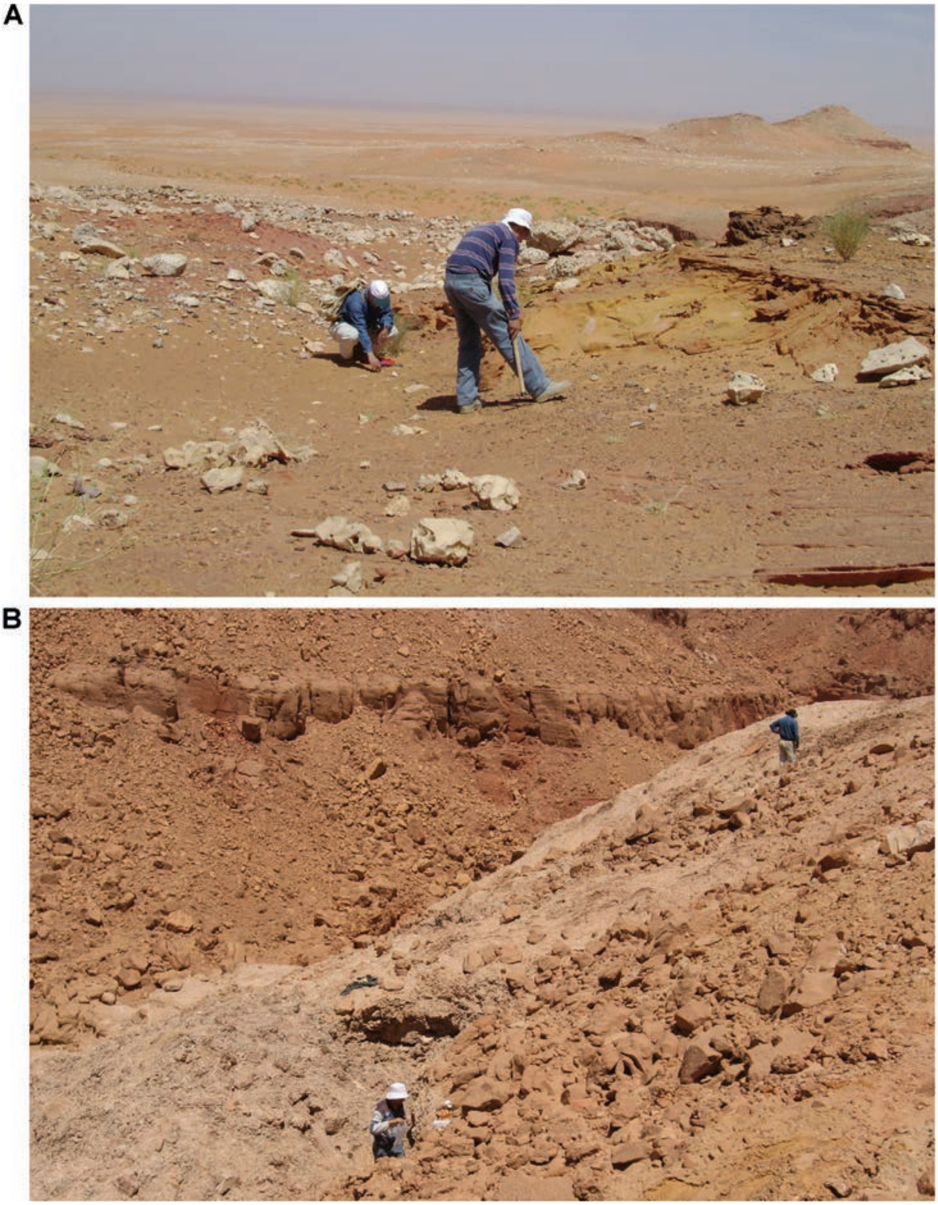

Figure 39. Surface and in situ collecting. **A** Surface collecting at Gara Sbaa **B** Collecting in situ fossils at Boumerade.

A number of bone elements preserve large numbers of borings (Ibrahim et al. 2014a), providing evidence of unrecorded soft-bodied nonvertebrate diversity (Fig. 41). These borings may have been made by insect larvae similar to those of the carrion beetle, *Osteocallis mandibulus*. The borings together with weather-induced cracking suggest that some bones were subaerially exposed for significant time prior to transport and burial (Roberts et al. 2007, Ibrahim et al. 2014a, fig. 3c).

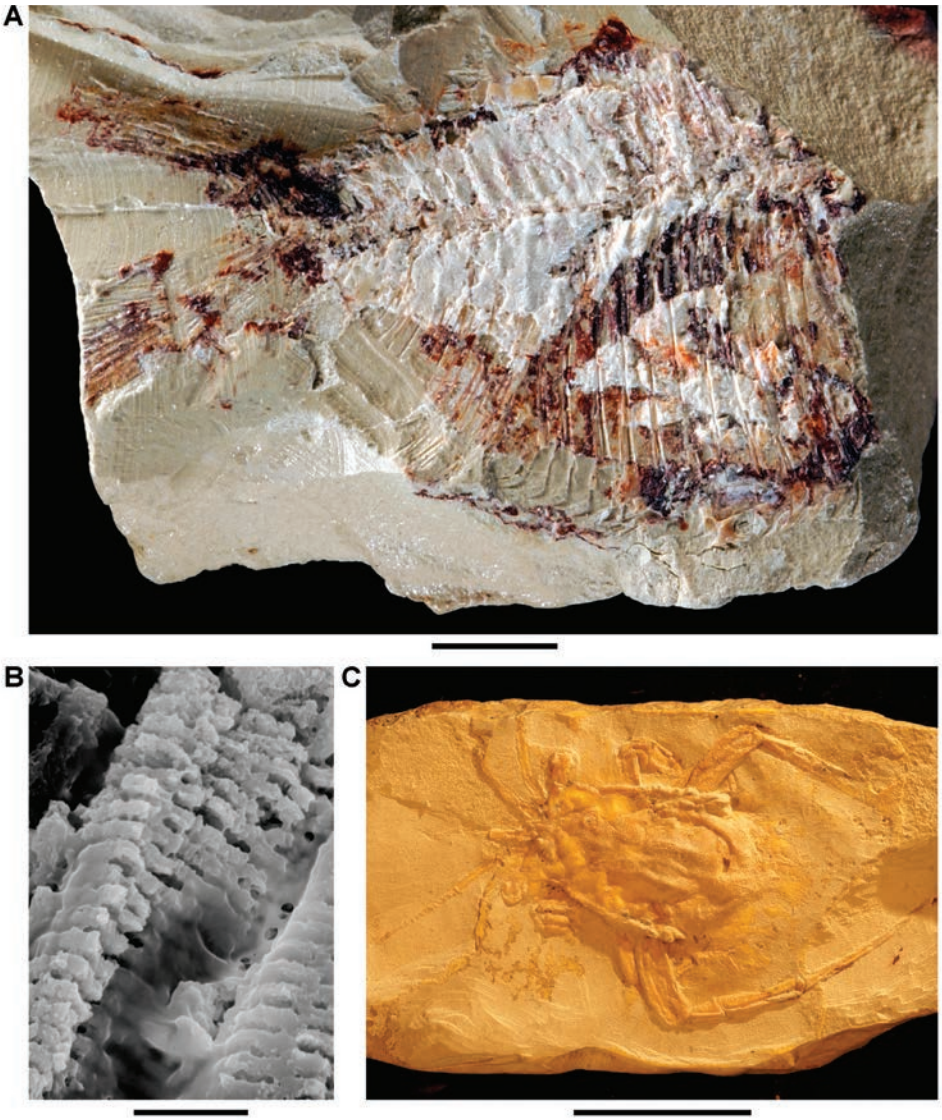

Figure 40. Taphonomy at Oum Tkout. **A** Indeterminate paraclupeid fish with mineralized muscles (white) **B** Soft tissue (muscle) preservation under high magnification **C** Complete decapod (UCRC PNV2). Scale bars equal 5 mm in **A**, 10 μm in **B**, 3 cm in **C**.

Nearly all specimens, except those in the singular pond deposit, are preserved in sandstone varying in grain size and degree of silicate cementation; the mudstones composing portions of the Douira Formation appear to be barren. Poorly cemented sandstone is the most common matrix, which is easily removed (Fig. 42A). Patches of more strongly cemented sediment occur (Fig. 42B), and some specimens have a thin hematitic layer adhering to bone surfaces (e.g., the cranium of *Carcharodontosaurus*). The matrix varies in color from yellow or beige to orange, pink and dark red (Fig. 42C).

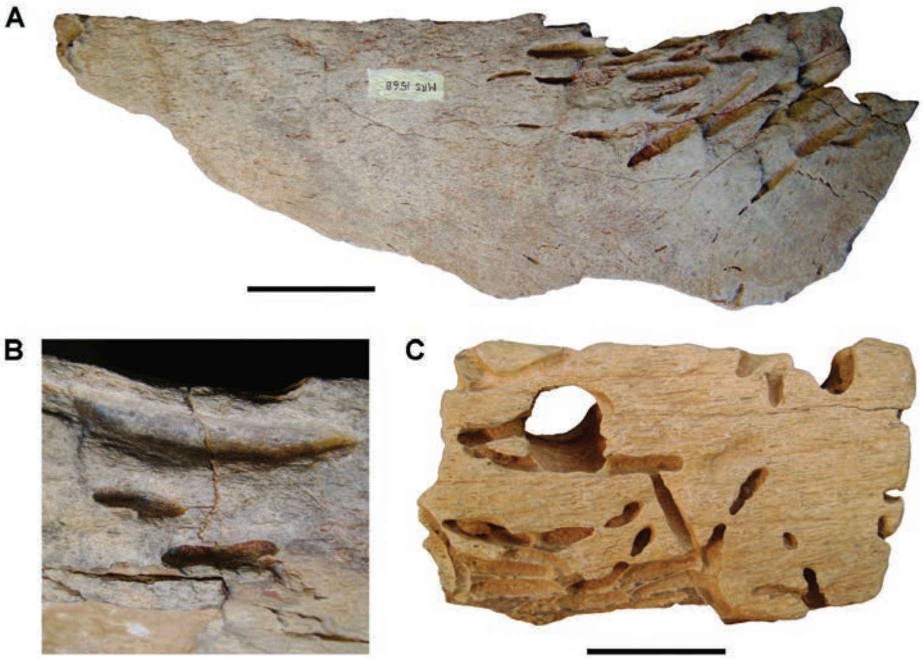

Figure 41. Invertebrate boring structures in dinosaur bone. **A** Large bone piece showing local concentration of burrows (MNHN-MRS 1568) **B** Close-up view of two burrows on large bone piece **C** Burrows of diverse diameter (UCRC I1). Scale bars equal 5 cm in **A** and 3 cm in **C**.

Similarly, the fossils exhibit a wide range of colors, although orange and red-brown are most common, with teeth commonly a darker color (Fig. 43). Some localities yield fossils of a particular color. Specimens from the Kouah Trick locality collected in the 1950s by R. Lavocat are very dark (Fig. 43B). Specimens from Douira, on the other hand, are often almost white or cream (Fig. 43A). In general, matrix and fossil color is highly variable and cannot be used to confidently identify place of origin.

Completeness. Quantitative logging of isolated specimens collected from several localities in 2008 shows that the majority (~75%) are too incomplete to estimate the percentage of missing bone. Of the remaining more complete specimens, more than half are less than 50% complete. At one locality, Aferdou N'Chaft, approximately half of the collected specimens are nearly complete, although this may be an artifact of small sample size. Clearly most specimens found in the Kem Kem Group are very fragmentary.

The prevalence of breakage among fossils suggests that they were deposited in a relatively high-energy environment and either reworked or transported a considerable distance. Bone breakage, however, does not appear to be a good proxy for distance of fluvial transport (Behrensmeyer 1991). Insect-boring of bones suggests that some terrestrial vertebrates were exposed subaerially before transport and burial.

Abrasion. Most of the fossils can be assigned to abrasion category 2 of Anderson et al. (2007, Table 3). Relatively few are highly abraded (Fig. 44A). At Boumerade,

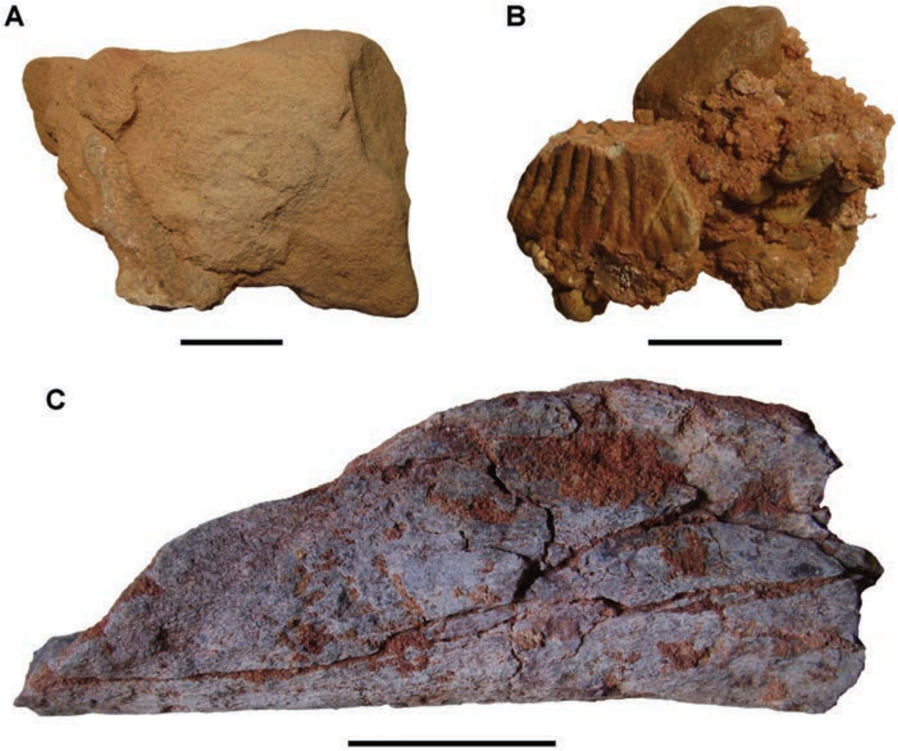

Figure 42. Sedimentary matrix associated with Kem Kem fossils. **A** Well-sorted, fine-grained sandstone on vertebrate bone (Gara Sbaa) **B** More oxidized coarse-grained matrix with the base of a rostral tooth of *Onchopristis numidus* ("valley near Boumerade") **C** Moderately-cemented, red-colored matrix on an isolated archosaur bone (MNHN, unnumbered). Scale bars equal 3 cm in **A**, 2 cm in **B**, 5 cm in **C**.

"Valley near Boumerade", and Aferdou N'Chaft, abrasion is minimal (Figs 45A, B, 46B). At Gara Sbaa a greater range of abrasion is present (categories 1–3, Fig. 45B). Systematic variation in the degree of abrasion between localities may indicate differences in the distance of fluvial transport prior to burial.

Bones, however, can travel long distances without accumulating signs of abrasion (Behrensmeyer 1982, 1991). Other confounding factors in the interpretation of abrasion include the potential for reworking of sandstone deposits within the Gara Sbaa and Douira formations. The time that fossil material has been subject to subaerial weathering in lag deposits prior to collection may represent another potentially confounding factor when considering breakage and abrasion.

Specimen size. Although Kem Kem fossils vary across a wide size range from < 1 cm to 2 m (Fig. 47), the vast majority of specimens measure less than 10 cm in length (Fig. 48). Gara Sbaa provides the best estimate of the range in specimen size, given the large number of fossils collected (Fig. 48B). Specimens collected in finer-grained sediment at Boumerade and the "Valley near Boumerade" tend to be smaller, the largest rarely exceeding 4 cm (Figs 48A, 49B). Aferdou N'Chaft and Iferda N'Ahouar locali-

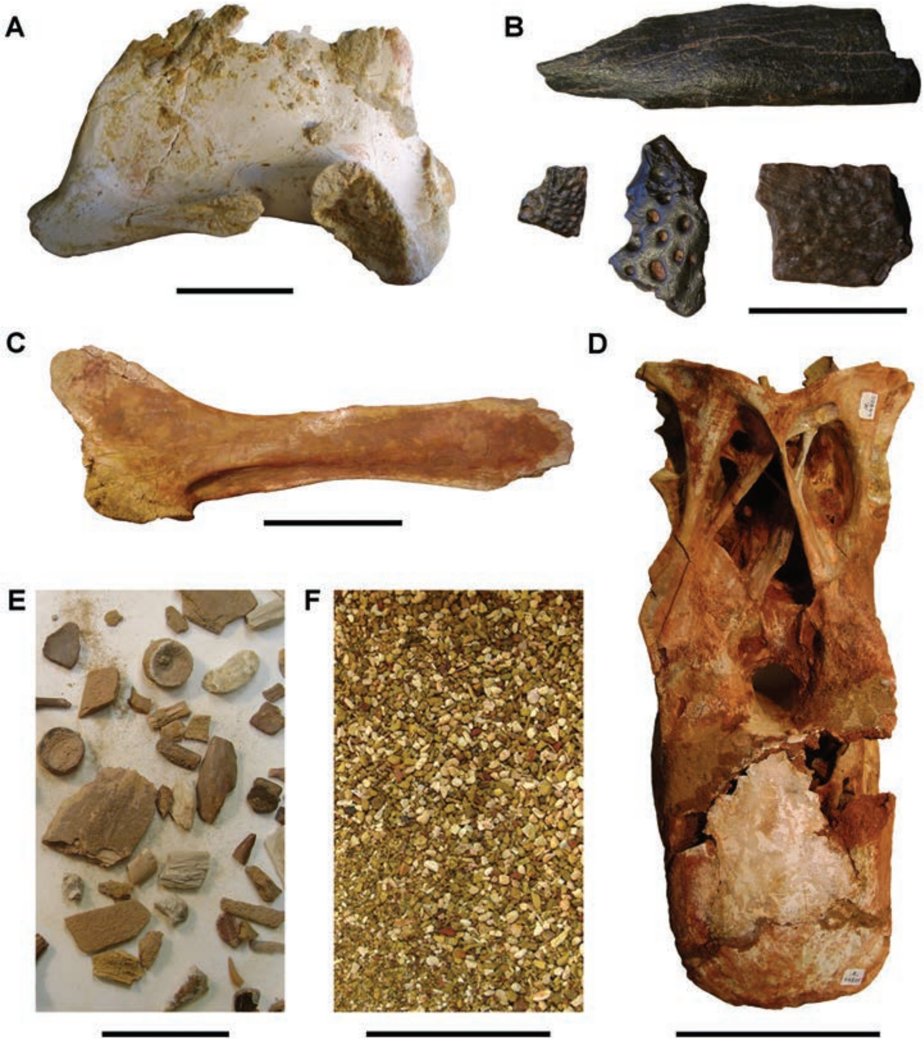

Figure 43. Color and size variation in Kem Kem vertebrate fossils. **A** Theropod distal quadrate (likely from Douira) **B** Archosaur bone and scute fragments (collected by R. Lavocat, Kouah Trick) **C** Theropod left scapula (MPDM 40, unknown locality) **D** Cf. *Rebbachisaurus* partial dorsal vertebra (NMC 50844, unknown locality) **E** Small and medium-sized fossil fragments (UCRC unnumbered) **F** Sieved microfossils (UCRC unnumbered). Scale bars equal 5 cm in **A** and **B**, 10 cm in **C**, 20 cm in **D**, 5 cm in **E**, 3 cm in **F**.

ties seem to have preserved a greater number of larger specimens (Figs 48B, 49A). The size differential between these localities may reflect variance in the hydrodynamics of the deposits. Somewhat larger specimens would be expected to be transported and buried in the higher-energy deposits of the Gara Sbaa Formation. It must be noted, however, that specimens eroding from the Douira Formation may well accumulate in lag deposits on the underlying outcrop of the Gara Sbaa Formation. As the majority of

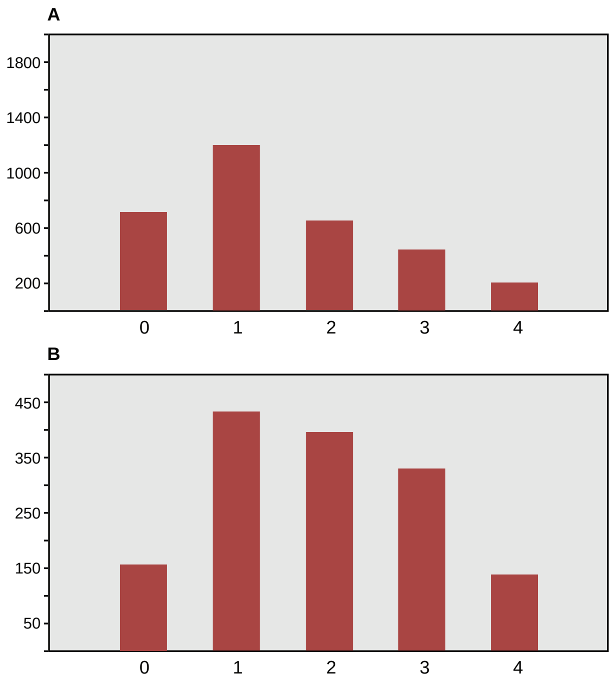

Figure 44. Abrasion of Kem Kem fossils (FSAC-KK collection). **A** All localities **B** Gara Sbaa. X-axis is the abrasion index (0-4; see Table 3); Y-axis is the number of specimens. Specimen counts in **A**: **0** 707 **1** 1207 **2** 647 **3** 439 **4** 195. Specimen counts in **B**: **0** 158 **1** 433 **2** 396 **3** 330 **4** 138.

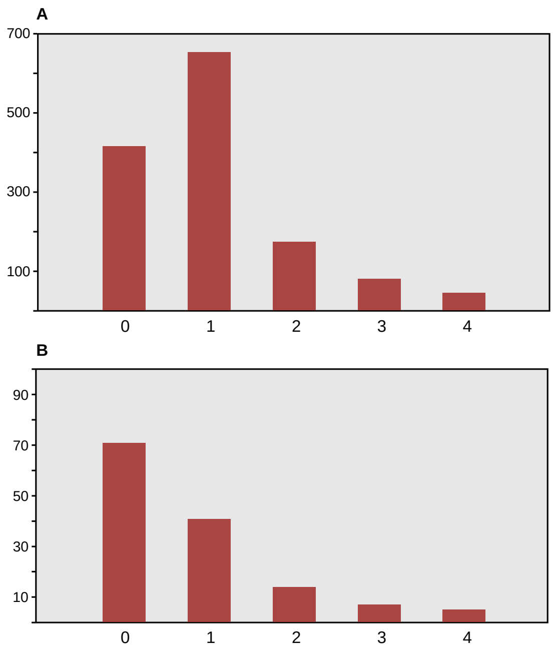

Figure 45. Abrasion of Kem Kem fossils (FSAC-KK collection). **A** Boumerade. **B** Aferdou N'Chaft. X-axis is the abrasion index (0-4; see Table 3); Y-axis is the number of specimens. Specimen counts in **A**: **0** 415 **1** 652 **2** 178 **3** 81 **4** 44. Specimen counts in **B**: **0** 71 **1** 41 **2** 14 **3** 7 **4** 5.

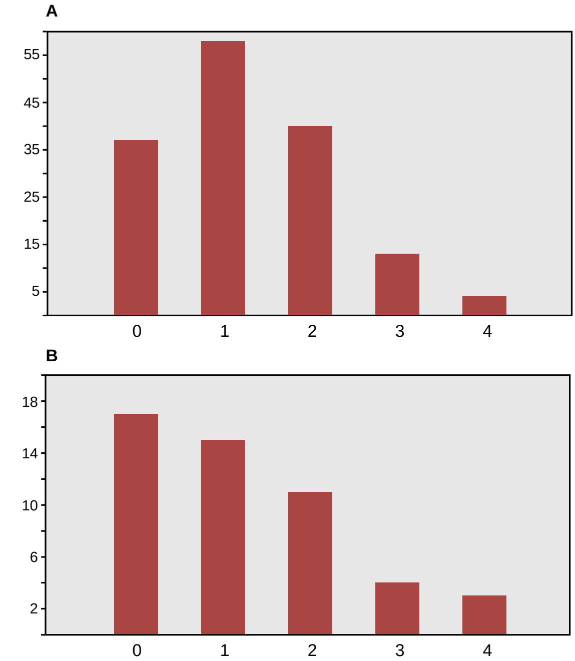

Figure 46. Abrasion of Kem Kem fossils (FSAC-KK collection). **A** Iferda N'Ahouar **B** Valley near Boumerade. X-axis is the abrasion index (0-4; see Table 3); Y-axis is the number of specimens. Specimen counts in **A**: **0** 37, **1** 58, **2** 40, **3** 13, **4** 4. Specimen counts in **B**: **0** 17, **1** 15, **2** 11, **3** 4, **4** 3.

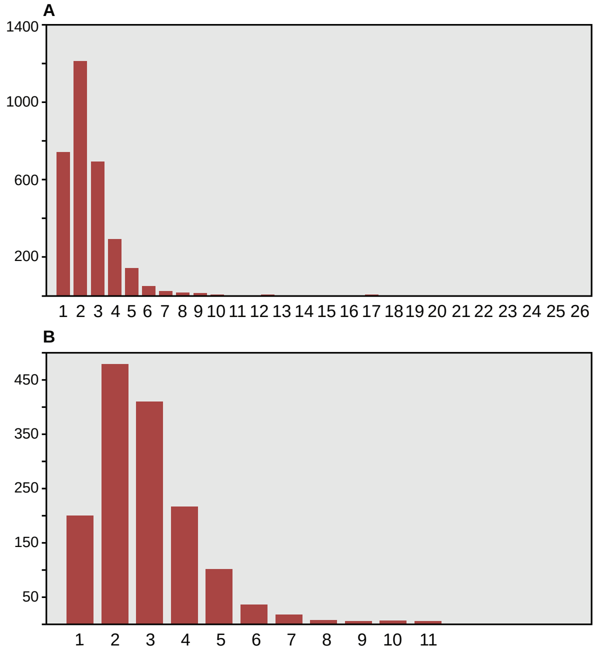

Figure 47. Size of vertebrate fossil elements found in the Kem Kem Group (FSAC-KK collection). **A** All localities. **B** Gara Sbaa. X-axis indicates size in 20 mm size bins; Y-axis indicates numbers of specimens. Specimen size bins: **1** 0-20 mm **2** 21-40 mm **3** 41-60 etc.

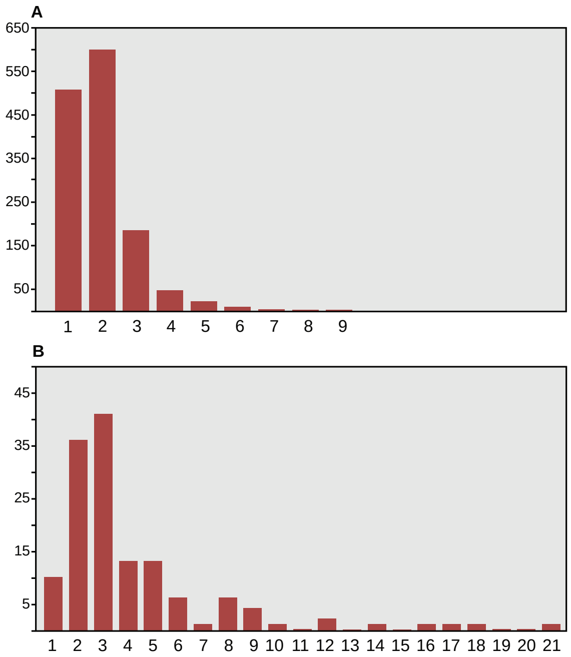

Figure 48. Size of vertebrate fossil elements found in the Kem Kem Group (FSAC-KK collection). **A** Boumerade. **B** Aferdou N'Chaft. X-axis indicates size in 20 mm size bins; Y-axis indicates numbers of specimens. Specimen size bins: **1** 0-20 mm **2** 21-40 mm **3** 41-60 etc.

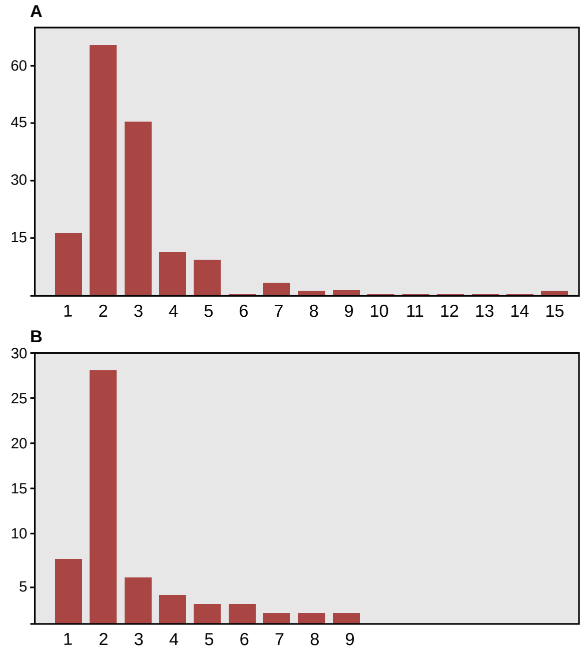

Figure 49. Size of vertebrate fossil elements found in the Kem Kem Group. **A** Iferda N'Ahouar **B** 'Valley near Boumerade' locality. X-axis indicates size in 20 mm size bins; Y-axis indicates numbers of specimens. Specimen size bins: **1** 0-20 mm **2** 21-40 mm **3** 41-60 etc.

specimens are not preserved *in situ,* some outcrops with considerable section exposed create uncertainty as regarding the formational provenance of specimens.

Very large fossil specimens are rare. Two partial sauropod limb bones were found in place. In 1995 the proximal end of a large sauropod ulna was discovered in the Gara Sbaa Formation, measuring 54 cm across its proximal articular end (see taxonomic section for further details). In 2008 the mid-section of a large titanosaur humerus was also recovered in this formation, measuring approximately 25 cm at the narrowest portion of its shaft and with a reconstructed length of approximately 1.5 m (Ibrahim et al. 2016).

In sum, there appears to be a strong taphonomic bias against very small (< 2 cm), large (> 6 cm), and soft (plant, nonvertebrate) specimens in the majority of localities in both the Gara Sbaa and Douira formations. The largest sample is from the locality Gara Sbaa, where nearly all vertebrate specimens fall into the 2–6 cm size range.

Systematics

Plant and nonvertebrate fossils

At the pond locality Oum Tkout, thin films are suggestive of bacteria or fungi (Eumycetes) (Fig. 50). Common plant fossils on Kem Kem outcrop include weathered pieces of petrified wood that probably represent araucarian conifers. Other macroplant remains at the pond locality Oum Tkout include other gymnosperms and spermatopsids (Fig. 51) and angiosperms (Garassino et al. 2006).

Body fossils of nonvertebrates are preserved almost exclusively at the pond locality Oum Tkout. The fine-grained mud sediment of the pond floor preserves whole and partial specimens of soft-bodied mollusks, crustaceans (prawn, macruran decapod, Fig. 52), and larval and mature insects (Fig. 53). The prawn *Cretapenaeus berberus* has been described from the freshwater locality Oum Tkout in the Douira Formation (Garassino et al. 2006) as well as in the overlying Akrabou Formation (Garassino et al. 2008). At least one macruran decapod remains to be described from Oum Tkout.

Elasmobranchii Bonaparte, 1838

The hooked rostral teeth of the sclerorhynchid, *Onchopristis numidus*, are the most common vertebrate fossil in Kem Kem group sediments (Dutheil 1999b, Cavin et al. 2010), readily found on outcrops along the length of the Guir and Kem Kem Hamadas (Fig. 54). This common northern African sclerorhynchid, initially described under the genus *Gigantichthys* from Algeria (Haug, 1905) and later placed in a new genus *Onchopristis* (Stromer 1917), has been recorded in Cenomanian-age rocks elsewhere in Algeria (Broin et al. 1971) and at sites across northern Africa, including Niger (Lapparent 1953, Dutheil 2001), Libya (Lefranc 1958) and Egypt (Stromer 1917, Tabaste

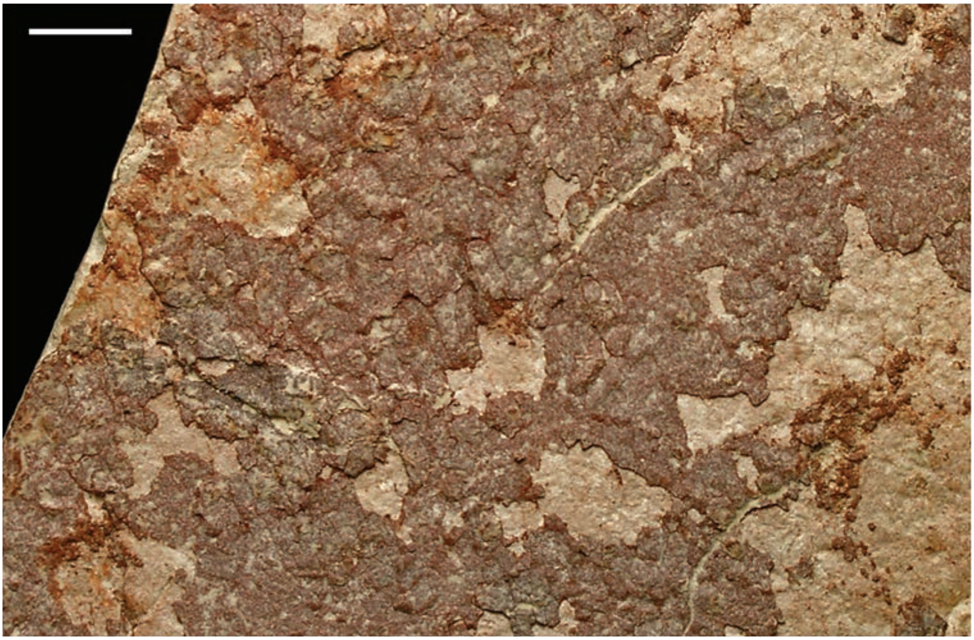

Figure 50. Possible biofilm (bacteria, fungi) from Oum Tkout, Douira Formation. Scale bar equals 5 mm.

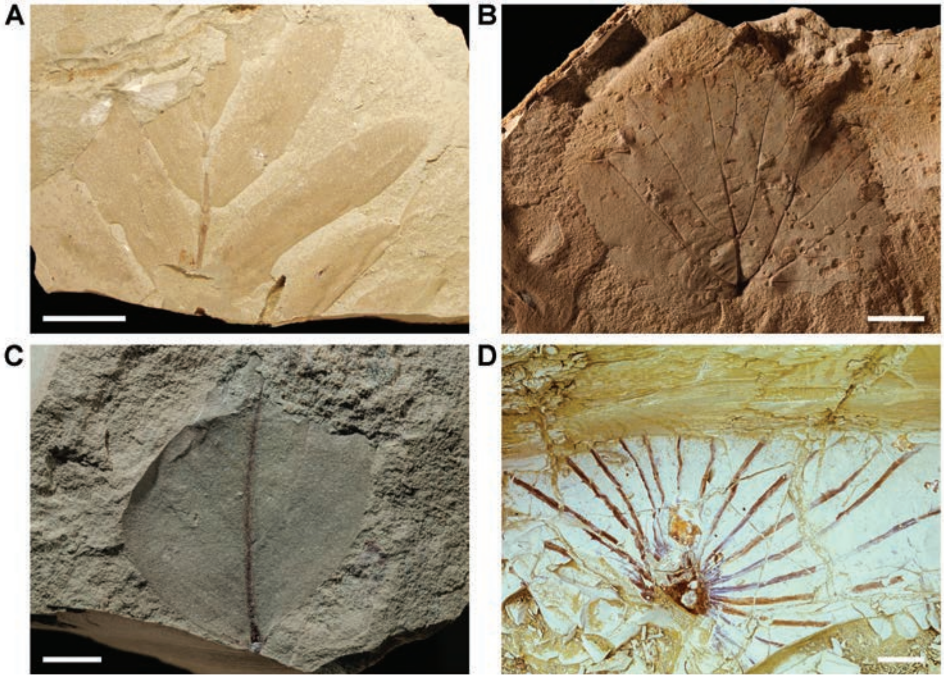

Figure 51. Simple and compound leaves are among the plant remains from Oum Tkout, Douira Formation. Scale bars equal 1 cm in **A** and **B**, 5 mm in **C** and 5 cm in **D**.

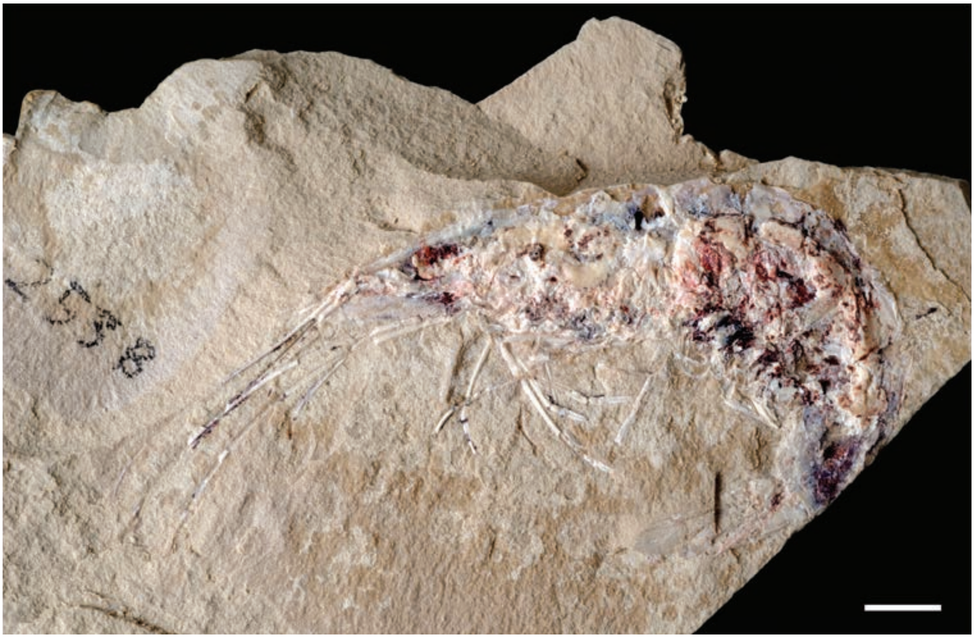

Figure 52. Decapod prawn *Cretapenaeus berberus* (Garassino et al. 2006) from Oum Tkout, Douira Formation. Scale bar equals 5 mm.

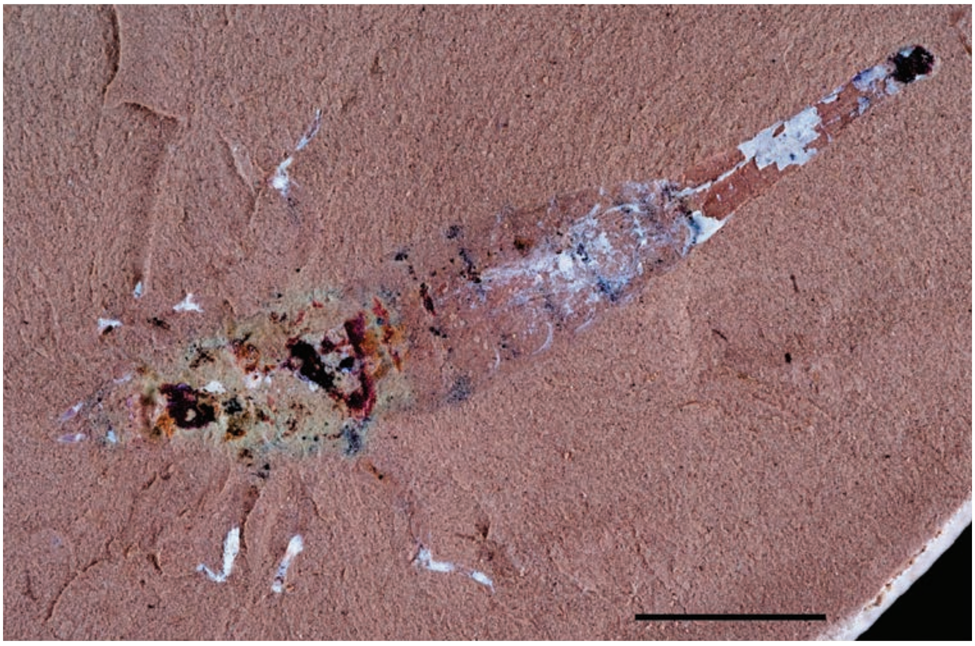

Figure 53. Dragonfly larva (Odonata indet.) from Oum Tkout, Douira Formation. Scale bar equals 5 mm.

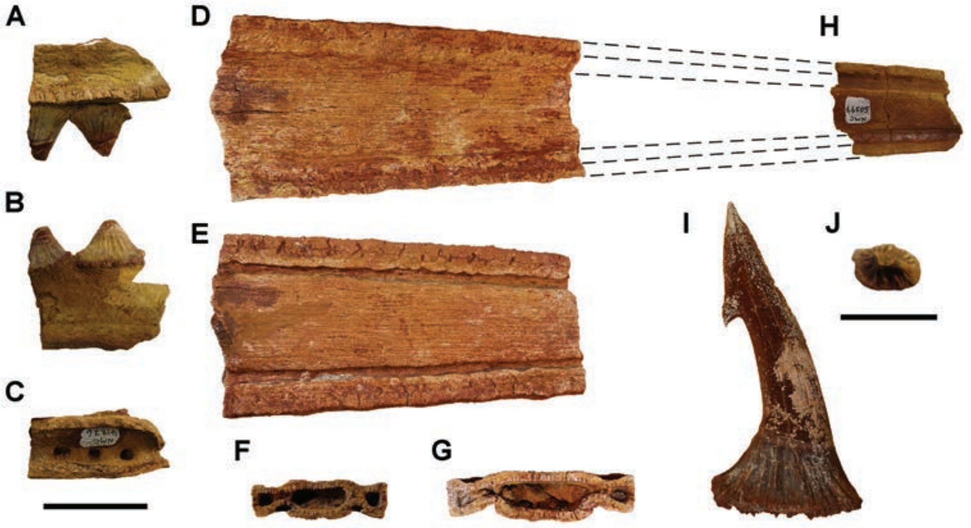

Figure 54. Selected isolated remains of the sclerorhynchid *Onchopristis numidus*. Rostral fragment with the bases of two rostral teeth in place (NMC 41876) in (**A**) ?dorsal, (**B**) ?ventral and (**C**) lateral view. Section of a large rostrum (FSAC-KK 937) in (**D**) dorsal, (**E**) ventral, (**F**) anterior and (**G**) posterior views **H** Small section of rostrum (NMC 50397) **I** Isolated rostral tooth **J** Placoid scale (MNHN-MRS 72) **D** and **H** reconstructed to show the approximate shape of the rostral blade. Scale bars equal 5 cm in **A-H**, 2 cm in **I** and **J**.

1963, Werner 1989). Most of the other elasmobranch genera reported in the Kem Kem Group come from screen-washing sediment for micro-vertebrate sampling (Dutheil 1996, Sereno et al. 1996, Cavin et al. 2010). These genera pertain to two clades of elasmobranchs, Hybodontoidea and Neoselachii (Table 8).

†Hybodontoidea Owen, 1846. The Kem Kem hybodontoids, represented by isolated teeth and fin spines, appear to be attributable to three genera, *Bahariyodon bartheli*, *Distobatus nutiae*, and *Tribodus* sp. (Dutheil 2001), and two indeterminate acrodontids (Sereno et al. 1996, Dutheil 1999a, Dutheil et al. 2001, Table 8). The genus *Hybodus* has yet to be reliably reported among isolated teeth in Kem Kem sediments. Diagnostic features of the genus and species reside in its acuminate, multicuspid teeth and cranial features (Maisey 1987); its fin spines thus far have not proven to be diagnostic.

Isolated tooth and fin spine specimens, in addition, cannot be paired with confidence. Two fin spine morphotypes occur in Kem Kem sediments, one with longitudinal striations and the other with tubercles (Fig. 55A–D). Longitudinal striations are known on the fin spines in *Lissodus* and *Tribodus* as well as in *Hybodus*, whereas fin spines with tubercules are reported in *Asteracanthus*. The partial fin spines collected at Aferdou N'Chaft, Boumerade, and Gara Sbaa may pertain to *Tribodus* or Acrodontidae indet., but this remains to be substantiated on the basis of association.

Table 8. The vertebrate assemblage recorded to date in the Kem Kem Group.

Elasmobranchii Bonaparte, 1838		
†Hybodontoidea Owen, 1846		
		†*Asteracanthus aegyptiacus* Stromer, 1927
		†*Bahariyodon* (*Lissodus*) *bartheli* (Werner, 1989)
		†*Distobatus nutiae* Werner, 1989
		†*Tribodus* Brito & Ferreira, 1989, sp. indet.
		†Acrodontidae Casier, 1959, gen. et sp. indet.
Neoselachii Compagno, 1977		
	Galea Shirai, 1996	
		†*Haimirichia amonensis* (Cappetta & Case, 1975)
		†Cretoxyrhinidae Glickman, 1958, gen. et sp. indet.
		†*Cenocarcharias tenuiplicatus* Cappetta & Case, 1975
Batoidea Compagno, 1973		
		†*Onchopristis numidus* (Haug, 1905)
		†*Marckgrafia lybica* Weiler, 1935
Actinopterygii Klein, 1885		
		†*Diplospondichthys moreaui* Filleul & Dutheil, 2008
Cladistia Cope, 1871		
		†*Bartschichthys* Gayet & Meunier, 1996, sp. indet.
		†*Sudania* Werner & Gayet, 1997, sp. indet.
		†*Serenoichthys kemkemensis* Dutheil, 1999b
		†*Bawitius* Grandstaff, et al. 2012, sp. indet.
Ginglymodi Cope, 1872		
†Lepisosteiformes Hay, 1929		
		† *Adrianaichthys pankowskii* (Forey et al., 2011)
	Lepisosteidae Cuvier, 1825, gen. et sp. indet.	
		†*Oniichthys falipoui* Cavin & Brito, 2001
		†*Obaichthys africanus* Grande, 2010
		†*Dentilepisosteus kemkemensis* Grande, 2010
Holostei Müller, 1844 (emended by Grande, 2010)		
	Amiiformes Hay, 1929	
		†*Calamopleurus africanus* Forey & Grande, 1998
Teleostei Müller, 1846		
	†Tselfatiiformes Nelson, 1994	
		†*Concavotectum moroccensis* Cavin & Forey, 2008
	Ichthyodectiformes Bardack & Sprinkle, 1969	
		†*Aidachar* (*Cladocyclus*) *pankowskii* Forey & Cavin, 2007
	Osteoglossomorpha Greenwood et al., 1966	
		†*Palaeonotopterus greenwoodi* Forey 1997
		†Notopteridae Bleeker, 1959, gen. et sp. indet.
	Acanthomorpha Rosen, 1973	
		†*Spinocaudichthys oumtkoutensis* Filleul & Dutheil, 2001
	Clupeomorpha Greenwood et al., 1966	
		†*Diplomystus* Cope, 1877, sp. indet.
	†*Triplomystus* Forey, Yi, Patterson, & Davis, 2003, sp. indet.	
	Characiformes Regan, 1911, gen. et sp. indet.	
Sarcopterygii Romer, 1955		
Actinistia Cope, 1871		
	†Mawsoniidae Schultze, 1993	
		†*Axelrodichthys lavocati* Tabaste, 1963
Dipnoi Müller, 1846		
	Ceratodontidae Gill, 1872	
		†*Ceratodus humei* Priem, 1914
		†*Neoceratodus africanus* (Haug, 1905)

†*Arganodus tiguidensis* (Tabaste, 1963)
Amphibia Gray, 1825
Caudata Scopoli, 1777
Sirenidae Gray, 1825
†*Kababisha* Evans et al., 1996, sp. indet.
Anura Fischer von Waldheim, 1813, gen. et sp. indet.
Pipidae Gray, 1825
†*Oumtkoutia anae* Rage & Dutheil, 2008
Testudines Batsch, 1788
Pleurodira Cope, 1865
†Araripemydidae Price, 1973, gen. et sp. indet.
†Euraxemydidae Gaffney et al., 2006
†*Dirqadim schaefferi* Gaffney et al., 2006
†Podocnemidoidea Cope, 1868
†*Hamadachelys escuilliei* Tong & Buffetaut, 1996
†*Galianemys whitei* Gaffney et al., 2002
†*Galianemys emringeri* Gaffney et al., 2002
Squamata Oppel, 1811
Ophidia Brongniart, 1800
†*Norisophis begaa* Klein et al., 2017
†Lapparentophiidae Hoffstetter, 1959
†*Lapparentophis ragei* Vullo, 2019
†Simoliophiidae Nopcsa, 1925
†*Simoliophis* cf. *libycus* Nessov et al., 1998
†Nigerophiidae Rage, 1975, gen. et sp. indet.
†Madtsoiidae Hoffstetter, 1961, gen. et sp. indet.
Iguania Cope, 1864
†*Jeddaherdan aleadonta* Apesteguía et al., 2016
†Borioteiioidea Nydam et al., 2007
†*Bicuspidon hogreli* Vullo & Rage, 2018
Crocodyliformes Hay, 1930
†Peirosauridae Gasparini, 1982
†*Hamadasuchus rebouli* Buffetaut, 1994
†Notosuchia Gasparini, 1971
†*Araripesuchus rattoides* Sereno & Larsson, 2009
†Candidodontidae Carvalho et al., 2004
†*Lavocatchampsa sigogneaurussellae* Martin and de Lapparent de Broin, 2016
†Sphagesauridae Kuhn, 1968, gen. et sp. indet.
Neosuchia
†Stomatosuchidae Stromer, 1925
†*Laganosuchus maghrebensis* Sereno & Larsson, 2009
†Aegyptosuchidae Kuhn,1936
†*Aegisuchus witmeri* Holliday & Gardner, 2012
†Pholidosauridae Zittel & Eastman, 1902
†*Elosuchus cherifiensis* de Lapparent de Broin, 2002
†Pterosauria Kaup, 1834
†Ornithocheiridae Seeley, 1870
†*Siroccopteryx moroccensis* Mader & Kellner, 1999
†*Coloborhynchus fluviferox* Jacobs et al., 2019
†*Ornithocheirus* Seeley, 1869, sp. indet.
†*Anhanguera* Campos & Kellner, 1985, sp. indet.
†Azhdarchidae Nessov, 1984
†*Alanqa saharica* Ibrahim et al., 2010
†*Xericeps curvirostris* Martill et al., 2018
†Tapejaridae Kellner, 1989, gen. et sp. indet.
†*Afrotapejara zouhrii* Martill et al., 2020

?†Chaoyangopteridae
†*Apatorhamphus gyrostega* McPhee et a., 2020
Dinosauria Owen, 1842
†Ornithischia Seeley, 1888, gen. et sp. indet.
†Sauropoda Marsh, 1878
†Rebbachisauridae Bonaparte, 1997
†*Rebbachisaurus garasbae* Lavocat, 1954b
†Titanosauria Bonaparte & Coria, 1993, gen. et sp. indet.
Theropoda Marsh, 1881, gen. et sp. indet.
†Noasauridae Bonaparte & Powell, 1980, gen. et sp. indet.
†Abelisauridae Bonaparte & Novas, 1985, gen. et sp. indet.
†*Deltadromeus agilis* Sereno et al., 1996
†Carcharodontosauridae Stromer, 1931
†*Carcharodontosaurus saharicus* Stromer, 1931
†Spinosauridae Stromer, 1915
†*Spinosaurus aegyptiacus* Stomer, 1915
†Dromaeosauridae Colbert & Russell, 1969, gen. et sp. indet.

Rare teeth of *Bahariyodon bartheli* (Werner 1989, Duffin 2001) have been recovered from Douira and Dar el Karib localities in the Douira Formation. Teeth of *Distobatus nutiae*, previously known only from the Bahariya Formation (Werner 1989) and from the Cenomanian of the Draa Ubari in Libya (Nessov et al. 1998, Rage and Cappetta 2002), were also collected from these two localities. A rare tooth morphotype, here attributed to *Tribodus* sp. (Table 8), is rectangular with vertical ridges, resembling the Brazilian species *Tribodus lima* (Brito and Ferreira 1989) and Egyptian species *Tribodus kuehnei* from the Bahariya Formation (Werner 1989).

Neoselachii Compagno, 1977

Galea, Wagler, 1851. The pond locality Oum Tkout has yielded isolated teeth of lamniform elasmobranchs (Dutheil 1999a). The remaining diverse ichthyofauna from this locality is freshwater (Dutheil 1999a). One tooth (Fig. 55E, F) pertains to the mackerel shark *Haimirichia amonensis* and another one to the cretoxyrhinid *Cenocarcharias tenuiplicatus* (Cappetta and Case 1975).

Batoidea Compagno, 1973. Rostral teeth of the large-bodied, sclerorhynchid batoid, *Onchopristis numidus* (Haug 1905), are the most abundant vertebrate element in Kem Kem sediments (Martill and Ibrahim 2012). The teeth of the rostrum and centra are occasionally found in place in both formations (Fig. 54A-C). New cranial and axial material of this taxon include a large rostrum (Fig. 54D-G), an articulated series of more than 50 vertebrae (Fig. 56), and an exceptional new specimen under study that preserves portions of the rostrum and skull in association with vertebral centra (Dutheil and Brito 2009).

The rostrum was recovered in two pieces from the Valley near Boumerade locality (Fig. 9, locality 7) near a partial quadrate that may pertain to *Spinosaurus* (Fig. 38A). With both pieces of the tapering rostrum positioned as they would be in life, it measures more than 40 cm in length (Fig. 54D, E). Anteriorly it tapers in width, and

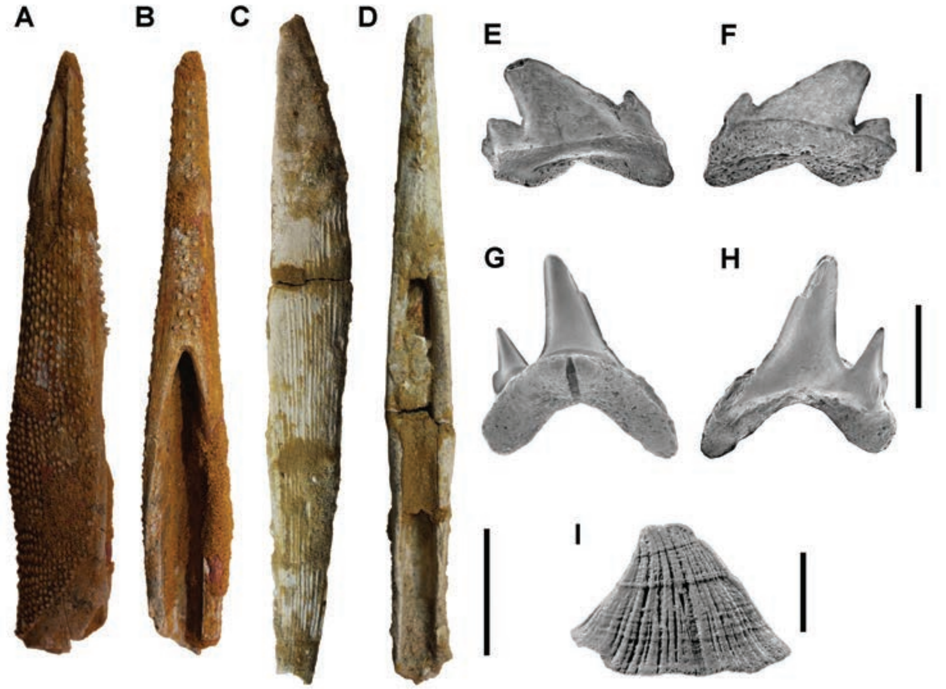

Figure 55. Elasmobranch fossils from Morocco and Egypt. Hybodontoidea fin spine with tubercles (FSAC-KK 943), in (**A**) lateral and (**B**) posterior view. Hybodontoidea fin spine (FSAC-KK 944), ornamented with striations in (**C**) lateral and (**D**) posterior view. Lamniform tooth (Poi-SGM 52) referred to *Haimirichia amonensis* in (**E**) labial and (**F**) lingual view. Lamniform tooth (Poi-SGM 53) referred to *Cenocarcharias* cf. *tenuiplicatus* in (**G**) labial and (**H**) lingual views. **I** Rostral tooth of *Peyeria libyca* (Poi SGM 61). Scale bars equal 3 cm in **A-D**, 3 mm in **E** and **F**, 2 mm in **G** and **H**, 1 cm in **I**.

ventrally it is marked by a sharp-edged trough approximately 5 mm deep and inset from the lateral margin (Fig. 54). A linear, straw-like texture is present on both dorsal and ventral surfaces of the calcified rostrum.

The calcified disc-shaped, biconvex vertebrae have concave sides and decrease in diameter toward the distal end of the series (Fig. 56), which places the series in the posterior portion of the axial column. The articulated series measures more than 80 cm and pertains to an individual that was probably several meters in length.

Another specimen of *Onchopristis numidus*, which was collected commercially from Kem Kem sediments, preserves portions of the cranium and several anterior vertebrae (Dutheil and Brito 2009). It provides direct evidence of association between the rostrum and rostal teeth of *O. numidus* and its oral teeth, oral osteoderms and vertebral centra. This specimen confirms previous suggestions by Tabaste (1963) that the common discoid centra in Kem Kem sediments initially described as *Platyspondylus foureaui* (Haug, 1905), pertain to this species.

Two additional Kem Kem batoids have been found in screen-washed sediment at Douira in the Douira Formation (Dutheil 2001, Table 8). *Marckgrafia lybica*, initially

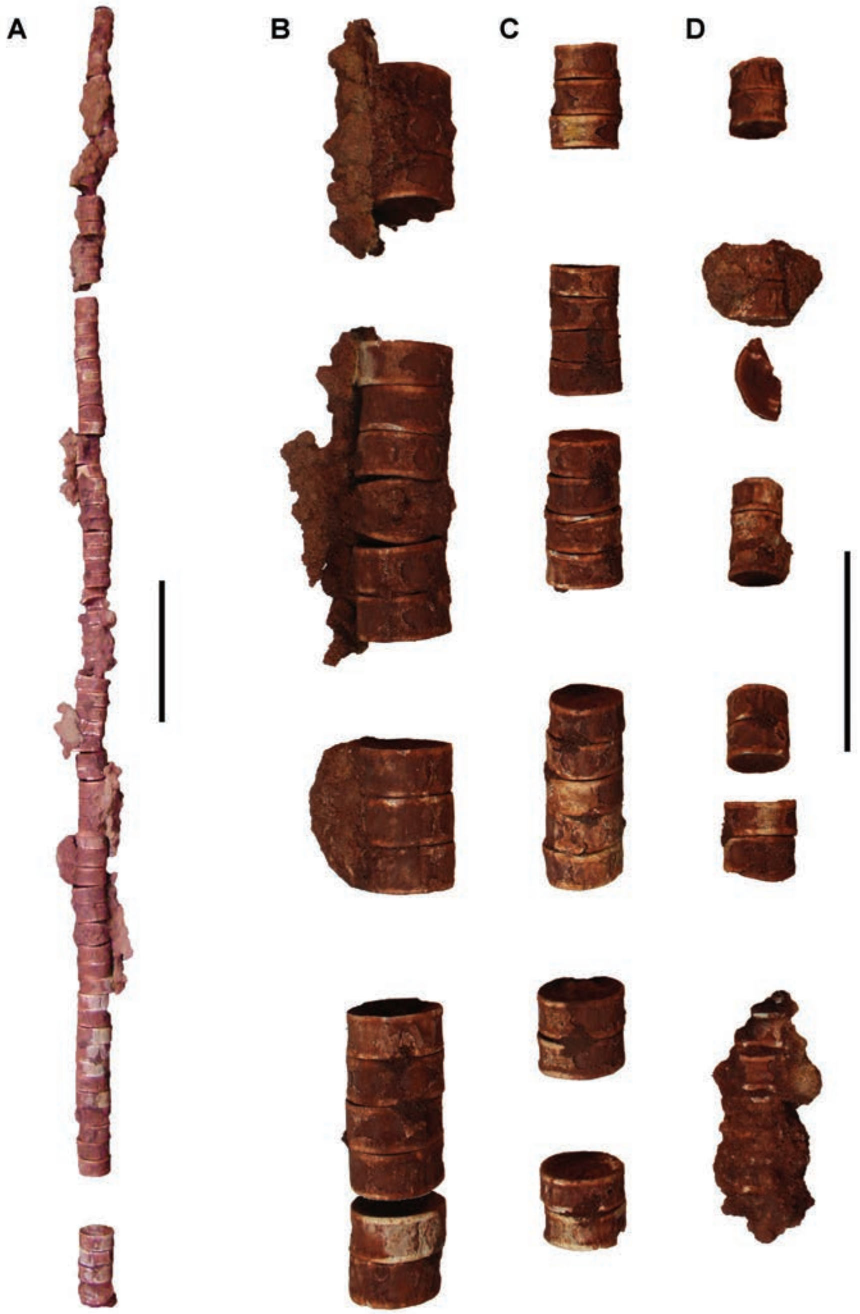

Figure 56. Associated elasmobranch vertebrae likely referable to *Onchopristis numidus* from the Kem Kem Group. **A** Reconstructed vertebral series. **B-D** Select vertebrae with sedimentary matrix. Scale bars equal 10 cm in **A**, 5 cm in **B-D**.

described from the Bahariya Formation of Egypt (Weiler 1935, Table 8) is represented by 13 teeth. The pristid *Peyeria libyca* was initially described from the Bahariya Formation of Egypt (Weiler 1935) and is represented by three teeth, although new evidence suggests it may comprise non-rostral denticles of *Onchopristis numidus* (Sternes and Shimada 2019). Two sections of caudal centra recovered from the pond locality of Oum Tkout may pertain to batoids on the basis of their numerical dominance among Kem Kem elasmobranchs.

Actinopterygii Klein, 1885

Actinopterygii are usually recovered as isolated bones except in rare instances and at the pond locality Oum Tkout, which has yielded nearly complete skeletons. Actinopterygii include basal clades, such as polypterids (Cladistia), lepisosteids and seminotiformes (Ginglymodi), Amiiformes, and a range of teleosts (Table 8). The review below adds new information to previous summaries of the Kem Kem ichthyofauna (Dutheil 1999a, Cavin et al. 2015).

Cladistia Cope, 1871. Cladistians are widespread in Africa (Stromer 1936, Stewart 1994, Gayet and Werner 1997) and have also been reported from the Americas (Gayet and Meunier 1991). Isolated ganoid scales are present in both formations of the Kem Kem Group, suggesting that polypterids may have been a common element of the ichthyofauna.

Four genera have been recorded. At the pond locality Oum Tkout in the Douira Formation, several articulated skeletons have been recovered of the small cladistian *Serenoichthys kemkemensis* (Dutheil 1999b, Fig. 57). At the same locality, isolated pinnulae (the spine that supports each dorsal finlet) are referable to *Bartschichthys* sp. (Dutheil 1999a), based on similarities to *Bartschichthys arnouldi* from similar age rocks in Niger (Gayet and Meunier 1996). Dutheil (1999a) referred another isolated pinnula from the same horizon to *Sudania* sp., as it closely resembled specimens from the Cenomanian Wadi Milk Formation in Sudan (Werner and Gayet 1997).

Large jaw bones with teeth (Cavin et al. 2015) and scales (Meunier et al. 2016) from the Kem Kem Group were recently referred to the Egyptian cladistian *Bawitius*. An isolated premaxilla (Fig. 58) also may pertain to this large-bodied genus. The bone has a pitted surface texture. The teeth are large, hollow and ankylosed to the bone. These features closely resemble a better-preserved partial skull from beds of Cenomanian age in Niger (Fig. 59). The Niger specimen is associated with scales, allowing positive referral to Cladistia.

Ginglymodi Cope, 1872, Semionotiformes Arambourg & Bertini, 1958. Several authors describe a range of ginglymoid and semionotiform fishes from disarticulated material (Dutheil 1999a, Cavin and Brito 2001, Grande 2010, Cavin et al. 2015). The moderate-sized *Oniichthys falipoui* and *Obaichthys africanus* are known

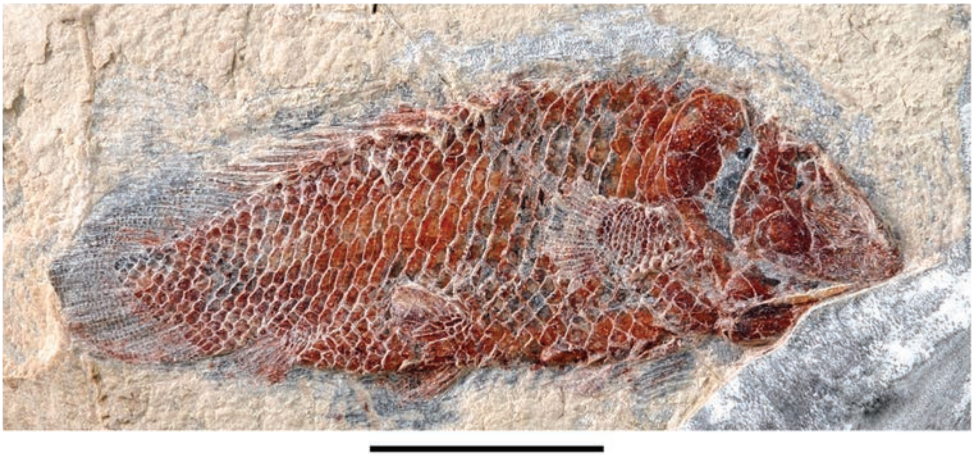

Figure 57. *Serenoichthys kemkemensis* Dutheil, 1999 from the Douira Formation. Scale bar equals 1 cm.

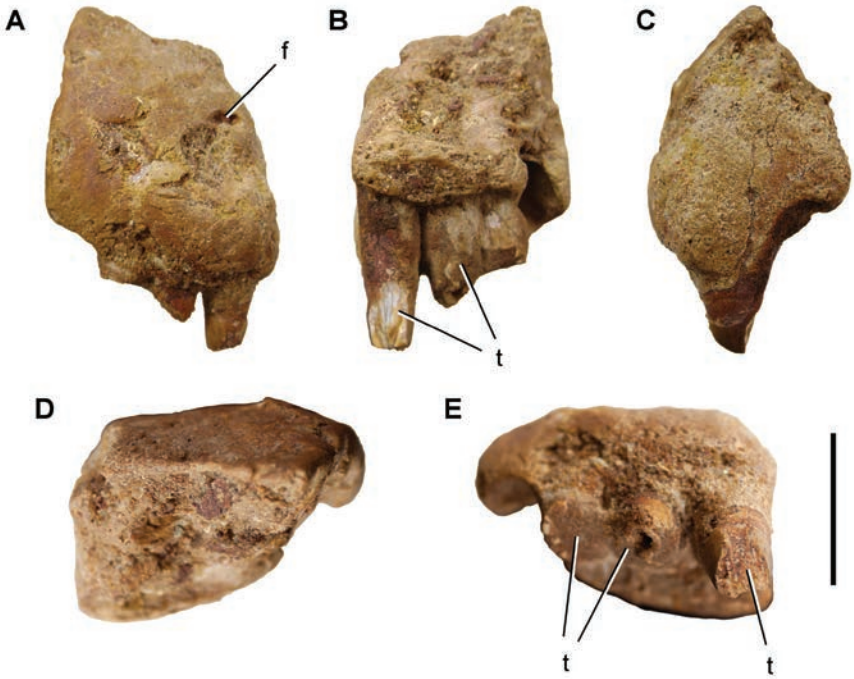

Figure 58. Large polypterid premaxilla (FSAC-KK 209) from the Kem Kem Group. In (**A**) anterior, (**B**) posterior, (**C**) lateral, (**D**) dorsal and (**E**) ventral view. Scale bar equals 3 cm. Abbreviations: **f** foramen **t** tooth.

from partial skeletons, and *Dentilepisosteus kemkemensis* is represented by scales (Cavin et al. 2015). Other isolated scales that likely pertain to this group measure more than 50 mm in length and are indicative of large-bodied species (Fig. 60). Pycnodonts have also been identified among disarticulated remains from the Kem Kem Group.

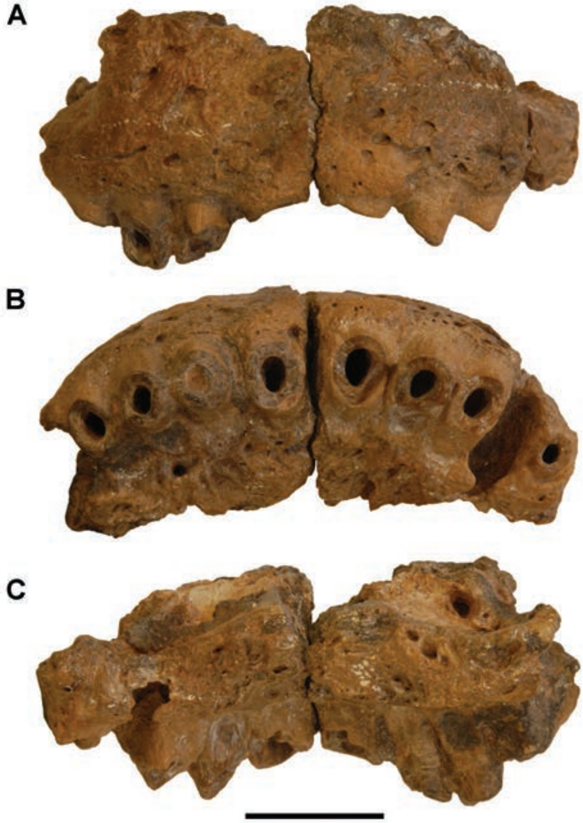

Figure 59. Large polypterid (MNBH IGU23) from the Cenomanian of Niger. Associated right and left premaxillae in (**A**) anterior, (**B**) ventral and (**C**) lingual view. Scale bar equals 3 cm.

Amiiformes Hay, 1929. Isolated teeth and several dentary fragments (Fig. 61A–D) pertain to amiids, which have been found in both formations of the Kem Kem Group. A partial amiiform skull, described as *Calamopleurus africanus* (Forey and Grande 1998), differs in skull proportions but otherwise is similar to the Brazilian species *C. cylindricus* (Cavin et al. 2015).

A curved dentary from Aferdou N'Chaft has at least 20 alveoli for small triangular teeth and lacks interdental plates (Fig. 62E–I). The texture and form of the dentary resembles that in amiids (Martinelli et al. 2013).

Teleostei Müller, 1846. More than a dozen genera of teleost fishes have been described from the Kem Kem Group (Table 8). Several are known from partial or complete skeletons from Oum Tkout. The elongate *Diplospondichthys moreaui* (Fig. 63) has an unusual combination of features that has left its position uncertain within Teleostei (Filleul and Dutheil 2004). The elongate freshwater acanthomorph *Spinocaudichthys oumtkoutensis* (Fig. 64) has also been recorded at the same locality (Filleul and Dutheil 2004).

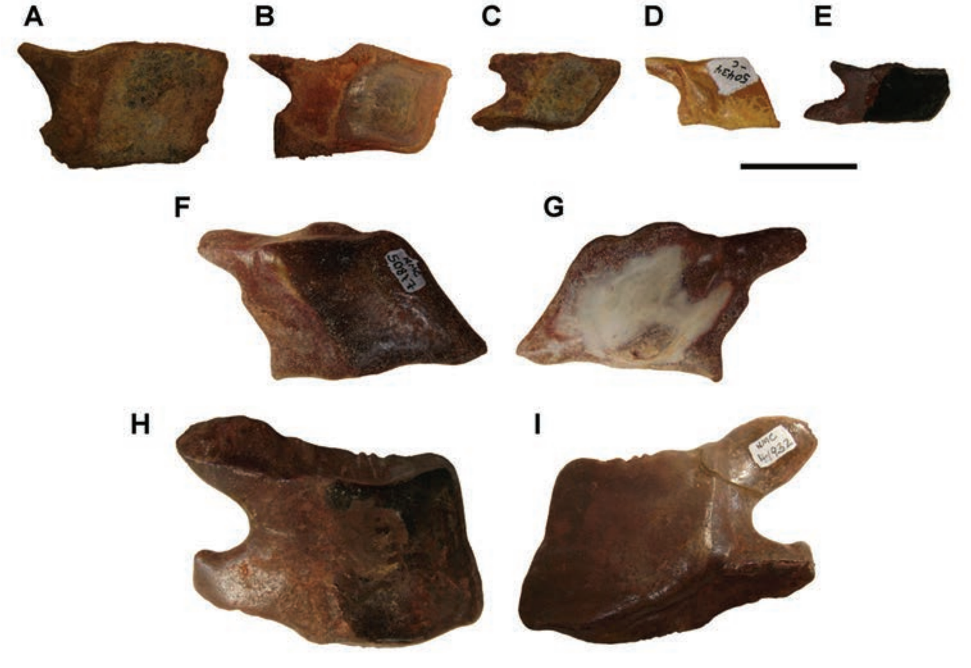

Figure 60. Scales of a large holostean from the Kem Kem Group. **A** FSAC-KK 530 (Gara Sbaa) **B** NMC 50434-A **C** FSAC-KK 531 (Gara Sbaa) **D** NMC 50434-C **E** NMC 50434-B **F** Lateral view of NMC 50817 **G** Medial view of NMC 50817 **H** Lateral view of NMC 41932 **I** Medial view of NMC 41932 (one of the largest known teleost scales from the Kem Kem Group). Scale bar equals 3 cm.

Forey and Cavin (2007) described a well-preserved ichthyodectiform braincase from an unknown locality in eastern Morocco as *Cladocyclus pankowskii*, which later was placed in the genus *Aidachar* (Cavin et al. 2015). A well-preserved dentary (Fig. 62A-C) is referable to *A. pankowskii* and is also similar to the ichthyodectine *Xiphactinus* (Leidy 1870, Schwimmer et al. 1997, Fig. 62D). Osteoglossiform and notopterid remains including skull fragments were assigned to *Palaeonopterus greenwoodi* (Forey 1997, Taverne 2000, Cavin and Forey 2001a). The median lingual dental plate of this species is composed of several superimposed layers of adjacent teeth (Meunier et al. 2013), which were previously described in error as *Pletodus* sp. (Dutheil 1999a). *Erfoudichthys rosae* is a small-bodied teleost of unknown affinity, known from an isolated skull (Cavin et al. 2015). Previously it was thought to be a gonorynchiform (Pittet et al. 2010).

Sarcopterygii Romer, 1955

Actinistia Cope, 1871. Isolated cranial bones pertaining to large-bodied coelacanths are present in both formations of the Kem Kem Group (Figs 65, 66). Numerous isolated cranial bones and scales were collected at Boumerade and Gara Sbaa in the Gara

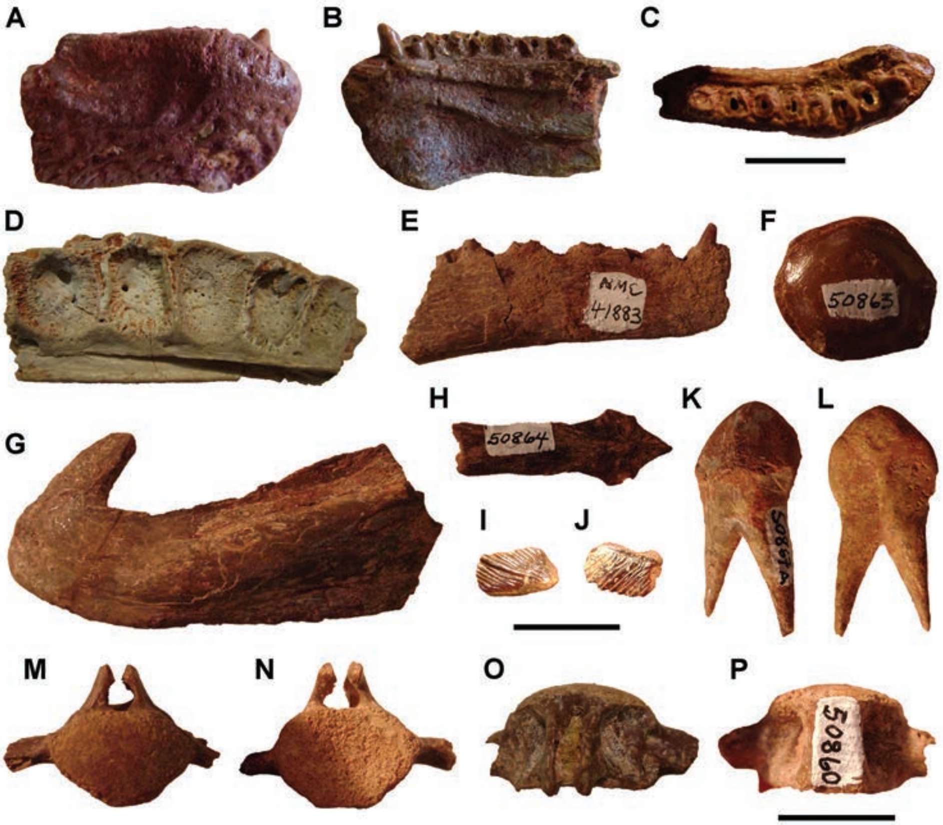

Figure 61. Actinopterygian remains from the Kem Kem Group. **A-C** Possible amiid dentary fragment (MDM 02) in right lateral, medial and dorsal (occlusal) views **D** Possible amiid dentary fragment (MSNM V 6417) **E** Dentary of an unidentified teleost (NMC 41883) **F** Possible notopterid dental plate (NMC 50863) **G** Probable teleost bone (NMC 41900) **H** Possible teleost parasphenoid (NMC 50864) **I**, **J** Possible *Obaichthys africanus* (Cavion et al. 2015) scales (NMC 50437) **K**, **L** Possible seminotiform scale (NMC 50867A) **M-P** Possible lepisosteid or obaichthyid vertebra in anterior, posterior, dorsal and ventral view. Scale bars equal 1 cm in **A-D**, 2 cm in **E-L**, 2 cm in **M-P**.

Sbaa Formation. Although the size and ornamentation of dermal cranial bones are easily recognized, their generic and specific assignment remains uncertain.

The genera *Mawsonia* and *Axelrodichthys* were originally described from South America on the basis of complete specimens in nodules of late Early Cretaceous age (Maisey 1986). In contrast, the African material largely consists of isolated bones from Morocco and Algeria (Tabaste 1963, Wenz 1981, Cavin and Forey 2004). Cavin et al. (2015) suggested that, in addition to *Mawsonia lavocati*, a second large coelacanth may be present within the Kem Kem Group, assigning an isolated cranial bone to the South American genus *Axelrodichthys*. More recently, after a review of actinistians assigned to the genera *Mawsonia* (Woodward 1907) and *Axelrodichthys* (Maisey 1986) *Mawsonia lavocati*

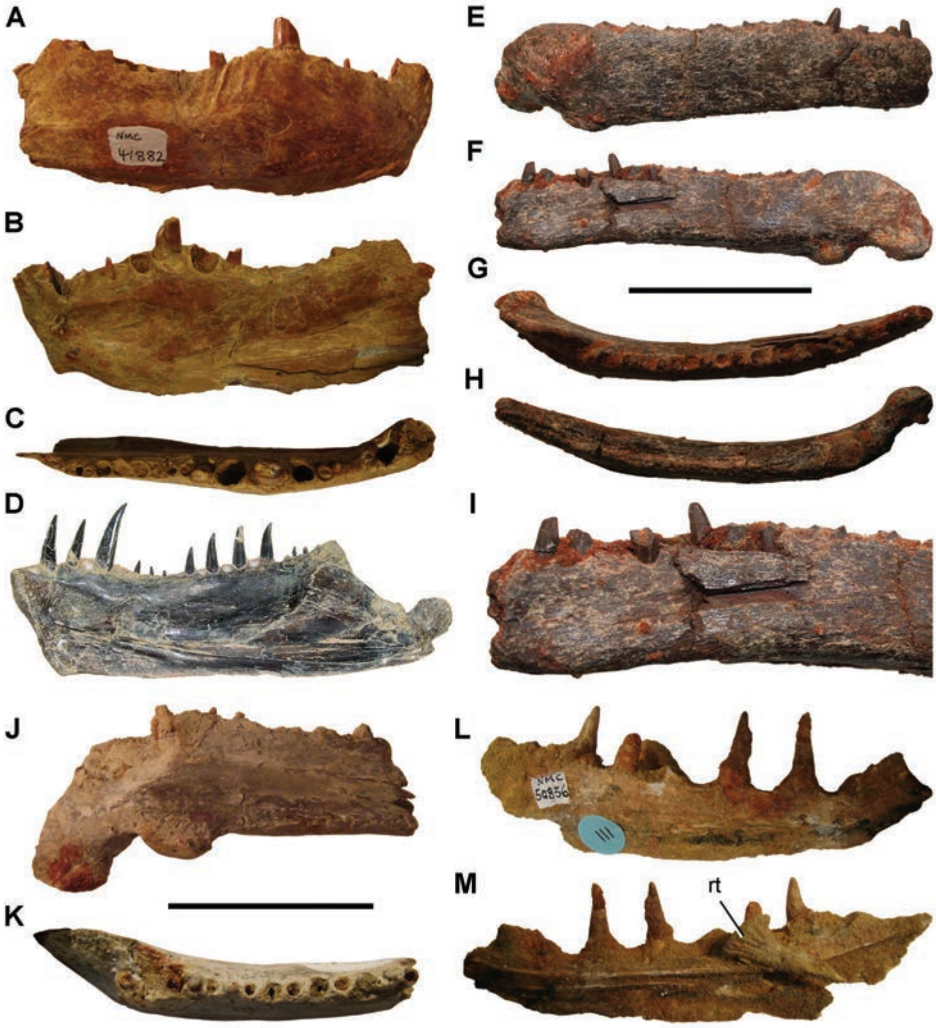

Figure 62. Isolated teleost dentaries from the Kem Kem Group. **A-C** Partial dentary of an ichthyodectiform with affinities to *Xiphactinus* and *Cladocyclus* (NMC 41882) in right lateral, medial and dorsal views **D** Right dentary of *Xiphactinus audax* (FHSM VP-2973) from the Late Cretaceous of the USA in medial view (courtesy of Mike Everhart) **E-I** Isolated dentary (FSAC-KK 906) of possible amiid in right lateral, medial, dorsal (occlusal) ventral, and medial (close-up) views **J-K** Dentary (NMC 41884) in ?right lateral and dorsal (occlusal) view. **L-M** Dentary of an unidentified predatory teleost with rostral tooth of *Onchopristis numidus* (NMC 50836) in lateral and medial view. Scale bars equal 5 cm in **A-C, E-H**, 10 cm in **D**, 3 cm in **I-K**, 5 cm in **L** and **M**. Abbreviation: **rt** rostral tooth.

was included in *Axelrodichthys* as *Axelrodichthys lavocati* by Carnier Fragoso et al. (2019). Yabumoto and Uyeno (2005) described a partial skull with lower jaws from an uncertain locality within the Kem Kem Group. They also provide a revision of the species that serves as a guide for assignment of isolated material.

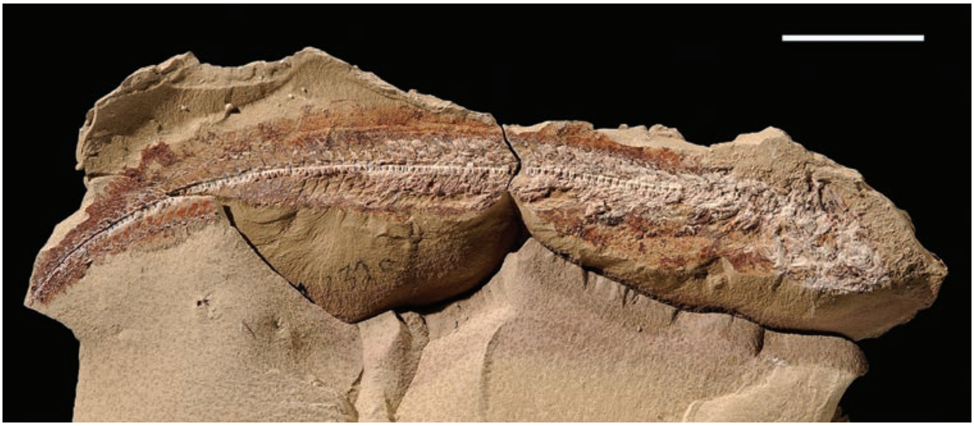

Figure 63. *Diplospondichthys moreaui* Filleul and Dutheil, 2004 from the Kem Kem Group. Scale bar equals 2 cm.

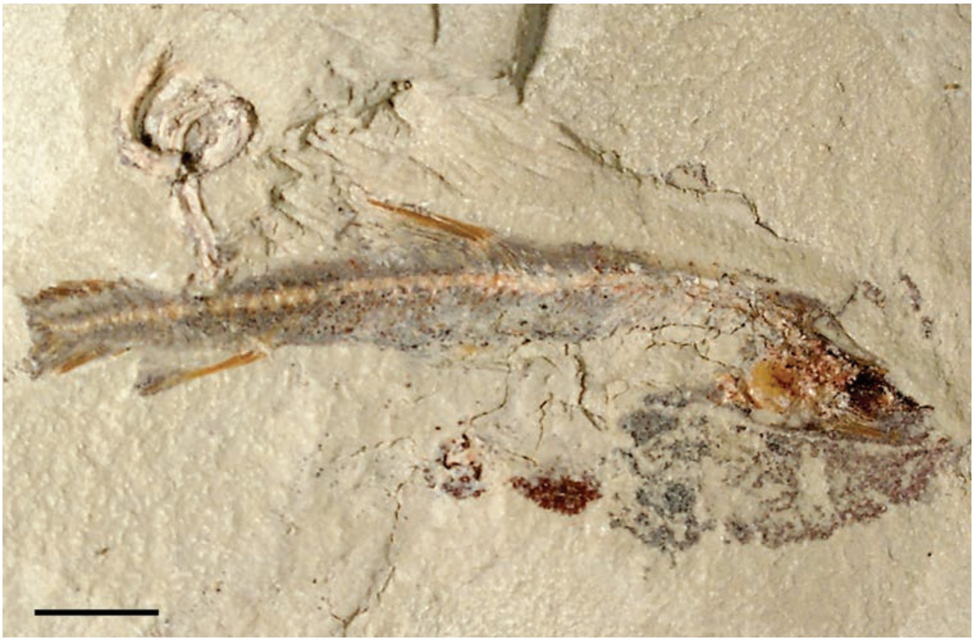

Figure 64. *Spinocaudichthys oumtkoutensis* Filleul and Dutheil, 2002 from the Kem Kem Group. Scale bar equals 5 mm.

Measuring approximately 30 cm long, the skull confirms the large size of the genus *Axelrodichthys* in lake and river deposits on Africa and South America (Carvalho and Maisey 2008, Carnier Fragoso et al. 2019). *Axelrodichthys lavocati* may have grown to a body length in excess of 4 m.

Dipnoi Müller, 1844. Lungfish tooth plates are common in both formations of the Kem Kem Group (Fig. 67). They vary considerably in size and ornamentation with distinctive morphological differences.

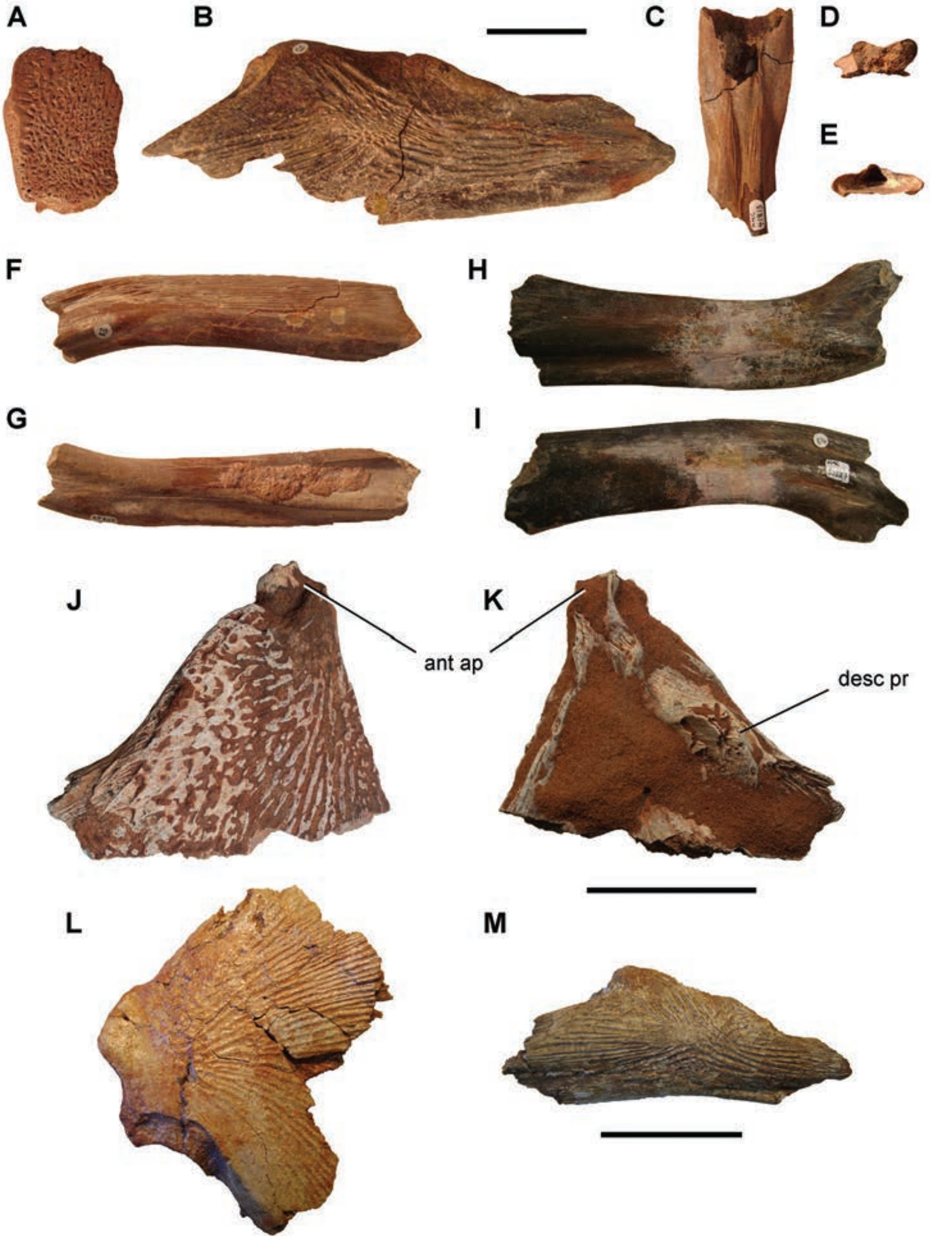

Figure 65. Isolated elements referable to *Axelrodichthys lavocati* or a closely related form from the Kem Kem Group. **A** Isolated pterotic (NMC 41877) **B** Right angular (NMC 50816). Parasphenoid (NMC 41813) in (**C**) dorsal, (**D**) anterior and (**E**) posterior view. Visceral arch element (NMC 50828) in (**F**) lateral and (**G**) medial view. Visceral arch element (NMC 50827) in (**H**) lateral and (**I**) medial view. Isolated postparietal of (FSAC-KK 157), collected at locality 5 (Figure 9) in (**J**) dorsal and (**K**) ventral views. **L** Isolated operculum (MNHN-MRS 926). **M** Left angular (MNHN-MRS 78), part of type material of *A. lavocati* (Tabaste 1963). Scale bars equal 5 cm in **A–I**, 5 cm in **J** and **K**, 10 cm in **L** and **M**. Abbreviations: **ant ap** anterior apophysis **desc pr** descending process of postparietal.

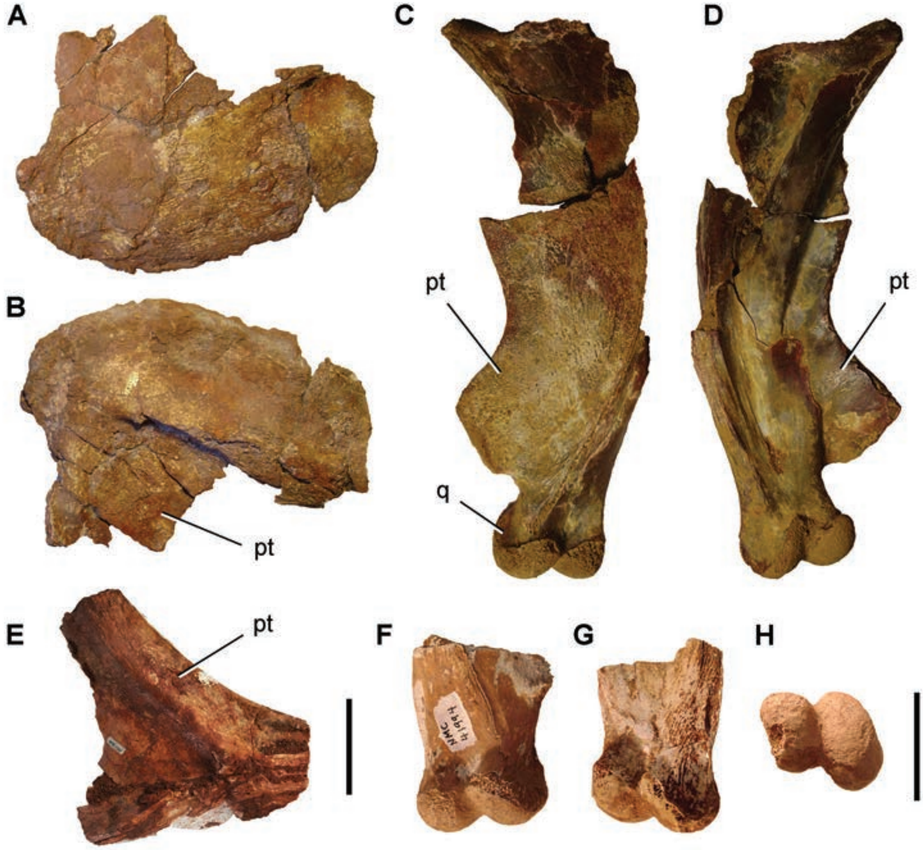

Figure 66. Large specimens from the Kem Kem Group likely referable to *Axelrodichthys*. Possible fragment of palatoquadrate region preserving a large part of the pterygoid (MNHN-MRS 926) in (**A**) lateral and (**B**) medial view. Large quadrate with a small section of the pterygoid (MPDM 14) in (**C**) lateral and (**D**) medial view. **E** Partial palate of *Axelrodichthys* (MNHN-MRS 1761) in lateral view. Quadrate (NMC 41994) in (**F**) lateral, (**G**) medial and (**H**) ventral view. Scale bars equal 10 cm in **A**, **B** and **E**, and 3 cm in **C**, **D** and **F-H**. Abbreviations: **pt** pterygoid **q** quadrate.

Ceratodontidae Gill, 1872. The generic taxonomy of fossil lungfish, which is based almost exclusively on toothplates, has been unsettled and species have been variously assigned to the extinct genus *Ceratodus* or to the living Australian genus *Protopterus* and living African genus *Neoceratodus*. The most recent assessment (Cavin et al. 2015) attributes Kem Kem lungfish to three ceratodontid genera, *Ceratodus*, *Neoceratodus*, and *Arganodus* (Table 8). Toothplates with deeply incised ridges (Fig. 67A) are the most common and were initially identified as *Ceratodus africanus* (Haug 1905, Tabaste 1963, Martin 1984). More recently, these are referred to *Neoceratodus africanus* (Martin 1982, Werner 1995, Cavin et al. 2015), which has been reported across all of northern Africa in similar age beds (Haug 1905, Peyer 1925, Werner 1995, Tabaste 1963, Schlüter and Schwarzhans 1978, Schaal 1984, Cavin

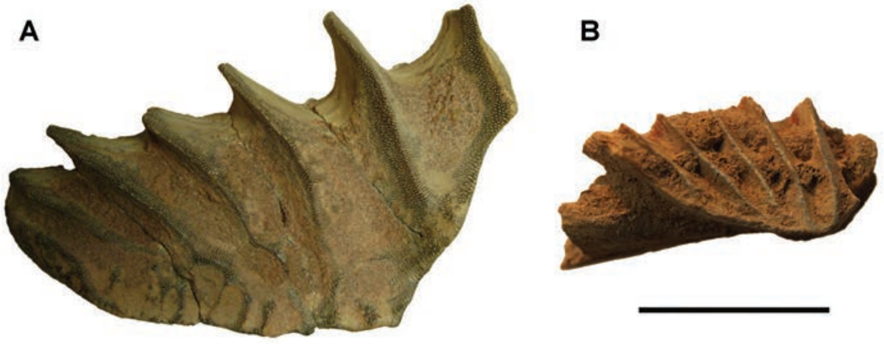

Figure 67. Size variation in lungfish tooth plates from the Kem Kem Group. **A** UCRC PV62, likely *Neoceratodus africanus* **B** FSAC-KK 2735. Note that some size differences can be even greater in exceptional specimens. Scale bar equals 3 cm.

et al. 2010). In the Kem Kem Group, the toothplates of *N. africanus* can measure more than10 cm.

Tabaste (1963) described small toothplates with low ornamentation and only four low ridges as *Ceratodus humei*. These have also been found across northern Africa in similar age beds (Haug 1905, Werner 1993, Martin 1984). Martin (1984) placed this species in the extant genus *Protopterus*, but later it was returned to the genus *Ceratodus* (Churcher and De Iuliis 2001). Recently Cavin et al. (2015) referred small toothplates with a characteristic radiating pattern of ridges to *Arganodus tiguidensis*, a species described originally from Algeria (Tabaste 1963) and later reported in Niger (Broin et al. 1974) and Brazil (Candeiro et al. 2011).

Amphibia Gray, 1825

Caudata Scopoli, 1777

Sirenidae Gray, 1825. Several localities in the finer-grained Douira Formation have yielded two braincases and 38 vertebrae pertaining to salamanders (Rage and Dutheil 2008). Most of the vertebrae come from the pond locality Oum Tkout, whereas the braincases were found at Taouz and Talidat localities. The occipital condyle of each partial braincase is transversely broad (Fig. 68A, B), which resembles the condition in the Sudanese genus *Kababisha* (Evans et al. 1996). The isolated vertebrae, which are tentatively referred to the same genus, resemble those of *Kababisha* and the South American genus *Noterpeton* (Rage and Dutheil 2008). The keeled centra, which are 2–3 mm in length, are flanked to each side by flange-shaped transverse processes. A foramen opens onto the dorsal surface of the flange. Unlike the Sudanese site, only a single sirenian appears to be present in the Douira Formation.

One trunk or anteriormost caudal vertebra of an indeterminate salamander is known from the Algerian locality Oued Bou Seroual in a level comparable to the

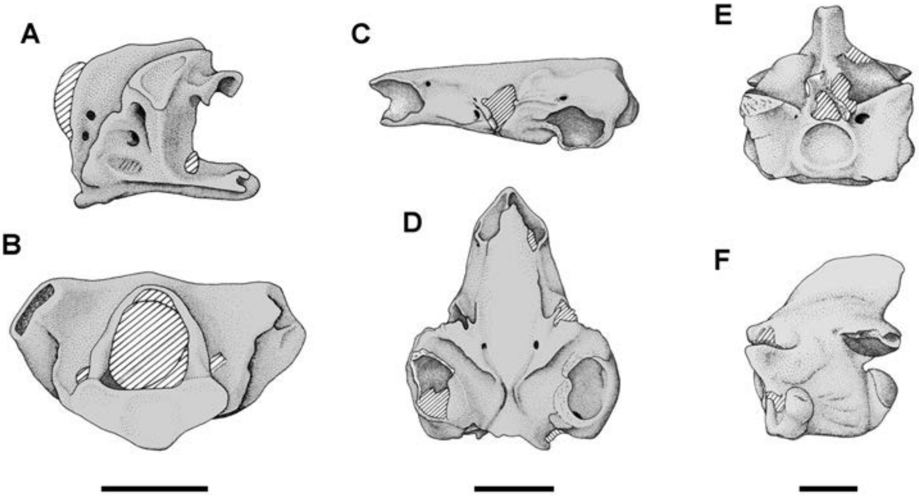

Figure 68. Amphibia and Squamata from the Douira Formation. Cf. *Kababisha* sp. braincase (UCRC PV50) from Oum Tkout in (**A**) lateral and (**B**) posterior view. *Oumtkoutia anae* braincase (UCRC PV63) from Oum Tkout in (**C**) lateral and (**D**) ventral view. *Simoliophis* cf. *S. lybicus* trunk vertebra UCRC PV127) from Douira in (**E**) anterior and (**F**) lateral view. Figures modified from Rage and Dutheil (2008). Scale bars equal 1 mm in **A** and **B**, 2 mm in **C** and **D**, 3 mm in **E** and **F**.

Douira Formation and approximately 25 km distant from Oum Tkout. The morphology of this procoelous vertebra suggests that it pertains to an elongate, snake-like salamander (Alloul et al. 2018).

Anura Fischer von Waldheim, 1813. A partial braincase, jaw fragments, and procoelous vertebrae probably pertain to several species of non-pipid anurans, but the remains are too fragmentary to assign to particular families (Rage and Dutheil 2008).

Pipidae Gray, 1825. The majority of the anuran material collected in the Douira Formation, as in other Gondwanan localities, is referable to the Pipidae (Rage and Dutheil 2008). The holotype for a new genus and species, *Oumtkoutia anae*, was found at the pond locality Oumt Tkout (Fig. 68C, D). The subtriangular cranium that narrows anteriorly and the distinctive vertebral morphology distinguish this species from other pipids (Rage and Dutheil 2008). Other localities (Dar el Karib, Taouz) generated additional isolated bones. In all there are 22 partial braincases, five vertebrae, and seven pelvic fragments.

Testudines Batsch, 1788

Testudines are common among vertebrate fossils in the Kem Kem Group (Gmira 1995). Isolated shell fragments are the most common (Fig. 69) followed by shell pieces (Fig. 70), vertebrae and limb bones (Fig. 71) and, rarely, partial plastron and carapace (Fig. 72) or skull material (Fig. 73). Named Kem Kem Group testudines, thus far, are

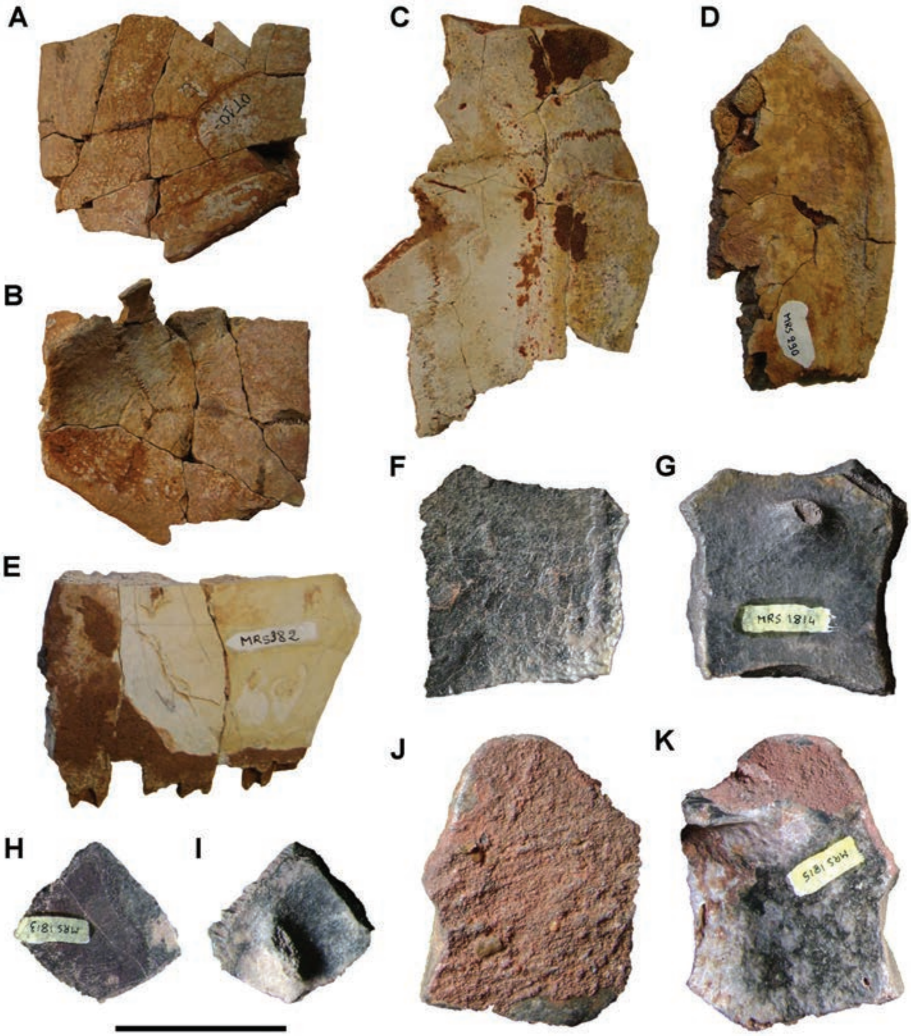

Figure 69. Testudinid shell fragments from the Kem Kem Group. Partial plastron with sutures (UCRC PV165) in (**A**) dorsal and (**B**) ventral view. Large shell fragment (UCRC PV166) with sutures highlighted by red and dark lines (**C**). Rounded and smooth shell fragment (MNHN-MRS 290) (**D**). MNHN-MRS 382 (**E**). Dark-colored carapace elements from Kouah Trick locality (MNHN-MRS 1813-1815) in (**F**, **H**, **J**) dorsal and (**G**, **I**, **K**) ventral view. Scale bar equals 5 cm.

based solely on isolated crania (Fig. 73). These were collected commercially from uncertain localities and horizons (Tong and Buffetaut 1996, Gaffney et al. 2002, 2006).

Cranial material pertains, thus far, exclusively to pleurodires; cryptodires have yet to be reported. Three genera of euraxymydid and podocnemidoid pleurodires were described on the basis of isolated crania (Gaffney et al. 2002, 2006); postcranial remains have yet to be definitively associated with any of the three genera *Dirqadim*, *Hamadachelys*, and *Galianemys*. Araripemydid pleurodires are known only from iso-

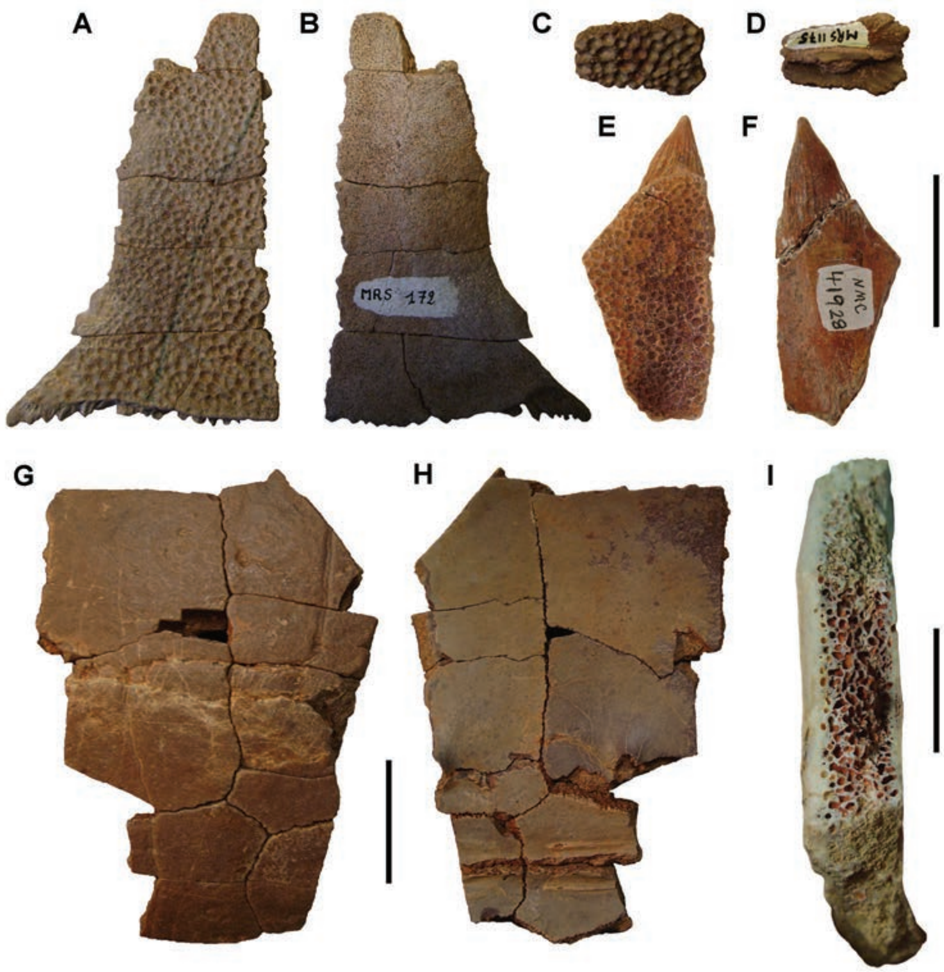

Figure 70. Possible araripemydid and podocnemidinuran shell fragments from the Kem Kem Group. Possible araripemydid plastral element (MNHN-MRS 172) in (**A**) dorsal and (**B**) ventral view. MNHN-MRS 1175 in (**C**) dorsal and (**D**) ventral view. NMC 41928 in (**E**) dorsal and (**F**) ventral view. Partial podocnemidinuran (?*Galianemys*) plastron collected at Iferda N'Ahouar (UCRC PV167) in (**G**) dorsal and (**H**) ventral view. Cross-sectional view of a typical shell fragment showing dense and cancellous bone (**I**). Scale bars equal 3 cm in **A-F**, 3 cm in **G** and **H**, 1 cm in **I**.

lated carapace fragments with pitted texture (Fig. 70A-F); no cranial material has been discovered.

Pleurodira Cope, 1865

Araripemydidae Price, 1973. The flattened, fragile-shelled araripemydids are much better known from slightly older (Aptian-Albian) rocks to the south in Niger (Sereno and ElShafie 2013) and contemporary deposits in Brazil, which were located across

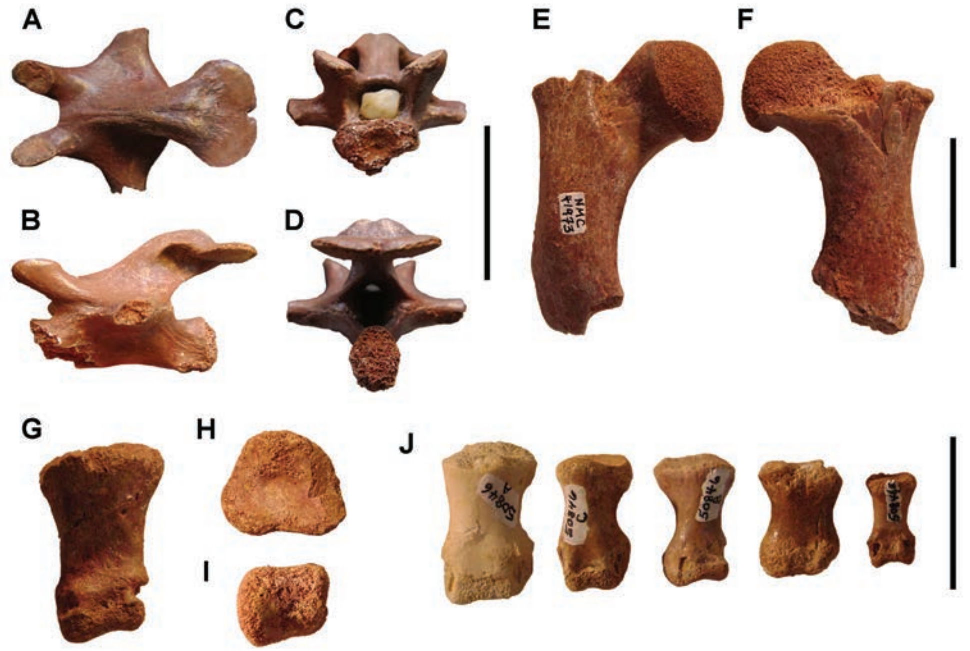

Figure 71. Isolated turtle postcrania from the Kem Kem Group. Pelomedusoides indet., cervical vertebra (BSPG 2006 I 61) in (**A**) dorsal, (**B**) left lateral, (**C**) anterior and (**D**) posterior view. ?Testudines indet., proximal part of femur (NMC 41973) in (**E**) anterior and (**F**) posterior view. Testudines indet., metapodial (NMC 41975) in (**G**) anterior, (**H**) proximal and (**I**) distal view. Testudines indet., selected metapodials (NMC 50846 A-E) in (**J**) dorsal view. Scale bars equal 3 cm in **A-D**, 3 cm in **E** and **F**, 3 cm in **G-J**.

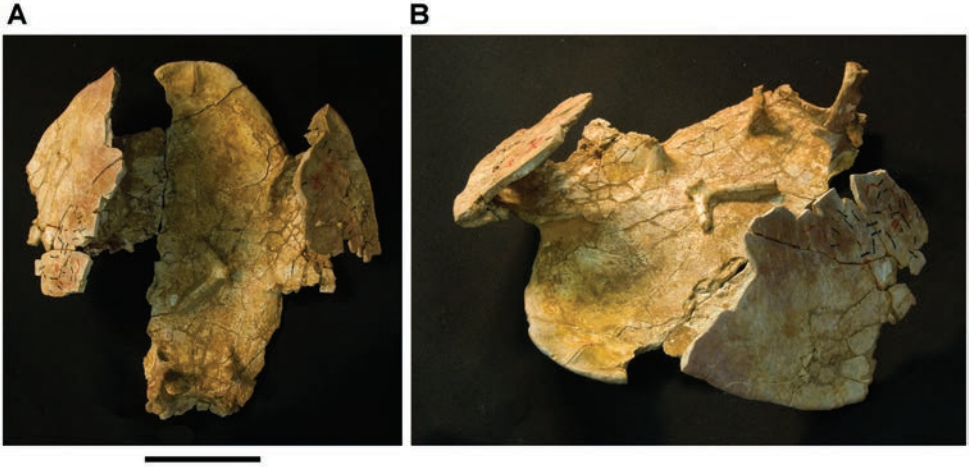

Figure 72. Partial shell and girdle bones of cf. *Galianemys* sp. (UCRC PV18) in (**A**) dorsal and (**B**) anterodorsolateral view. Scale bar for **A**: 10 cm.

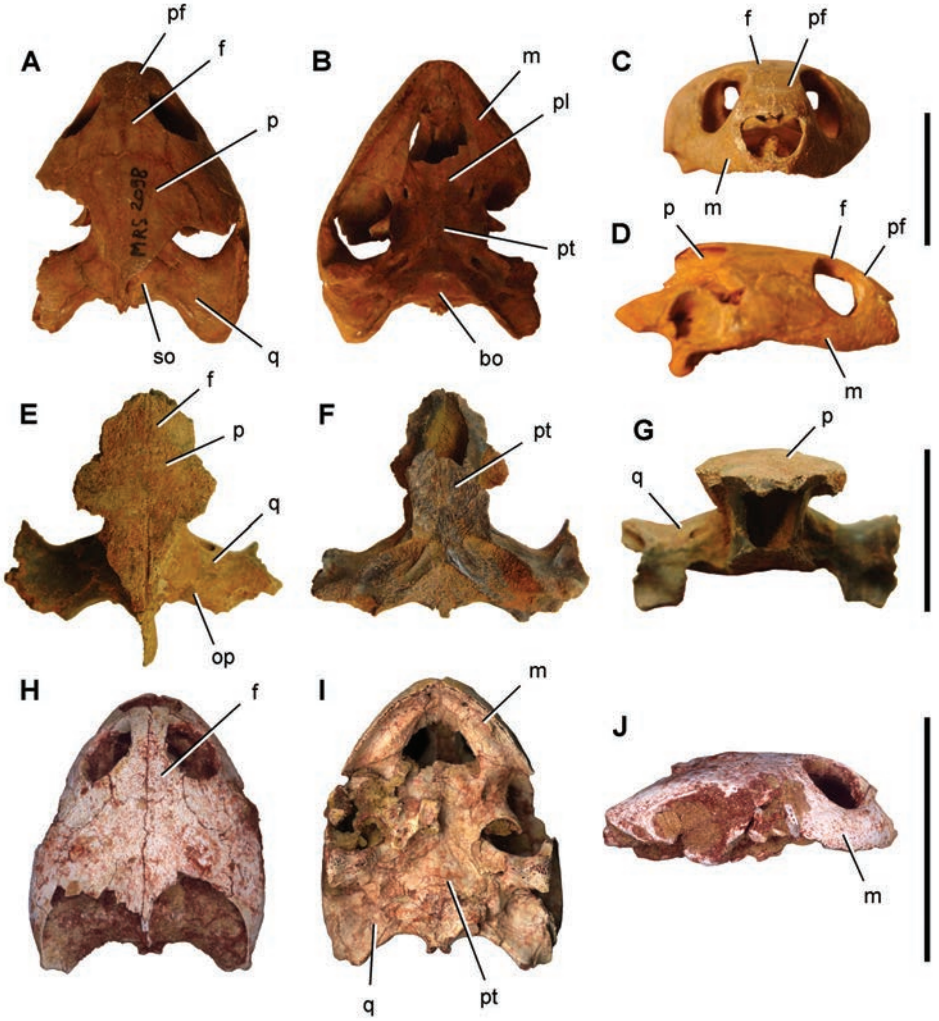

Figure 73. Turtle skull elements from the Kem Kem Group. One of two undescribed skulls likely referable to *Galianemys whitei* (MNHN-MRS 2098) in (**A**) dorsal, (**B**) ventral, (**C**) anterior and (**D**) right lateral view. Partial skull of *Galianemys* sp. (FSAC-KK 938) in (**E**) dorsal, (**F**) ventral and (**G**) anterior view. Skull of *Dirqadim schaefferi* (MDEt 41) in (**H**) dorsal, (**I**) ventral and (**J**) right lateral view. Scale bars equal 3 cm in **A-D**, **E-G** and **H-J**. Abbreviations: **bo** basioccipital, **f** frontal, **m** maxilla, **op** opisthotic, **p** parietal, **pf** prefrontal, **pl** palatine, **pt** pterygoid, **q** quadrate, **so** supraoccipital.

a then narrower Atlantic Ocean (Gaffney et al. 2006). The araripemydid carapace is composed of thin, flat, densely pitted bones that resemble carapace fragments from the Kem Kem Group (Fig. 70A-F). We tentatively refer this Kem Kem material to the Araripemydidae on the basis of these features and await the recovery of more complete specimens (Table 8).

That decision, to limit referral of partial isolated shell elements of this form to Araripemydidae, is prudent and based on a cladistic diagnosis of the family that specifically cites shell structure and texture among some 20 synapomorphies that unite the two valid genera *Araripemys* and *Laganemys* (Sereno and ElShafie 2013: 219). Some authors, in contrast, have attempted to refer isolated shell pieces from South America and Africa to specific araripemydid genera or species, when the material does not exhibit more specific diagnostic features.

Thin ornamented shell material characterizing araripemydids is rare in the Kem Kem Group (Fig. 70A-F). One thin ornamented partial hypoplastron (Fig. 70A, B) was found among more than 400 shell fragments collected by Lavocat in the 1950s and referred by Gmira (1995) to the genus *Araripemys* as an indeterminate species (also Cavin et al. 2010). This partial plastron element, however, is insufficient for generic or specific assignment within Araripemydidae, as there are no features that allow reference to *Araripemys*, a genus based on material from earlier Aptian-Albian deposits in Brazil. Specimens referable to *Araripemys* are limited to those from the Araripe Basin of Brazil (Gaffney et al. 2006).

In a similar manner, Broin (1980) erected a new genus and species, *Taquetochelys decorata*, on the basis of a hypoplastron fragment from the Aptian-Albian Elrhaz formation of Niger (Sereno and ElShafie 2013: fig. 14 B, C). It is less complete than the hypoplastron from the Kem Kem araripemydid. Broin listed ten additional shell fragments as paratypes and cited (with uncertain status) an additional 31 shell pieces. This material was surface collected from a region known as Gadoufaoua during four expeditions in the 1960s and early 1970s. There is no specific type locality cited, and the material is surely derived from many individuals.

The diagnosis offered by Broin (1980: 42) for *Taquetochelys decorata* was inadequate at the time it was coined: "Pleurodire à carapace décorée de très petites granulalions et bourrelets, serrés. Petits mésoplastrons latéraux, courts". She tried to differentiate the species on details of its beaded decorative pattern and the presence of a mesoplastron, which she inferred from the beveled margin of the hypoplastron. Neither are diagnostic at the generic or species level, as pointed out by Gaffney et al. (2006: 111). The general form of decoration is similar between Nigerien and Brazilian specimens, and the presence of a mesoplastron is a primitve feature, its loss diagnostic for *Araripemys* (Sereno and ElShafie 2013).

A nearly complete, articulated skeleton with a skull was later described from the Elrhaz Formation as *Laganemys tenerensis* (Sereno and ElShafie 2013), its diagnosis including more than 20 autapomorphies in the skull and postcranial skeleton. These features clearly distinguish this taxon from the contemporary Brazilian genus and species *Araripemys barretoi*, its diagnosis also revised. The hypoplastron of the holotypic specimen of *L. tenerensis* also exhibits several shape and ornamentation differences to the hypoplastron originally described as *Taquetochelys decorata* (Sereno and ElShafie 2013: fig. 14.14). Sereno and ElShafie (2013) came to the same conclusion as Gaffney et al. (2006), that the material upon which *Taquetochelys decorata* is based is insufficient and should be regarded as a *nomen dubium*.

More recently Pérez-García (2017, 2018) has attempted to resurrect *Taquetochelys decorata* and reduce *Laganemys tenerensis* as a junior synonym by arguing that the differences cited between their hypoplastra fall within an acceptable range of individual variation. Pérez-García stated that "the anterior margin of the hypoplastron" was broken away, rendering ineffective any shape differences based on this bone. But our examination and specimen figures of the element show it as complete anteriorly (Pérez-García 2018: fig. 1O; see also Broin 1980: pl. III, fig. 10), and the anterior margin was cited for evidence of the presence of a mesoplastron.

Fragmentary holotypic specimens without diagnostic features lend themselves to subsequent taxonomic ambiguity. At no point has any author offered a revised diagnosis of *T. decorata* based solely on the holotypic partial hypoplaston or even on the numerous additional shell pieces referred to this taxon by Broin (1980). The material associated with *Taquetochelys decorata* consists entirely of isolated shell fragments that lack diagnostic features and specific locality data. Although this material is generally consistent with the nearly complete holotype skeleton of *Laganemys tenerensis*, there is no solid basis for regarding them as the same taxon. Gaffney et al. (2006: 111) remarked that it is impossible to diagnose *T. decorata* on the basis of the holotypic plastron piece. We agree and regard *Taquetochelys* and *T. decorata* as *nomina dubia*, the material referable to the Araripemydide on the basis of its ornamentation and several other familial synapomorphies (Sereno and ElShafie 2013: 219).

We anticipate eventual recovery of more complete araripemydid remains from the Kem Kem Group. More complete remains may resolve its taxonomic distinction and its affinities with the slightly older African and South American genera, *Laganemys* and *Araripemys,* respectively.

Euraxemydidae Gaffney et al., 2006

***Dirqadim schaefferi* Gaffney et al., 2006.** *Dirqadim schaefferi* from the Kem Kem Group is closely related to the slightly older and more completely preserved *Euraxemys esswini* from the Santana Formation in Brazil (Gaffney et al. 2006). *D. schaefferi* is known from two crania, one of which is nearly complete (Fig. 73H-J), both of which were commercially collected from unknown locations. Currently no postcranial material from the Kem Kem Group can be referred to the family, genus or species. A few features of the shell are diagnostic for *Euraxemys esswini* (Gaffney et al. 2006: 40) and may eventually allow reference of more complete shell material from the Kem Kem Group to *D. schaefferi.*

Podocnemidoidea Cope, 1868

***Hamadachelys* Tong & Buffetaut, 1996.** *Hamadachelys escuilliei* (Tong and Buffetaut 1996) is known from a well-preserved cranium and mandible. A second mandible was referred to this species (Gaffney et al. 2006: fig. 251). All of this material was collected commercially from unknown localities in the Kem Kem Group. The diagnostic features of *H. escuilliei* are in need of revision in light of the significant cranial material discovered and described since this taxon was named. The narrow interor-

bital distance in dorsal view of the cranium (Tong and Buffetaut 1996) more closely resembles *Euryaxemys* from Brazil than the contemporaneous genera *Dirqadim* and *Galianemys*. Postcranial material cannot be reliably referred to *Hamadachelys escuilliei*. *Hamadachelys* has been positioned phylogenetically between euryaxemydids and *Galianemys* at the base of the podocnemidoid radiation (Gaffney et al. 2006, 2011).

***Galianemys* Gaffney et al., 2002.** The podocnemidoid *Galianemys* is the best known turtle in the Kem Kem Group and is represented by several nearly complete and partial crania (Gaffney et al. 2002). Two species were named, *G. whitei* and *G. emringeri*, with several specimens referred to one or the other species. We figure two additional crania here (Fig. 73A-G), the more complete of which was commercially collected and is referable to *Galianemys whitei* (Fig. 73A-D). The prefrontal-frontal suture is straight rather than posteriorly convex (Fig. 73A), the jugal is separated from the posterior orbital margin by significant maxilla-postorbital contact (Fig. 73A), and the jugal contacts the palatine on the posterolateral aspect of the palate (Fig. 73B). These are diagnostic characters that differentiate this species (Gaffney et al. 2006). Unlike *Dirqadim*, another pleurodire from the Kem Kem Group (Gaffney et al. 2006), the triturating surface on the maxilla expands posteriorly assuming a broad tringular shape in ventral view (Fig. 73B), only minor ventral embayment is present along the jugal-quadratogugal margin in lateral view (Fig. 73D), and the U-shaped temporal emargination along the posterior margin of the skull roof is quite deep in dorsal view (Fig. 73A).

The second, less complete cranium (Fig. 73E-G), discovered in 2008 by a local in the Douira Formation at Aferdou N'Chaft east of Taouz (Fig. 9, locality 14), is referable to the genus *Galianemys* by the depth of the U-shaped posterior temporal emargination. Missing portions of the cranium, however, prevent assignment to a particular species.

Two very similar shell types, tentatively referred to *Galianemys* by Gaffney et al. (2006: figs 271–274), are based on nearly complete specimens of large size (carapace length approximately 55 cm) that were collected commercially from the Kem Kem Group. More recently Cavin et al. (2010) reported that these two shells were found at Tizi Tazguart, a locality south of Jbel Zireg (Fig. 9, locality 9) and near another site that yielded some 30 turtle shells that remain at large. Cavin et al. (2010) regarded the two large shell types as confidently referable to *Galianemys,* and Karl (2010: fig. 1) tentatively referred them to the two species, *G. whitei* and *G. emringeri*. No evidence has been forwarded, however, to justify these referrals, as there currently exists no clear association of cranial and postcranial remains for any testudines in the Kem Kem Group.

A partial carapace, complete plastron and associated pectoral and pelvic girdles of a fairly large testudine (Fig. 72) was collected in 1995 from a site near our section at Oum Tkout in the Douira Formation. The Douira Formation is approximately 100 m in thickness in this region, and the associated shell and girdle material was found approximately 28 m above the first substantial mudstone at the base of the formation. It matches one of the shell types described by Gaffney et al. (2006: fig. 274) and Karl (2010: fig. 1.2). On the plastron, the intergular scute is broad and equal in transverse

width to adjacent gular scutes, a plastral scute pattern attributed without justification to *G. whitei* (Karl 2010: fig. 1.2). The carapace measures 31 cm in length (Fig. 72), or approximately 56% the size of the specimens described by Gaffney et al. (2006). These shell types may pertain to *Galianemys* and its two closely related species, although that needs to be established by specimens associating cranial and shell material. At present the single specimen we figure here (Fig. 72) is the only one that provides any evidence of association for Kem Kem Group testudines, in this case between shell and non-shell postcranial bones.

Squamata, Oppel, 1811

In recent years, fossil discoveries have brought to light jaw fragments and, more rarely, nearly complete skeletons of extinct genera positioned as stem taxa to extant squamate clades. For Iguania and Ophidia, in particular, new fossils from circum-Tethyan sites have drawn their stem lineages back to the Early Cretaceous and, in some cases, to the Early Jurassic (Evans et al. 2002, Hsiang et al. 2015, Caldwell et al. 2015, Martill et al. 2015, Simões et al. 2017).

From Morocco more specifically, fragmentary squamate material was first reported in abundance from the Early Cretaceous site Anoual (Broschinski and Sigogneau-Russell 1996). The Kem Kem Group, thus far, has yet to yield abundant isolated vertebrae or more complete remains of squamates, respectively, from sediment screening or the pond locality Oum Tkout. Squamate remains consist of rare, isolated jaw fragments and vertebrae pertaining to stem acrodont iguanians, borioteiioids, and ophidians.

Iguania Cope, 1864
Acrodonta Cope, 1864
Jeddaherdan. A jaw fragment with blunt, unornamented, imbricate crowns that are ankylosed to the dentary was described as *Jeddaherdan aleadonta*, a new acrodont iguanian with potential affinities with uromasticine agamids (Apesteguía et al. 2016a). It was collected approximately 70 years ago by French paleontologist René Lavocat from a locality (Gara Tabroumit) southwest of Gara Sbaa. The horizon remains unknown. The only other fossil discovered so far pertaining to a squamate is a single trunk vertebra from Taouz of indeterminate relationship (Rage and Dutheil 2008).

Ophidia Brongniart, 1800
Norisophis. More than 100 ophidian vertebrae were recovered from field work in the Kem Kem Group between 1995 and 2018. They were found at sites in both formations, with several recovered at the pond locality Oum Tkout in the Douira Formation (Rage and Dutheil 2008). More recently, isolated vertebrae were also found in the Gara Sbaa Formation at Aferdou N'Chaft and nearby localities pertaining to *Norisophis begaa*, a new basal snake (Klein et al. 2017). Centrum length is a little more than 5 mm.

Lapparentophiidae Hoffstetter, 1959

Lapparentophis. Vullo (2018) described two moderately elongate mid-trunk vertebrae from near Begaa (close to Taouz), naming a new species in the genus *Lapparentophis*, *L. ragei*, which was known previously from Algeria. The diameter of the neural canal is much smaller than that of the cotyle, which is slightly broader than high. Centrum length is a little more than 1 cm.

Simoliophiidae Nopcsa, 1925

Simoliophis. Rage and Dutheil (2008) referred isolated vertebrae to *Simoliophis* cf. *lybicus* (Fig. 68E, F). They were found in both formations at several localities including Taouz, Oum Tkout, Douira and Dar El Karib. Two simoliophiid sacral vertebrae were also identified from Taouz and Oum Tkout localities that show similarities to the sacral vertebrae of snakes with hind limbs (Caldwell and Lee 1997, Rage and Escuillé 2000, Apesteguía and Zaher 2006).

Nigerophiidae Rage, 1975. Rage and Dutheil (2008) referred 18 dorsal vertebrae to this extinct aquatic snake family, which has been recorded from circum-Tethyan sites of Late Cretaceous and Paleocene age (Rage and Werner 1999). The dorsal centra are elongate and transversely narrow with tall, anteroposteriorly short neural spines.

Madtsoiidae Hoffstetter, 1961. Rage and Dutheil (2008) referred some 20 ophidian vertebrae from Taouz and Oum Tkout to this diverse extinct family, the neural arches characterized by a pair of parazygantral foramina on the posterior aspect of the neural spine and the absence of prezygapophyseal processes (Wilson et al. 2010). Rage and Dutheil (2008) regarded these Kem Kem vertebrae as distinct from the similar-sized matsoiid snake recorded from roughly coeval Cenomanian age rocks in Sudan (Werner and Rage 1994). These snakes grew to modest adult size with dorsal centra approximately 5 mm in length.

Crocodyliformes Hay, 1930

The Kem Kem Group has yielded a diverse array of crocodyliforms in size and trophic adaptations, ranging from small insectivorous or herbivorous candidodontid and sphagesaurid notosuchians less than 1 m in body length to large carnivorous neosuchians approaching the 12-meter length of *Sarcosuchus* (Broin and Taquet 1966, Sereno et al. 2001). Long-snouted crocodyliform fossils discovered in the late 1940s were initially assigned to the genus *Thoracosaurus* (Lavocat 1955), a eusuchian genus known only from North America and Europe (de Lapparent de Broin 2002). Later it was given a new genus *Elosuchus*, as *E. cherifiensis* (de Lapparent de Broin 2002, Young et al. 2017, Meunier and Larsson 2017). A braincase of this genus and species was erroneously referred to *Libycosuchus* (Buffetaut 1976), a very different short-snouted crocodyliform from the contemporaneous Bahariya Formation of Egypt (Stromer 1914).

By the 1990s, considerable new crocodyliforms fossils came to light in commercial collections (e.g., Russell 1996) and in the course of field work (Sereno and Larsson 2009). Based on fragmentary material, Buffetaut (1994) named *Hamadasuchus rebouli*,

a peirosaurid crocodyliform. Larsson and Sues (2007) later described and attributed a complete cranium to this genus and species. Sereno and Larsson (2009) named a notosuchian, *Araripesuchus rattoides,* and a stomatosuchid, *Laganosuchus maghrebiensis,* on the basis of partial dentaries of distinctive form. A single elongate distal caudal vertebra was initially described as pertaining to a new neotheropod dinosaur, *Kemkemia auditorei* (Cau and Maganuco 2009), which was soon regarded as a *nomen dubium* after its close resemblance to the distal caudals of extant crocodilians became apparent (Lio et al. 2012). Derived notosuchian teeth came to light in screen-washed sediment (Larsson and Sidor 1999), and more recently a small notosuchid with multicuspid crowns was named *Lavocatchampsa sigogneaurussellae* (Martin and de Lapparent de Broin 2016). We review Kem Kem Group crocodyliforms below, a group that will likely increase in diversity with the continued recovery of new specimens.

Notosuchia Gasparini, 1971
Uruguaysuchidae Gasparini, 1971

This family of notosuchians, united as a clade in some analyses (e.g., Leardi et al. 2015), includes the genera *Anatosuchus*, *Uruguaysuchus* and *Araripesuchus*.

Araripesuchus. The speciose genus *Araripesuchus*, known initially from South America and later from Africa and Madagascar, also occurs in the Kem Kem Group as *Araripesuchus rattoides* (Fig. 74). It was named on the basis of a partial, edentulous dentary with 14 alveoli that was collected commercially and thus comes from an uncertain locality and horizon (NMC 41893, as CMN 41893 in Sereno and Larsson 2009). Referred specimens include partial dentaries from the Douira Formation collected at Dar El Karib (near Erfoud) and another collected commercially from an uncertain locality and horizon (BSPG 2008 I 41, Fig. 74E, F).

BSPG 2008 I 41 preserves dentary teeth 2–6 and 10 and the roots of dentary teeth 11 and 12. The socket for the first dentary tooth projects anteriorly and presumably held a procumbent and slightly enlarged tooth, a diagnostic feature for the species. As in *Araripesuchus wegeneri*, the fourth dentary tooth is enlarged, whereas dentary teeth 3, 5, and 6 are smaller and subconical. The alveoli for dentary teeth 9 and 10 are incompletely separated, and dentary tooth 10 has a labiolingually compressed crown with its carina and apex rounded by tooth abrasion as occurs in *A. wegeneri* (Sereno and Larsson 2009).

A. rattoides does appear to be distinct from *A. wegeneri*, which is much better known from complete skulls and skeletons from the older (Aptian-Albian) Elrhaz Formation in Niger. Compared to the latter, many of the dentary alveoli are exposed in lateral view of the best-preserved dentary of *A. rattoides* (Fig. 74A) with the dentary symphysis held in a vertical plane. In dorsal view with the symphysis in a sagittal plane, the skull appears to be proportionately narrower than a comparable view of the dentary in *A. wegeneri* (Sereno and Larsson 2009). Specimen BSPG 2008 I 41 (Fig. 74E, F) is identical in form but slightly larger than the holotype of *A. rattoides*. Differences in the exposure of the alveoli as figured here are due to the orientation of the specimen when photographed (the dentary symphysis tipped from a vertical plane).

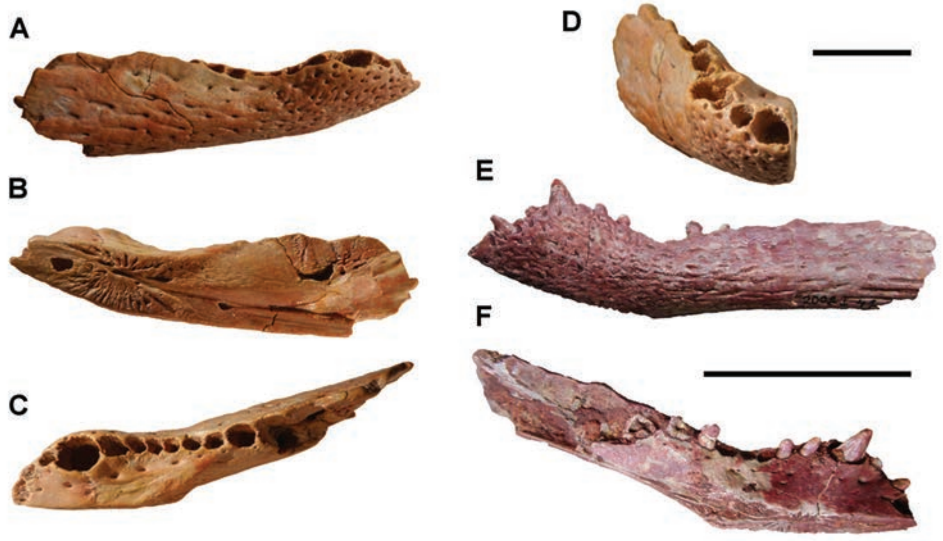

Figure 74. Specimens referred to *Araripesuchus rattoides* from the Kem Kem Group. NMC 41893 in (**A**) right lateral, (**B**) medial, (**C**) dorsal (occlusal) and (**D**) anterior view. BSPG 2008 I 41 in (**E**) left lateral and (**F**) dorsomedial view. Scale bars equal 3 cm in **A-C**, **E** and **F**, and 1 cm in **D**.

Candidodontidae Carvalho et al., 2004

This derived family of small notosuchians, first described by its namesake genus *Candidodon* from Brazil (Carvalho and Campos 1988), now is well known from Early Cretaceous genera from east Africa, such as *Malawisuchus* and *Pakasuchus* (O'Connor et al. 2010). Candidodontidae forms a clade in some phylogenetic analyses of notosuchians (O'Connor et al. 2010). In other analyses *Candidodon* and allies are positioned as basal outgroups to *Notosuchus* and other notosuchians including sphagesaurids (e.g., Pol et al. 2014, Leardi et al. 2015).

Lavocatchampsa. Recently a commercially collected partial skull from the Kem Kem Group was described as *Lavocatchampsa sigogneaurussellae* (Martin and de Lapparent de Broin 2016). Its complex, multicuspid crown morphology includes a labial and lingual cingulum reminiscent of that in the molariform teeth of Cretaceous mammaliaforms. Other derived features include the absence of a caniniform tooth. Unlike *Notosuchus* and close relatives, masticatory movement appears to have been orthal rather than propalinal. The relationships of *Lavocatchampsa* within Candidodontidae are uncertain. It has been resolved as the sister group to the somewhat older genera *Malawisuchus* and *Pakasuchus* (Martin and de Lapparent de Broin 2016). *Lavocatchampsa* also resembles *Adamantinasuchus* (Nobre and Carvalho 2006) from the slightly younger Adamantina Formation of Brazil.

Notosuchia indeterminate. Small multicuspid crocodyliform teeth from the Douira Formation were first reported by Larsson and Sidor (1999). They were dis-

covered in a clay-rich horizon immediately beneath the fossiliferous pond deposit at Oum Tkout. Two tooth forms were described, both of which differ from the crown morphology in *Lavocatchampsa* (Martin and de Lapparent de Broin 2016). In the first tooth form, the crowns are subtriangular in labiolingual views and have a major row of cusps flanked by lower parallel rows to each side (UCRC VP155, VP156), as figured in Larsson and Sidor (1999: fig. 2, as SGM-Rep 4, -Rep 5). The crown shape, apical row of cusps, and accessory cusp rows resemble the molarifom crowns in *Candidodon* (Pol et al. 2014), *Adamantinasuchus* (Nobre and Carvalho 2006) and the Bolivian notosuchian *Yacarerani* (Novas et al. 2009). The first tooth form, however, is distinctive in the variable size and location of the cusps and its pattern of tooth-to-tooth abrasion. A large, low-angle, planar wear facet truncates one of the crowns (Larsson and Sidor 1999: fig. 2D), which differs from the wear pattern present in *Lavocatchampsa* (Martin and de Lapparent de Broin 2016).

In the second tooth form (UCRC VP157) described by Larsson and Sidor (1999: fig. 3, as SGM-Rep 6), multiple cusp rows also occur, but the crown has a more rounded profile than in many notosuchians such as *Malawisuchus* (Gomani 1997). The crown is ovate in occlusal view with a main cusp and several accessory cusps oriented along the major axis. The main cusp is located at the extreme mesial or distal end of the crown, rather than a central location, assuming the major axis of the crown is mesiodistal. Grossly similar to the second tooth form are ovate crowns with few cusps and an unusual texture of vertical striations pertaining to an unnamed notosuchian from Upper Cretaceous rocks in Brazil (Montefeltro et al. 2009). The second tooth form from the Kem Kem Group, however, differs in a number of regards as noted by Montefeltro et al. (2009) and cannot be assigned to any currently known notosuchian. These tooth forms suggest that there exists a greater diversity of small notosuchians in the Kem Kem Group than are currently recognized.

Peirosauridae Gasparini, 1982

Peirosaurids are a diverse and loosely united group of crocodyliforms lying outside Neosuchia. Some authors have united peirosaurids and sebecids as Sebecia outside Notosuchia (Larsson and Sues 2007, Sereno and Larsson 2009, Meunier and Larsson 2017), whereas others have positioned peirosaurids and sebecids as independent clades within Notosuchia (Leardi et al. 2015, Fiorelli et al. 2016). Adding to this lack of resolution are studies that name new taxa on the basis of fragmentary cranial remains from the Kem Kem Group, which include the holotypic specimen of *Hamadasuchus rebouli* (Buffetaut 1974) and a partial braincase referred initially to the Egyptian genus *Lybicosuchus* (Buffetaut 1976). Further confusion has ensued with the use of the poorly defined taxon Trematochampsidae, to which peirosaurids have sometimes been assigned. Neither its namesake genus and species *Trematochampsa taqueti*, which was based on isolated fragments from Niger (Buffetaut 1976), nor the Family Trematochampsidae Buffetaut 1974 appear to be valid (Meunier and Larsson 2017).

In 2007 a suite of commercially collected material was described and referred to *H. rebouli*, including a nearly perfect adult cranium (ROM 52620, Fig. 75), braincases

(ROM 54511, 52059), a dentary (ROM 49282) as well as more fragmentary subadult specimens (Larsson and Sues 2007). The more gracile dentary differs in several regards from MDEC001. In dorsal view, the first three alveoli are more mesially positioned relative to the fourth alveolus given the sharper median convergence of the dentary rami. The fourth and thirteenth alveoli are largest, with a more dramatic increase in diameter from the eleventh alveolus. The splenial bounds the eleventh and more distal alveoli. Hypertrophied alveolar bone is not present either medially or laterally, and no groove is present. These differences render it difficult to refer the dentary or other material to *H. rebouli*. This assignment also was questioned by Cavin et al. (2010: 398), although no justification was provided. They also questioned referral to *H. rebouli* by Larsson and Sues (2007) of the braincase (MNHN-MRS 3101, Fig. 76C) initially identified as *Libycosuchus* by Buffetaut (1974). Yet, this specimen is clearly closer in morphology to the supratemporal fossa of ROM 52620 (Fig. 75; Larsson and Sues 2007) than to the holotypic specimen of the short-snouted Egyptian crocodyliform *Libycosuchus brevirostris* (BSP 1912 VIII 574-578, Figs 77A, F, 78).

More recently, a nearly complete skull and lower jaws were commercially collected from the Kem Kem Group and have yet to be described in detail (BSPG 2005 I 83, Rauhut and López-Arbarello 2006). At just more than 30 cm in length (Fig. 79), it is almost the exact same size as the previously described perfect cranium (Fig. 75) and, likewise, was referred to *H. rebouli*. We see little reason to doubt the assignment to *H. rebouli* of the new skull (BSPG 2005 I 83, Fig. 79) as well as a suite of more fragmentary material from the Kem Kem Group (Figs 80–82). One braincase was collected from the Gara Sbaa Formation at Aferdou N'Chaft and represents the only partial skull of *H. rebouli* from a known horizon and locality (FSAC-KK 930, Fig. 81A, B). Another specimen that was commercially collected from an unknown horizon and locality preserves the edentulous, fused dentary-splenial symphysis from an adult skull (CMN 41784, Fig. 82D-F). The description of the new complete skull BSPG 2005 I 83 should carefully refine the diagnostic characters listed for this genus and species based on the first complete cranium (Larsson and Sues 2007: 534). With that knowledge, many isolated cranial specimens may be referred with greater justification.

Neosuchia Clark, 1988

Stomatosuchidae Stromer, 1925

This derived family of moderate to large-sized crocodyliforms was first described as *Stomatosuchus inermis* from the likely Cenomanian-age Bahariya Formation of Egypt (Stromer, 1925). The nearly 2 m long flat cranium of the holotype was found in articulation with a very slender U-shaped mandible (Sereno and Larsson 2009). This iconic crocodyliform became all the more enigmatic when the holotype and only known specimen was destroyed in WWII. The lack of material has rendered uncertain the position of Stomatosuchidae within Crocodyliformes. As discussed below, the possible association of vertebrae with procoelous centra with this skull type suggests that Stomatosuchidae may fall within Neosuchia.

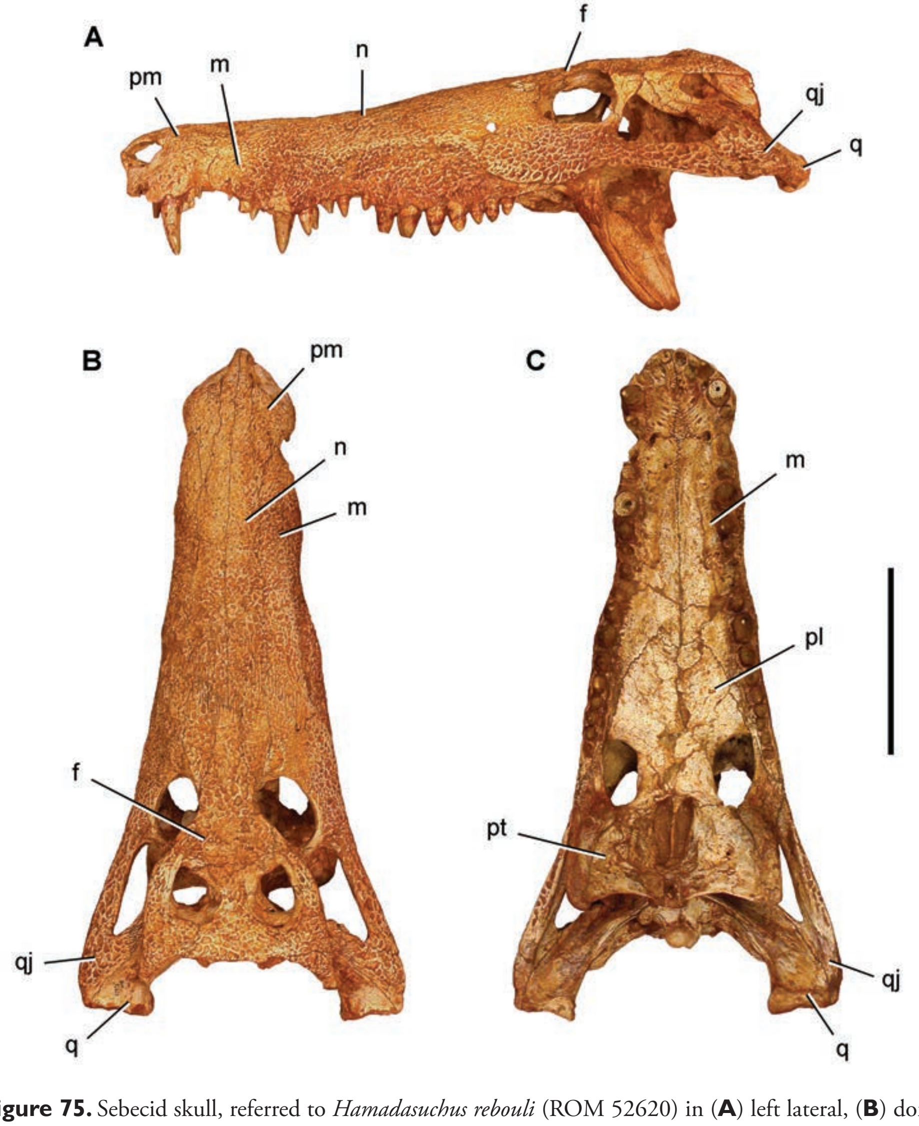

Figure 75. Sebecid skull, referred to *Hamadasuchus rebouli* (ROM 52620) in (**A**) left lateral, (**B**) dorsal and (**C**) ventral views (from H.-D. Sues; Larsson and Sues 2002). Scale bar equals 10 cm. Abbreviations: **f** frontal **m** maxilla **n** nasal **pl** palatine **pm** premaxilla **pt** pterygoid **q** quadrate **qj** quadratojugal.

Laganosuchus. Recently remains of a similar crocodyliform surfaced in the Echkar Formation of Niger (Sereno and Larsson 2009). This material was described as *Laganosuchus thaumastos* (Fig. 83A, B). A similar species, named *L. maghrebensis*, was described from dentary fragments from the contemporaneous Kem Kem Group (Fig. 83C-K; Sereno and Larsson 2009).

The holotype (UCRC PV2, Fig. 83C-E) preserves the first four alveoli, the first opened to fully expose an erupting crown. The tapering crown lacks recurvature and

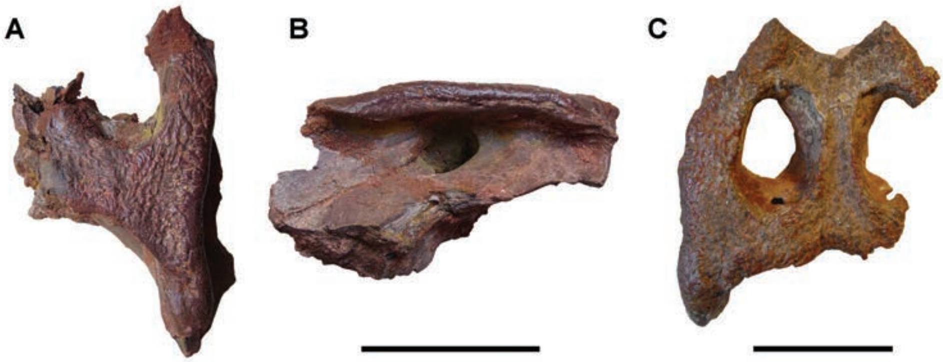

Figure 76. Other sebecid cranial specimens. Posterolateral skull roof (FSAC-KK 930) in (**A**) dorsal and (**B**) right lateral view. Partial braincase of *Hamadasuchus rebouli* (MNHN-MRS 3101) in (**C**) dorsal view (initially referred to *Libycosuchus,* Buffetaut 1994). Scale bars equal 10 cm in **A** and **B**, 5 cm in **C**.

exhibits light fluting on its lingual side. A referred specimen (NMC 50838, Sereno and Larsson 2009, Fig. 83I-K) preserves a similar portion of the anterior dentary ramus and also includes an erupting tooth in what appears to be the third alveolus. The tooth is approximately 17 mm in height and curves slightly distally at its tip. A second referred specimen with a fully erupted crown may also pertain to *L. maghrebensis* (NMC 41786, Fig. 84).

A larger dentary piece of *L. maghrebensis* was figured by Rauhut (2009), comprising the anterior portion of the left dentary and fused symphyseal end of the right dentary (Fig. 83F-H). The first alveolus contains a fully erupted caniniform crown followed by 10 empty alveoli (BSPG 2008 I 62, Fig. 83F-H). The dentary ramus thickens toward the symphysis, unlike the other smaller specimens of the species (Fig. 83D, G, J). A thickened symphyseal shelf was described as diagnostic of *L. thaumastos* (Sereno and Larssson 2009). Given the larger size of BSPG 2008 I 62, symphyseal thickening may occur with maturity. As in *L. thaumastos* (Sereno and Larsson 2009), the first, second and fourth alveoli are larger than the others (Fig. 83G). Unlike *L. thaumastos*, the more distal alveoli are closely spaced (Fig. 83A, G). In *L. thaumastos,* in contrast, the alveoli distal to the fourth are all well separated, except for the sixth and seventh (Fig. 83A). In addition, the alveoli in *L. thaumastos* are separated by an undulating margin that exposes the alveolar rim in lateral view (Fig. 83A). The more complete dentary of *L. maghrebensis* (BSPG 2008 I 62) confirms the original assessment that Moroccan and Nigerien specimens represent distinct species.

Aegisuchus. *Aegisuchus witmeri* was named on a commercially collected braincase of uncertain locality in the Kem Kem Group (ROM 54530, Holliday and Gardner 2012). This specimen closely resembles the braincase of *Aegyptosuchus peyeri* (Stromer 1933) from the Bahariya Formation of Egypt (Fig. 85). Although the braincase of *Aegyptosuchus peyeri* survived WWII, associated procoelous vertebrae suggestive of neosuchian relationships were destroyed (Stromer 1933).

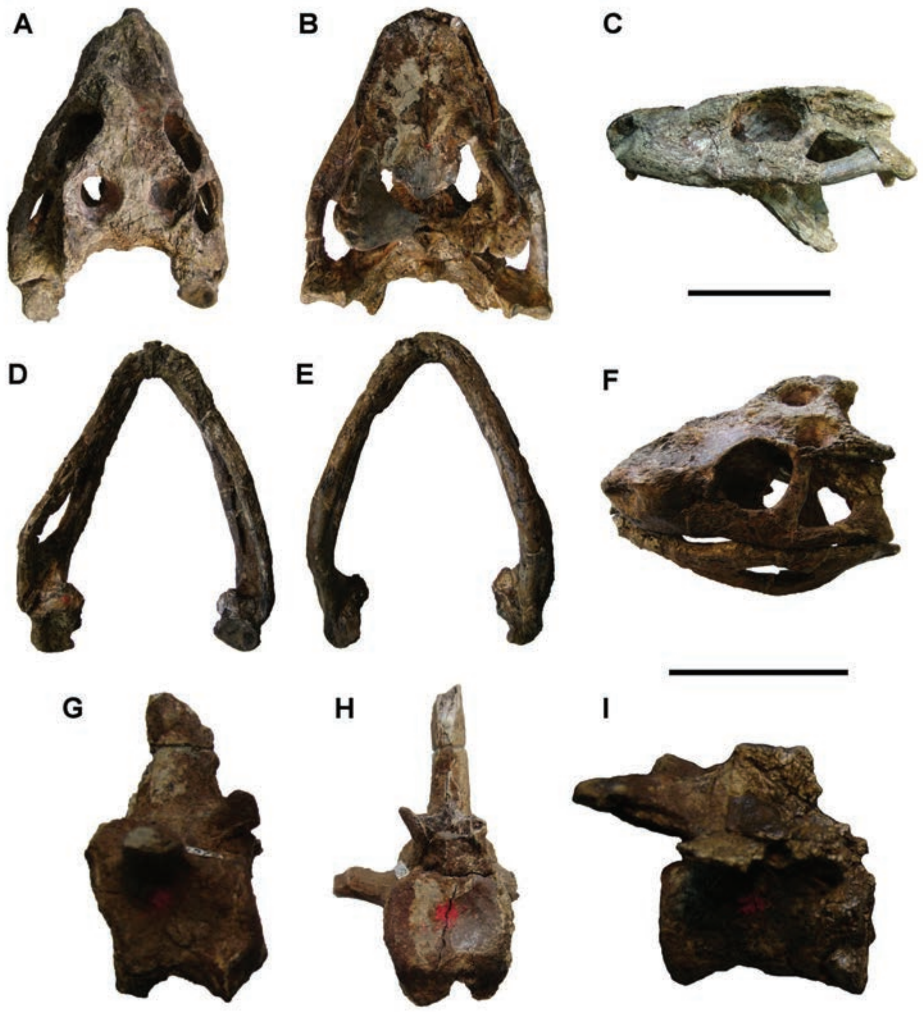

Figure 77. Original material of *Libycosuchus brevirostris* Stromer, 1914 (BSP 1912 VIII 574-578). Cranium in (**A**) dorsal, (**B**) ventral (**C**) left lateral view. Lower jaw in (**D**) dorsal and (**E**) ventral view. Skull in (**F**) dorsolateral view. ?Sacral vertebra in (**G**) lateral view. Caudal vertebra in (**H**) anterior and (**I**) lateral view. Scale bars equal 10 cm in **A-E**, 3 cm in **G-I**.

Striking features of the braincase link *Aegisuchus* and *Aegyptosuchus*, which Holliday and Gardner (2012) placed in Aegyptosuchidae on the basis of several cranial characters. In dorsal view, the orbits are very closely spaced, and supratemporal fossae are small, widely separated, and displaced anteriorly, and the occipital surface is broadly exposed in dorsal view (Fig. 85). The skull roof between the dorsally facing orbits is particularly narrow, the medial margin of each orbit closer to the midline than the medial rim of the supratemporal fossa. The quadrate shafts are very broad and posteriorly

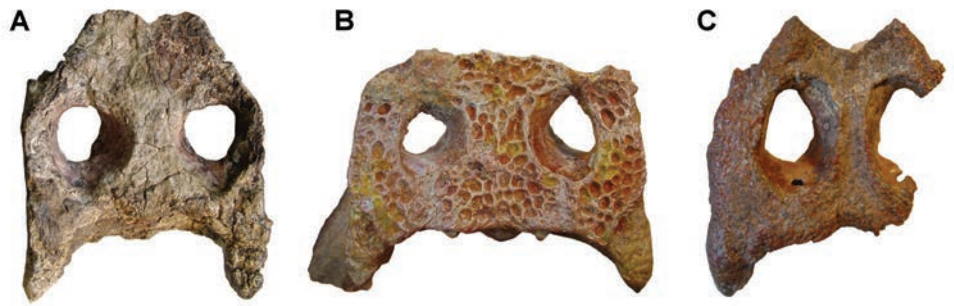

Figure 78. Comparison of the braincases – in dorsal view – of the small crocodyliforms (**A**) *Libycosuchus brevirostris*, (**B**) sebecid indet. (FSAC-KK 08) and (**C**) a specimen initially referred to *Hamadasuchus rebouli* (MNHN-MRS 3101). Specimens adjusted to similar size (see Figs 76, 77 and 81 for scale in **A**-**C**).

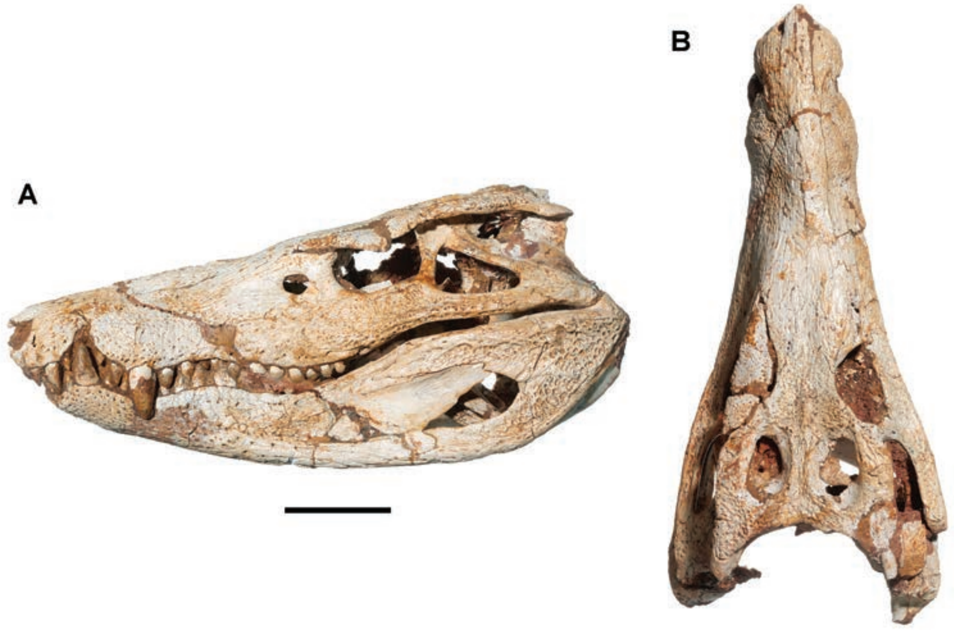

Figure 79. Skull of *Hamadasuchus rebouli* (BSPG 2005 I 83, Rauhut & López-Arbarello, 2006) in (**A**) lateral and (**B**) dorsal view (see). Scale bar equals 5 cm.

angled, indicating that the skull is quite dorsoventrally compressed. As reconstructed by Holliday and Gardner (2012: fig. 1), the cranium quite possibly was very low and elongate as in *Stomatosuchus inermis*.

All of these features, nevertheless, appear to be present in *Stomatosuchus inermis*, the braincase of which was only partially preserved (Stromer 1925, Sereno and Larsson 2009). The Egyptian *Aegyptosuchus peyeri*, thus, might constitute a junior synonym of *Stomatosuchus inermis* from the same formation. We are unable to differentiate the two

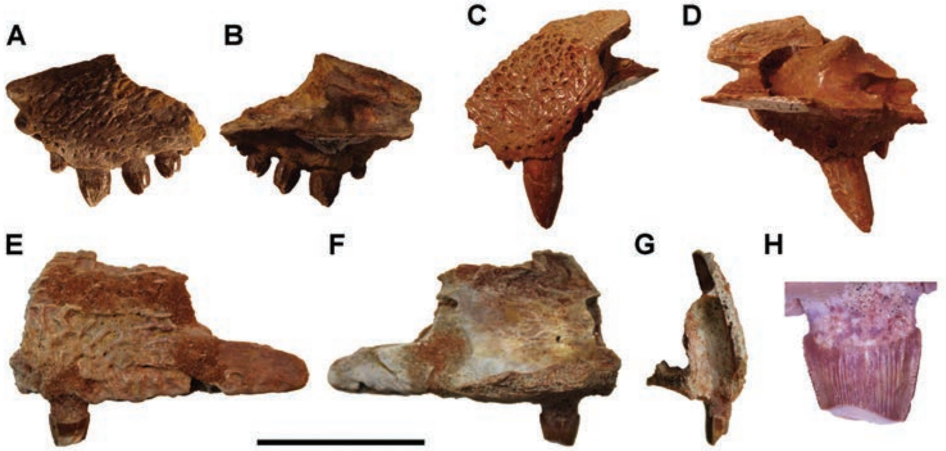

Figure 80. Sebecid upper jaw bones from the Kem Kem Group. Partial left maxilla (NMC 41866) in (**A**) lateral and (**B**) medial view. Partial left premaxilla (NMC 41892) in (**C**) anterolateral and (**D**) medial view. Maxillary fragment (FSAC-KK 932) in (**E**) lateral, (**F**) medial and (**G**) ?anterior view. Base of the maxillary crown (FSAC-KK 932) in (**H**) lateral view. Scale bar equals 3 cm in **A-G**, 1 cm in **H**.

on the basis of available images of *Stomatosuchus inermis*. The family Aegyptosuchidae, in turn, might well be redundant with Stomatosuchidae. Among the Moroccan specimens, there is no overlap between the braincase of *Aegisuchus witmeri* and the dentary sections of *Laganosuchus maghrebiensis*. Given their similar size, it is entirely possible that more complete specimens from the Kem Kem Group will show that *Aegisuchus witmeri* is a junior synonym of *Laganosuchus maghrebiensis*.

Pholidosauridae Zittel & Eastman, 1902

The first specimens of a long-snouted crocodyliform discovered in the Kem Kem Group were identified as *"Thoracosaurus" cherifiensis* (Lavocat 1955). *Thoracosaurus*, a eusuchian genus from North America and Europe, was abandoned in favor of a new genus *Elosuchus* (de Lapparent de Broin 2002), which came to be known from nearly complete crania and mandibles from Morocco, Algeria, and Niger. Given that there are several long-snouted clades of crocodyliforms, the taxonomic status and affinity of this material, including the specimens from the Kem Kem Group, has been uncertain.

Recently, the material of *Elosuchus* from Morocco and Algeria was redescribed as pertaining to two species of pholidosaurids. The Moroccan material, based on specimens from the Kem Kem Group, was attributed to *E. cherifiensis*, whereas the fossils from the potentially slightly older Albian beds in Algeria (at Gara Samani, Fig. 1) were attributed to a new species *E. broinae* (Meunier and Larsson 2017). The two species differ only in minor features. The Nigerien material originally described as *E. felixi* was transferred to a new genus *Fortignathus* and identified as a dyrosaurid (Young et al. 2017).

Elosuchus. Lavocat (1955) based "*Thoracosaurus*" *cherifiensis* on isolated crocodyliform bones from Gara Sbaa that were never illustrated or numbered. As most of

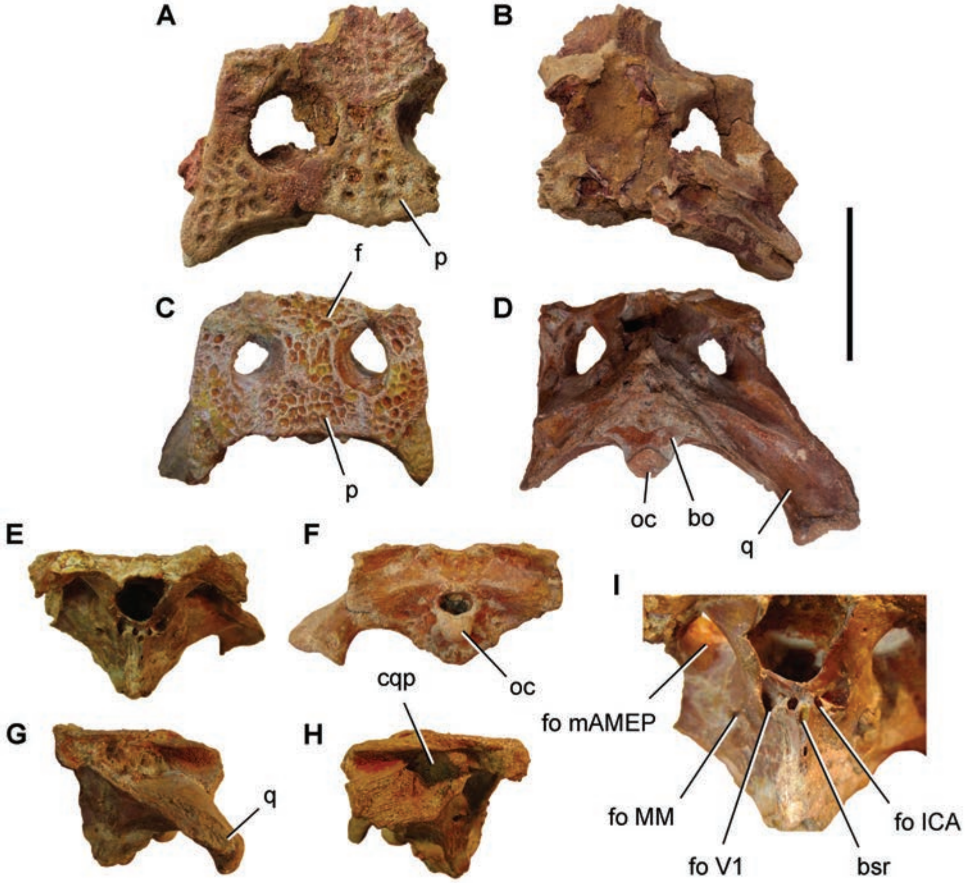

Figure 81. Sebecid braincases from Aferdou N'Chaft. Braincase (FSAC-KK 1237) referable to *Hamadasuchus rebouli* in (**A**) dorsal and (**B**) ventral view. Partial braincase (FSAC-KK 08) in (**C**) dorsal, (**D**) ventral, (**E, I**) anterior, (**F**) posterior, (**G**) left lateral and (**H**) right lateral view. Scale bar equals 5 cm in **A-H**. Abbreviations: **bo** basioccipital **bsr** basisphenoid recess **cqp** cranioquadrate passage **f** frontal **fo ICA** foramen for internal carotid artery **fo MM** maxillomandibular foramen **fo mAMEP** Musculus adductor mandibulae externus profundus **fo V1** ophthalmic foramen **oc** occipital condyle **p** parietal **q** quadrate.

this material was subsequently lost, a nearly complete skull was designated as the lectotype for *E. cherifiensis* (MNHN E 1, Meunier and Larsson 2017). As the skull was commercially collected, its exact horizon and locality within the Kem Kem Group are unknown. Features listed in a revised generic diagnosis include five premaxillary teeth, the first of which is displaced posterior to the second, paddle-shaped lacrimal, strong anterior process of the squamosal that overlaps much of the postorbital laterally, quadratojugal overlapping all of the quadrate near its laterodistal condyle, and a small pit at the anteromedial end of the dentary near the symphysis that receives the tip of the first premaxillary tooth (Meunier and Larsson 2017).

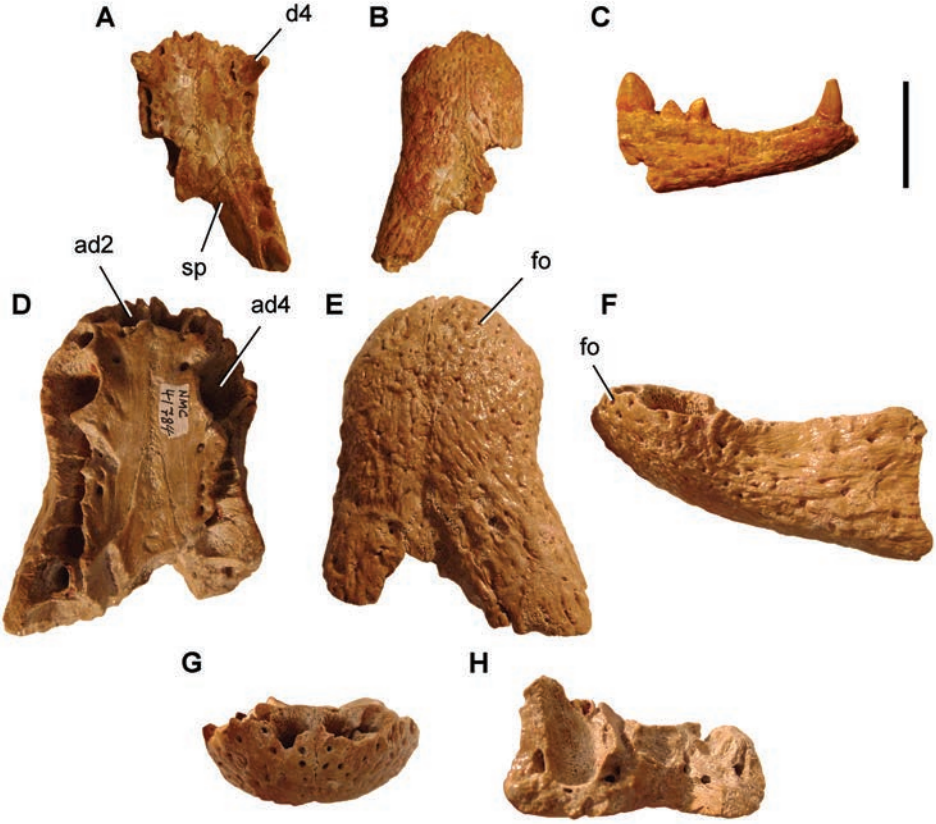

Figure 82. Mandibular symphyses of *Hamadasuchus rebouli***.** Mandibular symphysis (MNHN-MRS 3110) in (**A**) dorsal, (**B**) ventral and (**C**) right lateral view. Mandibular symphysis (NMC 41784) in (**D**) dorsal, (**E**) ventral, (**F**) left lateral, (**G**) anterior and (**H**) posterior view. Scale bar equals 3 cm. Abbreviations: **ad2**, **4** alveolus for dentary tooth 2, 4 **d4** dentary tooth 4 **fo** foramen **sp** splenial.

Using the revised diagnosis and lectotype cranium, additional specimens can be referred to *E. cherifiensis*, which include paired premaxillae and partial rostra (Figs 86, 87), the posterior half of crania (Fig. 88A-E), fragmentary cranial bones (Fig. 89), mandibular rami (Figs 90, 91), and, with less confidence, isolated teeth (Fig. 92). The bones of *E. cherifiensis* were surface collected from many sites in the Kem Kem Group. A partial premaxilla (FSAC-KK 923, Fig. 89A-E) was surface collected in the Gara Sbaa Formation, and an isolated jugal (FSAC-KK 09, Fig. 88F-H) and mandibular symphysis (FSAC-KK 753, Fig. 91) were collected from the Douira Formation.

De Lapparent de Broin (2002) referred large isolated scutes to *Elosuchus*, but the association of the skull remains and scutes described is unclear. Large osteoderms have previously been attributed to "*Sarcosuchus* sp". (Sereno et al. 1996), although evidence for this specific genus is lacking in the Kem Kem Group. Two morphologi-

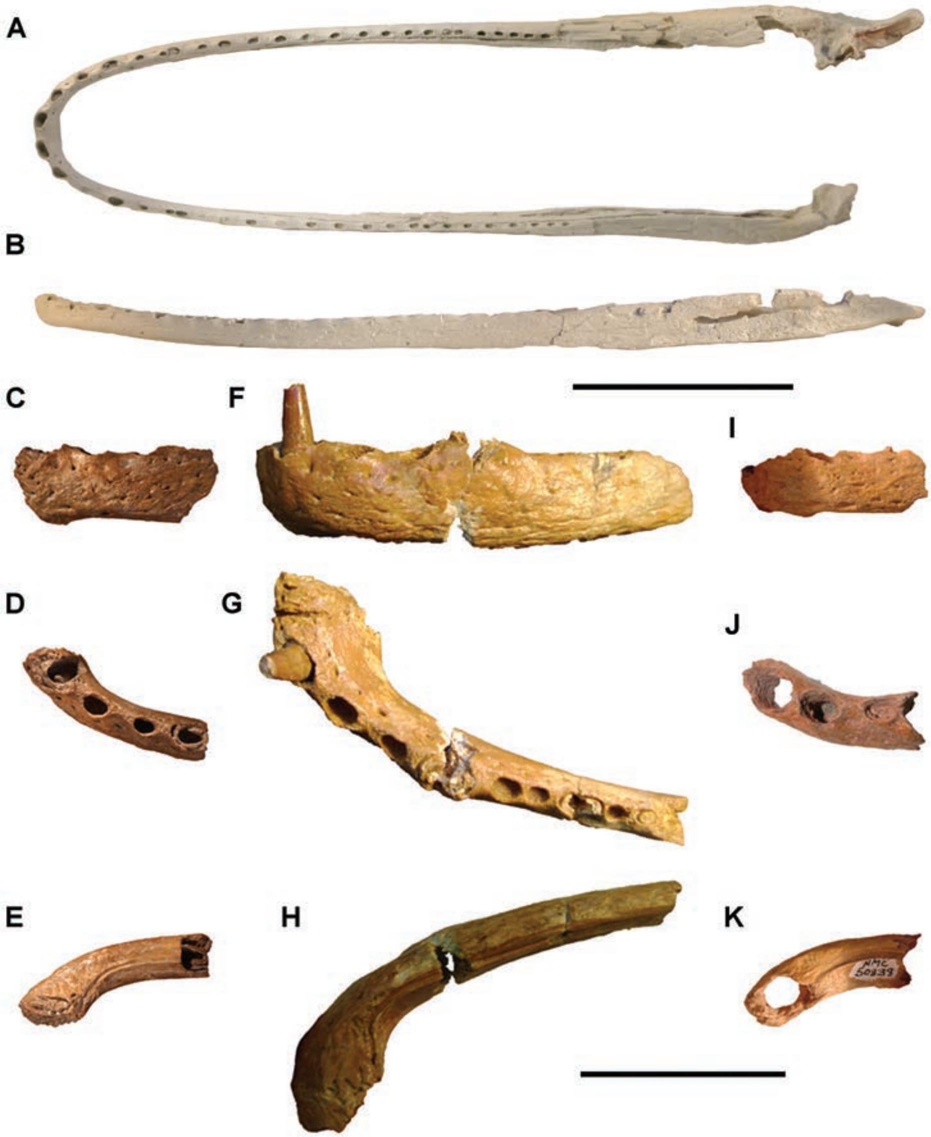

Figure 83. Specimens of *Laganosuchus* from the Echkar Formation in Niger and the Kem Kem Group in Morocco. Cast of lower jaws (UCRC PVC9) of the holotypic specimen of *L. thaumastos* (MNN IGU13) in (**A**) dorsal and (**B**) left lateral view. Holotypic specimen of *L. maghrebensis* (UCRC PV2) from the Kem Kem Group in (**C**) left lateral, (**D**) dorsal and (**E**) ventral view. BSPG 2008 I 62 in (**F**) left lateral, (**G**) dorsal (occlusal) and (**H**) ventral view. Partial dentary of *L. maghrebensis* (NMC 50838) in (**I**) lateral, (**J**) dorsal (occlusal) and (**K**) ventral view. Scale bars equal 20 cm in **A** and **B**, 6 cm in **C–K**.

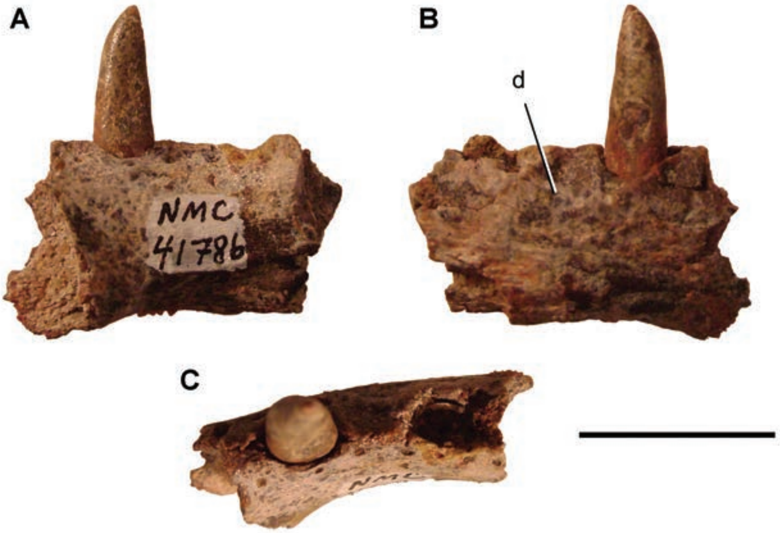

Figure 84. Dentary fragment with tooth referred to *Laganosuchus maghrebensis* (NMC 41786) in (**A**) lateral, (**B**) medial and (**C**) dorsal view. Scale bar equals 2 cm. Abbreviation: **d** dentary.

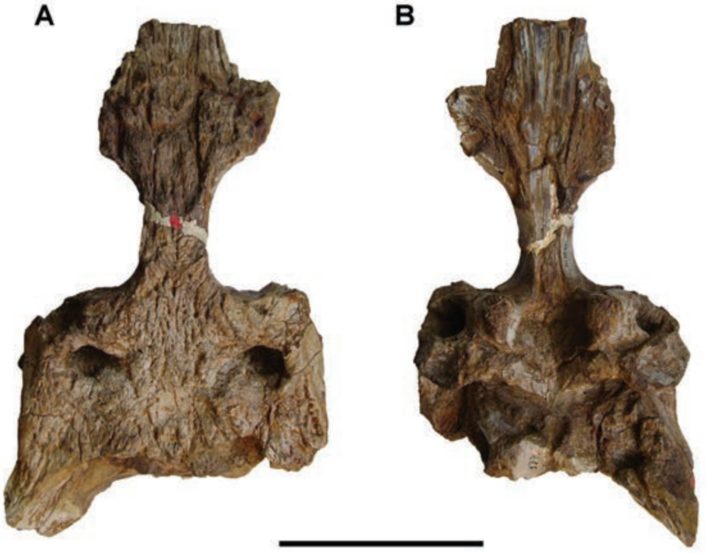

Figure 85. Holotypic specimen of *Aegyptosuchus peyeri* Stromer, 1933 from the Bahariya Formation of Egypt in dorsal (**A**) and ventral (**B**) view. Scale bar equals 10 cm.

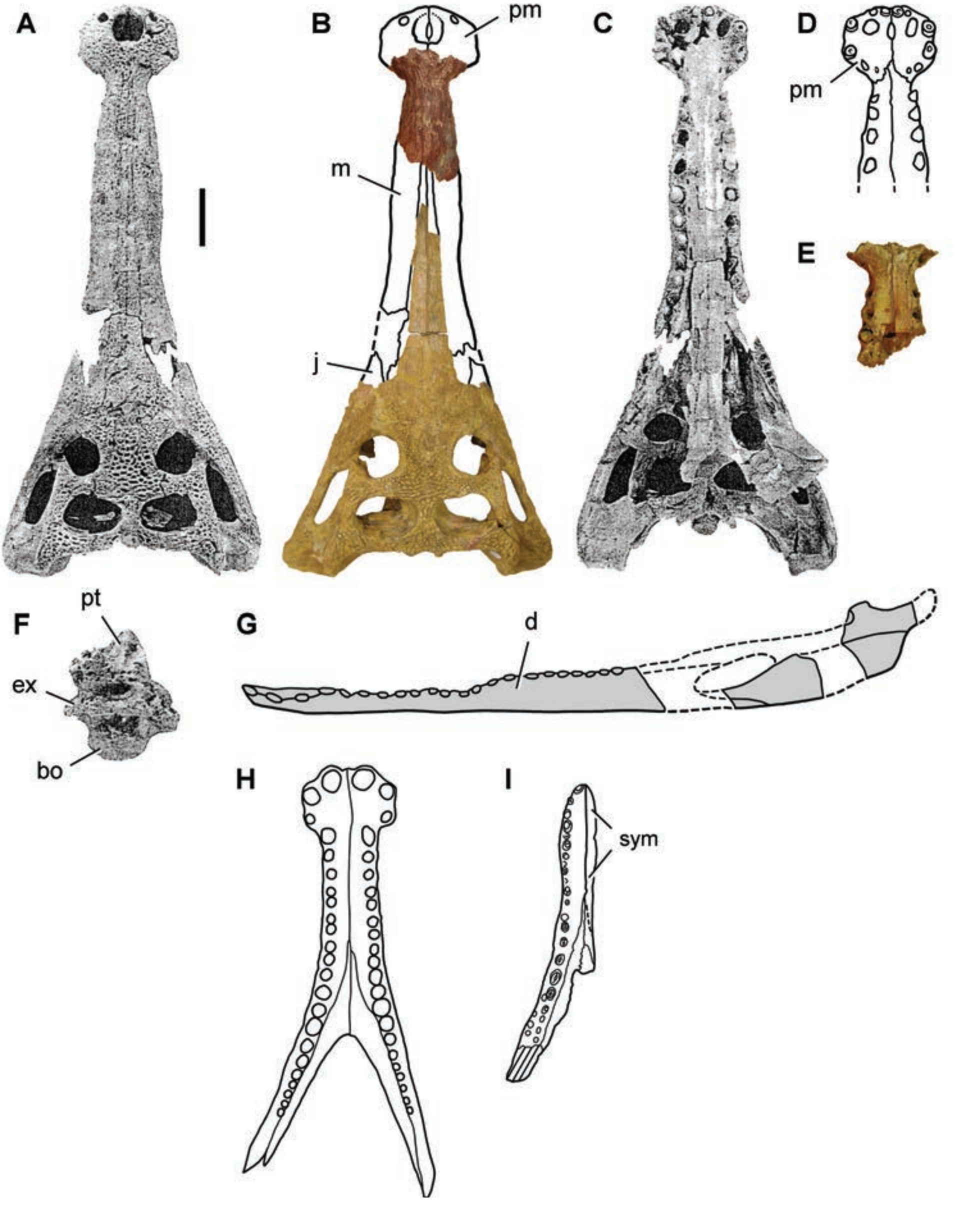

Figure 86. Cranial remains of the crocodyliform *Elosuchus*. **A, C** *E. cherifiensis* cranium (MNHN SAM 129) in dorsal and ventral views **B** *E.cherifiensis* (MNHN SAM 129) with posterior skull roof (NMC 41912) and anterior snout section (FSAC-KK 10) superimposed, **D** *E.cherifiensis* premaxilla (MNHN SAM 129), drawing in ventral view **E** *E.cherifiensis* premaxilla FSAC-KK 10 **F** *E. cherifiensis* occipital piece (MNHN-MRS 340-25) **G** *E. cherifiensis* lower jaw reconstructed from several specimens (MNHN-E 43, SAM 138, SAM 137-157; redrawn from Lapparent de Broin 2002) **H** *E.cherifiensis* anterior dentary symphysis (MNHN E 43) in dorsal view **I** Partial dentary symphysis (MNHN-INA 25). Abbreviations: **bo** basioccipital, **d** dentary, **ex**, exoccipital **j** jugal **m** maxilla **pm** premaxilla **pt** pterygoid **sym** symphysis. Scale bars equal 10 cm in **A-C** and **E-G**, 20 cm in **H**.

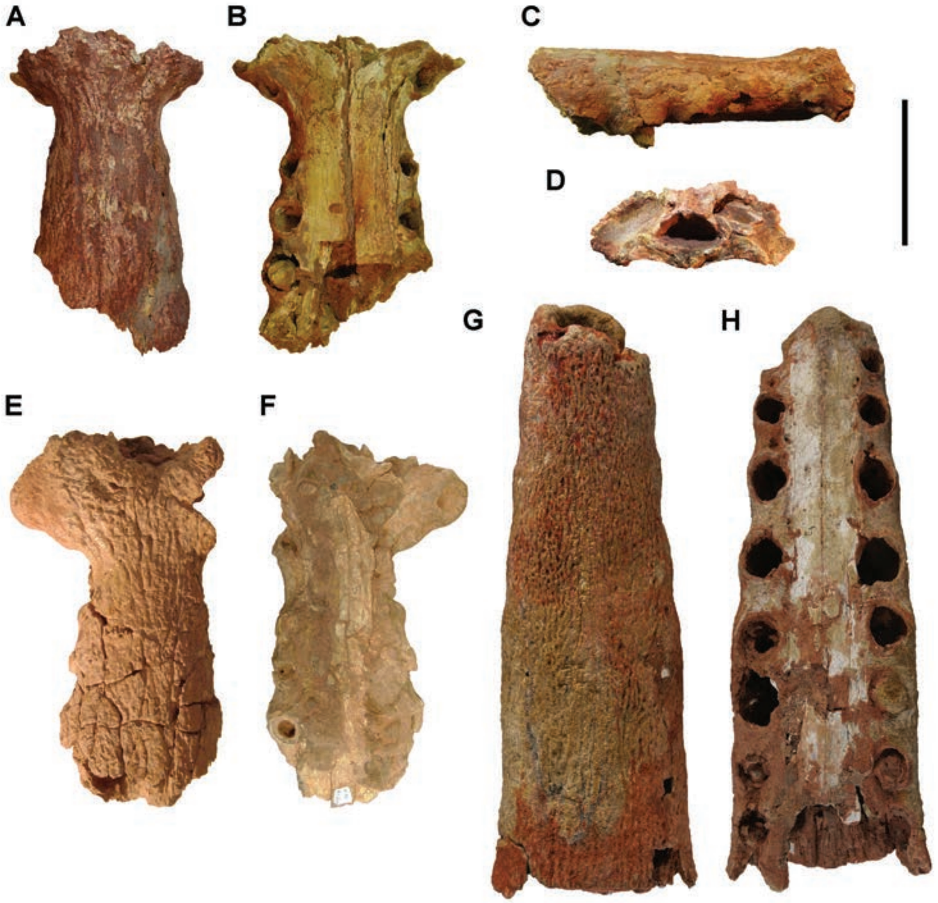

Figure 87. Rostral fragments referred to cf. *Elosuchus*. Rostral fragment (FSAC-KK 10) in (**A**) dorsal, (**B**) ventral, (**C**) right lateral and (**D**) anterior view. Rostral fragment (NMC 41866) in (**E**) dorsal and (**F**) ventral view. Maxillary piece (MNHN-MRS 3111) in (**G**) dorsal and (**H**) ventral view. Scale bar equals 10 cm.

cally identical isolated dentary fragments show the substantial range in size among material attributed to *E. cherifiensis* (UCRC PV159, CMN 41785, Fig. 89J, K). Meunier and Larsson (2017) noted some *Elosuchus* material from the Kem Kem differs from MNHN E 1, notably in the parietal bar separating the supratemporal fenestrae. MNHN E 1 and other material they refer to *E. cherifiensis* has a waisted bar, whereas other specimens have a broad, sculpted bar, similar to that of *E. broinae* (CMN 41912, ROM 52586, 64698, 64699). There may be more than a single species of *Elosuchus* within the Kem Kem Group (Meunier and Larsson 2017).

Neosuchia indeterminate. A large left jugal (FSAC-KK 07, Fig. 93) was found in situ in the Douira Formation at the locality Aferdou N'Chaft (Fig. 9, locality 14). With a preserved length of more than15 cm, the individual is approximately 150% the size of the adult cranium described by Larsson and Sues (2007: fig. 2; see also Fig.

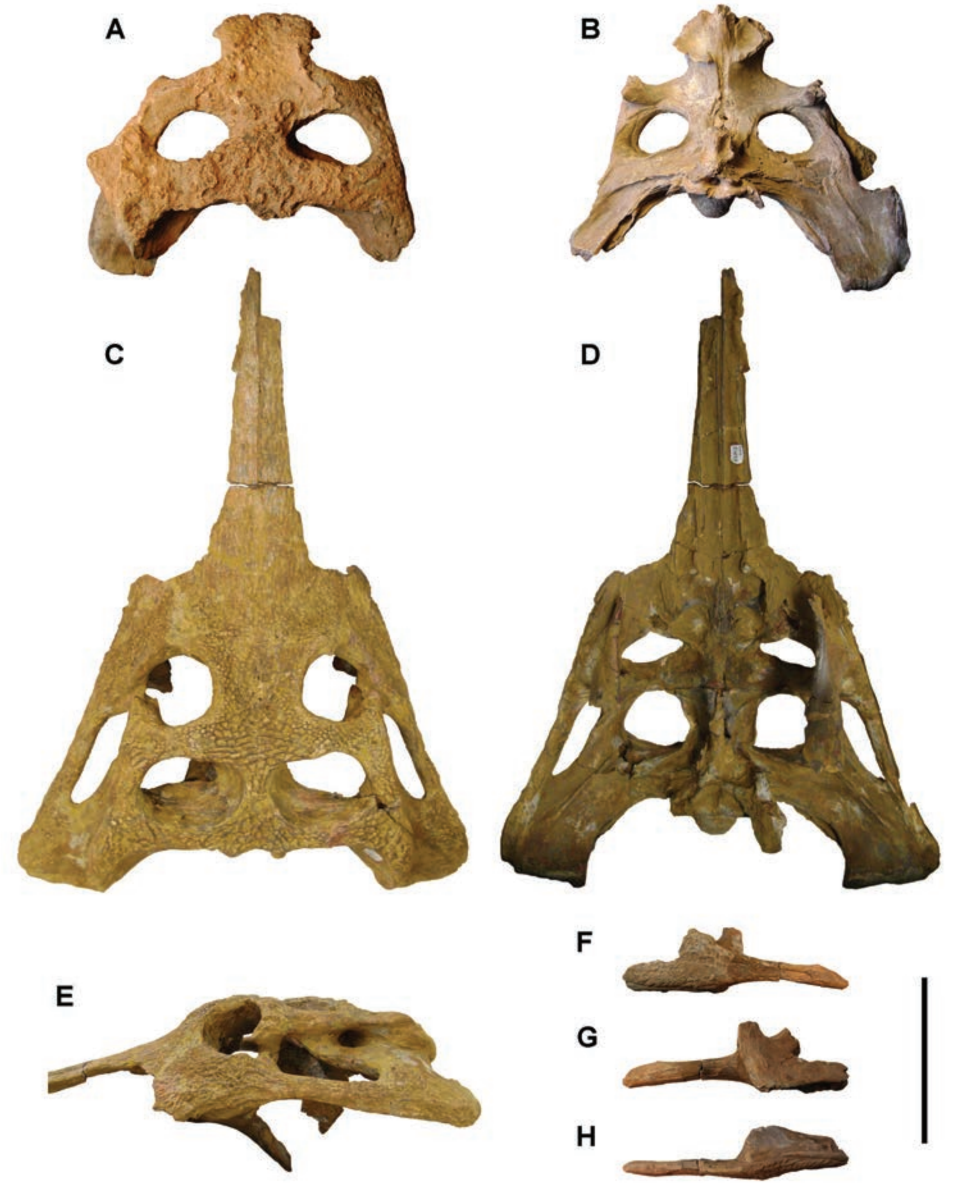

Figure 88. Braincase and jugal of the crocodyliform *Elosuchus cherifiensis*. Braincase (MNHN-MRS 3115) in (**A**) dorsal and (**B**) ventral view. Braincase (NMC 41912) in (**C**) dorsal, (**D**) ventral and (**E**) left lateral view. Jugal (FSAC-KK 09) in (**F**) left lateral, (**G**) medial and (**H**) ventral view. Scale bar equals 20 cm.

75A). The postorbital bar, which is smooth and inset in neosuchians, appears to be less inset from the sculpted surface of the jugal in FSAC-KK 07 than in *H. rebouli* (Larsson and Sues 2007). The posterior ramus, in addition, tapers distally and is deflected medi-

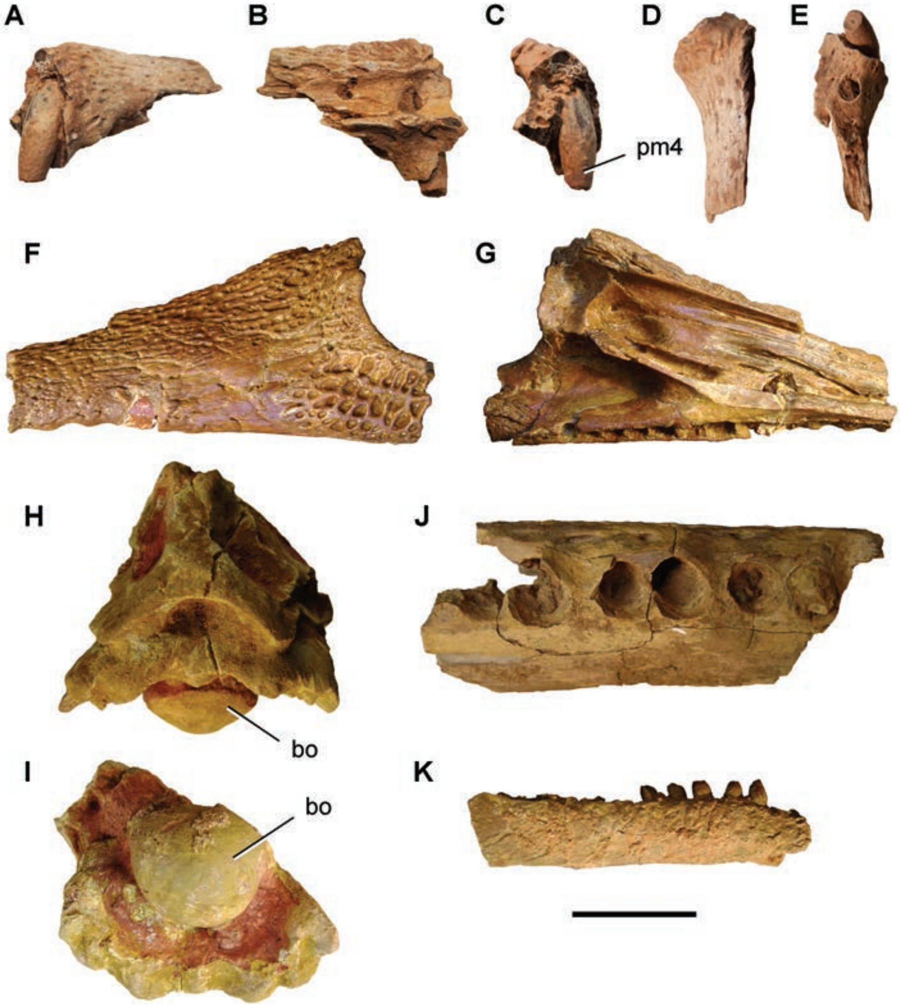

Figure 89. Fragmentary bones tentatively referred to the crocodyliform *Elosuchus*. Left premaxilla (FSAC-KK 923) in (**A**) lateral, (**B**) medial, (**C**) anterior, (**D**) dorsal and (**E**) ventral view. ?Lacrimal (BSPG 2008 I 60) in (**F**) lateral and (**G**) medial view. Basioccipital and basisphenoid (FSAC-KK 926) in (**H**) ventral and (**I**) posterior view. Left dentary fragment (UCRC PV168) in (**J**) dorsal view. **K** Left dentary fragment with five teeth (NMC 41785) in lateral view. Scale bar equals 5 cm. Abbreviations: **bo** basioccipital **pm4** premaxillary tooth 4.

ally (Fig. 93A, C). In *H. rebouli*, in contrast, the posterior process of the jugal expands slightly in dorsoventral height and is deflected slightly laterally.

Four foramina are visible on medial aspect of the body of the jugal (Fig. 93D). In extant crocodilians, one to three foramina open into the body of the jugal for the jugal nerve. The condition in *H. rebouli* is not known. Two other foramina pierce the large jugal near the ventral margin (Fig. 93C).

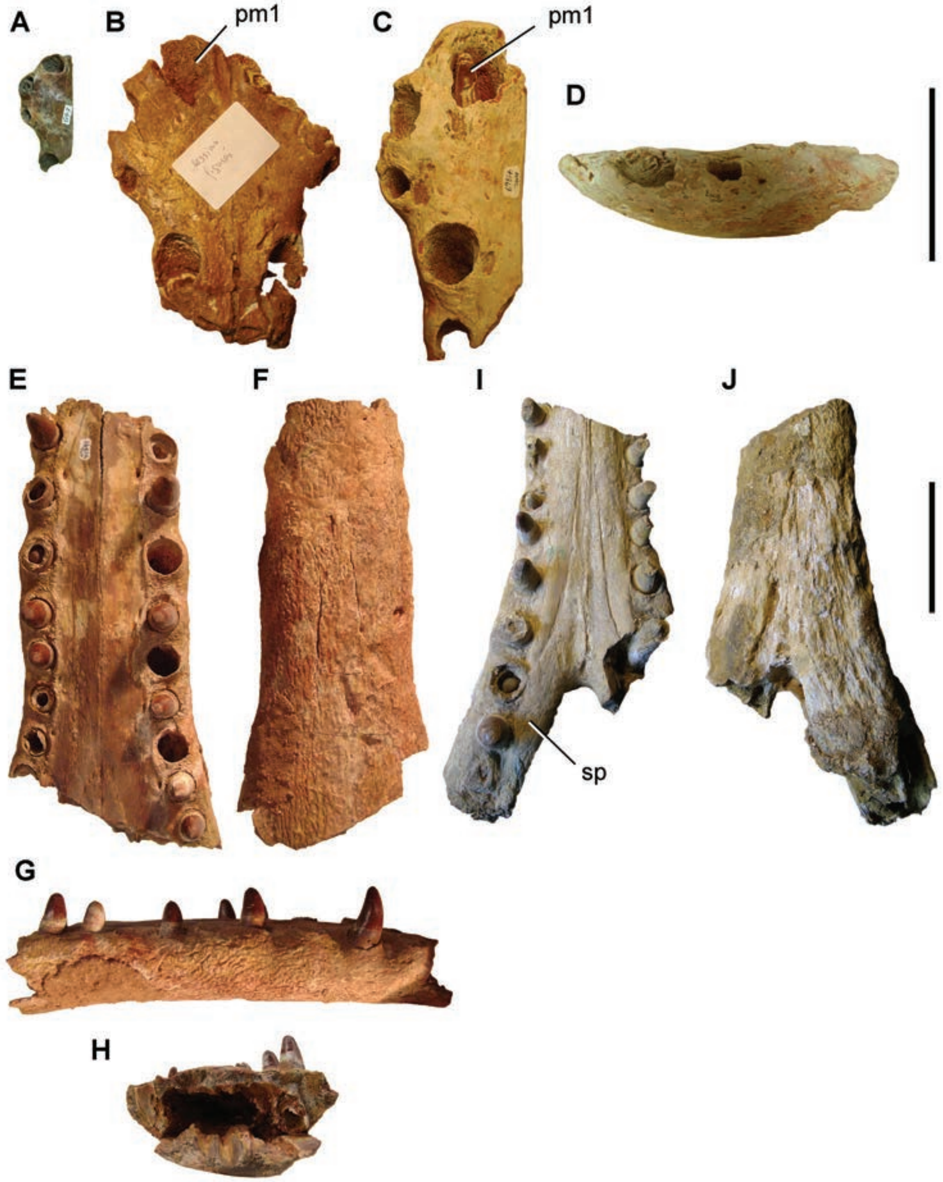

Figure 90. Dentaries of the crocodyliform *Elosuchus cherifiensis*. **A** Left anterior dentary (UCRC PV169) in dorsal view **B** Dentary symphysis (E 44 (MNHN; Lapparent de Broin 2002) in dorsal view **C**, **D** Left anterior dentary (NMC 41867) in dorsal and left lateral views **E-H** Dentary symphysis (NMC 41791) in dorsal, ventral, right lateral and anterior view. **I**, **J** Dentary and splenial symphysis (MNHN-MRS 3112) in dorsal and ventral view. Scale bars equal 10 cm. Abbreviations: **pm1** premaxillary tooth 1 **sp** splenial.

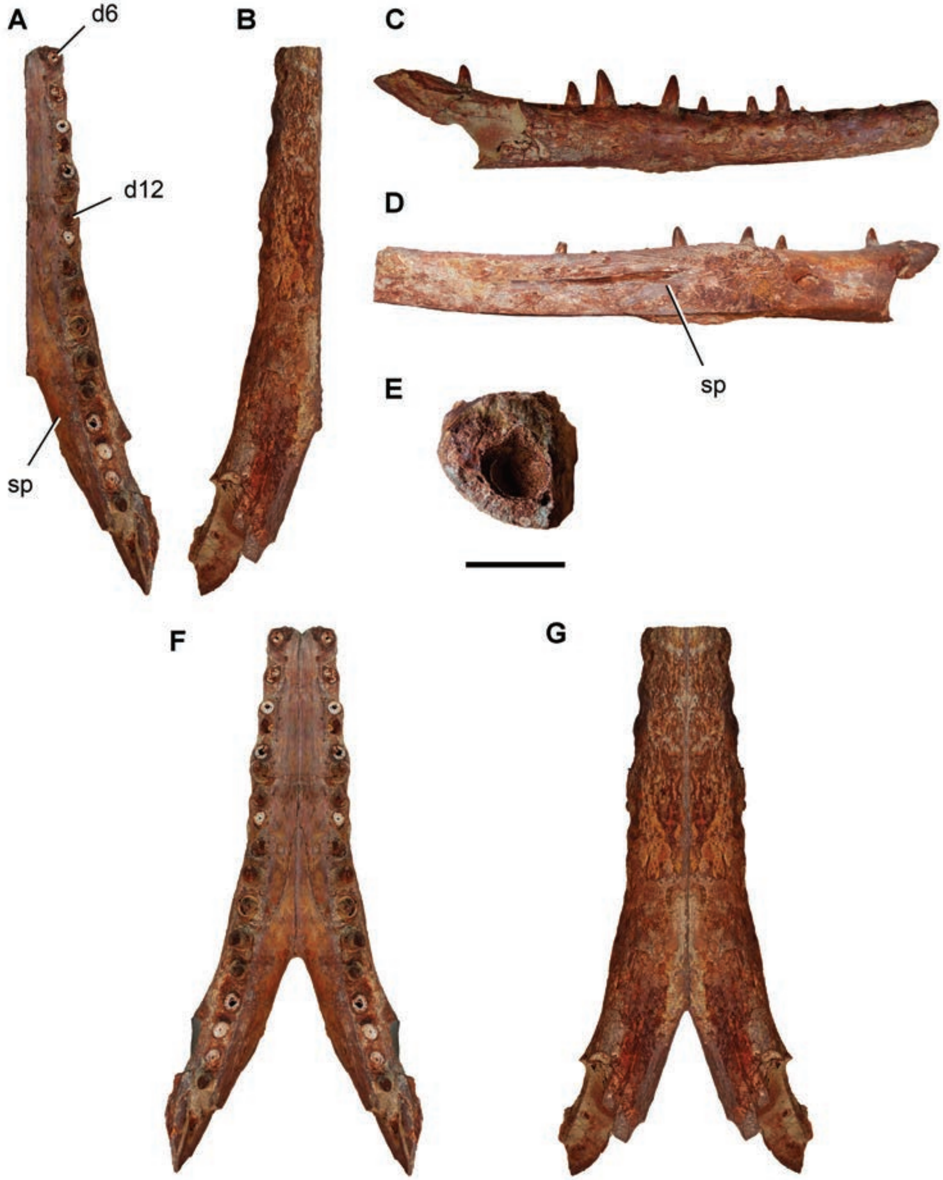

Figure 91. Mandibular symphysis of the crocodyliform *Elosuchus cherifiensis*. Right anterior dentary (FSAC-KK 753) in (**A**) dorsal, (**B**) ventral, (**C**) right lateral, (**D**) medial and (**E**) anterior view. Reconstructed symphysis of *Elosuchus cherifiensis* using reflected image of FSAC-KK 753 in (**F**) dorsal and (**G**) ventral view. Scale bar equals 5 cm in **A-D**, **F** and **G**, and 2 cm in **E**. Abbreviations: **d6**, **12** dentary tooth 6, 12 **sp** splenial.

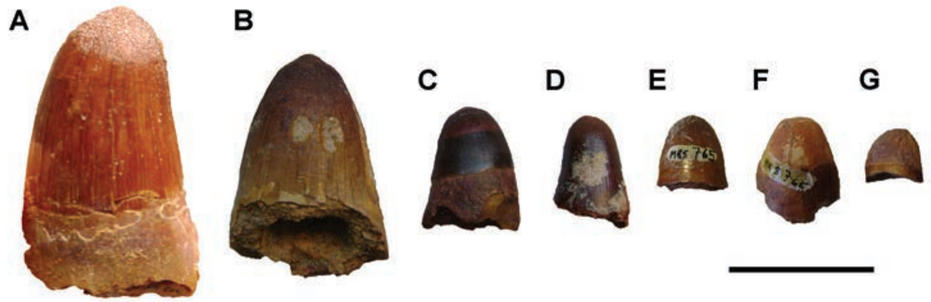

Figure 92. Isolated pholidosaurid crocodyliform teeth. **A** NMC 41791 (see Fig. 88E) **B**-**D** BSPG 1993 IX 334 **E** MNHN-MRS 765 **F** MNHN-MRS 766 **G** MNHN-MRS 767. Scale bar equals 3 cm.

Pterosauria Kaup, 1834

Although not recognised as pterosaurian at the time, the first pterosaur remains to be recovered from the Kem Kem Group consisted of isolated teeth collected by Lavocat in the late 1940s and early 1950s and now in the MNHN collections. Isolated, recurved, striated pterosaur teeth, likely ornithocheirid in most cases, are fairly common in these deposits. Kellner and Mader (1997: fig. 4) were the first to figure one of these teeth, four distinct morphotypes were described by Wellnhofer and Buffetaut (1999: figs 6–10) and additional examples are figured here (Fig. 94).

The first remains to be confidently identified as pterosaurian, an elongate mid-cervical vertebra referred to the Azhdarchidae, was described in a short abstract by Kellner and Mader (1996) and later figured by Rodrigues et al. (2011: fig 4). The following year, in another abstract, Mader and Kellner (1997) briefly described a jaw fragment with teeth (LINHM 016, Fig. 95A, B). Formally described by these authors in 1999, this specimen formed the holotype of *Siroccopteryx moroccensis*, a colobo-rhynchine, and the first pterosaur to be named from the Kem Kem Group. Recently, Jacobs et al. (2019) named a second coloborhynchine from the Kem Kem Group, *Coloborhynchus fluviferox*, and reported jaw fragments attributable to *Anhanguera* sp. and *Ornithocheirus* sp. (Jacobs et al. 2020).

Wellnhofer and Buffetaut (1999) published the first direct evidence for edentulous pterosaurs in the Kem Kem group. This included a well preserved but fragmentary rostrum (BSP 1993 IX 338, Fig. 96A-E) identified as pteranodontid, a fragment of a mandibular symphysis bearing a deep ventral crest (BSP 1997 I 67, Fig 97), identified as tapejarid and an elongate, lance-shaped fragment of a mandibular symphysis (BSP 1996 I 36; Wellnhofer and Buffetaut 1999: fig. 4) identified as azhdarchid. The latter, and BSP 1993 IX 338, were subsequently assigned to *Alanqa saharica* (Fig. 96; Ibrahim et al. 2010). Recently, however, BSP 1993 IX 338 has been reassigned to a new chaoyangopterid from the Kem Kem Group (McPhee et al. 2020) The identification of BSP 1997 I 67 as tapejarid is supported by the recent discovery of further tapejarid jaw material in the Kem Kem Group (Martill et al. 2020). Kellner et al. (2007) described

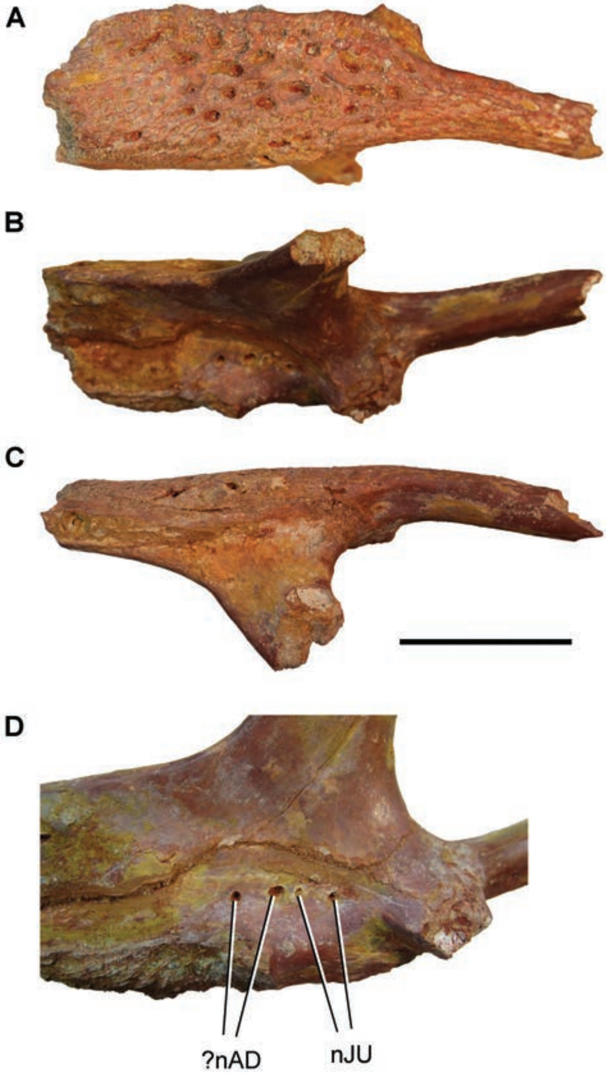

Figure 93. Sebecid left jugal (FSAC-KK 07) in (**A**) lateral, (**B**, **D**) medial and (**C**) ventral view. Scale bar equals 5 cm in **A–C**. Abbreviations: **nAD** dorsal alveolar branch of the maxillary nerve (V_2) **nJU** jugal branch of the maxillary nerve (V_2).

the fourth example of an edentulous jaw from the Kem Kem Group (MN 7054-V) and tentatively identified is as pteranodontid.

Alanqa saharica, an azhdarchid founded on a well-preserved fragment of a mandibular symphysis (FSAC KK 26; Fig 98) collected in 2008, and reinterpreted here as part of the rostrum was the first edentulous Kem Kem Group pterosaur to be named (Ibrahim et al. 2010). Additional remains, including fragments of the rostrum and mandibular symphysis (Kellner et al. 2007, Ibrahim et al. 2010, Martill and Ibrahim

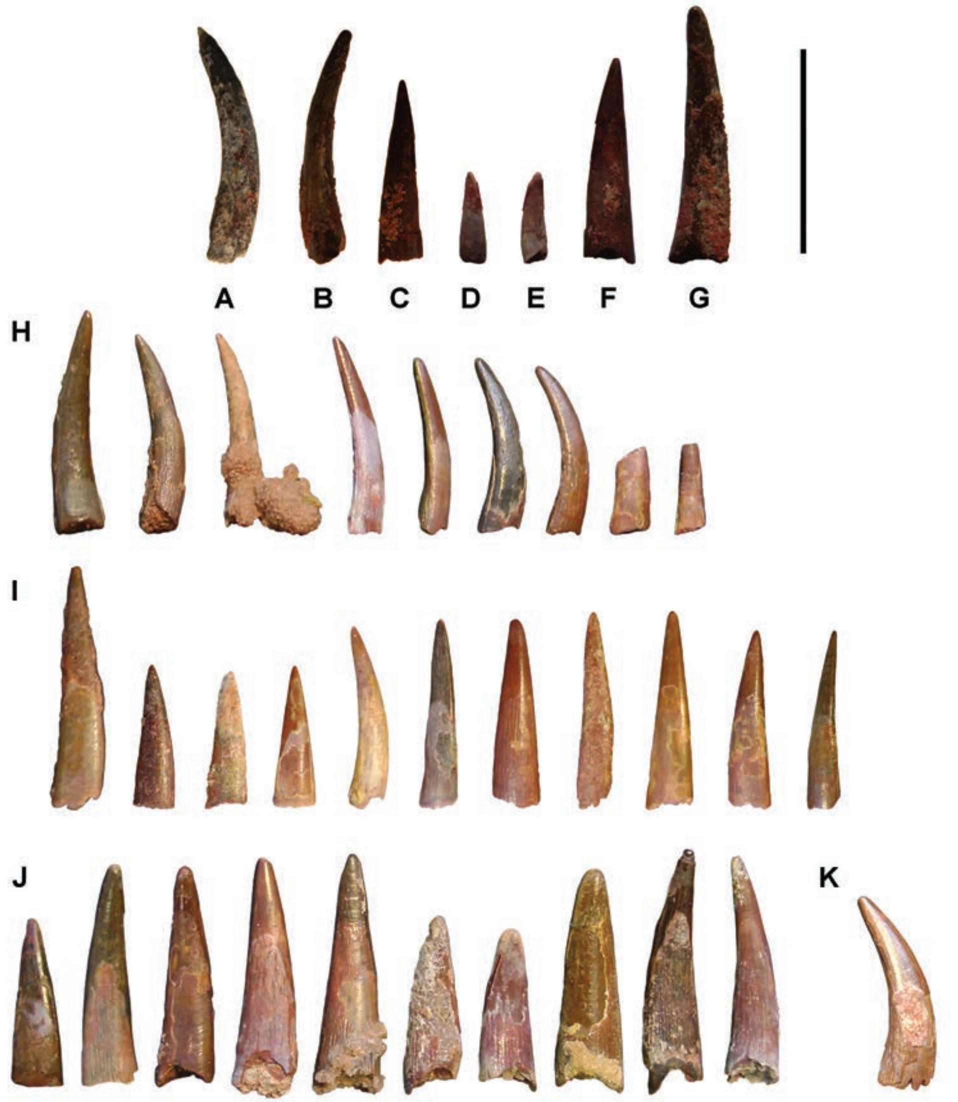

Figure 94. Ornithocheirid pterosaur teeth from the Kem Kem Group. **A** FSAC-KK 885 **B** FSAC-KK 44 **C** FSAC-KK 886 **D**, **E** FSAC-KK 197 in labial and lingual views **F** FSAC-KK 887 **G** FSAC-KK 941 **H** Teeth of 'morphotype 1' of Wellnhofer and Buffetaut (1999) (BSPG 1993 IX 590-596) **I** Teeth of 'morphotype 2' of Wellnhofer and Buffetaut (1999) (BSPG 1993 IX 597-607) **J** Teeth of 'morphotype 3' of Wellnhofer and Buffetaut (1999) (BSPG 1993 IX 608-617) **K** Tooth of 'morphotype 4' of Wellnhofer and Buffetaut (1999) (BSPG 1993 IX 618). Scale bar equals 3 cm.

2015) and, more tentatively, cervical vertebrae (Ibrahim et al. 2010, Rodrigues et al. 2011) and a partial humerus (Rodrigues et al. 2011) have been assigned to this taxon (Averianov 2014). A second azhdarchid, *Xericeps curvirostris*, founded on a fragmentary, elongate, curved mandibular symphysis was described by Martill et al. in 2018

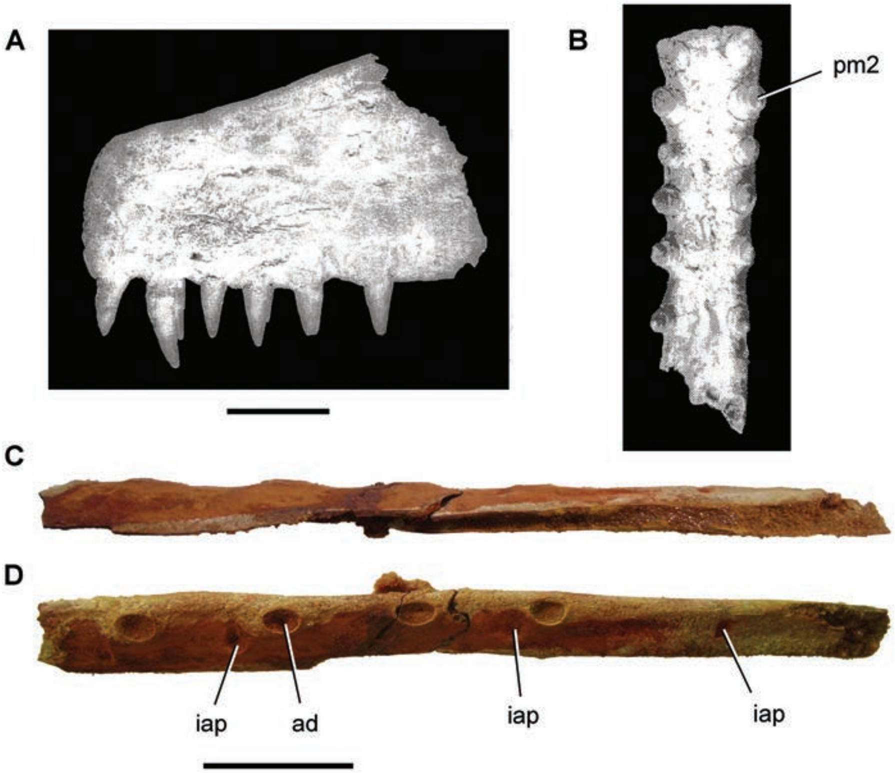

Figure 95. Ornithocheirid pterosaur jaw fragments from the Kem Kem Group. Premaxillae of *Siroccopteryx moroccensis* in (**A**) left lateral and (**B**) ventral views (from Mader and Kellner 1999). Dentary ramus of an indeterminate ornithocheirid (FSAC-KK 33) in (**C**) ?left lateral and (**D**) ?dorsal view. Scale bars equal 3 cm. Abbreviations: **ad** alveolus for dentary tooth **iap** interalveolar pit **pm2** premaxillary tooth 2.

(Fig. 99). Following the erection of this taxon, the second azhdarchid from the Kem Kem Group, it can no longer be automatically assumed that azhdarchid postcranial remains from these deposits pertain only to *Alanqa*.

All of the Kem Kem Group pterosaur material consists of isolated, often fragmentary specimens (Figs 94–102). Some originate from commercial sources and lack precise locality data. In numerical terms, teeth (Fig. 94) far outnumber skeletal remains, with many hundreds, and possibly more than one thousand already recovered. Among skeletal remains, fragments of the rostrum and mandibular symphysis of edentulous pterosaurs (Figs 96–101), seemingly all azhdarchoids, outnumber all other skeletal elements described so far. The relatively common occurrence of azdarchoid jaw remains is in sharp contrast to the comparatively rare incidence of ornithocheirid jaw fragments which, to date, number only six examples (Mader and Kellner 1999, Jacobs et al. 2019, 2020). This disparity is perhaps due to the unusually robust construction of the jaws of azhdarchoids in which the tips of rostra and mandibular symphyses are composed of relatively thick cortical bone with only a small central lumen (Fig. 101D).

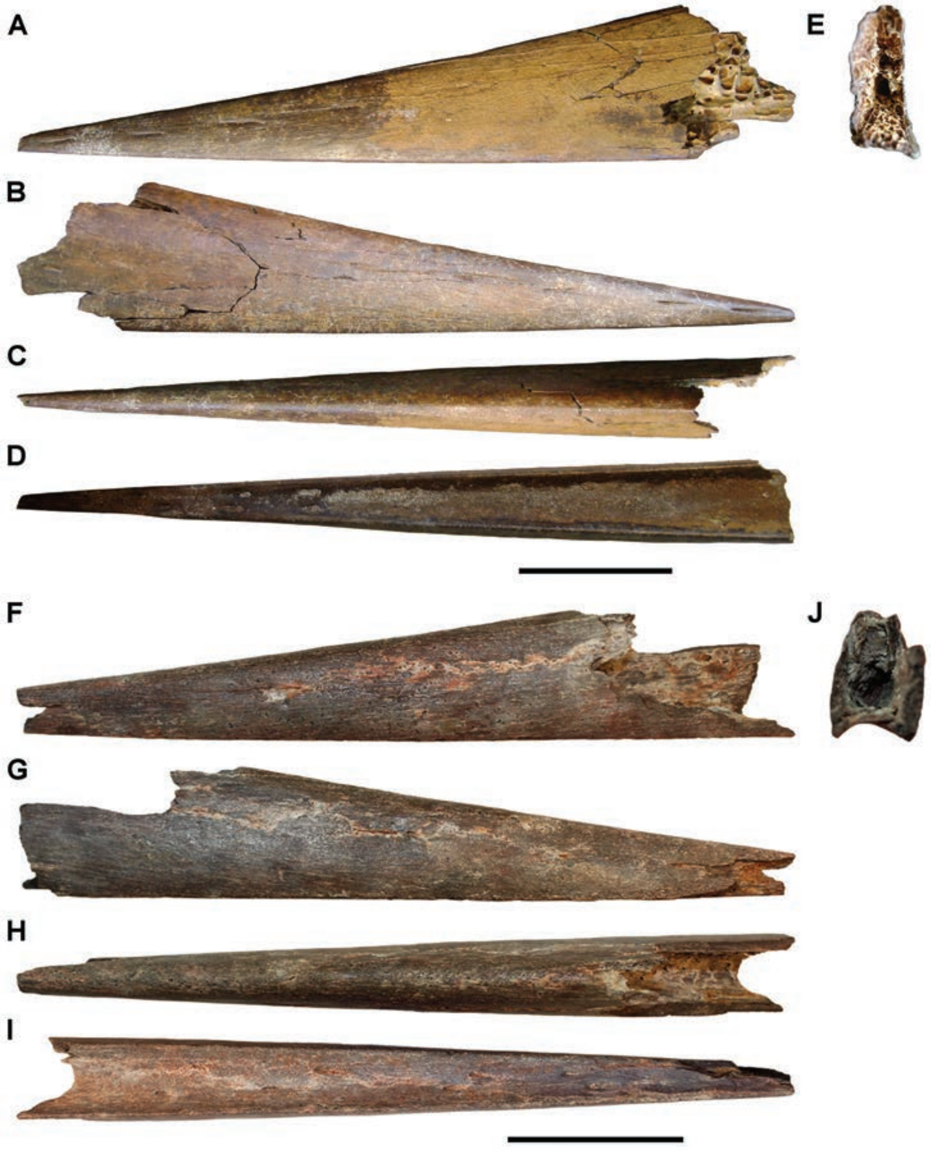

Figure 96. Rostral fragment (BSP 1993 IX 338) referred by McPhee et al. (2020) to a new ?chaoyangopterid azhdarchoid from the Kem Kem Group in (**A**) left lateral, (**B**) right lateral, (**C**) dorsal, (**D**) ventral and (**E**) posterior view. Fragmentary azhdarchoid rostrum (FSAC-KK 27) in (**F**) left lateral, (**G**) right lateral, (**H**) dorsal, (**I**) ventral and (**J**) posterior view. Scale bars equal 5 cm in **A-E**, 3 cm in **F-J**.

Postcranial remains including cervical vertebrae (Fig. 102; Ibrahim et al. 2010, Rodrigues et al. 2011) and limb bones (Rodrigues et al. 2011) are relatively rare and fragmentary, but often well preserved, undistorted and exhibit fine anatomical detail. Recently collected material, seemingly all referable to azhdarchoids and including ad-

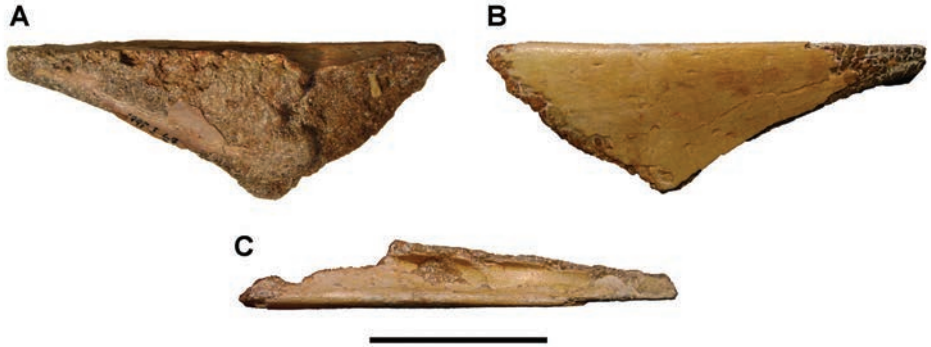

Figure 97. Fragment of the mandibular symphysis of a tapejarid pterosaur (BSP 1997 I 67) from the Kem Kem Group in (**A**) ?left lateral, (**B**) ?right lateral and (**C**) dorsal (occlusal) view. Scale bar equals 5 cm.

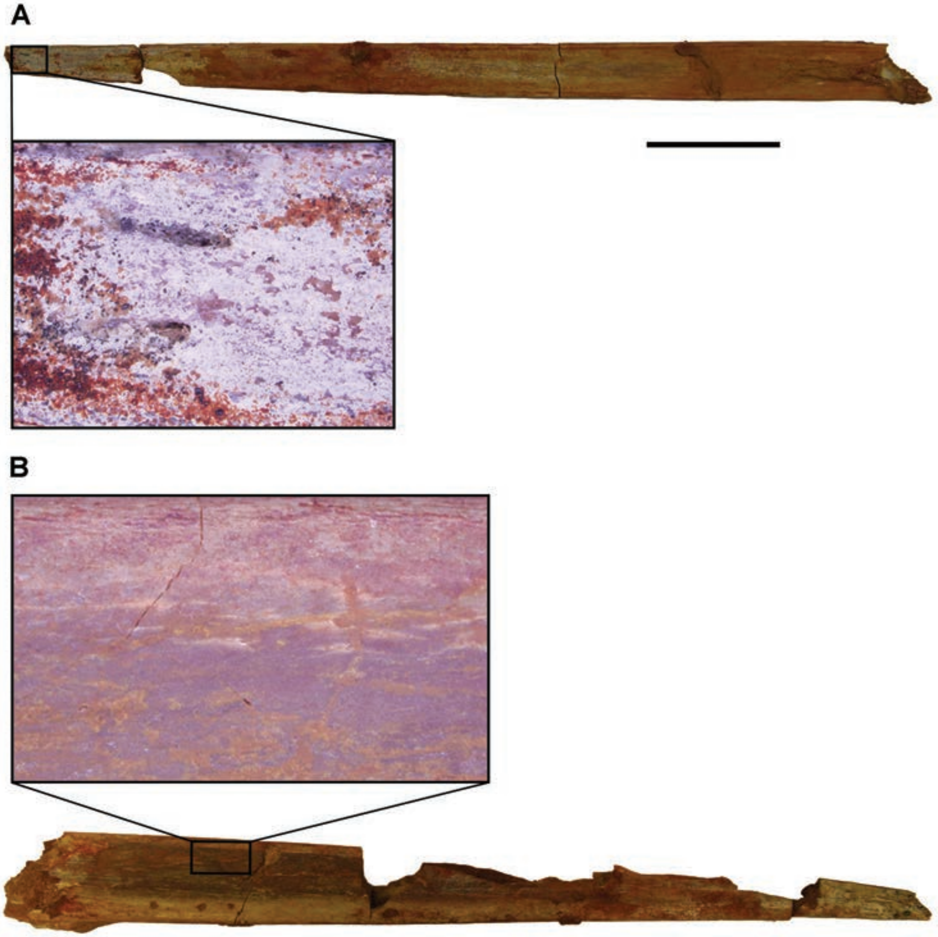

Figure 98. Rostrum of *Alanqa saharica* (FSAC-KK 26) from the Kem Kem Group in (**A**) ventral and (**B**) lateral views with magnified view of foramina and ventral margin. Scale bar equals 5 cm.

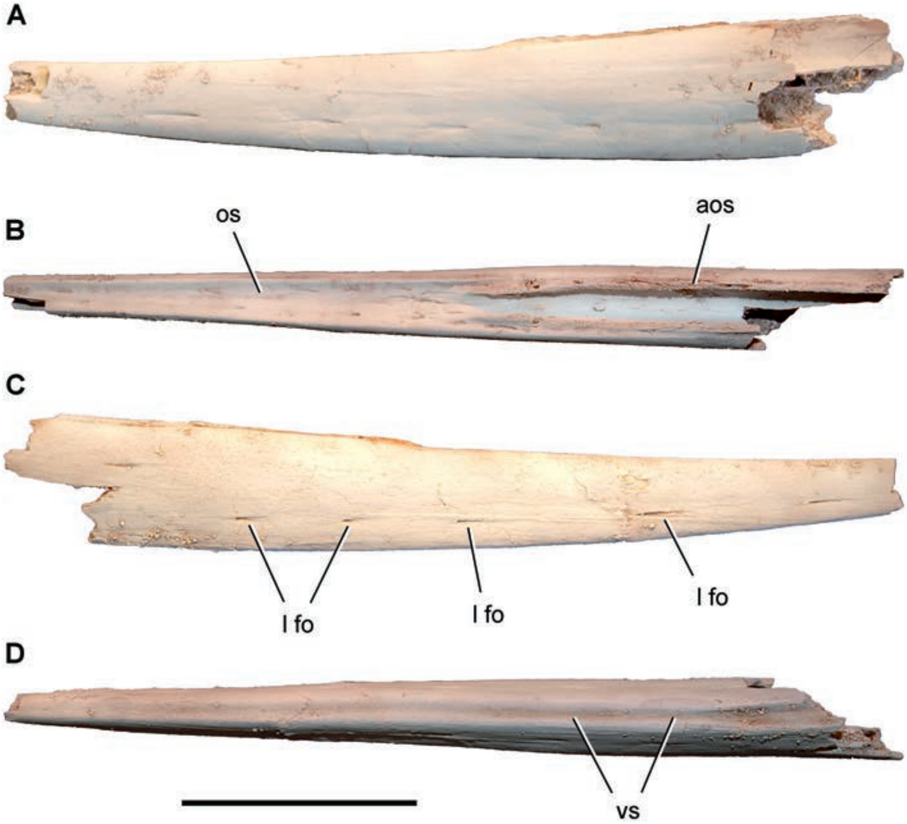

Figure 99. Fragment, coated in ammonium chloride, of the mandibular symphysis of *Xericeps curvirostris*, Martill et al. 2018, FSAC-KK 10700 in (**A**) left lateral, (**B**), occlusal, (**C**), right lateral and (**D**), ventral views. Reproduced from Martill et al. 2018. Abbreviations: **os** occlusal surface **aos** accessory occlusal surface **l fo** lateral foramina **vs** ventral sulcus. Scale bar equals 50 mm.

ditional cervicals, forelimb elements (humerus, ulna, wing-metacarpal, wing-phalanges), and hind limb elements (femur, tibia), has yet to be described.

Ornithocheiroidea Seeley, 1891
Ornithocheiridae Seeley, 1870

Siroccopteryx. *Siroccopteryx moroccensis* Mader & Kellner, 1999, based on the anterior portion of a rostrum that retains teeth (LINHM 016, Fig. 95A, B), was found near Begaa, a village close to the north end of the Kem Kem Hamada, according to the private collector involved in the sale of the material (Mader and Kellner 1999). The holotype, bearing the anteriomost six pairs of teeth, consists of coossified premaxillae and possibly a small portion of the anterior ends of the maxillae. The original description (Mader and Kellner 1999) has been supplemented by additional observations in more recent studies (Unwin 2001, Veldmeijer 2003, Rodrigues and Kellner 2008). The anterior profile measures approximately 30 mm in width at the base and has a height,

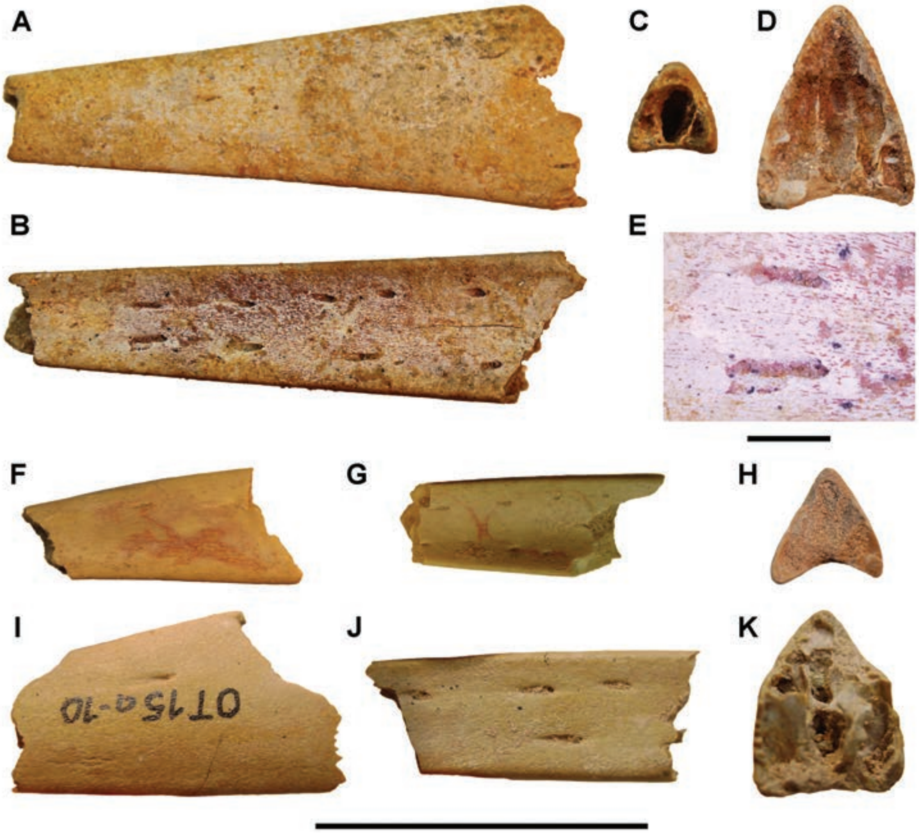

Figure 100. Fragments of jaws of a ?chaoyangopterid pterosaur from the Kem Kem Group. FSAC-KK 29 in (**A**) left lateral, (**B**) ?dorsal, (**C**) anterior and (**D**) posterior views **E** Detailed view of anterior-most paired foramina. FSAC-KK 32 in (**F**) left lateral, (**G**) ?dorsal and (**H**) posterior view. UCRC PV161 in (**I**) left lateral, (**J**) ?dorsal and (**K**) posterior view. Scale bar equals 5 cm in **A-D** and 4 cm in **F-K**. **E** scale bar equals 5 mm.

to the apex, of 43 mm. A rostrum of these general dimensions corresponds in size to large ornithocheirids with wingspans of 3–4 m (Martill and Unwin 2012).

Several authors have suggested that *Siroccopteryx* may be synonymous with *Coloborhynchus* (Unwin 2001, Veldmeijer 2003, Ibrahim et al. 2010). Conversely, Rodrigues and Kellner (2008) and Jacobs et al. (2019) have argued that it represents a genus distinct from other ornithocheirids. LINHM 016 exhibits several distinctive features including an unexpanded, near parallel profile, in palatal view (Fig 95B), a relatively narrow, deep rostrum, a rounded anterior profile of the rostrum in lateral aspect (Fig 95A) and a posteriorly displaced rostral crest. In combination these characters appear to distinguish *Sirocccopteryx* from coloborhynchines, typified for example by *Coloborhynchus clavirostris* (Owen, 1874) and *Uktenadactylus* (*Coloborhynchus*) *wadleighi* (Lee

1994), and ornithocheirids more generally. Pending the discovery of more complete remains, *Siroccopteryx* is retained here as a distinct taxon.

Coloborhynchus. *Coloborhynchus fluviferox* Jacobs et al. 2019, based on the anteriormost portion of a rostrum bearing two pairs of teeth (FSAC-KK 10701), likely from Aferdou N'Chaft, appears to represent a second ornithocheirid from the Kem Kem Group. The holotype and only known specimen exhibits typical coloborhynchine characters such as the development of a tall, triangular, vertically reflected palatal surface on the anterior termination of the rostrum, pierced by the first pair of dental alveoli, and a tall, narrow rostral crest that rises directly from the anteriormost tip of this surface. This rostral fragment is somewhat larger than the holotype of *Siroccopteryx moroccensis* and, based on comparison with other more complete remains of ornithocheirids (Martill and Unwin 2012), likely represents a relatively large individual at least 4 m in wingspan.

Ornithocheiridae indet. A partial mandibular ramus 160 mm in length and bearing four dental alveoli was collected from Aferdou N'Chaft from the Gara Sbaa Formation (FSAC-KK 33, Fig. 95C, D). Caudal to the posteriormost dental alveolus, likely the last in the series, the dorsal surface of the mandibular ramus is smooth and rounded. Small pits may have accommodated the tips of teeth located in the rostrum (cf. Myers 2010), and suggest that the upper tooth row extended further posteriorly than the lower tooth row. Comparison with more complete material of *Anhanguera* (Wellnhofer 1985, 1991) and *Coloborhynchus* (Kellner and Tomida 2000, Veldmeijer 2003) suggest that, when complete, the mandibles were approximately 600 mm in length, representative of a large ornithocheirid 4–5 m in wingspan. Until Kem Kem ornithocheirids are better understood it is not possible to determine whether this fragment pertains to the two named coloborhynchines, or a third ornithocheirid. Jacobs et al (2020) recently described a mandible tip that they referred to *Anhanguera*. In addition, they figured two isolated rostral tips that they referred to, respectively, *Ornithocheirus* and a species of *Coloborhynchus* distinct from *C. fulviferox*.

Isolated, often incomplete, ornithocheirid teeth have been described by Kellner and Mader (1997) and Wellnhofer and Buffetaut (1999). They are present in several collections (MNHN, UCRC, FSAC) and have been recovered by the authors from multiple localities (Boumerade, Gara Sbaa, Zguilma, Aferdou N'Chaft, Taouz, and Jorf). Tooth crowns often show apical wear and the absence, in many cases, of a root suggests that many of these teeth may have been shed after some root resorption (Fig. 94).

All teeth recovered to date can be assigned to one of the four morphotypes recognized by Wellnhofer and Buffetaut (1999). They include: slender and recurved crowns with an oval cross-section near the tip (morphotype I, Fig. 94H); slender, flattened, gently recurved crowns (morphotype II, Fig. 94I); robust, wide-based, nearly straight crowns with two carinae (morphotype III, Fig. 94C, J); and very robust, large, recurved crowns approximately 35 mm in length (morphotype IV, Fig. 94K). Tooth morphology and size can vary quite considerably along the tooth row in ornithocheirids (e.g. Campos and Kellner 1985, Wellnhofer 1985, 1991, Kellner and Tomida 2000, Frey et al. 2003, Veldmeijer 2003, Wang et al. 2012, 2014). Moreover, this variation was

likely compounded by allometric shape changes related to ontogeny, with hatchlings of 0.3–0.4 m wingspan (Unwin and Deeming 2019) achieving sizes, at maturity, of 4 m or more, an order of magnitude larger. Even so, it is difficult to accommodate all four tooth morphotypes within the dentition of a single ornithocheirid and it seems likely that multiple species were present in the Kem Kem Group. This is consistent with the recognition of four distinct genera, *Siroccopteryx*, *Coloborhychus*, *Anhanguera* and *Ornithocheirus*, but assignment of individual teeth to these, or other Kem Kem ornithocheirids (Jacobs et al. 2020) will require further work.

Azhdarchoidea Nessov, 1984
Tapejaridae Kellner, 1989

Tapejaridae indet. The anterior portion of an edentulous mandibular symphysis bearing a large ventral crest (BSP 1997 I 67) was described by Wellnhofer and Buffetaut (1999; Fig. 97). The jaw is Y-shaped in cross-section with rami that diverge only slightly posteriorly. Slit-shaped foramina are present on the external surfaces, and cancellous bone is exposed on broken surfaces. The ventral crest is large and subtriangular in shape, with a slightly concave antero-ventral margin. The fragment measures 118 mm in length and 16 mm in posterior width, with a maximum of 10 mm across the occlusal surface. Based on comparison with more completely known tapejarids such as *Sinopterus* from the Jiufotang Formation of China (Wang and Zhou 2003, Lü et al. 2006) it seems likely that BSP 1997 I 67 originally had a wingspan of around 3 m.

The morphology of the jaw and deep ventral crest corresponds well to that of tapejarids such as *Tapejara* (e.g., Vullo et al. 2012: fig. 5C). By contrast other edentulous pterosaurs either have a low mandibular crest (thalassodromeids) or none at all (pteranodontians, chaoyangopterids, azhdarchids) (Witton 2009, Vullo et al. 2012). Identification of BSP 1997 I 67 as tapejarid is supported by the recent discovery, in the Kem Kem group, of additional tapejarid material recently described by Martill et al. (2020). This new material assigned to a new taxon, *Afrotapejara zouhrii* Martill et al., 2020, suggests that a fragmentary rostrum, MN 7054-V, described by Kellner et al. (2007: fig. 1) is also tapejarid.

? Chaoyangopteridae

Apatorhamphus. A fragment of an edentulous rostrum missing its anterior tip (FSAC-KK 5010) collected at Aferdou N'Chaft has been made the holotype of a third genus and species of azhdarchoid, *Apatorhamphus gyrostega*, possibly a chaoyangopterid, from the Kem Kem Group (McPhee et al. 2020). McPhee et al. assigned several additional fragmentary rostra to *A. gyrostega* including FSAC-KK 5011, 5012 and 5013, BSP 1993 IX 338, originally identified as pteranodontid (Wellnhofer and Buffetaut 1999; Fig. 96A-E) and more recently as azhdarchid (Averianov et al. 2008, Ibrahim et al. 2010, Averianov 2014), and CMN 50895, originally determined by Rodrigues et al. (2011, fig1) as possibly a fragment of the mandibular symphysis of a dsungaripteroid. An additional specimen, FSAC-KK 5013, identified as a fragment of the mandibular symphysis, is almost perfectly complimentary to the holotype and tentatively assigned by McPhee et al. to this new taxon. Further jaw fragments including FSA-KK 27 (Fig.

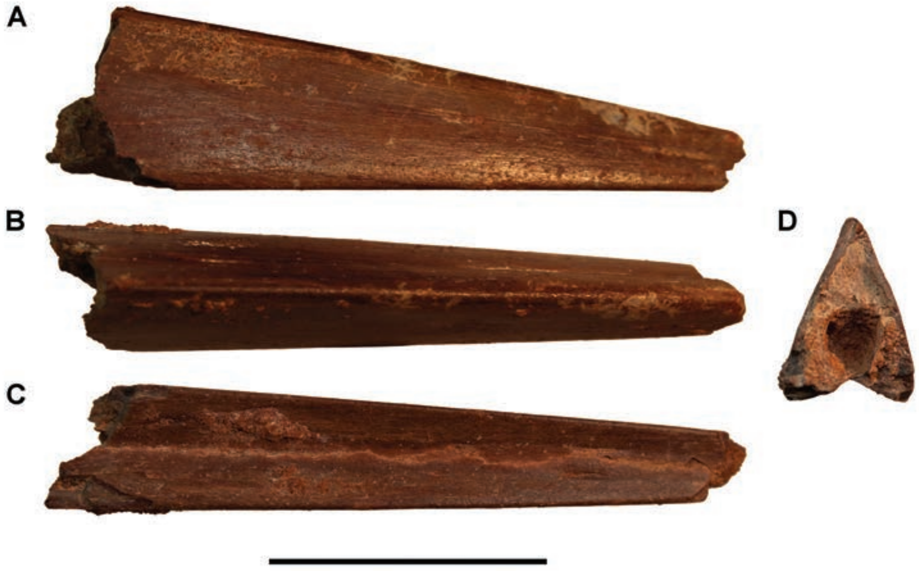

Figure 101. Azhdarchid pterosaur rostral fragment from the Kem Kem Group**.** FSAC-KK 28 in (**A**) lateral, (**B**) ?dorsal, (**C**) ?ventral and (**D**) posterior view. Scale bar equals 3 cm.

96F-J), FSAC-KK 29 (Fig 100A-E), FSAC-KK 32 (Fig 100F-H) and UCRC PV161 (Fig. 100I-K) collected in 1995 near Taouz, likely also pertain to this pterosaur.

The rostrum shows a relatively rapid increase in depth posteriorly and has a slightly concave dorsal profile, which is typical of chaoyangopterids, but not other azhdarchoids. The lateral and palatal surfaces bear prominent foramina and the palatal surface has well developed dental margins, but no median ridge. Posteriorly the bone walls of the rostrum are remarkably thin, but toward the tip they become much more robust enclosing a deep but increasingly narrow central lumen. Unlike azhdarchids, the jaws of which have a 'Y' shaped cross-section (Wellnhofer and Buffetaut 1999, fig. 4a; Ibrahim et al. 2010: fig. 2c, d), the jaws of this pterosaur have a rounded, sub-triangular cross-section (e.g., Fig. 100C, D, H, K). FSAC-KK 5010 is 211 mm long. Comparison with more complete remains of chaoyangopterids (e.g., Lü et al. 2008) suggests that the prenarial rostrum of this individual was at least 0.3 m in length, the skull at least 0.6 m long and the wingspan in the region of 3–4 m, or more.

A combination of features including the shape of the rostrum, its unusual cross-sectional profile and the shape and distribution of foramina appear to distinguish FSAC-KK 5010 from other edentulous taxa found in the Kem Kem group, and azhdarchoids more generally although, in the latter case, comparison is often hampered by severe compression of the skull remains, for example in taxa from the Crato and Jiufotang Formations. While FSAC-KK 5010 compares more closely to the rostrum of chaoyangopterids than to other azhdarchoids, the possibility that it might, for example, be thalassodromeid cannot be entirely excluded, hence the caution in assigning this new species to Chaoyangopteridae.

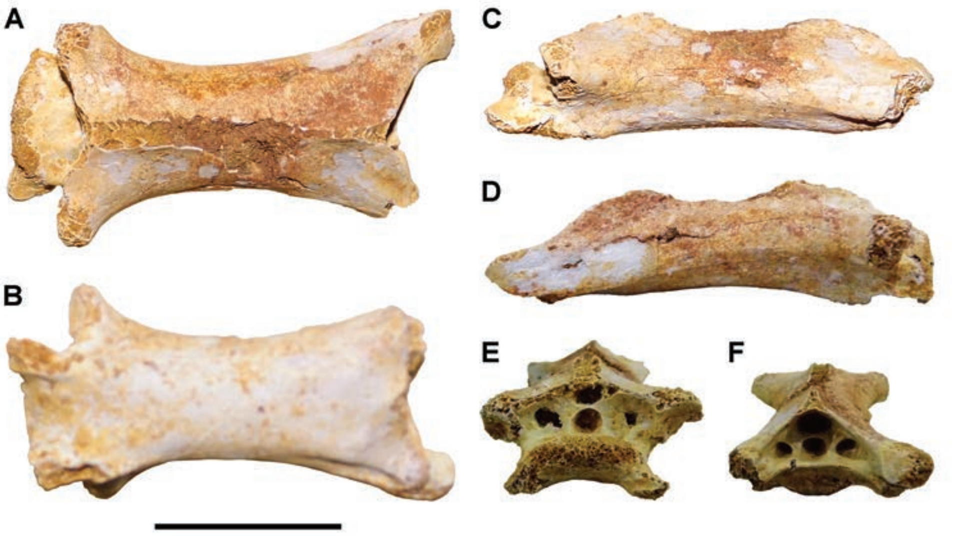

Figure 102. Near complete ?third cervical of an azhdarchid pterosaur from the Kem Kem Group**.** FSAC-KK 3088 in (**A**) ventral, (**B**) dorsal, (**C**) right lateral, (**D**) left lateral, (**E**) anterior and (**F**) posterior view. Scale bar equals 5 cm.

Azhdarchidae Nessov, 1984

Alanqa. *Alanqa saharica*, Ibrahim et al. 2010, was founded on a partial mandibular symphysis (FSAC-KK 26; Fig. 98), reinterpreted here as a rostrum, collected *in situ* in 2008 at Aferdou N'Chaft (Ibrahim et al. 2010). Other Kem Kem group remains that can be assigned to this taxon include BSP 1996 I 36 (Wellnhofer and Buffetaut 1999: fig. 4), CMN 50859 (Rodrigues et al. 2011: fig. 1), FSAC-KK 4000 (Martill and Ibrahim 2015, fig. 3), a specimen from a private collection (Martill and Ibrahim 2015: fig. 5) and FSAC-KK 28 (Fig. 101).

Fragments of the rostrum and mandibular symphysis of this pterosaur have been described in detail, and figured by Wellnhofer and Buffetaut (1999), Ibrahim et al. (2010), and Martill and Ibrahim (2015). *Alanqa saharica* is characterized by remarkably straight jaw margins and a pronounced boss on the palatal surface of the rostrum, which is matched by paired accessory facets on the occlusal surface of the mandibular symphysis (Martill and Ibrahim 2015: fig. 4f). The latter features have not been reported in any other pterosaur, with the exception of *Xericeps curvirostris* Martill et al. 2018, suggesting a close relationship between these taxa. The holotype, FSAC-KK 26, appears to belong to an individual with an estimated wingspan of 3–4 m (Ibrahim et al. 2010).

The only phylogenetic analysis to include *Alanqa* to date (Longrich et al. 2018) recovered this pterosaur as a thallassodromeid. However, this was based on the assumption that the holotype represented the mandibular symphysis, rather than the rostrum, as proposed here. Three features: highly elongate slender jaws with remarkably straight margins and an unusual 'Y' shaped cross-section, as found for example in *Quetzalcoatlus*

(Kellner and Langston 1996: fig. 7), suggest that *Alanqa* and its close relative, *Xericeps*, are members of Azhdarchidae.

Xericeps. *Xericeps curvirostris* Martill et al. 2018, is represented by a single fragment of a mandibular symphysis (FSAC-KK 10700) commercially collected from the Douira Formation at Aferdou N'Chaft (Fig. 99). The holotype, described in detail by Martill et al. 2018, is distinguished by its curvature in lateral view, with markedly concave dorsal and convex ventral margins. Unique to this pterosaur there is a pronounced midline groove on the ventral border of the mandibular symphysis. Comparison with more complete remains of azhdarchoids suggests that FSAC-KK 10700 was a large individual of at least 3–4 m in wingspan.

Among azhdarchoids the long slender mandibular symphysis of *Xericeps curvirostris* (Fig. 99) is most closely comparable to that of azhdarchids such as *Quetzalcoatlus* (Kellner and Langston 1996) and *Alanqa* (Ibrahim et al. 2010). Indeed, *Alanqa saharica* and *Xericeps curvirostris* share a seemingly unique feature: well developed paired accessory occlusal surfaces on the posterior portion of the dorsal surface of the mandibular symphysis (Martill and Ibrahim 2015, Martill et al. 2018). These similarities, and the co-occurence of the remains in the same deposit, raise the possibility that *A. saharica* and *X. curvirostris* (Table 8) might be synonymous. Jaw morphology can be quite variable in pterosaurs, as a result of ontogeny, sexual dimorphism (reflected in the presence or absence of crests) and natural variation (Averianov 2014). Moreover, the morphological variation subtended by FSAC-KK 26 and FSAC-KK 10700 falls within the range of variation exhibited by, for example, the jaws of *Rhamphorhynchus* (Bennett 1995). However, additional, more complete remains are needed to demonstrate, or discount, synonymy.

Azhdarchidae indet.

The Kem Kem Group has yielded several well-preserved cervical vertebrae comparable to those described for azhdarchids such as *Quetzalcoatlus* (Howse 1986), *Phosphatodraco* (Pereda-Suberbiola et al. 2003) and *Cryodrakon* (Hone et al. 2019). These include two highly elongate vertebrae (Kellner and Mader 1996, Rodrigues et al. 2011), likely the fourth or fifth in the cervical series, and a fragment of a much larger cervical representing an individual of at least 6 m in wingspan (Ibrahim et al. 2010: fig. 6). A second individual of comparable size is represented by a nearly complete cervical vertebra (FSAC-KK 3088; Fig. 102). This relatively short vertebra, approximately three times longer than wide, is most closely comparable in its proportions to the third cervical of *Phosphatodraco* (Pereda-Suberbiola et al. 2003). Additional azhdarchid vertebrae, some of very large size, have yet to be described.

Azhdarchoidea indet. Numerous limb bones including the humerus, ulna, wing-metacarpal, wing-phalanges, femur and tibia have been collected in recent years. So far, however, only a single humerus has been described (Rodrigues et al. 2011). The humerus was assigned by these authors to the Azhdarchoidea of which four species (a tapejarid, a ? chaoyangopterid and two azhdarchids) have now been described from the Kem Kem group. It is not clear, at present, to which if any of these taxa the humerus may belong. The same will likely apply to the many, as yet undescribed, limb bones of azhdarchoids.

Dinosauria Owen, 1842

Dinosaurs are represented by theropods and sauropods, as well as fragmentary remains of uncertain affinities, including a large ornithischian footprint (Kellner and Mader 1997, Novas et al. 2005a, Cavin et al. 2010, Ibrahim et al. 2016). Theropods are represented by abelisaurids (Russell 1996, Mahler 2005, Zitouni et al. 2019), spinosaurids (Buffetaut 1989, 1992, Russell 1996, Milner 2003, Dal Sasso et al. 2005, Ibrahim et al. 2014b), carcharodontosaurids (Russell 1996, Sereno et al. 1996), and the enigmatic *Deltadromeus* (Sereno et al. 1996). Sauropods include a rebbachisaurid (Lavocat 1954, Russell 1996, Wilson and Allain 2015) and a titanosaur (Russell 1996, Ibrahim et al. 2016).

Isolated and often fragmentary dinosaur bones and teeth are found in all major localities in both formations in the Kem Kem Group. Skull bones are rare and usually consist of jaw fragments, pieces of braincase, or the quadrate condyles. On rare occasions, partial skulls and associated and even articulated skeletons are preserved (Lavocat 1954, Sereno et al. 1996, Ibrahim et al. 2014b, Wilson and Allain 2015). The bones, teeth, and footprints of theropods are more common than those pertaining to sauropods and especially ornithischians.

Ornithischia Seeley, 1888

Ornithischian teeth and footprints are extremely rare in the Kem Kem Group; ornithischian cranial or postcranial bones have yet to be identified. Evidence from a single small isolated crown (Fig. 103) and a single large footprint (Sereno et al. 1996) indicate that small- and large-bodied ornithischians were at least transiently present.

A small partial subtriangular crown was recovered from Oum Tkout in the Douira Formation from a small-bodied ornithischian (Fig. 103). Both sides of the crown have similar enamel thickness and a broadly rounded primary ridge, which is slightly more prominent on the presumed labial side (Fig. 103A). There are no secondary ridges, enamel texture or wear facets. The denticles, which number six to each side of the large apical denticle, decrease in size toward the base of the crown, curve away from the crown midline, and terminate in blunt rounded tips. The axes of the denticles are angled ca. 45° to the crown axis. Near the fracture surface on the labial side (Fig. 103A), the enamel appears to curve away from the crown base, suggesting that a swollen cingulum may have joined the small basal denticles fore and aft.

The small size of the crown (~ 1 cm in height), symmetrical enamel, rounded form of the primary ridge, absence of secondary ridges, low number of denticles, basal cingulum and absence of tooth-to-tooth wear facets are consistent with the form and function of a basal ornithischian tooth, possibly from a thyreophoran. Among thyreophorans, ankylosaur and stegosaur teeth often have a similar subtriangular crown shape but usually differ in exhibiting more surface ornamentation, pointed denticles, a bulbous cingulum and tooth-to-tooth wear facets. Although ankylosaur teeth often show an arched divergence of the denticle axis present in the Kem Kem specimen, basal thyreophoran *Scutellosaurus* has crowns that are otherwise similar in shape and ornamentation and are often unworn (Colbert 1981). Thyreophorans are rare in the Cretaceous of

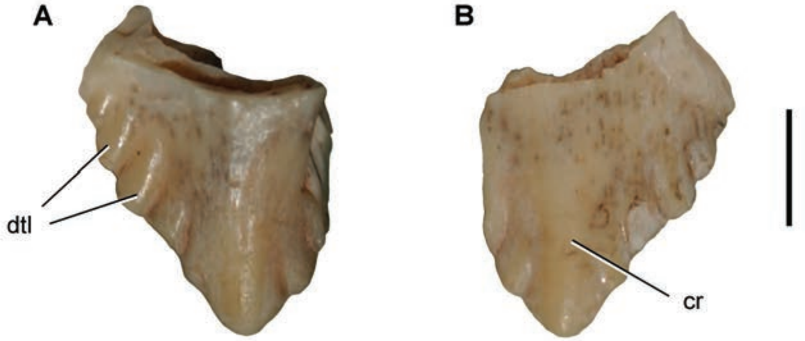

Figure 103. Possible thyreophoran tooth. UCRC PV162 unnumbered in (**A**) labial and (**B**) lingual view. Scale bar equals ~5 mm. Abbreviations: **cr** crown **dtl** denticles.

Africa but have been reported from the older Tiouraren Formation of Niger (Ridgwell and Sereno 2010). The unnamed Tiouraren thyreophoran has crowns that are generally similar to those in *Scutellosaurus*. The presence of such a simple, symmetrically enameled crown form in the Late Cretaceous is unusual and limited to thyreophorans.

A single large (~ 51 cm long and wide), clover-shaped, three-toed footprint records the presence of a large ornithischian, presumable a large iguanodontian, in the Douira Formation (Sereno et al. 1996, Ibrahim et al. 2014a). Ornithopod teeth of any form, however, have yet to be reported from the Kem Kem Group.

Sauropoda Marsh, 1878

The Kem Kem Group preserves sauropod remains, usually as rare isolated teeth and bone fragments. Only one associated partial postcranial specimen has been recovered (Figs 104, 105), that of the diplodocoid *Rebbachisaurus garasbae* from the Gara Sbaa Formation (Lavocat 1954b, Wilson and Allain 2015). Fragmentary pieces of the fragile rebbachisaurid neural arches, centra, and cylindrical sauropod teeth occur at multiple localities (Figs 106–108). The proximal ends of a humerus and ulna (Fig. 109) from large-bodied titanosaurs were recovered in the Douira and Gara Sbaa formations, respectively (Sereno et al. 1996, Ibrahim et al. 2016). Sauropod remains, preserved mainly as isolated teeth and bone fragments (Holwerda et al. 2018), probably indicate that these two sauropod groups were contemporaries in both formations.

Rebbachisauridae Bonaparte, 1997

Rebbachisaurus. *Rebbachisaurus garasbae* (Figs 104, 106) is a medium-sized sauropod, the holotype of which was excavated between 1949–1952 at Gara Sbaa in the Gara Sbaa Formation (Lavocat 1954a, 1954b, 1955, Wilson and Allain 2015). The bones were partially articulated and originally included 11 vertebrae from dorsal, sacral and anterior caudal regions and portions of both girdles. Some of these vertebrae, several ribs and a pelvic bone have been lost (Wilson and Allain 2015). Lavocat figured and described

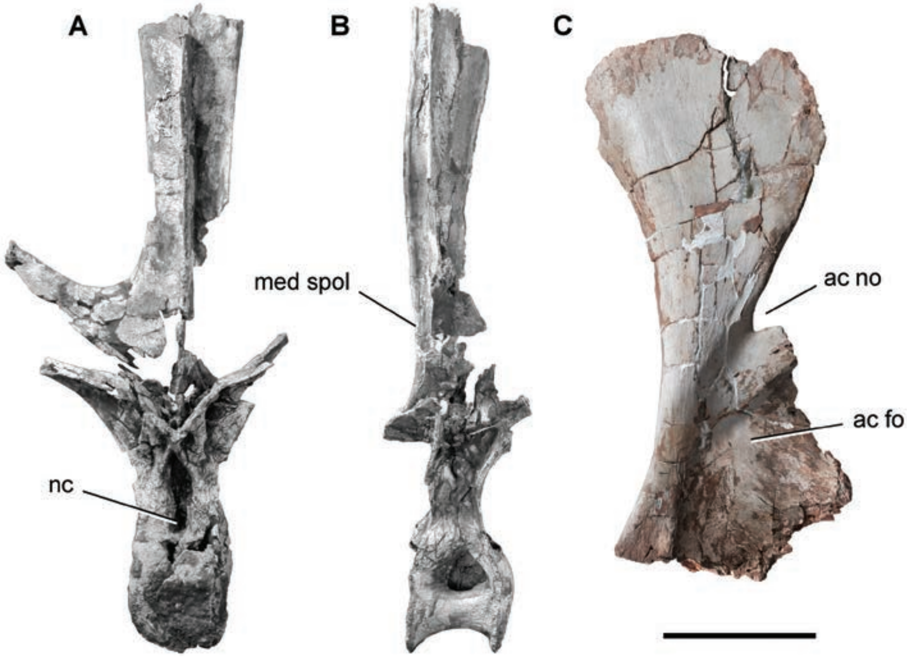

Figure 104. Holotype material of *Rebbachisaurus garasbae*. **A** Dorsal vertebra (MNHN-MRS 1957) in anterior view and (**B**) lateral view. **C** Scapula (MNHN-MRS 1957) in lateral view. Scale bar equals 30 cm. Abbreviations: **ac fo** acromial fossa **ac no** acromial notch **nc** neural canal **med spol** medial spinopostzygapophyseal lamina. Photographs of fossils of the sauropod *Rebbachisaurus garasbae* were taken by Christian Lemzaouda (from Wilson and Allain 2015).

only a few bones. The unusual morphology of the vertebrae and skull and the diplodocoid affinity of *Rebbachisaurus* was only revealed later on the basis of better preserved skulls and skeletons of *Limaysaurus* in Argentina (Calvo and Salgado 1995, Salgado et al. 2004) and *Nigersaurus* in Niger (Sereno et al. 1999, 2007, Sereno and Wilson 2005).

Thin laminae, large oval pleurocoels and highly pneumatized bone characterize the centra and neural arches of *R. garasbae* (Figs 104A, 106C, E) and other rebbachisaurids and the scapula (Fig. 104C) has a narrow U-shaped notch between the acromion and blade (Sereno et al. 2007). Recently an anterior caudal vertebra was described that is consistent with vertebral morphology of rebbachisaurids (Mannion and Barrett 2013).

Isolated narrow, subcylindrical teeth from the Kem Kem Group (Holwerda et al. 2018), first reported by Kellner and Mader (1997), may also pertain to *R. garasbae* (Fig. 107). This tooth form is rarer than the more swollen, pointed and more strongly textured crowns that are referred to Titanosauria (Fig. 108). The figured tooth was found in situ at Aferdou N'Chaft in the Douira Formation, whereas the holotype of *R. garasbae* was found in the underlying Gara Sbaa Formation. This tooth form, on the other hand, may pertain to a narrow-crowned titanosaurian sauropod, the remains of which have been found in situ in the Douira Formation.

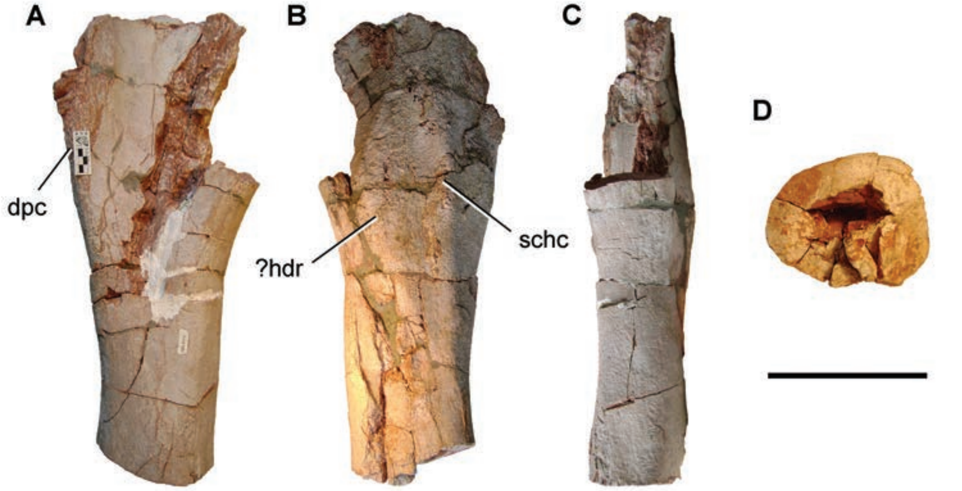

Figure 105. Left humerus of *Rebbachisaurus garasbae*. MNHN-MRS 2002 in (**A**) anterior, (**B**) posterior, (**C**) lateral and (**D**) cross-sectional view. Scale bar equals 20 cm. Abbreviations: **dpc** deltopectoral crest **hdr** ridge emanating from humeral head **schc** insertion for M. scapulo-humeralis cranialis.

The enamel on the potential rebbachisaurid crown is present on both sides and is lightly textured (Fig. 107), unlike crowns in the derived rebbachisaurid *Nigersaurus* (Sereno and Wilson 2005). There are no grooves on mesial and distal crown edges for adjacent crowns, and the crown tip is truncated by a single low-angle wear facet (Fig. 107B), rather than a pair of wear facets as in *Nigersaurus* (Sereno and Wilson 2005). If these slender subcylindrical crowns pertain to *R. garasbae*, the Kem Kem rebbachisaurid may not have had the derived self-supporting tooth batteries present in *Nigersaurus*.

Titanosauria Bonaparte & Coria, 1993

Isolated teeth and several postcranial bones pertain to one or more titanosaurian sauropods (Figs 108, 109), the teeth comprising the majority of those found pertaining to sauropods. The crowns are swollen to a greater degree and shorter than the potential rebbachisaurid crown (Fig. 107). Some preserve pointed, unworn tips (Fig. 108G, J), whereas others are truncated by a single high-angle wear facet (Fig. 108A, K, R-T, W).

Some crowns seem particularly narrow (Fig. 108G, I, P, W) or relatively broad with a posteriorly oriented tip (Fig. 108Q). Many narrow-crowned sauropods have larger slightly swollen upper crowns than lower crowns (Table 9), and distal displacement of the crown tip may only characterize the more distal crowns in the tooth rows. Identifying titanosaurian taxa on the basis of isolated teeth is not possible.

Isolated sauropod caudal vertebrae, a partial humerus and a tarsal bone have also been identified as titanosaurian (Russell 1996, Mannion and Barrett 2013, Ibrahim et al. 2016), and some of these are indicative of large body size comparable to the possibly coeval titanosaurian *Paralititan stromeri* from Egypt (Smith et al. 2001).

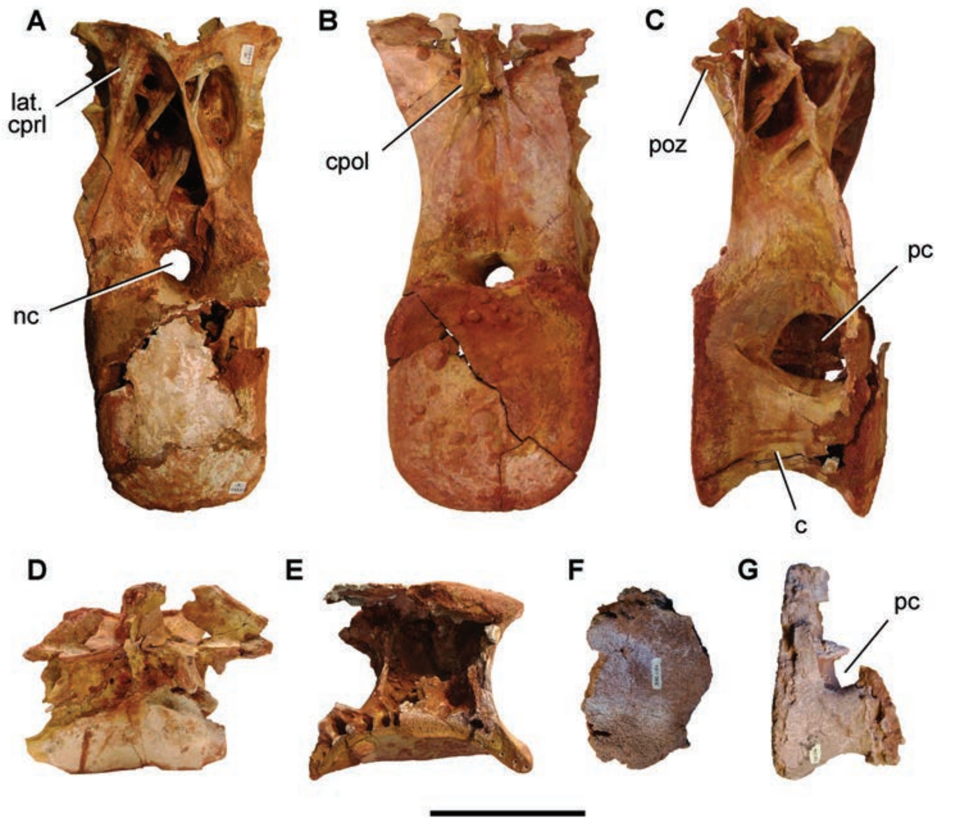

Figure 106. Axial remains of *Rebbachisaurus garasbae*. NMC 50844 in (**A**) anterior, (**B**) posterior, (**C**) right lateral and (**D**) dorsal views **E** Internal appearance of centrum **F** Posterior articular section of vertebra (MNHN-MRS 1184) **G** Partial centrum (MNHN-MRS 1857) in right lateral view. Scale bar equals 20 cm. Abbreviations: **c** centrum **cpol** centropostzygapophyseal lamina **lat. cprl** lateral centroprezygapophyseal lamina **nc** neural canal **pc** pleurocoel **poz** postzygapophysis.

Table 9. Dimensions of select sauropod crowns from the Kem Kem Group. Measurements in mm. Abbreviation: CMN Canadian Museum of Nature, Ottawa, Canada.

Specimen	Height	Basal width
CMN 50441A	50	14
CMN 50441B	51	11
CMN 50441C	44	11
CMN 50441D	42	13
CMN 50441E	42	11
CMN 50441F	36	11
CMN 50441G	28	10
CMN 50825	57	11
CMN 41808	37	10
CMN 41810	49	12
CMN 41812	49	12
CMN 50822	52	14

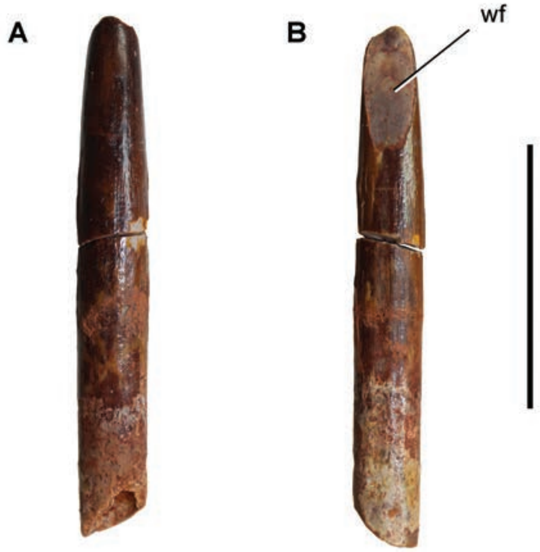

Figure 107. Sauropod tooth collected at Aferdou N'Chaft, tentatively referred to Rebbachisauridae. FSAC-KK 910 in (**A**) labial and (**B**) lingual view. Scale bar equals 3 cm. Abbreviation: **wf** wear facet.

The proximal end of an ulna from a very large sauropod was found in situ in the Douira Formation (Fig. 109, Table 10, UCRC PV9). In lateral or medial views, the rounded olecranon is as prominent proximally as in *Camarasaurus* (Fig. 109A, B). The shaft is very broadly arched to accommodate the head of the radius, more so than in *Camarasaurus*, and the lateral process extends only a short distance from the olecranon. As a result, in proximal view the ulna is T-shaped rather than Y-shaped, and the radial fossa is exceptionally broad (Fig. 109C). The anteromedial (coronoid) process, in contrast, is very prominent, the shaft under the process concave (Fig. 109A). In all of these regards, the ulna closely resembles that of the titanosaur *Isisaurus* (Wilson and Upchurch 2003: fig. 7), and we tentatively suggest that there was a very large-bodied titanosaurian sauropod in the Kem Kem fauna (Ibrahim et al. 2016). The size of this proximal ulna is remarkable. Its broadest proximal width measures 51 cm (Fig. 109C). This measure, approximately one-half meter across the olecranon and coronoid process, is more than twice as large as in other sauropods such as *Camarasaurus* (Table 10) and suggests that

Table 10. Maximum transverse width of the proximal end of large sauropod ulnae in comparison to the large ulna from the Gara Sbaa Formation (UCRC PV362). Measurements in mm. Abbreviation: UCRC University of Chicago Research Collection, Chicago, USA (measurements based on the references provided below).

Taxon/specimen	Maximum width
Neosauropoda indet. UCRC PV362	440
Camarasaurus grandis (Ostrom and McIntosh 1966)	210
Diamantinasaurus matildae (Hocknull et al. 2009)	338
Titanosaurus colberti (Jain and Bandyopadhyay 1997)	221

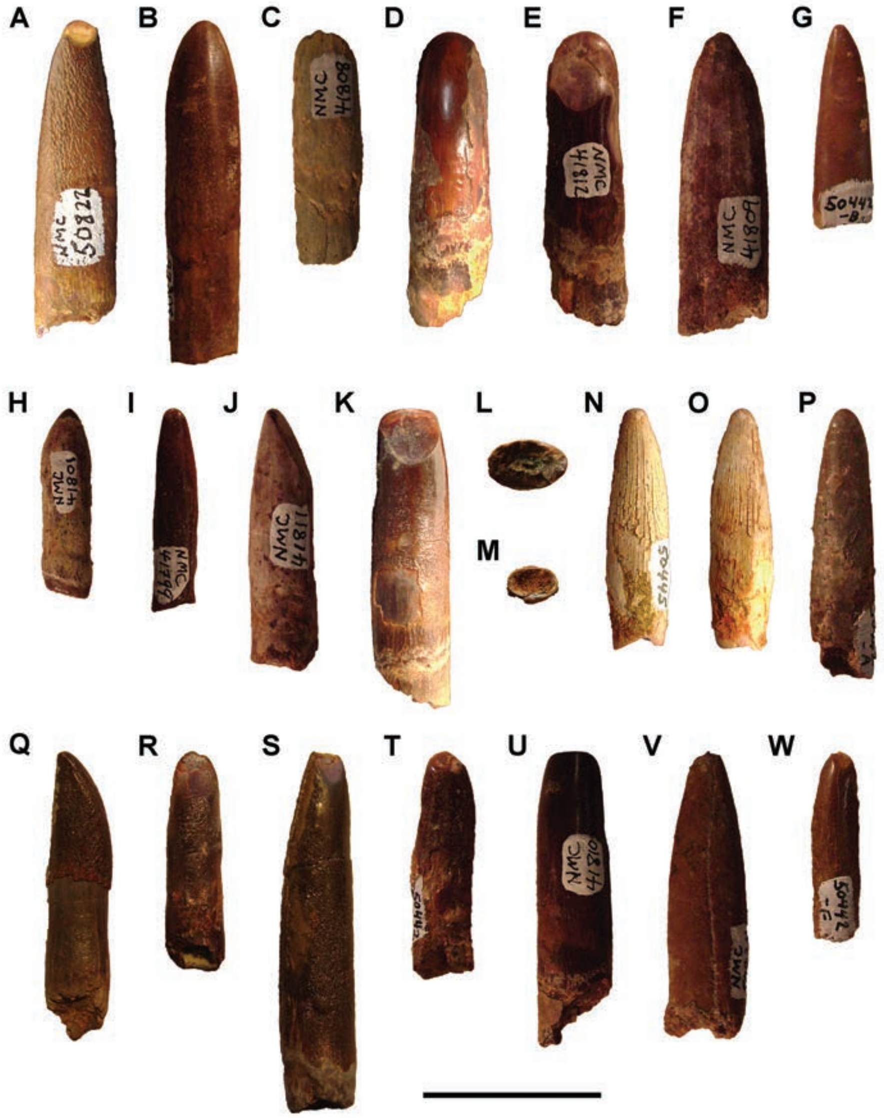

Figure 108. Isolated sauropod teeth from the Kem Kem Group. **A** NMC 50822 in lingual view **B** NMC 50825 in labial view **C** NMC 41808 in labial view. NMC 41812 in (**D**) labial and (**E**) lingual views **F** NMC 41809 in labial view **G** NMC 50442B in labial view **H** NMC 41801 in labial view **I** NMC 41799 in lingual view **J** NMC 41811 in lateral view. NMC 41810 in (**K**) lingual and (**L**) cross-sectional view. NMC 50445 in (**M**) cross-sectional, (**N**) labial and (**O**) lingual views **P** NMC 50442A in labial view **Q-S** BSPG 1993 IX 331 in (**Q**) lateral and (**R**, **S**) lingual views respectively **T** NMC 50442D in lingual view **U** NMC 41810 in labial view **V** NMC 50824 in labial view **W** NMC 50442 in labial view. Scale bar equals 3 cm.

the distal end of its associated humerus would also have measured as wide. A spheroidal depression in lateral view appears to be a postmortem artifact (Fig. 109B).

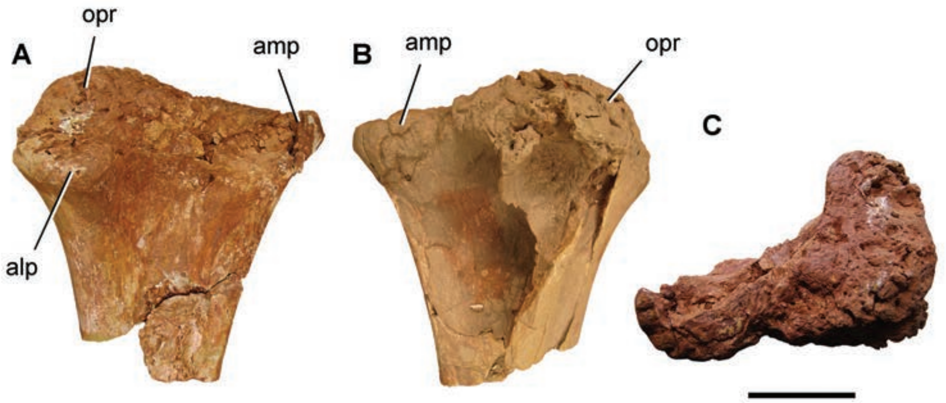

Figure 109. Proximal section of a right ulna of a giant sauropod. UCRC PV9 in (**A**) anterior, (**B**) posterior and (**C**) distal view. Scale bar equals 20 cm. Abbreviations: **alp** anterolateral process **amp** anteromedial process **opr** olecranon process.

Theropoda Marsh, 1881

Theropods dominate among dinosaur fossils recovered in the Kem Kem Group. Most specimens are isolated teeth found in surface debris, but some are partial jaw pieces, vertebrae, unguals and, very rarely, articulated partial skulls and skeletons. The best-known Kem Kem theropods are large-bodied basal neotheropods including abelisaurids, spinosaurids, and carcharodontosaurids. Controversy has surrounded the generic and specific assignment of these Kem Kem theropods. Are they congeneric or conspecific with similar age taxa named a century ago (e.g., Stromer 1915, 1934) from localities in Egypt at similar latitude but some 4,000 km distant? Those fossils, to render the question more challenging, were destroyed in World War II, and comparable Egyptian exemplars have never been found since. We review our field discoveries from the Kem Kem Group and all previous discussions on these large-bodied theropods. We also review the scant tooth and bone records for more advanced paravian theropods.

Theropod teeth are relatively common on outcrop surfaces at many localities in the Kem Kem Group. A few were discovered *in situ* near the boundary between the formations at Aferdou N'Chaft (Fig. 110E, K, L) and Boumerade (Fig. 110F, G). The familial identity of some theropod teeth, especially those of intermediate size (~ 1–4 cm), remains uncertain (Fig. 110F, G). These crowns are laterally compressed with serrated mesial and distal carinae. The serrations are equal in size mesially and distally, and the distal carina is nearly straight rather than markedly recurved.

A portion of the isolated teeth can be placed in one of three tooth morphotypes. *Tooth morphotype 1* includes relatively small teeth that may pertain to abelisauroid theropods (Fig. 110E, L). The crowns are strongly laterally compressed, slightly curved lingually, and narrower in labial or lingual views than carcharodontosaurid teeth. The posterior distal carina is straight in lateral view, the serrations are equal-sized fore and aft, and there are no marginal wrinkles or grooves. These teeth are nearly identical to those in an abelisaurid maxilla from the Kem Kem Group (Mahler 2005), which are very similar to those of the Nigerien abelisaurid *Rugops* (Sereno et al. 2004).

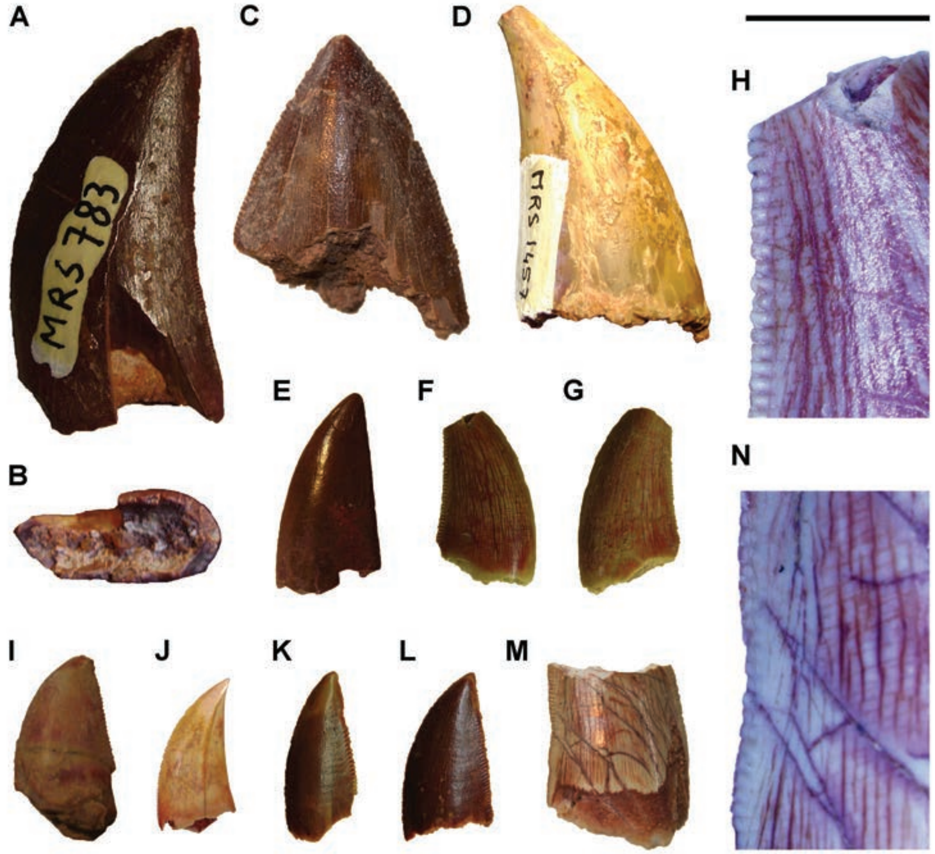

Figure 110. Indeterminate theropod teeth from the Kem Kem Group. MNHN-MRS 783 in (**A**) lateral and (**B**) cross-sectional views **C** FSAC-KK 914 in lateral view **D** MNHN-MRS 1457 in lateral view **E** FSAC-KK 913 in lateral view. FSAC-KK 909 in (**F**) labial and (**G**) lingual views **H** Detailed view of FSAC-KK 909. **I** FSAC-KK unnumbered in lateral view **J** NMC 50446-C **K** FSAC-KK 915 in lateral view **L** FSAC-KK 912 in lateral view **M** FSAC-KK 916 in labial view **N** Detailed view of denticles of FSAC-KK 916. Scale bar equals 2 cm in **A-C**, **E-G** and **I-M**, 3 cm in **D** and 5 mm in **H** and **N**.

Tooth morphotype 2 includes the largest theropod teeth from the Kem Kem Group and may pertain to carcharodontosaurid theropods (Fig. 110A-C; Sereno et al. 1996). As Stromer (1931) noted, the distal one-half of the distal carina is gently convex rather than straight or concave (Fig. 110A, C). The serrations are equal in size on mesial and distal carina and are often flanked by enamel wrinkles. Some of these wrinkles continue as subtle raised growth lines across the crown to the opposing carina. The carcharodontosaurids *Carcharodontosaurus* (Sereno et al. 1996) and *Mapusaurus* (Coria and Currie 2006) exhibit these features, which vary in prominence along the tooth rows and in emerging erupting crowns. Although marginal enamel wrinkles are prominently expressed in several carcharodontosaurids, they also sporadically occur in other theropods including *Tyrannosaurus rex*, as they appear to form during growth of the crown in the alveolar crypt (Brusatte et al. 2007).

Tooth morphotype 3 includes relatively small teeth that may pertain to dromaeosaurid theropods. They are laterally compressed with a distal carina that is either concave, straight or gently convex. They are characterized by having distinctly larger serrations on the distal than mesial carina (Fig. 110F, G, K). Some have short "blood grooves" between the serrations (Fig. 110). In these regards, they resemble teeth recovered and described by Amiot et al. (2004) from the Kem Kem Group as well as considerably older teeth from earliest Cretaceous sediments to the north near Anoual, Morocco (Knoll and Ruiz-Omeñaca 2009).

Pointed manual and pedal unguals pertaining to theropods have also been recovered from the Kem Kem Group (Russell, 1996). One ungual was surface collected from Gara Sbaa (Fig. 111F-H) and presumably came from the Gara Sbaa Formation. Another ungual came from Aferdou N'Chaft at the base of the Douira Formation (Fig. 111W), and many others were commercially collected from unknown localities (Fig. 111A-E, I-V, X, Y).

Many unguals are approximately 2–4 cm in length and cannot be assigned to a particular theropod subgroup. Some are regarded as manual unguals, because they are laterally compressed, recurved along their length, and have a rounded rather than flattened ventral margin (Fig. 111I-M, P). Other unguals are regarded as pertaining to the pes, because they are less recurved and have a flattened ventral margin (Fig. 111F-H, R, W, Y). The pedal unguals do not look like those associated with *Deltadromeus*, which have a low, raised V-shaped platform along the margins of the ventral side and a notched end to the ungual attachment groove. Nor are they like the characteristic flat-bottomed pedal unguals of *Spinosaurus* (Stromer 1915, Ibrahim et al. 2014b) or possible spinosaurid unguals reported from India (Novas and Bandyopadhyay 2001, Novas et al. 2005a: fig. 2). The diversity in ungual form figured here suggests there exists a hidden diversity of non-avian theropods in the Kem Kem Group.

Manual ungual morphotype 1 are large manual unguals exceeding 5 cm in length and may pertain to spinosauroid theropods (Fig. 111A, B). Russell (1996: fig. 24) was first to figure this characteristic manual ungual. In lateral view, the ungual is gently recurved and has a dorsoventrally shallow base. The flexor tubercle is distally offset, and an attachment groove for the ungual sheath is positioned near the central axis of the ungual in lateral view. The dorsal edge of the base of the ungual is elevated as an extensor tuberosity, a feature otherwise known among maniraptoran theropods. Other manual unguals of similar form are more recurved with a more ventrally offset attachment groove for the ungual sheath (Fig. 111C-E). These may pertain to lateral manual digits of spinosaurids, a theropod similar to *Megaraptor* (Porfiri et al. 2007), or to another unknown theropod subgroup.

Manual ungual morphotype 2 are large robust unguals approximately 3–5 cm in length that may pertain to carcharodontosaurid theropods (Fig. 111N, O). They are strongly recurved with a dorsoventrally deep base and flexor tubercle, tapering rapidly to their tip. The attachment groove for the ungual sheath is positioned near, or dorsal to, the central axis of the ungual in lateral view. Manual unguals, nevertheless, are nearly unknown among carcharodontosaurid theropods (Coria and Currie 2006: fig. 25). Robust, curved manual unguals of this general form, in addition, occur in many

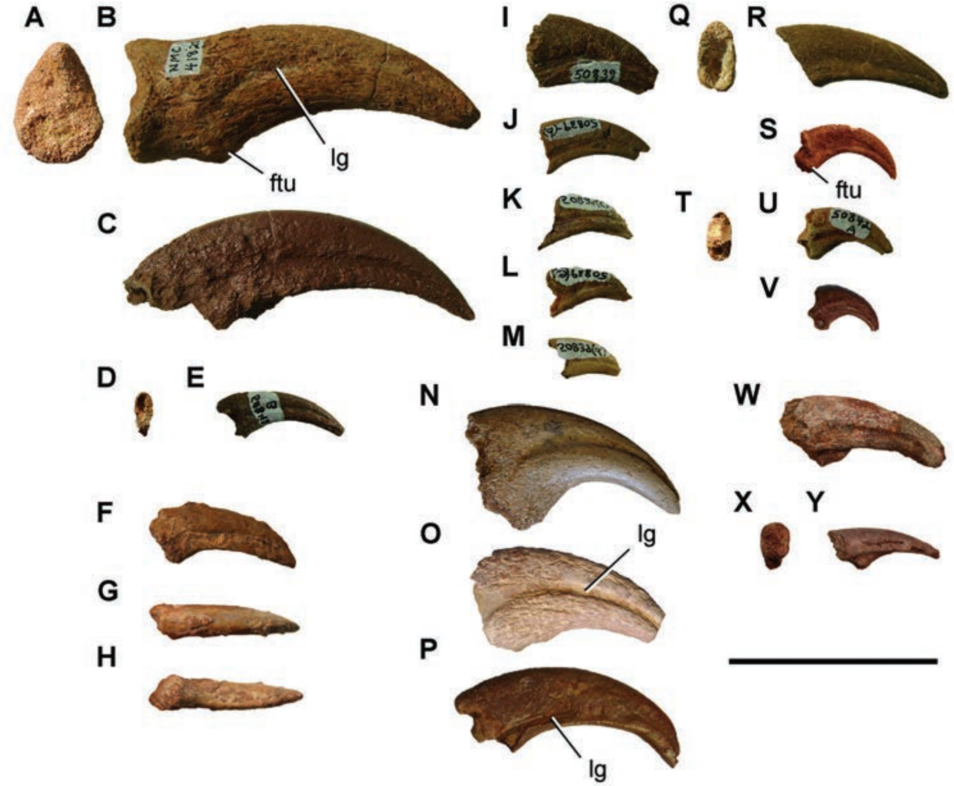

Figure 111. Isolated unguals from the Kem Kem Group. NMC 41820 in (**A**) posterior and (**B**) lateral views **C** NMC 50987 (cast) in lateral view. NMC 50842b in (**D**) posterior and (**E**) lateral view. FSAC-KK 918 in (**F**) lateral, (**G**) dorsal and (**H**) ventral views **I** NMC 50839a in lateral view **J** NMC 50839b in lateral view **K** 50839c (reversed) in lateral view **L** NMC 50839e in lateral view **M** NMC 50839d (reversed) in lateral view **N** MPDM 43 in lateral view **O** BSPG 1993 IX 311 in lateral view **P** BSPG 1993 IX 330 in lateral view. NMC 50826 in (**Q**) posterior and (**R**) lateral views **S** NMC 50386 in lateral view. NMC 50842A in (**T**) posterior and (**U**) lateral views **V** BSPG 1993 IX 330 in lateral view **W** FSAC-KK 202 in lateral view. FSAC-KK 917 in (**X**) posterior and (**Y**) lateral view. Scale bar equals 5 cm. Abbreviations: **ftu** flexor tubercle **lg** lateral groove.

other basal tetanuran theropods such as *Allosaurus* (Madsen 1976), *Sinraptor* (Currie and Zhao 1993) and *Afrovenator* (Sereno et al. 1994), and their recurvature varies across the inner three manual digits.

Manual ungual morphotype 3 are moderate-sized unguals approximately 2–3 cm in length that may pertain to paravian or dromaeosaurid theropods (Fig. 111S-V). They are strongly recurved with a well-developed flexor tubercle (Ostrom 1969). In some of these unguals, the flexor tubercle is pendant (Fig. 111V). Manual unguals similar in general form, nonetheless, are present in more basal coelurosaurs such as compsognathids (Dal Sasso and Maganuco 2011), and so assignment to Paraves or another theropod subgroup is not possible.

Abelisauroidea Bonaparte & Novas, 1985. Russell (1996) described two edentulous dentary fragments with squared alveoli as evidence of abelisaurids in the Kem Kem Group, referring the smaller one to the genus *Majungasaurus*. This smaller, abraded dentary piece (Fig. 112G, H) may well pertain to an abelisaurid on the basis of the slender proportions of the dentary ramus, its dorsal curvature in lateral view, the relatively small, squared alveoli of uniform size, and the shallow depth of the tooth crypts (Table 11). There is no justification, however, for reference to the Malagasy genus *Majungasaurus*, which Russell (1996) based solely on the preserved number of alveoli. The larger specimen is better identified as pertaining to a carcharodontosaurid, as discussed below (see also Ibrahim et al. 2017).

A commercially collected maxilla with several crowns in place (Fig. 112A-D) provided the first well preserved evidence of abelisaurid theropods in the Kem Kem Group (Mahler 2005). The maxilla and teeth closely resemble the Nigerien abelisaurid *Rugops primus* in size and form (Figs 112, 113C-E, 114, Sereno et al. 2004). It also closely resembles maxillae of other abelisaurids, in particular a maxilla of similar age from Argentina (Lamanna et al. 2002). The maxilla has a nearly identical pattern of external sculpting, grooves and foramina, the anterior margin of the maxilla has a nearly vertical orientation, the alveoli are subrectangular, and the teeth are relatively slender with a nearly straight distal carina. Because the diagnostic features of *Rugops primus* are located elsewhere on the skull, we cannot refer the maxilla to that genus or species. We support Mahler's interpretation of this maxilla (UCRC PV10) as an indeterminate abelisaurid.

Additional commercially collected specimens have been described more recently, including a pair of maxillae with teeth close in form to UCRC PV10 (D'Orazi Porchetti et al. 2011), cervical vertebrae (Smyth et al. 2020), a more questionable partial ilium (Zitouni et al. 2019), and the proximal end of a large femur (Chiarenza and Cau 2016). There is evidence, thus, for at least one mid- to large-sized Kem Kem abelisaurid, a subgroup known to survive in northern locales on Africa until the end of the Cretaceous (Smith and Lamanna 2006, Longrich et al. 2017). In addition, a single anterior cervical vertebra has been recovered and allied with Noasauridae (Smyth et al. 2020), a group of small-bodied abelisauroids.

Deltadromeus. The partial and only known postcranial skeleton of *Deltadromeus agilis* (Sereno et al. 1996, UCRC PV11) was discovered weathering from a coarse-grained sandstone in the upper third of the Gara Sbaa Formation (Fig. 115). Many

Table 11. Alveolar length in the middle of the maxilla in three abelisaurid theropods showing maximum length centered on alveoli 6–8. Measurements in mm. Abbreviations: FMNH Field Museum of Natural History, Chicago, USA; UCRC University of Chicago Research Collection, Chicago, USA; MNBH Musée national Boubou Hama, Niamey, République du Niger.

Specimen	Alveolus number					
	4	5	6	7	8	9
Majungasaurus crenatissimus (FMNH PR 2100)	19.1	19.6	17.0	21.0	20.6	19.6
Abelisauridae indet. (UCRC PV10)	15.1	16.4	17.3	18.0	18.0	17.3
Rugops primus (MNBH IGU1)	13.5	15.6	15.9	15.3	14.5	14.2

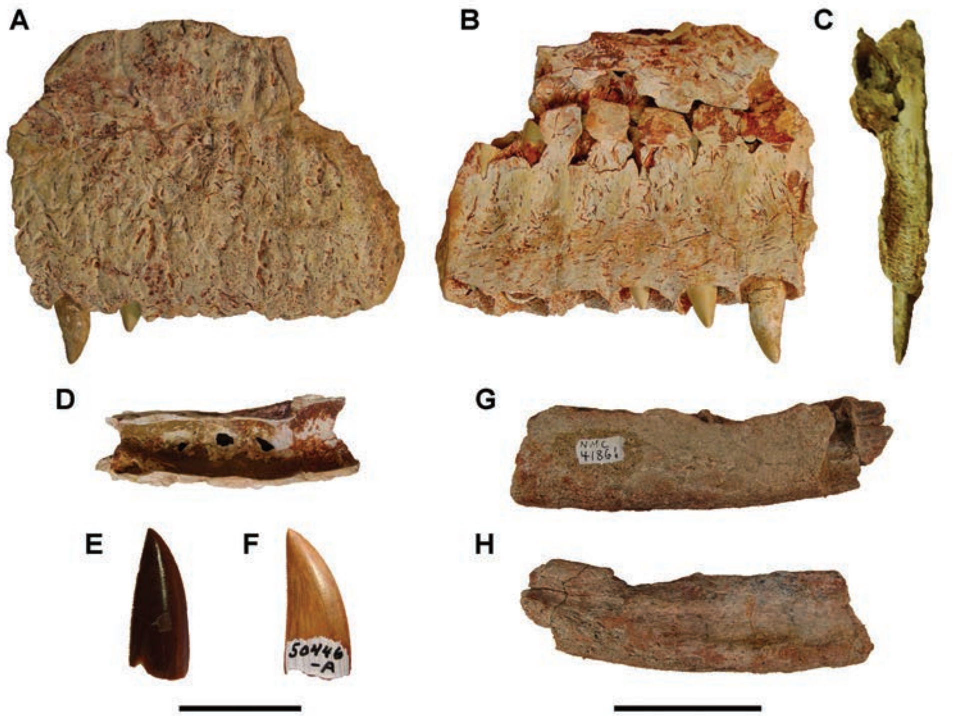

Figure 112. Abelisaurid jaw elements and teeth from the Kem Kem Group. Right maxilla (UCPC PV10) in (**A**) lateral, (**B**) medial, (**C**) anterior and (**D**) dorsal view. ?Abelisaurid tooth (MSNM V6053) in (**E**) labial view. ?Abelisaurid tooth (NMC 50446) in (**F**) labial view. Right dentary section (NMC 41861) in (**G**) lateral and (**H**) medial view. Scale bars equal 5 cm in **A-D**, **G** and **H**, 2 cm in **E** and **F**.

portions of the skeleton are preserved in articulation (Figs 116, 117). Rostral teeth of the sawfish *Onchopristis* and crocodyliform teeth were found near the skeleton.

The form of the coracoid, femur, and the proximal ends of the tibia and fibula of *D. agilis*, in particular, match that of isolated material recovered from the Bahariya oasis in the Western Desert in Egypt and referred by Stromer (1934) to his theropod taxon *Bahariasaurus ingens*. Although Sereno et al. (1996) noted several derived features shared by these overlapping bones, they erected a new taxon *Deltadromeus agilis* for the Moroccan partial skeleton, because Stromer's bones were not part of his holotypic specimen for *B. ingens*, they probably pertain to multiple individuals, and they have all been destroyed.

Stromer (1934: 24, pl. II, figs 4, 9, 10) was explicit in designating a specimen he numbered 1922 X47 as the holotype of *B. ingens*. Composed of some vertebral parts, a rib fragment, a proximal ischium, and conjoined pubes, the fragmentary holotype of *B. ingens* differs from the Moroccan skeleton most noticeably in the shape of the iliac peduncle of the ischium and in several details of the pubes, as reported by Sereno et al. (1996: 991, note 32). Thus, the Moroccan skeleton cannot be referred to *B. ingens*, which we regard as a *nomen dubium*.

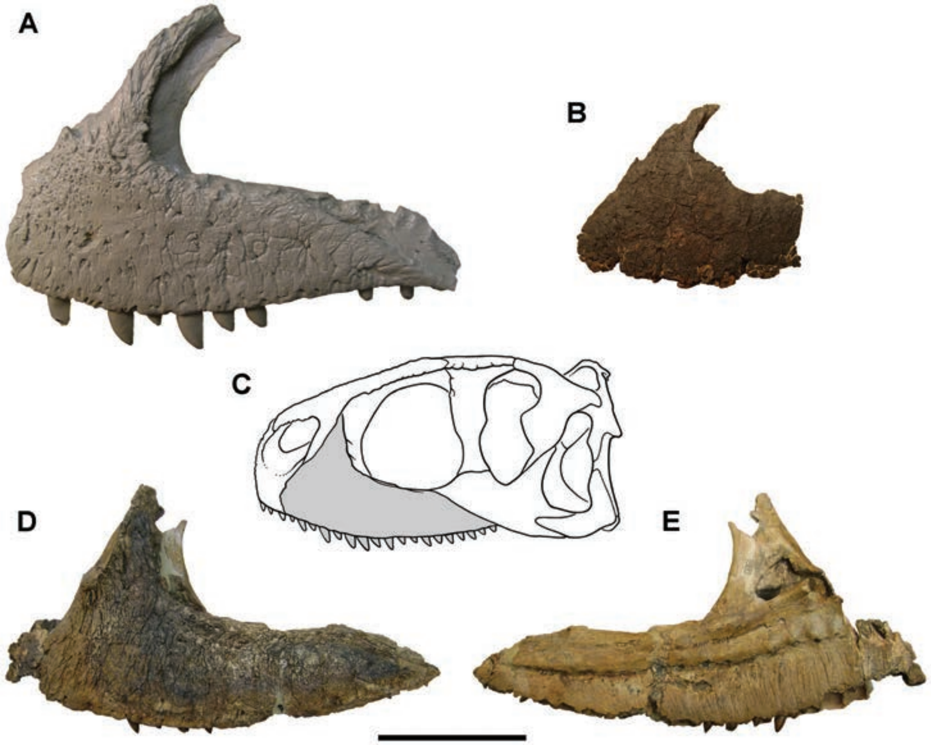

Figure 113. Abelisaurid maxillae from Madagascar and Niger. **A** Left maxilla of *Majungasaurus crenatissimus* (FMNH PR 2100, cast) in lateral view **B** Left maxilla of *Kryptops palaios* (MNN GAD1) **C** Reconstructed skull of *Rugops primus* (modified from Sereno et al. 2004) with maxilla in gray. **D**, **E** Left maxilla of *Rugops primus* (MNN IGU1) in lateral and medial view. Scale bar equals 10 cm in **A**, **B**, **D** and **E**.

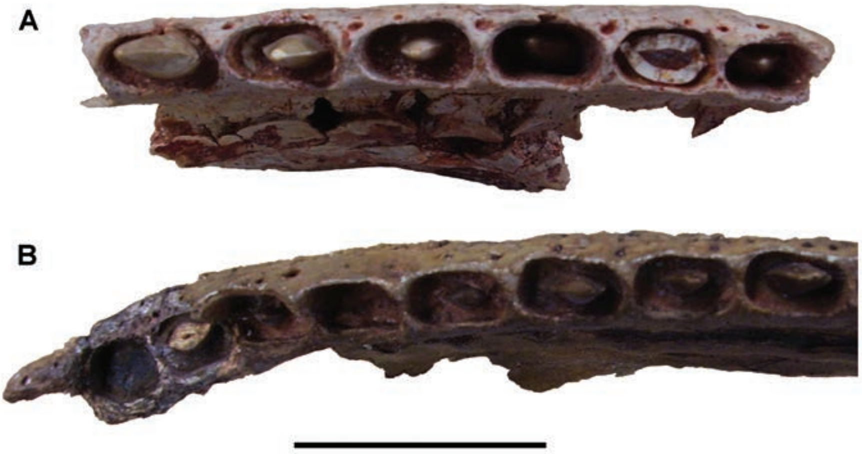

Figure 114. Comparison of abelisaurid maxillae from Morocco and Niger. **A** Left maxilla (UCPC 10, mirrored) in ventral view **B** Left maxilla of *Rugops primus* (MNN IGU1) in ventral view. Scale bar equals 5 cm.

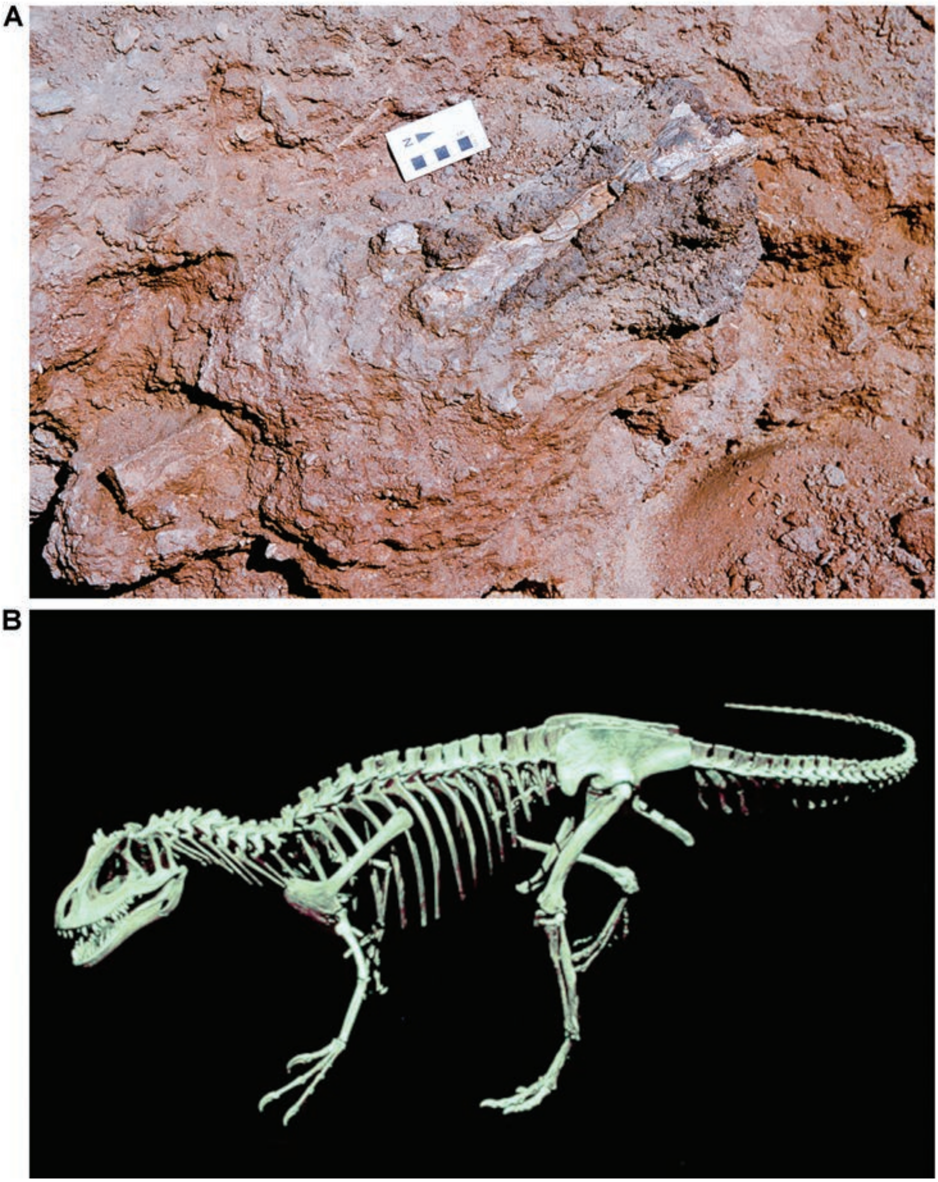

Figure 115. Excavation and skeletal reconstruction of *Deltadromeus agilis*. **A** Excavation of the fibula **B** Mounted skeletal reconstruction using casts of the holotype UCRC PV11. Scale bar equals 5 cm in **A**. Length of skeleton in **B** is about 8 m.

Four of Stromer's referred bones, however, bear a striking resemblance to comparable bones in the skeleton of *D. agilis*. The coracoid is expanded anteroposteriorly with a subrectangular posterior process; the femur has a very similar proximal end (narrow head, prominent leaf-shaped anterior trochanter), narrow shaft proportions, the unusual accessory trochanter projecting posteriorly from the posterolateral edge of

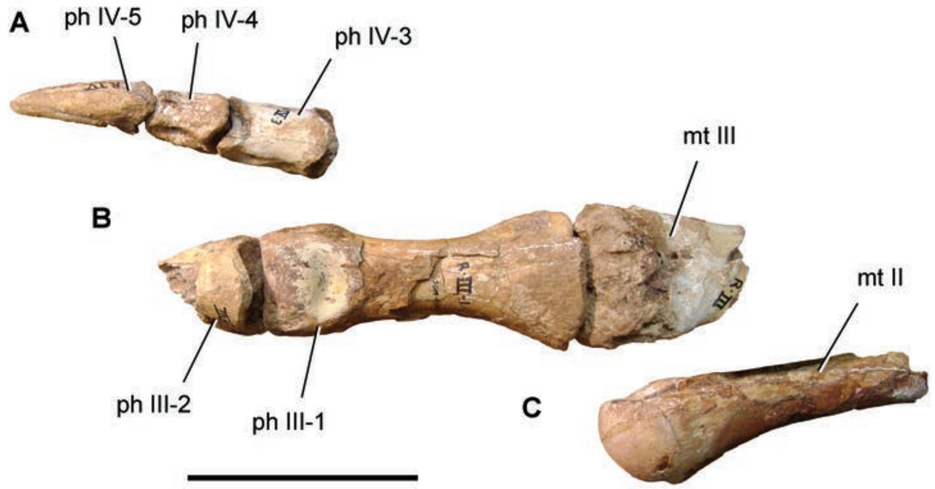

Figure 116. Well-preserved elements of the right pes of *D. agilis*. **A** Digit IV in dorsal view. **B** Digit III in dorsal view. **C** Digit II in dorsal view. Scale bar equals 10 cm. Abbreviations: **mt II**, **III** metatarsals II, III **ph III-1**, **2** pedal phalanges 1 and 2 of digit III **ph IV-3**, **4**, **5** pedal phalanges 3-5 of digit IV.

the shaft below the fourth trochanter, and a projecting anterior extension of the medial distal condyle (Stromer 1934: pl. III, fig. 5b); the proximal end of the tibia is very broad transversely across the condyles; and the proximal fibula has a very large, oval fibular fossa, a prominent anterior trochanter, distal to which it narrows rapidly to a slender shaft. We regard all of these bones as potentially indistinguishable from those of *D. agilis* and, for that reason, referred them to that genus and species (Sereno et al. 1996). In doing so, *D. agilis* became the third large theropod species shared between the Kem Kem Group and the Bahariya Formation (after *Spinosaurus aegyptiacus* and *Carcharodontosaurus saharicus*).

Recently, Ijouiher (2016) stated that Sereno et al. (1996) regarded *Deltadromeus agilis* as a senior synonym of *Bahariasaurus ingens*, that Stromer (1934: pl. II, fig. 2) regarded a pubis and other material he referred to *B. ingens* as a "paratype", that none of Stromer's referred material resembles *D. agilis* in any particular way, and that the referred femur does not have an autapomorphic accessory trochanter like that of *D. agilis*. All of the above statements are incorrect. Ijouiher (2016) confused the autapomorphic accessory trochanter of the femur in *D. agilis*, which we have not observed in any other theropod, with a ridge that is present in many theropods along the lateral edge of the distal extensor depression. The accessory trochanter projects posteriorly, not laterally or anteriorly, from the shaft, and is positioned approximately the same distance above the condyles as the fourth trochanter is positioned below the head of the femur (Stromer 1934: pl. II, fig. 2). The prominent anterior extension of the medial distal condyle is also visible in this figure of the Egyptian femur, another feature we have not observed in any other theropod besides *D. agilis*.

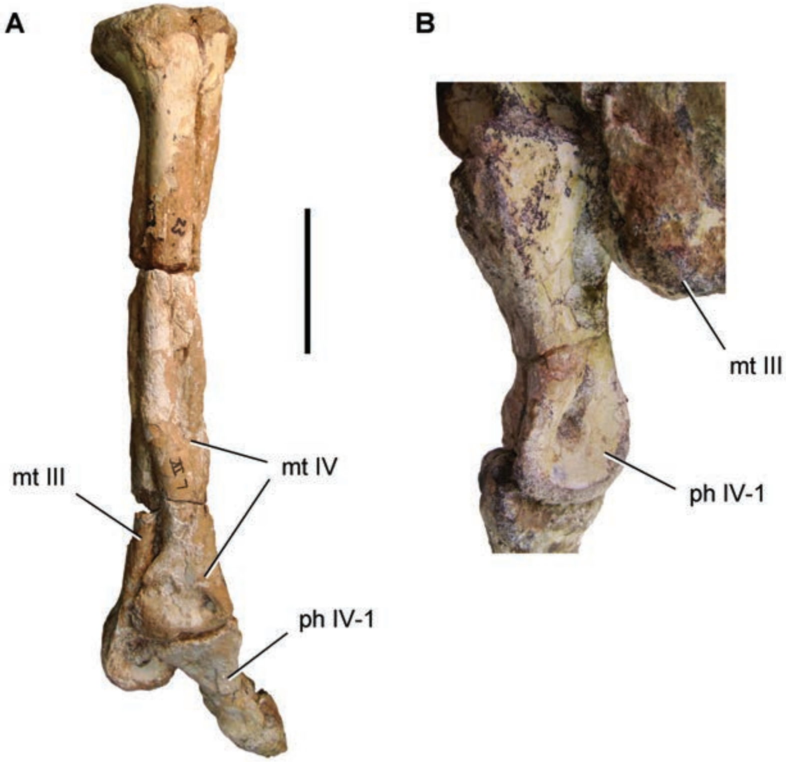

Figure 117. Left metatarsals of *Deltadromeus agilis* (UCRC PV11). **A** Lateral view **B** Detailed view of the first phalanx of digit IV. Scale bar equals 10 cm in **A**. Abbreviations: **mt III**, **IV** metatarsal III, IV **ph IV-1** phalanx 1 of digit IV.

The Egyptian femur is 122 cm long (Stromer 1934: 35) versus 74 cm for that of the holotype of *D. agilis* (Sereno et al. 1996), the axial column of which shows several indications of immaturity. Based on the linear dimensions of the Egyptian femur, that individual would be approximately 165% the size of the Moroccan individual (Fig. 118B) and only ~10% less than the largest individuals of *Tyrannosaurus rex* (Brochu 2003).

All of the material of *Deltadromeus agilis* is now fully prepared. Previously unprepared fragments include shaft pieces that have completed the humerus and fibula. These have been included in a revised skeletal restoration (Fig. 118A) showing the slender limb proportions of *D. agilis*. The skeleton is characterized by an enlarged acromion and coracoid, strap-shaped scapular blade, very elongate and slender humerus, pubis with slender sharply angled pubic foot, femur with unusual trochanters, tibia with a proximally prominent cnemial crest and fibula with a large fibular fossa.

Deltadromeus agilis most closely resembles the recently described "didactyl" theropod *Gualicho shinyae* from the similar-age Huincul Formation of southern Argentina (Apesteguía et al. 2016b). The similarity of the derived pectoral girdle, slender forelimbs, form of the pubis, and form of the major hind limb bones suggests a close relationship.

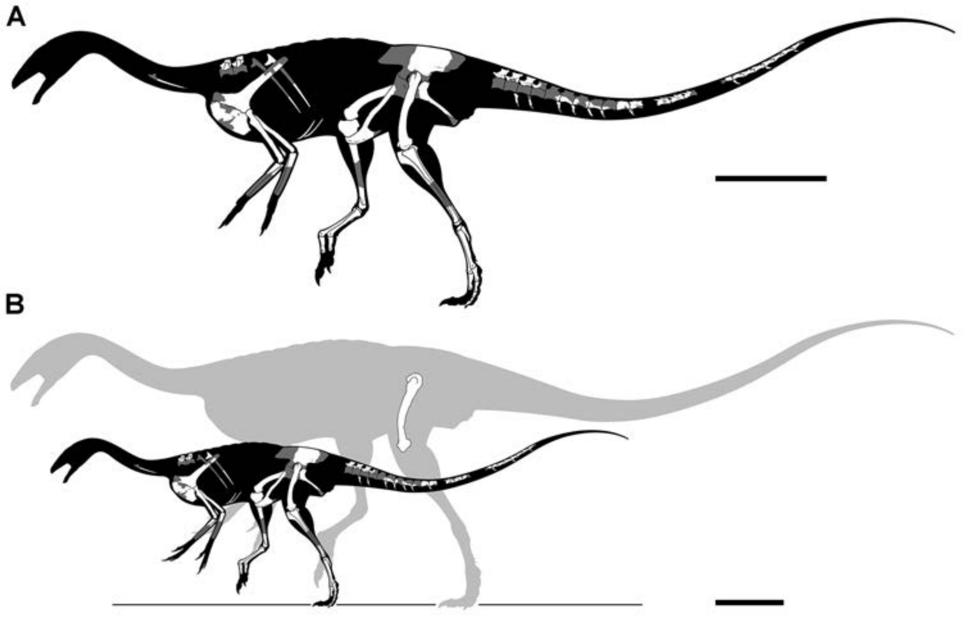

Figure 118. *Deltadromeus agilis* from Morocco and Egypt. **A** Revised reconstruction based on UCRC PV11 **B** Holotype compared to a large femur (now lost) referred to the genus and species from the Bahariya Formation, Egypt. Known elements in white. Scale bars: 1 m.

Unfortunately, neither specimen preserves any part of the skull. Unlike *D. agilis* the humerus in *G. shinyae* is not as elongate, although both have slender humeri characterized by a relatively narrow proximal end and reduced deltopectoral crest. Although described as didactyl, enough of metacarpal 3 is preserved in *G. shinyae* to suggest that a slender third digit was present and functional (*contra* Apesteguía et al. 2016b).

Whereas the new Argentine material adds tantalizing details to what is known collectively from these specimens, the phylogenetic position of these genera to each other and to other theropods remains ambiguous (Apesteguía et al. 2016b). Originally *D. agilis* was thought to be a basal coelurosaurian, based largely on the derived morphology of the distal tibia (anteroposteriorly flattened), large medial fossa on the proximal fibula, and the tall astragalar ascending process (Sereno et al. 1996). As more information on basal theropods emerged, *Deltadromeus* was tentatively repositioned as a noasaurid (Sereno et al. 2004) or abelisauroid (Carrano and Sampson 2008), based on several features such as the form of the pectoral girdle (expanded coracoid, strap-shaped scapular blade) and slender distal condyles of metatarsal 4 (Sereno et al. 1996: fig. 3K).

Spinosauridae Stromer, 1915. Spinosaurid fossils from the Kem Kem Group are most commonly found as isolated specimens, which has generated controversy over how many taxa are present. We review that material below, including the more complete specimens recovered recently that in our view suggest there is but one species present, *Spinosaurus aegyptiacus.*

Isolated specimens. Subconical teeth, jaw fragments with empty subcircular alveoli, and fragments of elongated neural spines are the most common spinosaurid

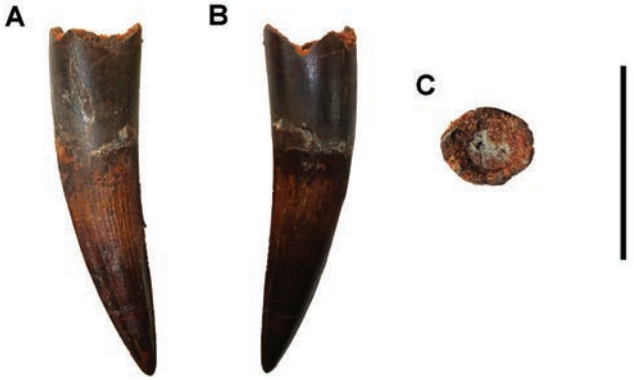

Figure 119. Tooth of *Spinosaurus aegyptiacus* (FSAC-KK 08) from the Kem Kem Group in (**A**) side, (**B**) opposing side and (**C**) basal view. Scale bar equals 5 cm.

specimens preserved in the Kem Kem Group (Figs 119–122). More rarely, partial or complete isolated cranial bones (Figs 123–125), vertebrae (Figs 126, 127), and limb bones are preserved. Cranial bones recovered during the 1995 expedition include fused nasals with a portion of the nasal crest (Dal Sasso et al. 2005: fig. 3), several frontals, and a complete paroccipital process (UCRC PV14–17). Recently described commercially collected cranial bones include a frontoparietal (Arden et al. 2019: fig. 2).

Isolated spinosaurid vertebrae have been described from cervicodorsal and caudal regions (Russell 1996, Sereno et al. 1996). Many of the cervicodorsal vertebrae have short, deeply keeled centra with very broad, kidney-shaped articular faces (Fig. 128), and the caudal vertebrae have neural spines that are anteroposteriorly, rather than transversely, compressed (Russell 1996: figs 10, 12). Limb bones at least tentatively referable to spinosaurids include a humerus, elongate penultimate manual phalanges, and elongate gently curved manual unguals (Russell 1996: figs 22–24, Ibrahim et al. 2014b).

More complete specimens. Very rarely more complete specimens have come to light representing partial crania or postcranial skeletons. Only one of each has been recovered so far from the Kem Kem Group. In 1975 a partial rostrum of an adult individual (MSNM V4047, Figs 123A, 124A, 129B) preserving the premaxillae, maxillae and anterior portions of the nasals was collected commercially (Dal Sasso et al. 2005). Although the locality and horizon within the Kem Kem Group for this specimen remains unknown, it is thought to have come from an outcrop east or southwest of Taouz (Dal Sasso et al. 2005).

In 2008 a partial postcranial skeleton (FSAC-KK 11888, Fig. 129) was discovered by a local collector farther north near Al Gualb Mesa several kilometers west of Zrigat (Ibrahim et al. 2014b, Fig. 129C). The first bones were excavated in 2008 and acquired a short time later by two of us (NI, SZ) from the collector and his associate. Due to the hardness of the surrounding matrix, the remainder of the specimen was collected over a couple of months. These bones comprising the majority of the specimen, were sold to an Italian geologist, who later transferred them to the Museo Civico di Storia Naturale in Milan. The connection between the first bones and this later material was

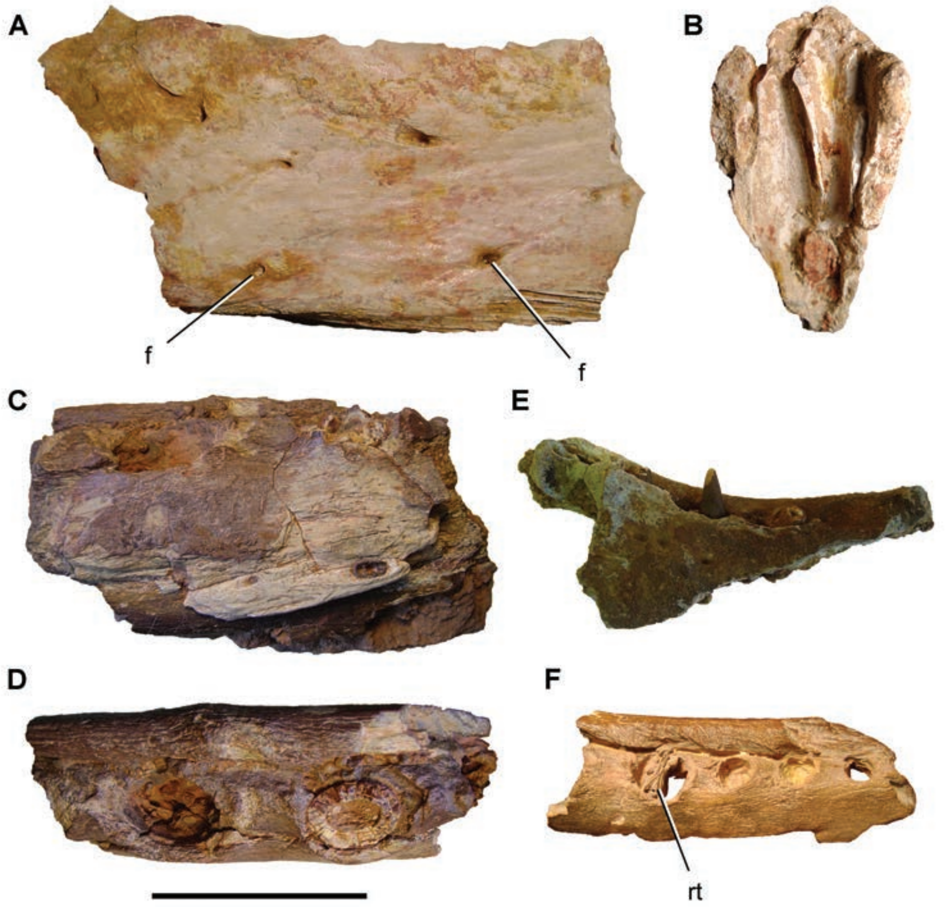

Figure 120. Dentary fragments of *Spinosaurus aegyptiacus* from the Kem Kem Group. Dentary section (MPDM 30) in (**A**) lateral and (**B**) ?anterior view. Dentary fragment (MNHN-MRS 1513) in (**C**) lateral and (**D**) dorsal view. Left dentary fragment with tooth (MSNM V6865) in (**E**) lateral view. Dentary fragment (MPDM 31, associated with *Onchopristis* rostral tooth) in (**F**) dorsal view. Scale bar equals 10 cm. Abbreviations: **f** foramen **rt** rostral tooth.

made by one of us (NI) in the course of collections research in Milan. The color of the adhering sandstone matrix was similar and the peculiar cross-sectional profile of the neural spines was identical.

In 2013 the Moroccan collector was located and took several of us (NI, DMM, SZ) to the site of discovery, which was located in a sandstone unit near the base of the Douira Formation. In 2014 the site was revisited for more intensive paleontological and geological work by several of us (NI, SZ, PCS, DMM) and others. The spoils and walls of the initial excavation were screened and further excavated. Teeth and additional fragments came to light. The locality was placed in section at the base of the Douira Formation, with a log of a very complete section across both formations compiled on the side of a nearby butte, Al Gualb Mesa (Ibrahim et al. 2014b).

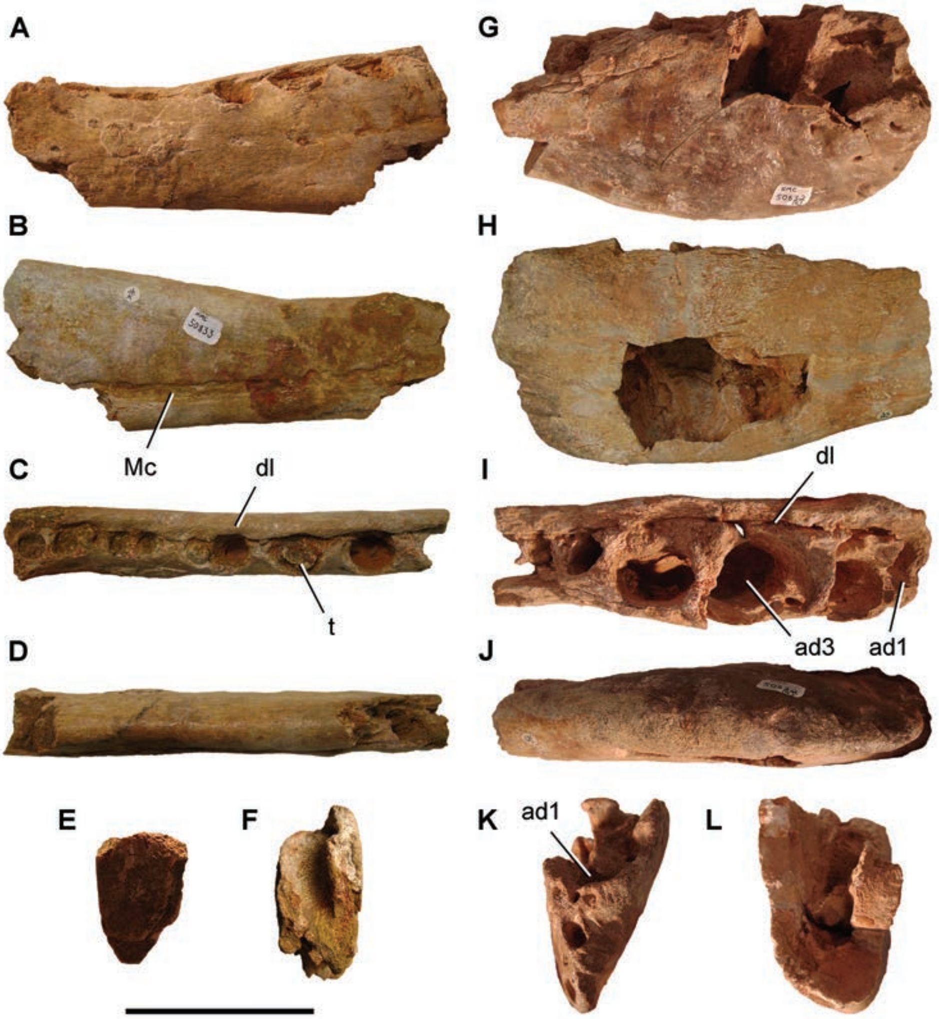

Figure 121. Dentary fragments of *Spinosaurus aegyptiacus* from the Kem Kem Group. Anterior section of the left dentary (NMC 50833) in (**A**) left lateral, (**B**) medial, (**C**) dorsal, (**D**) ventral, (**E**) anterior and (**F**) posterior view. Symphyseal end of the right dentary (NMC 50832) in (**G**) lateral, (**H**) medial, (**I**) dorsal, (**J**) ventral, (**K**) anterior and (**L**) posterior view. Scale bar equals 10 cm. Abbreviations: **ad1 3** alveolus for dentary tooth 1, 3 **dl** dental lamina **Mc** Meckel's canal **t** tooth.

***Spinosaurus aegyptiacus*, partial associated skeleton.** Evers et al. (2015: 70, 71) questioned the association of FSAC-KK 11888 as a single individual, claiming "there is no information on the original association of the remains, nor is there additional information on the locality". Neither claim is true. The specimen (FSAC-KK 11888) was found, like Stromer's specimens, by a commercial collector who did not document the precise positions of the bones in the field. Unlike the case with Stromer's material, however, there is a lot more evidence regarding the locality, how the specimen was

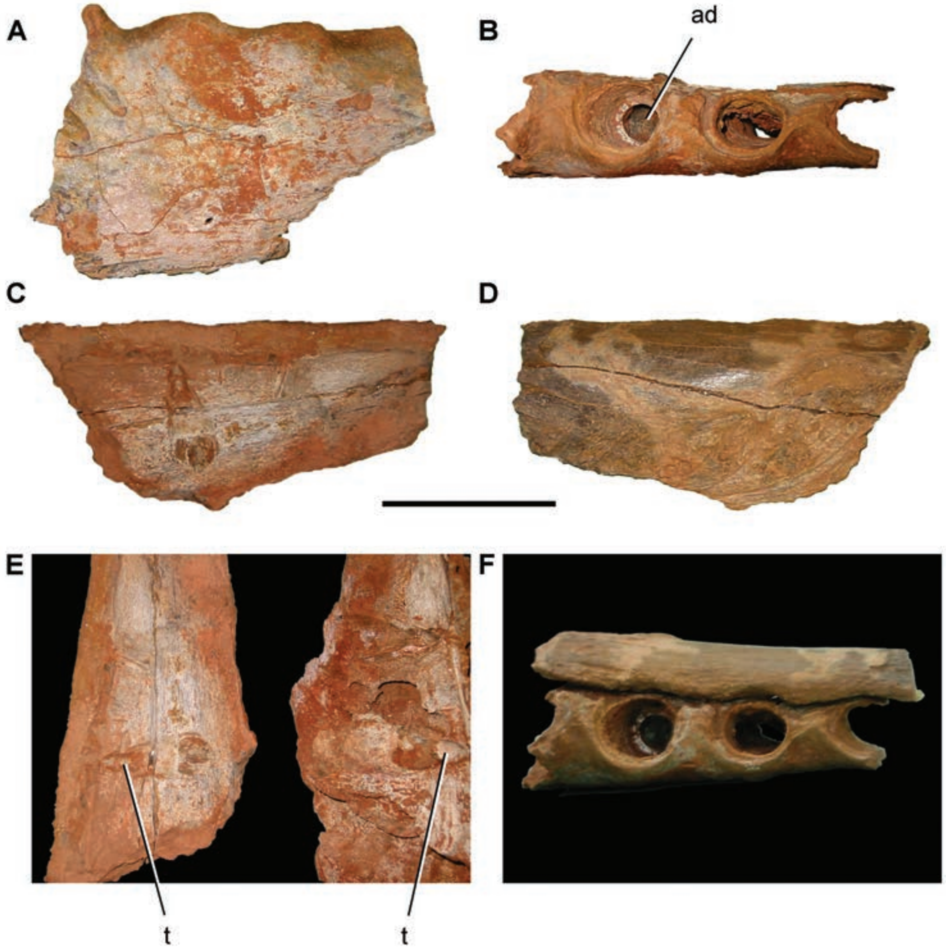

Figure 122. Dentary fragments of *Spinosaurus aegyptiacus* from the Kem Kem Group. Anterior section of the left dentary (IMPG 969-1) in (**A**) lateral and (**B**) dorsal view. Posterior section of the left dentary (IMPG 969-2) in (**C**) lateral and (**D**) medial view. Part and counterpart aligned in (**E**) lateral and (**F**) dorsal view. The two specimens clearly belong to the same jaw section and individual (*contra* Buffetaut 1989, 1992). Scale bar equals 10 cm in **A-D**. Abbreviations: **ad** alveolus in the dentary **t** tooth.

recovered, the condition of the material, and the nature of associated matrix. It was collected over a relatively short interval in 2008, transported a short distance before being sold in two lots. We have this account in detail from the original Moroccan collector and the Italian geologist who purchased the main lot of bones. Several of the current authors are familiar with the region where this specimen comes from, have interviewed the commercial collector and Italian geologist, have visited the locality, and have compared pieces found at the site to the original set of excavated bones. Most of the bones are only slightly crushed or compressed and preserve in places similar variably colored sandstone matrix.

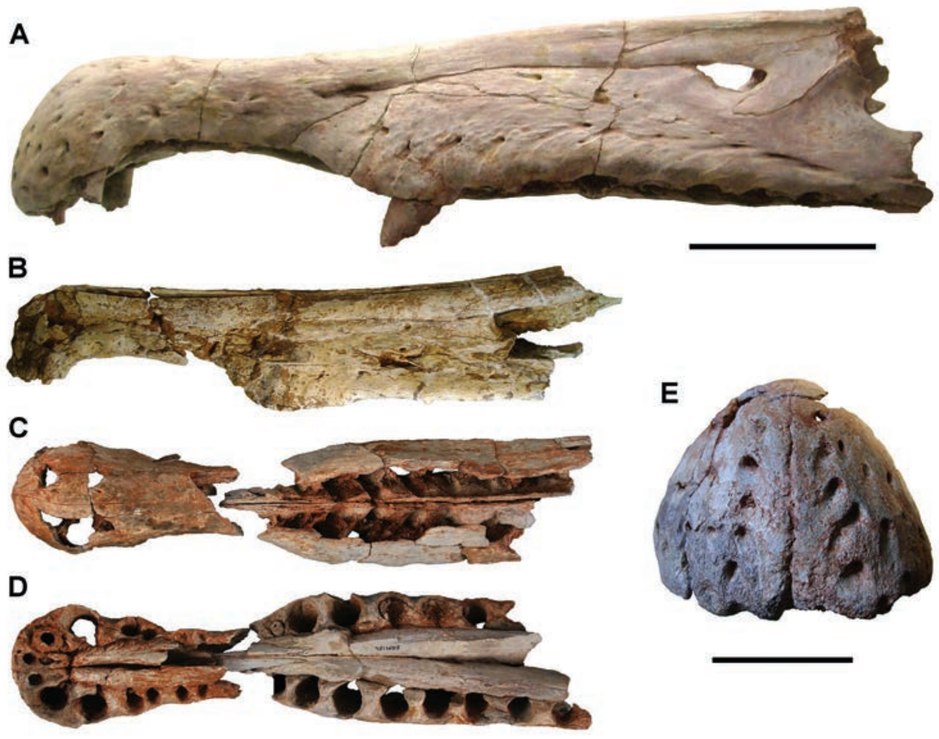

Figure 123. Comparison of *Spinosaurus aegyptiacus* rostra from Morocco and Algeria. Moroccan rostra include MSNM V4047 in (**A**) left lateral view and BMNH 16420 in (**B**) left lateral view. Algerian rostrum MNHN SAM 124 is shown in (**C**) dorsal, (**D**) ventral and (**E**) anterior view. Scale bars equal 20 cm in **A-D**, 10 cm in **E**.

Unlike Stromer's holotypic specimen, the quadrates and several of the excavated girdle and limb bones of the new specimen comprise perfectly matched opposites. All three bones of the pelvic girdle and both hind limbs have opposites of similar size. There are no duplicate bones. A portion of one of the manual digits, in addition, was found in articulation; the end of one phalanx was preserved in articulation with the base of the succeeding nonterminal phalanx, both adhered by matrix in position to a portion of a dorsal vertebra. The concavo-convex interphalangeal articulations in the right pes are precisely fitted, suggesting that at least portions of the hind limbs were also preserved in articulation when excavated. The preserved bones of the axial column also have compatible dimensions. Thus, the evidence from the preserved material and locality (*contra* Evers et al. 2015) suggests that several portions of the girdles and limbs were articulated when discovered. Much of the axial column appears to have been disarticulated *in situ*, although portions of the cervical, dorsal and caudal regions were concentrated within a few cubic meters of matrix. Of the skull, only a few teeth and cranial fragments were collected.

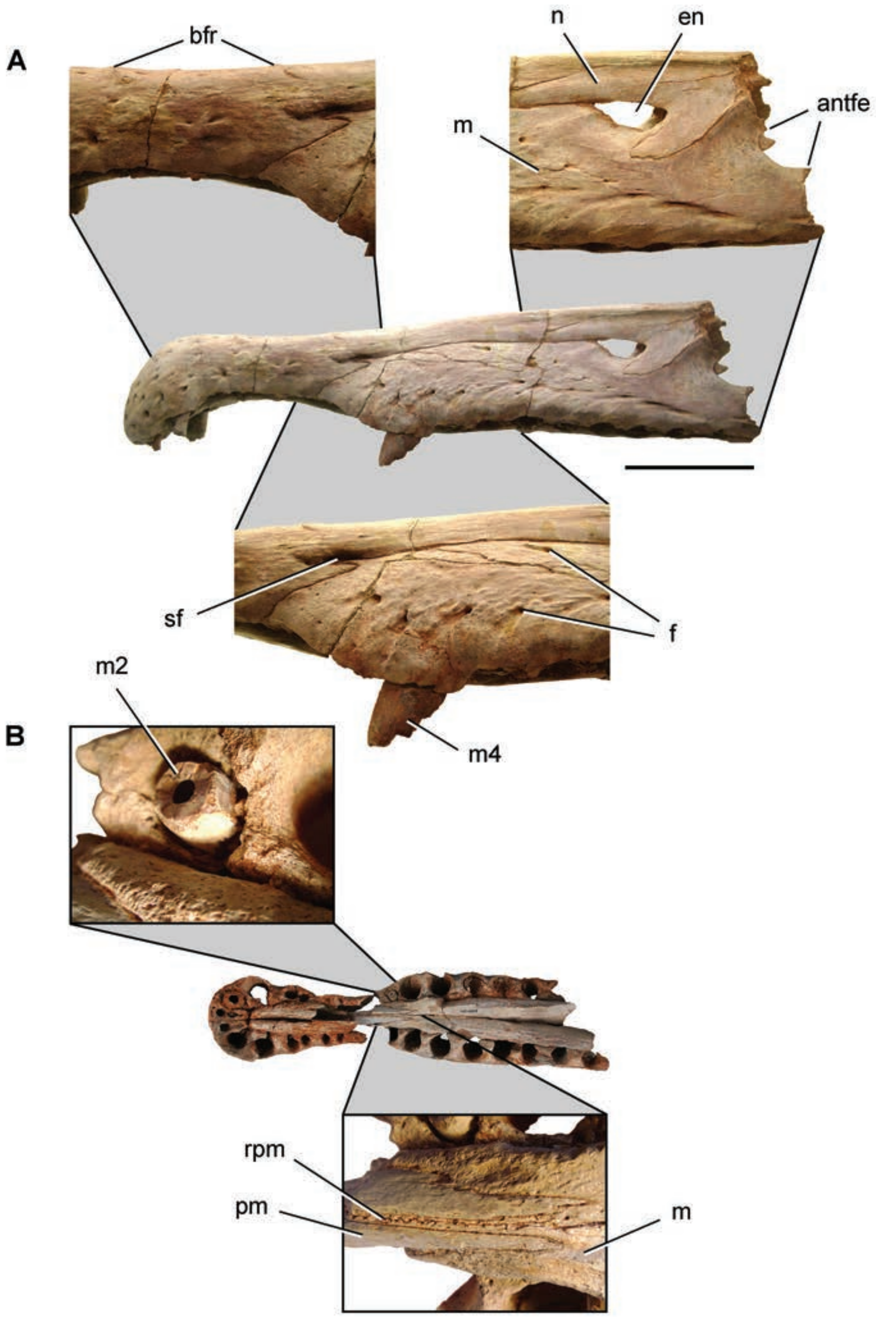

Figure 124. Rostral portions of *Spinosaurus* from Morocco and Algeria. Rostrum (MSNM V4047) in (**A**) left lateral view with enlarged sections. Rostrum (MNHN SAM 124) from Algeria in (**B**) ventral view with enlarged sections. Scale bar equals 20 cm for full lateral and ventral view. Abbreviations: **antfe** antorbital fenestra **bfr** bone fractures **en** external naris **f** foramen **m2**, **4** maxillary tooth 2, 4 **m** maxilla **n** nasal **pm** premaxilla **rpm** rostro-medial process of maxillae **sf** subnarial foramen.

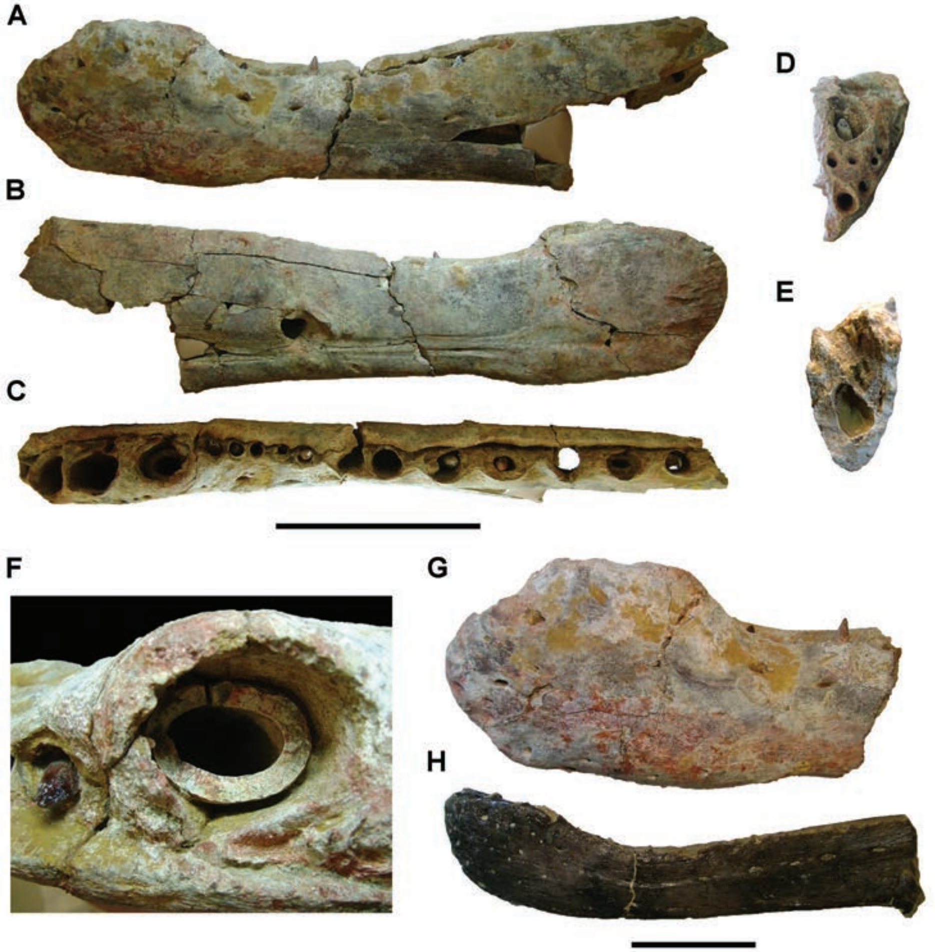

Figure 125. Dentary portion of *Spinosaurus aegyptiacus* from the Kem Kem Group**.** NHMUK VP 16421 in (**A**) left lateral, (**B**) medial, (**C**) dorsal (occlusal), (**D**) anterior and (**E**) posterior view. **F** Detailed view of dentary alveolus. Size comparison of (**G**) the anterior end of the dentary of *Spinosaurus aegyptiacus* (NHMUK VP 16421) and (**H**) the partial dentary of *Baryonyx walkeri* (NHMUK VP R9951). Scale bars equal 20 cm in **A-E**, 10 cm in **G** and **H**.

Evers et al. (2015: 71) also posited that "most vertebrates in the 'Kem Kem beds' are found in multi-taxonomic bone beds". They suggested, further, that the many matching bones of the new specimen may have been the handiwork of "local collectors" and "fossil dealers", who simply assembled the partial skeleton prior to sale. The first is not an accurate description of the disposition of most fossils in the Kem Kem Group, and the second is not a plausible hypothesis for the origin of the specimen. First, extensive bonebeds packed with paucispecific assemblages as found in Cretaceous deposits of North America (Eberth and Currie 2010) are not present in the Kem

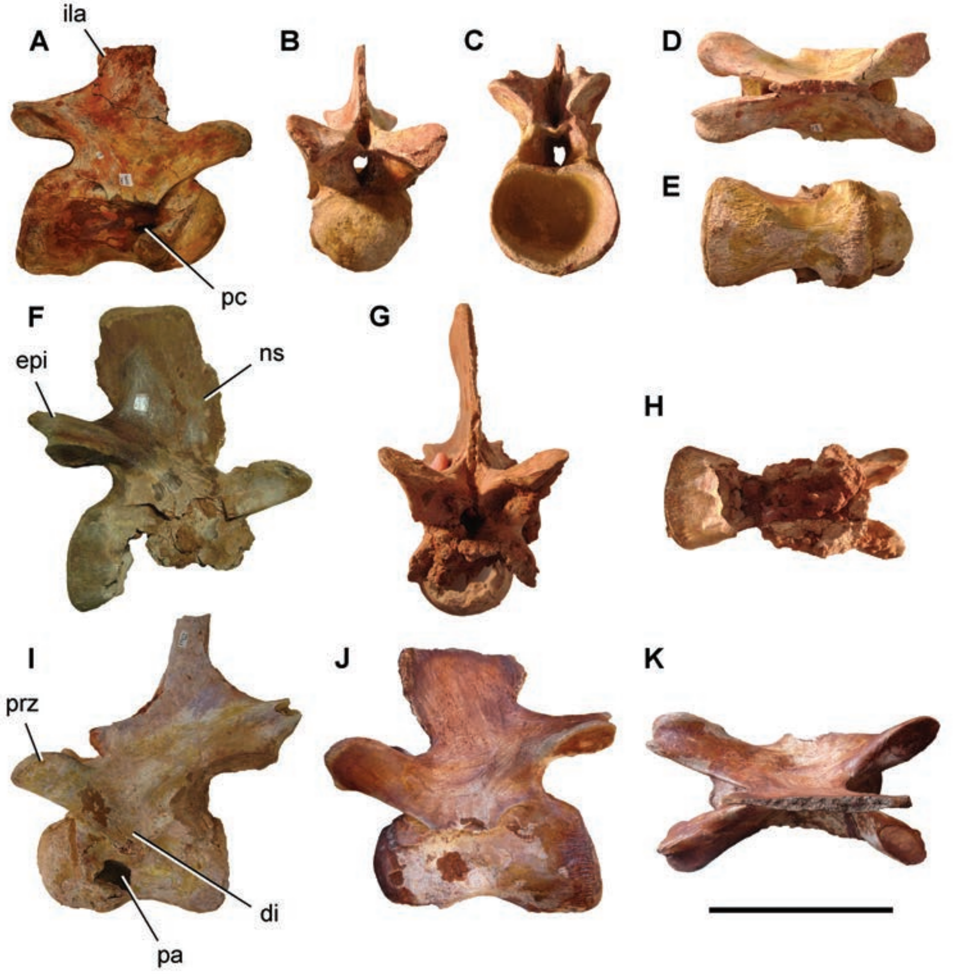

Figure 126. Cervical vertebrae of *Spinosaurus* cf. *aegyptiacus* from the Kem Kem Group. Mid cervical vertebra (NMC 50791) in (**A**) right lateral, (**B**) anterior, (**C**) posterior, (**D**) dorsal and (**E**) ventral view. Posterior cervical vertebra (NMC 50790) in (**F**) right lateral, (**G**) anterior and (**H**) ventral view. Mid cervical vertebra (NMC 41768) in (**I**) left lateral view. Posterior cervical vertebra (MPDM 33) in (**J**) left lateral and (**K**) dorsal view. Scale bar equals 20 cm. Abbreviations: **di** diapophysis **epi** epipophysis **ila** interspinous ligament attachment **ns** neural spine **pa** parapophysis **pc** pleurocoel **prz** prezygapophysis.

Kem Group. Channel lag deposits are most commonly the sedimentological setting mined by commercial collectors, which preserve isolated, transported teeth and bone pertaining to many species. The site yielding the new specimen, in contrast, is not a poorly cemented channel lag but rather a well cemented, massive sandstone preserving the remains of a single partially articulated individual. Most Kem Kem localities, furthermore, are located east and southwest of Taouz, as evidenced by the location and concentration of excavation tunnels and spoils. The new specimen, in contrast, comes

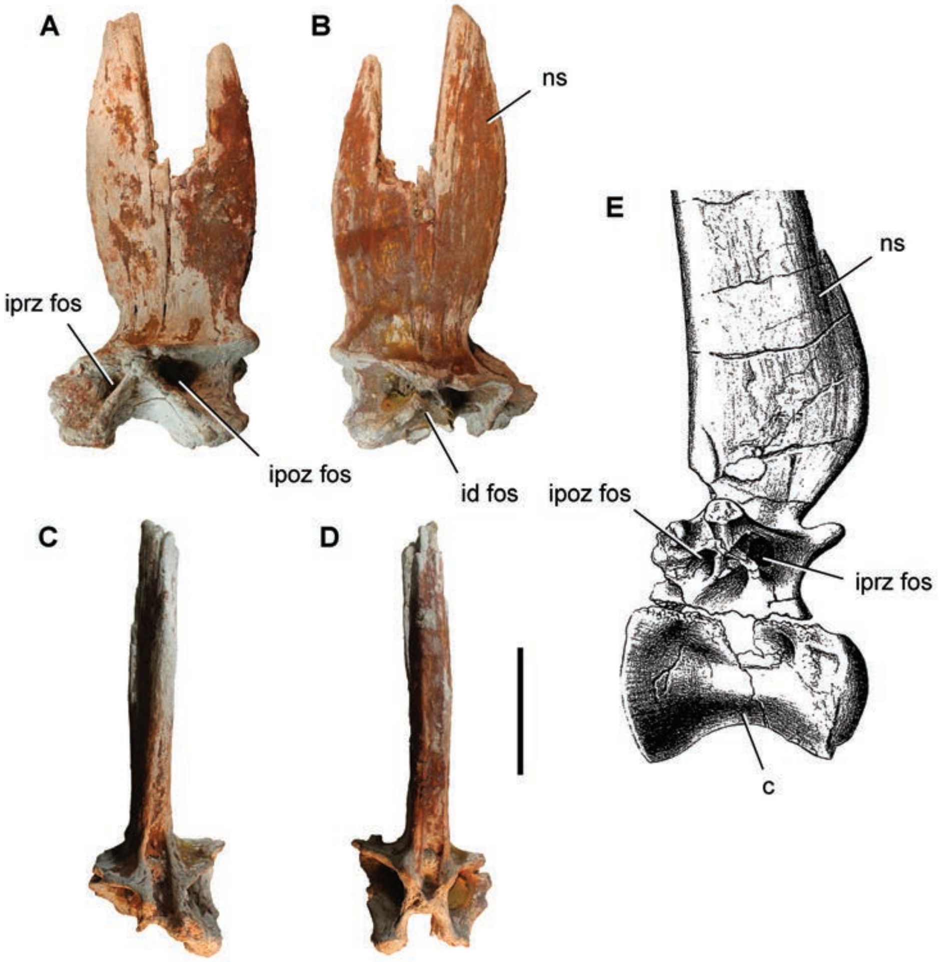

Figure 127. Dorsal vertebrae of *Spinosaurus aegyptiacus*. Anterior dorsal neural arch with partial spine (FSAC-KK 04) from the Kem Kem Group in (**A**) left lateral, (**B**) right lateral, (**C**) anterior and (**D**) posterior view. Ventral portion of a mid dorsal vertebra (Stromer 1915) from the Bahariya Formation in Egypt in (**E**) right lateral view. Scale bar equals 10 cm in **A-D**. Abbreviations: **c** centrum **id fos** infradiapophysial fossa **ipoz fos** infrapostzygapophysial fossa **iprz fos** infraprezygapophysial fossa **ns** neural spine.

from an isolated locality north of Taouz in a region with few additional localities. There were no accessory sites or fossils anywhere near the quarry site, which is perched on the sidewall of a ravine rather than an erosional surface that could concentrate fossil bone.

The bones of the new specimen could not plausibly have been assembled from a disparate collection of spinosaurid bones. The bones of the axial column are congruent in size, and paired girdle and long bones of similar size and preservation support interpretation of the material as pertaining to a single individual from a single locality. We regard this specimen of *Spinosaurus* as an associated partial skeleton. This specimen

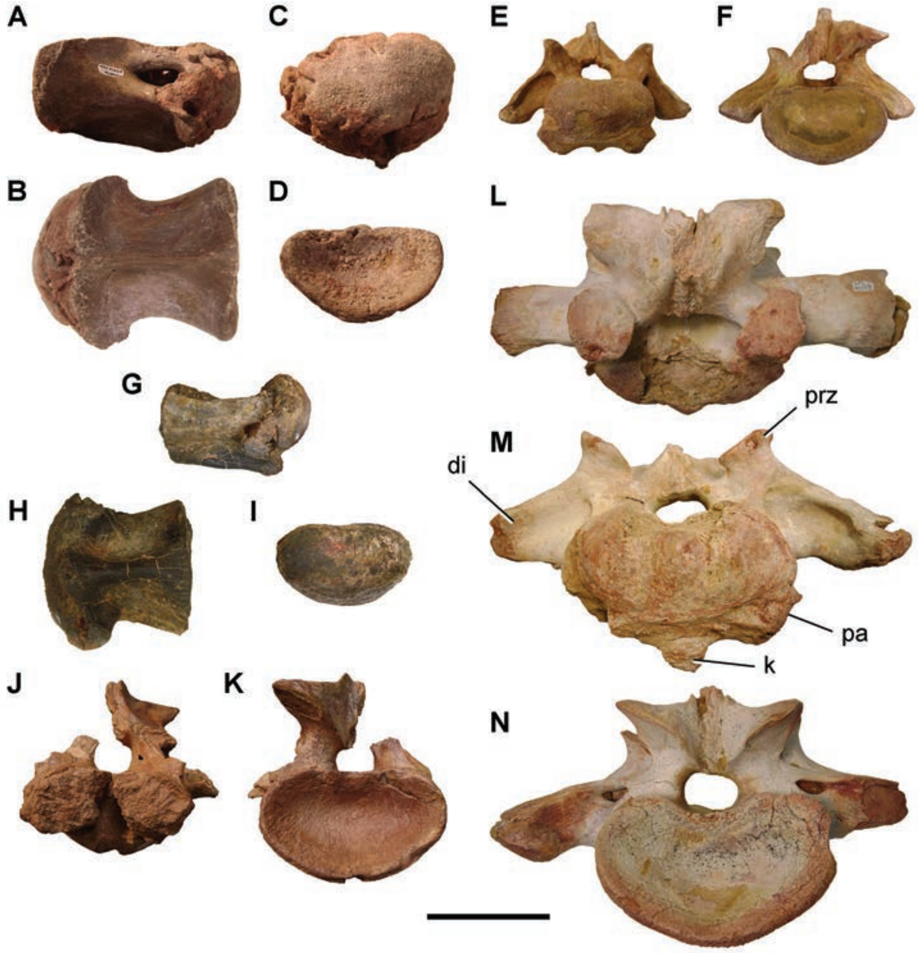

Figure 128. Problematic cervical vertebrae from Niger and Morocco referable to *Spinosaurus*. NMC 41629 in (**A**) right lateral, (**B**) ventral, (**C**) anterior and (**D**) posterior view. NMC 41774 in (**E**) anterior and (**F**) posterior view. MNN IGU 11 in (**G**) right lateral, (**H**) ventral and (**I**) posterior view. UCRC PV13 in (**J**) anterior and (**K**) posterior view. NMC 41857 in (**L**) dorsal, (**M**) anterior and (**N**) posterior view. Scale bar equals 10 cm. Abbreviations: **di** diapophysis **k** keel **pa** parapophysis **prz** prezygapophysis.

joins the holotypes of *Rebbachisaurus garasbae* (Wilson and Allain 2015) and *Deltadromeus agilis* (Sereno et al. 1996) as only the third partial articulated dinosaur skeleton recovered in the Kem Kem Group, the first from the Douira Formation, and the only one with both cranial and postcranial remains preserved.

A more plausible concern regarding associated specimens from the Kem Kem Group is actually opposite that imagined by Evers et al. (2015). Because field jackets and lab preparation are not employed by commercial collectors in the Kem Kem Group, associated bones of a partial skeleton or skull are easily overlooked, disassociated in the course of excavation, and sold individually. It is very doubtful that the associated cranial and

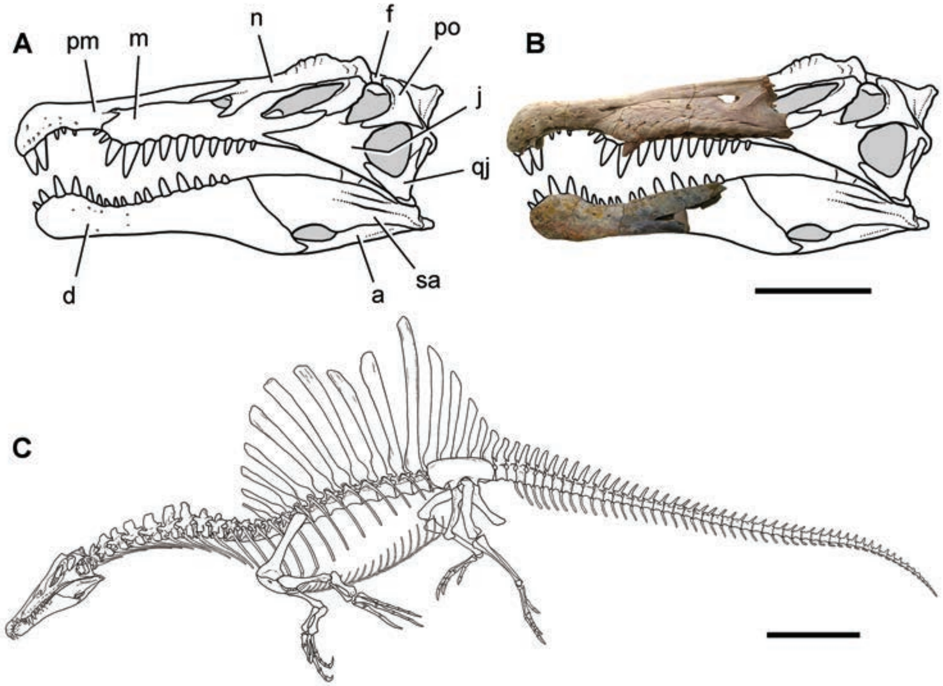

Figure 129. Skull and skeleton of *Spinosaurus aegyptiacus*. **A** Skull reconstruction in left lateral view (after Dal Sasso et al. 2005) **B** Skull reconstruction with adult rostrum (MSNM V4047) and dentary (NHMUK VP R 16421) superimposed **C** Skeletal reconstruction (swimming pose, after Ibrahim et al. 2014b). Scale bars equal 40 cm in **A**, **B** and 1 m in **C**. Abbreviations: **a** angular **d** dentary, **f** frontal, **j** jugal **m** maxilla **n** nasal **pm** premaxilla **po** postorbital **qj** quadratojugal **sa**, surangular.

postcranial specimens of *Carcharodontosaurus* and *Deltadromeus* (Sereno et al. 1996) would have survived as intact specimens if discovered by common commercial collectors. Compared to the original material from the Bahariya Formation in Egypt, the new partial skeleton stands as the most complete and best documented single specimen of *Spinosaurus aegyptiacus*, the first for this taxon to come to light in more than a century, and the first dinosaur specimen in the world, thus far, sold commercially, traced back to its original locality, and then repatriated to a recognized regional collection.

***Spinosaurus aegyptiacus*, neotype designation.** The partial skeleton of *Spinosaurus* (FSAC-KK 11888) preserves fragments of the skull, portions from all major parts of the axial column, portions or entire bones from both girdles, and portions or complete parts of both fore and hind limbs (Ibrahim et al. 2014b). Most importantly, it preserves cervical, dorsal and caudal vertebrae, a fibula, and pedal unguals that partially overlap with the Egyptian material described by Stromer (1915, 1934). These bones include the most salient, diagnostic features for the genus and species, *Spinosaurus aegyptiacus*, as noted by Stromer (1915: 28), features that recently were listed with other autapomorphies in a formal diagnosis (Ibrahim et al. 2014b).

Because critical portions of the skeleton overlap with that of Stromer's holotype from Egypt, the new Kem Kem specimen provides an excellent opportunity for neotype designation following standard guidelines (Ibrahim et al. 2014b, ICZN 1999: Art. 75.3). The morphology of the dorsal vertebrae, in particular, distinguishes all currently known spinosaurids that preserve this portion of the axial column. In *Spinosaurus aegyptiacus*, the mid dorsal vertebrae have elongate hourglass-shaped centra, neural spine height up to ten times greater than centrum height, maximum neural spine width near the spine base with a narrowed midsection, dense neural spine bone with a narrow elliptical cancellous zone in its center; and subtle vertical striae covering the proximal one-third of dorsal neural spines (Ibrahim et al. 2014b: suppl. material). The new specimen shares all of these autapomorphies with the holotypic specimen except the cross-sectional structure of the neural spine, which cannot be determined from Stromer's text and figures. Other autapomorphies are present on both the Bahariya and Kem Kem specimens and other referred material. A revised diagnosis for the species *S. aegyptiacus* was presented (Ibrahim et al. 2014b: suppl. material).

Evers at al. (2015) rejected this designation of a neotype without discussion of any of the diagnostic features noted above, which characterize only the original Bahariya and new Kem Kem specimens. The additional reasons given for rejecting the proposed neotype are questionable. The entirety of Stromer's holotypic specimen was destroyed in 1944, and since then no significant additional material of *S. aegyptiacus* has been discovered in the relatively small areas of outcrop available in the Western Desert of Egypt. Designation of a neotype partial skeleton found elsewhere is justified, given the frequent reference to this genus and species and the recovery of additional spinosaurid material from other regions of northern Africa. An existing specimen with greater skeletal coverage than any other is preferred as a type, rather than relying solely on a limited set of drawings and two photographs of the destroyed holotype.

Evers et al. (2015) also argued that other large theropods from the Kem Kem Group, namely *Carcharodontosaurus saharicus* and *Deltadromeus agilis*, are quite distinct from comparable fossils recovered from the Bahariya Formation, and so the spinosaurids may also be distinct. Even a cursory comparison does not support this conclusion. A partial skull of *C. saharicus* from the Douira Formation in Morocco was designated as a neotype for *Carcharodontosaurus saharicus*, because the bones that overlap those described by Stromer (1934), including the maxilla, nasal and braincase, are virtually indistinguishable (Sereno et al. 1996). Several autapomorphies were given for *C. saharicus* that are present in the original Bahariya and new Kem Kem specimens, such as the patterning and extent of external grooves and the laterally protruding ventral margin of the external antorbital fenestra (Brusatte and Sereno 2007). Such is not the case with material of *Carcharodontosaurus* discovered further south in Niger, which was attributed to a distinct species (Brusatte and Sereno 2007). *D. agilis* from the Gara Sbaa Formation in Morocco, likewise, has a diagnostic accessory trochanter and unusual condylar extension at the distal end of the femur, which are indistinguishable from a larger femur from the Bahariya Formation attributed to *Bahariasaurus ingens* (Stromer, 1934: pl. 3, fig. 5). That genus and species would have been applied to the

partial skeleton named *D. agilis* from the Gara Sbaa Formation, had the isolated femur, rather than an assortment of fragmentary axial and girdle bones of dubious association, been selected by Stromer as the name-bearing material (Sereno et al. 1996, see further discussion above under *Deltadromeus*).

Evers et al. (2015) also suggested that the geographic distance between the holotypic and neotypic localities for *Spinosaurus aegyptiacus* argues against neotypic designation. Geographic distance alone, however, is not a strong argument against overlapping, comparable fossil material, particularly when the two sites are bridged by many contemporary fossil-bearing localities of similar latitude along a continuous coastline. The great similarity of taxa shared between these two formations of similar latitude and general coastal setting is not surprising.

Milner (2003) has argued in favor of generic, if not specific, synonymy between *Suchomimus tenerensis* and *Baryonyx walkeri*, spinosaurids across much greater distances and latitude. These spinosaurids come from localities on different continents separated by more than 3,000 km and 40° of latitude. Not surprisingly, the material for these genera shows a number of differences, and the case for their synonymy cannot be maintained when comparing the material in detail. The case for referral to *Spinosaurus aegyptiacus* is different. We maintain that the broad skeletal overlap and great similarity between the holotypic specimen of *Spinosaurus aegyptiacus* and the partial skeleton from the Douira Formation justify designation of the latter as a neotype.

***Spinosaurus aegyptiacus,* lost holotype.** In 1944 all of the spinosaurid bones described by Stromer (1915, 1934, 1936) were destroyed in war, including the partial skeleton that formed the basis of *Spinosaurus aegyptiacus* (BSPG 1912 VIII 19). All that remains are Stromer's lithographic plates (1915: pl. 1, 2) and two photographs, the first of the right mandibular ramus in lateral view and the second a view of the holotypic specimen as mounted on a wall in the Bayerische Staatssammlung (Smith et al. 2006).

The bones of the holotype were collected by Richard Markgraf in 1912 (Nothdurft et al. 2002) and regarded by Stromer (1915) as a single individual. They were found in proximity of one another but disarticulated and eroding from the surface. Stromer questioned the association of the single anterior caudal vertebra but concluded that its shorter length and greater centrum diameter than preserved parts of the sacral vertebrae indicated the tail was robustly proportioned.

Rauhut (2003: 35, 36) questioned the association of the holotype, pointing to differences in the neural spine height between cervical and dorsal vertebrae, the lack of lamination and pneumatization exactly as seen in *Baryonyx* and *Suchomimus*, and the fact that some carcharodontosaurids such as *Acrocanthosaurus* also have tall dorsal neural spines. None of these reasons, however, constitute evidence against association of all of the holotypic material. Neural spine height clearly varies substantially between the cervical, dorsal and caudal regions in spinosaurids, as documented in *Suchomimus tenerensis*. With much additional spinosaurid material now available for other taxa such as *Baryonyx*, *Suchomimus* and *Ichthyovenator*, we know that the extent of lamination and pneumaticity varies not only along the presacral column of single individuals as might be expected, but also between spinosaurid genera. Recent authors have ac-

cepted the association of Stromer's holotype, including those discussing the validity of spinosaurid taxa in Morocco (Evers et al. 2015: 68).

"*Spinosaurus* B". A second, less complete partial skeleton (BSPG 1922 X 45) also was recovered by Markgraf at a single outcrop along the western foot of Djebel Harra in the Bahariya Formation (Stromer 1934: 7) and subsequently destroyed in war. Stromer remarked that, as with the holotype, some of the bones lay weathered on the surface and others were embedded nearby in a hard marl. Given the small size of the limb bones versus the vertebrae, Stromer regarded the material as pertaining to two individuals of an unnamed theropod closely related to *Spinosaurus*, designating it "*Spinosaurus* B" (Stromer 1934: 21). Overlap with the holotype of *Spinosaurus aegyptiacus*, however, is limited to two strongly pinched, spool-shaped dorsal centra (Stromer 1915, 1934) and teeth that were never figured.

The current controversy over the number of spinosaurids in the Kem Kem Group originates with the non-overlap between these two Egyptian specimens. Russell (1996) regarded the very broad, squat, deeply keeled cervicodorsal centra of "*Spinosaurus* B" as distinctive. Hence, he designated a comparable isolated vertebra from the Kem Kem Group, now identified as a first dorsal vertebra (Evers et al. 2015), as the holotype of a new genus and species (*Sigilmassasaurus brevicollis*), referring it to a new family (Sigilmassasauridae). There is no overlap, however, between Russell's holotype, likely D1, and the holotype of *Spinosaurus aegyptiacus*, which preserves only anterior cervical vertebrae (most likely C3, C4) and mid dorsal vertebrae (Stromer 1915).

The critical importance of the new specimen of *Spinosaurus aegyptiacus* from the Douira Formation is that it overlaps both the holotype of *Spinosaurus aegyptiacus* and "*Spinosaurus* B" and connects the axial column to the hindlimbs in a single individual, confirming both the initial development of aventral rugose platform on the centrum of a mid-cervical (Fig. 130A) and the diminutive size and girth of the hindlimb bones (Ibrahim et al. 2014b). There exists considerable overlap between the Kem Kem specimen and "*Spinosaurus* B", which compares closely in absolute size and form (Ibrahim et al. 2014b: Suppl. Material, fig. S2).

Evers et al. (2015: 71) recognized that "in both cases there is a set of matching vertebrae on the one hand, and a set of matching limb elements on the other hand". They claimed, however, that there are significant differences between the specimens and nothing diagnostic compared to other neotetanuran theropods. We cannot confirm those statements. The hindlimb bones are remarkably short relative to the dorsal centra in both specimens; the ratio of dorsal centrum length to tibial length differs by less than 4%. The neotype and "*Spinosaurus* B" share remarkably similar and distinctive morphology. The distal caudal vertebrae show the same, unique morphology of the neural spine, which is flattened anteroposteriorly rather than transversely. The femur exhibits the same striking morphology of the distal condyles, which are developed as transversely narrow processes separated by a spacious, ventrally expanding intercondylar fossa. The tibia also is similar with a gracile shaft, weak tibiofibular crest and cnemial expansion, and a similar subtriangular anterodistal fossa for the astragalus. The pedal ungual has a conspicuously flattened ventral surface as in the neotype. Two of the

four unguals in the right pes of the neotype show very gentle curvature of the ventral surface preserved in side view of the single ungual of "*Spinosaurus* B". The differences between the overlapping bones of these two specimens are minor and lie within an acceptable range of individual and preservation variation. All of these bones can be distinguished from the more typical neotetanuran condition in *Allosaurus* (Madsen 1976) or *Sinraptor* (Currie and Zhao 1993).

"*Spinosaurus maroccanus*" and an intact spinosaurid neck (*Suchomimus tenerensis*). Russell (1996: 356) designated a single mid cervical vertebra (CMN 50791) as the holotype of a new species, based solely on a supposed difference in ratio between centrum length and height of the posterior articular face. Russell (1996) based his new species on a proportionately longer centrum than the single cervical centrum figured by Stromer (1915) in the holotype of *Spinosaurus aegyptiacus*. Russell (1996) and Taquet and Russell (1998) then referred additional cranial and postcranial material to *S. maroccanus*, although there was no overlap with the holotypic specimen.

An articulated neck of of the Early Cretaceous spinosaurid *Suchomimus tenerensis* was recovered from the Elrhaz Formation of Niger (Fig. 130B). The vertebral column, which is the most complete, articulated cervicodorsal series known for any spinosaurid, shows that centrum length varies from 90 mm to 139 mm in postaxial cervical vertebrae. Given the changing proportions within the cervical series, a simple length-height centrum ratio in isolated cervical vertebrae cannot be the basis for distinguishing among spinosaurids. Likewise, there is no basis for the referral of additional material to "*Spinosaurus maroccanus*" that does not overlap with the holotypic mid cervical vertebra.

Evers et al. (2015: 54) referred Russell's mid cervical vertebra to "*Sigilmassasaurus brevicollis*", arguing that CMN 50791 has the anticipated greater centrum length than the more distal cervicodorsal vertebra designated as the holotype of "*S. brevicollis*". Key for these authors is the presence in CMN 50791 of one of the diagnostic features of "*S. brevicollis*", namely, the "elevated, rugose ventral platform" that is "clearly visible in lateral view". The rugose ventral platform or "plateau", they argued, is not present in *Spinosaurus aegyptiacus* (Stromer 1915), and so they regarded "*Spinosaurus maroccanus*" as a junior synonym of "*Sigilmassasaurus brevicollis*".

The mid cervical vertebra of the neotypic specimen of *Spinosaurus aegyptiacus* (FSAC-KK 11888), nonetheless, has the same absolute centrum length and proportions as the holotype of "*Spinosaurus maroccanus*" and also has a clearly demarcated, rugose ventral platform (Fig. 130A). The platform is gently convex and not quite as flat as in CMN 50791. These vertebrae, otherwise, are remarkably similar in the size and position of the pleurocoel and parapophysis, the size and orientation of the base of the transverse process and neural spine, and the size, shape and orientation of the prezygapophyses. There are no comparable mid cervical vertebrae in the holotype of *Spinosaurus aegyptiacus* or in other material from Egypt (Stromer 1915, 1934). The neotypic specimen bridges an important comparative gap and shows that at least some cervical vertebrae in *Spinosaurus aegyptiacus* have a rugose, elevated ventral platform. The similarity between these vertebrae suggests that "*Spinosaurus maroccanus*" is best interpreted as a junior synonym of *Spinosaurus aegyptiacus*.

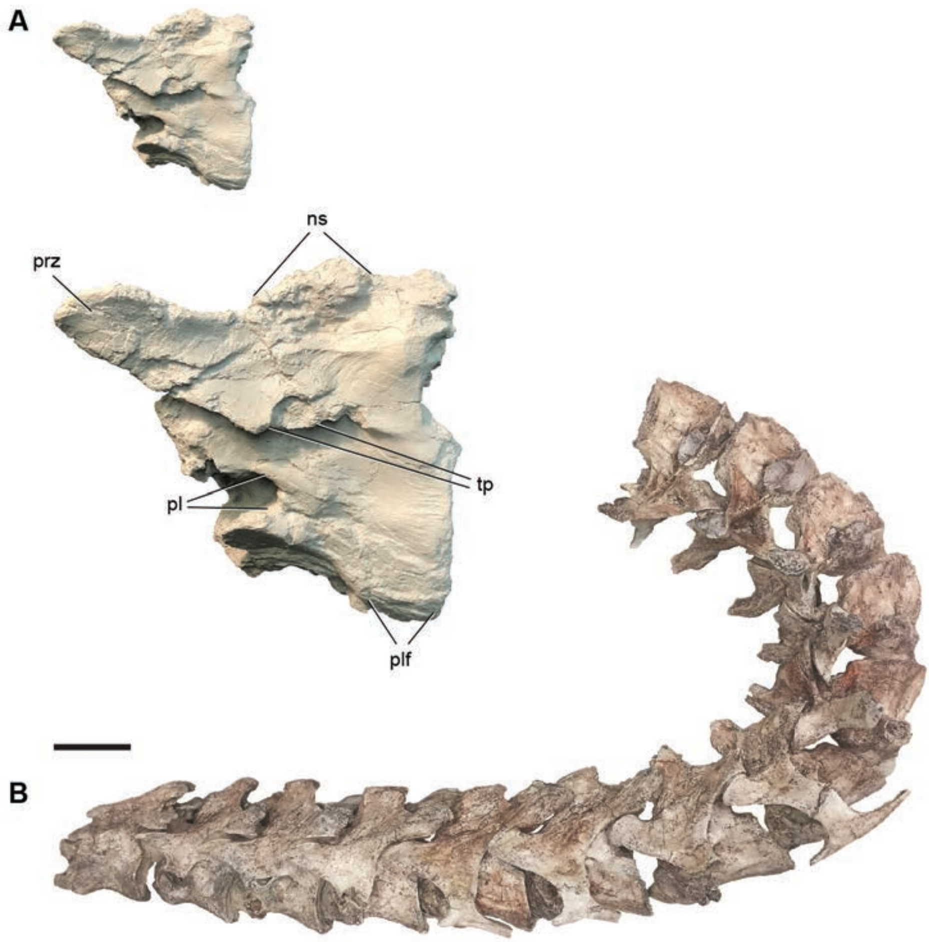

Figure 130. Spinosaurid cervicodorsal vertebrae. **A** Partial mid cervical of *Spinosaurus aegyptiacus* (FSAC-KK 11888, cast) lacking the anterior end of the centrum, upper portion of the neural spine and postzygapophyses, **B** Articulated cervicodorsal series C2-D4) of *Suchomimus tenerensis* (MNBH GAD70) in left lateral view with most of the cervical ribs removed. Scale bar equals 5 cm in **A** (bottom), 10 cm in **A** (top) and **B**. Abbreviations: **ns** neural spine **pl** pleurocoel **plf** platform **prz** prezygapophysis **tp** transverse process.

"*Sigilmassasaurus*". Erected on a single isolated vertebra, Russell (1995) and Evers et al. (2015) argued in favor of a second large-bodied spinosaurid in the Kem Kem Group named "*Sigilmassasaurus brevicollis*". The hypothesis that two large-bodied spinosaurids are present in the Kem Kem Group (Russell 1995) relies on three points (Evers et al. 2015). First, the integrity and taxonomic indentity of the partial skeleton FSAC-KK 11888 as a single individual representing *Spinosaurus aegyptiacus* must be disproved, because it exhibits features of both *Spinosaurus* and "*Sigilmassasaurus*" and reinforces and extends the skeletal features diagnostic of the original type species, *Spinosaurus aegyptiacus.* We outline above, to the contrary, considerable evidence

supporting the association of the neotypic specimen as a single individual. The specimen overlaps many of the bones of the holotypic specimen of *Spinosaurus aegyptiacus* (Stromer 1915), including those exhibiting its most outstanding autapomorphies. It also overlaps several bones of Stromer's less complete second specimen ("*Spinosaurus* B"), suggesting that it, too, may pertain to a single individual of the same species. Finally, a mid-cervical of the neotypic specimen (Fig. 130A) also overlaps the holotypic mid cervical of "*Spinosaurus maroccanus*", showing that a rugose, raised platform is present in at least some mid cervical vertebrae of *Spinosaurus aegyptiacus*. The raised ventral platform is the only feature in the emended diagnosis given for "*Sigilmassasaurus brevicollis*" (Evers et al. 2015) that occurs outside the missing transitional portion of the cervicodorsal column of *Spinosaurus aegytriacus*. And the platform is clearly present in the neotypic mid cervical vertebrae of *Spinosaurus aegytiacus* (Fig. 130A). The complete cervicodorsal series for *Suchomimus tenerensis* (Fig. 130B) shows the marked transition in vertebral form (length, ventral rugosities or platform, ventral keel, stoutness and angulation of the transverse process, etc.) from the mid cervicals through the anteriormost four dorsals, a transformation in a suite of features that may characterize all spinosaurids.

Second, "*Sigilmassasaurus brevicollis*", which is based on an isolated anterior dorsal vertebra (Russell 1996, McFeeters et al. 2013, Evers et al. 2015), must be shown to be distinct from *Spinosaurus aegyptiacus*. Unfortunately, the holotypic and neotypic specimens of *Spinosaurus aegyptiacus* do not include any posterior cervical or anteriormost dorsal vertebrae, and so the comparison cannot be made. The broad, squat centrum of Stromer's "*Spinosaurus* B" is very similar to centra found in Cenomanian rocks in Niger (Brusatte and Sereno 2007). Are there also two large-bodied, contemporary spinosaurids in the Bahariya Formation in Egypt and Echkar Formation in Niger?

Third, three papers have suggested there are two spinosaurids in the Kem Kem Group based on differences in preserved snouts and isolated frontals and quadrates. All are compromised by stratigraphic or identification issues that undermine support for two contemporary spinosaurids. Taquet and Russell (1998) described a snout from Algeria (Fig. 123C-E) from beds often regarded as older (Albian) than the Kem Kem Group (Cenomanian). They referred the snout, without citing any evidence, to "*Spinosaurus maroccanus*" from the Kem Kem Group, a species based on an isolated vertebra (Russell 1996). The contour of the alveolar margin and the size of some of the teeth, indeed, look slightly different than the condition in a large snout of *Spinosaurus aegyptiacus* from the Kem Kem Group (Hendrickx et al. 2016: fig. 11). The point we underscore here, however, is that the Algerian snout does not come from the Kem Kem Group and may well be older.

Hendrickx et al. (2016: fig. 3) suggested that there are two spinosaurid quadrate morphotypes in the Kem Kem Group, assigning five quadrates of varying completeness to *Spinosaurus aegyptiacus* based on overlap with the neotypic specimen. They assigned one fragmentary specimen to "*Sigilmassasaurus brevicollis*". This specimen, which preserves only the lower one-third of the bone and differs from the other quadrates in some details, cannot be assigned to "*Sigilmassasaurus brevicollis*", a taxon based

on an isolated vertebra. Furthermore, we are not confident it pertains to a spinosaurid. The shaft angles form the condyles as in spinosaurids, but more of the bone is needed to be sure it pertains to a spinosaurid.

Finally, two frontal morphs were described by Arden et al. (2019). The first, tentatively referred to ? *Spinosaurus aegyptiacus,* resembles spinosaurid frontals, which are best known in *Suchomimus* and *Baryonyx*. The frontal is triangular, has an arched orbital roof exposed in lateral view, small ovate cerebral fossae and a narrow olfactory tract. The most complete specimen described by Arden et al. (2019: fig. 2E-H) is a frontoparietal, and it clearly does not pertain to a spinosaurid. The parietal is very long with an equally long sagittal crest bounding very broad and expansive supratemporal fossae. The braincase would continue a considerable distance posterior to the frontal, a condition resembling the skull roof of a marine crocodyliform. Spinosaurids such as *Baryonyx*, *Suchomimus* and *Irritator* have anteroposteriorly short parietals and small supratemporal fossae.

A second large specimen consists of two, broad fused frontals that much more closely resemble a carcharodontosaurid than a spinosaurid (Arden et al. 2019: fig. 3). The posterior end of the frontals is upturned, suggesting that the parietals and nuchal wedge of the supraooccipital projected dorsally in that region. The frontals are broad with a marked median crest and lack a deep embayment on each side for the prefrontals. This construction is unusual for a spinosaurid. We are not convinced that the orbital margin, the roof of which is not markedly exposed in lateral view, is free of other roofing elements. This specimen may belong to a carcharodontosaurid. Referral to "*Sigilmassasaurus brevicollis*", a taxon based on an isolated vertebra, is not supported. In sum, we are not convinced that the isolated spinosaurid material in the Kem Kem Group supports the presence of more than a single large-bodied spinosaurid.

Two-piece puzzle. Are the squat cervicodorsal vertebrae of "*Spinosaurus* B" and "*Sigilmassasaurus brevicollis*" simply the missing parts of the axial column of the original spinosaurid, *Spinosaurus aegyptiacus*? We favor this hypothesis. Evers et al. (2015: 54) acknowledged that "drastic changes" in morphology occur along the cervical series in the best known spinosaurids (*Baryonyx*, *Suchomimus*, *Ichthyovenator*). Nonetheless, they promoted the assignment of Kem Kem spinosaurid material, and presumably the Egyptian material as well, to two genera of spinosaurids, based on their interpretation of the location of isolated vertebrae within the cervical series. Following this interpretation, they reconstructed the cervicodorsal series for "*Sigilmassasaurus brevicollis*" with low neural spines quite similar to that in *Baryonyx* or *Suchomimus* (Evers et al. 2015: fig. 25). They suggested that the companion spinosaurid to "*Sigilmassasaurus*" would be characterized by taller, rectangular shaped neural spines, stronger epipophyses, a small centroprezygapophyseal fossa medial to the base of the prezygapohpysis, and no development of a rugose, elevated ventral platform on the posterior half of the centrum (Evers et al. 2015: fig. 19). Yet all of these features are emphasized in more anterior cervical vertebrae. Some vertebrae, furthermore, appear to be transitional showing some, but not all, of these features. The mid cervical vertebra of the neotype of *Spinosaurus aegyptiacus* (Fig. 130A) has a broad spine base (broken) and, thus, presumably would

have had a fairly tall neural spine. It has a low centrodiapophyseal lamina and shows partial development ventrally of a rugose elevated platform.

To better understand how vertebral form changes along the neck, we briefly describe a complete cervicodorsal series from a partial articulated skeleton of *Suchomimus tenerensis* (Fig. 130B). Ten cervical and four anterior dorsal vertebrae are preserved in articulation. A marked change in the length and strength of the ribs define the cervicodorsal boundary; the ribs were removed to expose the centra in lateral view. Centrum length is shortest in C3 and longest in C7-C9, with intervening and more distal cervical vertebrae fairly similar in length. Centrum length, thus, would not help position isolated mid or posteriormost cervical vertebrae. Centrum length decreases in the anterior dorsals from D1 to D4. A rugose triangular scar is present on the ventral side of the posterior half of the centrum in C2-C9, foreshadowing the rugose elevated platform in "*Sigilmassasaurus brevicollis*". The rugosities are strongest in C9. More posteriorly in C10-D4, the rugosity recedes and is replaced by a prominent medial keel, which is deepest in D1. D1 alone has a fossa ventral to its enlarged, circular parapophysis and a particularly stout, ventrally deflected transverse process. Epipophyses, which are bulbous and prominent in anterior cervicals, decrease in size distally, disappearing posterior to D3. The neural spine is subrectangular in C4-C7 with tallest proportions in C4. The neural spine decreases in anteroposterior width posterior to C7, reaching its smallest peg-like form in D1 and D2. In C10 and D1, the ligament scar on the posterior aspect of the neural spine is set deep within a fossa, its ventral end very close to the roof of the neural canal as in "*Sigilmassasaurus*". The series is similar to comparable disarticulated vertebrae of *Baryonyx* (Charig and Milner 1997). Unlike *Baryonyx*, however, none of the cervical centra in *Suchomimus* have posterior articular faces that are broader than tall.

The *Suchomimus* cervicodorsal series suggests that centrum proportions alone may not securely position vertebrae in the cervical series. All of the anterior dorsal vertebrae (D1-D4), likewise, are remarkably short. The rectangular shape and height of neural spines appears to be greatest in C3 and C4, decreasing markedly in height and width from C8-D2. In *Spinosaurus aegyptiacus*, the anterior cervical neural spines (C3 and C4) were relatively taller, as preserved in the holotype of *Spinosaurus aegyptiacus* (Stromer 1915) and some isolated vertebrae from the Kem Kem Group (Russell 1996, Evers et al. 2015). The neural spines of posterior cervical and anterior dorsal vertebrae, however, seem to be low and rudimentary in all spinosaurids to accommodate dorsiflexion of the neck. Reduction of the bony roof over the neural canal, which brings the ligament scar of the neural spine near the neural canal, occurs in a pair of vertebrae at the junction of the neck and trunk (C10, D1). This condition, possibly to enhance dorsiflexion of the neck, was previously described as a unique feature of "*Sigilmassasaurus brevicollis*". The triangular rugosity on the posterior half of the ventral aspect of the centrum increases in strength in the posterior cervicals and appears to be the precursor to the raised platform seen in vertebrae from the Kem Kem Group. The transitional morphology observed in the vertebral series for *Suchomimus* provides a blueprint of changing vertebral morphology that links together the various vertebral

forms observed in the Kem Kem Group. As a consequence, we regard "*Sigilmassasaurus brevicollis*" as a junior synonym of *Spinosaurus aegyptiacus*.

Carcharodontosauridae Stromer, 1931. When more complete cranial material of the African genus *Carcharodontosaurus* came to light from the Douira Formation, Sereno et al. (1996) recognized carcharodontosaurids as a global Cretaceous-age radiation of distinctive allosauroid theropods, including the closely related South American genus *Giganotosaurus* (Coria and Salgado 1995, Calvo and Coria 1998) and the somewhat older North American genus *Acrocanthosaurus* (Harris 1998, Currie and Carpenter 2000). Since then a large amount of carcharodontosaurid fossil material has come to light. Some specimens represent close relatives, such as *Mapusaurus* from Argentina (Coria and Currie 2006). Other material represents basal carcharodontosaurids from Lower Cretaceous rocks, such as *Concavenator* from Spain (Ortega et al. 2010), *Eocarcharia* from Niger (Sereno and Brusatte 2008) and *Neovenator* from England (Brusatte et al. 2008).

Carcharodontosaurus. Two blade-shaped teeth with a nearly straight distal carina and marginal enamel wrinkles were described as *Megalosaurus saharicus* (Depéret and Savornin 1927). They were discovered at two locales ca. 3 km apart near the oasis of Timimoun in east central Algeria (Fig. 1), approximately 450 km west of the Kem Kem outcrops in Morocco. The teeth, which surely pertained to two individuals, were subsequently lost, and no casts were made. The teeth, which were well illustrated by Depéret and Savornin, no longer exhibit features regarded as diagnostic at the specific or generic level (Brusatte and Sereno 2007). Similar teeth are relatively common in the Kem Kem Group (Fig. 131).

Stromer (1931: pl. 1) described very similar teeth associated with a fragmentary cranium (braincase, maxilla, nasal) and several postcranial bones (several vertebrae, a chevron, proximal ischium, both femora, and a fibula). These were collected in 1914 by his assistant Markgraf near the Bahariya oasis in the Western Desert in Egypt and sustained damage in transport to Germany. Although no field map exists, Stromer was confident the bones belonged to a single individual of the species described by Depéret and Savornin, which he placed in a new genus *Carcharodontosaurus* (*Megalosaurus*) *saharicus*. Stromer's specimen, unfortunately, was destroyed in 1944 during World War II (Nothdurft et al. 2002). The only surviving piece is the endocast from the braincase (Fig. 132E, F).

In 1995 a more complete cranium (Figs 133–137) was discovered in a sandstone in the lower one-third of the Douira Formation at the locality Iferda N'Ahouar near Er Remlia (Sereno et al. 1996). The braincase was exposed with portions of the remainder of the cranium embedded in the cliff. Preserved bones include both maxillae (Fig. 133), nasals (Fig. 134A-C), lacrimals (Fig. 134D, E), jugals (Fig. 135) and postorbitals (Fig. 136). Several of these bones were near their natural articulation. The palate and lower jaw were represented by single bone pieces, and nothing from the postcranial skeleton was recovered. The bone configuration at the site suggests that the postcranial skeleton was not present.

The Moroccan cranium (UCRC PV12) closely resembles Stromer's specimen from the Western Desert of Egypt. The braincase has an extremely prominent nuchal crest

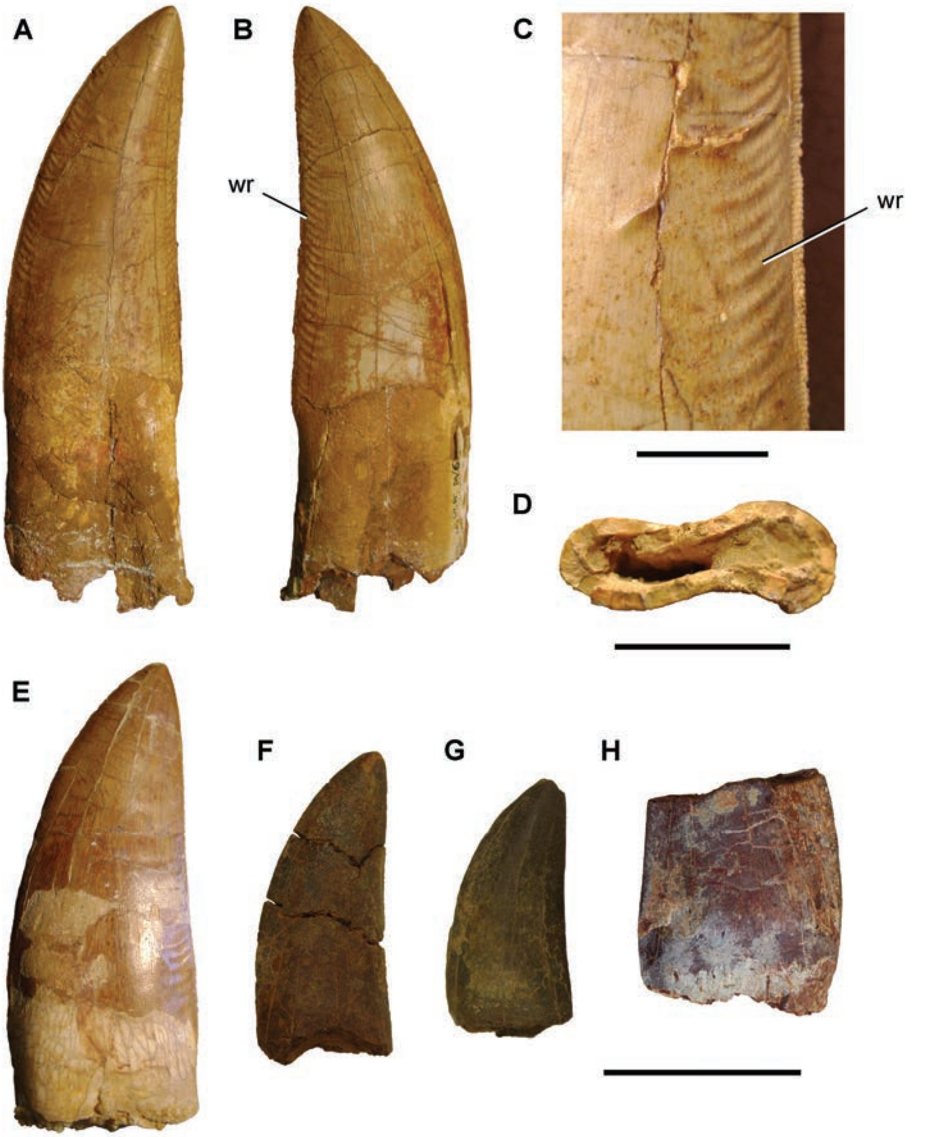

Figure 131. Teeth from Morocco and Niger referred to *Carcharodontosaurus*. Teeth of UCRC PV12 (*C. saharicus*) in (**A**) labial and (**B**) lingual view. **C** Detail of wrinkled enamel **D** Cross-sectional view. **E** MPDM 16 in lingual view. **F** Tooth (UCRC PV163) in lingual view. **G** Tooth (UCRC PV164) in lingual view. **H** FSAC-KK 907 in labial view. Scale bars equal 5 cm in **A** and **B**, and **E-H**, 1 cm in **C** and 3 cm in **D**. Abbreviation: **wr** wrinkled enamel.

that projects vertically at a right angle to the skull roof (Stromer 1931: pl. 1, fig. 4). The anterior surface of this vertical projection, formed by the parietal, extends slightly forward, overhanging the dorsal skull roof (Sereno et al. 1996: fig. 2B). A subtriangular posterior process of the parietal caps the dorsal surface of the supraoccipital nuchal

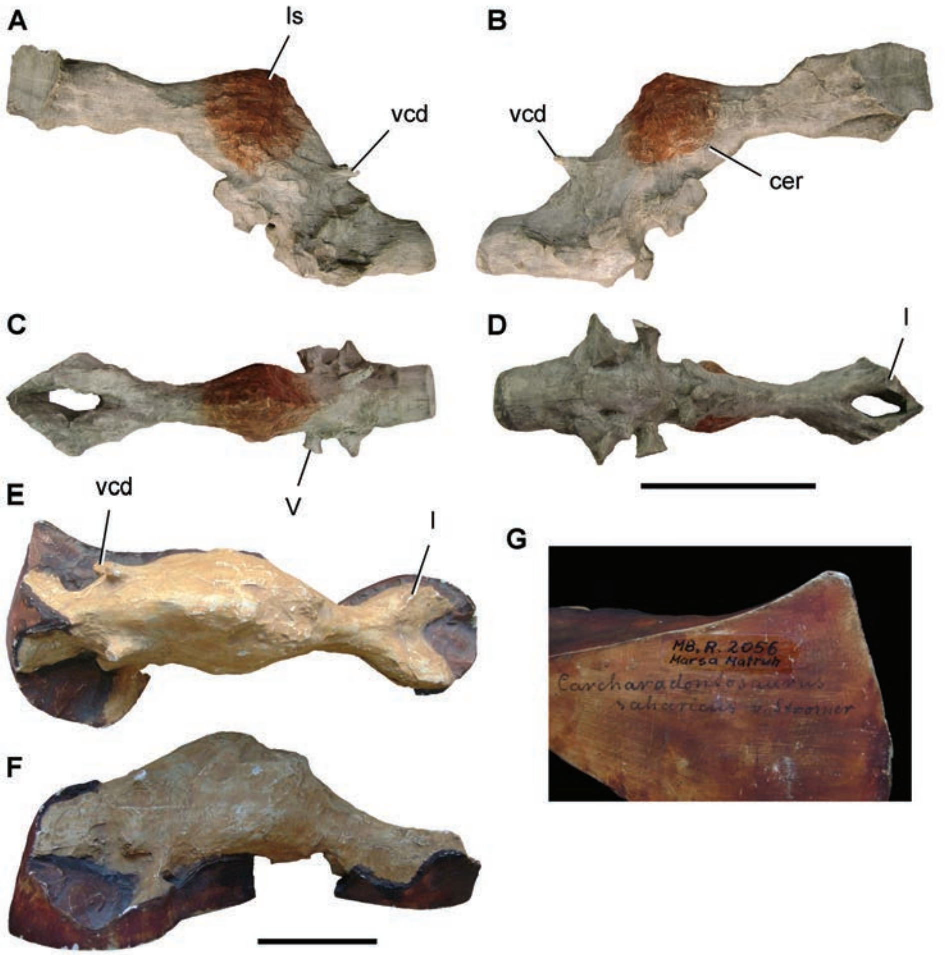

Figure 132. Endocasts of *Carcharodontosaurus saharicus*. Endocast (from UCRC PV12) in (**A**) left lateral, (**B**) right lateral, (**C**) dorsal and (**D**) ventral view. Endocast (MB. R. 2056) in (**E**) dorsal, (**F**) right lateral views and (**G**) bottom of cast. Scale bars equal 10 cm in **A-D**, 5 cm in **E** and **F**. Abbreviations: **cer** cerebrum **ls** longitudinal sinus **vcd** vena capitus dorsalis **I**, **V** cranial nerves I and V.

process. Comparable parts of the braincase of *Giganotosaurus* are different; the nuchal crest is less prominent, the parietal posterior process is tongue-shaped, and the supraoccipital nuchal process is broadly exposed to each side of the parietal process (Coria and Currie 2003: figs 1, 3, 5, 6). The maxilla (Fig. 133) is virtually identical to that figured by Stromer (1931: pl. 1, fig. 6) but differs in details from maxillae of *Giganotosaurus* (Coria and Salgado 1995) and *Mapusaurus* (Coria and Currie 2006). The maxilla (Fig. 133) is particularly deep proximally; the anteromedial process is positioned very high; the anterior sutural margin for the premaxilla slopes at an angle of ca. 65° to the alveolar margin, the sutural margin for the premaxilla is broadly exposed in medial view; the

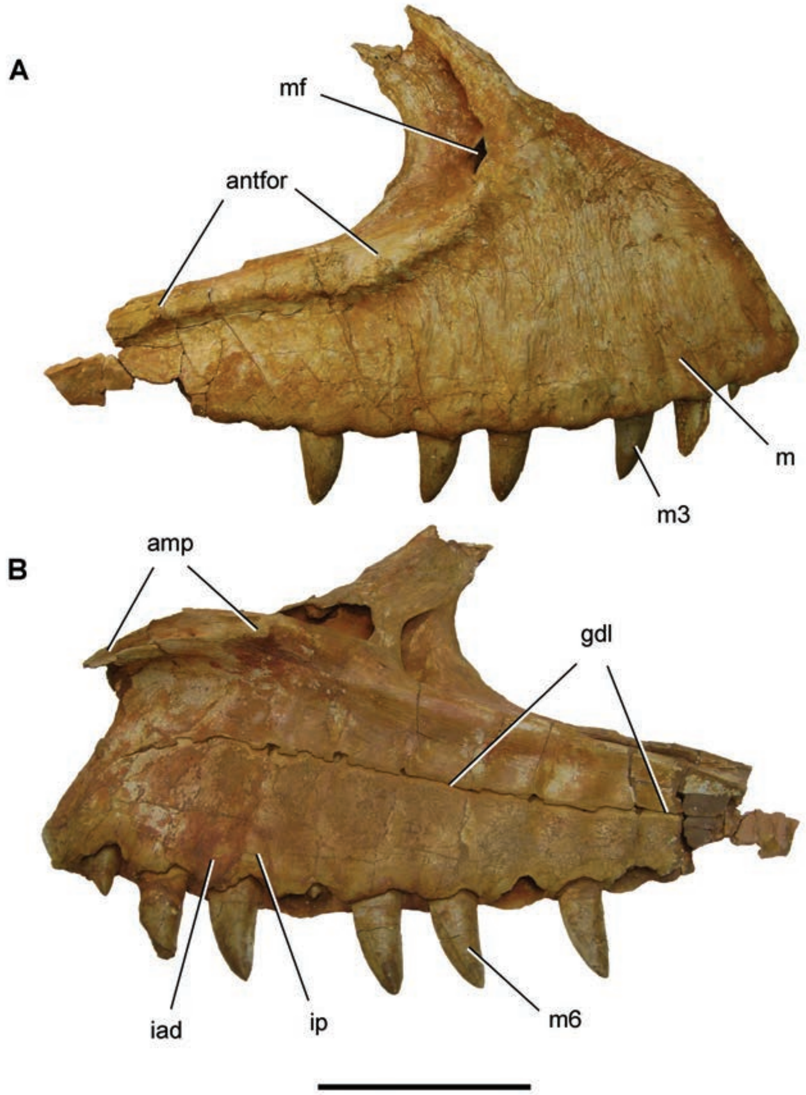

Figure 133. Right maxilla of *Carcharodontosaurus saharicus*. UCRC PV12 in (**A**) lateral and (**B**) medial view. (See also Brusatte and Sereno 2007). Scale bar equals 20 cm. Abbreviations: **amp** anteromedial process **antfor** antorbital fossa ridge **gdl** groove for dental lamina **iad** interalveolar depression **ip** interdental plate **m** maxilla **m3**, **6** maxillary teeth 3, 6 **mf** maxillary fenestra.

rim of the entire margin of the antorbital fossa is everted and rounded; and the external surface of the maxilla has linear rugosities that arc ventrally from the antorbital margin (Stromer 1931: pl. 1, fig. 6). The nasal (Fig. 134A-C) also matches Stromer's specimen closely (Stomer 1931: pl. 1, fig. 7) and differs from *Mapusaurus* (Coria and Currie 2006: fig. 3). It is gently waisted at midlength in lateral view, has an extensive narial fossa anteriorly, and distributed rugosities posteriorly.

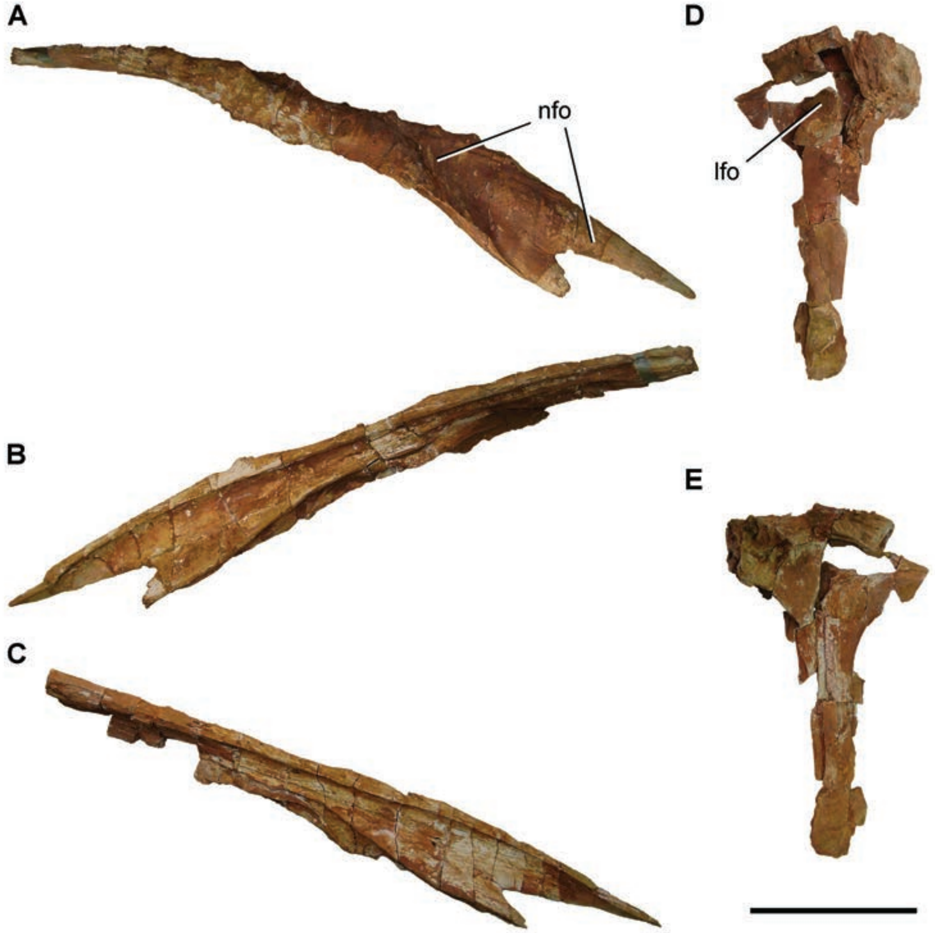

Figure 134. Nasal and lacrimal of *Carcharodontosaurus saharicus* (UCRC PV12). Right nasal in (**A**) lateral and (**B**) medial views **C** Left nasal in medial view. Left lacrimal in (**D**) lateral and (**E**) medial view. Scale bar equals 20 cm. Abbreviations: **lfo** lacrimal fossa **nfo** narial fossa.

The very close match between Moroccan and Egyptian cranial bones figured prominently in the decision to designate the Moroccan cranium as the neotype for *Carcharodontosaurus saharicus* (Brusatte and Sereno 2007). Following Article 75.3 (ICZN 2000), the generic name deserved to be sustained given its continued and widespread use after the original material was destroyed; the neotype is distinctive and consistent with the original name-bearing holotype; and the neotype comes from similar nearshore deposits of Cenomanian age at a similar latitude along the northern margin of Africa. The distance between Egyptian and Moroccan specimens is bridged by localities that have yielded similar blade-shaped teeth with marginal enamel wrinkles (Benton et al. 2000), including the original Algerian locality of Timimoun. The distance between the neotype locality and Stromer's site in the Western Desert of Egypt is not sufficient reason to reject neotypical designation (*contra* Evers et al. 2015: 75).

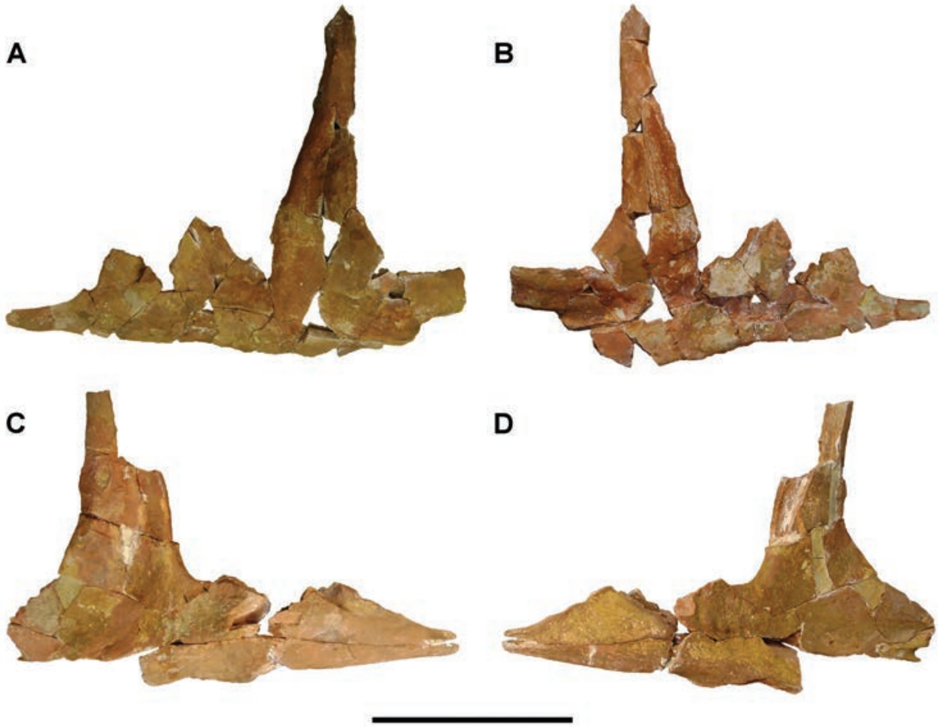

Figure 135. Jugal of *Carcharodontosaurus saharicus* (UCRC PV12). Left jugal in (**A**) lateral and (**B**) medial view. Right jugal in (**C**) lateral and (**D**) medial view. Scale bar equals 20 cm.

Additional cranial material referable to *Carcharodontosaurus saharicus* includes a section of the dentary from a large individual (Fig. 138A-C, F, G) originally described as pertaining to an abelisaurid (Russell 1996). The deep proportions and alveolar morphology of this specimen clearly suggest it belongs to a large, strong-jawed predator with relatively large laterally compressed teeth (Ibrahim et al. 2017). Another fragment of the alveolar margin of the dentary may also pertain to *C. saharicus* (Fig. 138D, E).

Sereno et al. (1996) and Brusatte and Sereno (2007) incorrectly referred stout, broad cervicodorsal centra from Morocco and Niger to *Carcharodontosaurus*. Given the large, heavy skull of advanced carcharodontosaurids such as *Carcharodontosaurus* and *Giganotosaurus*, they had reasoned that the isolated, broad, stoutly constructed vertebrae found in Morocco, Egypt and Niger probably belonged to carcharodontosaurids rather than to spinosaurids or another unknown theropods. At that time, almost nothing was known about the cervicodorsal vertebrae of large-skulled Cenomanian carcharodontosaurids. Likewise, spinosaurid cervicodorsal centra of undoubted association, namely those pertaining to *Baryonyx* Charig and Milner 1997) and *Suchomimus* (Sereno et al. 1998), did not exhibit the broad, kidney-shaped articular faces of these odd vertebrae.

More recently discovered material of both of these theropod groups has settled this question. The stout, broad-faced centra, close in form to those described from Cenomanian-age rocks in Egypt as "*Spinosaurus* B" (Stromer 1931), were found in an

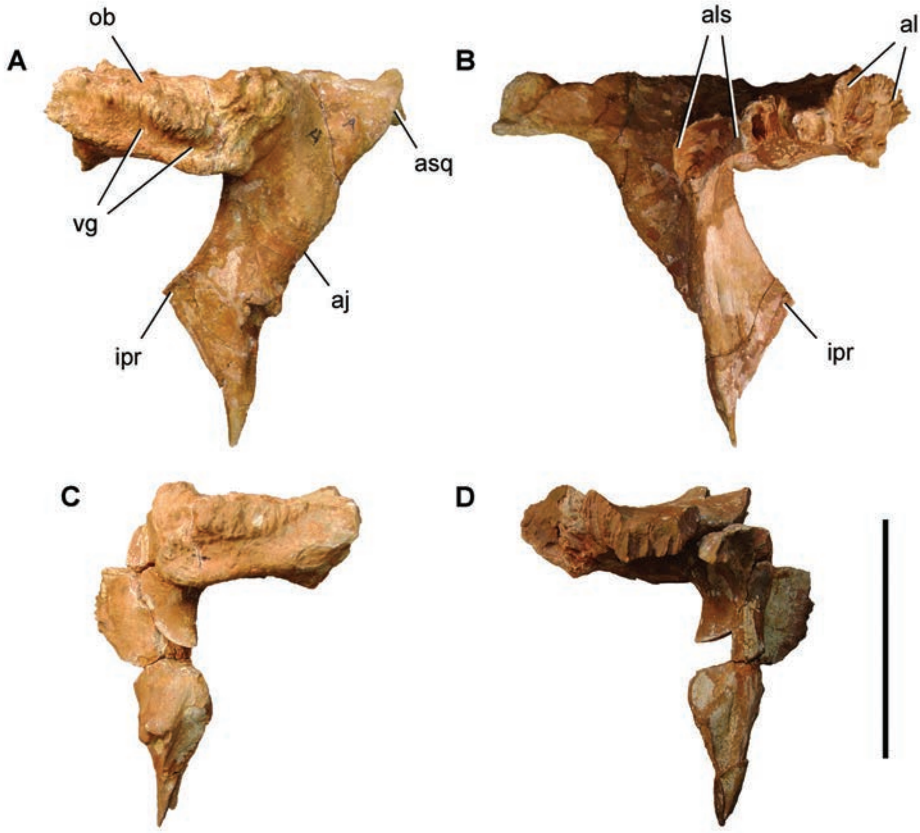

Figure 136. Postorbital of *Carcharodontosaurus saharicus* (UCRC PV12). Left postorbital in (**A**) lateral and (**B**) medial view. Right postorbital in (**C**) lateral and (**D**) medial view. Scale bar equals 20 cm. Abbreviations: **aj** articular surface for the jugal **al** articular surface for the lacrimal **als** articular surface for the laterosphenoid **asq** articular surface for the squamosal **ipr** intraorbital process **ob** orbital brow **vg** vascular groove.

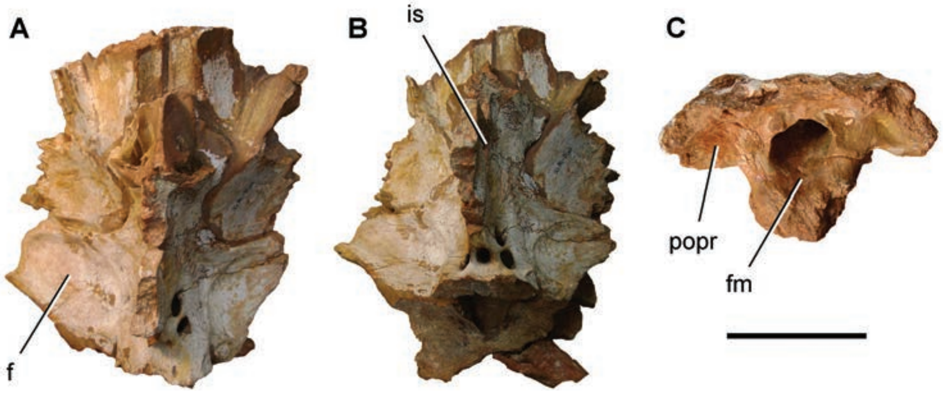

Figure 137. Posterior skull elements of *Carcharodontosaurus saharicus* (UCRC PV12). Braincase in (**A**) ventral, (**B**) anteroventral view and (**C**) posterior view. Scale bar equals 10 cm. Abbreviations: **f** frontal **fm** foramen magnum **is** interorbital septum **popr** paroccipital process.

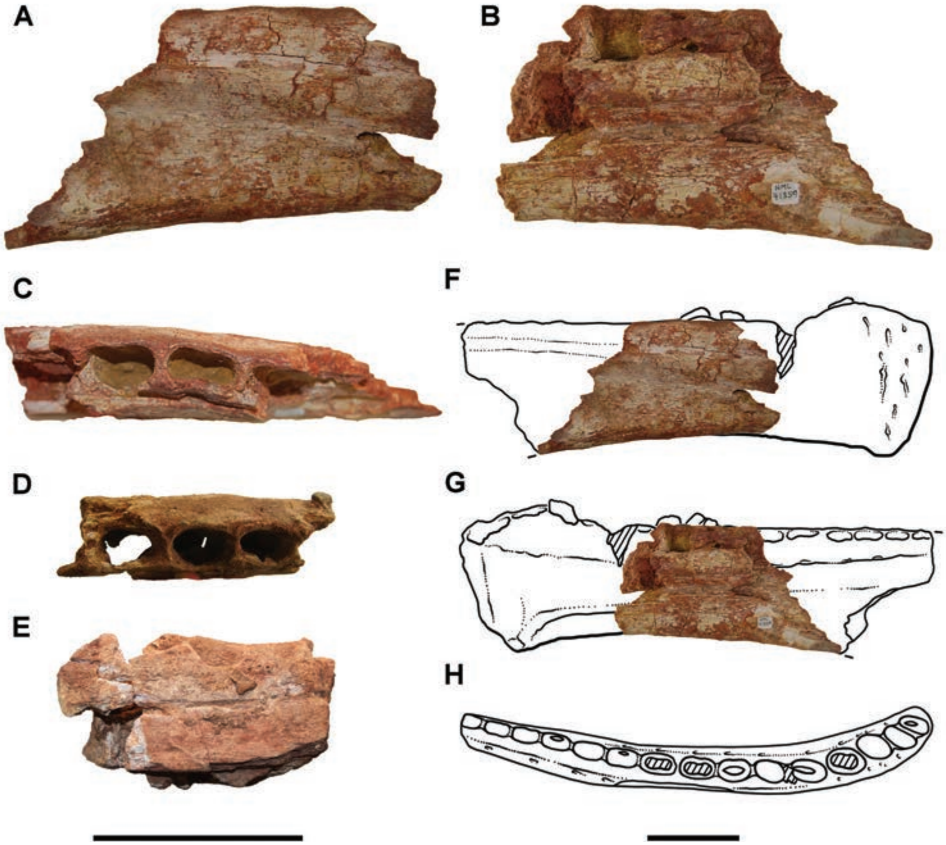

Figure 138. Dentary fragments likely referable to *Carcharodontosaurus saharicus*. NMC 41859 in (**A**) right lateral, (**B**) medial and (**C**) dorsal (occlusal) view. FSAC-KK 02 in (**D**) dorsal (occlusal) and (**E**) medial view. NMC 41859 matched with the dentary of *Giganotosaurus carolinii* (MUCPv-95, redrawn and reversed from Calvo and Coria 1998) in (**F**) right lateral, (**G**) medial and (**H**) dorsal (occlusal) view. Scale bars equal 10 cm in **A-E** and 10 cm in **F-H**. **F-G** adjusted to same size (MUCPv-95 is 610 mm long, Calvo and Coria 1998).

associated skeleton of *Ichthyovenator* (Allain et al. 2012, Allain 2014). We regard vertebrae similar to those of "*Spinosaurus* B" found in the Kem Kem Group in Morocco (described as "*Sigimassasaurus*"; Russell 1996) and in the Eckar Formation in Niger (Brusatte and Sereno 2007) as referrable to *Spinosaurus* (see also discussion above). New carcharodontosaurid material pertaining to the Argentine genera *Mapusaurus* (Coria and Currie 2006 and *Tyrannotitan* (Novas et al. 2005b), on the other hand, show that these large-skulled predators have short cervical centra with subcircular articular faces. Among spinosaurids, in contrast, stout cervicodorsal centra with broad, kidney-shaped articular faces were widespread. Based on the cranial material from Morocco, tentatively referred specimens, and postcranial material of closely related carcharodontosaurids, cranial and skeletal reconstructions (Figs 139, 140) are possible for *Carcharodontosaurus* (Sereno et al. 1996, Ibrahim et al. 2017).

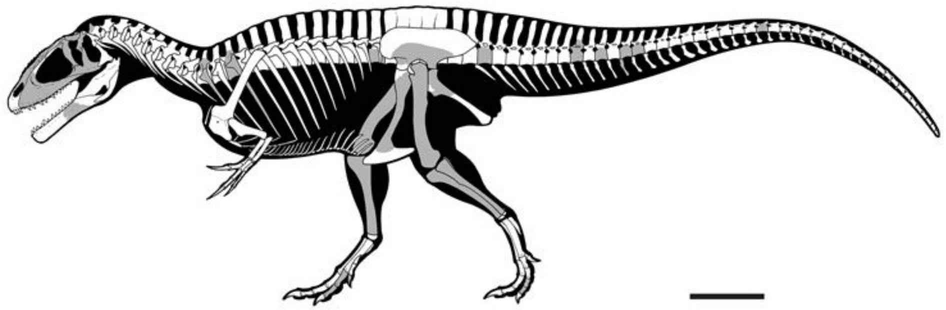

Figure 139. Preliminary skeletal silhouette reconstruction of *Carcharodontosaurus saharicus* with preserved bones (including tentative referrals) shown in gray (after Ibrahim et al. 2017). Scale bar equals 1 m.

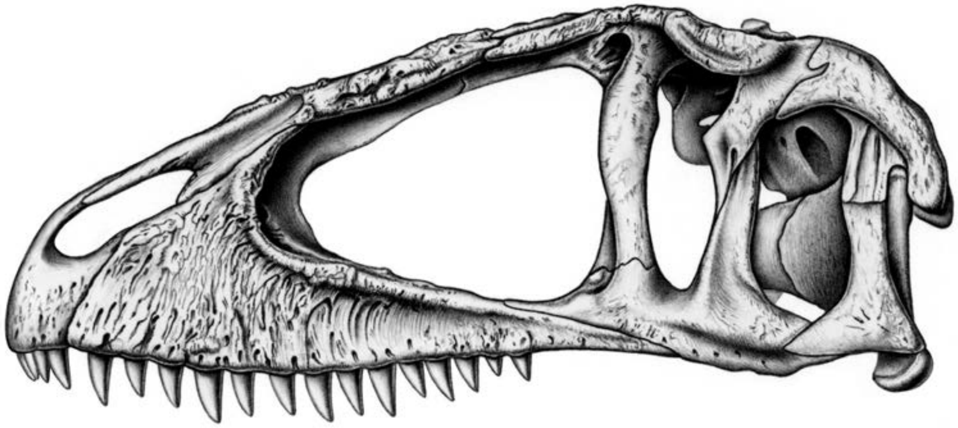

Figure 140. Reconstruction of the cranium of *Carcharodontosaurus saharicus* in left lateral view (premaxilla shortened, after Sereno et al. 1996).

"***Sauroniops***"**.** Recently another carcharodontosaurid taxon, *Sauroniops pachytholus*, was described from the Kem Kem Group based on an isolated commercially collected left frontal (Cau et al. 2012, 2013). This disarticulated bone measures approximately 19 cm in length, with ca. 12 cm exposed on the dorsal skull roof posterior to the nasal suture (Cau et al. 2012). In the neotype skull of *Carcharodontosaurus saharicus* (Sereno et al. 1996: fig. 1B), the length of the frontal on the skull roof is approximately 20 cm, and it is fused to its opposite and to the parietal; figures and measurements given by Stromer (1931: pl. 1, fig. 4) suggest a similar size and state of fusion for the frontals in the original holotype of *C. saharicus*. The disarticulated frontal of *S. pachytholus*, thus, is approximately 60% the size of the frontal in the original holotype and neotype of *C. saharicus*. This size disparity casts in a new light what amount to minor differences between these specimens.

We do not regard the diagnostic features given to distinguish *S. pachytholus* from *C. saharicus* as convincing. Great weight is given to the exact contour of the interdigitating lateral sutural edge of the frontal, an edge that is overlapped in part by adjacent bones (Cau et al. 2013). In addition, a prefrontal articular facet on the frontal in *S. pachytholus* is said to be absent in *C. saharicus*. In the neotype skull of *C. saharicus*, the suture between the lacrimal and prefrontal is visible only in ventral view of the dorsal skull roof (preserved on the right side). It shows that the prefrontal portion of the co-ossified lacrimal-prefrontal articulates with the frontal distal to the location of the "prefrontal facet" in *S. pachytholus*. The exact course of the lacrimal-prefrontal suture with the frontal, in addition, is less step-shaped as a result of overlap along an interdigitating and, in places swollen, suture between these elements. Other immature skull elements of *C. saharicus* found in the Kem Kem Group, such as a postorbital, show less prominent development of brow ornamentation as one would anticipate in the course of growth.

Likewise, we fail to discern other alleged differences, such as "prominent frontal shelves overhanging the supratemporal fossa", which is similar to a feature ("invaginated anteromedial corner of the supratemporal fossa") cited previously in the diagnosis of *C. saharicus* (Brusatte & Sereno, 2007: 904). The anterolateral corner of the supratemporal fossa is said to have a "distinct corner rather than a more gently curved anterolateral margin" in *C. saharicus*. However, only the central portion of the supratemporal fossa is preserved in *S. pachytholus* (Cau et al. 2013: fig. 2CD). Cau et al. (2013) cited more widely spaced nasal articulations in *S. pachytholus*, although *C. saharicus* shows a similar W-shaped nasal-frontal suture common to many theropods. *Sauroniops pachytholus* is said to have as a diagnostic feature an "extensively ossified interorbital septum", despite the fact that the very well ossified interorbital septum in carcharodontosaurids is well known and has yielded very complete endocasts in *Acrocanthosaurus* (Franzosa and Rowe 2005), *Giganotosaurus* (Coria and Currie 2003) and *C. saharicus* (Stromer 1931, Larsson et al. 2000, Larsson 2001). We fail to discern any basic differences to the condition in *C. saharicus* and regard *Sauroniops* and *S. pachytholus* as junior synonyms of *Carcharodontosaurus* and *C. saharicus,* respectively.

Dromaeosauridae Matthew & Brown, 1922. Dromaeosaurid teeth have been reported from several unspecified localities in the Kem Kem region (Amiot et al. 2004). They are recognized by the absence, or smaller size, of serrations on the mesial carina (Fig. 110I, K). Although well documented on Laurasian landmasses and on South America (Unenlagiinae), dromaeosaurids have been positively identified on Africa only from associated pedal bones in Cenomanian-age rocks in Sudan (Rauhut and Werner 1995, Rauhut 1999). Didactyl pes tracks have been reported from possible Middle or early Late Jurassic beds in Niger that may pertain to an early African deinonychosaurian (Mudroch et al. 2011). Variation in crown shape in the small available Kem Kem sample of potential dromaeosaurid teeth allows only the current preliminary assessment that dromaeosaurid theropods were present in the Kem Kem fauna.

Discussion

Stratigraphic resolution

Supergroup recognition for formational trilogy. Choubert (1948) recognized the prominent regional carbonate platform (“Calcaires cénomano-turoniens”) as the uppermost unit of his “trilogie mésocrétacée”, with the underlying two nonmarine units particularly thick and exposed in the Kem Kem region. Although referencing the platform and similar three-part sequences elsewhere in Morocco and beyond, Choubert did not designate type sections or formally name any of these units. The underlying two units eventually were collectively called the “Kem Kem beds” (Sereno et al. 1996). They were deposited on a north-sloping ramp called the “Kem Kem embayment” (Essafraoui et al. 2015), which has been previously cited as the “Taouz Basin” (Cavin et al. 2010) or “Tafilalt Basin” (Wendt 1985, Bockwinkel et al. 2013).

In this study, we recognize Choubert’s “trilogie mésocrétacée” as the Hamadian Supergroup (Table 7). Based on the Arabic word “hamada”, or rocky plateau, the uppermost carbonate platform is often developed as a prominent, barren, plateau covering broad areas of north Africa. The carbonate platform and the pair of softer underlying strata comprise a sustained global, stepwise marine transgression of ca. 10 My duration that began sometime during the Albian ca. 100 Ma and continued through to the Turonian ca. 90 Ma (Ferrandini et al. 1985, Ettachfini and Andreu 2004, Ettachfini et al. 2005, Walker et al. 2018). We have limited our correlations of Hamadian Supergroup rocks to formations within Morocco. Correlation across northern Africa is now possible incorporating new paleontological and chronological data on comparable strata nearby in western Algeria (Benyoucef et al. 2012, 2014) and farther to the east in Tunisia (Fanti et al. 2012), Libya (Rage and Cappetta 2002) and Egypt (Catuneanu et al. 2006).

Group recognition for formational duo. We recognize as the Kem Kem Group the first two non-marine units of the Hamadian Supergroup, which extends across much of central and eastern Morocco north and south of the Pre-African Trough (Table 7). In the Kem Kem region, the pair of non-marine units are particularly fossiliferous and characterized by a number of distinctive sedimentological and ichnological features. We recognize these successive, comformable formations as the Gara Sbaa and Douira formations, with stratotypes at fossiliferous and well exposed nominotypical localities in the Kem Kem region.

Similar pairs of strata, the lower coarser-grained, are present in the Tinghir region to the north near Aoufous and northeast near Goulmima, although they are not connected by continuous outcrop to the Kem Kem region. These deposits, which are generally thinner and much less fossiliferous, appear to have been deposited in depressions separate from the Kem Kem embayment. We do not agree with applying formational names from the Tinghir region (e.g., Ifezouane and Aoufous formations) to the distinctive, mapable strata of the Kem Kem embayment (*contra* Cavin et al. 2010). Rather, we recognize the continuing need to formalize these comparable Tinghir region formations (Table 7) by way of additional study and description of stratotype sections.

Biostratigraphic resolution

In his pioneering geological work on Hammadian supergroup rocks in Morocco, Lavocat sometimes referred to beds by the most common fossil, such as the land snail *Clavator* or the sawfish *Onchopristis*. The "grès à *Onchopristis*" (*Onchopristis* sandstone), for example, corresponds with the Gara Sbaa Formation of this paper, as Lavocat indicated graphically (1954b: pl. IV, fig. 11). It would be incorrect, however, to infer a precise biostratigraphic zonation from Lavocat's informal stratigraphic nomenclature. The extinct genera Lavocat cited for convenience in affiliation with particular strata are not necessarily limited to those beds.

Fossils collected from the Kem Kem Group, for the most part, are derived from two sources: surface-collected specimens from known or unknown localities that have weathered from their original point of burial and commercially collected specimens from channel sandstones in the Gara Sbaa Formation lacking precise associated locality or stratigraphic data. Most known Kem Kem fossils, thus, are not associated with reliable stratigraphic data. For this reason, little has been said in the literature about biostratigraphic zonation within the Kem Kem Group.

Only a few vertebrate specimens in the Kem Kem Group have been collected *in situ* by professionals from a precise geographic locality and positioned by measurements on a local stratigraphic section. We have cited most of these specimens in this paper. Some of these specimens are unique, such as the partial articulated skeleton of the theropod *Deltadromeus* (Sereno et al. 1996), which was discovered in the upper one-third of the Gara Sbaa Formation. As a singular find, it is known from a point in time rather than a time interval or range. The pond locality Oum Tkout at the base of the Douira Formation, likewise, preserves hard and soft-bodied organisms, most of which are not found elsewhere in either formation of the Kem Kem Group (Cavin et al. 2010: table 1). Singular finds contribute significantly to the diversity of the Kem Kem fauna but offer little insight into biostratigraphic range. The major question remains: Is the Kem Kem vertebrate assemblage, in general, a contemporaneous and long-lived fauna (several million years) that was established in multiple local environments — or is it a composite noncontemporaneous fauna with major components limited to one or the other of the two formations in the Kem Kem Group?

To evaluate range, we are limited to considering some of the more common fossil vertebrates in these formations, which comprise teeth and generically diagnostic bone pieces. Only these are common enough to provide a sense of range. They have been found in both formations (as surface-collected and *in situ* specimens): the elasmobranch *Onchopristis*, dipnoan *Ceratodus*, theropod dinosaurs *Spinosaurus* and *Carcharodontosaurus*, and bones referable to possibly the same or similar rebbachisaurid and titanosaurid sauropods. This evidence, although meager, suggests that at least some aquatic and terrestrial vertebrate genera ranged across both formations.

Cavin et al. (2010: 394) termed the entire faunal list from the Kem Kem Group the "Kem Kem compound assemblage". That terminology, however, implies that there is proof that the assemblage is a time-averaged mixture of successive or discrete faunas.

Our field work has not generated any direct evidence in support of this hypothesis. We agree that the diverse Kem Kem flora and fauna so far discovered (Table 8) is an "assemblage", rather than a "biota", because that diversity is certain to have been recorded from separate and sometimes ephemeral or migrating habitats. The pond locality Oum Tkout is the best-known example of a local habitat that records many species only known from this site. Kem Kem Group formations, like many Mesozoic-age terrestrial strata, are characterized by poorly constrained vertebrate biostratigraphic ranges. We simply do not have the necessary stratigraphic control to know more than we stated above, that some common aquatic and terrestrial vertebrates appear to be present in both formations. Thus we refer to the vertebrate fauna as the "Kem Kem vertebrate assemblage", which we have updated and compiled in Table 8.

Taxonomic resolution

The Kem Kem vertebrate assemblage (Table 8) is certain to be revised based on the recovery of additional diagnostic fossil remains and additional study of existing collections. The fragmentary nature of the fossil evidence prevents resolution at the generic and species level for many taxa, their identification remaining indeterminate at the level of family. Is this a relatively complete snapshot of Kem Kem vertebrate diversity? How might taxonomic or preservation biases have affected this summary?

Two potential pitfalls in the evaluation of fragmentary remains have opposing effects on the estimation of taxonomic diversity. Over-splitting taxa that represent a single species exaggerates diversity, whereas under-splitting (lumping) taxa that are actually distinct underestimates diversity.

Over-splitting, perhaps the more common of the two, occurs when taxa are erected on fragmentary remains that do not overlap, when taxa are differentiated on features that appear during growth and maturation, or when few distinguishing features prove not to be diagnostic. Although estimates vary among vertebrate and nonvertebrate groups and living versus extinct taxa, at least 30% and possibly as much as 50% of non-avian dinosaurian taxa have been relegated to taxonomic error of one sort or another (Benton 2008). Over-splitting, in our opinion, has been a significant factor in the taxonomic analysis of Kem Kem remains, given the fragmentary and isolated nature of most fossil material. Theropod dinosaurs, the focus of considerable attention within the vertebrate fauna, are a case in point. Consideration must be given not only to their taxonomic differentiation among Kem Kem fossils, but also to teeth and bones from distant locales along the northern coast of Africa. In our systematic review of the theropod dinosaurs in the Kem Kem Group, for example, we argue that current fossil evidence supports the existence of only one genus and species, rather than two or more genera and species of abelisaurids, carcharodontosaurids, and spinosaurids. We regard the single carcharodontosaurid (*Carcharodontosaurus saharicus*) and spinosaurid (*Spinosaurus aegytiacus*) as conspecific with their counterparts in the likely contemporaneous Bahariya Formation of western Egypt. In this case, alternative interpretations have

split both of these genera, doubling the number of large-bodied predators recorded in the Kem Kem Group.

Under-splitting occurs when taxonomists lump together non-overlapping remains, assuming that they pertain to a single, rather than closely related, second species or genus. Only overlapping material can directly highlight differences; novel species or genera may remain unrecognized, when material that does not overlap is referred to the same taxon. This is especially true regarding the fragmentary, and often transported, fossils in the Kem Kem Group. Reference to a single taxon, however, may constitute a prudent decision, when no direct comparative evidence exists to justify the presence of a second taxon.

Archosaurian reptiles, the focus of much ongoing research, comprise most of the moderate to large-sized terrestrial vertebrates in the Kem Kem Group. A few of these genera will be discarded for various reasons in future taxonomic work. Other existing genera, on the other hand, are likely to be split, so the current tally is probably a reasonably accurate sum of current knowledge of Kem Kem Group taxonomic diversity (Table 8).

Trends in research activity and collection efforts allow a preliminary assessment of our current state of knowledge. After a period of relatively little research in the region, renewed interest began with the 1995 American-Moroccan survey expedition and subsequent publications (Sereno et al. 1996, Currie 1996). Field and collections research were initiated shortly thereafter by several European institutions. At the same time, commercial collecting by locals dramatically expanded in the 1980s, and publications began to appear based on the purchase of individual specimens and diverse vertebrate collections (e.g., Russell 1996). Moroccan collections, in addition, were established after 2000 in Marrakech (Musée d'histoire naturelle de Marrakech) and Casablanca (Faculté des Sciences Aïn Chock). Now there exists a diverse community of professional researchers and students working on Kem Kem fossils compared to the single-handed efforts of Lavocat and Tabaste in the 1950s and 1960s.

Given the continued input of new specimens and the continuing expansion of paleontological research, we predict that diversity in the Kem Kem Group will increase substantially in the coming decades. Based on our review of existing collections, this increase will include scores of taxa from the pond locality Oum Tkout including non-vertebrates, such as plants, insects, and ostracods, as well as an array of actinopterygian fish. We also anticipate a continuing trickle of new terrestrial vertebrates that will be named on better preserved specimens that are diagnostic at present only at the familial level, including turtles and various kinds of archosaurs. As nearly half of the reptilian families listed are indeterminate (Table 8), better preserved specimens will offer future opportunity to recognize new reptilian genera.

Other well-known Cenomanian vertebrate assemblages, such as those recorded from localities in the Bahariya Formation in the Western Desert of Egypt (Rauhut 1995, Ijouiher 2016), the Algora locality in Spain (Torices et al. 2012), or the Aquitaine Basin in southwest France (Vullo et al. 2005, 2007, Vullo and Néraudeau 2008) have yielded fewer vertebrate genera and families than that for the Kem Kem Group

(Table 8). We believe there are two principal factors generating this differential. The first is the much larger area of exposed fossiliferous outcrop for the Kem Kem Group, which stretches in pockets along more than 200 km of cliffline. The second factor is the diversity of paleoenvironments represented in the Kem Kem Group, which includes the prograding deltaic sediments of the Gara Sbaa Formation as well as the coastal flats and quiet pond paleoenvironments of the Douira Formation.

In summary, the Kem Kem assemblage of nonvertebrates and vertebrates is likely to continue to show dramatic increase in diversity in the coming decades. Nonetheless, the array of taxa currently known, which extends from plants across a range of aquatic and terrestrial vertebrates, is sufficiently mature to allow a summary of the vertebrate assemblage and a discussion of its paleoecological context.

Kem Kem assemblage

Assemblage character

Predominance of aquatic predators. The Kem Kem assemblage is dominated by aquatic and subaquatic nonvertebrates and vertebrates, nearly all of which are predators. Approximately 85% of the vertebrate taxa in the assemblage are aquatic or subaquatic (Table 8). Most of the described vertebrates, with the exception of some of the pterosaurs and dinosaurs, lived exclusively or predominantly within an aquatic setting, which would include pond, river, delta, and nearshore habitats. Most of the taxa in the assemblage, thus, are utilizing aquatic food resources, which like modern marine food webs are often predator-dominated (e.g., Friedlander et al. 2012).

Aquatic habitats, which include pond, river, delta and nearshore settings, supported a diverse array of elasmobranchs, actinopterygians and basal sarcopterygians in marine, brackish and freshwater settings. Currently numbering approximately 40 distinct species (Table 8) and likely to substantially increase in coming decades, these aquatic vertebrates provided the primary resource for predators in the Kem Kem assemblage. Many of these aquatic species are piscivorous, which by their dentition and/or skull form is also the case for several pleurodiran turtles, several long-snouted or flat-skulled crocodyliforms, ornithocheirid pterosaurs, and the theropod dinosaur *Spinosaurus*. The ramp of the Kem Kem embayment was dissected by deltaic waterways that provided ample habitat for a rich aquatic protein source for the food web.

The overabundance of predatory versus herbivorous dinosaurs was first noticed by Stromer (1936) in the Bahariya Formation of Egypt and has been described as “Stromer’s riddle” (McGowan and Dyke 2009). Many later authors underscored this imbalance (Sereno et al. 1996, Russell 1996, Russell and Paesler 2003, Läng et al. 2013), some proposing that it may have arisen as an artifact of collecting or observational bias (McGowan and Dyke 2009). In Morocco the overabundance is recorded in the numbers of specimens and footprints as well as in taxonomic diversity. The predominance of predatory dinosaurs in bone, tooth and footprint records has been confirmed by our field work in the Kem Kem Group and by sampling at other locali-

ties in northern Africa (e.g., western Algeria, Benyoucef et al. 2015). Thus, the overabundance and diversity of theropods is not an artifact of collecting or observational bias (*contra* McGowan and Dyke 2009).

Neither can the overabundance of predatory dinosaur fossils have been generated by preservation factors or selective long-distance transport. No one has described plausible taphonomic biases that would favor the preservation of non-avian theropod tooth, bone and footprints over herbivorous dinosaurs or crocodilians.

Likewise, the evidence for long-distance fluvial transport of vertebrate teeth or bone from one habitat or region to another has come under recent reevaluation. Decades of actual studies have failed to substantiate long-distance fluvial transport of vertebrate fossils as a common phenomenon. The main supporting evidence, bone and tooth abrasion, has been shown to correlate poorly with distance traveled. Long-distance fluvial transport now is regarded by some as only a minor factor in biasing assemblage composition (Behrensmeyer and Rogers 2017). Time-averaging may play a larger role in mixing assemblages of species that did not cohabit. Yet, in the Kem Kem Group, we have very little evidence for any stratigraphic differentiation of the vertebrate fauna.

Thus, the taxonomic and numerical predominance of predatory dinosaurs, which has been termed an "unbalanced food web" (Läng et al. 2013), appears to be a real signal. These large-bodied theropods are supported primarily or secondarily by the abundance and availability of aquatic protein resources (Amiot et al. 2010, Hassler et al. 2018). The dissected deltaic plain and nearshore environments may have enhanced aquatic resources while limiting, or rendering patchy areas of available vegetation for large-bodied dinosaurian herbivores.

Diversity of crocodyliform and dinosaurian predators. Both crocodyliform and dinosaurian predators exhibit unusually diverse cranial form indicative of diverse feeding strategies. Crocodyliforms include an unnamed large-bodied taxon with recurved, transversely compressed teeth presumably preying primarily upon terrestrial animals (Larsson et al. 2015). Three of the four large-bodied theropods in the fauna are known from cranial material and vary markedly in skull and dental form, ranging from scavenging with rasping dentition (*Rugops*-like abelisaurid), primarily piscivorous with a puncturing and ensnaring dentition (*Spinosaurus*), and macropredaceous with sabre-shaped teeth (*Carcharodontosaurus*) (Figs 112, 119, 133). The piscivorous habit of spinosaurs has been supported by oxygen isotopic values from bone apatite (Amiot et al. 2010). Calcium isotopic values from tooth enamel extend these results, supporting resource partitioning among large theropods in both Kem Kem and Gadoufaoua faunas (Hassler et al. 2018).

This triumvirate of large predators (abelisaurid, spinosaurid, carcharodontosaurid) has been recorded at Cenomanian localities across northern Africa with precursors in the Aptian-Albian of both South America and Africa (Sereno and Brusatte 2008, Benyoucef et al. 2015). In the Kem Kem Group and Bahariya Formation of Egypt, this trio of large predators is joined by a fourth, *Deltadromeus* (Sereno et al. 1996, Fig. 118), a gracile fleet-footed form with elongate forelimbs. As no part of the skull was preserved, its trophic preferences remain unknown.

Absence of Aves and Mammalia. The Kem Kem assemblage has yet to yield diagnostic remains of a bird or mammal. Riff et al. (2002) described a small isolated vertebra from the Kem Kem Group as avian solely on the basis of its capacious neural canal, drawing comparisons to other Gondwanan taxa now regarded as non-avian (alvarezsaurids, unenlagines). Cavin et al. (2010), probably on that basis, added indeterminate avians to their faunal list. Definitive and diagnostic body fossil evidence for either of these clades has yet to be unequivocally demonstrated.

Avians and eutherian mammals usually are represented in Cretaceous vertebrate faunas as diverse as that from the Kem Kem Group. The absence of any body fossils of these two clades is extraordinary for five reasons. First, eutherian mammals are present in earlier deposits on Africa including localities in Morocco (e.g., Sigogneau-Russell 1995, Heinrich 1998, Lasseron et al. 2019). Second, scores of fragile pterosaur bones are preserved in both formations of the Kem Kem Group, many of these were as fragile as avian remains. Third, the nearshore environments and abundant insects and fish of all sizes would have offered ample resources for avifauna. Fourth, extensive screen-washing of Kem Kem fluvial sediments has yielded thousands of minute fish bones and teeth as well as the small, cusped crowns of notosuchian crocodyliforms (Larsson and Sidor 1999) but no avian bones or a single crown pertaining to a eutherian mammal. Finally, the tranquil pond deposit at Oum Tkout presents an excellent opportunity for preservation of small, fragile remains. Plant fronds, insects, soft-bodied decapods and the vertebrae of small amphibians are preserved, but as yet no remains of a bird or mammal have been discovered. In Tunisia trace evidence has come to light recently for both groups from the Cenomanian-age Zebbag Formation (Contessi and Fanti 2012, Contessi 2013).

A variety of small, multicuspid notosuchian crocodilians are present in the Kem Kem assemblage, some of which may have been insectivorous or herbivorous (Larsson and Sidor 1999). Other notosuchians with similar trophic modes include *Araripesuchus rattoides* (Sereno and Larsson 2009, Fig. 74A-D) and *Lavocatchampsa* (Martin and de Lapparant de Broin 2016), and these crocodyliforms may have displaced mammals as the dominant small-bodied herbivores.

Assemblage summary. Geologic, taphonomic and collection biases influence the fossil record of taxonomic diversity (Brett and Baird 1986, Benton 1998, Kidwell and Holland 2002), all of which have played a role in shaping the Kem Kem assemblage. The vast majority of Kem Kem Group sediments, for example, do not have the preservation potential for the herbaceous fronds, leaves, and soft-bodied nonvertebrates that are abundant in the single pond locality Oum Tkout in the Douira Formation. We summarize here what we currently know about the flora and fauna of the Kem Kem assemblage as a whole.

Plants are preserved most commonly as woody stem or trunk fragments. Complete leaves and fronds of herbaceous and woody plants including ferns, ginkgoes, gymnosperms and angiosperms have been recovered from the pond locality Oum Tkout, many of which remain to be described. *Nonvertebrates* include shelly and soft-bodied mollusks, crustaceans including the prawn *Cretapenaeus* (Garassino et al. 2006, Fig.

52) and an undescribed macruran decapod (Fig. 40C), and insects including larval and mature mayflies and dragonflies (Fig. 53). Unionoid clams occur rarely as steinkerns (internal casts), their valves lost to dissolution.

The hooked rostral teeth (Fig. 54I) and discoid calcified centra (Fig. 56) of the sclerorhynchid batoid *Onchopristis* are the most common vertebrate fossils in Kem Kem Group sediments. In rare instances, sections of calcified rostra are preserved with fused teeth projecting from their margins (Fig. 54A, B), and a series of centra are preserved in articulation (Fig. 56). *Onchopristis* is one of more than a dozen elasmobranch genera that inhabited coastal and inland brackish waters on the Kem Kem embayment. An array of hybodontoid and neoselachian sharks were present (Table 8), the presence of several confirming a Cenomanian age for the middle section of the Kem Kem Group (Sereno et al. 1996).

Actinopterygian fish are particularly diverse and likely to add to their diversity in the future. They include more than a dozen genera of basal forms including cladistians, lepisosteiforms, and amiiformes. Cladistians include whole body specimens of the small-bodied *Serenoichthys* (Fig. 57) from the Oum Tkout pond locality as well as robust jaw bones and scales of the several-meter long cladistian *Bawitius* (Cavin et al. 2010, Meunier et al. 2016, Figs 58, 59). Lepisosteid and seminotiform fish also range in size from small to large, with the largest scales measuring more than 5 cm in length (Fig. 60). Amiiforms are represented by jaw bones (Fig. 61A-D) and a partial skull referred to *Calamopleurus* (Forey and Grande 1998). More advanced teleost fish, which currently include eight genera across a range of subgroups and body forms (Table 8), are likely to double in number in the coming years from specimens collected from the freshwater pond locality Oum Tkout.

Sarcopterygian fish are common in both formations of the Kem Kem Group, including the large coelacanth *Axelrodichthys* recently described from a 30 cm skull (Figs 65, 66). Toothplates of varying size and ornamentation provide evidence of lungfish (Fig. 67), which are tentatively referred to at least two genera, *Ceratodus* and the smaller-bodied genus *Arganodus*.

Salamander vertebrae assigned to *Kababisha* and a second unnamed genus were recovered from screenwashed sediment from microsites in the Douira Formation. At least two *frogs* are present in the Kem Kem assemblage, one a non-pipid anuran known from a partial braincase, jaw fragments and vertebrae (Rage and Dutheil 2008) and the other pertaining to a new pipid *Oumtkoutia* from the pond locality (Fig. 68).

Turtles are common fossils in the Kem Kem Group, although most specimens are isolated shell fragments (Gmira 1995, Figs 69–71). A thin flat, pitted plastral element (Fig. 70A, B) suggests the presence of an araripemydid pleurodire in the Kem Kem assemblage, although little else can be said until more complete specimens are found. Four additional pleurodires including *Dirqadim*, *Hamadachelys* and two species of *Galianemys,* are known from more complete material including several skulls (Fig. 73) and a partial shell (Fig. 72).

Snakes and *iguanian* and *borioteiioid squamates* are known from isolated jaw framents and vertebrae in both formations and at the pond site Oum Tkout. There are at least three

basal snakes including *Lapparentophis*, *Simoliophis*, and one assigned to a new genus *Norisophis* (Fig. 68). Dentaries with crowns have been described as a new iguanian *Jeddaherdan* (Apesteguía et al. 2016a) and a new borioteiioid *Bicuspidon* (Vullo and Rage 2018).

Crocodyliforms are a well-represented and diverse group in the Kem Kem assemblage currently known from abundant teeth, osteoderms, braincases, and skull bones, and partial and complete skulls. Several skulls rank among the most complete for any Kem Kem vertebrate (e.g., Fig. 79). The material is presently assigned to six genera (*Hamadasuchus*, *Araripesuchus*, *Lavocatchampsa*, *Laganosuchus*, *Aegisuchus*, *Elosuchus*), which is certain to be revised and expanded in the future. These crocodyliforms range in size and trophic adaptations from small-bodied, multipcuspid herbivores (*Lavocatchampsa*, Martin and de Lapparent de Broin 2016) to large-bodied, long-snouted piscivores (*Elosuchus*, de Lapparent de Broin 2002, Young et al. 2017, Meunier and Larsson 2017). *Laganosuchus maghrebiensis* may have been a sit-and-wait predator, snapping together its dorsoventrally flattened, U-shaped jaws (Sereno and Larsson 2009, Fig. 83). A large unnamed peirosauird is known from the Kem Kem Group and the Echkar Formation of Niger (Larsson et al. 2015).

Pterosaurs of large wingspan (4–6 m) and disparate trophic adaptations are known from abundant elongate, curved teeth, jaw ends (rostra and mandibulae), vertebrae and limb bones. Three families of pterosaurs have been found: long-snouted, dentate ornithocheirids (Figs 94, 95), long-snouted, edentate neoazhdarchids (Figs 98–101), and a deep-jawed, toothless tapejarid (Fig. 97). The ornithocheirids *Siroccopteryx* and *Coloborhynchus* have elongate, interdigitating teeth for ensnaring fish during aerial or shallow-water dip-feeding (Mader and Kellner 1997, Wellnhofer and Buffetaut 1999). Four morphotypes among isolated teeth have been recongised, some slender and others more robust and broad-based. All may pertain to ornithocheirid pterosaurs. The azhdarchids *Alanqa* and *Xericeps* are known from pointed, edentulous rostral and mandibular fragments (Ibrahim et al. 2010, Averianov 2014, Martill et al. 2018) and may have been aerial dip-feeding predators or terrestrial foragers. The toothless tapejarid dentary was likely short in length with a deep submandibular flange (Fig. 97). Tapejarid trophic strategy remains unknown.

Dinosaurian fossils and footprints are common in Kem Kem Group sediments, the latter from several levels in the upper one-half of the Douira Formation (Sereno et al. 1996). The rarest of all dinosaur remains are the teeth and footprints of ornithischians, each known literally from individual specimens. Isolated sauropod teeth, partial vertebrae, postcranial fragments and footprints are rare, although more common than their ornithischian counterparts. Finally, isolated non-avian theropod teeth are the most common relatively well preserved dinosaurian remains followed by partial vertebrae, long bone pieces, unguals, and, more rarely, partial skull bones. Only four dinosaurs (*Rebbachisaurus*, *Spinosaurus*, *Carcharodontosaurus*, *Deltadromeus*) are known from single associated partial skulls or skeletons (Lavocat 1951, Wilson and Allain 2015, Ibrahim et al. 2014b, Sereno et al. 1996). Non-avian theropods, likewise, are by far the most common trackmaker among preserved footprints in the Douira Formation (Ibrahim et al. 2014a).

Ornithischians are extremely rare in the Kem Kem Group. A single isolated tooth and footprint form the Douira Formation provide the only evidence thus far for these herbivores, which were represented by an array of small- and large-bodied genera in the Aptian-Albian of Niger (Gadoufaoua) perhaps ~15 million years prior. The single denticulate, unworn crown, which was recovered from Oum Tkout, comes from a small-bodied, dentally unspecialized ornithischian that may pertain to a thyreophoran (Fig. 103). A single large clover-shaped, three-toed footprint measures 0.51 m in length and records the presence of a large iguanodontian in the Douira Formation (Sereno et al. 1996, Ibrahim et al. 2014a).

Sauropods include a rebbachisaurid diplodocoid (Lavocat 1954b, Russell 1996) and a titanosaurian (Russell 1996, Ibrahim et al. 2016). *Rebbachisaurus garasbae* (Fig. 104), the best known and only named sauropod taxon, is based on asssociated postcranial material from the upper part of the Gara Sbaa Formation (Lavocat 1954a, 1954b, 1955, Wilson and Allain 2015). Slender subcylindrical teeth found in isolation may pertain to *R. garasbae* (Fig. 107), although this is less certain than the more common vertebral fragments, characterized by thin laminae joining pneumatized centra and neural arches (Fig. 106). The narrow-crowned tooth has enamel on both sides, unlike the more derived one-sided enameled crowns in the older rebbachisaurid *Nigersaurus* (Sereno and Wilson 2005).

Isolated teeth (Fig. 108) and several postcranial bones (Figs 109) pertain to one or more titanosaurian sauropods (Ibrahim et al. 2016, Holwerda et al. 2018). The isolated crowns are slightly swollen and exhibit a single high-angle wear facet (Fig. 108A, K, R-T, W). A large partial humerus, tarsal and isolated caudal vertebrae pertain to a titanosaurian sauropod (Russell 1996, Mannion and Barrett 2013, Ibrahim et al. 2016) with body size comparable to the likely contemporary Egyptian titanosaurian *Paralititan stromeri* (Smith et al. 2001). The proximal end of an ulna, which has a proximal width of 0.51 m (Fig. 109), and the partial humerus (Ibrahim et al. 2016) document the presence of very large-bodied titanosaurians in the Kem Kem assemblage.

Non-avian theropods are known most commonly from isolated remains. Some of these specimens are complete enough to evaluate at a familial taxonomic level. Isolated theropod teeth and unguals are sufficiently abundant that some can be sorted into distinct tooth morphotypes, each allied with a particular non-avian theropod clade (Abelisauroidea, Spinosauridae, Carcharodontosauridae, Dromaeosauridae). Some isolated non-avian theropod unguals, likewise, can be divided into those from the manus and pes. Manual unguals, which tend to diverge more strongly in form than pedal unguals, are divisable into three morphotypes, each allied with particular non-avian theropod clades (Spinosauridae, Carcharodontosauridae, Dromaeosauridae).

Non-avian theropods identified at a generic or species level include an abelisaurid close to the genus *Rugops* from Niger (Russell 1996, Mahler 2005, D'Orazi Porchetti et al. 2011), the spinosaurid *Spinosaurus aegyptiacus* (Buffetaut 1989, 1992, Russell 1996, Milner 2003, Dal Sasso et al. 2005, Ibrahim et al. 2014b), the carcharodontosaurid *Carcharodontosaurus saharicus* (Sereno et al. 1996, Russell 1996), and the probable basal coelurosaurian *Deltadromeus agilis* (Sereno et al. 1996). We do not believe

there is solid evidence in favor of a second larger abelisaurid (unnamed, Chiarenza and Cau 2016), a distinctive species (*S. maroccanus*, Russell 1996) or second heavier-bodied spinosaurid (*Sigilmassasaurus brevicollis*, Evers et al. 2015), or a second smaller carcharodontosaurid (*Sauroniops*, Cau et al. 2012, 2013).

Regarding abelisauroids, we support the interpretation of the described maxillae and several other bones from the Kem Kem Group as likely pertaining to a single mid- to large-sized abelisaurid close to the Nigerien genus *Rugops* and another maxilla of similar age from Argentina (Lamanna et al. 2002). Additional abelisaurid bones have been described recently, the affinity of which remains uncertain (Smyth et al. 2020). Noasaurids also appear to be present based on the presence of an isolated cervical vertebra (Smyth et al. 2020).

Regarding spinosaurids, we support the designation of a new partial skeleton (FSAC-KK 11888) as a neotype on grounds of convincing overlap with Stromer's now destroyed Egyptian holotype (Stromer 1915, Ibrahim et al. 2014b). That specimen, for the first time, preserves portions of the axial column that help bridge between Stromer's holotype and the isolated stout vertebrae eventually identified as "*Sigilmassasaurus brevicollis*". Thus, we argue that the material represents a single taxon rather than evidence of two large-bodied spinosaurids.

Regarding carcharodontosaurids, we review and support the evidence used to designate a well-preserved skull (UCRC PV12) as the neotype of *Carcharodontosaurus saharicus* in place of Stromer's now destroyed Egyptian holotype (Stromer 1915, Brusatte and Sereno 2007). We provide comparative measurements to show that the cranial piece recently described as a second carcharodontosaurid, "*Sauroniops pachytholus*" (Cau et al. 2012, 2013), is only 60% the size of the neotype and exhibits the more subdued ornamentation that characterizes immature carcharodontosaurid cranial bones in the Kem Kem Group. As a result, we do not support its generic or specific distinction.

In sum, we support the presence in the Kem Kem assemblage of four well established large-bodied theropods, rather than seven, namely, an unnamed abelisaurid, *Spinosaurus aegyptiacus*, *Carcharodontosaurus saharicus* and *Deltadromeus agilis* (Table 8).

Comparable African assemblages

Some of the taxa listed in the Kem Kem vertebrate assemblage (Table 8) are also present in contemporaneous (late Albian to Cenomanian age) localities to the east across north Africa, from Algeria to Tunisia, Libya and eventually to the Bahariya Formation of Egypt (Fig. 1B). To the southeast toward the center of the Sahara, there are localities that share vertebrate taxa in southern Algeria and especially in the Echkar and Farak formations in Niger. Many of these sites are located in sections capped by a comparable plateau-forming late Cenomanian-Turonian limestone.

Guir Basin, western Algeria. Haug (1905) initially recorded *Onchopristis numidus* and *Ceratodus africanus* from Algerian sites. More recently, the geology and vertebrate remains have been described based on two localities in the Guir Basin of western Alge-

ria within 50 kms of the Moroccan-Algerian border near Béchar. Much of the faunal diversity and abundances present in the Kem Kem assemblage are also present in the Guir Basin, along with the predominance of theropod teeth, and absence of dinosaurian herbivores, birds, and mammals (Benyoucef et al. 2012, 2014, 2015). *Spinosaurus* teeth, in particular, outnumber other kinds of theropod teeth, although differentiating *Spinosaurus* from large crocodilian teeth can be challenging. Both localities (Kénadsa, Menaguir) show a fining-upward sequence with marked deposition of mud at mid-section, and this presumably corresponds to the formational division in the Kem Kem Group. The sediments and fauna suggest a marginal marine setting, and the thin stratigraphic section (~ 10 m) includes an overlying Cenomanian-Turonian limestone platform (Benyoucef and Meister 2015, Benyoucef et al. 2015: fig. 3).

Tataouine region, southern Tunisia. Field work over the last two decades in the Tataouine region in southern Tunisia (Fig. 1) has clarified the stratigraphy and vertebrate paleontology of rocks exposed along the north-south trending Dahar Plateau (Benton et al. 2000, Fanti et al. 2012). The regional geology, which resembles that in outcrops in neighboring Libya, is characterized by two well exposed formations (Ain el Guettar, Zebbag) with age estimates spanning Aptian-Cenomanian stages (Fanti et al. 2012: fig. 16).

Ain el Guettar Formation deposition, resting on an erosional unconformity, is composed of basal fluvial sandstones marked by channels grading upsection into estuarine and subtidal sandstones of the Oum ed Diab Member. It reaches a thickness of up to ~200 m comparable to the Kem Kem Group. Its age has been variously estimated to be as old as Barremian (Busson 1967, 1970) or as young as late Albian (Fanti et al. 2015). We regard the Aptian-Albian age as most likely, given the recent discovery of diagnostic remains of the giant crocodyliform *Sarcosuchus* (Dridi 2018) and the smaller notosuchian *Araripesuchus*.

Abundant fragmentary remains have been recovered of elasmobranchs, actinopterygians, sarcopterygians, crocodyliforms, pterosaurs, and saurischian dinosaurs. Unfortunately, currently known elasmobranch genera do not constrain the age estimate to a particular stage. The first partial articulated skeleton of a dinosaur from Tunisia was found recently in the basal part of the formation, the rebbachisaurid *Tataouinea hannibalis* (Fanti et al. 2015). Phylogenetic analysis of the skeleton places it closer to *Rebbachisaurus* from Morocco than to *Nigersaurus* from Niger (Fanti et al. 2015: fig. 22). As in the slightly older Elrhaz Formation in Niger (Aptian-Albian), and in the slightly younger Kem Kem Group in Morocco (Cenomanian), titanosaurian sauropods are also present (Fanti et al. 2014). In sum, the fauna from the Ain el Guettar Formation is probably older than the Kem Kem assemblage and either intermediate or closer to the age of the older Aptian-Albian fauna of the Elrhaz Formation in Niger.

The base of the overlying Zebbag Formation (Kerker Member) begins with a distinctive carbonate bed including the ammonite *Knemiceras* that is regarded as late Albian in age. Unfortunately, this marked the initiation of a marine transgression in the central region of coastal northern Africa, with subsequent deposition characterized by carbonaceous marls and evaporites indicative of an offshore marine platform (Fanti

et al. 2012: fig. 16). No vertebrate remains have been recovered from this formation, although the Kerker Member has yielded footprints of birds and mammals (Contessi and Fanti 2012, Contessi 2013).

Mizdah Formation, western Libya. Nessov et al. (1998) found a diverse marine and terrestrial vertebrate assemblage of teeth, bones, bone fragments and fin spines at the locality Draa Ubari in the Mizdah Formation of western Libya. They considered the beds as Santonian-Coniacian in age (~86 Ma) without explanation, focusing their paleontological attention on an isolated caudal vertebra of a mammal and a large sampling of vertebrae of a marine snake, which they attributed to a new species, *Simoliophis libycus*. The presence of abundant teeth of the sawfish *Onchopristis*, however, should have immediately tipped them to the potential for a slightly older Cenomanian age, comparable to the Kem Kem Group in Morocco and Bahariya Formation in Egypt where teeth of this elasmobranch are common.

Rage and Cappetta (2002) corrected the probable age of the Draa Ubari locality as Cenomanian, based on three elasmobranchs (*Onchopristis* sp., *Distobatus* sp., *Haimirichia amonensis*) and the marine snake *Simoliophis libycus*. More geological and paleontological work is needed at Draa Ubari and adjacent outcrops to better characterize the paleoenvironments and fauna present in this region of Africa's northern margin.

Bahariya Formation, western Egypt. The Bahariya Formation rests unconformably on much older basement rocks, the contact poorly exposed. The formation reaches a thickness of ~ 200 m in the region of the Bahariya Oasis (Catuneanu et al. 2006), comparable to that of the Kem Kem Group, representing a low-energy, low-gradient coastal plain. Deposits include lagoons, tidal flats and channels, oyster reefs, and mangroves (Lacovara et al. 2003), with most of the dinosaur material derived from a basal sandstone unit.

There is considerable overlap in the faunal assemblages between the Bahariya Formation of western Egypt (Ijouiher 2016) and the Kem Kem Group (Table 8), the latter generally better known. Common faunal elements include crabs, elasmobranchs including the sawfish *Onchopristis*, bony fish including the large cladistian *Bawitius* and the lepisosteiform *Obaichthys*, basal sarcopterygians including the actinistian *Axelrodichthys* and dipnoan *Neoceratodus*, pleurodiran turtles, squamates including the sea snake *Simoliophis*, crocodylomorphs including stomatosuchids, titanosaurian sauropods, and the three large predators *Deltadromeus agilis*, *Spinosaurus aegytiacus*, and *Carcharodontosaurus saharicus*. Both faunas lack evidence of Aves or Mammalia. One element in the Bahariyan fauna not yet recorded in the Kem Kem Group is a polycotylid plesiosaur, although a well preserved plesiosaur skull and skeleton (*Thililua longicollis*) have been discovered in the Cenomanian-Turonian limestone (Akrabou Formation) northwest of the Kem Kem embayment near Goulmima (Bardet et al. 2003).

Echkar Formation, central Niger. Several genera in the Kem Kem assemblage have also been reported to the southeast in the Echkar Formation of Niger (Fig. 1), which has been argued to be of comparable Cenomanian age (Sereno et al. 2004). The most fossiliferous outcrops of the Echkar Formation are located in the region of Iguidi, overlain by a prominent Cenomanian-Turonian limestone plateau.

The fauna shows less marine influence, as it was a good distance from a substantial marine margin. Common faunal elements between the Kem Kem Group and the Echkar Formation include large cladistians, basal sarcopterygians including the actinistian *Axelrodichthys* and dipnoan *Neoceratodus*, pleurodiran turtles, crocodylomorphs including araripesuchids and an unnamed very large neosuchian (Larsson et al. 2015), rebbachisaurid and titanosaurian sauropods, an abelisaurid theropod similar to *Rugops primus* (Sereno et al. 2004), and the two large predators *Spinosaurus aegyptiacus* and *Carcharodontosaurus saharicus* (Fig. 131F, G). Both faunas lack evidence of Aves or Mammalia.

Conclusions

Kem Kem paleoenvironments

Kem Kem Group formations are envisioned as the headland of a vast river system feeding north to a prograding delta laid down on the Kem Kem embayment (Guiraud et al. 2005, Fig. 33). The sequence begins with conglomeratic beds deposited in anastomosing channels on weathered Paleozoic strata (Fig. 32, stage 1). Prograding delta sediments of the Gara Sbaa Formation, (Fig. 32, stage 2) give way to coastal deposits and sabkas of the Douira Formation (Fig. 32, stage 3), which are suddenly overrun by a marine transgression of the Akrabou Formation (Fig. 32, stage 4). The Gara Sbaa and Douira formations, thus, capture the transition, likely in the Early and Middle Cenomanian, from fluvial-deltaic to lower-energy coastal, pond and sabkha paleoenvironments.

Similar conditions have generated similar fining-upward sequences in basins elsewhere in Morocco (Cavin et al. 2010) and western Algeria (Benyoucef at al. 2015). Farther east along the southern shore of the Tethys Sea near present day Tunisia, the marine transgression inundates the coastal margin during the Cenomanian, sooner than in the Kem Kem region (Fanti et al. 2012). The Dahar plateau preserves fluvial and lagoonal deposits laid down on a prograding delta that infilled the Tatouine Basin (Anderson et al. 2007). Coastal mangrove deposits characterize the Cenomanian-age Bahariya Formation in Egypt (Smith et al. 2001) with open-water lagoon, tidal flat, and tidal channel facies (Lacovara et al. 2003). All of the above-cited regions along the northern shores of Africa involve low-gradient marine coastal plains with broad back-barrier deltaic environments that exhibit both marine and freshwater influences.

The Kem Kem delta was dominated by rapidly moving (lotic) paleoenvironments with water flowing toward the open ocean and much rarer still water (lentic) paleoenvironments such as ponds (Oum Tkout locality). In the dominant lotic paleoenvironments, there is evidence for both freshwater and brackish conditions; some of the recovered fauna (e.g., dipnoans) prefer freshwater habitats whereas others (e.g., lamnifom sharks) prefer brackish conditions. Some aquatic genera, such as *Onchopristis* or *Axelrodichthys* may have been adapted to both freshwater and marine paleoenvironments. In the upper portion of the Gara Sbaa Formation and the Douira Formation,

tidal indicators suggest brackish conditions may have predominated as the transgression continued. Hothouse conditions characterized by harsh seasonality, periodic aridity and elevated sea-surface temperatures likely prevailed during deposition of Kem Kem Group rocks and comparable deposits along the northern shore of Africa (Wendler and Wendler 2016).

Gara Sbaa Formation, a prograding delta. The conglomeratic components at the base of the Gara Sbaa Formation are locally derived clasts from underlying Paleozoic strata (Fig. 32, stage 1). The thick sequences of overlying sandstones are characterized by laterally extensive cross-bedding deposited in broader, deeper fluvial channels (Fig. 32, stage 2). The rarity of mudstones indicates lateral reworking in channels of earlier channel and floodplain deposits, which also generated pebble lags and incorporated extensive vertebrate tooth and bone debris (Rogers 1993, Fig. 36).

Tidal influence indicative of the proximity of coastal settings first appears in the upper portion of the Gara Sbaa Formation and into the Douira Formation and includes mud drapes, flaser and lenticular bedding. The upper portion of the Gara Sbaa Formation exhibits features of a prograding delta front, including larger, possibly tidally influenced, channels and large-scale cross-bedding (Fig. 24).

Most vertebrate fossils are isolated either in laminar cross-bedded sandstones or in channel lag deposits. Only two partial articulated vertebrate skeletons have been recovered in the formation, both from the uppermost portion of the section. The first is a partial skeleton of the diplodocoid sauropod *Rebbachisaurus garasbae* (Fig. 104; Lavocat 1951, Wilson and Allain 2015), and the second is the partial skeleton of the enigmatic theropod *Deltadromeus agilis* (Figs 115–118; Sereno et al. 1996).

Douira Formation, a coastal mudflat. The first significant mudstone bed identifies the base of the lower-energy Douira Formation, which is composed of finer-grained strata that often exhibit tidal influences. Sandstones are more common near the base of the formation, giving way to ledge-forming Siltstone, mudstone, claystone and thin gypsiferous evaporites. Douira beds, like those in the Gara Sbaa Formation, are predominantly red-hued rocks that fine upward as well as northward along the outcrop toward the mouth of the delta.

Mudstones and claystones dominate Douira beds and are characterized by mottling, slickensides, and blocky to crumbly textures with rare calcitic nodules, gypsum crystals, root traces and burrows. Whereas a thin (1 cm) gypsum horizon occurs just below a calcareous mudstone, evaporite deposits of similar lithology are thicker and more common in Kem Kem Group rocks to the west and north of the Kem Kem embayment.

Two other features of the Douira Formation are noteworthy. A freshwater pond deposit, Oum Tkout, is located low in the section halfway along the south-north axis of outcrops on the delta (Fig. 9, locality 6). Its laminated clays preserve a spectacular array of hard and soft-bodied vertebrates, nonvertebrates, and plants (e.g., Dutheil 1999a, 1999b). The second feature, high in the section, are footprint horizons that record, at times with high fidelity and remarkable depth, dinosaur tracks and other ichnological traces (Sereno et al. 1996, Ibrahim et al. 2014a).

The lower portion of the Douira Formation has yielded associated remains of vertebrates, including a partial shell of the pleurodire turtle *Galianemys* sp., a partial skeleton of *Spinosaurus aegyptiacus* (Ibrahim et al. 2014b), and a partial skull of *Carcharodontosaurus saharicus* (Figs 72, 129, 133–137; Sereno et al. 1996).

Kem Kem paleoecosystem

"Stomer's riddle". The overabundance of predatory versus herbivorous dinosaurs, recently dubbed "Stromer's riddle" (McGowan and Dyke 2009), was first noticed by Stromer (1936) in the Bahariya Formation of Egypt (Fig. 1). Theropod overdominance characterizes several localities in northern Africa during Cenomanian times (Sereno et al. 1996, Russell 1996, Russell and Paesler 2003, Läng et al. 2013, Ibrahim et al. 2014a). At least four large-bodied theropod genera were present in the Kem Kem assemblage: an abelisaurid, *Spinosaurus aegyptiacus*, *Carcharodontosaurus saharicus*, and *Deltadromeus agilis*. A similar suite of four large-bodied theropods are present in contemporaneous beds in Egypt (Stromer 1934, 1936), and at least the first three are present in less fossiliferous Cenomanian-age deposits in Algeria (Benyoucef et al. 2015) and Niger (Sereno and Brusatte 2008).

In most Mesozoic terrestrial assemblages, only one or two large-bodied theropod genera are present. *Tyrannosaurus rex*, for example, may be the only large-bodied predator in the Late Cretaceous (Maastrichtian) habitats on Laramidia (Horner et al. 2011), whereas *Tarbosaurus* and the longer-snouted *Alioramus* may have co-occurred in Asia during the same interval (Brusatte and Carr 2016). Three large-bodied predators (*Ceratosaurus*, *Torvosaurus* and *Allosaurus*) have been recorded in the Late Jurassic (Kimmeridgian-Tithonian) Morrison Formation of western North America. Marked differences between these predators in skull architecture and tooth size and shape suggest marked differences in feeding (Henderson 1998), and only one of the triumvirates (*Allosaurus*) is present and abundant at many sites.

Two additional features set apart the Kem Kem and Bahariya predators and their faunas, in addition to the overabundance of large-bodied dinosaurian predators. First, at least three of the four large-bodied predators present in both the Kem Kem and Bahariya assemblages are among the largest (top 10%) dinosaurian predators on record. Maximum adult body size for *Carcharodontosaurus* and *Spinosaurus* is based on Kem Kem specimens (Sereno et al. 1996, Dal Sasso et al. 2005), and both genera are widely appreciated as > 12 m long "giants". The largest specimen of *Deltadromeus* is a femur from the Bahariya Formation (Stromer 1934). Destruction of the original Bahariyan material has resulted in little notice of the size of the femur now attributed to *Deltadromeus* (Sereno et al. 1996). With a femoral length of 122 cm (Stromer 1934), the femur is 165% greater than the length of that of the immature holotypic specimen of *Deltadromeus* from the Gara Sbaa Formation (Fig. 118B). The Egyptian femur is 91% the length of the femur in *Tyrannosaurus* at maximum adult body size (Brochu 2003).

Thus, not only are there four large-bodied predators in the Kem Kem and Bahariya assemblages, three are among the largest land predators known.

Second, large-bodied herbivores are neither diverse nor abundant. Only three large bodied herbivores have been recovered as diagnostic body fossils in the Kem Kem and Bahariya assemblages, the rebbachisaurid *Rebbachisaurus garasbae* from the Gara Sbaa Formation (Lavocat 1954, Wilson and Allain 2015) and the titanosaurians *Paralititan stromeri* and *Aegyptosaurus baharijensis* (Smith et al. 2001) from the Bahariya Formation. The companion titanosaurian in the Kem Kem Group (Ibrahim et al. 2016) and rebbachisaurid in the Bahariya Formation (Stromer 1932) are known only from fragmentary material. Additionally, there is a single footprint of a large iguanodontian in the Douira Formation (Sereno et al. 1996, Ibrahim et al. 2014a). Thus, large-bodied herbivores likely co-occurred with the four large-bodied predators in the Kem Kem assemblage, but they are not as diverse as in many other Cretaceous formations nor particularly common as fossils.

Signature Cretaceous fauna, northern Africa. The Kem Kem assemblage is dominated by aquatic and subaquatic nonvertebrates and vertebrates (~85%, ~40 species), nearly all of which are predators (Table 8). Most of the described vertebrates, with the exception of some of the pterosaurs and dinosaurs, lived exclusively or predominantly within an aquatic setting, which would include pond, river, delta, and nearshore habitats. Most of the taxa in the assemblage, thus, are predators utilizing aquatic food resources as in modern marine food webs (Friedlander et al. 2012).

Co-occurrence of a trio of large-bodied theropod predators, an abelisaurid, spinosaurid and carcharodontosaurid, characterizes faunas of northern Africa for a duration of at least 20 Ma from the middle (Aptian-Albian) to early Late Cretaceous (Cenomanian) time (Sereno and Brusatte 2008, Fanti et al. 2014). Because the fossil record before and after this interval is poorly represented on Africa, 20 Ma is the minimum duration for this ecological guild (Simberloff and Dayan 1991) of large-bodied predators.

The earliest record of this trio is approximately 115 Ma in the Aptian-Albian Elrhaz Formation of Niger. The spinosaurid *Suchomimus* (Sereno et al. 1998) is the best known and most abundant compared to the rare (single) bone records of the abelisaurid *Kryptops* and carcharodontosaurid *Eocarcharia* (Sereno and Brusatte 2008). In skull shape and tooth form, these three predators are strongly divergent. *Suchomimus* has an extremely long skull and specialized dentition with an ensnaring premaxillary terminal rosette; *Kryptops* has a very short skull with small recurved teeth of similar size; *Eocarcharia* had a skull of intermediate length and more typical tetanurine shape with recurved, blade-shaped teeth that reach maximum size near the front of the maxilla. From these cranial and dental specializations, we infer that the dominant mode of feeding may have been fish-eating, scavenging, and macropredaceous hunting, respectively.

Fanti et al. (2014) suggested that there may be paleoecological separation of spinosaurids from abelisaurids and carcharodontosaurids based on the predominance of spinosaurid teeth in estuarine deposits (Oum ed Diab Member) versus the presence of all three in underlying channel deposits (Chenini Member and basal reworked unit of the Oum ed Diab Member). We regard such conclusions, based solely on isolated transported teeth in a crocodylomorph-dominated assemblage, to be poorly supported. We never found such paleoecological separation among this trio of predators in either

formation in the Kem Kem Group nor in the Elrhaz or Echkar formations in Niger. The Elrhaz Formation, which is composed almost exclusively of fluvial cross-bedded sandstones distant from any marine margin, would be regarded as more terrestrial than the overlying Echkar Formation and either of the formations in the Kem Kem Group. Yet, *Suchomimus* is clearly the most abundant large-bodied theropod in the Elrhaz Formation, based on isolated remains and several partial articulated skeletons. That surprising circumstance underscores the ecological flexibility, noncompetitive nature, and robustness of this ecological guild of predators, which thrived for at least 20 Ma, despite varying local habitats and environmental conditions. In Niger, as best as can be discerned in the field in both the Elrhaz and Echkar formations, this trio seem to co-occur during the entire transgressive sequence from inland to nearshore habitats.

Three other aspects of the Elrhaz fauna are also present in the younger Kem Kem and Bahariya faunas. First, large-bodied herbivores include iguanodontian ornithischians and two ecologically distinct sauropods (a rebbachisaurid and titanosaurian). In both faunas, thus, large herbivores remain a mixture of ornithopods and macronarian sauropods, which is not always the case on other landmasses during the Late Cretaceous.

Second, crocodylomorphs are very diverse and include at least one giant form with a skull length approaching 2 m. In the Elrhaz Formation, that crocodylomorph giant is *Sarcosuchus* (Sereno et al. 2001), which has been reported as far north as Tunisia (Dridi 2018). In both the Echkar Formation in Niger and the Kem Kem Group, an unnamed genus with skull size nearly equaling *Sarcosuchus* is present (Larsson et al. 2015). Likewise, in the Bahariya Formation of Egypt, the skull of the unusual sit-and-wait predator *Stomatosuchus* also approaches 2 m in length (Stromer 1925, 1936, Sereno and Larsson 2009).

Finally, mammals and birds are conspicuously absent or very rare in the Aptian-Albian Elrhaz Formation and in the younger (Cenomanian) Echkar, Kem Kem, and Bahariya faunas, although tracks of both groups were recently reported in the Cenomanian-age Zebbag Formation of southern Tunisia (Contessi and Fanti 2012; Contessi 2013). Mammals have been recorded in Early Cretaceous rocks of northern Africa (Sigogneau-Russell 1995, Heinrich 1998), so their rarity in formations of Aptian-Cenomanian age across northern Africa, which include fluvial and pond settings that have been extensively sampled and screen-washed, is notable and contrasts markedly with the record of these two groups from similar age Cretaceous deposits on northern landmasses.

Acknowledgements

We thank Simone Maganuco and Jeffrey Wilson for comments on earlier versions of this paper, Carol Abraczinskas for assistance with the figures, and Marco Auditore for skeletal drawings of *Spinosaurus*, *Carcharodontosaurus*, and *Deltadromeus*. Figure 8 created by DMM and Roy Smith. Figure 130 courtesy of the Fossil Lab, University of Chicago. The reviewers of this paper, Cristiano Dal Sasso, Daniela Schwartz, and Romain Vullo, provided helpful comments and suggestions, as did three anony-

mous experts on several of the taxonomic groups discussed in this paper. For access to material in their care, we thank Ronan Allain, Paul Barrett, Alain Cabot, Steve Cumbaa, Cristiano Dal Sasso, Margaret Feuerstack, Angela Milner, Oliver Rauhut, Kieran Shepherd, and Hans-Dieter Sues. Funding for fieldwork was provided by the National Geographic Society (to NI, PCS), University College Dublin (Ad Astra Research Scholarship to NI), the University of Portsmouth (to DMM), the Muséum national d'Histoire naturelle (to DBD), the National Science Foundation (to PCS), The Packard Foundation (to PCS), and The Women's Board of the University of Chicago (to PCS). The review of the fossil material from the Kem Kem Group began as part of a doctoral dissertation at the University College Dublin and postdoctoral study at the University of Chicago (by NI).

References

Allain R (2014) New material of the theropod *Ichthyovenator* from ban Kalum type locality (Laos): implications for the synonymy of *Spinosaurus* and *Sigilmassasaurus* and the phylogeny of Spinosauridae. Journal of Vertebrate Paleontology, Program and Abstracts 34: 78A.

Allain R, Xaisanavong T, Richir P, Khentavong B (2012) The first definitive Asian spinosaurid (Dinosauria: Therpoda) from the Early Cretaceous of Laos. Naturwissenschaften 99: 369–377. https://doi.org/10.1007/s00114-012-0911-7

Alloul T, Rage J-C, Hamididouche R, Jalil N-E (2018) First report on Cretaceous vertebrates from the Algerian Kem Kem beds. A new procoelous salamander from the Cenomanian, with remarks on African Caudata. Cretaceous Research 84: 384–388. https://doi.org/10.1016/j.cretres.2017.11.019

Amiot R, Buffetaut E, Tong H, Boulad L, Kabiri L (2004) Isolated theropod teeth from the Cenomanian of Morocco and their palerbiogeographical significance. Revue de Paléobiologie, volume special, 9: 143–149.

Amiot R, Buffetaut E, Lécuyer C, Wang X, Boudad L, Ding Z, Fourel F, Hutt S, Martineau F, Medeiros MA, Mo J, Simon L, Suteethorn V, Sweetman S, Tong H, Zhang F, Zhou Z (2010) Oxygen isotope evidence for semi-aquatic habits among spinosaurid theropods. Geology 38: 139–142. https://doi.org/10.1130/G30402.1

Anderson PE, Benton MJ, Trueman CN, Paterson BA, Cuny G (2007) Palaeoenvironments of vertebrates on the southern shore of Tethys: The nonmarine Early Cretaceous of Tunisia. Palaeogeography, Palaeoclimatology, Palaeoecology 243: 118–131. https://doi.org/10.1016/j.palaeo.2006.07.015

Apesteguía S, Zaher H (2006) A Cretaceous terrestrial snake with robust hindlimbs and a sacrum. Nature 440: 1037–1040. https://doi.org/10.1038/nature04413

Apesteguía S, Daza JD, Simões TR, Rage J-C (2016a) The first iguanian lizard from the Mesozoic of Africa. Royal Society Open Science 3: 160462. https://doi.org/10.1098/rsos.160462

Apesteguía S, Smith ND, Juárez Valieri R, Makovicky PJ (2016b) An unusual new theropod with a didactyl manus from the Upper Cretacoeus of Patagonia, Argentina. PLoS ONE 11(7): e0157793.. https://doi.org/10.1371/journal.pone.0157793

Arden TMS, Klein CG, Zourhi S, Longrich NR (2019) Aquatic adaptation in the skull of carnivorous dinosaurs (Theropoda: Spinosauridae) and the evolution of aquatic habits in spinosaurids. Cretaceous Research 93: 275–284. https://doi.org/10.1016/j.cretres.2018.06.013

Averianov AO (2014) Review of taxonomy, geographic distribution, and paleoenvironments of Azhdarchidae (Pterosauria) ZooKeys 432: 1–107. https://doi.org/10.3897/zookeys.432.7913

Averianov AO, Arkhangelsky MS, Pervushov EM (2008) A new Late Cretaceous azhdarchid (Pterosauria, Azhdarchidae) from the Volga region. Paleontological Journal 42: 634–642. https://doi.org/10.1134/S0031030108060099

Baidder L (2007) Structuration de la bordure septentrionale du Craton Ouest-Africain du Cambrien à l'Actuel : cas de l'Anti-Atlas oriental du Maroc. (Thèse d'Etat). Université Hassan II Aïn Chock, Faculté des sciences, Casablanca, Maroc.

Baidder L, Michard A, Soulaimani A, Fekkak A, Eddebbi A, Rjimati EC, Raddi Y (2016) Fold interference pattern in thick-skinned tectonics; a case study from the external Variscan belt of Eastern Anti-Atlas, Morocco. Journal of African Earth Sciences 119: 204–225. https://doi.org/10.1016/j.jafrearsci.2016.04.003

Bailey JB (1997) Neural spine elongation in dinosaurs: sailbacks or buffalo-backs? Journal of Paleontology 71: 1124–1146. https://doi.org/10.1017/S0022336000036076

Bardet N, Suberbiola XP, Jalil NE (2003) A new polycotylid plesiosaur from the Late Cretaceous (Turonian) of Morocco. Comptes Rendus Palevol, 2: 307–315. https://doi.org/10.1016/S1631-0683(03)00063-0

Behrensmeyer AK (1982) Time resolution in fluvial vertebrate assemblages. Paleobiology 8: 211–227. https://doi.org/10.1017/S0094837300006941

Behrensmeyer AK (1991) Terrestrial Vertebrate Accumulation. In: Allison P, Briggs DEG (Eds) Taphonomy: Releasing the Data Locked in the Fossil Record. Plenum, New York, 291–335. https://doi.org/10.1007/978-1-4899-5034-5_6

Behrensmeyer AK, Rogers RR (2017) Tyranny of transport in taphonomy: how far do bones really move and how much does it matter? Journal of Vertebrate Paleontology, e-Supplement 37: 79A, 80A.

Bellion Y, Lefranc JP, Michard J-G (1990) Précisions sur l'age des sédiments méso-cénozoiques à l'ouest de l'Adrar des Iforas (Sahara, Mali, Afrique de l'ouest): implications paléógéographiques. Actes du Congrès National des Societes Savantes 115 Section des Sciences, 7–27.

Belvedere M, Jalil N-E, Breda A, Gattolin G, Bourget H, Khaldoune F, Dyke GJ (2013) Vertebrate footprints from the Kem Kem beds (Morocco): A novel ichnological approach to faunal reconstruction. Palaeogeography, Palaeoclimatology, Palaeoecology 383–384: 52–58. https://doi.org/10.1016/j.palaeo.2013.04.026

Bennett SC (1995) A statistical study of *Rhamphorhynchus* from the Solnhofen Limestone of Germany – year-classes of a single large species. Journal of Paleontology 69: 569–580. https://doi.org/10.1017/S0022336000034946

Benton MJ (1998) The quality of the fossil record of vertebrates. In: Donovan SK, Paul CRC (Eds) The Adequacy of the Fossil Record. Wiley, New York, 269–303.

Benton MJ (2008) How to find a dinosaur, and the role of synonymy in biodiversity studies. Paleobiology 34: 516–533. https://doi.org/10.1666/06077.1

Benton MJ, Bouaziz S, Buffetaut E, Martill D, Ouaja M, Soussi M, Trueman C (2000) Dinosaurs and other fossil vertebrates from fluvial deposits in the Lower Cretaceous of southern

Tunisia. Palaeogeography, Palaeoclimatology, Palaeoecology 157: 227–246. https://doi.org/10.1016/S0031-0182(99)00167-4

Benyoucef M, Meister C (2015) Lithostratigraphic evolution, facies analysis and depositional environment of the Cenomanian-lower Turonian in the Guir area, Southwestern Algeria. Cretaceous Research 53: 68–88. https://doi.org/10.1016/j.cretres.2014.10.009

Benyoucef M, Meister C, Bensalah M, Malti FZ (2012) La plateforme préafricaine (Cénomanien supérieur-Turonien inférieur) dans la région de Béchar (Algérie): stratigraphie, paléoenvironnements et signification paléobiogéographique. Revue de Paléobiologie 31: 205–218.

Benyoucef M, Läng E, Cavin L, Mbarki K, Adaci M, Bensalah M (2015) Overabundance of piscivorous dinosaurs (Theropoda: Spinosauridae) in the mid-Cretaceous of North Africa: The Algerian dilemma. Cretaceous Research 55: 44–55. https://doi.org/10.1016/j.cretres.2015.02.002

Benyoucef M, Adaci M, Meister C, Läng E, Malti FZ, Mebarki K, Cherif A, Zaoui D, Benyoucef A, Bensalah M (2014) Le «Continental Intercalaire» dans la région du Guir (Algérie): nouvelles données paléontologiques, ichnologiques et sédimentologiques. Revue de Paléobiologie 33: 281–297.

Bockwinkel J, Becker RT, Ebbinghausen V (2013) Late Givetian ammonoids from Hassi Nebech (Tafilalt Basin, Anti-Atlas, southern Morocco). Fossil Record 16: 5–65. https://doi.org/10.1002/mmng.201300001

Boucot AJ, Gray J (2001) A critique of Phanerozoic climate models involving changes in the CO_2 content of the atmosphere. Earth-Science Reviews 56: 1–159. https://doi.org/10.1016/S0012-8252(01)00066-6

Brett CE, Baird GC (1986) Comparative taphonomy: a key to paleoenvironmental interpretation using fossil preservation. Palaios 1: 207–227. https://doi.org/10.2307/3514686

Brito PM, Ferreira PLN (1989) The first hybodont shark, *Tribodus limae* n. sp. from the lower Cretaceous of Chapada do Araripe (North-East Brazil). Anais da Academia Brasileira de Ciências 61: 53–57.

Brochu CA (2003) Osteology of *Tyrannosaurus rex*: insights from a nearly complete skeleton and high-resolution computed tomographic analysis of the skull. Society of Vertebrate Paleontology Memoir 7: 1–138. https://doi.org/10.2307/3889334

Broin F de (1980) Les tortues de Gdoufaoua (Aptian du Niger): Apercu les palégéographie des Pelomedusidae (Pleurodira). Memoires de la Sociéte géologique de France 139: 39–46.

Broin F de, Taquet P (1966) Découverte d'un crocodilien nouveau dans le Crétacé inferieur du Sahara. Comptes Rendus de l'Académie des Sciences de Paris, Série D 262: 2326–2329.

Broin F de, Grenot C, Vernet R (1971) Sur la découverte d'un nouveau gisement de vertébrés dans le Continental Intercalaire saharien: la Gara Samani (Algérie). Comptes Rendus de l'Académie des Sciences à Paris, Série D 272: 1219–1221.

Broin F de, Buffetaut E, Koeniguer JC, Rage J-C, Russell DE, Taquet P, Vergnaud-Grazzini C, Wenz S (1974) La faune de vertébrés continentaux du gisement d'In Beceten (Sénonien du Niger). Comptes Rendus Hebdomadaires des Seances de l'Academie des Sciences à Paris, Série D 279: 469–472.

Broschinksi A, Sigogneau-Russell D (1996) Remarkable lizard remains from the Lower Cretaceous of Anoual (Morocco). Annales de Paléontologie (Vert.–Invert.) 82: 147–175.

Brusatte SL, Sereno PC (2007) A new species of *Carcharodontosaurus* (Dinosauria: Theropoda) from the Cenomanian of Niger and a revision of the genus. Journal of Vertebrate Paleontology 24: 902–916. https://doi.org/10.1671/0272-4634(2007)27[902:ANSOCD]2.0.CO;2

Brusatte SL, Carr TD (2016) The phylogeny and evolutionary history of tyrannosaurid dinosaurs. Scientific Reports 6, 20252. https://doi.org/10.1038/srep20252

Brusatte SL, Benson RBJ, Hutt S (2008) The osteology of *Neovenator salerii* (Dinosauria: Theropoda) from the Wealden Group (Barremian) of the Isle of Wight. Palaeontographical Society Monographs 162: 1–75.

Brusatte SL, Benson RBJ, Carr TD, Williamson TE, Sereno PC (2007) The systematic utility of theropod enamel wrinkles. Journal of Vertebrate Paleontology 27: 1052–1056. https://doi.org/10.1671/0272-4634(2007)27[1052:TSUOTE]2.0.CO;2

Buffetaut E (1974) Trematochampsa taqueti, un Crocodilien nouveau du Senonien inferieur du Niger. Comptes rendus hebdomadaire des séances de l'Academie des Sciences Paris 279: 1749–1752.

Buffetaut E (1976) Der Land-Krokodilier *Libycosuchus* Stromer und die Familie Libycosuchidae (Crocodylia, Mesosuchia) aus der Kreide Afrikas. Mitteilungen Bayerische staatssammlung für Paläontologie und Historische Geologie 51: 1–20.

Buffetaut E (1989) New remains of the enigmatic dinosaur *Spinosaurus* from the Cretaceous of Morocco and the affinities between *Spinosaurus* and *Baryonyx*. Neues Jahrbuch fur Geologie und Paläontologie Monatshefte 79–87. https://doi.org/10.1127/njgpm/1989/1989/79

Buffetaut E (1992) Remarks on the Cretaceous theropod dinosaurs *Spinosaurus* and *Baryonyx*. Neues Jahrbuch für Geologie und Paläontologie Monatshefte 2: 88–96. https://doi.org/10.1127/njgpm/1992/1992/88

Buffetaut E (1994) A new crocodilian from the Cretaceous of southern Morocco. Comptes rendus de l'Académie des Sciences de Paris 319: 1563–1568.

Busson G (1967) Le Mésozoïque saharien, 1ière partie: l'Extrême Sud tunisien. Publications du Centre de Recherche sure les Zones arides, Centre National de la Recherche Scientifique, Paris, série Géologie 8: 1–194.

Busson G (1970) Le Mésozoïque saharien. 2ème partie: Essai des synthèse des données des sondages algéro-tunisiens. Publication du Centre de Recherche sur la Zones arides, Centre National de la Recherche Scientifique, Paris, Série Géologie 11: 1–810.

Cailleux A, Soleilhavoup F (1976) Les kerkoubs, concrétions calcareuses du sud-algérien. Géologie Méditerranéenne 3: 251–264. https://doi.org/10.3406/geolm.1976.981

Caldwell MW, Lee MSY (1997) A snake with legs from the marine Cretaceous of the Middle East. Nature 386: 705–709. https://doi.org/10.1038/386705a0

Caldwell MW, Nydam RL, Palci A, Apesteguía S (2015) The oldest known snakes from the Middle Jurassic-Lower Cretaceous provide insights on snake evolution. Nature Communications 2015: 11. https://doi.org/10.1038/ncomms6996

Calvo JO, Salgado L (1995) *Rebbachisaurus tessonei* sp. nov. a new Sauropoda from the Albian-Cenomanian of Argentina; new evidence on the origin of the Diplodocidae. Gaia 11: 13–33.

Calvo JO, Coria RA (1998) New specimen of *Giganotosaurus carolinii* (Coria & Salgado, 1995) supports it as the largest theropod ever found. Gaia 15: 117–122.

Campos D de A, Kellner AWA (1985) Panorama of the flying reptiles study in Brazil and South America (Pterosauria/ Pterodactyloidea/ Anhangueridae). Anais de Academia Brasileira de Ciências 57: 141–142, 453–466.

Candeiro CRA, Fanti F, Therrien F, Lamanna MC (2011) Continental fossil vertebrates from the mid-Cretaceous (Albian-Cenomanian) Alcântara Formation, Brazil, and their relationship with contemporaneous faunas from North Africa. Journal of African Earth Sciences 60: 79–92. https://doi.org/10.1016/j.jafrearsci.2011.02.004

Cappetta H, Case GR (1975) Sélaciens nouveaux du Crétacé du Texas. Geobios 8: 303–307. https://doi.org/10.1016/S0016-6995(75)80028-3

Carnier Fragoso LG, Brito P, Yabumoto Y (2019) *Axelrodichthys* araripensis Maisey, 1986 revisited. Historical Biology 31: 1350–1372. https://doi.org/10.1080/08912963.2018.1454443

Carrano MT, Sampson SD (2008) The phylogeny of ceratosauria (Dinosauria: Theropoda). Journal of Systematic Palaeontology 6: 183–236. https://doi.org/10.1017/S1477201907002246

Carvalho IS, Campos DA (1988) Um mamífero triconodonte do Cretáceo Inferior do Maranhão, Brasil. Anais da Academia Brasileira de Ciências 60: 437–446.

Carvalho MSS, Maisey JG (2008) New occurence of *Mawsonia* (Sarcopterygii: Actinista) from the Early Cretaceous of the Sanfranciscana Basin, Minas Gerais. Geological Society, London, Special Publications, 295: 109–144. https://doi.org/10.1144/SP295.8

Catuneanu O, Khalifa MA, Wanas HA (2006) Sequence stratigraphy of the Lower Cenomanian Bahariya Formation, Bahariya Oasis, Western Desert, Egypt. Sedimentary Geology 190: 121–137. https://doi.org/10.1016/j.sedgeo.2006.05.010

Cau A, Maganuco S (2009) A new theropod dinosaur, represented by a single unusual caudal vertebra, from the Kem Kem Beds (Cretaceous) of Morocco. Atti della Società Italiana di Scienze Naturali e del Museo Civico di Storia Naturale di Milano 150: 239–257.

Cau A, Dalla Vecchia FM, Fabbri M (2012) Evidence for a new carcharodontosaruid from the Upper Cretaceous of Morocco. Acta Palaeontologica Polonica 57: 661–665. https://doi.org/10.4202/app.2011.0043

Cau A, Dalla Vecchia FM, Fabbri M (2013) A thick-skulled theropod (Dinosauria, Saurischia) from the Upper Cretaceous of Morocco with implications for carcharodontosaurid cranial evolution. Cretaceous Research 40: 251–260. https://doi.org/10.1016/j.cretres.2012.09.002

Cavin L, Brito PM (2001) A new Lepisosteidae (Actinopterygii, Ginglymodi) from the Cretaceous of the Kem Kem beds, Southern Morocco. Bulletin de la Société Géologique de France 172: 141–150. https://doi.org/10.2113/172.5.661

Cavin L, Dutheil DB (1999) A new Cenomanian ichthyofauna from Southeastern Morocco and its relationships with the other early Late Cretaceous Moroccan faunas. Geologie en Mijnbouw 78: 261–266. https://doi.org/10.1023/A:1003810123806

Cavin L, Forey PL (2001a) Osteology and systematic affinities of *Palaeonotopterus greenwoodi* Forey 1997 (Teleostei: Osteoglossomorpha). Zoological Journal of the Linnean Society 133: 25–52. https://doi.org/10.1111/j.1096-3642.2001.tb00621.x

Cavin L, Forey PL (2001b) New coelacanth fish remains from the Cretaceous of the Kem Kem beds, SE Morocco. In: Tintori A, Arratia G (Eds) Mesozoic Fishes 3 – Systematics, Paleoenvironnements and Biodiversity. Verlag Dr. Friedrich Pfeil, Munich, 493–506.

Cavin L, Forey PL (2004) New mawsoniid coelacanth (Sarcopterygii, Actinistia) remains from the Cretaceous of the Kem Kem beds, SE Morocco. In: Tintori A, Arratia G (Eds), Mesozoic Fishes 3 – Systematics, Paleoenvironments and biodiversity. Verlag Dr. Friedrich Pfeil, Munich, 493–506.

Cavin L, Boudad L, Tong H, Läng E, Tabouelle J, Vullo R (2015) Taxonomic composition and trophic structure of the continental bony fish assemblage from the early Late Cretaceous of southeastern Morocco. PLoS ONE 10(5): e0125786. https://doi.org/10.1371/journal.pone.0125786

Cavin L, Boudad L, Duffaud S, Kabiri L, Le Loeuff J, Rouget I, Tong H (2001) L'évolution paléoenvironnementale des faunes de poissons du Crétacé supérieur du bassin de Tafilalt et des régions avoisinantes (Sud-Est du Maroc), implications paléobiogéographiques. Comptes Rendus de l'Académie des Sciences Série IIa 333: 677–683. https://doi.org/10.1016/S1251-8050(01)01674-3

Cavin L, Tong H, Boudad L, Meister C, Piuz A, Tabouelle J, Aarab M, Amiot R, Buffetaut E, Dyke G, Hua S, Le Loeuff J (2010) Vertebrate assemblages from the early Late Cretaceous of southeastern Morocco: An overview. Journal of African Earth Sciences 57: 391–412. https://doi.org/10.1016/j.jafrearsci.2009.12.007

Charig AJ, Milner AC (1997) *Baryonyx walkeri*, a fish-eating dinosaur from the Wealden of Surrey. Bulletin of the Natural History Museum of London (Geol.) 53: 11–70.

Chiarenza AA, Cau A (2016) A large abelisaurid (Dinosauria, Theropoda) from Morocco and comments on the Cenomanian theropods from North Africa. PeerJ 4: e1754. https://doi.org/10.7717/peerj.1754

Choubert G (1939) Sur le Crétacé du Maroc oriental. Comptes rendus de l'Académie des Sciences de Paris 208: 209–211.

Choubert G (1948) Essai sur la paléogéographie du Mésocrétacé marocain. Société des Sciences naturelles du Maroc, 1920–1945, 307–329.

Choubert G (1952) Histoire géologique du domaine de l'Anti-Atlas. Géologie du Maroc. Service Géologique, Notes et Mémoires 100, 75–95.

Churcher CS, De Iuliis G (2001) A new species of *Protopterus* and a revision of *Ceratodus humei* (Dipnoi: Ceratodontiformes) from the Late Cretaceous Mut Formation of eastern Dakhleh Oasis, Western Desert of Egypt. Palaeontology 44: 305–323. https://doi.org/10.1111/1475-4983.00181

Colbert EH (1981) A primitive ornithischian dinosaur form the Kayenta Formation of Arizona. Bulletin of the Museum of Northern Arizona 53: 1–61.

Contessi M (2013) First report of mammal-like tracks from the Cretaceous of North Africa (Tunisia). Cretaceous Research 42: 48–54. https://doi.org/10.1016/j.cretres.2013.01.003

Contessi M, Fanti F (2012) First record of bird tracks in the Late Cretaceous (Cenomanian) of Tunisia. Palaios 27: 455–464. https://doi.org/10.2110/palo.2011.p11-114r

Coria RA, Salgado L (1995) A new giant carnivorous dinosaur from the Cretaceous of Patagonia. Nature 377: 224–226. https://doi.org/10.1038/377224a0

Coria RA, Currie PJ (2003) The braincase of *Giganotosaurus carolinii* (Dinosauria: Theropoda) from the Upper Cretaceous of Argentina. Journal of Vertebrate Paleontology 22: 802–811. https://doi.org/10.1671/0272-4634(2002)022[0802:TBOGCD]2.0.CO;2

Coria RA, Currie PJ (2006) A new carcharodontosaurid (Dinosauria, Theropoda) from the Upper Cretaceous of Argentina. Geodiversitas 28: 71–118.

Currie PJ (1996) Out of Africa: meat-eating dinosaurs that challenge *Tyrannosaurus rex*. Science 272: 971–972. https://doi.org/10.1126/science.272.5264.971

Currie PJ, Zhao XJ (1993) A new carnosaur (Dinosauria, Theropoda) from the Jurassic of Xinjiang, People's Republic of China. Canadian Journal of Earth Sciences 30: 2037–2081. https://doi.org/10.1139/e93-179

Currie PJ, Carpenter K (2000) A new specimen of *Acrocanthosaurus atokensis* (Theropoda, Dinosauria) from the Lower Cretaceous Antlers Formation (Lower Cretaceous, Aptian) of Oklahoma, USA. Geodiversitas 22: 207–246.

Dal Sasso C, Maganuco S (2011) *Scipionyx samniticus* (Theropoda: Compsognathidae) from the Lower Cretaceous of Italy: Osteology, ontogenetic assessment, phylogeny, soft tissue anatomy, taphonomy and palaeobiology. Memorie della Società Italiana di Scienze Naturali e del Museo Civico di Storia Naturale 37: 1–282.

Dal Sasso C, Maganuco S, Buffetaut E, Mendez MA (2005) New information on the skull of the enigmatic theropod *Spinosaurus*, with remarks on its size and affinities. Journal of Vertebrate Paleontology 25: 888–896. https://doi.org/10.1671/0272-4634(2005)025[0888:NIOTSO]2.0.CO;2

Delfaud J, Zellouf K (1995) Existence, durant le Jurassique et le Crétacé inférieur d'un paléo-Niger coulant du Sud vers le Nord au Sahara occidental. Bassins sedimentaires africains 118: 93–104.

Depéret C, Savornin J (1927) La faune de reptiles et de poissons albiens de Timimoun (Sahara algérien). Bulletin de la société géologique de France 27: 257–265.

Dominik W (1985) Stratigraphie und Sedimentologie (Geochemie, Schwermineralanalyse) der Oberkreide von Bahariya und ihre Korrelation zum Dakhla-Becken (Western Desert, Ägypten). Berliner Geowissenschaftliche Abhandlungen Reihe A Geologie und Palaeontologie 62: 1–173.

D'Orazi Porchetti S, Nicosia U, Biava A, Maganuco S (2011) New abelisaurid material from the Upper Cretaceous (Cenomanian) of Morocco. Rivista Italiana di Paleontologia e Stratigrafia 17: 463—472.

Dridi J (2018) New fossils of the giant pholidosaurid genus *Sarcosuchus* from the Early Cretaceous of Tunisia. Journal of African Earth Sciences 147: 268–280. https://doi.org/10.1016/j.jafrearsci.2018.06.023

Dubar G (1949) Carte géologique provisoire du Haut Atlas de Midelt, échelle au 1/200000eme, Notice explicative. Notes et Mémoires du Service Géologique du Maroc, 59bis, Rabat, 60 pp.

Duffin CJ (2001) Synopsis of the selachian genus *Lissodus* Brough, 1935. Neues Jahrbuch für Geologie und Paläontologie Abhandlungen 221: 145–218.

Dutheil DB (1996) Nouvelles découvertes d'élasmobranches dans le Crétacé supéieur de Sud marocain, implication stratigraphique. In: Vénec-Peyré M-T, Tassy P (Eds) Deuxième Congrès national de Paléontologie – Paléontologie, histoire récente et perspective à l'aube de l'an 2000, Paris, 13–15 novembre 1996, Paris, Résumé p. 28.

Dutheil DB (1999a) An overview of the freshwater fish fauna from the Kem Kem beds (Late Cretaceous: Cenomanian) of southeastern Morocco. In: Arratia G, Schultze H-P (Eds) Mesozoic Fishes 2 – Systematics and the Fossil Record. Verlag Dr. Friedrich Pfeil, Munich, 553–563.

Dutheil DB (1999b) The first articulated fossil cladistian, *Serenoichthys kemkemensis*, gen. et sp. nov., from the Cretaceous of Morocco. Journal of Vertebrate Paleontology 19: 243–246. https://doi.org/10.1080/02724634.1999.10011138

Dutheil DB (2001) New record of freshwater fish from the Cenomanian of Niger. Journal of Vertebrate Paleontology 21(3): 46A.

Dutheil DB, Brito PM (2009) Articulated cranium of *Onchopristis numidus* (Sclerorhynchidae, Elasmobranchii) from the Kem Kem beds, Morocco. First North African Vertebrate Palaeontology Meeting, Marrakech, Morocco, May 25–27, 2009, Abstract.

Dutheil DB, Moreau F, Brito PM (2001) Articulated freshwater elasmobranchs from the Cenomanian of Morocco. In: Tintori A (Ed), Mesozoic Fishes 3 – Systematics, Paleoenvironnements and Biodiversity. Serpiano, August 26–31, 2001, Abstract.

Dyke GJ (2010) Palaeoecology: Different dinosaur ecologies in deep time? Current Biology 20: 983–985. https://doi.org/10.1016/j.cub.2010.10.001

Eberth D, Currie PJ (2010) Stratigraphy, sedimentology, and taphonomy of the Albertosaurus bonebed (upper Horseshoe Canyon Formation; Maastrichtian), southern Alberta, Canada. Canadian Journal of Earth Sciences 47: 1119–1143. https://doi.org/10.1139/E10-045

Essafraoui B, Ferry S, Groshény D, Içame N, El Aouli H, Masrour M, Bulot LG, Géraud Y, Aoutem M (2015) Sequence stratigraphic architecture of marine to fluvial deposits across a passive margin (Cenomanian, Atlantic margin, Morocco, Agadir transect). Carnets de Geologie-Notebooks on Geology 15: 137–172. https://doi.org/10.4267/2042/56909

Ettachfini EM, Andreu B (2004) Le Cénomanien et le Turonien de la plate-forme Préafricaine du Maroc. Cretaceous Research 25: 277–302. https://doi.org/10.1016/j.cretres.2004.01.001

Ettachfini EM, Souhel A, Andreu B, Caron M (2005) La limite Cénomanien-Turonien dans le Haut Atlas central, Maroc. Geobios: 38: 57–68. https://doi.org/10.1016/j.geobios.2003.07.003

Evans SE, Milner AR, Werner C (1996) Sirenid salamanders and a gymnophionan amphibian from the Cretaceous of the Sudan. Palaeontology 39: 77–95.

Evans SE, Prasad GVR, Manhas BK (2002) Fossil lizards from the Jurassic Kota Formation of India. Journal of Vertebrate Paleontology 22: 299–312. https://doi.org/10.1671/0272-4634(2002)022[0299:FLFTJK]2.0.CO;2

Evers SW, Rauhut OW, Milner AC, McFeeters B, Allain R (2015) A reappraisal of the morphology and systematic position of the theropod dinosaur *Sigilmassasaurus* from the "middle" Cretaceous of Morocco. PeerJ 3: e1323. https://doi.org/10.7717/peerj.1323

Falcon-Lang HJ, Cantrill DJ, Nichols GJ (2001) Biodiversity and terrestrial ecology of a mid-Cretaceous high-latitude floodplain, Alexander Island, Antarctica. Journal of the Geological Society London 158: 709–724. https://doi.org/10.1144/jgs.158.4.709

Fanti F, Contessi M, Franchi F (2012) The "Continental Intercalaire" of southern Tunisia: Stratigraphy, paleontology, and paleoecology. Journal of African Earth Sciences 73–74: 1–23. https://doi.org/10.1016/j.jafrearsci.2012.07.006

Fanti F, Cau A, Hassine M (2014) Evidence of titanosauriforms and rebbachisaurids (Dinosauria: Sauropoda) from the Early Cretaceous of Tunisia. Journal of African Earth Sciences 90: 1–8. https://doi.org/10.1016/j.jafrearsci.2013.10.010

Fanti F, Cau A, Cantelli L, Hassine M, Auditore M (2015) New information on *Tataouinea hannibalis* from the Early Creteacous of Tunisia and implications for the tempo and mode of rebbachisaurid sauropod evolution. PLoS ONE 10(4): e0123475. https://doi.org/10.1371/journal.pone.0123475

Ferrandini M (1988) Les foraminifères benthiques du Cénomanien supérieur dans le sud-est marocain. Revue de Paléobiologie, Volume Spécial Benthos 86, 2: 429–430.

Ferrandini M, Philip J, Babinot J-F, Ferrandini J, Tronchettti G (1985) La plate-forme carbonatée du Cénomano-Turonien de la région d'Erfoud-Errachidia (Sud-Est marocain): stratigraphie et paléoenvironnements. Bullettin de la Société Géologique de France 8: 559–564. https://doi.org/10.2113/gssgfbull.I.4.559

Filleul A, Dutheil DB (2001) *Spinocaudichthys oumtkoutensis* a freshwater acanthomorph from the Cenomanian Morocco. Journal of Vertebrate Paleontology 21: 774–780. https://doi.org/10.1671/0272-4634(2001)021[0774:SOAFAF]2.0.CO;2

Filleul A, Dutheil DB (2004) A peculiar actinopterygian fish from Cretaceous of Morocco. Journal of Vertebrate Paleontology 24: 290–298. https://doi.org/10.1671/3004

Fiorelli LE, Leardi JM, Hechenleitner EM, Pol D, Basilici G, Grellet-Tinner G (2016) A new Late Cretaceous crocodyliform from the western margin of Gondwana (La Rioja Province, Argentina). Cretaceous Research 60: 194–209. https://doi.org/10.1016/j.cretres.2015.12.003

Fischer AG (1982) Long-term climatic oscillations recorded in stratigraphy. In: Berger WH, Crowell JC (Eds) Climate in Earth history. National Academy Press, Washington D.C., 97–104.

Forey PL (1997) A Cretaceous notopterid (Pisces: Osteoglossomorpha) from Morocco. South African Journal of Science 93: 564–569.

Forey PL, Grande L (1998) An African twin to the Brazilian *Calamopleurus* (Actinopterygii: Amiidae). Zoological Journal of the Linnean Society 123: 179–195. https://doi.org/10.1111/j.1096-3642.1998.tb01299.x

Forey PL, Cavin L (2007) A new species of *Cladocyclus* (Teleostei: Ichthyodectiformes) from the Cenomanian of Morocco. Palaeontologia Electronica 10. http://palaeolectronica.org/paleo/2007_3/133/index.html

Franzosa J, Rowe T (2005) Cranial endocast of the Cretaceous theropod dinosaur *Acrocanthosaurus atokensis*. Journal of Vertebrate Paleontology 25: 859–864. https://doi.org/10.1671/0272-4634(2005)025[0859:CEOTCT]2.0.CO;2

Frey E, Martill DM, Buchy M-C (2003) A new crested ornithocheirid from the Lower Cretaceous of NE Brazil and the unusual death of an unusual pterosaur. In: Buffetaut E, Mazin

J-M (Eds) Evolution and Palaeobiology of Pterosaurs. Geological Society Special Publication 217: 55–63. https://doi.org/10.1144/GSL.SP.2003.217.01.05

Friedlander AM, Zgliczynski BJ, Ballesteros E, Aburto-Oropeza O, Bolaños A, Sala E (2012) The shallow-water fish assemblage of Isla del Coco National Park, Costa Rica: structure and patterns in an isolated, predator-dominated ecosystem. Revista de Biología Tropical 60: 321–338.

Gaffney ES, Tong H, Meylan AP (2002) *Galianemys*, a new side-necked turtle (Pelomedusoides: Bothremydidae) from the Late Cretaceous of Morocco. American Museum Novitates 3379: 1–20. https://doi.org/10.1206/0003-0082(2002)379<0001:GANSNT>2.0.CO;2

Gaffney ES, Tong H, Meylan PA (2006) Evolution of the side-necked turtles, the families Bothremydidae, Euraxemydidae, and Araripemydidae. Bulletin of the American Museum of Natural History 300: 1–698. https://doi.org/10.1206/0003-0090(2006)300[1:EOTSTT]2.0.CO;2

Gaffney ES, Meylan PA, Wood RC, Simons E, de Almeida Campos D (2011) Evolution of the side-necked turtles: The Family Podocnemididae. Bulletin of the American Museum of Natural History 350: 1–237. https://doi.org/10.1206/350.1

Gale AS (2000) The Cretaceous world. In: Culver SJ, Rawson PF (Eds) Biotic response to global change: the last 145 years. Cambridge University Press 2006: 4–19. https://doi.org/10.1017/CBO9780511535505.003

Garassino A, Pasini G, Dutheil DB (2006) *Cretapenaeus berberus* n. gen. n. sp. (Crustacea, Decapoda, Penaeidae) from the Late Cretaceous (Cenomanian) of southeastern Morocco. Atti della Società italiana di Scienza naturali e del Museo civico di Storia naturale, Milano 147: 3–17.

Garassino A, De Angelis A, Pasini G (2008) New decapod assemblage from the Upper Cretaceous (Cenomanian-Turonisn) of Gara Sbaa, southestern Morocco. Atti della Società italiana di Scienza naturali e del Museo civico di Storia naturale, Milano 149: 37–67.

Gauthier H (1960) Contribution à l'étude géologique des formations post-liasiques des bassins du Dadès et du Haut-Todra (Maroc méridional). Notes et Mémoires du Service géologique du Maroc 119: 1–212.

Gayet M, Meunier FJ (1991) First discovery of Polypteridae (Pisces, Cladistia) outside of Africa. Geobios 24: 463–466. https://doi.org/10.1016/S0016-6995(06)80246-9

Gayet M, Meunier FJ (1996) A propos du genre *Bartschia* Gayet & Meunier, 1996: rectification de nomenclature en *Bartschichthys* nov. gen. Cybium 20: 314.

Gayet M, Werner C (1997) Strange Polypteriformes from the Upper Cretaceous of In Becetem (Niger) and Wadi Milk Formation (Sudan). Geobios Mémoire spécial 20: 249–255. https://doi.org/10.1016/S0016-6995(97)80030-7

Gmira S (1995) Etudes des chéloniens fossiles du Maroc: anatomie, systématique, phylogénie. Cahiers de Paléontologie, CNRS Editions, Paris, 140 pp.

Gomani EM (1997) A crocodyliform from the Early Cretaceous dinosaur beds, northern Malawi. Journal of Vertebrate Paleontology 17: 280–294. https://doi.org/10.1080/02724634.1997.10010975

Grande L (2010) An empirical synthetic pattern study of gars (Lepisosteiformes) and closely related species, based mostly on skeletal anatomy. The resurrection of Holostei. American Society of Ichthyologists and Herpetologists, Special Publication 6: 1–871.

Guiraud R, Bosworth W, Thierry J, Delplanque A (2005) Phanerozoic geological evolution of Northern and Central Africa: An overview. Journal of African Earth Sciences 43: 83–143. https://doi.org/10.1016/j.jafrearsci.2005.07.017

Haddoumi H, Almeras Y, Bodergat AM, Charriere A, Mangold C, Benshili K (1998) Ages et environnements des Couches rouges d'Anoual (Jurassique moyen et Crétacé inférieur, Haut Atlas oriental, Maroc). Comptes Rendus de l'Académie des Sciences 327: 127–133. https://doi.org/10.1016/S1251-8050(98)80043-8

Haddoumi H, Allain R, Meslouh S, Metais G, Monbaron M, Pons D, Rage J-C, Vullo R, Zouhri S, Gheerbrant E (2016) Guelb el Ahmar (Bathonian, Anoual Syncline, eastern Morocco): first continental flora and fauna including mammals from the Middle Jurassic of Africa. Gondwana Research 29: 290–319. https://doi.org/10.1016/j.gr.2014.12.004

Hahn G, Hahn R (2003) New multituberculate teeth from the Early Cretaceous of Morocco. Acta Palaeontologica Polonica 48: 349–356.

Haq BU, Hardenbol J, Vail PR (1987) The new chronostratigraphic basis of Cenozoic and Mesozoic sea level cycles. Timing and depositional history of eustatic sequences: constraints on seismic stratigraphy. Cushman Foundation for Forminiferal Research, Special Publications 24: 7–13.

Harris JD (1998) A reanalysis of *Acrocanthosaurus atokensis*, its phylogenetic status, and paleobiogeographic implications, based on a new specimen from Texas. New Mexico Museum of Natural History and Science, Bulletin 13: 1–71.

Hassler A, Martin JE, Amiot R, Tacail T, Godet FA, Allain R, Balter V (2018) Calcium isotopes offer clues on resource partitioning among Cretaceous predatory dinosaurs. Proceedings of the Royal Society B 285: e 20180197. https://doi.org/10.1098/rspb.2018.0197

Haug E (1904) Sur la faune des couches à *Ceratodus* crétacées du Djoua, près Timassânine (Sahara) Comptes Rendus de l'Académie des Sciences 138: 1529–1531. https://doi.org/10.5962/bhl.part.24288

Haug E (1905) Paléontologie. Documents scientifiques de la mission saharienne (mission Foureau-Lamy). Publication de la Société de Géographie Paris 751–832.

Heinrich W-D (1998) Late Jurassic mammals from Tendaguru, Tanzania, East Africa. Journal of Mammalian Evolution 5: 269–290. https://doi.org/10.1023/A:1020548010203

Henderson DM (1998) Skull and tooth morphology as indicators of niche partitioning in sympatric Morrison Formation theropods. Gaia 15: 219–226.

Hendrickx C, Mateus O, Buffetaut E (2016) Morphofunctional analysis of the quadrate of Spinosauridae (Dinosauria: Theropoda) and the presence of *Spinosaurus* and a second spinosaurine taxon in the Cenomanian of North Africa. PLoS ONE 11(1): e0144695. https://doi.org/10.1371/journal.pone.0144695

Herman AB, Spicer RA (1996) Palaeobotanical evidence for a warm Cretaceous Arctic Ocean. Nature 380: 330–333. https://doi.org/10.1038/380330a0

Hocknull SA, White MA, Tischler TR, Cook AG, Calleja ND, Sloan T, Elliott DA (2009) New mid-Cretaceous (latest Albian) dinosaurs from Winton, Queensland, Australia. PLoS ONE 4(7): e6190. https://doi.org/10.1371/journal.pone.0006190

Holliday CM, Gardner NM (2012) A new eusuchian crocodyliform with novel cranial integument and its significance for the origin and evolution of Crocodylia. PLoS ONE 7(1): e30471. https://doi.org/10.1371/journal.pone.0030471

Holwerda FM, Díaz Díaz V, Blanco A, Montie R, Reumer JWF (2018) Late Cretaceous sauropod tooth morphotypes may provide supporting evidence for faunal connections between North Africa and Southern Europe. PeerJ 6: e5925. https://doi.org/10.7717/peerj.5925

Holz M (2015) Mesozoic paleogeography and paleoclimates – a discussion of the diverse greenhouse and hothouse conditions of an alien world. Journal of South American Earth Sciences 61: 91–107. https://doi.org/10.1016/j.jsames.2015.01.001

Hone DHE, Habib MB, Therrien F (2019) *Cryodrakon boreas*, gen. et sp. nov., a Late Cretaceous Canadian azhdarchid pterosaur. Journal of Vertebrate Paleontology 39(3): e1649681. https://doi.org/10.1080/02724634.2019.1649681

Horner JR, Goodwin MB, Myhrvold N (2011) Dinosaur census reveals abundant *Tyrannosaurus* and rare ontogenetic stages in the Upper Cretaceous Hell Creek Formation (Maastrichtian), Montana, USA. PLoS ONE 6(2): e16574. https://doi.org/10.1371/journal.pone.0016574

Howse SCB (1986) On the cervical vertebrae of the Pterodactyloidea (Reptilia: Archosauria). Zoological Journal of the Linnean Society 88: 307–328. https://doi.org/10.1111/j.1096-3642.1986.tb02249.x

Hsiang AY, Field DJ, Webster TH, Behlke ADB, Davis MB, Racicot RA, Gauthier JA (2015) The origin of snakes: revealing the ecology, behavior, and evolutionary history of the early snakes using genomic, phenomics, and the fossil record. BMC Evolutionary Biology 15: 87–109. https://doi.org/10.1186/s12862-015-0358-5

Hu X-M, Wagreich M, Sames B (2017) Special topic: Cretaceous greenhouse palaeoclimate and sea-level changes. Science China Earth Sciences 60: 1–4. https://doi.org/10.1007/s11430-016-0278-3

Ibrahim N, Sereno PC, Zouhri S (2017) Les dinosaures du Maroc – aperçu historique et travaux récents. In: Zouhri S (Ed) Paléontologie des vertébrés du Maroc: état des connaissances. Mémoires de la Societé géologique de France 2017: 249–284.

Ibrahim N, Unwin DM, Martill DM, Baidder L, Zouhri S (2010) A new pterosaur (Pterodactyloidea: Azhdarchidae) from the Upper Cretaceous of Morocco. PLoS ONE 5(5): e10875. https://doi.org/10.1371/journal.pone.0010875

Ibrahim N, Dal Sasso C, Maganuco S, Fabbri M, Martill DM, Gorscak E, Lamanna MC (2016) Evidence of a derived titanosaurian (Dinosauria, Sauropoda) in the 'Kem Kem beds' of Morocco, with comments on sauropod paleoecology in the Cretaceous of Africa. In: Khosla A, Lucas S (Eds) Recent advances in Cretaceous biodiversity, paleoenvironments and palaeobiogeographic implications. New Mexico Museum of Natural History and Science Bulletin 71: 149–159.

Ibrahim N, Varricchio DJ, Sereno PC, Wilson JA, Dutheil DB, Martill DM, Baidder L, Zouhri S (2014a) Dinosaur footprints and other ichnofauna from the Cretaceous Kem Kem beds of Morocco. PLoS ONE 9(3): e90751. https://doi.org/10.1371/journal.pone.0090751

Ibrahim N, Sereno PC, Dal Sasso C, Maganuco S, Fabbri M, Martill DM, Zouhri S, Myhrvold N, Iurino DA (2014b). Semiaquatic adaptations in a giant predatory dinosaur. Science 345: 1613–1616. https://doi.org/10.1126/science.1258750

ICZN [International Commission of Zoological Nomenclature] (1999) International Code of Zoological Nomenclature, 4th ed. International Trust for Zoological Nomenclature, London, United Kingdom, 306 pp.

Ijouiher J (2016) A reconstruction of the paleoecology and environmental dynamics of the Bahariya Formation of Egypt. PeerJ Preprints 4: e2470v1. https://doi.org/10.7287/peerj.preprints.2470v1

Jacobs, ML, Martill DM, Ibrahim N, Longrich N (2019) A new species of *Coloborhynchus* (Pterosauria, Ornithocheiridae) from the mid-Cretaceous of North Africa. Cretaceous Research 95: 77–88. https://doi.org/10.1016/j.cretres.2018.10.018

Jacobs ML, Martill DM, Unwin DM, Ibrahim N, Zouhri S, Longrich N (2020) New toothed pterosaurs (Pterosauria: Ornithocheiridae) from the middle Cretaceous Kem Kem beds of Morocco and implications for pterosaur palaeobiogeography and diversity. Cretaceous Research 110: e104413. https://doi.org/10.1016/j.cretres.2020.104413

Jain SL, Bandyopadhyay S (1997) New titanosaurid (Dinosauria: Sauropoda) from the Late Cretaceous of central India. Journal of Vertebrate Paleontology 17: 114–136. https://doi.org/10.1080/02724634.1997.10010958

Joly F (1962) Etudes sur le relief du Sud-Est marocain. Travaux de l'Institut Scientifique Chérifien Série Géologie et Géographie Physique 10: 1–578.

Karl H-V (2010) Turtle shell remains (Testudines: Bothremydidae) from the Cenomanian of Morocco. Studia Geologica Salmanticensia 46: 47–54.

Kellner AWA (1989) A new edentate pterosaur of the Lower Cretaceous from the Araripe Basin, Northeast Brazil. Anais da Academia Brasileira de Ciências 61: 439–446.

Kellner AWA, Langston W (1996) Cranial remains of *Quetzalcoatlus* (Pterosauria, Azhdarchidae) from Late Cretaceous sediments of Big Bend National Park. Journal of Vertebrate Paleontology 16: 222–231. https://doi.org/10.1080/02724634.1996.10011310

Kellner AWA, Mader BJ (1996) First report of Pterosauria (Pterodactyloidea, Azhdarchidae) from Cretaceous rocks of Morocco. Journal of Vertebrate Paleontology, Program and Abstracts 16(3): 45A. https://doi.org/10.1080/02724634.1996.10011310

Kellner AWA, Mader BJ (1997) Archosaur teeth from the Cretaceous of Morocco. Journal of Paleontology 7: 525–527. https://doi.org/10.1017/S0022336000039548

Kellner AWA, Tomida Y (2000) Description of a new species of Anhangueridae (Pterodactyloidea) with comments on the pterosaur fauna from the Santana Formation (Aptian-Albian), northeastern Brazil. National Science Museum Monographs Tokyo 17: 1–135.

Kellner AWA, Mello AMS, Ford T (2007) A survey of pterosaurs from Africa with the description of a new specimen from Morocco. Paleontologia: Cenarios de Vida 2007: 257–267.

Kidder DL, Worsley TR (2010) Phanerozoic large igneous provinces (LIPs), HEATT (haline euxinic acidic thermal transgression) episodes, and mass extinctions. Palaeogeography, Palaeoclimatology, Palaeoecology 295: 162–191. https://doi.org/10.1016/j.palaeo.2010.05.036

Kidder DL, Worsley TR (2012) A human-induced hothouse climate. Geological Society of America Today 22: 4–11. https://doi.org/10.1130/G131A.1

Kidwell SM, Holland SM (2002) Quality of the fossil record: implications for evolutionary biology. Annual Review of Ecology and Systematics 33: 561–588. https://doi.org/10.1146/annurev.ecolsys.33.030602.152151

Kilian C (1931) Des principaux complexes continentaux du Sahara. Comptes Rendus sommaires de la Société géologique de France 9: 109–111.

Kilian C (1937) Esquisse géologique du Sahara français à l'est du 6ème degré de longitude. Chronique des Mines et de la Colonization 58: 21–22.

Klein CG, Longrich NR, Ibrahim N, Zouhri S, Martill DM (2017) A new basal snake from the mid-Cretaceous of Morocco. Cretaceous Research 72: 134–141. https://doi.org/10.1016/j.cretres.2016.12.001

Knoll F, Ruiz-Omeñaca JI (2009) Theropod teeth from the basalmost Cretaceous of Anoual (Morocco) and their palaeobiogeographical significance. Geological Magazine 146: 602–616. https://doi.org/10.1017/S0016756809005950

Kuhnt W, Holbourn A, Gale A, Chellai EH, Kennedy WJ (2009) Cenomanian sequence stratigraphy and sea-level fluctuations in the Tarfaya Basin (SW Morocco). Geological Society of America Bulletin 121: 1695–1710. https://doi.org/10.1130/B26418.1

Lacovara, KJ, Smith JR, Smith JB, Lamanna MC (2003) The Ten Thousand Islands Coast of Florida: a modern analog to low-energy mangrove coasts of Cretaceous epeiric seas. In: Davis Jr. RA (Ed). Proceedings of the 5th International Conference on Coastal Sediments, Clearwater Beach, Florida 5: 1773–1784.

Lamanna MC, Martínez RD, Smith JB (2002) A definitive abelisaurid theropod dinosaur from the Early Cretaceous of Patagonia. Journal of Vertebrate Paleontology 22: 58–69. https://doi.org/10.1671/0272-4634(2002)022[0058:ADATDF]2.0.CO;2

Lamsdell JC, Tashman JN, Pasini G, Garassino A. (2020) A new limulid (Chelicerata, Xiphosurida) from the Late Cretaceous (Cenomanian-Turonian) of Gara Sbaa, southeast Morocco. Cretaceous Research 106: e104230. https://doi.org/10.1016/j.cretres.2019.104230

Läng E, Boudad L, Maio L, Samankassou E, Tabouelle J, Tong H, Cavin L (2013) Unbalanced food web in a Late Cretaceous dinosaur assemblage. Palaeogeography, Palaeoclimatology, Palaeoecology 381: 26–32. https://doi.org/10.1016/j.palaeo.2013.04.011

Lapparent AF (1949) Sur la stratigraphie et la paléontologie du Djoua (Sahara central). Comptes Rendus de l'Académie des Sciences à Paris 228: 1040–1042.

Lapparent AF (1951) Découverte de dinosauriens, associés à une faune de reptiles et de poissons, dans le Crétacé inférieur de l'extrême sud tunisien. Comptes Rendus de l'Académie des Sciences de Paris 232: 1430–1432.

Lapparent AF (1953) Reconnaissance de gisements à dinosauriens du Tamesna (Sahara méridional). Travail de l'Institut de Recherche saharienne 10: 7–17.

Lapparent AF (1958) Sur les dinosaures du 'continental intercalaire' du Sahara central. Comptes Rendus de l'Académie des Sciences de Paris 246: 1237–1240.

Lapparent, AF (1960) Les dinosauriens du "continental intercalaire" du Sahara central. Mémoires de la Société Géologique de France (N. S.) 88A: 1–57.

Lapparent AF, Lelubre M (1948) Interprétation stratigraphique des séries continentales entre Ohanet et Bourarhet (Sahara Central). Comptes Rendus de l'Académie des Sciences de Paris 227: 1106–1108.

Lapparent de Broin F de (2002) *Elosuchus*, a new genus of crocodile from the Lower Cretaceous of the North of Africa, Comptes Rendus Palevol 1: 275–285. https://doi.org/10.1016/S1631-0683(02)00049-0

Larsson HCE (2001) Endocranial anatomy of *Carcharodontosaurus saharicus* (Theropoda: Allosauroidea) and its implications for theropod brain evolution. In: Tanke DH, Carpenter K (Eds) Mesozoic Vertebrate Life. Indiana University Press, Bloomington, 19–33.

Larsson HCE, Sereno PC, Wilson JA (2000) Forebrain enlargement among nonavian theropod dinosaurs. Journal of Vertebrate Paleontology 20: 615–618. https://doi.org/10.1671/0272-4634(2000)020[0615:FEANTD]2.0.CO;2

Larsson HCE, Sidor CA (1999) Unusual crocodylomorph teeth from the Late Cretaceous (Cenomanian) of Southeastern Morocco. Journal of Vertebrate Paleontology 19: 398–401. https://doi.org/10.1080/02724634.1999.10011152

Larsson HCE, Sues H-D (2007) Cranial osteology and phylogenetic relationships of *Hamadasuchus rebouli* (Crocodyliformes: Mesoeucrocodylia) from the Cretaceous of Morocco. Zoological Journal of the Linnean Society 149: 533–567. https://doi.org/10.1111/j.1096-3642.2007.00271.x

Larsson HCE, Sereno PC, Evans D (2015) New giant Late Cretaceous crocodyliform with feeding adaptations convergent on spinosaurids. Journal of Vertebrate Paleontology e-Supplement 35: 161A.

Lasseron, M., Allain R, Gheerbrant E, Haddoumi H, Jalil N-E, Métais G, Rage J-C, Vullo R, Zouhri S (2019) New data on the microvertebrate fauna from the Upper Jurassic or lowest Cretaceous of Ksar Metlili (Anoual Syncline, eastern Morocco). Geological Magazine 157: 1–26. https://doi.org/10.1017/S0016756819000761

Lavocat R (1948) Découverte de Crétacé à vértebrés dans le soubassement de la Hammada du Guir (Sud marocain). Comptes Rendus de l'Académie des Sciences de Paris 226: 1291–1292.

Lavocat R (1949) Les gisements de vertébrés crétacés du Sud marocain. Compte rendu sommaire des Séances de la Société géologique de France 7: 125–126.

Lavocat R (1951) Découvertes de restes d'un grand dinosaurien sauropode dans le Crétacé du Sud marocain. Comptes rendus de l'Académie des Sciences de Paris 232: 169–170.

Lavocat R (1952) Les gisements de dinosauriens du Crétacé du sud marocain. Compte rendu sommaire des séances de la Société Géologique de France 1–2: 12–13.

Lavocat R (1954a) Reconnaissance géologique dans les hammadas des confins algéro-marocains du sud. Notes et Mémoires du Service géologiques (Maroc) 116: 1–147.

Lavocat R (1954b) Sur les dinosaures du Continental intercalaire des Kem-Kem de la Daoura. In: 19ème Congrès géologique International, Alger 1952, Comptes Rendus session XII-3, 15: 65–68.

Lavocat R (1955) Découverte d'un crocodilien du genre *Thoracosaurus* dans le Crétacé supérieur d'Afrique. Bulletin du Muséum national d'Histoire Naturelle de Paris 27: 338–340.

Leardi JM, Pol D, Novas FE, Riglos MS (2015) The postcranial anatomy of *Yacarerani boliviensis* and the phylogenetic significance of the notosuchian postcranial skeleton. Journal of Vertebrate Paleontology 35(6) 35(6): e995187. https://doi.org/10.1080/02724634.2014.995187

Lee YN (1994) The Early Cretaceous pterodactyloid pterosaur *Coloborhynchus* from North America. Palaeontology 37: 755–763.

Lefranc J-P (1958) Stratigraphie des séries continentales intercalaires au Fezzan nord-occidental (Libye). Comptes rendus de Académie des Sciences Paris 247: 1360–1363.

Lefranc J-P (1963) Morphologie, stratigraphie, struture et paleogéographie des formations continentales intercalaires du Fezzan nord-occidental (Libye). PhD Thesis, University of Paris, Paris.

Lefranc J-P, Guiraud R (1990) The Continental intercalaire of northwestern Sahara and its equivalents in the neighbouring regions. Journal of African Earth Sciences 10: 27–77. https://doi.org/10.1016/0899-5362(90)90047-I

Leidy J (1870) Remarks on ichthyodorulites and on certain fossil Mammalia. Proceedings of the Academy of Natural Sciences of Philadelphia 22: 12–13.

Lio G, Agnolin F, Cau A, Maganuco S (2012) Crocodyliform affinities for *Kemkemia auditorei* Cau & Maganuco, 2009 from the Late Cretaceous of Morocco. Atti della Società Italiana di Scienze Naturali e del Museo Civico di Storia Naturale di Milano 153: 119–126. https://doi.org/10.4081/nhs.2012.119

Longrich NR, Martill DM, Andres B (2018) Late Maastrichtian pterosaurs from North Africa and mass extinction of Pterosauria at the Cretaceous-Paleogene boundary. PLOS Biology 16(4): e1002627. https://doi.org/10.1371/journal.pbio.1002627

Longrich NR, Pereda-Suberbiola P, Jalil N-E, Khaldoune F, Jourani E (2017) An abelisaurid from the latest Cretaceous (late Maastrichtian) of Morocco, North Africa. Cretaceous Research 76: 40–52. https://doi.org/10.1016/j.cretres.2017.03.021

Lü JC, Unwin DM, Xu L, Zhang X (2008) A new azhdarchoid pterosaur from the Lower Cretaceous of China and its implications for pterosaur phylogeny and evolution. Naturwissenschaften 95(9): 891–897. https://doi.org/10.1007/s00114-008-0397-5

Lü J, Liu J, Wang X, Gao C, Meng Q, Ji Q (2006) New material of pterosaur *Sinopterus* (Reptilia: Pterosauria) from the Early Cretaceous Jiufotang Formation, western Liaoning, China. Acta Geologica Sinica 80: 783–789. https://doi.org/10.1111/j.1755-6724.2006.tb00302.x

Mader BJ, Kellner AWA (1997) First occurrence of Anhangueridae (Pterosauria, Pterodactyloidea) in Africa. Journal of Vertebrate Paleontology, Program and Abstracts 17(3): 62A.

Mader BJ, Kellner AWA (1999) A new Anhanguerid pterosaur from the Cretaceous of Morocco. Boletim do Museu Nacional. Geologia 45: 1–11.

Madsen JH (1976) *Allosaurus fragilis*: a revised osteology. Utah Geological Survey Bulletin 109: 1–163.

Mahler L (2005) Record of Abelisauridae (Dinosauria: Theropoda) from the Cenomanian of Morocco. Journal of Vertebrate Paleontology 25: 236–239. https://doi.org/10.1671/0272-4634(2005)025[0236:ROADTF]2.0.CO;2

Maisey JG (1986) Coelacanths from the Lower Cretaceous of Brazil. American Musem Novitates 2866: 1–30.

Maisey JG (1987) Cranial anatomy of the Lower Jurassic shark *Hybodus reticulatus* (Chondrichthyes: Elasmobranchii), with comments on hybodontid systematics. American Museun Novitates 2878: 1–39.

Mannion PD, Barrett PM (2013) Additions to the sauropod dinosaur fauna of the Cenomanian (early Late Cretaceous) Kem Kem beds of Morocco: Paleobiogeographical implications of the mid-Cretaceous African sauropod fossil record. Cretaceous Research 45: 49–59. https://doi.org/10.1016/j.cretres.2013.07.007

Martill DM, Ibrahim N (2012) Aberrant rostral teeth of the sawfish *Onchopristis numidus* from the Kem Kem beds (? early Late Cretaceous) of Morocco and a reappraisal of *Onchopristis* in New Zealand. Journal of African Earth Sciences 64: 71–76. https://doi.org/10.1016/j.jafrearsci.2011.11.009

Martill DM, Unwin DM (2012) The world's largest toothed pterosaur, NHMUK R481, an incomplete rostrum of *Coloborhynchus capito* (Seeley, 1870) from the Cambridge Greensand of England. Cretaceous Research 34: 1–9. https://doi.org/10.1016/j.cretres.2011.09.003

Martill DM, Ibrahim N (2015) An unusual modification of the jaws in cf. *Alanqa*, a mid-Cretaceous azhdarchid pterosaur from the Kem Kem beds of Morocco. Cretaceous Research 53: 59–67. https://doi.org/10.1016/j.cretres.2014.11.001

Martill DM, Sweetman SC, Witton MP (2011b) Pterosaurs. In: Batten DJ (Ed.) English Wealden fossils. Field Guides to Fossils. Palaeontological Association, London 14: 370–390.

Martill DM, Tischlinger H, Longrich NR (2015) A four-legged snake form the Early Cretaceous of Gondwana. Science 349: 416–419. https://doi.org/10.1126/science.aaa9208

Martill DM, Unwin DM, Ibrahim N, Longrich N (2018) A new edentulous pterosaur form the Cretaceous Kem Kem beds of south eastern Morocco. Cretaceous Research 84: 1–12. https://doi.org/10.1016/j.cretres.2017.09.006

Martill DM, Smith R, Unwin DM, Kao A, McPhee J, Ibrahim N (2020) A new tapejarid (Pterosauria, Azhdarchoidea) from the mid-Cretaceous Kem Kem beds of Takmout, southern Morocco. Cretaceous Research. https://doi.org/10.1016/j.cretres.2020.104424

Martill DM, Ibrahim N, Brito PM, Baidder L, Zouhri S, Loveridge R, Naish D, Hing R (2011a) A new Plattenkalk Konservat Lagerstätte in the Upper Cretaceous of Gara Sbaa, south-eastern Morocco. Cretaceous Research 32: 433–446. https://doi.org/10.1016/j.cretres.2011.01.005

Martin M (1982) Nouvelles données sur la phylogénie et la systématique des dipneustes postpaléozoïques. Comptes Rendues de l'Academie des Sciences, Paris 294: 611–614. https://doi.org/10.1016/S0016-6995(82)80102-2

Martin M (1984) Révision des Argonodontidés et des Néoceratodontidés (Dipnoi, Ceratodontiformes) du Crétacé africain. Neues Jahrbuch für Geologie und Paläontologie, Abhandlungen 169: 225–260.

Martin JE, Lapparent de Broin F de (2016) A miniature notosuchian with multicuspid teeth from the Cretaceous of Morocco. Journal of Vertebrate Paleontology 36(6): e1211534. https://doi.org/10.1080/02724634.2016.1211534

Martinelli AG, Bogan S, Agnolin FL, Ribeiro LC, Cavellani CL, Ferraz ML, Teixeira VP (2013) First fossil record of amiid fishes (Halecomorphi, Amiiformes, Amiidae) from the late Cretaceous of Uberaba, Minas Gerais state, Brazil. Alcheringa: an Australasian Journal of Palaeontology 37: 105–113. https://doi.org/10.1080/03115518.2012.709444

McFeeters B, Ryan MJ, Hinic-Frlog S, Schröder-Adams C (2013) A reevaluation of *Sigilmassasaurus brevicollis* (Dinosauria) from the Cretaceous of Morocco. Canadian Journal of Earth Sciences 50: 636–649. https://doi.org/10.1139/cjes-2012-0129

McGowan AJ, Dyke GJ (2009) A surfeit of theropods in the Moroccan Late Cretaceous? Comparing diversity estimates from field data and fossil shops. Geology 37: 843–846. https://doi.org/10.1130/G30188A.1

McNulty Jr CL, Slaughter BH (1962) A new sawfish from the Woodbine Formation (Cretaceous) of Texas. Copeia 4: 775–777. https://doi.org/10.2307/1440678

McPhee J, Ibrahim N, Kao A, Unwin DM, Smith R, Martill DM (2020) A new ? chaoyangopterid (Pterosauria: Pterodactyloidea) from the Cretaceous Kem Kem beds of Southern Morocco. Cretaceous Research 110: 104410. https://doi.org/10.1016/j.cretres.2020.104410

Meunier L, Larsson HCE (2017) Revision of the phylogenetic affinities of *Elosuchus* (Crocodyliformes). Zoological Journal of the Linnean Society 179: 169–200. https://doi.org/10.1111/zoj.12448

Meunier FJ, Dutheil D, Brito PM (2013) Histological study of the median lingual dental plate of the Cretaceous fish †*Palaeonotopterus greenwoodi* (Teleostei: Osteoglossomorpha) from the Kem-Kem beds, Morocco. Cybium 37: 121–125.

Meunier FJ, Eustache R-P, Dutheil D, Cavin L (2016) Histology of ganoid scales from the early Late Cretaceous of the Kem Kem beds, SE Morocco: systematic and evolutionary implications. Cybium 40: 121–132.

Milner AC (2003) Fish-eating theropods, a short review of the systematics, biology and palaeobiogeography of spinosaurs. In: Huerta Hurtado P, Torcida Fernandez-Baldor F (Eds) Actas de las II Jornadas Internacionales sobre Paleontología de Dinosaurios y su Entorno (2001). Colectivo Arqueologico-Paleontologico de Salas, Salas de Los Infantes, 129–138.

Montefeltro FC, Laurini CR, Langer MC (2009) Multicusped crocodyliform teeth from the Upper Cretaceous (São José do Rio Preto Formation, Bauru Group) of São Paulo, Brazil. Cretaceous Research 30: 1279–1286. https://doi.org/10.1016/j.cretres.2009.07.003

Mudroch A, Richter U, Joger U, Kosma R, Idé O, Maga A (2011) Didactyl tracks of paravian theropods (Maniraptora) from the ? Middle Jurassic of Africa. PLoS ONE 6(2): e14642. https://doi.org/10.1371/journal.pone.0014642

Myers TS (2010) A new ornithocheirid pterosaur from the Upper Cretaceous (Cenomanian-Turonian) Eagle Ford Group of Texas. Journal of Vertebrate Paleontology 30: 280–287. https://doi.org/10.1080/02724630903413099

Nessov LA (1984) Pterosaurs and birds from the Late Cretaceous of central Asia. Paleontologicheskii Zhurnal 1: 38–49.

Nessov LA, Zhegallo VI, Averianov AO (1998) A new locality of Late Cretaceous snakes, mammals, and other vertebrates in Africa (western Libya). Annales de Paléontologie 84: 265–274. https://doi.org/10.1016/S0753-3969(99)80002-0

Nobre PH, Carvalho I de S (2006) *Adamantinasuchus navae*: A new Gondwanan Crocodylomorpha (Mesoeucrocodylia) from the Late Cretaceous of Brazil. Gondwana Research 10: 370–378. https://doi.org/10.1016/j.gr.2006.05.008

North American Commission on Stratigraphic Nomenclature (2005) North American Stratigraphic Code. American Association of Petroleum Geologists, Bulletin 89: 1547–1591. https://doi.org/10.1306/07050504129

Nothdurft W, Smith JB, Lamanna MC, Lacovara KJ, Poole JC, Smith JR (2002) The Lost Dinosaurs of Egypt. Random House, New York, 256 pp.

Novas FE, Bandyopadhyay S (2001) Abelisaurid pedal unguals fromn the Late Cretaceous of India. Associación Paleontológica Argentina. Publicación Especial 7. VII International Symposium on Mesozoic Terrestrial Ecosystems, 145–149.

Novas FE, Dalla Vecchia F, Pais DF (2005a) Theropod pedal unguals from the Late Cretaceous (Cenomanian) of Morocco, Africa. Revista del Museo Argentino de Ciencias Naturales 7: 167–175. https://doi.org/10.22179/REVMACN.7.333

Novas FE, Valais S de, Vickers-Rich P, Rich T (2005b) A large Cretaceous theropod from Patagonia, Argentina, and the evolution of carcharodontosaurids. Naturwissenschaften 92: 226–230. https://doi.org/10.1007/s00114-005-0623-3

Novas FE, Pais DF, Pol D, Carvalho IDS, Scanferla A, Mones A, Riglos MS (2009) Bizarre notosuchian crocodyliform with associated eggs from the Upper Cretaceous of Bolivia. Journal of Vertebrate Paleontology 29: 1316–1320. https://doi.org/10.1671/039.029.0409

O'Connor PM, Sertich JJW, Stevens NJ, Roberts EM, Gottfried MD, Hieronymus TL, Jinnah ZA, Ridgely R, Ngasala SE, Temba J (2010) The evolution of mammal-like crocodyliforms in the Cretaceous period of Gondwana. Nature 466: 748–751. https://doi.org/10.1038/nature09061

Ogg JG, Ogg G, Gradstein FM (2016) A Concise Geologic Time Scale: 2016. Elsevier.

O'Leary MA, Roberts E, Head JJ, Bouaré M, Sissoko F (2004) A saltasaurid titanosaurian (Dinosauria: Sauropoda) from the "Continental Intercalaire" of Mali. Journal of Vertebrate Paleontology 24: 923–930. https://doi.org/10.1671/0272-4634(2004)024[0923:TDSRFT]2.0.CO;2

Ortega F, Escaso F, Sanz JL (2010) A bizarre, humped Carcharodontosauria (Theropoda) from the Lower Cretaceous of Spain. Nature 467: 203–206. https://doi.org/10.1038/nature09181

Ostrom JH (1969) Osteology of *Deinonychus antirrhopus*, an unusual theropod from the Lower Cretaceous of Montana. Bulletin of the Peabody Museum of Natural History 30: 1–165.

Ostrom JH, McIntosh JS (1966) Marsh's Dinosaurs: the Collections from Como Bluff. Yale University Press, New Haven, 388 pp.

Parente M, Frijia G, Di Lucia M, Jenkyns HC, Woodfine RG, Baroncini F (2008) Stepwise extinction of larger foraminifers at the Cenomanian-Turonian boundary: A shallow-water perspective on nutrient fluctuations during Oceanic Anoxic Event 2 (Bonarelli Event). Geology 36: 715–718. https://doi.org/10.1130/G24893A.1

Pereda-Suberbiola X, Bardet N, Jouve S, Iarochène M, Bouya B, Amaghzaz M (2003) A new azhdarchid pterosaur from the Late Cretaceous phosphates of Morocco. In: Buffetaut E, Mazin J-M (Eds) Evolution and Palaeobiology of Pterosaurs. Geological Society of London Special Publications, London, 80–90. https://doi.org/10.1144/GSL.SP.2003.217.01.08

Pérez-García A (2017) A new turtle taxon (Podocnemidoidea, Bothremydidae) reveals the oldest known dispersal event of the crown Pleurodira from Gondwana to Laurasia. Journal of Vertebrate Paleontology 15: 709–731. https://doi.org/10.1080/14772019.2016.1228549

Pérez-García A (2018) Identification of the Lower Cretaceous pleurodiran turtle *Taquetochelys decorata* as the only African araripemydid species. Comptes Rendus Palevol 18: 24–32. https://doi.org/10.1016/j.crpv.2018.04.004

Peyer B (1925) Ergebnisse der Forschungsreisen Prof. E. Stromers in den Wüsten Ägyptens. II. Wirbeltier-Reste der Baharje-Stufe (Unterstes Cenoman): 6. Die *Ceratodus*-Funde. Abhandlungen der Bayerischen Akademie Wissenschaften 30: 5–32.

Pittet F, Cavin L, Poyato-Ariza FJ (2010) A new teleostean fish from the early Late Cretaceous (Cenomanian) of SE Morocco, with a discussion of its relationships with ostariophysans. In: Grande T, Poyata-Ariza FJ, Diogo R (Eds), Gonorhynchiformes and Ostariophysan Relationships: A Comprehensive Review, Science Publishers, Enfield, 332–355. https://doi.org/10.1201/b10194-8

Pol D, Nascimento PM, Carvalho AB, Riccomini C, Pires-Domingues RA, Zaher H (2014) A new notosuchian from the Late Cretaceous of Brazil and the phylogeny of advanced notosuchians. PLoS ONE 9(4): e93105. https://doi.org/10.1371/journal.pone.0093105

Porfiri JD, Dos Santos D, Calvo JO (2007) New information on *Megaraptor namunhuaiquii* (Theropoda: Tetanurae), Patagonia: Considerations on paleoecological aspects. Arquivos do Museu Nacional, Rio de Janiero 65: 545–550.

Rage J-C, Werner C (1999) Mid-Cretaceous (Cenomanian) snakes from Wadi Abu Hashim, Sudan: the earliest snake assemblage. Palaeontologia Africana 35: 85–110.

Rage J-C, Escuillié F (2000) Un nouveau serpent bipède du Cénomanien (Crétacé). Implications phylétiques. Comptes Rendus de l'Académie des Sciences Paris 330: 1–8. https://doi.org/10.1016/S1251-8050(00)00146-4

Rage J-C, Cappetta H (2002) Vertebrates from the Cenomanian, and the geological age of the Draa Ubari fauna (Libya). Annales de Paléontologie 88: 79–84. https://doi.org/10.1016/S0753-3969(02)01040-6

Rage J-C, Dutheil DB (2008) Amphibians and squamates from the Cretaceous (Cenomanian) of Morocco. A preliminary study. Palaeontographica Americana 286: 1–22. https://doi.org/10.1127/pala/285/2008/1

Rhalmi M (2000) Le Cénomano-Turonien au Sud du Haut-Atlas Central marocain (Bassins de Ouarzazate et ErrachidiaBoudnib-Erfoud). Sédimentation, Stratigraphie et Diagenèse. PhD Thesis, Université Cadi-Ayyad, Marrakech, 220 p.

Rauhut OWM (1995) Zur systematischen Stellung der afrikanischen Theropoden *Carcharodontosaurus* Stromer 1931 und *Bahariasaurus* Stromer 1934. Berliner Geowissenschaften Abhlandlungen E 16: 357–375.

Rauhut OWM (1999) A dinosaur fauna from the Upper Cretaceous (Cenomanian) of northern Sudan. Palaeontologia Africana 35: 61–84.

Rauhut OWM (2003) The interrelationships and evolution of basal theropod dinosaurs. Special Papers in Palaeontology 69: 1–213.

Rauhut OWM (2009) Neue Reste von Archosauriern aus der "mittleren" Kreide von Marokko. Freunde der Bayerischen Staatssammlung für Paläontologie und Historische Geologie e.V. Jahresbericht und Mitteilungen 37: 40–45.

Rauhut OWM, Werner C (1995) First record of the family Dromaeosauridae (Dinosauria: Theropoda) in the Cretaceous of Gondwana (Wadi Milk Formation, northern Sudan). Paläontologische Zeitschrift 69: 475–489. https://doi.org/10.1007/BF02987808

Rauhut OWM, López-Arbarello A (2006) Wirbeltierreste aus der "mittleren" Kreide des Kem Kem, Marokko. Freunde der Bayerischen Staatssammlung für Paläontologie und Historische Geologie e.V. Jahresbericht und Mitteilungen 34: 41–45.

Reijers TJA, Petters SW, Nwajide CS (1997) The niger delta basin. In: Selley RC (Ed) Sedimentary Basins of the World, Elsevier Press 3: 151–172. https://doi.org/10.1016/S1874-5997(97)80010-X

Ridgwell N, Sereno PC (2010) A basal thyreophoran (Dinosauria, Ornithischia) from the Tiouraren Formation of Niger. Journal of Vertebrate Paleontology Program and Abstracts 2010: 150A–151A.

Riff D, Kellner AWA, Mader B, Russell D (2002) On the occurrence of an avian vertebra in Cretaceous strata of Morocco, Africa. Anais da Academia Brasileira Ciências 74: 367–368. https://doi.org/10.1590/S0001-37652002000200023

Roberts EM, Rogers RM, Foreman BZ (2007) Continental insect borings in dinosaur bone: examples from the Late Cretaceous of Madagascar and Utah. Journal of Paleontology 81: 201–208. https://doi.org/10.1666/0022-3360(2007)81[201:CIBIDB]2.0.CO;2

Rodrigues T, Kellner AWA (2008) Review of the pterodactyloid pterosaur *Coloborhynchus*. Zitteliana B 28: 219–228.

Rodrigues T, Kellner AWA, Mader BJ, Russell DA (2011) New pterosaur specimens from the Kem Kem beds (Upper Cretaceous, Cenomanian) of Morocco. Rivista Italiana di Paleontologia e Stratigrafia 117: 149–160.

Rogers RR (1993) Systematic patterns of time-averaging in the terrestrial vertebrate record: a Cretaceous case study. In: Kidwell SM, Behrensmeyer AK (Eds) Taphonomic Approaches to Time Resolution in Fossil Assemblages. Short Courses in Paleontology 6. Paleontological Society, Knoxville, 228–249. https://doi.org/10.1017/S2475263000001136

Russell DA (1996) Isolated dinosaur bones from the Middle Cretaceous of the Tafilalt, Morocco. Bulletin du Muséum national d'Histoire Naturelle de Paris C Sciences de la Terre 19: 349–402.

Russell DA, Paesler MA (2003) Environments of Mid-Cretaceous Saharan dinosaurs. Cretaceous Research 24: 569–588. https://doi.org/10.1016/S0195-6671(03)00072-7

Salgado L, Garrido A, Cocca SE, Cocca JR (2004) Lower Cretaceous rebbachisaurid sauropods from Cerro Aguada Del León, Neuquén Province, northwestern Patagonia, Argentina. Journal of Vertebrate Paleontology 24: 903–912. https://doi.org/10.1671/0272-4634(2004)024[0903:LCRSFC]2.0.CO;2

Schaal S (1984) Oberkretazische Osteichthyes (Knochenfische) aus dem Bereich von Bahariya und Kharge, Ägypten, und ihre Aussagen zur Paläokologie und Stratigraphie. Berliner Geowissenschaftliche Abhandlungen Reihe A 53: 1–76.

Schlüter T, Schwarzhans W (1978) Eine Bonebed-Lagerstätte aus dem Wealden Süd Tunesiens (Umgebung Ksar Krerachfa). Berliner Geowiss. Abhandlungen A 8: 53–65.

Schmidt KH (1992) The tectonic history of the Pre-Saharan depression (Morocco) – a geomorphological interpretation. Geolologische Rundschau 81: 211–219. https://doi.org/10.1007/BF01764550

Schwimmer DR, Stewart JD, Williams GD (1997) *Xiphactinus vetus* and the distribution of *Xiphactinus* species in the eastern United States. Journal of Vertebrate Paleontology 17: 610–615. https://doi.org/10.1080/02724634.1997.10011007

Scotese CR (2002) PALEOMAP (http://www.scotese.com)

Sereno PC, Wilson JA (2005) A sauropod tooth battery: structure and evolution. In: Curry Rogers KA, Wilson JA (Eds) The Sauropods: Evolution and Paleobiology. University of California Press, Berkeley, 157–177. https://doi.org/10.1525/california/9780520246232.003.0006

Sereno PC, Brusatte SL (2008) Basal abelisaurid and carcharodontosaurid theropods from the Lower Cretaceous Elrhaz Formation of Niger. Acta Palaeontologica Polonica 53: 15–46. https://doi.org/10.4202/app.2008.0102

Sereno PC, Larsson HCE (2009) Cretaceous crocodyliforms from the Sahara. ZooKeys 28:1–143. https://doi.org/10.3897/zookeys.28.325

Sereno PC, ElShafie SJ (2013) A new long-necked turtle, *Laganemys tenerensis* (Pleurodira: Araripemydidae) from the Elrhaz Formation (Aptian-Albian) of Niger. In: Brinkman D (Ed) Morphology and Evolution of Turtles. Springer-Verlag, Frankfurt, 215–250. https://doi.org/10.1007/978-94-007-4309-0_14

Sereno PC, Wilson JA, Conrad JL (2004) New dinosaurs link southern landmasses in the mid-Cretaceous. Proceedings of the Royal Society Series B 271: 1325–1330. https://doi.org/10.1098/rspb.2004.2692

Sereno PC, Larsson HCE, Sidor CA, Gado B (2001) The giant crocodyliform *Sarcosuchus* from the Cretaceous of Africa. Science 294: 1516–1519. https://doi.org/10.1126/science.1066521

Sereno PC, Wilson JA, Larsson HCE, Dutheil DB, Sues H-D (1994) Early Cretaceous dinosaurs from the Sahara. Science 266: 267–271. https://doi.org/10.1126/science.266.5183.267

Sereno PC, Wilson JA, Witmer LM, Whitlock JA, Maga A, Ide O, Rowe TA (2007) Structural Extremes in a Cretaceous Dinosaur. PLoS ONE 2(11): e1230. https://doi.org/10.1371/journal.pone.0001230

Sereno PC, Dutheil DB, Iarochène M, Larsson HCE, Lyon GH, Magwene PM, Sidor CA, Varricchio DJ, Wilson JA (1996) Predatory dinosaurs from the Sahara and Late Cretaceous faunal differentiation. Science 272: 986–991. https://doi.org/10.1126/science.272.5264.986

Sereno PC, Beck AL, Dutheil DB, Larsson HCE, Lyon GH, Moussa B, Sadleir RW, Sidor CA, Varricchio DJ, Wilson GP, Wilson JA (1999) Cretaceous sauropods from the Sahara and the uneven rate of skeletal evolution among dinosaurs. Science 286: 1342–1347. https://doi.org/10.1126/science.286.5443.1342

Sereno PC, Beck AL, Dutheil DB, Gado B, Larsson HCE, Lyon GH, Marcot JD, Rauhut OWM, Sadleir RW, Sidor CA, Varricchio DJ, Wilson GP, Wilson JA (1998) A long-snouted predatory dinosaur from Africa and the evolution of spinosaurids. Science 282: 1298–1302. https://doi.org/10.1126/science.282.5392.1298

Sigogneau-Russell D (1995) Two possibly aquatic triconodont mammals from the Early Cretaceous of Morocco. Acta Palaeontologica Polonica 40: 149–162.

Simberloff D, Dayan T (1991) The guild concept and the structure of ecological communities. Annual Review of Ecology and Systematics 22: 115–143. https://doi.org/10.1146/annurev.es.22.110191.000555

Simões TR, Caldwell MW, Weinschütz LC, Wilner E, Kellner AWA (2017) Mesozoic lizards from Brazil and their role in early squamate evolution in South America. Journal of Herpetology 51: 307–315. https://doi.org/10.1670/16-007

Smith JB, Lamanna MC (2006) An abelisaurid from the Late Cretaceous of Egypt: implications for theropod biogeography. Naturwissenschaften 93: 242–245. https://doi.org/10.1007/s00114-006-0092-3

Smith JB, Lamanna MC, Mayr H, Lacovara KJ (2006) New information regarding the holotype of *Spinosaurus aegyptiacus* Stromer, 1915. Journal of Paleontology 80: 400–406. https://doi.org/10.1666/0022-3360(2006)080[0400:NIRTHO]2.0.CO;2

Smith JB, Lamanna MC, Lacovara KJ, Dodson P, Smith JR, Poole JC, Giegengack R, Attia Y (2001) A giant sauropod dinosaur from an Upper Cretaceous mangrove deposit in Egypt. Science 292: 1704–1706. https://doi.org/10.1126/science.1060561

Smyth RSH, Ibrahim N, Kao A, Martill DM (2020) Abelisauroid cervical vertebrae from the Cretaceous Kem Kem beds of Southern Morocco and a review of Kem Kem abelisauroids. Cretaceous Research 108: e104330. https://doi.org/10.1016/j.cretres.2019.104330

Soliman HE, Khalifa MA (1993) Stratigraphy, facies and depositional environments of the Lower Cenomanian Bahariya Formation, Bahariya Oasis, Western Desert, Egypt. Egyptian Journal of Geology 37: 193–209.

Stauch A, Blanc M (1962) Description d'un sélacien rajiforme des eaux douces du Nord-Cameroun: *Potamotrygon garouaensis* n. sp. Bulletin du Musuem national d'Histoire naturelle 34: 166–171.

Sternes PC, Shimada K (2019) Paleobiology of the Late Cretaceous sclerorhynchid sawfish, *Ischyrhizamira* (Elasmobranchii: Rajiformes), from North America based on new anatomical data. Historical Biology 31:1323–1340. https://doi.org/10.1080/08912963.2018.1452205

Stewart KM (1994) A late Miocene fish fauna from Lothagam, Kenya. Journal of Vertebrate Paleontology 14: 592–594. https://doi.org/10.1080/02724634.1995.10011580

Stromer E (1914) Ergebnisse der Forschungsreisen Prof. E. Stromers in dem Wüsten Ägyptens II, Wirbeltier-Reste der Baharije-Stufe (unterstes Cenomnan): Abhandlungen der Koniglich Bayerischen Akademie der Wissencschaften Mathematisch-physikalische Klasse 27: 3–16.

Stromer E (1915) Ergebnisse der Forschungsreisen Prof. E. Stromer in den Wüsten Agyptens. II. Wirbeltier-Reste der Baharîje-Stufe (unterstes Cenoman). 3. Das Original des Theropoden *Spinosaurus aegyptiacus* nov. gen., nov. spec. Abhandlungen der Königlich Bayerischen Akademie der Wissenschaften Mathematisch-Physikalische Klasse 28: 1–32.

Stromer E (1917) Ergebnisse der Forschungsreisen Prof. E. Stromers in den Wüsten Ägyptens. II. Wirbeltier-Reste der Baharije-Stufe (unterstes Cenoman). 4: Die Säge des Pristiden *Onchopristis numidus* Haug sp. und über die Sägen der Sägehaie. Abhandlungen der Königlich Bayerischen Akademie der Wissenschaften, Mathematisch-Naturwissenschaftliche Abteilung 28: 1–28.

Stromer E (1925) Ergebnisse der Forschungsreisen Prof. E. Stromers in den Wüsten Ägyptens. II. Wirbeltier-Reste der Baharije-Stufe (unterstes Cenoman). 7. *Stomatosuchus inermis* Stromer, ein schwach bezahnter Krokodilier und 8. Ein Skelettrest des Pristiden *Onchopristis numidus* Haug sp. Abhandlungen der Königlich Bayerischen Akademie der Wissenschaften, Mathematisch-Physikalische Klasse 30: 1–22.

Stromer E (1931) Ergebnisse der Forschungsrisen Prof. E. Stromers in den Wüsten Ägyptens. II. Wirbeltierreste der Baharje-Stufe (unterstes Cenoman). 10. Ein Skelett-Rest von *Carcharodontosaurus* nov. gen. Abhandlungen der Bayerischen Akademie der Wissenschaften Mathematisch-Naturwissenschaftliche Abteilung Neue Folge 9: 1–23.

Stromer E (1932) Wirbeltiere-Reste der Baharije-Stufe (unterstes Cenoman). 11. Sauropoda. Abhandlungen der Bayerischen Akademie der Wissenschaften Mathematisch naturwissenschafiliche Abteilung Neue Folge 10: 3–21.

Stromer E (1933) Ergebnisse der Forschungsreisen Prof. E. Stromers in den Wüsten Ägyptens. II. Wirbeltierreste der Baharîje-Stufe (unterstes Cenoman). 12. Die procölen Crocodilia.

Abhandlungen der Bayerischen Akademie der Wissenschaften Mathematisch-naturwissenschaftliche Abteilung Neue Folge 15: 1–31.

Stromer E (1934) Ergebnisse der Forschungsreisen Prof E. Stromers in den Wüsten Ägyptens. II. Wirbeltierreste der Baharje-Stufe (unterstes Cenoman). 13. Dinosauria von E. Stromer. Abhandlungen der Bayerischen Akademie der Wissenschaften Mathematisch- Naturwissenschaftliche Abteilung Neue Folge 22: 1–79.

Stromer E (1936) Ergebnisse der Forschungsreisen Prof. E. Stromers in den Wüsten Ägyptens. VII. Baharîje-Kessel und –Stufe mit deren Fauna und Flora. Eine ergänzende Zusammenfassung. Abhandlungen der Bayerischen Akademie der Wissenschaften Mathematisch Naturwissenschaftliche Abteilung 33: 1–102.

Tabaste N (1963) Etude de restes de poissons du Crétacé saharien. Mémoires de l'Institut Français d'Afrique Noire Mélanges ichthyologiques 68: 436–485.

Taquet P, Russell DA (1998) New data on spinosaurid dinosaurs from the Early Cretaceous of the Sahara. Comptes Rendus de l'Académie des Sciences à Paris Sciences de la Terre et des Planètes 327: 347–353. https://doi.org/10.1016/S1251-8050(98)80054-2

Taverne L (2000) Nouvelles données ostéologiques et phylogénétiques sur *Palaeonotopterus greenwoodi*, notoptéridé (Teleostei, Osteoglossomorpha) du Cénomanien inférieur continental (Crétacé) du Maroc. Stuttgarter Beilträge zur Naturkunde, Serie B (Geologie and Paläontologie) 293: 1–24.

Taverne L (2004) On a complete hyomandibular of the Cretaceous Moroccan notopterid *Palaeonotopterus greenwoodi* (Teleostei, Osteoglossomorpha). Stuttgarter Beilträge zur Naturkunde, Serie B (Geologie und Paläontologie) 348: 1–7.

Taverne L, Maisey JG (1999) A notopterid skull (Teleostei, Osteoglossomorpha) from the continental Early Cretaceous of Southern Morocco. American Museum Novitates 3260: 1–12.

Thorson TB (1970) Freshwater stingrays, *Potamotrygon* SPP.: failure to concentrate urea when exposed to saline medium. Life Sciences 9: 893–900. https://doi.org/10.1016/0024-3205(70)90059-7

Tong H, Buffetaut E (1996) A new genus and species of pleurodiran turtle from the Cretaceous of southern Morocco. Neues Jahrbuch für Geologie und Paläontologie Abhandlungen 199: 133–150. https://doi.org/10.1127/njgpa/199/1996/133

Torices A, Barroso-Barcenilla F, Cambra-Moo O, Pérez-García A (2012) Palaeontological and palaeobiogeographical implications of the new Cenomanian vertebrate site of Algora, Guadalajara, Spain. Cretaceous Research 237: 231–239. https://doi.org/10.1016/j.cretres.2012.04.004

Tucker ME (2011) Sedimentary Rocks in the Field, 4e. Wiley, Hoboken, New Jersey.

Unwin DM (2001) An overview of the pterosaur assemblage from the Cambridge Greensand (Cretaceous) of Eastern England. Mitteilungen Museum für Naturkunde Berlin, Geowissenschaftliche Reihe 4: 189–222. https://doi.org/10.1002/mmng.4860040112

Unwin DM, Deeming DC (2019) Pre-natal developmental patterns and their implications for super-precocial flight ability in pterosaurs. Proceedings Royal Society Series B 286: 201904. doi.org/10.1098/rspb.2019.0409 https://doi.org/10.1098/rspb.2019.0409

Veldmeijer AJ (2003) *Coloborhynchus spielbergi* sp. nov. (Pterodactyloidea) from the Albian (Lower Cretaceous) of Brazil. Scripta Geologica 125: 35–139.

Voigt S (2000) Cenomanian-Turonian composite ∂[13]C curve for Western and Central Europe: the role of organic and inorganic carbin fluxes. Palaeogeography, Palaeoclimatology, Palaeoecology 160: 91–104. https://doi.org/10.1016/S0031-0182(00)00060-2

Voigt S, Gale AS, Voigt T (2006) Sea-level change, carbon cycling and palaeoclimate during the Late Cenomanian of northwest Europe; an integrated palaeoenvironmental analysis. Cretaceous Research 27: 836–858. https://doi.org/10.1016/j.cretres.2006.04.005

Vullo R, Néraudeau D (2008) Cenomanian vertebrate assemblages from southwestern France, a new insight into the European mid-Cretaceous continental fauna. Cretaceous Research 29: 930–935. https://doi.org/10.1016/j.cretres.2008.05.010

Vullo R, Néraudeau D, Lenglet T (2007) Dinosaur teeth from the Cenomanian of Charentes, western France: evidence for a mixed Laurasian-Gondwanan assemblage. Journal of Vertebrate Paleontology 27: 931–943. https://doi.org/10.1671/0272-4634(2007)27[931:DTFTCO]2.0.CO;2

Vullo R, Guinot G, Barbe G (2016) The first articulated specimen of the Cretacoeus mackerel shark *Haimirichia amonensis* gen. nov. (Haimirichiidae fam. nov.) reveals a novel ecomorphological adaptation within the Lamniformes (Elasmobranchii). Journal of Systematic Palaeontology 14: 1003–1024. https://doi.org/10.1080/14772019.2015.1137983

Vullo R, Rage J-C (2018) The first Gondwanan borioteiioid lizard and the mid-Cretaceous dispersal event between North America and Africa. The Science of Nature 105: e61. https://doi.org/10.1007/s00114-018-1588-3

Vullo R, Néraudeau D, Allain R, Cappetta H (2005) Un nouveau gisement à microrestes de vertébrés continentaux et littoraux dans le Cénomanien inférieur de Fouras (Charente-Maritime, Sud-Ouest de la France). Comptes Rendus Palevol 4: 95–107. https://doi.org/10.1016/j.crpv.2004.11.006

Vullo R, Marugán-Lobón J, Kellner AWA, Buscalioni AD, Gomez B, de la Fuente M, Moratalla JJ (2012) A new crested pterosaur from the Early Cretaceous of Spain: the first european tapejarid (Pterodactyloidea: Azhdarchoidea). PLoS ONE 7(7): e38900. https://doi.org/10.1371/journal.pone.0038900

Walker JG, Geissman JW, Bowring SA, Babcock LE (compilers) (2018) Geologic Time Scale v. 5.0. Geological Society of America.

Wang X, Zhou Z (2003) Two new pterodactyloid pterosaurs from the Early Cretaceous Jiufotang Formation of Western Liaoning, China. Vertebrata PalAsiatica 41: 34–41.

Wang X, Kellner AW A, Jiang S, Cheng X (2012) New toothed flying reptile from Asia: close similarities between early Cretaceous pterosaur faunas from China and Brazil. Naturwissenschaften 99: 249–257. https://doi.org/10.1007/s00114-012-0889-1

Wang X, Kellner AWA, Jiang S, Wang Q, Ma Y, Paidoula Y, Cheng X, Rodrigues T, Meng X, Zhang J, Li N, Zhou Z (2014) Sexually dimorphic tridimensionally preserved pterosaurs and their eggs from China. Current Biology 24: 1323–1330. https://doi.org/10.1016/j.cub.2014.04.054

Weiler W (1935) Neue Untersuchungen an den Fischresten. In: Ergebnisse der Forschungsreisen Prof. E. Stromers in den Wüsten Ägyptens. II Wirbeltierreste der Baharije Stufe (unterstes Cenoman) 16 Abhandlungen der Bayerischen Akademie der Wissenschaften Mathematisch-Naturwissenschaftliche Abteilung 32: 1–57.

Weishampel DB, Dodson P, Osmólska H (Eds) (1990) The Dinosauria. University of California Press, Berkeley, 733 pp.

Weishampel DB, Dodson P, Osmólska H (Eds) The (2004) Dinosauria. University of California Press, Berkeley, 880 pp.

Wellnhofer P (1985) Neue Pterosaurier aus der Santana Formation (Apt) der Chapada do Araripe, Brasilien. Palaeontographica A 187: 105–182.

Wellnhofer P (1991) Weitere Pterosaurierfunde aus der Santana-Formation (Apt) der Chapada do Araripe, Brasilien. Palaeontographica A 215: 43–101.

Wellnhofer P, Buffetaut E (1999) Pterosaur remains from the Cretaceous of Morocco. Paläontologische Zeitschrift 73: 133–142. https://doi.org/10.1007/BF02987987

Wendler JE, Wendler I (2016) What drove sea-level fluctuations during the mid-Cretaceous greenhouse climate? Palaeogeography, Palaeoclimatology, Palaeoecology 441: 412–419. https://doi.org/10.1016/j.palaeo.2015.08.029

Wendt J (1985) Disintegration of the continental margin of northwestern Gondwana: Late Devonian of the eastern Anti-Atlas (Morocco). Geology 13: 815–818. https://doi.org/10.1130/0091-7613(1985)13<815:DOTCMO>2.0.CO;2

Wendt J, Aigner T, Neugebauer J (1984) Cephalopod limestone deposition on a shallow pelagic ridge: the Tafilalt Platform (upper Devonian, eastern Anti-Atlas, Morcco) Sedimentology 31: 601–625. https://doi.org/10.1111/j.1365-3091.1984.tb01226.x

Wenz S (1981) Un Coelacanthe géant *Mawsonia lavocati* Tabaste, de l'Albien-base du Cénomanien du Sud marocain. Annales de Paléontologie 67: 128–138.

Werner C (1989) Die Elasmobranchier Fauna des Gebel Dist member der Bahariya Formation (Obercenoman) der Oase Bahariya, Ägypten. Palaeo Ichthyologica 5: 1–112.

Werner C (1993) Eine neue Fundstelle terrestrischer Wirbeltiere aus der Kreide des Sudan. In: Kohring R (Ed) Miscellanea Palaeontologica 2. Berliner Geowissenschaften Abhandlungen, Reihe E9: 201–209.

Werner C (1995) Neue Funde von mesozoischen Wirbeltieren in Aethiopien. Berliner Geowissenschaftliche Abhandlungen A16: 377–383.

Werner C, Rage J-C (1994) Mid-Cretaceous snakes from Sudan. A preliminary report on an unexpectedly diverse snake fauna. Comptes Rendus de l'Académie des Sciences Paris 2: 247–252.

Werner C, Gayet M (1997) New fossil Polypteridae from the Cenomanian of Sudan. An evidence of their high diversity in the early Late Cretaceous. Cybium 21: 67–81.

Wilson JA, Upchurch P (2003) A revision of *Titanosaurus* Lydekker (Dinosauria – Sauropoda), the first dinosaur genus with a 'Gondwanan' distribution. Journal of Systematic Palaeontology 1: 125–160. https://doi.org/10.1017/S1477201903001044

Wilson JA, Allain R (2015) Osteology of *Rebbachisaurus garasbae* Lavocat, 1954, a diplodocoid (Dinosauria, Sauropoda) from the early Late Cretaceous – aged Kem Kem beds of southeastern Morocco. Journal of Vertebrate Paleontology 35: e1000701. https://doi.org/10.1080/02724634.2014.1000701

Wilson JA, Mohabey DM, Peters SE, Head JJ (2010) Predation upon hatchling dinosaurs by a new snake from the Late Cretaceous of India. PLoS Biology 8(3): e1000322. https://doi.org/10.1371/journal.pbio.1000322

Witton MP (2009) A new species of *Tupuxuara* (Thalassodromidae, Azhdarchoidea) from the Lower Cretaceous Santana Formation of Brazil, with a note on the nomenclature of Thalassodromidae. Cretaceous Research 30: 1293–1300. https://doi.org/10.1016/j.cretres.2009.07.006

Woodward AS (1907) On the Cretaceous formation of Bahia (Brazil), and on the vertebrate fossils collected therein. II. The vertebrate fossils. Quarterly Journal of the Geological Society 63: 131–139. https://doi.org/10.1144/GSL.JGS.1907.063.01-04.11

Yabumoto Y, Uyeno T (2005) New materials of a Cretaceous coelacanth, *Mawsonia lavocati* Tabaste from Morocco. Bulletin of the National Science Museum Tokyo 31: 39–49.

Young MT, Hastings AK, Allain R, Smith TJ (2017) Revision of the enigmatic crocodyliform *Elosuchus felixi* Lapparent de Broin, 2002 from the Lower-Upper Cretaceous boundary of Niger: potential evidence for an early origin of the clade Dyrosauridae. Zoological Journal of the Linnean Society 179: 377–403. https://doi.org/10.1111/zoj.12452

Zitouni S, Laurent C, Dyke G, Jalil N-E (2019) An abelisaurid (Dinosauria: Theropoda) ilium from the Upper Cretaceous (Cenomanian) of the Kem Kem beds, Morocco. PLoS ONE 14(4): e0214055. https://doi.org/10.1371/journal.pone.0214055
